# Supplementary material for: 3,3-Difluoroallyl ammonium salts: highly versatile, stable and selective gem-difluoroallylation reagents
Source: Nat Commun. 2021 May 31;12:3257. doi: 10.1038/s41467-021-23504-2 (PMC8167079; doi:10.1038/s41467-021-23504-2)
Supplement: Supplementary file 1 — Supplementary Information [file 41467_2021_23504_MOESM1_ESM.pdf]

Supplementary Information

3,3-Difluoroallyl Ammonium Salts: Highly Versatile, Stable and Selective *gem*-Difluoroallylation Reagents

Fei Ye[†], Yao, Ge[†], Anke Spannenberg, Helfried Neumann, Li-Wen Xu, and Matthias Beller*

* Correspondence to: matthias.beller@catalysis.de

Table of contents

Table of contents 1
1. General information 2
2. Experimental section 3
2.1 Chemical list 3
2.2 General procedure for the synthesis of ammonium salts 1: 5
2.3 General procedure for the preparation of gem-difluoroallyl substituted compound 3-60: 7
2.4 Optimization of reaction conditions 8
3. Characterization of new compounds 11
4. X-ray crystal structure analysis of compound 44 and 70 57
5. ^{1}H, ^{13}C, ^{19}F NMR spectra for new compounds 59
6. HPLC spectra for compound 66 187

1. General information

All reactions were carried out under an atmosphere of dry argon using standard Schlenk technique or Ace pressure tube. Chemicals were purchased from *Sigma-Aldrich*, *TCI*, *Alfa Aesar*, *Fluorochem* or *ABCR*. Toluene, DMF, DCM, THF were dried and degassed and stored in ©Aldrich Sure/Store flasks under argon. Other chemicals were used as received. Multiplets of NMR were assigned as s (singlet), d (doublet), t (triplet), dd (doublet of doublet), dt (doublet of triplet), td (triplet of doublet), m (multiplet), and br. s (broad singlet). NMR data were recorded on Bruker Avance 300 or Bruker ARX 400 spectrometers at room temperature. ^{1}H and ^{13}C NMR spectra were referenced to signals of deuterated solvents and residual protiated solvents, respectively. ^{19}F NMR spectra were not referenced to signals of fluorinated internal standard. High resolution mass spectra (HRMS) were recorded on Agilent 6210. The data are given as mass units per charge (m/z).

2. Experimental section

2.1 Chemical list

Compound S1:

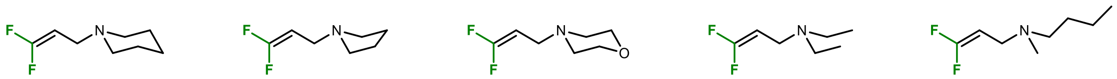

S1a **S1b** **S1c** **S1d** **S1e**

Compound 1:

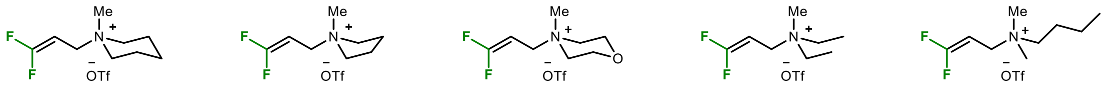

1a **1b** **1c** **1d** **1e**

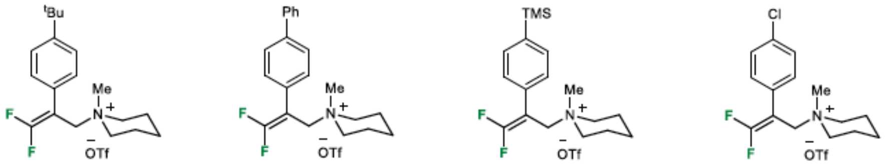

1f **1g** **1h** **1i**

Compound 2:

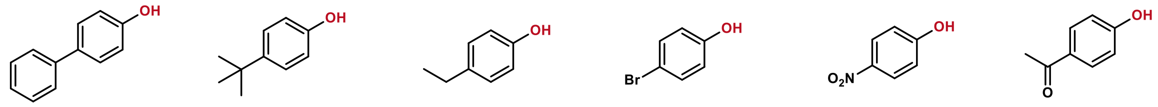

2a **2b** **2c** **2d** **2e** **2f**

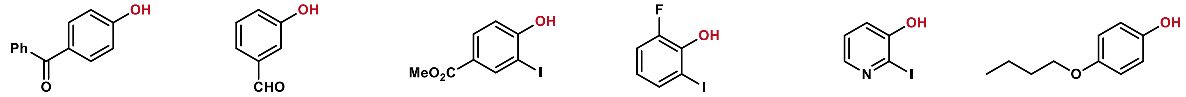

2g **2h** **2i** **2j** **2k** **2l**

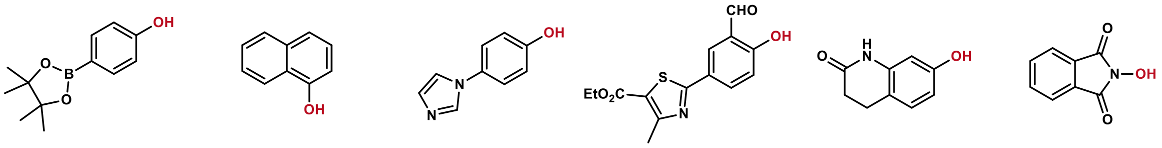

2m **2n** **2o** **2p** **2q** **2r**

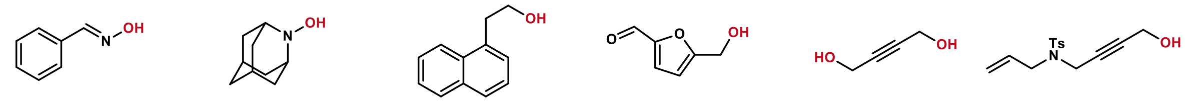

2s **2t** **2u** **2v** **2w** **2x**

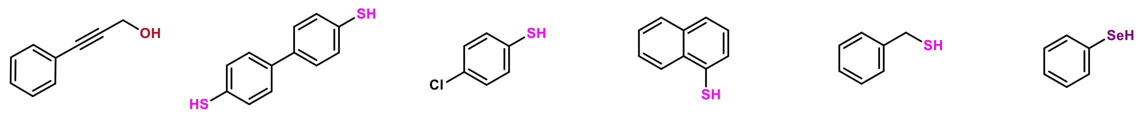

2y **2z** **2aa** **2ab** **2ac** **2ad**

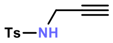

2ae

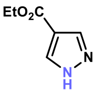

2af

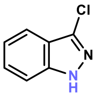

2ag

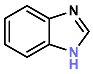

2ah

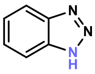

2ai

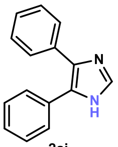

2aj

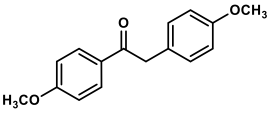

2ak

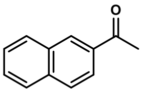

2al

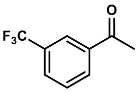

2am

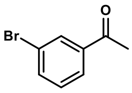

2an

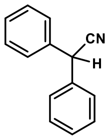

2ao

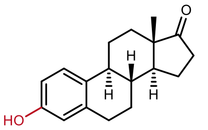

2ap

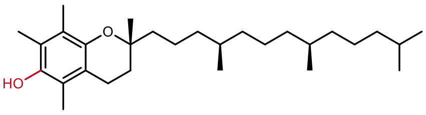

2aq

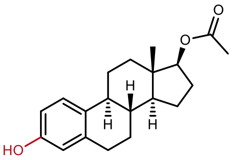

2ar

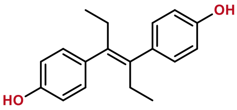

2as

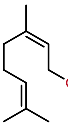

2at

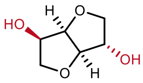

2au

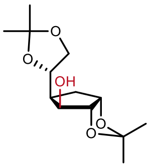

2av

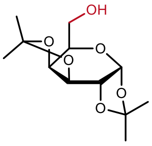

2aw

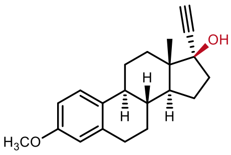

2ax

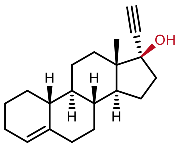

2ay

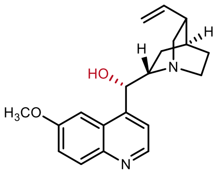

2az

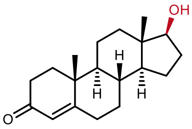

2ba

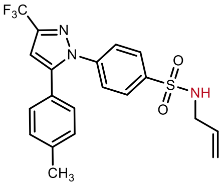

2bb

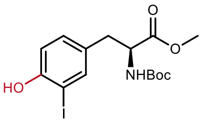

2bc

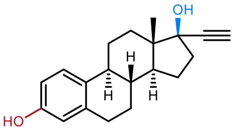

2bd

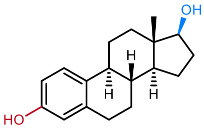

2be

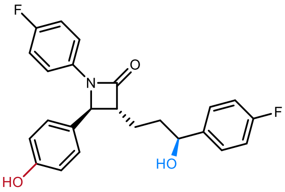

2bf

*2.2 General procedure for the synthesis of ammonium salts **1:***

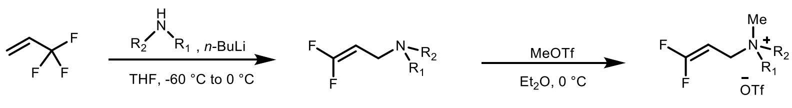

Procedure A: Under protection of argon, a 100 mL oven-dried flask equipped with a stirring bar was charged with piperidine (30 mmol, 1.0 equiv) and anhydrous THF (30 mL). The flask was cooled to -60 °C using a dry ice/ethanol bath and after cooling for 10 min, a 2.5 M solution of *n*-BuLi (12 mL, 1.0 equiv) in hexanes was added dropwise to the flask. The resulting mixture was stirred at -60 °C for 1 h, then warmed to 0 °C in an ice-water bath and stirred for additional 1 h. Another 250 mL oven-dried flask was charged with 3,3,3-trifluoropropene stock solution (47 mL, 0.83 M in THF, 1.3 equiv) and cooled to -60 °C, followed by dropwise addition of the previously prepared lithium amide solution through a needle over 1 h. The reaction mixture was slowly warm to room temperature and stirred for additional 12 h before quenching with water (20 mL). After evaporate the major solvent, the organic layer was extracted with diethyl ether (×3), washed with brine, dried over Na_2SO_4, filtered, and concentrated under reduced pressure. The residue was purified by vacuum distillation to afford the desired compounds **S1**.

Large scale protocol: Under protection of argon, a 250 mL oven-dried flask equipped with a stirring bar was charged with piperidine (8.6 g, 100 mmol, 1.0 equiv) and anhydrous THF (100 Ml, 1.0 M). The flask was cooled to -60 °C using a dry ice/ethanol bath and after cooling for 10 min, a 2.5 M solution of *n*-BuLi (40 mL, 100 mmol, 2.5 M in hexanes, 1.0 equiv) was added dropwise to the flask. The resulting mixture was stirred at -60 °C for 1 h, then warmed to 0 °C in an ice-water bath and stirred for additional 1 h (details see Supplementary Figure 1.). Another 500 mL oven-dried flask was charged with trifluoropropene stock solution (133 mL, 130 mmol, 0.98 M in THF, 1.3 equiv) and cooled to -60 °C, followed by dropwise addition of the previously prepared lithium amide solution using dropping funnel over 2 h. The reaction mixture was slowly warm to room temperature and stirred for additional 5 h before quenching with water (20 mL). The organic solvent was first evaporated under reduced pressure, then the organic layer was extracted with diethyl ether (×3), washed with brine, dried over Na_2SO_4, filtered, and concentrated under reduced pressure. The residue was purified by vacuum distillation (detailed see Supplementary Figure 2., oil bath temperature is 60 °C, steam temperature is 42 °C, vacuum is 20 mbar) to afford the desired compounds **S1a**.

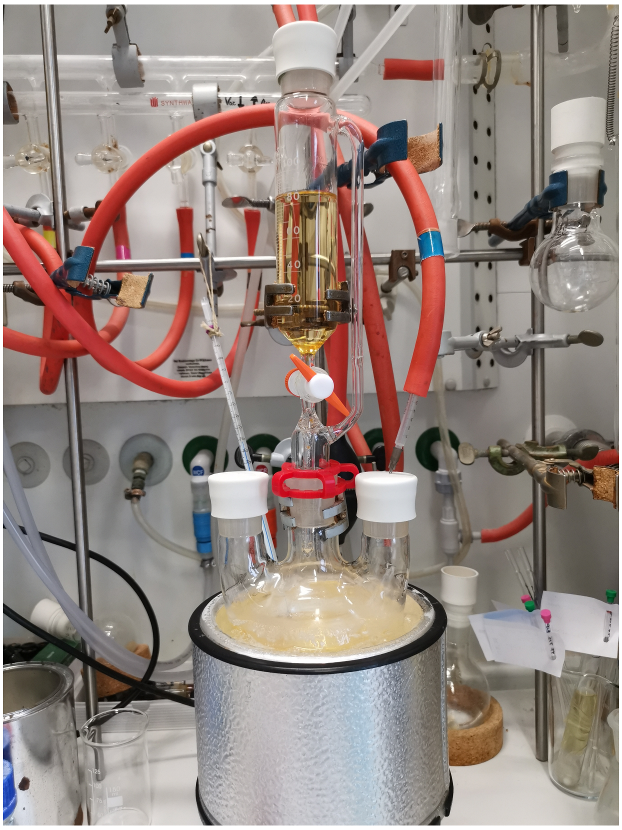

Supplementary Figure 1. Large scale synthesis of compound S1a

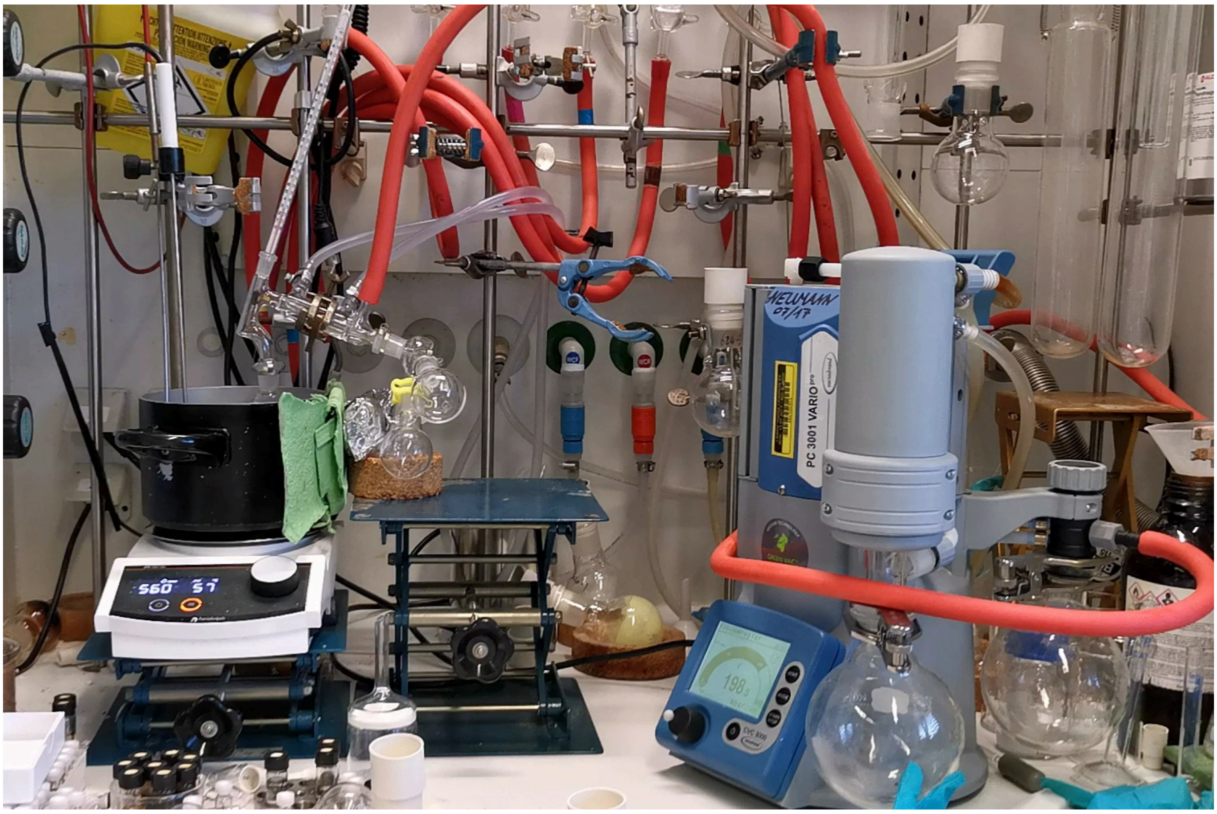

Supplementary Figure 2. Vacuum distillation of compound S1a

Procedure B: To a Schlenk tube equipped with a magnetic stir bar were added amine **S1** (1 equiv) and dry Et_2O (0.2 M). The flask was cooled to 0 °C using an ice-water bath, MeOTf (1.3 equiv) was added dropwise to the flask over 5 min. The solution was stirred at 0 °C for 1-2 h and gradually became cloudy and white. The solution was filtrated and washed with dry Et_2O (3 × 10 mL). Drying under vacuum for 5 h to give the pure products **1a-1e**.

*2.3 General procedure for the preparation of gem-difluoroallyl substituted compound **3-60**:*

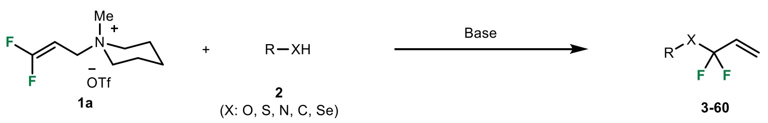

Procedure C: To a 25 mL oven-dried pressure tube equipped with a magnetic stir bar were added difluoroallyl ammonium salt **1** (0.36 mmol, 1.2 equiv), phenol **2** (0.3 mmol, 1 equiv), NaH (60 % dispersion in mineral oil, 0.45 mmol, 1.5 equiv), and then degassed DMF (1.5 mL) was introduced under argon atmosphere. The sealed pressure tube was stirred at room temperature for 30 min before quenched with 1 mL water. The resulting mixture was diluted with ethyl acetate (5 mL), followed by the addition of 1,4-difluorobenzene (0.2 mmol, 19.5 µL) as internal standard. Then 250 µL of the solution was taken to measure the conversion, yield and regioselectivity by ^{19}F NMR. After, 5 mL water was introduced and the organic layer was extracted with diethyl ether (×3), washed with brine, dried over Na_2SO_4, filtered, and concentrated under reduced pressure. The residue was purified by chromatography on silica gel (It is worthy to note that some fluorinated products are unstable and should be purified using 2% Et_3N as the eluent) to afford the desired product.

Procedure D: To a pressure tube (25 mL) equipped with a magnetic stir bar difluoroallyl ammonium salt **1** (0.36 mmol), Cs_2CO_3 (0.15 mmol), toluene (2.5 mL), and then nucleophiles **2** (0.3 mmol) were added. The sealed pressure tube was vigorously stirred for 18 h at 50 °C under air atmosphere. After cooling to room temperature, the reaction mixture was diluted with ethyl acetate (10 mL) and filtered through a short pad of celite eluting with ethyl acetate (3 × 10 mL). After evaporation, the residue was purified by chromatography on silica gel to afford the desired product. Notably, some of the fluorinated products can only be separated without decomposition with 2% triethylamine in the eluent.

2.4 Optimization of reaction conditions

Supplementary Table 1. The effect of different parameters for the synthesis of compound 3.

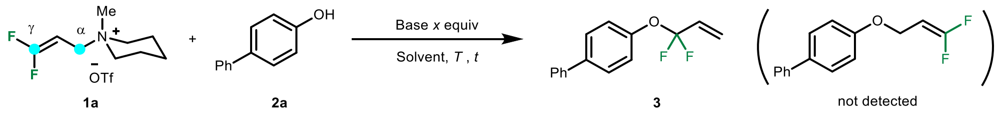

Entry[a]	Base (eq.)	Solv. (con.)	*T* (°C)	*t* (h)	3 (%)
1[b,c]	Cs_2CO_3 (1.0)	Toluene (0.1 M)	80	18	92
2	Cs_2CO_3 (1.0)	Toluene (0.1 M)	80	18	87
3	Cs_2CO_3 (0.5)	Toluene (0.1 M)	80	18	96
**4**	**Cs_2CO_3 (0.5)**	**Toluene (0.1 M)**	**50**	**18**	**>99**
5	Cs_2CO_3 (0.2)	Toluene (0.1 M)	80	18	59
6	Cs_2CO_3 (1.2)	H_2O/THF (9:1, 0.1 M)	50	18	97
7	Cs_2CO_3 (1.0)	H_2O/THF (9:1, 0.1 M)	23	18	63
**8**	**Cs_2CO_3 (0.5)**	**H_2O/THF (9:1, 0.1 M)**	**80**	**18**	**94**
9	Cs_2CO_3 (0.5)	H_2O/THF (9:1, 0.1 M)	50	18	86
10	K_2CO_3 (1.0)	H_2O/THF (9:1, 0.1 M)	80	18	84
11	K_2CO_3 (1.0)	H_2O/THF (9:1, 0.1 M)	50	18	77
12	K_2CO_3 (1.0)	H_2O/THF (9:1, 0.1 M)	23	18	49
13	KOH (1.0)	H_2O/THF (9:1, 0.1 M)	23	18	72
14	KOH (1.2)	H_2O/THF (9:1, 0.1 M)	50	18	90
15	KOH (1.2)	H_2O/THF (9:1, 0.1 M)	80	18	90
16	NaOH (1.0)	H_2O/THF (9:1, 0.1 M)	23	18	64
17	^{t}BuOK (1.0)	H_2O/THF (9:1, 0.1 M)	23	18	65
18	DIEA (1.0)	CH_2Cl_2 (0.1 M)	80	18	86
**19[c]**	**NaH(1.5)**	**DMF (0.2 M)**	**23**	**0.5**	**>99**

[a] Reaction conditions: **1a** (0.24 mmol), **2** (0.2 mmol), base (x equiv), and solvent were stirred under air atmosphere, the yield was determined by ^{19}F-NMR using 1,4-difluorobenzene as internal standard. [b] $PdBr_2$ (5 mol%) and $^{n}BuPAd_2$ (10 mol%) were used. [c] The reaction was performed under argon atmosphere.

Supplementary Table 2. Screening different gem-difluorinated allyl ammonium salts 1.

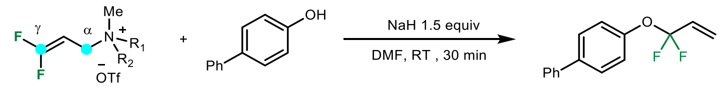

1 2a 3

Entry[a]	1		3 (%)
1	**1a**	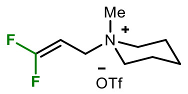	>99
2	**1b**	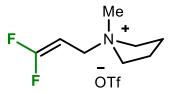	97
3	**1c**	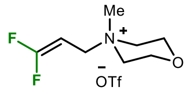	97
4	**1d**	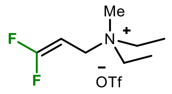	98
5	**1e**	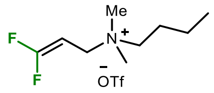	90

[a] Reaction conditions: The mixture of **1** (0.36 mmol), **2a** (0.3 mmol), NaH (1.5 equiv), and DMF (1.5 mL) was stirred under argon atmosphere, the yield was determined by ^{19}F-NMR using 1,4-difluorobenzene as internal standard.

Supplementary Table 3. The effect of different parameters for the synthesis of compound 23.

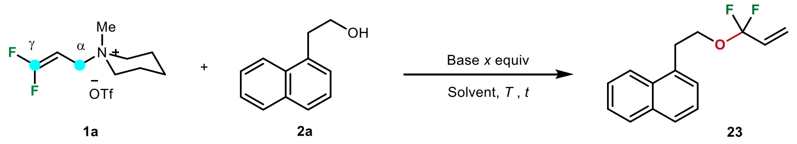

Entry[a]	Base (eq.)	Solv. (con.)	*T* (°C)	*t* (h)	3 (%)
1[b]	Cs_2CO_3 (1.0)	Toluene (0.05 M)	80	18	12
2[b]	Cs_2CO_3 (1.0)	H_2O/THF (1:1, 0.05 M)	80	18	NR
3	^{t}BuOK (1.5)	DMF (0.1 M)	80	18	62
4	^{t}BuONa (1.5)	DMF (0.1 M)	80	18	90
5	NaH (1.0)	Toluene (0.05 M)	80	18	58
6	NaH (1.5)	DMF (0.1 M)	80	18	>99
7	KHMDS (1.2)	DMF (0.1 M)	80	18	22
8	NaOEt (1.5)	DMF (0.1 M)	80	18	28
9	NaOMe (1.5)	DMF (0.1 M)	80	18	54

[a] Reaction conditions: **1a** (0.24 mmol), **2x** (0.2 mmol), base (x equiv), and solvent were stirred under argon atmosphere, the yield was determined by ^{19}F-NMR using 1,4-difluorobenzene as internal standard. [b] The reaction was performed under air atmosphere.

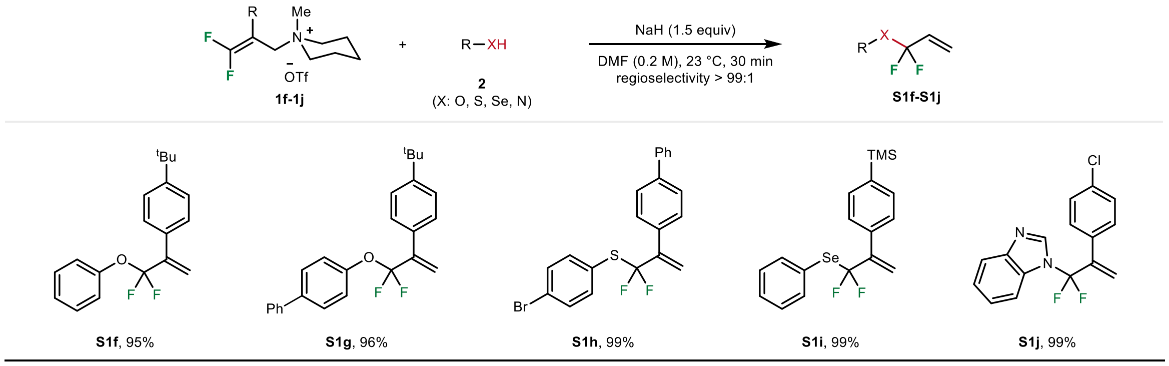

Supplementary Figure 3. Regioselective substitution of aryl-substituted 3,3-difluoroallyl ammonium salts 1f-1j by different O-, N-, S-, and Se-nucleophiles: Substrate scope.

3. Characterization of new compounds

1-(3,3-Difluoroallyl)piperidine (S1a)

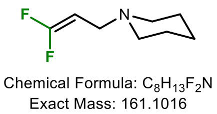

This compound was obtained following the general procedure A. Starting from 3,3,3-trifluropropene (0.83 M in THF, 156 mL, 130 mmol), pieridine (10 mL, 100 mmol), *n*-butyl lithium (2.5 M in hexane, 100 mmol, 1.0 equiv) and THF (200 mL). Purification by vacuum distillation (oil bath temperature is 60 °C, steam temperature is 42 °C, vacuum is 20 mbar) to afford the desired compounds **S1a** as a colorless oil (9.0 g, 56%).

^{1}H NMR (300 MHz, $CDCl_3$) δ 4.33 (dtd, *J* = 24.9, 7.9, 2.6 Hz, 1H), 2.95 (ddd, *J* = 7.8, 2.1, 1.7 Hz, 2H), 2.38 (t, *J* = 5.4 Hz, 4H), 1.64 – 1.51 (m, 4H), 1.49 – 1.34 (m, 2H).

^{13}C NMR (75 MHz, $CDCl_3$) δ 157.4 (dd, *J* = 289.0, 288.0 Hz), 75.5 (dd, *J* = 21.0, 18.6 Hz), 54.0, 51.5 (d, *J* = 6.0 Hz), 26.0, 24.3.

^{19}F NMR (282 MHz, $CDCl_3$) δ -86.0 (dd, *J* = 41.7, 2.4 Hz, 1F), -88.9 (dd, *J* = 41.7, 24.7 Hz, 1F).

HRMS (ESI): calculated for $C_8H_{14}F_2N$ $[M+H]^+$: 162.1094, found: 162.1095.

1-(3,3-Difluoroallyl)pyrrolidine (S1b)

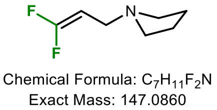

This compound was obtained following the general procedure A. Starting from 3,3,3-trifluropropene (0.8 M in THF, 49 mL, 39 mmol), pyrrolidine (2.1 g, 30 mmol), *n*-butyl lithium (1.5 M in hexane, 30 mmol, 1.0 equiv) and THF (100 mL). Purification by vacuum distillation (oil bath temperature is 60 °C, steam temperature is 41 °C, vacuum is 40 mbar) to afford the desired compounds **S1b** as a colorless oil (1.6 g, 36%).

^{1}H NMR (300 MHz, $CDCl_3$) δ 4.38 (dtd, *J* = 24.7, 7.8, 2.4 Hz, 1H), 3.09 (ddd, *J* = 7.9, 2.2, 1.7 Hz, 2H), 2.57 – 2.46 (m, 4H), 1.82 – 1.76 (m, 4H).

^{13}C NMR (75 MHz, $CDCl_3$) δ 157.2 (dd, J = 287.3, 285.0 Hz), 76.4 (dd, *J* = 21.8, 18.4 Hz), 68.1, 48.0 (d, *J* = 6.5 Hz), 25.7.

^{19}F NMR (282 MHz, $CDCl_3$) δ -86.7 (d, *J* = 41.6 Hz, 1F), -88.7 (dd, *J* = 41.6, 24.6 Hz, 1F).

HRMS (ESI): calculated for $C_7H_{12}F_2N$ $[M+H]^+$: 148.0938, found: 148.0942.

4-(3,3-Difluoroallyl)morpholine (S1c)

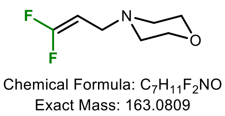

This compound was obtained following the general procedure A. Starting from 3,3,3-trifluropropene (0.8 M in THF, 49 mL, 39 mmol), morphine (2.6 g, 30 mmol), *n*-butyl lithium (1.5 M in hexane, 30 mmol, 1.0 equiv) and THF (100 mL). Purification by vacuum distillation (oil bath temperature is 65 °C, steam temperature is 48 °C, vacuum is 14 mbar) to afford the desired compounds **S1c** as a colorless oil (2.6 g, 53%).

^{1}H NMR (300 MHz, $CDCl_3$) δ 4.31 (dtd, *J* = 24.7, 7.9, 2.4 Hz, 1H), 3.78 – 3.59 (m, 4H), 2.98 (ddd, *J* = 7.9, 2.1, 1.6 Hz, 2H), 2.53 – 2.36 (m, 4H).

^{13}C NMR (75 MHz, $CDCl_3$) δ 157.6 (dd, *J* = 288.0, 286.5 Hz), 74.9 (dd, *J* = 21.9, 18.4 Hz), 67.0, 53.1, 51.2 (d, *J* = 6.5 Hz).

^{19}F NMR (282 MHz, $CDCl_3$) δ -85.2 (d, *J* = 39.7 Hz, 1F), -88.1 (dd, *J* = 39.7, 24.6 Hz, 1F).

HRMS (ESI): calculated for $C_7H_{12}F_2NO$ $[M+H]^+$: 164.0887, found: 164.0889.

***N,N*-diethyl-3,3-difluoroprop-2-en-1-amine (S1d)**

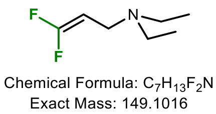

This compound was obtained following the general procedure A. Starting from 3,3,3-trifluropropene (1.0 M in THF, 39 mL, 39 mmol), diethylamine (2.19 g, 30 mmol), *n*-butyl lithium (2.2 M in hexane, 30 mmol, 1.0 equiv) and THF (100 mL). Purification by vacuum distillation (oil bath temperature is 60 °C, steam temperature is 42 °C, vacuum is 80 mbar) to afford the desired compounds **S1d** as a colorless oil (0.9 g, 20%).

^{1}H NMR (300 MHz, $CDCl_3$) δ 4.27 (dtd, *J* = 25.2, 7.7, 2.7 Hz, 1H), 3.12 (dt, *J* = 7.8, 1.9 Hz, 2H), 2.48 (q, *J* = 7.2 Hz, 4H), 1.02 (t, *J* = 7.2 Hz, 6H).

^{13}C NMR (75 MHz, $CDCl_3$) δ 157.2 (t, *J* = 288.4 Hz), 74.9 (t, *J* = 19.3 Hz), 68.0, 44.9 (d, *J* = 5.8 Hz), 25.7.

^{19}F NMR (282 MHz, $CDCl_3$) δ -85.9 (dd, *J* = 43.4, 2.6 Hz, 1F), -89.1 (dd, *J* = 43.0, 25.1 Hz, 1F).

HRMS (ESI): calculated for $C_7H_{14}F_2N$ $[M+H]^+$: 150.1094, found: 150.1096.

***N*-(3,3-difluoroallyl)-N-methylbutan-1-amine (1e)**

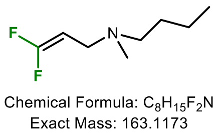

This compound was obtained following the general procedure A. Starting from 3,3,3-

trifluropropene (1.0 M in THF, 39 mL, 39 mmol), *N*-methylbutylamine (2.61 g, 30 mmol), *n*-butyl lithium (2.2 M in hexane, 30 mmol, 1.0 equiv) and THF (100 mL). Purification by vacuum distillation (oil bath temperature is 60 °C, steam temperature is 42 °C, vacuum is 30 mbar) to afford the desired compounds **S1d** as a colorless oil (1.38 g, 28%).

^{1}H NMR (300 MHz, Chloroform-*d*) δ 4.30 (dtd, *J* = 25.0, 7.8, 2.6 Hz, 1H), 3.01 (dt, *J* = 7.8, 1.9 Hz, 2H), 2.36 – 2.26 (m, 2H), 2.20 (s, 3H), 1.51 – 1.36 (m, 2H), 1.36 – 1.23 (m, 2H), 0.90 (t, *J* = 7.2 Hz, 3H).

^{13}C NMR (75 MHz, $CDCl_3$) δ 157.4 (dd, *J* = 287.3, 286.5 Hz), 75.3 (dd, *J* = 20.3, 18.8 Hz), 56.6, 49.9 (d, *J* = 5.9 Hz), 41.7, 29.7, 20.8, 14.1.

^{19}F NMR (282 MHz, Chloroform-*d*) δ -85.99 (dd, *J* = 42.0, 2.4 Hz, 1F), -88.89 (dd, *J* = 42.0, 25.0 Hz, 1F).

HRMS (ESI): calculated for $C_8H_{16}F_2N$ $[M+H]^+$: 164.1250, found: 164.1253.

Compound 1a

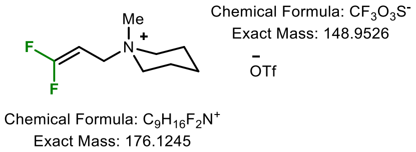

This compound was obtained following the general procedure B. Starting from amine **S1a** (9.0 g, 60 mmol), methyl trifluoromethanesulfonate (12.8 g, 78 mmol, 1.3 equiv), and diethyl ether (100 mL). Filtration to afford **1a** (19.0 g, 100%) as a white solid. M.p. 58–59 °C.

^{1}H NMR (300 MHz, Acetone-d_6) δ 5.06 (dtd, *J* = 24.1, 8.7, 1.6 Hz, 1H), 4.24 (dt, *J* = 8.6, 1.3 Hz, 2H), 3.58 (t, *J* = 5.8 Hz, 4H), 3.23 (s, 3H), 2.04 – 1.91 (m, 4H), 1.82 – 1.65 (m, 2H).

^{13}C NMR (75 MHz, Acetone- d_6) δ 160.3 (t, *J* = 294.0 Hz), 120.1 (t, *J* = 319.5 Hz), 70.2 (dd, *J* = 30.2, 16.4 Hz), 61.1, 58.2, 47.5, 21.5, 20.6.

^{19}F NMR (282 MHz, Acetone-d_6) δ -78.9 (d, *J* = 21.4 Hz, 1F), -79.0 (s, 3F), -81.5 – -81.7 (m, 1F).

HRMS (ESI): calculated for $C_9H_{16}F_2N$ $[M-OTf]^+$: 176.1256, found: 176.1257.

Compound 1b

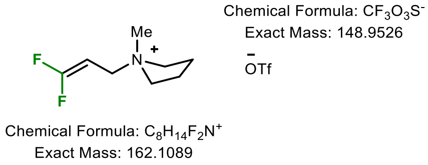

This compound was obtained following the general procedure B. Starting from amine **S1b** (1.6 g, 10.9 mmol), methyl trifluoromethanesulfonate (2.3 g, 14.1 mmol, 1.3 equiv), and diethyl ether (30 mL). Filtration to afford **1b** (3,2 g, 96%) as a white solid. M.p. 44–45 °C.

^{1}H NMR (300 MHz, Acetone-d_6) δ 5.11 (dtd, *J* = 24.0, 8.6, 1.6 Hz, 1H), 4.26 (dt, *J* = 8.6, 1.3 Hz, 2H), 3.80 – 3.68 (m, 4H), 3.26 (s, 3H), 2.36 – 2.27 (m, 4H).

^{13}C NMR (75 MHz, Acetone-d_6) δ 162.2 (d, *J* = 293.3 Hz), 122.2 (d, *J* = 321.4 Hz), 71.5 (dd, *J* = 29.9, 16.4 Hz 64.4 – 64.0 (m), 57.52 (dd, *J* = 8.0, 3.9 Hz), 49.3 – 48.2 (m), 22.3.

^{19}F NMR (282 MHz, Acetone-d_6) δ -79.0 (s, 3F), -79.9 (dd, *J* = 22.8, 1.4 Hz, 1F), -82.3 (t, *J* = 23.4 Hz, 1F).

HRMS (ESI): calculated for $C_8H_{14}F_2N$ $[M\text{-}OTf]^+$: 162.1099, found: 162.1099.

Compound 1c

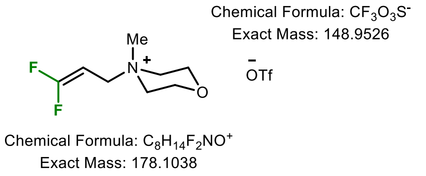

This compound was obtained following the general procedure B. Starting from amine **S1c** (2.4 g, 14.7 mmol), methyl trifluoromethanesulfonate (3.1 g, 19.1 mmol, 1.3 equiv), and diethyl ether (30 mL). Filtration to afford **1c** (4.3 g, 86%) as a white solid. M.p. 67–68 °C.

^{1}H NMR (300 MHz, Acetone-d_6) δ 5.10 (dtd, *J* = 24.1, 8.7, 1.5 Hz, 1H), 4.39 (dt, *J* = 8.7, 1.3 Hz, 2H), 4.19 – 4.03 (m, 4H), 3.77 – 3.61 (m, 4H), 3.39 (s, 3H).

^{13}C NMR (75 MHz, Acetone-d_6) δ 162.4 (d, *J* = 292.2 Hz), 122.2 (d, *J* = 321.4 Hz), 69.9 (dd, *J* = 30.5, 16.2 Hz), 61.2, 59.9, 59.4, 46.9.

^{19}F NMR (282 MHz, Acetone-d_6) δ -78.3 (d, *J* = 19.5 Hz, 1F), -79.1 (s, 3F), -80.9 (dd, *J* = 24.1, 19.5 Hz, 1F).

HRMS (ESI): calculated for $C_8H_{14}F_2NO$ $[M\text{-}OTf]^+$: 178.1043, found: 178.1039.

Compound 1d

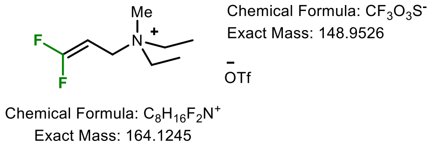

This compound was obtained following the general procedure B. Starting from amine **S1d** (0.9 g, 6 mmol), methyl trifluoromethanesulfonate (1.26 g, 7.8 mmol, 1.3 equiv), and diethyl ether (20 mL). Filtration to afford **1a** (1.86 g, 100%) as a colorless oil.

^{1}H NMR (300 MHz, Acetone-d_6) δ 5.05 (dtd, *J* = 24.0, 8.6, 1.7 Hz, 1H), 4.18 (dt, *J* = 8.6, 1.3 Hz, 2H), 3.58 (qd, *J* = 7.3, 1.1 Hz, 4H), 3.18 (s, 3H), 1.44 (tt, *J* = 7.3, 1.8 Hz, 6H).

^{13}C NMR (75 MHz, Acetone-d_6) δ 162.2 (d, *J* = 289.5 Hz), 122.3 (d, *J* = 319.5 Hz), 70.5 (dd, *J* = 30.2, 16.2 Hz), 56.77 (t, *J* = 2.9 Hz), 55.5 – 55.2 (m), 47.1 (t, *J* = 4.1 Hz), 8.1.

^{19}F NMR (282 MHz, Acetone-d_6) δ -79.0 (s, 3F), -79.1 (d, J = 22.1 Hz, 1F), -81.7 (t, J = 23.0 Hz, 1F).

HRMS (ESI): calculated for $C_8H_{16}F_2N$ [M-OTf]$^+$: 164.1256, found: 164.1257.

Compound 1e

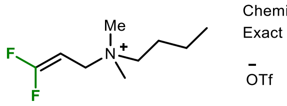

Chemical Formula: $CF_3O_3S^-$
Exact Mass: 148.9526

Chemical Formula: $C_9H_{18}F_2N^+$
Exact Mass: 178.1402

This compound was obtained following the general procedure B. Starting from amine **S1e** (0.96 g, 5.9 mmol), methyl trifluoromethanesulfonate (1.2 g, 7.8 mmol, 1.3 equiv), and diethyl ether (20 mL). Filtration to afford **1e** (1.93 g, 100%) as a slight yellow oil.

^{1}H NMR (300 MHz, Acetone-d_6) δ 5.08 (dtd, J = 24.0, 8.6, 1.6 Hz, 1H), 4.23 (dt, J = 8.6, 1.3 Hz, 2H), 3.57 – 3.48 (m, 2H), 3.28 (s, 6H), 1.96 – 1.81 (m, 2H), 1.50 – 1.36 (m, 2H), 0.98 (t, J = 7.4 Hz, 3H).

^{13}C NMR (75 MHz, Acetone-d_6) δ 122.0 (d, J = 318.0 Hz), 70.7 (dd, J = 30.2, 16.4 Hz), 64.5 – 64.4 (m), 58.9 – 58.4 (m), 50.6 – 50.2 (m), 25.1, 20.2, 13.8.

^{19}F NMR (282 MHz, Acetone-d_6) δ -78.9 (d, J = 21.1 Hz, 1F), -79.0 (s, 3F), -81.6 – -81.8 (m, 1F).

HRMS (ESI): calculated for $C_9H_{18}F_2N$ [M-OTf]$^+$: 178.1412, found: 178.1408.

4-((1,1-Difluoroallyl)oxy)-1,1'-biphenyl (3)

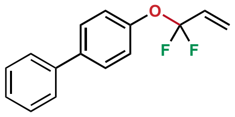

Chemical Formula: $C_{15}H_{12}F_2O$
Exact Mass: 246.0856

This compound was obtained following the general procedure C. Starting from ammonium salt **1a** (117 mg, 0.36 mmol, 1.2 equiv), [1,1'-biphenyl]-4-ol **2a** (51 mg, 0.3 mmol), NaH (60% dispersion in mineral oil, 18 mg, 0.45 mmol, 1.5 equiv), and DMF (1.5 mL). Purification on silica gel (Pentane/Ethyl acetate gradient from 100/0 to 98/2) afforded **3** (73 mg, 99%) as a colorless oil.

^{1}H NMR (300 MHz, $CDCl_3$-d) δ 7.61 – 7.53 (m, 4H), 7.49 – 7.41 (m, 2H), 7.39 – 7.32 (m, 1H), 7.32 – 7.26 (m, 2H), 6.18 – 6.01 (m, 1H), 6.01 – 5.91 (m, 1H), 5.62 (dd, J = 10.4, 1.0 Hz, 1H).

^{13}C NMR (75 MHz, $CDCl_3$) δ 149.9, 140.4, 138.8, 129.6 (t, J = 33.8 Hz), 129.0, 128.2, 127.5, 127.2, 122.2, 121.0 (t, J = 6.8 Hz), 120.9 (t, J = 257.3 Hz).

^{19}F NMR (282 MHz, $CDCl_3$) δ -68.6 (d, J = 5.6 Hz, 2F).

HRMS (EI): calculated for $C_{15}H_{12}F_2O$ [M]$^+$: 246.0851, found: 246.0844.

1-(*Tert*-butyl)-4-((1,1-difluoroallyl)oxy)benzene (4)

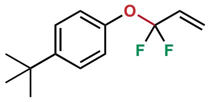

Chemical Formula: $C_{13}H_{16}F_2O$
Exact Mass: 226.1169

This compound was obtained following the general procedure C. Starting from ammonium salt **1a** (117 mg, 0.36 mmol, 1.2 equiv), 4-(tert-butyl)phenol **2b** (45 mg, 0.3 mmol), NaH (60% dispersion in mineral oil, 18 mg, 0.45 mmol, 1.5 equiv), and DMF (1.5 mL). Purification on silica gel (Pentane/Ethyl acetate gradient from 100/0 to 98/2) afforded **4** (67 mg, 99%) as a colorless oil.

^{1}H NMR (300 MHz, $CDCl_3$) δ 7.39 – 7.32 (m, 2H), 7.16 – 7.09 (m, 2H), 6.15 – 5.98 (m, 1H), 5.97 – 5.87 (m, 1H), 5.58 (dd, *J* = 10.5, 1.0 Hz, 1H), 1.32 (s, 9H).

^{13}C NMR (75 MHz, $CDCl_3$) δ 148.5, 148.0, 129.7 (t, *J* = 33.9 Hz), 126.3, 121.7 (t, *J* = 6.5 Hz), 121.5, 120.8 (t, *J* = 257.3 Hz), 34.6, 31.6.

^{19}F NMR (282 MHz, $CDCl_3$) δ -68.6 (d, *J* = 6.4 Hz, 2F).

HRMS (EI): calculated for $C_{13}H_{16}F_2O$ $[M]^+$: 226.1164, found: 226.1166.

1-((1,1-Difluoroallyl)oxy)-4-ethylbenzene (5)

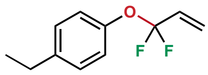

Chemical Formula: $C_{11}H_{12}F_2O$
Exact Mass: 198.0856

This compound was obtained following the general procedure C. Starting from ammonium salt **1a** (117 mg, 0.36 mmol, 1.2 equiv), 4-ethylphenol **2c** (36.6 mg, 0.3 mmol), NaH (60% dispersion in mineral oil, 18 mg, 0.45 mmol, 1.5 equiv), and DMF (1.5 mL). Purification on silica gel (Pentane/Ethyl acetate gradient from 100/0 to 99/1) afforded **5** (58 mg, 98%) as a colorless oil.

^{1}H NMR (300 MHz, $CDCl_3$) δ 7.21 – 7.06 (m, 4H), 6.14 – 5.97 (m, 1H), 5.97 – 5.86 (m, 1H), 5.58 (dd, *J* = 10.4, 1.0 Hz, 1H), 2.64 (q, *J* = 7.6 Hz, 2H), 1.24 (t, *J* = 7.6 Hz, 3H).

^{13}C NMR (75 MHz, $CDCl_3$) δ 148.3, 141.7, 129.7 (t, *J* = 34.0 Hz), 128.8, 122.0, 121.8 (t, *J* = 6.4 Hz), 120.8, 28.4, 15.7.

^{19}F NMR (282 MHz, $CDCl_3$) δ -68.7 (d, *J* = 6.3 Hz, 2F).

HRMS (EI): calculated for $C_{11}H_{12}F_2O$ $[M]^+$: 198.0851, found: 198.0860.

1-Bromo-4-((1,1-difluoroallyl)oxy)benzene (6)

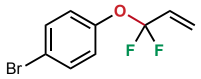

Chemical Formula: $C_9H_7BrF_2O$
Exact Mass: 247.9648

This compound was obtained following the general procedure C. Starting from ammonium salt **1a** (117 mg, 0.36 mmol, 1.2 equiv), 4-bromophenol **2d** (51.6 mg, 0.3 mmol), NaH (60% dispersion in mineral oil, 18 mg, 0.45 mmol, 1.5 equiv), and DMF (1.5 mL). Purification on silica gel (Pentane/Ethyl acetate gradient from 100/0 to 98/2) afforded **6** (72 mg, 97%) as a colorless oil.

^{1}H NMR (300 MHz, $CDCl_3$) δ 7.50 – 7.43 (m, 2H), 7.12 – 7.05 (m, 2H), 6.12 – 5.96 (m, 1H), 5.96 – 5.87 (m, 1H), 5.61 (dd, *J* = 10.3, 1.1 Hz, 1H).

^{13}C NMR (75 MHz, $CDCl_3$) δ 149.5, 132.6, 129.2 (t, *J* = 33.3 Hz), 123.8, 122.2 (t, *J* = 6.5 Hz), 120.7 (t, *J* = 257.8 Hz), 118.8.

^{19}F NMR (282 MHz, $CDCl_3$) δ -69.1 (d, *J* = 6.1 Hz, 2F).

HRMS (EI): calculated for $C_9H_6BrF_2O$ $[M-H]^+$: 246.9565, found: 246.9567.

1-((1,1-Difluoroallyl)oxy)-4-nitrobenzene (7)

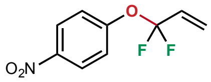

Chemical Formula: $C_9H_7F_2NO_3$
Exact Mass: 215.0394

This compound was obtained following the general procedure C. Starting from ammonium salt **1a** (117 mg, 0.36 mmol, 1.2 equiv), 4-nitrophenol **2e** (41.7 mg, 0.3 mmol), NaH (60% dispersion in mineral oil, 18 mg, 0.45 mmol, 1.5 equiv), and DMF (1.5 mL). Purification on silica gel (Pentane/Ethyl acetate gradient from 98/2 to 95/5) afforded **7** (60 mg, 93%) as a colorless oil.

^{1}H NMR (300 MHz, $CDCl_3$) δ 8.31 – 8.19 (m, 2H), 7.42 – 7.29 (m, 2H), 6.16 – 6.01 (m, 1H), 6.01 – 5.92 (m, 1H), 5.67 (dd, *J* = 10.1, 1.1 Hz, 1H).

^{13}C NMR (75 MHz, $CDCl_3$) δ 155.4, 128.7 (t, *J* = 32.7 Hz), 125.5, 122.9 (t, *J* = 6.7 Hz), 121.5, 120.9 (t, *J* = 260.3 Hz).

^{19}F NMR (282 MHz, $CDCl_3$) δ -69.5 (d, *J* = 6.2 Hz, 2F).

HRMS (EI): calculated for $C_9H_7F_2NO_3$ $[M]^+$: 215.0389, found: 215.0392.

1-(4-((1,1-Difluoroallyl)oxy)phenyl)ethan-1-one (8)

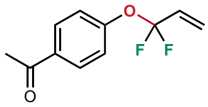

Chemical Formula: $C_{11}H_{10}F_2O_2$
Exact Mass: 212.0649

This compound was obtained following the general procedure C. Starting from ammonium salt

1a (117 mg, 0.36 mmol, 1.2 equiv), 1-(4-hydroxyphenyl)ethan-1-one **2f** (40.8 mg, 0.3 mmol), NaH (60% dispersion in mineral oil, 18 mg, 0.45 mmol, 1.5 equiv), and DMF (1.5 mL). Purification on silica gel (Pentane/Ethyl acetate gradient from 98/2 to 90/10) afforded **8** (54 mg, 85%) as a colorless oil.

^{1}H NMR (300 MHz, $CDCl_3$) δ 8.01 – 7.92 (m, 2H), 7.33 – 7.21 (m, 2H), 6.14 – 5.99 (m, 1H), 5.98 – 5.88 (m, 1H), 5.63 (dd, *J* = 10.3, 1.0 Hz, 1H), 2.59 (s, 3H).

^{13}C NMR (75 MHz, $CDCl_3$) δ 196.9, 154.3, 134.3, 130.1, 129.1 (t, *J* = 33.2 Hz), 122.4 (t, *J* = 6.6 Hz), 121.2, 120.9 (t, *J* = 259.5 Hz), 26.7.

^{19}F NMR (282 MHz, $CDCl_3$) δ -69.0 (d, *J* = 6.3 Hz, 2F).

HRMS (EI): calculated for $C_{11}H_{10}F_2O_2$ $[M]^+$: 212.0643, found: 212.0647.

(4-((1,1-Difluoroallyl)oxy)phenyl)(phenyl)methanone (9)

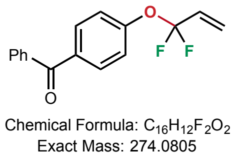

This compound was obtained following the general procedure C. Starting from ammonium salt **1a** (117 mg, 0.36 mmol, 1.2 equiv), (4-hydroxyphenyl)(phenyl)methanone **2g** (59.4 mg, 0.3 mmol), NaH (60% dispersion in mineral oil, 18 mg, 0.45 mmol, 1.5 equiv), and DMF (1.5 mL). Purification on silica gel (Pentane/Ethyl acetate gradient from 98/2 to 90/10) afforded **9** (77 mg, 98%) as a colorless oil.

^{1}H NMR (300 MHz, $CDCl_3$) δ 7.88 – 7.75 (m, 4H), 7.63 – 7.56 (m, 1H), 7.53 – 7.45 (m, 2H), 7.35 – 7.27 (m, 2H), 6.17 – 6.01 (m, 1H), 6.01 – 5.91 (m, 1H), 5.64 (dd, *J* = 10.3, 1.0 Hz, 1H).

^{13}C NMR (75 MHz, $CDCl_3$) δ 195.6, 153.8, 137.6, 134.6, 132.6, 132.6, 131.9, 130.1, 129.1 (t, *J* = 33.1 Hz), 128.5, 122.4 (t, *J* = 6.5 Hz), 121.1, 120.9 (t, *J* = 259.5 Hz).

^{19}F NMR (282 MHz, $CDCl_3$) δ -68.9 (d, *J* = 6.3 Hz, 2F).

HRMS (EI): calculated for $C_{16}H_{12}F_2O_2$ $[M]^+$: 274.0800, found: 274.0802.

3-((1,1-Difluoroallyl)oxy)benzaldehyde (10)

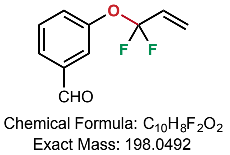

This compound was obtained following the general procedure C. Starting from ammonium salt **1a** (117 mg, 0.36 mmol, 1.2 equiv), 3-hydroxybenzaldehyde **2h** (36.6 mg, 0.3 mmol), NaH (60% dispersion in mineral oil, 18 mg, 0.45 mmol, 1.5 equiv), and DMF (1.5 mL). Purification on silica

gel (Pentane/Ethyl acetate gradient from 98/2 to 90/10) afforded **10** (45 mg, 76%) as a colorless oil.

^{1}H NMR (300 MHz, $CDCl_3$) δ 10.00 (d, *J* = 0.4 Hz, 1H), 7.77 – 7.69 (m, 2H), 7.57 – 7.43 (m, 2H), 6.24 – 6.00 (m, 1H), 6.00 – 5.86 (m, 1H), 5.63 (dd, *J* = 10.3, 1.0 Hz, 1H).

^{13}C NMR (75 MHz, $CDCl_3$) δ 191.4, 151.1, 137.9, 130.3, 129.1 (t, *J* = 33.4 Hz), 127.9, 127.1, 122.4, 122.3 (t, *J* = 6.6 Hz), 120.9 (t, *J* = 259.5 Hz).

^{19}F NMR (282 MHz, $CDCl_3$) δ -69.0 (d, *J* = 6.3 Hz, 2F).

HRMS (EI): calculated for $C_{10}H_8F_2O_2$ $[M]^+$: 198.0487, found: 198.0493.

Methyl 4-((1,1-difluoroallyl)oxy)-3-iodobenzoate (11)

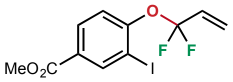

Chemical Formula: $C_{11}H_9F_2IO_3$
Exact Mass: 353.9564

This compound was obtained following the general procedure C. Starting from ammonium salt **1a** (117 mg, 0.36 mmol, 1.2 equiv), methyl 4-hydroxy-3-iodobenzoate **2i** (83.4 mg, 0.3 mmol), NaH (60% dispersion in mineral oil, 18 mg, 0.45 mmol, 1.5 equiv), and DMF (1.5 mL). Purification on silica gel (Pentane/Ethyl acetate gradient from 98/2 to 90/10) afforded **11** (91 mg, 86%) as a colorless oil.

^{1}H NMR (300 MHz, $CDCl_3$) δ 8.50 (d, *J* = 2.1 Hz, 1H), 8.00 (dd, *J* = 8.6, 2.1 Hz, 1H), 7.39 (dt, *J* = 8.6, 1.6 Hz, 1H), 6.13 – 6.05 (m, 2H), 5.71 – 5.62 (m, 1H), 3.91 (s, 3H).

^{13}C NMR (75 MHz, $CDCl_3$) δ 165.1, 154.1, 141.3, 131.0, 129.2 – 128.2 (m, 2C), 123.2 (t, *J* = 6.8 Hz), 121.2 (t, *J* = 261.8 Hz), 120.8 (t, *J* = 2.5 Hz), 89.7, 52.6.

^{19}F NMR (282 MHz, $CDCl_3$) δ -67.9 (d, *J* = 2.3 Hz, 2F).

HRMS (EI): calculated for $C_{11}H_9F_2IO_3$ $[M]^+$: 353.9559, found: 353.9557.

2-((1,1-Difluoroallyl)oxy)-1-fluoro-3-iodobenzene (12)

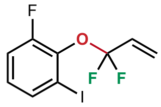

Chemical Formula: $C_9H_6F_3IO$
Exact Mass: 313.9415

This compound was obtained following the general procedure C. Starting from ammonium salt **1a** (117 mg, 0.36 mmol, 1.2 equiv), 2-fluoro-6-iodophenol **2j** (71.4 mg, 0.3 mmol), NaH (60% dispersion in mineral oil, 18 mg, 0.45 mmol, 1.5 equiv), and DMF (1.5 mL). Purification on silica gel (Pentane/Ethyl acetate gradient from 99/1 to 95/5) afforded **12** (86 mg, 92%) as a colorless oil.

^{1}H NMR (300 MHz, $CDCl_3$) δ 7.61 (dt, *J* = 8.0, 1.5 Hz, 1H), 7.19 – 7.09 (m, 1H), 7.01 – 6.91 (m, 1H), 6.23 – 6.07 (m, 1H), 6.07 – 5.98 (m, 1H), 5.68 – 5.61 (m, 1H).

^{13}C NMR (75 MHz, $CDCl_3$) δ 135.0 (d, *J* = 3.7 Hz), 128.8 (t, *J* = 32.7 Hz), 128.7 (d, *J* = 7.8 Hz), 122.8 (t, *J* = 6.6 Hz), 117.43 (d, *J* = 20.3 Hz), 94.3.

^{19}F NMR (282 MHz, $CDCl_3$) δ -67.1 (dd, *J* = 19.7, 6.2 Hz, 2F), -120.3 – -120.6 (m, 1F).

HRMS (EI): calculated for $C_9H_6F_3IO$ $[M]^+$: 313.9410, found: 313.9415.

3-((1,1-Difluoroallyl)oxy)-2-iodopyridine (13)

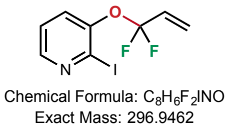

This compound was obtained following the general procedure C. Starting from ammonium salt **1a** (117 mg, 0.36 mmol, 1.2 equiv), 2-iodopyridin-3-ol **2k** (40.2 mg, 0.15 mmol), NaH (60% dispersion in mineral oil, 18 mg, 0.45 mmol, 1.5 equiv), and DMF (1.5 mL). Purification on silica gel (Pentane/Ethyl acetate gradient from 99/1 to 90/10) afforded **13** (73 mg, 88%) as a colorless oil.

^{1}H NMR (300 MHz, $CDCl_3$) δ 8.24 (dd, *J* = 4.6, 1.6 Hz, 1H), 7.59 – 7.53 (m, 1H), 7.25 (dd, *J* = 8.1, 4.6 Hz, 1H), 6.20 – 6.01 (m, 2H), 5.77 – 5.63 (m, 1H).

^{13}C NMR (75 MHz, $CDCl_3$) δ 148.1, 147.4, 128.5 (t, *J* = 32.6 Hz), 128.4 (t, *J* = 2.1 Hz), 123.5, 123.4 (t, *J* = 6.9 Hz), 121.3 (t, *J* = 262.5 Hz), 115.3.

^{19}F NMR (282 MHz, $CDCl_3$) δ -67.8 (dd, *J* = 3.3, 1.1 Hz, 2F).

HRMS (EI): calculated for $C_8H_6F_2INO$ $[M]^+$: 296.9457, found: 296.9466.

1-Butoxy-4-((1,1-difluoroallyl)oxy)benzene (14)

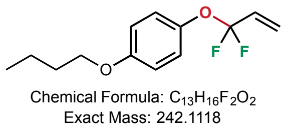

This compound was obtained following the general procedure C. Starting from ammonium salt **1a** (780 mg, 2.4 mmol, 1.2 equiv), 4-butoxyphenol **2l** (332.4 mg, 2 mmol), NaH (60% dispersion in mineral oil, 120 mg, 3.0 mmol, 1.5 equiv), and DMF (10 mL). Purification on silica gel (Pentane/Ethyl acetate gradient from 100/0 to 99/1) afforded **14** (480 mg, 99%) as a colorless oil.

^{1}H NMR (300 MHz, $CDCl_3$) δ 7.16 – 7.07 (m, 2H), 6.90 – 6.80 (m, 2H), 6.13 – 5.96 (m, 1H), 5.95 – 5.85 (m, 1H), 5.57 (dd, *J* = 10.5, 1.0 Hz, 1H), 3.94 (t, *J* = 6.5 Hz, 2H), 1.86 – 1.66 (m, 2H), 1.59 – 1.38 (m, 2H), 0.98 (t, *J* = 7.4 Hz, 3H).

^{13}**C NMR** (75 MHz, $CDCl_3$) δ 157.0, 143.6, 129.7 (t, *J* = 34.0 Hz), 123.4, 121.7 (t, *J* = 6.4 Hz), 120.8 (t, *J* = 256.5 Hz), 115.0, 68.2, 31.5, 19.4, 14.0.

^{19}**F NMR** (282 MHz, Chloroform-*d*) δ -68.9 (d, *J* = 6.7 Hz, 2F).

HRMS (EI): calculated for $C_{13}H_{16}F_2O_2$ $[M]^+$: 242.1112, found: 242.1113.

2-(4-((1,1-Difluoroallyl)oxy)phenyl)-4,4,5,5-tetramethyl-1,3,2-dioxaborolane (15)

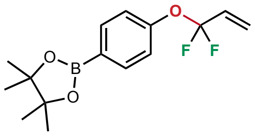

Chemical Formula: $C_{15}H_{19}BF_2O_3$
Exact Mass: 296.1395

This compound was obtained following the general procedure C. Starting from ammonium salt **1a** (117 mg, 0.36 mmol, 1.2 equiv), 4-(4,4,5,5-tetramethyl-1,3,2-dioxaborolan-2-yl)phenol **2m** (66 mg, 0.3 mmol), NaH (60% dispersion in mineral oil, 18 mg, 0.45 mmol, 1.5 equiv), and DMF (1.5 mL). Purification on silica gel (Pentane/Ethyl acetate gradient from 95/5 to 90/10) afforded **15** (60 mg, 68%) as a colorless oil.

^{1}**H NMR** (300 MHz, $CDCl_3$) δ 7.84 – 7.75 (m, 2H), 7.25 – 7.14 (m, 2H), 6.14 – 5.97 (m, 1H), 5.97 – 5.85 (m, 1H), 5.59 (dd, *J* = 10.4, 1.1 Hz, 1H), 1.34 (s, 12H).

^{13}**C NMR** (75 MHz, $CDCl_3$) δ 153.1, 136.3, 129.5 (t, *J* = 33.7 Hz), 122.0 (t, *J* = 6.5 Hz), 120.9, 120.8 (t, *J* = 258.0 Hz), 84.0, 25.0.

^{19}**F NMR** (282 MHz, $CDCl_3$) δ -68.5 (d, *J* = 6.2 Hz, 2F).

HRMS (EI): calculated for $C_{15}H_{19}BF_2O_3$ $[M]^+$: 296.1390, found: 296.1393.

1-((1,1-Difluoroallyl)oxy)naphthalene (16)

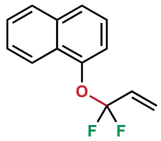

Chemical Formula: $C_{13}H_{10}F_2O$
Exact Mass: 220.0700

This compound was obtained following the general procedure C. Starting from ammonium salt **1a** (117 mg, 0.36 mmol, 1.2 equiv), naphthalen-1-ol **2n** (72.1 mg, 0.3 mmol), NaH (60% dispersion in mineral oil, 18 mg, 0.45 mmol, 1.5 equiv), and DMF (1.5 mL). Purification on silica gel (Pentane/Ethyl acetate gradient from 98/2 to 95/5) afforded **16** (109 mg, 99%) as a colorless oil.

^{1}**H NMR** (300 MHz, $CDCl_3$) δ 8.21 – 8.09 (m, 1H), 7.91 – 7.80 (m, 1H), 7.79 – 7.67 (m, 1H), 7.60 – 7.47 (m, 2H), 7.47 – 7.38 (m, 2H), 6.30 – 6.11 (m, 1H), 6.05 (dtd, *J* = 17.3, 1.8, 1.0 Hz, 1H), 5.66 (dd, *J* = 10.5, 0.9 Hz, 1H).

^{13}**C NMR** (75 MHz, $CDCl_3$) δ 146.3 (t, *J* = 1.6 Hz), 134.8, 129.5 (t, *J* = 33.8 Hz), 128.0, 127.9, 126.6, 126.5, 125.7, 125.5, 122.2, 122.1 (t, *J* = 6.5 Hz), 121.3 (t, *J* = 258.0 Hz), 117.9, 117.6 (t, *J* = 1.4 Hz).

^{19}**F NMR** (282 MHz, $CDCl_3$) δ -68.2 (d, *J* = 6.2 Hz, 2F).

HRMS (EI): calculated for $C_{13}H_{10}F_2O$ $[M]^+$: 220.0694, found: 220.0697.

1-(4-((1,1-Difluoroallyl)oxy)phenyl)-1*H*-imidazole (17)

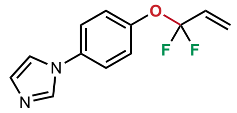

Chemical Formula: $C_{12}H_{10}F_2N_2O$
Exact Mass: 236.0761

This compound was obtained following the general procedure C. Starting from ammonium salt **1a** (117 mg, 0.36 mmol, 1.2 equiv), 4-(1H-imidazol-1-yl)phenol **2o** (48 mg, 0.3 mmol), NaH (60% dispersion in mineral oil, 18 mg, 0.45 mmol, 1.5 equiv), and DMF (1.5 mL). Purification on silica gel (Pentane/Ethyl acetate gradient from 98/2 to 95/5) afforded **17** (70 mg, 99%) as a colorless oil.

^{1}**H NMR** (300 MHz, $CDCl_3$) δ 7.81 (s, 1H), 7.41 – 7.27 (m, 4H), 7.26 – 7.16 (m, 2H), 6.16 – 5.99 (m, 1H), 5.99 – 5.89 (m, 1H), 5.63 (dd, *J* = 10.3, 1.0 Hz, 1H).

^{13}**C NMR** (75 MHz, $CDCl_3$) δ 149.4, 135.8, 134.9, 130.6, 129.1 (t, *J* = 33.3 Hz), 123.4 , 122.7, 122.3 (t, *J* = 6.5 Hz), 120.8 (t, *J* = 258.8 Hz), 118.5.

^{19}**F NMR** (282 MHz, $CDCl_3$) δ -69.0 (d, *J* = 6.2 Hz, 2F).

HRMS (EI): calculated for $C_{12}H_{10}F_2N_2O$ $[M]^+$: 236.0756, found: 236.0760.

Ethyl 2-(4-((1,1-difluoroallyl)oxy)-3-formylphenyl)-4-methylthiazole-5-carboxylate (18)

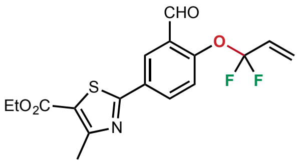

Chemical Formula: $C_{17}H_{15}F_2NO_4S$
Exact Mass: 367.0690

This compound was obtained following the general procedure C. Starting from ammonium salt **1a** (117 mg, 0.36 mmol, 1.2 equiv), ethyl 2-(3-formyl-4-hydroxyphenyl)-4-methylthiazole-5-carboxylate **2p** (87.4 mg, 0.3 mmol), NaH (60% dispersion in mineral oil, 18 mg, 0.45 mmol, 1.5 equiv), and DMF (1.5 mL). Purification on silica gel (Pentane/Ethyl acetate gradient from 95/5 to 80/20) afforded **18** (86 mg, 78%) as a white solid. M.p. 99–100 °C.

^{1}**H NMR** (300 MHz, $CDCl_3$) δ 10.36 (s, 1H), 8.47 – 8.39 (m, 1H), 8.24 (dd, *J* = 8.7, 2.4 Hz, 1H), 7.50 (dt, *J* = 8.8, 1.8 Hz, 1H), 6.25 – 6.06 (m, 1H), 6.06 – 5.95 (m, 1H), 5.71 (dd, *J* = 10.3, 0.9 Hz,

1H), 4.36 (q, J = 7.1 Hz, 2H), 2.78 (s, 3H), 1.39 (t, J = 7.1 Hz, 3H).

^{13}C NMR (75 MHz, $CDCl_3$) δ 187.9, 167.3, 162.2, 161.4, 153.8, 133.1, 131.2, 129.2, 128.4 (t, J = 32.6 Hz), 127.1, 123.5, 123.1 (t, J = 6.7 Hz), 122.9, 121.0, 61.6, 17.6, 14.5.

^{19}F NMR (282 MHz, $CDCl_3$) δ -68.8 (d, J = 6.5 Hz, 2F).

HRMS (ESI): calculated for $C_{17}H_{16}F_2NO_4S$ $[M]^+$: 368.0768, found: 368.0770.

7-((1,1-Difluoroallyl)oxy)-3,4-dihydroquinolin-2(1*H*)-one (19)

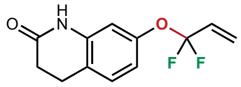

Chemical Formula: $C_{12}H_{11}F_2NO_2$
Exact Mass: 239.0758

This compound was obtained following the general procedure D. Starting from ammonium salt **1a** (1.78 g, 5.5 mmol, 1.1 equiv), 7-hydroxy-3,4-dihydroquinolin-2(1H)-one **2q** (0.815 g, 5.0 mmol), Cs_2CO_3 (0.815 g, 2.5 mmol, 0.5 equiv), and Toluene (30 mL). Purification on silica gel (Pentane/Ethyl acetate gradient from 90/10 to 2/1) afforded **19** (1.08 g, 90%) as a white solid.

^{1}H NMR (300 MHz, $CDCl_3$) δ 9.09 (s, 1H), 7.11 (d, J = 8.1 Hz, 1H), 6.89 – 6.79 (m, 1H), 6.74 – 6.65 (m, 1H), 6.14 – 5.94 (m, 1H), 5.97 – 5.84 (m, 1H), 5.59 (dd, J = 10.4, 1.1 Hz, 1H), 2.95 (dd, J = 8.6, 6.5 Hz, 2H), 2.70 – 2.57 (m, 2H).

^{13}C NMR (75 MHz, $CDCl_3$) δ 172.2, 149.7, 138.3, 129.4 (t, J = 33.6 Hz), 128.7, 122.0 (t, J = 6.4 Hz), 121.0, 120.8 (t, J = 258.0 Hz), 116.4, 109.6, 30.8, 24.9.

^{19}F NMR (282 MHz, $CDCl_3$) δ -68.7 (d, J = 6.1 Hz, 2F).

HRMS (EI): calculated for $C_{12}H_{11}F_2NO_2$ $[M]^+$: 239.0752, found: 239.0755.

2-((1,1-Difluoroallyl)oxy)isoindoline-1,3-dione (20)

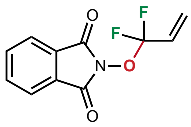

Chemical Formula: $C_{11}H_7F_2NO_3$
Exact Mass: 239.0394

This compound was obtained following the general procedure C. Starting from ammonium salt **1a** (117 mg, 0.36 mmol, 1.2 equiv), N-Hydroxyphthalimide **2r** (40.2 mg, 0.15 mmol), NaH (60% dispersion in mineral oil, 18 mg, 0.45 mmol, 1.5 equiv), and DMF (1.5 mL). 95% NMR yield of the desired product was determined by crude ^{19}F NMR. The desired product **20** (119 mg, 50%) was isolated via precipitation from water as a white solid. M.p. 143–145 °C.

^{1}H NMR (300 MHz, Acetone-d_6) δ 7.98 – 7.94 (m, 4H), 6.37 – 6.19 (m, 1H), 6.13 – 6.02 (m, 1H), 5.90 (dd, J = 10.8, 0.5 Hz, 1H).

^{13}C NMR (75 MHz, Acetone-d_6) δ 163.2, 136.1, 130.0, 127.0 (t, J = 29.9 Hz), 125.9 (t, J = 6.5 Hz),

124.6, 123.7.

^{19}F NMR (282 MHz, Acetone-d_6) δ -76.6 (d, J = 2.2 Hz, 2F).

HRMS (EI): calculated for $C_{11}H_7F_2NO_3$ $[M]^+$: 239.0389, found: 239.0387.

(*E*)-benzaldehyde *O*-(1,1-difluoroallyl) oxime (21)

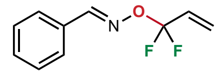

This compound was obtained following the general procedure C. Starting from ammonium salt **1a** (117 mg, 0.36 mmol, 1.2 equiv), (E)-benzaldehyde oxime **2s** (36.6 mg, 0.3 mmol), NaH (60% dispersion in mineral oil, 18 mg, 0.45 mmol, 1.5 equiv), and DMF (1.5 mL). Purification on silica gel (Pentane/Ethyl acetate gradient from 99/1 to 95/5, with 2% Et_3N) afforded **21** (58 mg, 99%) as a colorless oil.

^{1}H NMR (300 MHz, $CDCl_3$) δ 8.24 (s, 1H), 7.73 – 7.61 (m, 2H), 7.51 – 7.36 (m, 3H), 6.18 – 5.98 (m, 1H), 5.97 – 5.86 (m, 1H), 5.63 (dd, J = 10.6, 1.0 Hz, 1H).

^{13}C NMR (75 MHz, $CDCl_3$) δ 154.4, 131.4, 130.7, 129.0, 128.6 (t, J = 32.0 Hz), 128.1, 122.4 (t, J = 6.5 Hz), 121.9 (t, J = 257.3 Hz).

^{19}F NMR (282 MHz, $CDCl_3$) δ -74.9 (dd, J = 7.0, 1.5 Hz, 2F).

HRMS (ESI): calculated for $C_{11}H_9F_2NO$ $[M+H]^+$: 198.0730, found: 198.0730.

2-((1,1-Difluoroallyl)oxy)-2-azaadamantane (22)

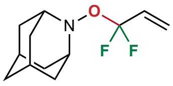

This compound was obtained following the general procedure C. Starting from ammonium salt **1a** (117 mg, 0.36 mmol, 1.2 equiv), *N*-Hydroxyphthalimide **2t** (46 mg, 0.3 mmol), NaH (60% dispersion in mineral oil, 18 mg, 0.45 mmol, 1.5 equiv), and DMF (1.5 mL). Purification on silica gel (Pentane/Ethyl acetate gradient from 100/0 to 99/1, with 2% Et_3N) afforded **22** (66 mg, 99%) as a colorless oil.

^{1}H NMR (300 MHz, $CDCl_3$) δ 6.04 – 5.86 (m, 1H), 5.83 – 5.72 (m, 1H), 5.48 (dd, J = 10.7, 1.1 Hz, 1H), 3.40 (s, 2H), 2.35 (d, J = 10.6 Hz, 2H), 2.10 (d, J = 13.6 Hz, 2H), 1.99 – 1.86 (m, 2H), 1.86 – 1.76 (m, 4H), 1.48 – 1.38 (m, 2H).

^{13}C NMR (75 MHz, $CDCl_3$) δ 129.7 (t, J = 33.5 Hz), 122.5 (t, J = 252.8 Hz), 121.0 (t, J = 6.3 Hz), 56.9, 36.7, 36.6, 30.3, 26.4, 25.8.

^{19}F NMR (282 MHz, $CDCl_3$) δ -76.1 (dd, J = 7.0, 1.8 Hz, 2F).

HRMS (EI): calculated for $C_{12}H_{17}F_2NO$ $[M]^+$: 229.1273, found: 229.1267.

1-(2-((1,1-Difluoroallyl)oxy)ethyl)naphthalene (23)

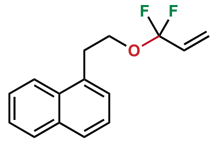

Chemical Formula: $C_{15}H_{14}F_2O$
Exact Mass: 248.1013

This compound was obtained following the general procedure C. Starting from ammonium salt **1a** (117 mg, 0.36 mmol, 1.2 equiv), 2-(naphthalen-1-yl)ethan-1-ol **2u** (46 mg, 0.3 mmol), NaH (60% dispersion in mineral oil, 18 mg, 0.45 mmol, 1.5 equiv), and DMF (1.5 mL). Purification on silica gel (Pentane/Ethyl acetate gradient from 99/1 to 95/5, with 2% Et_3N) afforded **23** (74.4 mg, 95%) as a colorless oil.

^{1}H NMR (300 MHz, Benzene-*d*$_6$) δ 7.78 – 7.69 (m, 1H), 7.60 – 7.51 (m, 1H), 7.50 – 7.42 (m, 1H), 7.20 – 7.16 (m, 1H), 7.11 – 7.04 (m, 2H), 7.02 – 6.96 (m, 1H), 5.75 – 5.58 (m, 1H), 5.58 – 5.50 (m, 1H), 4.91 – 4.84 (m, 1H), 4.03 (t, *J* = 7.5 Hz, 2H), 3.10 (t, *J* = 7.4 Hz, 2H).

^{13}C NMR (75 MHz, Benzene-*d*$_6$) δ 134.4, 133.9, 132.6, 130.2 (t, *J* = 34.9 Hz), 129.2, 127.6, 127.2, 126.4, 125.8, 125.7, 123.8, 122.0, 120.74 (t, *J* = 6.5 Hz), 63.7 (t, *J* = 5.9 Hz), 33.1.

^{19}F NMR (282 MHz, Benzene-*d*$_6$) δ -71.9 (dd, *J* = 5.6, 2.0 Hz, 2F).

HRMS (EI): calculated for $C_{15}H_{14}F_2O$ $[M]^+$: 248.1007, found: 248.1017.

5-(((1,1-Difluoroallyl)oxy)methyl)furan-2-carbaldehyde (24)

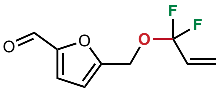

Chemical Formula: $C_9H_8F_2O_3$
Exact Mass: 202.0442

This compound was obtained following the general procedure C. Starting from ammonium salt **1a** (117 mg, 0.36 mmol, 1.2 equiv), 5-(hydroxymethyl)furan-2-carbaldehyde **2v** (37.8 mg, 0.3 mmol), NaH (60% dispersion in mineral oil, 18 mg, 0.45 mmol, 1.5 equiv), and DMF (1.5 mL). Purification on silica gel (Pentane/Ethyl acetate gradient from 99/1 to 95/5, with 2% Et_3N) afforded **24** (38 mg, 63%) as a colorless oil.

^{1}H NMR (300 MHz, $CDCl_3$) δ 9.64 (s, 1H), 7.21 (dd, *J* = 3.6, 0.4 Hz, 1H), 6.59 (dt, *J* = 3.6, 0.6 Hz, 1H), 6.00 – 5.86 (m, 1H), 5.85 – 5.77 (m, 1H), 5.55 – 5.49 (m, 1H), 4.96 (s, 2H).

^{13}C NMR (75 MHz, $CDCl_3$) δ 178.0, 155.6, 153.1, 129.0 (t, *J* = 33.5 Hz), 121.9 (t, *J* = 6.5 Hz), 121.7, 121.4 (t, *J* = 256.5 Hz), 112.3, 57.6 (t, *J* = 7.3 Hz).

^{19}F NMR (282 MHz, $CDCl_3$) δ -72.98 – -73.11 (m, 2F).

HRMS (EI): calculated for $C_9H_8F_2O_3$ $[M]^+$: 202.0436, found: 202.0433.

1,4-Bis((1,1-difluoroallyl)oxy)but-2-yne (25)

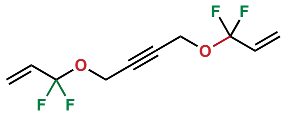

This compound was obtained following the general procedure C. Starting from ammonium salt **1a** (390 mg, 1.2 mmol, 2.4 equiv), but-2-yne-1,4-diol **2w** (43 mg, 0.5 mmol), NaH (60% dispersion in mineral oil, 60 mg, 1.5 mmol, 3.0 equiv), and DMF (1.5 mL). Purification on silica gel (Pentane/Ethyl acetate gradient from 99/1 to 95/5, with 2% Et_3N) afforded **25** (107 mg, 90%) as a colorless oil.

^{1}H NMR (300 MHz, $CDCl_3$) δ 6.00 – 5.86 (m, 2H), 5.86 – 5.77 (m, 2H), 5.56 – 5.47 (m, 2H), 4.60 (s, 4H).

^{13}C NMR (75 MHz, $CDCl_3$) δ 129.1 (t, *J* = 33.5 Hz), 121.8 (t, *J* = 6.6 Hz), 121.4 (t, *J* = 256.5 Hz), 81.1, 51.8 (t, *J* = 8.1 Hz).

^{19}F NMR (282 MHz, $CDCl_3$) δ -73.8 (dd, *J* = 5.6, 1.2 Hz, 4F).

***N*-allyl-*N*-(4-((1,1-difluoroallyl)oxy)but-2-yn-1-yl)-4-methylbenzenesulfonamide (26)**

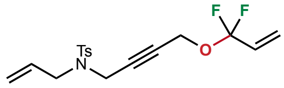

Chemical Formula: $C_{17}H_{19}F_2NO_3S$
Exact Mass: 355.1054

This compound was obtained following the general procedure C. Starting from ammonium salt **1a** (336 mg, 1.03 mmol, 1.2 equiv), N-allyl-N-(4-hydroxybut-2-yn-1-yl)-4-methylbenzene-sulfonamide **2x** (240 mg, 0.86 mmol), NaH (60% dispersion in mineral oil, 51.6 mg, 1.29 mmol, 1.5 equiv), and DMF (4.5 mL). Purification on silica gel (Pentane/Ethyl acetate gradient from 95/5 to 90/10, with 2% Et_3N) afforded **26** (248 mg, 81%) as a colorless oil.

^{1}H NMR (300 MHz, $CDCl_3$) δ 7.77 – 7.68 (m, 2H), 7.32 – 7.26 (m, 2H), 5.94 – 5.65 (m, 3H), 5.54 – 5.47 (m, 1H), 5.32 – 5.19 (m, 2H), 4.24 (t, *J* = 1.9 Hz, 2H), 4.12 (t, *J* = 1.9 Hz, 2H), 3.80 (dt, *J* = 6.4, 1.3 Hz, 2H), 2.41 (s, 3H).

^{13}C NMR (75 MHz, $CDCl_3$) δ 143.7, 136.0, 132.0, 129.6, 129.1 (t, *J* = 33.8 Hz), 127.9, 121.7 (t, *J* = 6.5 Hz), 121.3 (t, *J* = 257.3 Hz), 120.1, 79.8, 79.8, 51.5 (t, *J* = 8.1 Hz), 49.3, 36.2, 21.6.

^{19}F NMR (282 MHz, $CDCl_3$) δ -73.7 (dd, *J* = 5.6, 1.5 Hz, 2F).

HRMS (EI): calculated for $C_{17}H_{19}F_2NO_3SNa$ $[M+Na]^+$: 378.0946, found: 387.0940.

(3-((1,1-Difluoroallyl)oxy)prop-1-yn-1-yl)benzene (27)

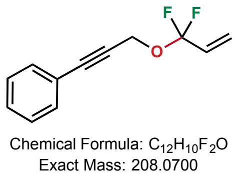

This compound was obtained following the general procedure C. Starting from ammonium salt **1a** (780 mg, 2.4 mmol, 1.2 equiv), 3-phenylprop-2-yn-1-ol **2y** (264 mg, 2.0 mmol), NaH (60% dispersion in mineral oil, 120 mg, 3.0 mmol, 1.5 equiv), and DMF (10 mL). Purification on silica gel (Pentane/Ethyl acetate gradient from 99/1 to 95/5, with 2% Et_3N) afforded **27** (410 mg, 99%) as a colorless oil.

^{1}H NMR (300 MHz, $CDCl_3$) δ 7.52 – 7.41 (m, 2H), 7.39 – 7.28 (m, 3H), 6.07 – 5.78 (m, 2H), 5.54 (dd, *J* = 10.1, 1.4 Hz, 1H), 4.79 (s, 2H).

^{13}C NMR (75 MHz, $CDCl_3$) δ 132.0, 129.3 (t, *J* = 33.7 Hz), 128.9, 128.5, 122.3, 121.7 (t, *J* = 6.6 Hz), 121.5 (t, *J* = 256.5 Hz), 86.8, 83.1, 52.6 (t, *J* = 8.1 Hz).

^{19}F NMR (282 MHz, $CDCl_3$) δ -73.5 (dd, *J* = 5.9, 1.6 Hz, 2F).

HRMS (EI): calculated for $C_{12}H_8F_2O$ $[M-H]^+$: 207.0616, found: 207.0621.

4,4'-Bis((1,1-difluoroallyl)thio)-1,1'-biphenyl (28)

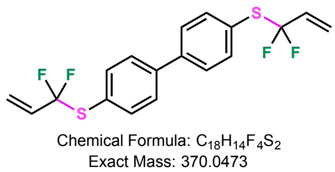

This compound was obtained following the general procedure C. Starting from ammonium salt **1a** (117 mg, 0.36 mmol, 2.4 equiv), [1,1'-biphenyl]-4,4'-dithiol **2z** (32.7mg, 0.15 mmol), NaH (60% dispersion in mineral oil, 18 mg, 0.45 mmol, 3.0 equiv), and DMF (1.5 mL). Purification on silica gel (Pentane/Ethyl acetate gradient from 99/1 to 95/5) afforded **28** (49 mg, 88%) as a colorless oil.

^{1}H NMR (300 MHz, $CDCl_3$) δ 7.73 – 7.64 (m, 4H), 7.64 – 7.57 (m, 4H), 6.19 – 5.98 (m, 2H), 5.68 (dt, *J* = 17.2, 2.3 Hz, 2H), 5.48 (dd, *J* = 10.8, 0.5 Hz, 2H).

^{13}C NMR (75 MHz, $CDCl_3$) δ 141.7, 136.8, 132.0 (t, *J* = 26.5 Hz), 127.9, 126.8 (t, *J* = 1.5 Hz) 125.8 (t, *J* = 273.8 Hz), 121.2 (t, *J* = 7.5 Hz).

^{19}F NMR (282 MHz, $CDCl_3$) δ -73.5 (dd, *J* = 8.5, 2.8 Hz, 4F).

HRMS (EI): calculated for $C_{18}H_{14}F_4S_2$ $[M]^+$: 370.0468, found: 370.0470.

(4-Chlorophenyl)(1,1-difluoroallyl)sulfane (29)

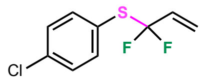

Chemical Formula: $C_9H_7ClF_2S$
Exact Mass: 219.9925

This compound was obtained following the general procedure C. Starting from ammonium salt **1a** (117 mg, 0.36 mmol, 1.2 equiv), 4-chlorobenzenethiol **2aa** (45.4mg, 0.3 mmol), NaH (60% dispersion in mineral oil, 18 mg, 0.45 mmol, 1.5 equiv), and DMF (1.5 mL). Purification on silica gel (Pentane/Ethyl acetate gradient from 99/1 to 98/2) afforded **29** (60 mg, 91%) as a colorless oil.

^{1}H NMR (300 MHz, $CDCl_3$) δ 7.58 – 7.49 (m, 2H), 7.39 – 7.32 (m, 2H), 6.13 – 5.94 (m, 1H), 5.65 (dtd, *J* = 17.2, 2.3, 0.4 Hz, 1H), 5.47 (dd, *J* = 10.8, 0.6 Hz, 1H).

^{13}C NMR (75 MHz, $CDCl_3$) δ 137.7, 136.7, 131.8 (t, *J* = 26.5 Hz), 129.4, 125.6 (t, *J* = 1.5 Hz), 125.5, 121.4 (t, *J* = 7.6 Hz).

^{19}F NMR (282 MHz, $CDCl_3$) δ -73.8 (dd, *J* = 9.6, 2.6 Hz, 2F).

(1,1-difluoroallyl)(naphthalen-1-yl)sulfane (30)

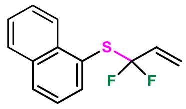

Chemical Formula: $C_{13}H_{10}F_2S$
Exact Mass: 236.0471

This compound was obtained following the general procedure C. Starting from ammonium salt **1a** (117 mg, 0.36 mmol, 1.2 equiv), naphthalene-1-thiol **2ac** (48 mg, 0.3 mmol), NaH (60% dispersion in mineral oil, 18 mg, 0.45 mmol, 1.5 equiv), and DMF (1.5 mL). Purification on silica gel (Pentane/Ethyl acetate gradient from 99/1 to 98/2) afforded **29** (70 mg, 99%) as a colorless oil.

^{1}H NMR (300 MHz, $CDCl_3$) δ 8.64 – 8.52 (m, 1H), 8.00 – 7.82 (m, 3H), 7.68 – 7.45 (m, 3H), 6.18 – 5.95 (m, 1H), 5.63 (dtd, *J* = 17.2, 2.3, 0.4 Hz, 1H), 5.39 (dd, *J* = 10.8, 0.5 Hz, 1H).

^{13}C NMR (75 MHz, $CDCl_3$) δ 137.6, 135.9, 134.3, 132.1 (t, *J* = 26.6 Hz), 131.5, 128.5, 127.3, 126.6, 126.5, 126.1, 125.6, 124.5, 120.8 (t, *J* = 7.5 Hz).

^{19}F NMR (282 MHz, $CDCl_3$) δ -73.2 (dd, *J* = 10.0, 2.3 Hz, 2F).

HRMS (EI): calculated for $C_{13}H_{10}F_2S$ $[M]^+$: 236.0466, found: 236.0465.

Benzyl(1,1-difluoroallyl)sulfane (31)

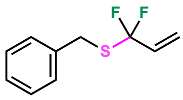

Chemical Formula: $C_{10}H_{10}F_2S$
Exact Mass: 200.0471

This compound was obtained following the general procedure C. Starting from ammonium salt **1a** (117 mg, 0.36 mmol, 1.2 equiv), phenylmethanethiol **2ac** (37.3mg, 0.3 mmol), NaH (60% dispersion in mineral oil, 18 mg, 0.45 mmol, 1.5 equiv), and DMF (1.5 mL). Purification on silica gel (Pentane/Ethyl acetate gradient from 99/1 to 98/2, with 2% Et_3N) afforded **31** (57 mg, 95%) as a colorless oil.

^{1}H NMR (300 MHz, $CDCl_3$) δ 7.40 – 7.26 (m, 5H), 6.16 – 5.95 (m, 1H), 5.75 (dtd, *J* = 17.2, 2.4, 0.5 Hz, 1H), 5.50 (dq, *J* = 10.8, 0.7 Hz, 1H), 4.08 (s, 2H).

^{13}C NMR (75 MHz, $CDCl_3$) δ 136.5, 132.1 (t, *J* = 26.5 Hz), 129.2, 128.8, 127.6, 126.7, 121.2 (t, *J* = 7.6 Hz), 33.3 (t, *J* = 3.0 Hz).

^{19}F NMR (282 MHz, $CDCl_3$) δ -74.1 (dd, *J* = 9.4, 2.3 Hz, 2F).

HRMS (EI): calculated for $C_{10}H_{10}F_2S$ $[M]^+$: 200.0466, found: 200.0469.

(1,1-Difluoroallyl)(phenyl)selane (32)

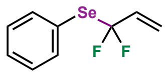

Chemical Formula: $C_9H_8F_2Se$
Exact Mass: 233.9759

This compound was obtained following the general procedure C. Starting from ammonium salt **1a** (117 mg, 0.36 mmol, 1.2 equiv), benzeneselenol **2ad** (47mg, 0.3 mmol), NaH (60% dispersion in mineral oil, 18 mg, 0.45 mmol, 1.5 equiv), and DMF (1.5 mL). Purification on silica gel (Pentane/Ethyl acetate gradient from 99/1 to 98/2, with 2% Et_3N) afforded **32** (68 mg, 97%) as a slight yellow oil.

^{1}H NMR (300 MHz, $CDCl_3$) δ 7.76 – 7.65 (m, 2H), 7.46 – 7.28 (m, 3H), 6.05 (dq, *J* = 17.2, 10.7 Hz, 1H), 5.45 (dtd, *J* = 17.2, 2.4, 0.5 Hz, 1H), 5.31 (dq, *J* = 10.9, 0.6 Hz, 1H).

^{13}C NMR (75 MHz, $CDCl_3$) δ 137.2, 133.0 (t, *J* = 24.2 Hz), 129.6, 129.3, 126.2, 122.3 (t, *J* = 288.8 Hz), 119.6 (t, *J* = 8.2 Hz).

^{19}F NMR (282 MHz, $CDCl_3$) δ -72.6 (dd, *J* = 10.7, 2.5 Hz, 2F).

HRMS (EI): calculated for $C_9H_{10}F_2Se$ $[M]^+$: 233.9918, found: 233.9914.

***N*-(1,1-difluoroallyl)-4-methyl-*N*-(prop-2-yn-1-yl)benzenesulfonamide (33)**

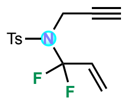

Chemical Formula: $C_{13}H_{13}F_2NO_2S$
Exact Mass: 285.0635

This compound was obtained following the general procedure C. Starting from ammonium salt **1a** (390 mg, 1.2 mmol, 1.2 equiv), 4-methyl-*N*-(prop-2-yn-1-yl)benzenesulfonamide **2ae** (209 mg, 1.0 mmol), NaH (60% dispersion in mineral oil, 60 mg, 1.5 mmol, 1.5 equiv), and DMF (5

mL). Purification on silica gel (Pentane/Ethyl acetate gradient from 90/10 to 80/20, with 2% Et_3N) afforded **33** (268 mg, 94%) as a colorless oil.

^{1}H NMR (300 MHz, $CDCl_3$) δ 7.87 – 7.76 (m, 2H), 7.33 – 7.27 (m, 2H), 6.20 – 6.03 (m, 1H), 5.73 (dtd, *J* = 17.2, 2.3, 0.4 Hz, 1H), 5.54 (dd, *J* = 10.8, 0.6 Hz, 1H), 4.33 (dt, *J* = 2.5, 1.6 Hz, 2H), 2.43 (s, 3H), 2.25 (t, *J* = 2.4 Hz, 1H).

^{13}C NMR (75 MHz, $CDCl_3$) δ 144.6, 137.4, 129.9 (t, *J* = 30.3 Hz), 129.6, 128.1, 122.5 (t, *J* = 8.0 Hz), 118.4, 78.8, 72.7, 35.1 (t, *J* = 2.5 Hz), 21.7.

^{19}F NMR (282 MHz, $CDCl_3$) δ -74.3 (d, *J* = 9.2 Hz, 2F).

HRMS (ESI): calculated for $C_{13}H_{13}F_2NO_2SNa$ $[M+Na]^+$: 308.0532, found: 308.0536.

Ethyl 1-(1,1-difluoroallyl)-1*H*-pyrazole-4-carboxylate (34)

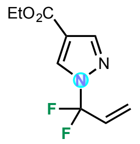

Chemical Formula: $C_9H_{10}F_2N_2O_2$
Exact Mass: 216.0710

This compound was obtained following the general procedure C. Starting from ammonium salt **1a** (117 mg, 0.36 mmol, 1.2 equiv), ethyl 1H-pyrazole-4-carboxylate **2af** (42 mg, 0.3 mmol), NaH (60% dispersion in mineral oil, 18 mg, 0.45 mmol, 1.5 equiv), and DMF (1.5 mL). Purification on silica gel (Pentane/Ethyl acetate gradient from 99/1 to 95/5, with 2% Et_3N) afforded **34** (64 mg, 99%) as a colorless oil.

^{1}H NMR (300 MHz, $CDCl_3$) δ 8.40 – 8.19 (m, 1H), 8.11 – 7.90 (m, 1H), 6.48 – 6.25 (m, 1H), 5.96 (dt, *J* = 17.2, 2.1 Hz, 1H), 5.80 (d, *J* = 10.9 Hz, 1H), 4.32 (q, *J* = 7.2 Hz, 2H), 1.35 (t, *J* = 7.1 Hz, 3H).

^{13}C NMR (75 MHz, $CDCl_3$) δ 162.3, 142.7, 131.0, 128.0 (t, *J* = 28.3 Hz), 123.6 (t, *J* = 7.6 Hz), 117.2, 60.8, 14.4.

^{19}F NMR (282 MHz, $CDCl_3$) δ -78.7 (d, *J* = 9.3 Hz, 2F).

HRMS (EI): calculated for $C_9H_{10}F_2N_2O_2$ $[M]^+$: 216.0705, found: 216.0706.

3-Chloro-1-(1,1-difluoroallyl)-1*H*-indazole (35)

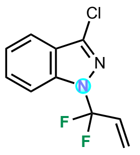

Chemical Formula: $C_{10}H_7ClF_2N_2$
Exact Mass: 228.0266

This compound was obtained following the general procedure C. Starting from ammonium salt

1a (117 mg, 0.36 mmol, 1.2 equiv), 3-chloro-1*H*-indazole **2ag** (45.6 mg, 0.3 mmol), NaH (60% dispersion in mineral oil, 18 mg, 0.45 mmol, 1.5 equiv), and DMF (1.5 mL). Purification on silica gel (Pentane/Ethyl acetate gradient from 99/1 to 95/5, with 2% Et_3N) afforded **35** (63 mg, 92%) as a colorless oil.

^{1}H NMR (300 MHz, $CDCl_3$) δ 7.87 – 7.78 (m, 1H), 7.71 (dt, *J* = 8.1, 1.0 Hz, 1H), 7.55 (ddd, *J* = 8.5, 7.0, 1.2 Hz, 1H), 7.35 (ddd, *J* = 8.0, 7.0, 0.8 Hz, 1H), 6.68 – 6.48 (m, 1H), 6.02 (dtd, *J* = 17.2, 2.1, 0.3 Hz, 1H), 5.81 (dd, *J* = 10.9, 0.4 Hz, 1H).

^{13}C NMR (75 MHz, $CDCl_3$) δ 129.4, 128.8 (t, *J* = 27.4 Hz), 123.6, 123.2, 122.6 (t, *J* = 7.5 Hz), 120.1, 118.3, 112.73 (t, *J* = 3.9 Hz).

^{19}F NMR (282 MHz, $CDCl_3$) δ -77.0 – -77.2 (m, 2F).

1-(1,1-Difluoroallyl)-1*H*-benzo[*d*]imidazole (36)

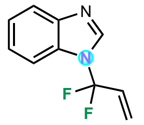

Chemical Formula: $C_{10}H_8F_2N_2$
Exact Mass: 194.0656

This compound was obtained following the general procedure C. Starting from ammonium salt **1a** (117 mg, 0.36 mmol, 1.2 equiv), 1*H*-benzo[*d*]imidazole **2ah** (35.4 mg, 0.3 mmol), NaH (60% dispersion in mineral oil, 18 mg, 0.45 mmol, 1.5 equiv), and DMF (1.5 mL). Purification on silica gel (Pentane/Ethyl acetate gradient from 99/1 to 95/5, with 2% Et_3N) afforded **36** (55 mg, 97%) as a colorless oil.

^{1}H NMR (300 MHz, $CDCl_3$) δ 8.06 (s, 1H), 7.87 – 7.77 (m, 1H), 7.68 – 7.58 (m, 1H), 7.41 – 7.32 (m, 2H), 6.41 – 6.24 (m, 1H), 5.96 (dt, *J* = 17.2, 2.4 Hz, 1H), 5.87 (d, *J* = 10.8 Hz, 1H).

^{13}C NMR (75 MHz, $CDCl_3$) δ 144.2, 139.6, 131.3, 128.6 (t, *J* = 30.8 Hz), 124.9 (t, *J* = 7.5 Hz), 124.6, 123.9, 120.9, 116.4 (t, *J* = 246.8 Hz), 112.57 (t, *J* = 2.6 Hz).

^{19}F NMR (282 MHz, $CDCl_3$) δ -76.8 (d, *J* = 8.3 Hz, 2F).

HRMS (EI): calculated for $C_{10}H_8F_2N_2$ $[M]^+$: 194.0650, found: 194.0654.

1-(1,1-Difluoroallyl)-1*H*-benzo[*d*][1,2,3]triazole (37)

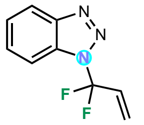

Chemical Formula: $C_9H_7F_2N_3$
Exact Mass: 195.0608

This compound was obtained following the general procedure C. Starting from ammonium salt **1a** (117 mg, 0.36 mmol, 1.2 equiv), 1*H*-benzo[*d*][1,2,3]triazole **2ai** (36 mg, 0.3 mmol), NaH (60%

dispersion in mineral oil, 18 mg, 0.45 mmol, 1.5 equiv), and DMF (1.5 mL). Purification on silica gel (Pentane/Ethyl acetate gradient from 99/1 to 1/1, with 2% Et_3N) afforded **37** (56 mg, 96%, dr = 3.5/1) as a colorless oil.

Major: **^{1}H NMR** (300 MHz, $CDCl_3$) δ 8.12 (dt, *J* = 8.3, 1.0 Hz, 1H), 7.89 – 7.83 (m, 1H), 7.61 (ddd, *J* = 8.3, 7.0, 1.0 Hz, 1H), 7.51 – 7.43 (m, 2H), 6.85 – 6.66 (m, 1H), 6.12 (dt, *J* = 17.2, 2.2 Hz, 1H), 5.93 (d, *J* = 10.9 Hz, 1H).

^{13}C NMR (75 MHz, $CDCl_3$) δ 146.3, 131.0, 129.3, 125.4, 123.8 (t, *J* = 7.7 Hz), 120.5, 119.2, 111.7 (t, *J* = 3.0 Hz).

^{19}F NMR (282 MHz, $CDCl_3$) δ -78.4 (d, *J* = 9.7 Hz, 2F).

Minor: **^{1}H NMR** (300 MHz, $CDCl_3$) δ 7.97 – 7.89 (m, 2H), 7.47 – 7.43 (m, 2H), 6.68 – 6.55 (m, 1H), 6.12 (dt, *J* = 17.2, 2.2 Hz, 1H), 5.90 (d, *J* = 10.9 Hz, 1H).

^{19}F NMR (282 MHz, $CDCl_3$) δ -79.41 (d, *J* = 9.3 Hz, 2F).

HRMS (EI): calculated for $C_9H_7F_2N_3$ $[M]^+$: 195.0603, found: 195.0601.

1-(1,1-Difluoroallyl)-4,5-diphenyl-1*H*-imidazole (38)

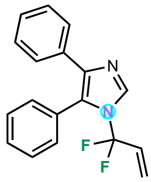

Chemical Formula: $C_{18}H_{14}F_2N_2$
Exact Mass: 296.1125

This compound was obtained following the general procedure C. Starting from ammonium salt **1a** (117 mg, 0.36 mmol, 1.2 equiv), 4,5-diphenyl-1*H*-imidazole **2aj** (66 mg, 0.3 mmol), NaH (60% dispersion in mineral oil, 18 mg, 0.45 mmol, 1.5 equiv), and DMF (1.5 mL). Purification on silica gel (Pentane/Ethyl acetate gradient from 90/10 to 2/1, with 2% Et_3N) afforded **38** (85 mg, 96%) as a white solid. M.p. 92–93 °C.

^{1}H NMR (300 MHz, $CDCl_3$) δ 7.91 (s, 1H), 7.54 – 7.30 (m, 7H), 7.25 – 7.04 (m, 3H), 5.92 (ddt, *J* = 17.6, 10.8, 8.9 Hz, 1H), 5.70 – 5.57 (m, 1H), 5.53 (d, *J* = 10.8 Hz, 1H).

^{13}C NMR (75 MHz, $CDCl_3$) δ 140.4 (d, *J* = 29.9 Hz), 134.5 (t, *J* = 3.0 Hz), 133.5, 131.9, 130.0, 129.3, 129.1 (t, *J* = 30.0 Hz), 128.7, 128.3, 127.2, 127.1, 126.7, 123.6 (t, *J* = 7.5 Hz).

^{19}F NMR (282 MHz, $CDCl_3$) δ -73.0 (d, *J* = 9.0 Hz, 2F).

HRMS (EI): calculated for $C_{18}H_{14}F_2N_2$ $[M]^+$: 296.1120, found: 296.1116.

(*Z*)-4,4'-(1-((1,1-difluoroallyl)oxy)ethene-1,2-diyl)bis(methoxybenzene) (39)

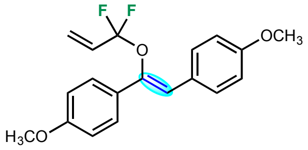

This compound was obtained following the general procedure C. Starting from ammonium salt **1a** (117 mg, 0.36 mmol, 1.2 equiv), 1,2-bis(4-methoxyphenyl)ethan-1-one **2ak** (77 mg, 0.3 mmol), NaH (60% dispersion in mineral oil, 18 mg, 0.45 mmol, 1.5 equiv), and DMF (1.5 mL). Purification on silica gel (Pentane/Ethyl acetate gradient from 99/1 to 95/5) afforded **39** (77 mg, 77%) as a colorless oil.

^{1}H NMR (300 MHz, $CDCl_3$) δ 7.63 – 7.56 (m, 2H), 7.56 – 7.49 (m, 2H), 6.96 – 6.84 (m, 4H), 6.36 (s, 1H), 6.06 – 5.90 (m, 1H), 5.84 – 5.73 (m, 1H), 5.50 (dd, *J* = 10.7, 0.8 Hz, 1H), 3.84 (s, 3H), 3.83 (s, 3H).

^{13}C NMR (75 MHz, $CDCl_3$) δ 159.8, 159.0, 144.9 (t, *J* = 2.3 Hz), 130.5, 130.2, 129.3 (t, *J* = 33.7 Hz), 127.5, 127.4, 121.7 (t, *J* = 257.3 Hz), 121.6 (t, *J* = 6.5 Hz), 117.7, 113.9, 113.7, 55.4, 55.3.

^{19}F NMR (282 MHz, $CDCl_3$) δ -65.5 (dd, *J* = 7.2, 1.9 Hz, 2F).

HRMS (EI): calculated for $C_{19}H_{18}F_2O_3$ $[M]^+$: 332.1219, found: 332.1219.

2-(1-((1,1-Difluoroallyl)oxy)vinyl)naphthalene (40)

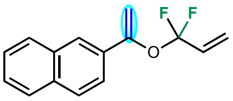

This compound was obtained following the general procedure C. Starting from ammonium salt **1a** (117 mg, 0.36 mmol, 1.2 equiv), 1-(naphthalen-2-yl)ethan-1-one **2al** (51 mg, 0.3 mmol), NaH (60% dispersion in mineral oil, 18 mg, 0.45 mmol, 1.5 equiv), and DMF (1.5 mL). Purification on silica gel (Pentane/Ethyl acetate gradient from 99/1 to 95/5, with 2% Et_3N) afforded **40** (54 mg, 74%) as a colorless oil.

^{1}H NMR (300 MHz, $CDCl_3$) δ 8.03 (d, *J* = 1.8 Hz, 1H), 7.90 – 7.77 (m, 3H), 7.66 (dd, *J* = 8.7, 1.9 Hz, 1H), 7.54 – 7.45 (m, 2H), 6.20 – 6.04 (m, 1H), 6.02 – 5.93 (m, 1H), 5.63 (dd, *J* = 10.5, 0.9 Hz, 1H), 5.48 (dt, *J* = 2.7, 0.8 Hz, 1H), 5.25 (dt, *J* = 2.9, 1.6 Hz, 1H).

^{13}C NMR (75 MHz, $CDCl_3$) δ 152.2, 133.6, 133.2, 132.5, 129.5 (t, *J* = 34.0 Hz), 128.7, 128.2, 127.7, 126.7, 126.6, 124.8, 123.1, 121.9 (t, *J* = 6.5 Hz), 121.2 (t, *J* = 258.0 Hz), 99.8 (t, *J* = 1.8 Hz).

^{19}F NMR (282 MHz, $CDCl_3$) δ -69.1 (d, *J* = 6.5 Hz, 2F).

HRMS (EI): calculated for $C_{15}H_{12}F_2O$ $[M]^+$: 246.0851, found: 246.0850.

1-(1-((1,1-Difluoroallyl)oxy)vinyl)-3-(trifluoromethyl)benzene (41)

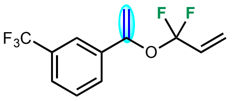

Chemical Formula: $C_{12}H_9F_5O$
Exact Mass: 264.0574

This compound was obtained following the general procedure C. Starting from ammonium salt **1a** (117 mg, 0.36 mmol, 1.2 equiv), 1-(3-(trifluoromethyl)phenyl)ethan-1-one **2am** (56 mg, 0.3 mmol), NaH (60% dispersion in mineral oil, 18 mg, 0.45 mmol, 1.5 equiv), and DMF (1.5 mL). Purification on silica gel (Pentane/Ethyl acetate gradient from 99/1 to 95/5, with 2% Et_3N) afforded **41** (64 mg, 81%) as a colorless oil.

^{1}H NMR (300 MHz, $CDCl_3$) δ 7.83 – 7.76 (m, 1H), 7.76 – 7.70 (m, 1H), 7.64 – 7.56 (m, 1H), 7.48 (tt, *J* = 7.8, 0.7 Hz, 1H), 6.15 – 5.98 (m, 1H), 5.98 – 5.86 (m, 1H), 5.62 (dd, *J* = 10.5, 0.9 Hz, 1H), 5.40 (dt, *J* = 2.9, 0.8 Hz, 1H), 5.24 (dt, *J* = 3.0, 1.6 Hz, 1H).

^{13}C NMR (75 MHz, $CDCl_3$) δ 150.9, 136.1, 131.1(d, *J* = 32.3 Hz), 129.2(t, *J* = 33.7 Hz), 129.1, 128.6, 125.68 (dd, *J* = 7.5, 3.8 Hz), 122.3 (d, *J* = 7.5, 3.9 Hz), 122.1 (t, *J* = 6.7 Hz), 121.1 (t, *J* = 258.8 Hz), 100.5 (t, *J* = 1.8 Hz).

^{19}F NMR (282 MHz, $CDCl_3$) δ -62.8 (s, 3F), -69.8 (d, *J* = 6.3 Hz, 2F).

HRMS (EI): calculated for $C_{12}H_9F_5O$ $[M]^+$: 264.0568, found: 264.0569.

1-Bromo-3-(1-((1,1-difluoroallyl)oxy)vinyl)benzene (42)

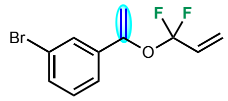

Chemical Formula: $C_{11}H_9BrF_2O$
Exact Mass: 273.9805

This compound was obtained following the general procedure C. Starting from ammonium salt **1a** (390 mg, 1.2 mmol, 1.2 equiv), 1-(3-bromophenyl)ethan-1-one **2an** (199 mg, 1.0 mmol), NaH (60% dispersion in mineral oil, 60 mg, 1.5 mmol, 1.5 equiv), and DMF (5 mL). Purification on silica gel (Pentane/Ethyl acetate gradient from 99/1 to 95/5) afforded **42** (273 mg, 99%) as a colorless oil.

^{1}H NMR (300 MHz, $CDCl_3$) δ 7.68 (t, *J* = 1.8 Hz, 1H), 7.51 – 7.40 (m, 2H), 7.26 – 7.18 (m, 1H), 6.13 – 5.98 (m, 1H), 5.96 – 5.87 (m, 1H), 5.63 – 5.58 (m, 1H), 5.33 (dt, *J* = 2.8, 0.8 Hz, 1H), 5.18 (dt, *J* = 2.9, 1.6 Hz, 1H).

^{13}C NMR (75 MHz, $CDCl_3$) δ 150.8, 137.3, 132.0, 130.0, 129.3 (t, *J* = 33.7 Hz), 128.5, 124.1, 122.7, 122.0 (t, *J* = 6.5 Hz), 121.1, 100.3 (t, *J* = 1.9 Hz).

^{19}F NMR (282 MHz, $CDCl_3$) δ -69.66 (d, *J* = 6.5 Hz, 2F).

HRMS (EI): calculated for $C_{11}H_9{}^{81}BrF_2O$ $[M]^+$: 275.9779, found: 275.9784.

3,3-Difluoro-2,2-diphenylpent-4-enenitrile (43)

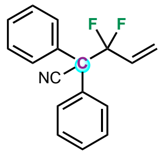

Chemical Formula: $C_{17}H_{13}F_2N$
Exact Mass: 269.1016

This compound was obtained following the general procedure C. Starting from ammonium salt **1a** (117 mg, 0.36 mmol, 1.2 equiv), 2,2-diphenylacetonitrile **2ao** (58 mg, 0.3 mmol), NaH (60% dispersion in mineral oil, 18 mg, 0.45 mmol, 1.5 equiv), and DMF (1.5 mL). Purification on silica gel (Pentane/Ethyl acetate gradient from 99/1 to 95/5) afforded **43** (80 mg, 99%) as a colorless oil.

^{1}H NMR (300 MHz, $CDCl_3$) δ 7.62 – 7.47 (m, 4H), 7.45 – 7.33 (m, 6H), 6.09 – 5.90 (m, 1H), 5.85 – 5.75 (m, 1H), 5.62 – 5.54 (m, 1H).

^{13}C NMR (75 MHz, $CDCl_3$) δ 134.4, 129.5 (t, *J* = 25.1 Hz), 129.1 (t, *J* = 2.2 Hz), 129.0, 128.9, 123.7 (t, *J* = 9.0 Hz), 119.1.

^{19}F NMR (282 MHz, $CDCl_3$) δ -98.7 (s, 2F).

HRMS (EI): calculated for $C_{17}H_{13}F_2N$ $[M]^+$: 269.1011, found: 269.1009.

(8*R*,9*S*,13*S*,14*S*)-3-((1,1-difluoroallyl)oxy)-13-methyl-6,7,8,9,11,12,13,14,15,16-decahydro-17*H*-cyclopenta[*a*]phenanthren-17-one (44)

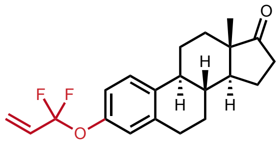

Chemical Formula: $C_{21}H_{24}F_2O_2$
Exact Mass: 346.1744

This compound was obtained following the general procedure C. Starting from ammonium salt **1a** (117 mg, 0.36 mmol, 1.2 equiv), Estrone **2ap** (81 mg, 0.3 mmol), NaH (60% dispersion in mineral oil, 18 mg, 0.45 mmol, 1.5 equiv), and DMF (1.5 mL). Purification on silica gel (Pentane/Ethyl acetate gradient from 95/5 to 90/10) afforded **44** (100 mg, 97%) as a white solid. M.p. 150–152 °C.

^{1}H NMR (300 MHz, $CDCl_3$) δ 7.25 (dd, *J* = 8.2, 1.3 Hz, 1H), 7.05 – 6.87 (m, 2H), 6.14 – 5.97 (m, 1H), 5.97 – 5.84 (m, 1H), 5.58 (dd, *J* = 10.4, 1.1 Hz, 1H), 3.01 – 2.81 (m, 2H), 2.51 (dd, *J* = 18.4, 8.5 Hz, 1H), 2.45 – 2.35 (m, 1H), 2.35 – 2.22 (m, 1H), 2.22 – 1.92 (m, 4H), 1.65 – 1.39 (m, 6H),

0.91 (s, 3H).

^{13}C NMR (75 MHz, $CDCl_3$) δ 220.9, 148.3, 138.0, 137.2, 129.7 (t, J = 33.9 Hz), 126.4, 122.1, 121.8 (t, J = 6.5 Hz), 120.8 (t, J = 257.3 Hz), 119.3, 50.6, 48.1, 44.2, 38.2, 36.0, 31.7, 29.6, 26.5, 25.9, 21.7, 14.0.

^{19}F NMR (282 MHz, $CDCl_3$) δ -68.5 (d, J = 6.2 Hz, 2F).

HRMS (EI): calculated for $C_{21}H_{24}F_2O_2$ $[M]^+$: 346.1739, found: 346.1743.

(*R*)-6-((1,1-difluoroallyl)oxy)-2,5,7,8-tetramethyl-2-((4*R*,8*R*)-4,8,12-trimethyltridec-yl)chromane (45)

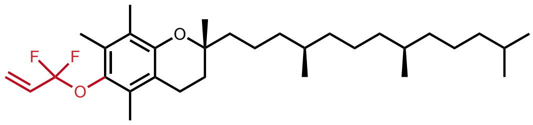

Chemical Formula: $C_{32}H_{52}F_2O_2$
Exact Mass: 506.3935

This compound was obtained following the general procedure C. Starting from ammonium salt **1a** (117 mg, 0.36 mmol, 1.2 equiv), (+)-*α*-Tocopherol **2aq** (129.2 mg, 0.3 mmol), NaH (60% dispersion in mineral oil, 18 mg, 0.45 mmol, 1.5 equiv), and DMF (1.5 mL). Purification on silica gel (Pentane/Ethyl acetate gradient from 99/1 to 98/2, with 2% Et_3N) afforded **45** (152 mg, 99%) as a colorless oil.

^{1}H NMR (300 MHz, $CDCl_3$) δ 6.09 (ddt, J = 17.3, 10.2, 6.0 Hz, 1H), 6.00 – 5.89 (m, 1H), 5.56 (dd, J = 10.4, 1.2 Hz, 1H), 2.59 (t, J = 6.8 Hz, 2H), 2.14 (s, 3H), 2.10 (s, 3H), 2.09 (s, 3H), 1.90 – 1.69 (m, 2H), 1.59 – 1.51 (m, 3H), 1.42 – 1.23 (m, 13H), 1.19 – 1.02 (m, 7H), 0.89 – 0.82 (m, 13H).

^{13}C NMR (75 MHz, $CDCl_3$) δ 149.4, 130.0, 129.6, 127.9, 123.2, 121.0 117.7, 75.2, 40.2, 39.5, 37.6, 37.6, 37.45, 33.0, 32.9, 31.3, 28.1, 25.0, 24.6, 24.1, 22.9, 22.8, 21.2, 20.8, 19.9, 19.8, 14.4, 13.5, 12.0.

^{19}F NMR (282 MHz, $CDCl_3$) δ -67.1 (s, 2F).

HRMS (EI): calculated for $C_{32}H_{52}F_2O_2$ $[M]^+$: 506.3930, found: 506.3935.

(8*R*,9*S*,13*S*,14*S*,17*S*)-3-((1,1-difluoroallyl)oxy)-13-methyl-7,8,9,11,12,13,14,15,16,17-decahydro-6*H*-cyclopenta[*a*]phenanthren-17-yl acetate (46)

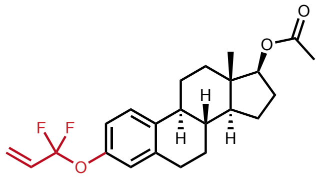

Chemical Formula: $C_{23}H_{28}F_2O_3$
Exact Mass: 390.2007

This compound was obtained following the general procedure C. Starting from ammonium salt

1a (117 mg, 0.36 mmol, 1.2 equiv), (8*R*,9*S*,13*S*,14*S*,17*S*)-3-hydroxy-13-methyl-7,8,9,11,12,13,14,15,16,17-decahydro-6*H*-cyclopenta[*a*]phenanthren-17-yl acetate **2ar** (94.2 mg, 0.3 mmol), NaH (60% dispersion in mineral oil, 18 mg, 0.45 mmol, 1.5 equiv), and DMF (1.5 mL). Purification on silica gel (Pentane/Ethyl acetate gradient from 95/5 to 90/10) afforded **46** (114 mg, 97%) as a white solid. M.p. 99–100 °C.

^{1}H NMR (300 MHz, $CDCl_3$) δ 7.24 (dd, *J* = 8.6, 1.0 Hz, 1H), 7.02 – 6.94 (m, 1H), 6.94 – 6.88 (m, 1H), 6.14 – 5.96 (m, 1H), 5.96 – 5.85 (m, 1H), 5.57 (dd, *J* = 10.4, 1.0 Hz, 1H), 4.69 (dd, *J* = 9.2, 7.6 Hz, 1H), 2.86 (dd, *J* = 8.7, 4.1 Hz, 2H), 2.37 – 2.13 (m, 3H), 2.06 (s, 3H), 1.95 – 1.83 (m, 2H), 1.82 – 1.65 (m, 1H), 1.64 – 1.19 (m, 8H), 0.83 (s, 3H).

^{13}C NMR (75 MHz, $CDCl_3$) δ 171.4, 148.1, 138.2, 137.7, 129.7 (t, *J* = 34.0 Hz), 126.4, 122.1, 121.7 (t, *J* = 6.0 Hz), 119.2, 82.8, 50.0, 44.1, 43.0, 38.4, 37.0, 29.7, 27.7, 27.2, 26.2, 23.4, 21.3, 12.2.

^{19}F NMR (282 MHz, $CDCl_3$) δ -68.5 (d, *J* = 6.4 Hz, 2F).

HRMS (EI): calculated for $C_{23}H_{28}F_2O_3$ $[M]^+$: 390.2001, found: 390.2007.

(*E*)-4,4'-(hex-3-ene-3,4-diyl)bis(((1,1-difluoroallyl)oxy)benzene) (47)

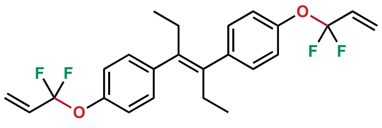

This compound was obtained following the general procedure C. Starting from ammonium salt **1a** (117 mg, 0.36 mmol, 2.4 equiv), diethylstilbestrol **2as** (40.2 mg, 0.15 mmol), NaH (60% dispersion in mineral oil, 18 mg, 0.45 mmol, 1.5 equiv), and DMF (1.5 mL). Purification on silica gel (Pentane/Ethyl acetate gradient from 98/2 to 95/5) afforded **47** (58 mg, 92%) as a colorless oil.

^{1}H NMR (300 MHz, $CDCl_3$) δ 7.24 – 7.14 (m, 8H), 6.18 – 6.02 (m, 2H), 5.95 (dtd, *J* = 17.3, 1.9, 1.0 Hz, 2H), 5.61 (dd, *J* = 10.5, 1.0 Hz, 2H), 2.13 (q, *J* = 7.4 Hz, 4H), 0.77 (t, *J* = 7.4 Hz, 6H).

^{13}C NMR (75 MHz, $CDCl_3$) δ 148.9 (t, *J* = 1.7 Hz), 139.8, 138.9, 129.8, 129.7 (t, *J* = 33.9 Hz), 121.8 (t, *J* = 6.5 Hz), 121.5, 120.9 (t, *J* = 257.3 Hz), 28.6, 13.4.

^{19}F NMR (282 MHz, $CDCl_3$) δ -68.7 (d, *J* = 6.2 Hz, 4F).

HRMS (EI): calculated for $C_{24}H_{24}F_4O_2$ $[M]^+$: 420.1707, found: 420.1712.

(*Z*)-1-((1,1-difluoroallyl)oxy)-3,7-dimethylocta-2,6-diene (48)

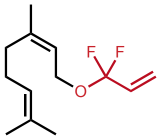

This compound was obtained following the general procedure C. Starting from ammonium salt **1a** (117 mg, 0.36 mmol, 1.2 equiv), (*Z*)-Nerol **2at** (46.3 mg, 0.3 mmol), NaH (60% dispersion in mineral oil, 18 mg, 0.45 mmol, 1.5 equiv), and DMF (1.5 mL). Purification on silica gel (Pentane/Ethyl acetate gradient from 99/1 to 98/2, with 2% Et_3N) afforded **48** (66 mg, 97%) as a colorless oil.

^{1}H NMR (300 MHz, $CDCl_3$) δ 5.99 – 5.83 (m, 1H), 5.82 – 5.72 (m, 1H), 5.46 (dd, *J* = 10.4, 1.3 Hz, 1H), 5.41 – 5.32 (m, 1H), 5.14 – 5.05 (m, 1H), 4.41 (dq, *J* = 7.3, 1.0 Hz, 2H), 2.13 – 2.03 (m, 4H), 1.79 – 1.75 (m, 3H), 1.71 – 1.65 (m, 3H), 1.62 – 1.58 (m, 3H).

^{13}C NMR (75 MHz, $CDCl_3$) δ 142.5, 132.4, 130.1 (t, *J* = 34.8 Hz), 123.7, 121.6 (t, *J* = 34.8 Hz), 120.9 (t, *J* = 6.5 Hz), 119.6, 60.2, 60.1, 60.0, 32.3, 26.8, 25.8, 23.7, 17.8.

^{19}F NMR (282 MHz, $CDCl_3$) δ -72.49 (dd, *J* = 6.1, 1.9 Hz, 2F).

HRMS (EI): calculated for $C_{13}H_{20}F_2O$ $[M]^+$: 230.1477, found: 230.1485.

(3*R*,3a*R*,6*S*,6a*R*)-3,6-bis((1,1-difluoroallyl)oxy)hexahydrofuro[3,2-*b*]furan (49)

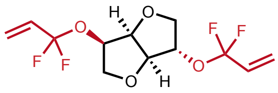

This compound was obtained following the general procedure C. Starting from ammonium salt **1a** (117 mg, 0.36 mmol, 2.4 equiv), Isosorbide **2au** (22 mg, 0.15 mmol), NaH (60% dispersion in mineral oil, 18 mg, 0.45 mmol, 3.0 equiv), and DMF (1.5 mL). Purification on silica gel (Pentane/Ethyl acetate gradient from 99/1 to 95/5, with 2% Et_3N) afforded **49** (44 mg, 98%) as a colorless oil.

^{1}H NMR (300 MHz, $CDCl_3$) δ 6.03 – 5.73 (m, 4H), 5.53 (dd, *J* = 7.3, 1.3 Hz, 1H), 5.51 – 5.47 (m, 1H), 4.86 – 4.74 (m, 2H), 4.69 (td, *J* = 4.3, 1.2 Hz, 1H), 4.62 (dd, *J* = 4.0, 1.2 Hz, 1H), 4.09 – 3.99 (m, 3H), 3.66 (t, *J* = 8.6 Hz, 1H).

^{13}C NMR (75 MHz, $CDCl_3$) δ 129.3 (t, *J* = 33.9 Hz), 129.1 (t, *J* = 33.6 Hz), 121.9 (t, *J* = 6.5 Hz), 121.6 (t, *J* = 6.5 Hz), 121.5 (t, *J* = 256.5 Hz), 121.3 (t, *J* = 255.8 Hz), 86.4, 81.0, 78.2 (t, *J* = 4.6 Hz), 74.3, 73.6, (t, *J* = 4.3 Hz), 69.4.

^{19}F NMR (282 MHz, $CDCl_3$) δ -70.1 – -70.7 (m, 3F), -72.2 (dd, *J* = 149.2, 6.2 Hz, 1F).

HRMS (EI): calculated for $C_{12}H_{14}F_4O_4$ $[M]^+$: 298.0823, found: 298.0831.

(3a*R*,5*R*,6*S*,6a*R*)-6-((1,1-difluoroallyl)oxy)-5-((*R*)-2,2-dimethyl-1,3-dioxolan-4-yl)-2,2-dimethyltetrahydrofuro[2,3-*d*][1,3]dioxole (50)

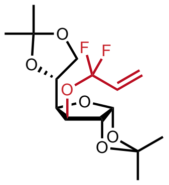

Chemical Formula: $C_{15}H_{22}F_2O_6$
Exact Mass: 336.1384

This compound was obtained following the general procedure C. Starting from ammonium salt **1a** (117 mg, 0.36 mmol, 1.2 equiv), 1,2:5,6-Di-*O*-isopropylidene-α-D-glucofuranose **2av** (78 mg, 0.3 mmol), NaH (60% dispersion in mineral oil, 18 mg, 0.45 mmol, 1.5 equiv), and DMF (1.5 mL). Purification on silica gel (Pentane/Ethyl acetate gradient from 99/1 to 95/5, with 2% Et_3N) afforded **50** (92 mg, 92%) as a colorless oil.

^{1}**H NMR** (300 MHz, $CDCl_3$) δ 5.99 – 5.83 (m, 2H), 5.83 – 5.73 (m, 1H), 5.52 (dd, *J* = 10.3, 1.3 Hz, 1H), 4.77 (dd, *J* = 2.2, 0.6 Hz, 1H), 4.67 (d, *J* = 3.8 Hz, 1H), 4.31 – 4.20 (m, 2H), 4.08 – 3.95 (m, 2H), 1.51 (d, *J* = 0.7 Hz, 3H), 1.42 (d, *J* = 0.7 Hz, 3H), 1.33 (d, *J* = 0.7 Hz, 3H), 1.32 (d, *J* = 0.7 Hz, 3H).

^{13}**C NMR** (75 MHz, $CDCl_3$) δ 129.2 (t, *J* = 34.0 Hz), 121.8 (t, *J* = 6.5 Hz), 112.4, 109.2, 105.1, 83.9, 80.0, 76.7 – 76.5 (m), 72.7, 66.8, 26.9, 26.8, 26.4, 25.5.

^{19}**F NMR** (282 MHz, $CDCl_3$) δ -68.8 – -69.9 (m, 1F), -72.0 – -73.0 (m, 1F).

HRMS (EI): calculated for $C_{14}H_{19}F_2O_6$ $[M-CH_3]^+$: 321.1144, found: 321.1145.

(3a*R*,5*R*,5a*S*,8a*S*,8b*R*)-5-(((1,1-difluoroallyl)oxy)methyl)-2,2,7,7-tetramethyltetra-hydro-5*H*-bis([1,3]dioxolo)[4,5-*b*:4',5'-*d*]pyran (51)

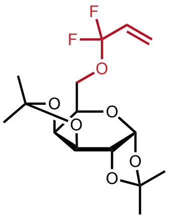

Chemical Formula: $C_{15}H_{22}F_2O_6$
Exact Mass: 336.1384

This compound was obtained following the general procedure C. Starting from ammonium salt **1a** (117 mg, 0.36 mmol, 1.2 equiv), 1,2:3,4-Di-O-isopropylidene-α-D-galactopyranose **2aw** (78 mg, 0.3 mmol), NaH (60% dispersion in mineral oil, 18 mg, 0.45 mmol, 1.5 equiv), and DMF (1.5 mL). Purification on silica gel (Pentane/Ethyl acetate gradient from 99/1 to 95/5, with 2% Et_3N) afforded **51** (92 mg, 92%) as a white solid. M.p. 54–55 °C.

^{1}**H NMR** (300 MHz, $CDCl_3$) δ 6.02 – 5.85 (m, 1H), 5.84 – 5.73 (m, 1H), 5.54 (d, *J* = 5.0 Hz, 1H),

5.47 (dd, J = 10.4, 1.2 Hz, 1H), 4.62 (dd, J = 7.9, 2.5 Hz, 1H), 4.32 (dd, J = 5.0, 2.5 Hz, 1H), 4.26 (dd, J = 7.9, 1.5 Hz, 1H), 4.14 – 3.97 (m, 3H), 1.53 (d, J = 0.7 Hz, 3H), 1.45 (d, J = 0.7 Hz, 3H), 1.34 (d, J = 0.7 Hz, 3H), 1.33 (d, J = 0.7 Hz, 3H).

^{13}C NMR (75 MHz, $CDCl_3$) δ 129.7 (t, J = 34.4 Hz), 121.5 (t, J = 255.0 Hz), 121.3 (t, J = 6.5 Hz), 109.7, 108.9, 96.4, 71.0, 70.8, 70.7, 66.7, 62.5 (t, J = 5.8 Hz), 26.1, 26.1, 25.1, 24.6.

^{19}F NMR (282 MHz, $CDCl_3$) δ -72.6 – -72.9 (m, 2F).

HRMS (EI): calculated for $C_{14}H_{19}F_2O_6$ $[M-CH_3]^+$: 321.1144, found: 321.1144.

(8*R*,9*S*,13*S*,14*S*,17*R*)-17-((1,1-difluoroallyl)oxy)-17-ethynyl-3-methoxy-13-methyl-7,8,9,11,12,13,14,15,16,17-decahydro-6*H*-cyclopenta[*a*]phenanthrene (52)

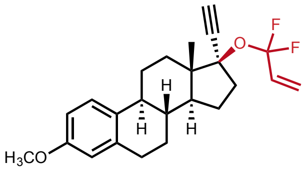

Chemical Formula: $C_{24}H_{28}F_2O_2$
Exact Mass: 386.2057

This compound was obtained following the general procedure C. Starting from ammonium salt **1a** (117 mg, 0.36 mmol, 1.2 equiv), Mestranol **2ax** (93.1 mg, 0.3 mmol), NaH (60% dispersion in mineral oil, 18 mg, 0.45 mmol, 1.5 equiv), and DMF (1.5 mL). Purification on silica gel (Pentane/Ethyl acetate gradient from 100/0 to 99/1, with 2% Et_3N) afforded **52** (113 mg, 98%) as a white solid. M.p. 99–101 °C.

^{1}H NMR (300 MHz, $CDCl_3$) δ 7.22 (d, J = 8.5 Hz, 1H), 6.72 (dd, J = 8.6, 2.8 Hz, 1H), 6.63 (d, J = 2.8 Hz, 1H), 6.04 – 5.86 (m, 1H), 5.80 – 5.70 (m, 1H), 5.44 (dd, J = 10.5, 1.0 Hz, 1H), 3.78 (s, 3H), 2.95 – 2.79 (m, 2H), 2.70 (s, 1H), 2.55 – 2.17 (m, 4H), 2.08 – 1.94 (m, 1H), 1.93 – 1.70 (m, 4H), 1.54 – 1.32 (m, 4H), 0.92 (s, 3H).

^{13}C NMR (75 MHz, $CDCl_3$) δ 157.6, 138.1, 131.9 (d, J = 107.7 Hz), 126.5, 120.2 (t, J = 6.8 Hz), 114.0, 111.7, 85.2 – 84.2 (m), 76.2, 55.4, 48.8, 47.7, 43.6, 39.4, 37.9, 32.8, 30.0, 27.5, 26.5, 23.3, 13.5.

^{19}F NMR (282 MHz, $CDCl_3$) δ -62.7 (dd, J = 154.8, 5.5 Hz, 1F), -64.4 (dd, J = 154.5, 5.8 Hz, 1F).

HRMS (EI): calculated for $C_{24}H_{28}F_2O_2$ $[M]^+$: 386.2052, found: 386.2059.

(8*R*,9*S*,10*R*,13*S*,14*S*,17*R*)-17-((1,1-difluoroallyl)oxy)-17-ethynyl-13-methyl-2,3,6,7,8,9,10,11,12,13,14,15,16,17-tetradecahydro-1*H*-cyclopenta[*a*]phenanthrene (53)

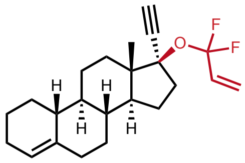

Chemical Formula: $C_{23}H_{30}F_2O$
Exact Mass: 360.2265

This compound was obtained following the general procedure C. Starting from ammonium salt **1a** (117 mg, 0.36 mmol, 1.2 equiv), Lynestrenol **2ay** (85.3 mg, 0.3 mmol), NaH (60% dispersion in mineral oil, 18 mg, 0.45 mmol, 1.5 equiv), and DMF (1.5 mL). Purification on silica gel (Pentane/Ethyl acetate gradient from 100/0 to 99/1, with 2% Et_3N) afforded **53** (107 mg, 99%) as a white solid. M.p. 85–86 °C.

^{1}H NMR (300 MHz, $CDCl_3$) δ 6.01 – 5.84 (m, 1H), 5.79 – 5.68 (m, 1H), 5.42 (dd, *J* = 10.5, 1.0 Hz, 1H), 5.39 (s, 1H), 2.64 (s, 1H), 2.47 – 2.25 (m, 2H), 2.25 – 2.14 (m, 1H), 2.09 – 1.84 (m, 5H), 1.84 – 1.56 (m, 7H), 1.45 – 1.30 (m, 2H), 1.27 – 1.10 (m, 4H), 0.91 (s, 3H), 0.77 – 0.63 (m, 1H).

^{13}C NMR (75 MHz, $CDCl_3$) δ 140.4, 131.0 (d, *J* = 35.7 Hz), 120.2, 120.1 (t, *J* = 6.7 Hz), 85.1, 76.1 – 75.9 (m), 57.6, 50.0, 48.7, 47.6, 42.1, 41.5, 37.8, 35.6, 32.7, 31.8, 28.9, 26.1, 25.6, 23.4, 22.2, 13.6.

^{19}F NMR (282 MHz, $CDCl_3$) δ -62.7 (dd, *J* = 154.8, 5.6 Hz, 1F), -64.4 (dd, *J* = 154.8, 5.9 Hz, 1F).

HRMS (EI): calculated for $C_{23}H_{30}F_2O$ $[M]^+$: 360.2259, found: 360.2267.

(1*S*,2*S*,4*S*,5*R*)-2-((*S*)-((1,1-difluoroallyl)oxy)(6-methoxyquinolin-4-yl)methyl)-5-vinylquinuclidine (54)

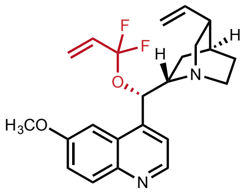

Chemical Formula: $C_{23}H_{26}F_2N_2O_2$
Exact Mass: 400.1962

This compound was obtained following the general procedure C. Starting from ammonium salt **1a** (117 mg, 0.36 mmol, 1.2 equiv), Quinine **2az** (97.3 mg, 0.3 mmol), NaH (60% dispersion in mineral oil, 18 mg, 0.45 mmol, 1.5 equiv), and DMF (1.5 mL). Purification on silica gel (Pentane/Ethyl acetate gradient from 99/1 to 95/5, with 2% Et_3N) afforded **54** (118 mg, 98%) as a slight yellow oil.

^{1}H NMR (300 MHz, $CDCl_3$) δ 8.74 (d, *J* = 4.5 Hz, 1H), 8.03 (d, *J* = 9.3 Hz, 1H), 7.47 – 7.35 (m, 2H), 7.28 (d, *J* = 12.6 Hz, 1H), 6.10 – 5.64 (m, 4H), 5.51 (dd, *J* = 10.3, 1.2 Hz, 1H), 5.02 – 4.88 (m, 2H), 3.95 (s, 3H), 3.38 – 3.13 (m, 2H), 3.05 (dd, *J* = 13.8, 10.0 Hz, 1H), 2.77 – 2.51 (m, 2H), 2.26 (s,

1H), 1.89 – 1.81 (m, 1H), 1.80 – 1.61 (m, 3H), 1.62 – 1.45 (m, 1H).

^{13}C NMR (75 MHz, $CDCl_3$) δ 158.0, 147.6, 144.9 (d, *J* = 3.8 Hz), 144.8, 141.9, 132.1, 130.1, 129.7, 126.4, 121.8, 121.4 (t, *J* = 6.7 Hz), 119.0, 114.6, 60.0, 57.3, 55.8, 42.5 (dd, *J* = 4.4, 2.3 Hz), 39.9, 27.9, 27.8.

^{19}F NMR (282 MHz, $CDCl_3$) δ -68.2 – -70.8 (m, 2F).

HRMS (ESI): calculated for $C_{23}H_{27}F_2N_2O_2$ $[M+H]^+$: 401.2040, found: 401.2030.

(8*R*,9*S*,10*R*,13*S*,14*S*,17*S*)-17-((1,1-difluoroallyl)oxy)-10,13-dimethyl-1,2,6,7,8,9,10,11,12,13,14,15,16,17-tetradecahydro-3*H*-cyclopenta[*a*]phenanthren-3-one (55)

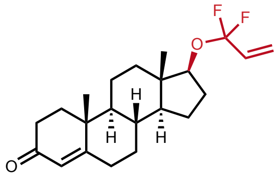

Chemical Formula: $C_{22}H_{30}F_2O_2$
Exact Mass: 364.2214

This compound was obtained following the general procedure C. Starting from ammonium salt **1a** (117 mg, 0.36 mmol, 1.2 equiv), Testosterone **2ba** (86.5 mg, 0.3 mmol), NaH (60% dispersion in mineral oil, 18 mg, 0.45 mmol, 1.5 equiv), and DMF (1.5 mL). Purification on silica gel (Pentane/Ethyl acetate gradient from 90/10 to 3/1, with 2% Et_3N) afforded **55** (72 mg, 67%) as a colorless oil.

^{1}H NMR (300 MHz, $CDCl_3$) δ 5.98 – 5.82 (m, 1H), 5.80 – 5.69 (m, 2H), 5.44 (dd, *J* = 10.5, 1.2 Hz, 1H), 4.14 (dd, *J* = 9.1, 7.8 Hz, 1H), 2.50 – 2.20 (m, 4H), 2.18 – 1.96 (m, 2H), 1.94 – 1.80 (m, 2H), 1.74 – 1.52 (m, 5H), 1.49 – 1.26 (m, 2H), 1.19 (s, 3H), 1.17 – 0.87 (m, 4H), 0.83 (s, 3H).

^{13}C NMR (75 MHz, $CDCl_3$) δ 199.6, 171.1, 130.4 (t, *J* = 35.6 Hz), 124.1, 121.3 (t, *J* = 253.5 Hz), 120.58 (t, *J* = 6.4 Hz), 82.8 (t, *J* = 3.8 Hz), 54.0, 50.3, 42.5, 38.8, 36.4, 35.9, 35.6, 34.1, 32.9, 31.6, 28.9, 23.5, 20.6, 17.6, 11.7.

^{19}F NMR (282 MHz, $CDCl_3$) δ -68.5 (t, *J* = 6.1 Hz, 2F).

HRMS (ESI): calculated for $C_{22}H_{30}F_2O_2Na$ $[M+Na]^+$: 387.2106, found: 387.2105.

***N*-allyl-*N*-(1,1-difluoroallyl)-4-(5-(*p*-tolyl)-3-(trifluoromethyl)-1*H*-pyrazol-1-yl)benzenesulfonamide (56)**

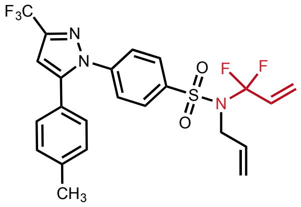

Chemical Formula: $C_{23}H_{20}F_5N_3O_2S$
Exact Mass: 497.1196

This compound was obtained following the general procedure C. Starting from ammonium salt **1a** (117 mg, 0.36 mmol, 1.2 equiv), *N*-allyl-4-(5-(*p*-tolyl)-3-(trifluoromethyl)-1*H*-pyrazol-1-yl)benzenesulfonamide **2bb** (84.2 mg, 0.3 mmol), NaH (60% dispersion in mineral oil, 18 mg, 0.45 mmol, 1.5 equiv), and DMF (1.5 mL). Purification on silica gel (Pentane/Ethyl acetate gradient from 95/5 to 90/10, with 2% Et_3N) afforded **56** (95 mg, 96%) as a colorless oil.

^{1}H NMR (300 MHz, $CDCl_3$) δ 8.01 – 7.89 (m, 2H), 7.55 – 7.43 (m, 2H), 7.23 – 7.06 (m, 4H), 6.82 – 6.66 (m, 2H), 6.39 (dd, *J* = 16.7, 1.6 Hz, 1H), 5.95 – 5.82 (m, 1H), 5.79 (dd, *J* = 10.4, 1.6 Hz, 1H), 5.34 – 5.23 (m, 2H), 4.50 (dt, *J* = 5.3, 1.6 Hz, 2H), 2.39 (s, 3H).

^{13}C NMR (75 MHz, $CDCl_3$) δ 145.4, 143.0, 140.1, 140.0, 133.7, 130.4 (t, *J* = 30.6 Hz), 129.9, 128.9, 128.9, 125.8, 125.4, 122.0 (t, *J* = 7.9 Hz), 118.7, 106.5, 48.7, 21.5.

^{19}F NMR (282 MHz, $CDCl_3$) δ -62.5, -73.8 (d, *J* = 9.7 Hz, 2F).

HRMS (EI): calculated for $C_{23}H_{20}F_2N_3O_2S$ $[M]^+$: 497.1191, found: 497.1193.

Methyl (*S*)-2-((*tert*-butoxycarbonyl)amino)-3-(4-((1,1-difluoroallyl)oxy)-3-iodophenyl)propanoate (57)

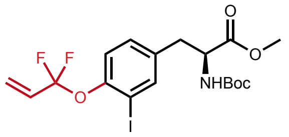

Chemical Formula: $C_{18}H_{22}F_2INO_5$
Exact Mass: 497.0511

This compound was obtained following the general procedure D. Starting from ammonium salt **1a** (117 mg, 0.36 mmol, 1.2 equiv), methyl (*S*)-2-((*tert*-butoxycarbonyl)amino)-3-(4-hydroxy-3-iodophenyl)propanoate **2bc** (126.3 mg, 0.3 mmol), Cs_2CO_3 (49 mg, 0.15 mmol, 0.5 equiv), and Toluene (3.0 mL). Purification on silica gel (Pentane/Ethyl acetate gradient from 90/10 to 70/30) afforded **57** (140 mg, 94%) as a colorless oil.

^{1}H NMR (300 MHz, $CDCl_3$) δ 7.59 (d, *J* = 2.1 Hz, 1H), 7.25 (dt, *J* = 8.4, 1.6 Hz, 1H), 7.09 (dd, *J* = 8.4, 2.1 Hz, 1H), 6.16 – 5.98 (m, 2H), 5.67 – 5.57 (m, 1H), 5.03 (br, 1H), 4.64 – 4.40 (m, 1H), 3.72 (s, 3H), 3.18 – 2.78 (m, 2H), 1.42 (s, 9H).

^{13}C NMR (75 MHz, $CDCl_3$) δ 172.0, 155.1, 149.6, 140.5, 135.5, 130.4, 129.1 (t, *J* = 33.3 Hz), 122.7 (t, *J* = 6.7 Hz), 121.9, 121.04 (t, *J* = 260.3 Hz), 90.7, 80.3, 54.4, 52.5, 37.3, 28.4.

^{19}F NMR (282 MHz, $CDCl_3$) δ -67.6 (d, *J* = 4.0 Hz, 2F).

HRMS (EI): calculated for $C_{18}H_{22}F_2INO_5Na$ $[M+Na]^+$: 520.0408, found: 520.0399.

(8*R*,9*S*,13*S*,14*S*,17*R*)-3-((1,1-difluoroallyl)oxy)-17-ethynyl-13-methyl-7,8,9,11,12,13,14,15,16,17-decahydro-6*H*-cyclopenta[*a*]phenanthren-17-ol (58)

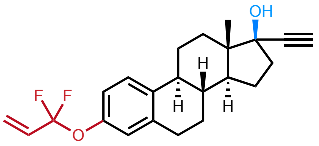

This compound was obtained following the general procedure D. Starting from ammonium salt **1a** (98 mg, 0.3 mmol, 1.0 equiv), Ethylnylestradiol **2bd** (88.9 mg, 0.3 mmol), Cs_2CO_3 (49 mg, 0.15 mmol, 0.5 equiv), and Toluene (3.0 mL). Purification on silica gel (Pentane/Ethyl acetate gradient from 95/5 to 90/10) afforded **58** (104 mg, 93%) as a white solid.

^{1}H NMR (300 MHz, $CDCl_3$) δ 7.31 – 7.21 (m, 1H), 7.02 – 6.95 (m, 1H), 6.94 – 6.89 (m, 1H), 6.14 – 5.98 (m, 1H), 5.96 – 5.85 (m, 1H), 5.58 (dd, *J* = 10.5, 1.0 Hz, 1H), 2.87 (dd, *J* = 8.3, 4.0 Hz, 2H), 2.61 (s, 1H), 2.44 – 2.20 (m, 3H), 2.10 – 1.66 (m, 7H), 1.60 – 1.32 (m, 4H), 0.89 (d, *J* = 0.7 Hz, 3H).

^{13}C NMR (75 MHz, $CDCl_3$) δ 148.1, 138.2, 137.7, 129.7 (t, *J* = 33.9 Hz), 126.4, 122.1, 121.7 (t, *J* = 6.5 Hz), 120.8 (t, *J* = 257.3 Hz), 119.2, 87.6, 80.0, 74.2, 49.7, 47.2, 43.8, 39.2, 39.1, 32.9, 29.7, 27.2, 26.4, 23.0, 12.8.

^{19}F NMR (282 MHz, $CDCl_3$) δ -68.4 (d, *J* = 6.1 Hz, 2F).

HRMS (EI): calculated for $C_{23}H_{26}F_2O_2$ $[M]^+$: 372.1895, found: 372.1888.

(8*R*,9*S*,13*S*,14*S*,17*S*)-3-((1,1-difluoroallyl)oxy)-13-methyl-7,8,9,11,12,13,14,15,16,17-decahydro-6*H*-cyclopenta[*a*]phenanthren-17-ol (59)

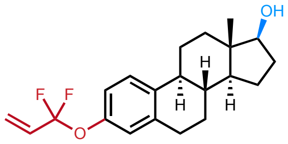

This compound was obtained following the general procedure D. Starting from ammonium salt **1a** (98 mg, 0.3 mmol, 1.0 equiv), *β*-Estradiol **2be** (81.7 mg, 0.3 mmol), Cs_2CO_3 (49 mg, 0.15 mmol, 0.5 equiv), and Toluene (3.0 mL). Purification on silica gel (Pentane/Ethyl acetate gradient from 95/5 to 90/10) afforded **59** (85 mg, 81%) as a white solid. M.p. 90–92 °C.

^{1}H NMR (300 MHz, $CDCl_3$) δ 7.34 – 7.18 (m, 1H), 7.03 – 6.93 (m, 1H), 6.96 – 6.86 (m, 1H), 6.15 – 5.96 (m, 1H), 5.98 – 5.84 (m, 1H), 5.58 (dd, *J* = 10.4, 1.0 Hz, 1H), 3.84 – 3.63 (m, 1H), 2.93 – 2.78 (m, 2H), 2.40 – 2.25 (m, 1H), 2.29 – 2.04 (m, 2H), 2.03 – 1.83 (m, 2H), 1.78 – 1.63 (m, 1H), 1.61 – 1.12 (m, 8H), 0.79 (s, 3H).

^{13}C NMR (75 MHz, $CDCl_3$) δ 148.1 (t, *J* = 1.8 Hz), 138.25, 137.8, 129.70 (t, *J* = 34.0 Hz), 126.4, 122.1, 121.7 (t, *J* = 6.5 Hz), 120.8 (t, *J* = 257.3 Hz), 119.1, 82.0, 50.2, 44.2, 43.3, 38.6, 36.8, 30.7,

29.7, 27.2, 26.3, 23.3, 11.2.

^{19}F NMR (282 MHz, $CDCl_3$) δ -68.4 (d, *J* = 6.3 Hz, 2F).

HRMS (EI): calculated for $C_{21}H_{26}F_2O_2$ $[M]^+$: 348.1895, found: 348.1892.

(3*R*,4*S*)-4-(4-((1,1-difluoroallyl)oxy)phenyl)-1-(4-fluorophenyl)-3-((*S*)-3-(4-fluoro-phenyl)-3-hydroxypropyl)azetidin-2-one (60)

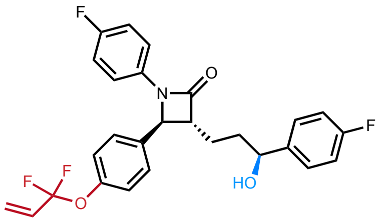

Chemical Formula: $C_{27}H_{23}F_4NO_3$
Exact Mass: 485.1614

This compound was obtained following the general procedure D. Starting from ammonium salt **1a** (65 mg, 0.2 mmol, 1.0 equiv), Ezetimb **2bf** (81.8 mg, 0.2 mmol), Cs_2CO_3 (32.6 mg, 0.1 mmol, 0.5 equiv), and Toluene (2.0 mL). Purification on silica gel (Pentane/Ethyl acetate gradient from 90/10 to 2/1) afforded **60** (81 mg, 84%) as a white solid.

^{1}H NMR (300 MHz, $CDCl_3$) δ 7.36 – 7.16 (m, 8H), 7.09 – 6.87 (m, 4H), 6.16 – 5.96 (m, 1H), 6.00 – 5.86 (m, 1H), 5.61 (dd, *J* = 10.4, 1.0 Hz, 1H), 4.71 (t, *J* = 6.1 Hz, 1H), 4.63 (d, *J* = 2.4 Hz, 1H), 3.09 (td, *J* = 7.4, 2.5 Hz, 1H), 2.35 (br, 1H), 2.08 – 1.81 (m, 3H).

^{13}C NMR (75 MHz, $CDCl_3$) δ 167.5, 162.39 (d, *J* = 238.2 Hz), 159.2 (d, *J* = 236.3 Hz), 150.6, 140.1 (d, *J* = 3.1 Hz), 134.8, 133.8 (d, *J* = 2.7 Hz), 129.3 (t, *J* = 33.4 Hz), 127.5 (d, *J* = 8.1 Hz), 127.0, 122.7, 122.1 (t, *J* = 6.5 Hz), 120.8 (t, *J* = 258.8 Hz), 118.5 (d, *J* = 7.8 Hz), 116.0 (d, *J* = 22.7 Hz), 115.5 (d, *J* = 21.4 Hz), 73.2, 61.0, 60.5, 36.7, 25.2.

^{19}F NMR (282 MHz, $CDCl_3$) δ -68.9 (d, *J* = 6.5 Hz, 2F), -114.8 (tt, *J* = 8.7, 5.4 Hz, 1F), -117.8 (ddd, *J* = 13.1, 8.5, 4.8 Hz, 1F).

HRMS (ESI): calculated for $C_{27}H_{23}F_4NO_3Na$ $[M+Na]^+$: 508.1506, found: 508.1597.

1-(*Tert*-butyl)-4-(3,3-difluoro-3-phenoxyprop-1-en-2-yl)benzene (S1f)

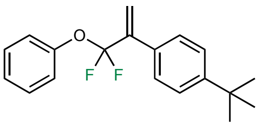

Chemical Formula: $C_{19}H_{20}F_2O$
Exact Mass: 302.1482

This compound was obtained following the general procedure D. Starting from ammonium salt **1f** (457 mg, 1.0 mmol, 1.0 equiv), Phenol (94.1 mg, 1.0 mmol), K_2CO_3 (138 mg, 1.0 mmol, 1.0 equiv), and THF/H_2O (1:5, 6 mL). Purification on silica gel (Pentane/Ethyl acetate gradient from 99/1 to 98/2) afforded **S1f** (296 mg, 98%) as a colorless oil.

^{1}**H NMR** (300 MHz, $CDCl_3$) δ 7.55 – 7.49 (m, 2H), 7.44 – 7.38 (m, 2H), 7.38 – 7.31 (m, 2H), 7.26 – 7.17 (m, 3H), 5.96 (td, *J* = 1.3, 0.4 Hz, 1H), 5.72 (td, *J* = 1.6, 0.4 Hz, 1H), 1.35 (s, 9H).

^{13}**C NMR** (75 MHz, $CDCl_3$) δ 151.7, 150.6, 141.5 (t, *J* = 29.3 Hz), 132.4, 129.4, 127.5, 125.7, 125.4, 122.1, 121.8, 119.0 (t, *J* = 5.9 Hz), 34.7, 31.4.

^{19}**F NMR** (282 MHz, $CDCl_3$) δ -67.4 (s, 2F).

HRMS (EI): calculated for $C_{19}H_{20}F_2O_1$ $[M]^+$: 302.1477, found: 302.1478.

4-((2-(4-(*Tert*-butyl)phenyl)-1,1-difluoroallyl)oxy)-1,1'-biphenyl (S1g)

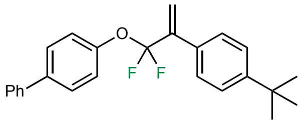

Chemical Formula: $C_{25}H_{24}F_2O$
Exact Mass: 378.1795

This compound was obtained following the general procedure D. Starting from ammonium salt **1f** (914 mg, 2.0 mmol, 1.0 equiv), [1,1'-biphenyl]-4-ol **2a** (340 mg, 2.0 mmol), K_2CO_3 (276 mg, 2.0 mmol, 1.0 equiv), and THF/H_2O (1:5, 6 mL). Purification on silica gel (Pentane/Ethyl acetate gradient from 99/1 to 98/2) afforded **S1g** (700 mg, 93%) as a white solid.

^{1}**H NMR** (300 MHz, $CDCl_3$) δ 7.74 – 7.50 (m, 6H), 7.50 – 7.39 (m, 4H), 7.39 – 7.26 (m, 3H), 5.99 (s, 1H), 5.74 (s, 1H), 1.36 (s, 9H).

^{13}**C NMR** (75 MHz, $CDCl_3$) δ 151.7, 150.0, 141.4, 140.5, 138.8, 132.4, 128.9, 128.2, 127.5, 127.2, 125.4, 122.3, 121.9, 119.3 –118.8 (m), 34.8, 31.4.

^{19}**F NMR** (282 MHz, $CDCl_3$) δ -67.4 (s, 2F).

HRMS (EI): calculated for $C_{25}H_{24}F_2O$ $[M]^+$: 378.1790, found: 378.1786.

(2-([1,1'-Biphenyl]-4-yl)-1,1-difluoroallyl)(4-bromophenyl)sulfane (S1h)

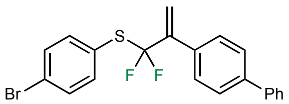

Chemical Formula: $C_{21}H_{15}BrF_2S$
Exact Mass: 416.0046

This compound was obtained following the general procedure C. Starting from ammonium salt **1g** (115 mg, 0.24 mmol, 1.2 equiv), 4-bromobenzenethiol **2d** (38 mg, 0.2 mmol), NaH (60% dispersion in mineral oil, 12 mg, 0.3 mmol, 1.5 equiv), and DMF (1.0 mL). Purification on silica gel (Pentane/Ethyl acetate gradient from 99/1 to 90/10) afforded **S1h** (82 mg, 99%) as a white solid.

^{1}**H NMR** (300 MHz, $CDCl_3$) δ 7.68 – 7.56 (m, 6H), 7.55 – 7.42 (m, 6H), 7.42 – 7.34 (m, 1H), 5.74 (t, *J* = 1.4 Hz, 1H), 5.62 (t, *J* = 1.3 Hz, 1H).

^{13}**C NMR** (75 MHz, $CDCl_3$) δ 143.4, 141.6, 140.5, 137.9, 134.5, 132.4, 129.0, 128.8, 127.7, 127.2,

127.12, 126.5, 125.0, 119.9 (t, J = 6.8 Hz), 100.1.

^{19}F NMR (282 MHz, $CDCl_3$) δ -71.1 (s, 2F).

HRMS (EI): calculated for $C_{21}H_{15}BrF_2S$ $[M]^+$: 416.0040, found: 416.0041.

(4-(3,3-Difluoro-3-(phenylselanyl)prop-1-en-2-yl)phenyl)trimethylsilane (S1i)

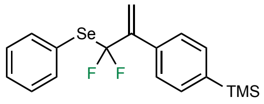

Chemical Formula: $C_{18}H_{20}F_2SeSi$
Exact Mass: 382.0468

This compound was obtained following the general procedure C. Starting from ammonium salt **1h** (114 mg, 0.24 mmol, 1.2 equiv), benzeneselenol **2ad** (32 mg, 0.2 mmol), NaH (60% dispersion in mineral oil, 12 mg, 0.3 mmol, 1.5 equiv), and DMF (1.0 mL). Purification on silica gel (Pentane/Ethyl acetate gradient from 99/1 to 95/5) afforded **S1i** (76 mg, 99%) as a slight yellow oil.

^{1}H NMR (300 MHz, $CDCl_3$) δ 7.60 – 7.52 (m, 2H), 7.45 – 7.35 (m, 4H), 7.34 – 7.20 (m, 3H), 5.48 – 5.41 (m, 1H), 5.36 – 5.29 (m, 1H), 0.20 (s, 9H).

^{13}C NMR (75 MHz, $CDCl_3$) δ 144.7 (t, J = 20.3 Hz), 141.2, 137.3, 133.4, 129.6, 129.2, 127.7, 125.7, 123.6, 118.7 (t, J = 7.4 Hz), -1.0.

^{19}F NMR (282 MHz, $CDCl_3$) δ -69.6 (s, 2F).

HRMS (EI): calculated for $C_{18}H_{20}F_2SeSi$ $[M]^+$: 382.0462, found: 382.0452.

1-(2-(4-Chlorophenyl)-1,1-difluoroallyl)-1*H*-benzo[*d*]imidazole (S1j)

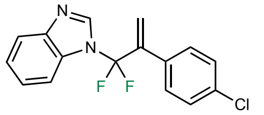

Chemical Formula: $C_{16}H_{11}ClF_2N_2$
Exact Mass: 304.0579

This compound was obtained following the general procedure C. Starting from ammonium salt **1i** (104.4 mg, 0.24 mmol, 1.2 equiv), 1*H*-benzo[*d*]imidazole **2ah** (23.6 mg, 0.2 mmol), NaH (60% dispersion in mineral oil, 12 mg, 0.3 mmol, 1.5 equiv), and DMF (1.0 mL). Purification on silica gel (Pentane/Ethyl acetate gradient from 5/1 to 1/1) afforded **S1j** (60 mg, 99%) as a colorless oil.

^{1}H NMR (300 MHz, $CDCl_3$*d*) δ 7.98 (s, 1H), 7.86 – 7.79 (m, 1H), 7.68 – 7.60 (m, 1H), 7.42 – 7.34 (m, 2H), 7.32 – 7.21 (m, 4H), 6.01 (t, J = 1.6 Hz, 1H), 5.96 (t, J = 1.2 Hz, 1H).

^{13}C NMR (75 MHz, $CDCl_3$) δ 144.0, 140.0 (d, J = 55.4 Hz), 139.9, 135.5, 132.5, 129.1, 129.0, 124.8, 124.0, 122.9 (t, J = 6.6 Hz), 121.0, 117.4, 112.5 (t, J = 2.5 Hz).

^{19}F NMR (282 MHz, $CDCl_3$) δ -75.5 (s, 2F).

HRMS (EI): calculated for $C_{16}H_{11}F_2N_2Cl$ $[M]^+$: 305.0657, found: 305.0654.

Butyl 4-([1,1'-biphenyl]-4-yloxy)-4,4-difluorobutanoate (61)

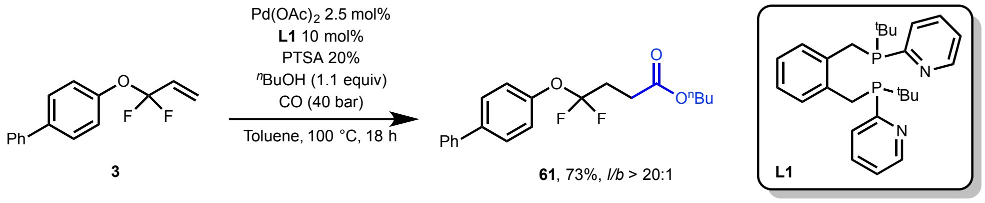

A 8 mL screw-cap vial was charged with $Pd(OAc)_2$ (2.5 mol%, 1.1 mg), ligand **L1** (10 mol%, 8.7 mg), PTSA•H_2O (20 mol%, 7.6 mg), compound **3** (0.2 mmol, 49.2 mg) and an oven-dried stirring bar. The vial was closed by PTFE/white rubber septum (Wheaton 13 mm Septa) and phenolic cap and connected with atmosphere with a needle. Then, the vial was under vacuum and recharged with argon for three times. After toluene (2.0 ml) and butanol (0.22 mmol, 20 μL) were injected by syringe; the vial was fixed in an alloy plate and put into Paar 4560 series autoclave (300 mL) under argon atmosphere. At room temperature, the autoclave is flushed with carbon monoxide for three times and carbon monoxide was charged to 40 *bar*. The reaction was heated under 100 °C for 18 hours. Afterwards, the autoclave was cooled to room temperature and the pressure was carefully released. After evaporation of the solvent, the residue was purified by chromatography on silica gel (Pentane/Ethyl acetate gradient from 95/5 to 90/10) to afford the desired product **61** as a white solid (51 mg, yield 73%, *l*/*b* > 20:1). M.p. 52–54 °C.

^{1}H NMR (300 MHz, $CDCl_3$) δ 7.61 – 7.51 (m, 4H), 7.47 – 7.39 (m, 2H), 7.38 – 7.31 (m, 1H), 7.27 – 7.20 (m, 2H), 4.14 (t, *J* = 6.7 Hz, 2H), 2.74 – 2.64 (m, 2H), 2.64 – 2.46 (m, 2H), 1.71 – 1.58 (m, 2H), 1.47 – 1.30 (m, 2H), 0.95 (t, *J* = 7.3 Hz, 3H).

^{13}C NMR (75 MHz, $CDCl_3$) δ 172.0, 140.4, 138.8, 128.9, 128.2, 127.5, 127.2, 124.6, 122.0, 64.9, 31.6 (t, *J* = 30.0 Hz), 30.8, 28.2, 19.3, 13.8.

^{19}F NMR (282 MHz, $CDCl_3$) δ -71.2 (t, *J* = 10.5 Hz, 2F).

HRMS (EI): calculated for $C_{20}H_{22}F_2O_3$ $[M]^+$: 348.1532, found: 348.1540.

4-([1,1'-Biphenyl]-4-yloxy)-4,4-difluorobutanal (62)

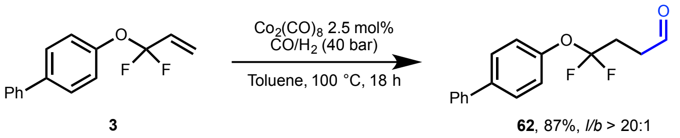

A 8 mL screw-cap vial was charged with $Co_2(CO)_8$ (2.5 mol%, 1.7 mg), compound **3** (0.1 mmol, 24.6 mg) and an oven-dried stirring bar. The vial was closed by PTFE/white rubber septum

(Wheaton 13 mm Septa) and phenolic cap and connected with atmosphere with a needle. Then, the vial was under vacuum and recharged with argon for three times. After toluene (2.0 ml) was injected by syringe; the vial was fixed in an alloy plate and put into Paar 4560 series autoclave (300 mL) under argon atmosphere. At room temperature, the autoclave is flushed with syngas for three times and syngas was charged to 40 *bar*. The reaction was heated under 100 °C for 18 hours. Afterwards, the autoclave was cooled to room temperature and the pressure was carefully released. After evaporation of the solvent, the residue was purified by chromatography on silica gel (Pentane/Ethyl acetate gradient from 95/5 to 80/20) to afford the desired product **62** as a white solid (24 mg, yield 87%, *l*/*b* > 20:1). M.p. 66–67 °C.

^{1}H NMR (300 MHz, $CDCl_3$) δ 9.86 (t, *J* = 1.0 Hz, 1H), 7.60 – 7.53 (m, 4H), 7.48 – 7.41 (m, 2H), 7.39 – 7.32 (m, 1H), 7.26 – 7.19 (m, 2H), 2.85 (t, *J* = 7.5 Hz, 2H), 2.64 – 2.48 (m, 2H).

^{13}C NMR (75 MHz, $CDCl_3$) δ 199.4, 149.7, 140.4, 138.9, 129.0, 128.2, 127.5, 127.2, 124.7 (t, *J* = 264.0 Hz), 122.0, 37.5 (t, *J* = 2.7 Hz), 28.9 (t, *J* = 30.6 Hz).

^{19}F NMR (282 MHz, $CDCl_3$) δ -70.9 (t, *J* = 10.9 Hz, 2F).

HRMS (EI): calculated for $C_{16}H_{14}F_2O_2$ $[M]^+$: 276.0956, found: 276.0957.

3,3-Difluoro-3-(naphthalen-1-yloxy)propane-1,2-diol (63)

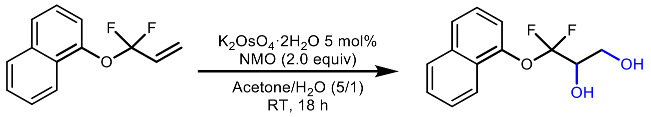

16 — **63**, 99%

To a 25 mL oven-dried pressure tube equipped with a magnetic stir bar were added compound **16** (210 mg, 0.95 mmol), K_2OsO_4•H_2O (5 mol%, 17.5 mg), 4-Methylmorpholine *N*-oxide (NMO, 222 mg, 1.9 mmol), and then Acetone (5 mL) and H_2O (1 mL) were introduced under argon atmosphere. The sealed pressure tube was vigorously stirred at 23 °C for 18 hours. The mixture was diluted with EtOAc (20 mL) and washed with $NaHCO_3$ (10 mL) and brine (10 mL). The combined organic layer was dried over anhydrous Na_2SO_4, filtered and concentrated. the residue was purified by chromatography on silica gel (Pentane/Ethyl acetate gradient from 4/1 to 2/1) to afford the desired product **63** as a white solid (238 mg, yield 99%). M.p. 77–78 °C.

^{1}H NMR (300 MHz, $CDCl_3$) δ 8.15 – 8.05 (m, 1H), 7.90 – 7.79 (m, 1H), 7.77 – 7.67 (m, 1H), 7.57 – 7.45 (m, 2H), 7.45 – 7.34 (m, 2H), 4.39 – 4.27 (m, 1H), 4.11 (d, *J* = 5.1 Hz, 2H), 2.98 (br, 2H, OH).

^{13}C NMR (75 MHz, $CDCl_3$) δ 145.5, 134.8, 127.9, 126.7, 126.6, 126.1, 125.4, 122.9, 121.9, 117.8, 72.5 (t, *J* = 30.0 Hz), 61.5.

^{19}**F NMR** (282 MHz, $CDCl_3$) δ -80.0 – -81.8 (m).

HRMS (EI): calculated for $C_{13}H_{12}F_2O_3$ $[M]^+$: 254.0749, found: 254.0749.

3,3-Difluoro-3-(2-(naphthalen-1-yl)ethoxy)propane-1,2-diol (64)

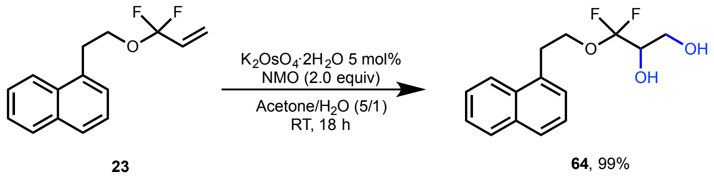

To a 25 mL oven-dried pressure tube equipped with a magnetic stir bar were added compound **23** (124 mg, 0.5 mmol), K_2OsO_4•H_2O (5 mol%, 9.2 mg), 4-Methylmorpholine *N*-oxide (NMO, 117.2 mg, 1.0 mmol), and then Acetone (2.5 mL) and H_2O (0.5 mL) were introduced under argon atmosphere. The sealed pressure tube was vigorously stirred at 23 °C for 18 hours. The mixture was diluted with EtOAc (20 mL) and washed with $NaHCO_3$ (10 mL) and brine (10 mL). The combined organic layer was dried over anhydrous Na_2SO_4, filtered and concentrated. the residue was purified by chromatography on silica gel (Pentane/Ethyl acetate gradient from 2/1 to 1/1) to afford the desired product **64** as a white solid (140 mg, yield 99%). M.p. 68–71 °C.

^{1}**H NMR** (300 MHz, $CDCl_3$) δ 8.07 – 7.97 (m, 1H), 7.91 – 7.84 (m, 1H), 7.81 – 7.73 (m, 1H), 7.60 – 7.32 (m, 4H), 4.27 (t, *J* = 7.2 Hz, 2H), 3.97 – 3.83 (m, 1H), 3.73 (d, *J* = 4.9 Hz, 2H), 3.44 (t, *J* = 7.2 Hz, 2H), 3.03 – 2.60 (br, 1H, OH), 2.03 – 1.71 (br, 1H, OH).

^{13}**C NMR** (75 MHz, $CDCl_3$) δ 134.0, 133.4, 132.0, 129.1, 127.8, 127.1, 126.4, 125.9, 125.6, 123.4, 71.9 (dd, *J* = 32.3, 30 Hz), 63.8 (t, *J* = 6.2 Hz), 61.3 (t, *J* = 2.3 Hz).

^{19}**F NMR** (282 MHz, $CDCl_3$) δ -83.4 – -85.6 (m, 2F).

HRMS (EI): calculated for $C_{15}H_{16}F_2O_2$ $[M]^+$: 282.1062, found: 282.1064.

3,3-Difluoro-7-tosyl-3,3a,4,5,5a,6,7,8-octahydro-1H-furo[3,4-e]isoindole (65)

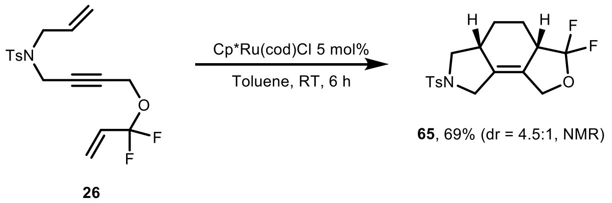

To a 25 mL oven-dried round-bottom flask equipped with a magnetic stir bar were added compound **26** (106.5 mg, 0.3 mmol), Cp*RuCl(cod) (5.7 mg, 0.015 mmol), and dry toluene (10 mL). The reaction mixture was stirred at room temperature under an atmosphere of argon until the spot of the starting material on TLC was disappeared (20 hours). After completion of the reaction, the solvent was removed under reduced pressure, and the residue was purified by column chromatography on silica gel to give a cycloaddition product **65** as a colorless oil (73

mg, 69%, dr = 4.5/1).

^{1}H NMR (300 MHz, $CDCl_3$) δ 7.73 – 7.66 (m, 2H), 7.38 – 7.30 (m, 2H), 4.65 – 4.52 (m, 1H), 4.47 – 4.36 (m, 1H), 3.95 – 3.85 (m, 1H), 3.85 – 3.77 (m, 1H), 3.69 – 3.57 (m, 1H), 2.88 – 2.68 (m, 1H), 2.59 – 2.45 (m, 2H), 2.45 (s, 3H), 1.88 – 1.69 (m, 3H), 1.13 – 0.99 (m, 1H).

^{13}C NMR (75 MHz, $CDCl_3$) δ 144.2, 133.4, 133.0, 130.0, 127.8, 125.8, 69.0, 53.0, 49.0, 41.6 (dd, J = 30.5=8, 28.5 Hz), 37.5, 23.6, 21.7, 18.8.

^{19}F NMR (282 MHz, $CDCl_3$) δ -73.6 (ddd, *J* = 139.5, 16.1, 5.9 Hz, 1F), -79.5 (ddd, *J* = 140.1, 8.5, 3.2 Hz, 1F).

HRMS (EI): calculated for $C_{17}H_{19}F_2NO_3S$ $[M]^+$: 378.0951, found: 378.0952.

(*Z*)-4-benzylidene-2,2-difluoro-3-methyltetrahydrofuran (66)

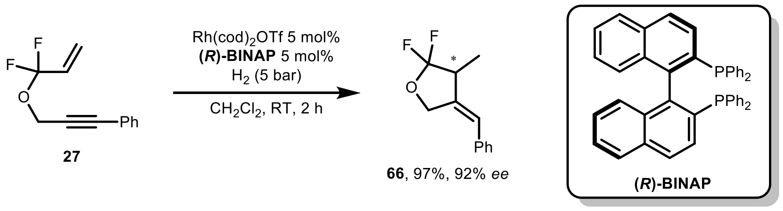

A 8 mL screw-cap vial was charged with $Rh(cod)_2OTf$ (5 mol%, 11.7 mg), (*R*)-BINAP (5 mol%, 15.6 mg), compound **27** (104.1 mg, 0.2 mmol) and an oven-dried stirring bar. The vial was closed by PTFE/white rubber septum (Wheaton 13 mm Septa) and phenolic cap and connected with atmosphere with a needle. Then, the vial was under vacuum and recharged with argon for three times. After dichloromethane (5.0 ml) was injected by syringe; the vial was fixed in an alloy plate and put into Paar 4560 series autoclave (300 mL) under argon atmosphere. At room temperature, the autoclave is flushed with hydrogen gas for three times and hydrogen gas was charged to 5 *bar*. The reaction was heated at room temperature for 2 hours. Afterwards, the pressure was carefully released. After evaporation of the solvent, the residue was purified by chromatography on silica gel (pure pentane) to afford the desired product **66** as a colorless oil (102 mg, yield 97%). The enantiomeric excess (92%) of the product was determined by chiral HPLC.

HPLC conditions: chiralcel AD-H, heptane/ethanol = 99/1, 0.5 mL/min, 210 nm). Major enantiomer: t1 = 7.13 min; minor enantiomer: t2 = 8.91 min.

^{1}H NMR (300 MHz, $CDCl_3$) δ 7.42 – 7.33 (m, 2H), 7.31 – 7.24 (m, 1H), 7.14 – 7.08 (m, 2H), 6.37 (q, *J* = 2.6 Hz, 1H), 5.09 – 4.89 (m, 2H), 3.36 – 3.07 (m, 1H), 1.36 (dd, *J* = 7.0, 1.1 Hz, 3H).

^{13}C NMR (75 MHz, $CDCl_3$) δ 137.9 (d, *J* = 4.1 Hz), 135.8, 128.8, 128.3, 127.6, 123.3, 70.8, 44.2 (t, *J* = 29.6 Hz), 12.1 (d, *J* = 4.5 Hz).

^{19}F NMR (282 MHz, $CDCl_3$) δ -76.6 (ddd, *J* = 140.4, 8.6, 3.0 Hz, 1F), -78.7 (ddd, *J* = 140.3, 11.2, 3.8 Hz, 1F).

HRMS (EI): calculated for $C_{12}H_{12}F_2O$ $[M]^+$: 210.0851, found: 210.0846.

Synthesis of compound 70

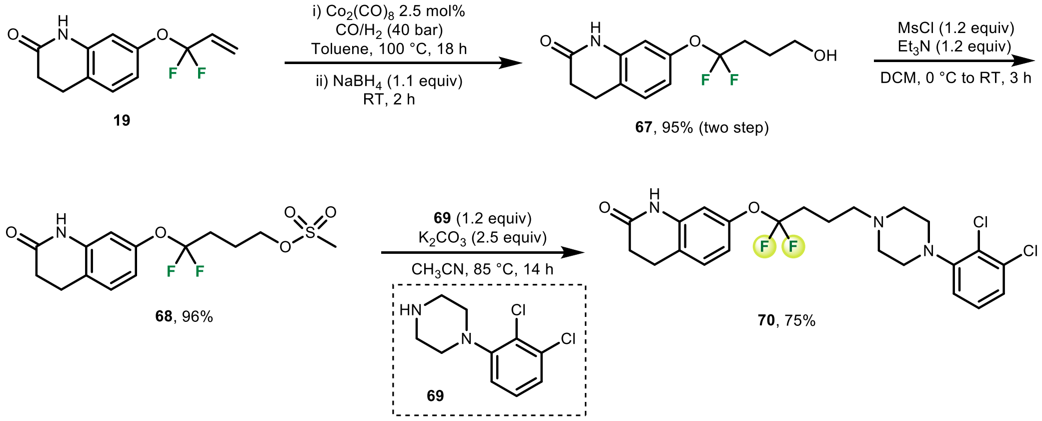

7-(1,1-Difluoro-4-hydroxybutoxy)-3,4-dihydroquinolin-2(1H)-one (67)

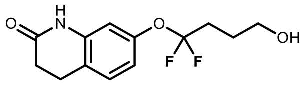

Chemical Formula: $C_{13}H_{15}F_2NO_3$
Exact Mass: 271.1020

A 25 mL screw-cap vial was charged with $Co_2(CO)_8$ (2.5 mol%, 42.8 mg), compound **19** (2.5 mmol, 597 mg) and an oven-dried stirring bar. The vial was closed by PTFE/white rubber septum (Wheaton 29 mm Septa) and phenolic cap and connected with atmosphere with a needle. Then, the vial was under vacuum and recharged with argon for three times. After toluene (20.0 ml) was injected by syringe; the vial was fixed in an alloy plate and put into Paar 4560 series autoclave (300 mL) under argon atmosphere. At room temperature, the autoclave is flushed with syngas for three times and syngas was charged to 40 *bar*. The reaction was heated under 100 °C for 18 hours. Afterwards, the autoclave was cooled to room temperature and the pressure was carefully released. After evaporation of the solvent, the product was used directly for the next step without further purification. The l/b selectivity (l/b > 20:1) was determined by ^{19}F NMR using 1,4-difluorobenzen as internal standard.

To the above obtained product, 1.1 equivalent of $NaBH_4$ and MeOH (10 mL) were introduced. The reaction mixture was stirred at 0 °C for 1 hour before quench with 10 mL water. The resulting mixture was extracted with ethyl acetate (3 x 20 mL) and washed with $NaHCO_3$ (10 mL) and brine (10 mL). The combined organic layer was dried over anhydrous Na_2SO_4, filtered and concentrated. The residue was purified by chromatography on silica gel (Pentane/Ethyl acetate gradient from 90/10 to 2/1) to afford the desired product **67** as a white solid (643 mg,

yield 95%).

^{1}H NMR (300 MHz, $CDCl_3$) δ 8.54 (s, 1H), 7.10 (d, *J* = 8.2 Hz, 1H), 6.85 – 6.74 (m, 1H), 6.64 (d, *J* = 2.1 Hz, 1H), 3.76 (dd, *J* = 6.6, 5.9 Hz, 2H), 2.94 (dd, *J* = 8.5, 6.3 Hz, 2H), 2.71 – 2.52 (m, 2H), 2.36 – 2.14 (m, 2H), 1.98 – 1.83 (m, 2H).

^{13}C NMR (75 MHz, $CDCl_3$) δ 171.9, 149.8 (t, *J* = 2.3 Hz), 138.1, 128.7, 125.3 (t, *J* = 264.0 Hz), 120.9, 116.2, 109.3, 61.7, 32.5 (t, *J* = 29.2 Hz), 30.8, 25.9, 24.9.

^{19}F NMR (282 MHz, $CDCl_3$) δ -70.4 (t, *J* = 11.3 Hz, 2F).

HRMS (ESI): calculated for $C_{13}H_{16}F_2NO_3$ $[M+H]^+$: 272.1098, found: 272.1100.

4,4-Difluoro-4-((2-oxo-1,2,3,4-tetrahydroquinolin-7-yl)oxy)butyl methanesulfonate (68)

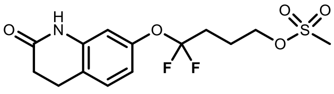

Chemical Formula: $C_{14}H_{17}F_2NO_5S$
Exact Mass: 349.0795

To a 25 mL oven-dried round-bottom flask equipped with a magnetic stir bar were added compound **67** (643 mg, 2.37 mmol), triethylamine (395 μL, 2.84 mmol) and dry dichloromethane (10 mL). Then methanesulfonyl chloride (220 μL, 2.84 mmol) was added dropwise under argon atmosphere at 0 °C in an ice-bath. The resulting mixture was vigorously stirred at 0 °C for 3 hours. The reaction mixture was extracted with DCM (20 mL) and washed with $NaHCO_3$ (10 mL) and brine (10 mL). The combined organic layer was dried over anhydrous Na_2SO_4, filtered and concentrated. the residue was purified by chromatography on silica gel (Pentane/Ethyl acetate gradient from 90/10 to 2/1) to afford the desired product **68** as a white solid (790 mg, yield 96%).

^{1}H NMR (300 MHz, $CDCl_3$) δ 8.74 (s, 1H, N-H), 7.11 (d, *J* = 8.3 Hz, 1H), 6.87 – 6.74 (m, 1H), 6.64 (d, *J* = 2.3 Hz, 1H), 4.34 (t, *J* = 6.2 Hz, 2H), 3.04 (s, 3H), 2.94 (dd, *J* = 8.6, 6.5 Hz, 2H), 2.64 (dd, *J* = 8.5, 6.4 Hz, 2H), 2.38 – 2.20 (m, 2H), 2.18 – 2.01 (m, 2H).

^{13}C NMR (75 MHz, $CDCl_3$) δ 171.9, 149.5, 138.3, 128.7, 124.7, 121.1, 116.1, 109.2, 68.5, 37.6, 32.2 (t, *J* = 29.7 Hz), 30.8, 24.9, 23.0 (t, *J* = 3.0 Hz).

^{19}F NMR (282 MHz, $CDCl_3$) δ -70.3 (t, *J* = 10.8 Hz, 2F).

7-(4-(4-(2,3-Dichlorophenyl)piperazin-1-yl)-1,1-difluorobutoxy)-3,4-dihydroquinolin-2(1H)-one (70)

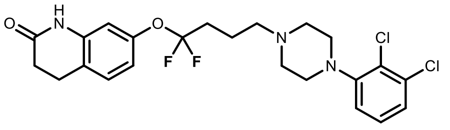

Chemical Formula: $C_{23}H_{25}Cl_2F_2N_3O_2$
Exact Mass: 483.1292

To a 25 mL oven-dried round-bottom flask equipped with a magnetic stir bar were added compound **68** (349 mg, 1.0 mmol) and K_2CO_3 (345 mg, 2.5 mmol), and dry acetonitrile (10 mL). A solution of 1-(2,3-dichlorophenyl)piperazine **69** (276 mg, 1.2 mmol) in acetonitrile (5 mL) was added dropwise under argon atmosphere. The resulting mixture was vigorously stirred at 85 °C for 14 hours. A suspension is produced during the reaction, after cooling down to room temperature, a white solid is obtained by filtration. Dissolve the white solid in water and extracted with dichloromethane (3 x 20 mL). The combined organic layer was dried over anhydrous Na_2SO_4, filtered and concentrated to obtain the target product **70** as a white solid (365 mg, 75%). M.p. 193–194 °C.

^{1}H NMR (300 MHz, $CDCl_3$) δ 8.32 (s, 1H), 7.22 – 7.03 (m, 3H), 6.96 (dd, *J* = 6.3, 3.4 Hz, 1H), 6.81 (dd, *J* = 8.2, 2.3 Hz, 1H), 6.70 – 6.54 (m, 1H), 3.24 – 3.01 (m, 4H), 3.00 – 2.89 (m, 2H), 2.66 (dt, *J* = 15.0, 6.3 Hz, 6H), 2.53 (t, *J* = 7.4 Hz, 2H), 2.21 (dt, *J* = 16.3, 11.0 Hz, 2H), 1.88 (dt, *J* = 15.1, 7.4 Hz, 2H).

^{13}C NMR (101 MHz, $CDCl_3$) δ 171.6, 151.3, 149.8, 138.2, 134.2, 128.7, 127.7, 127.6, 124.8, 120.9, 118.8, 116.2, 109.2, 57.4, 53.3, 51.3, 33.8 (t, *J* = 21.8 Hz), 30.82, 24.96, 20.08.

^{19}F NMR (282 MHz, $CDCl_3$) δ -70.5 (t, *J* = 11.2 Hz, 2F).

HRMS (EI): calculated for $C_{23}H_{25}Cl_2F_2N_3O_2$ $[M]^+$: 483.1286, found: 483.1279.

Synthesis of pramocaine analogue 73.

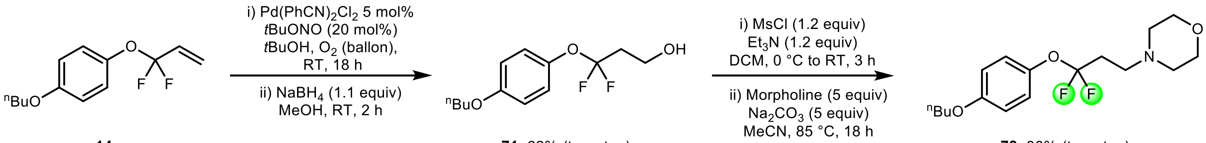

3-(4-Butoxyphenoxy)-3,3-difluoropropan-1-ol (71)

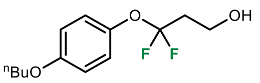

Chemical Formula: $C_{13}H_{18}F_2O_3$
Exact Mass: 260.1224

$Pd(PhCN)_2Cl_2$ (9.6 mg, 0.025 mmol) was weighed directly into a 25 mL oven-dried Schlenk tube, purge oxygen 3 times. Under an atmosphere of oxygen (1 atm, balloon) *t*BuOH (8 mL) and *t*BuONO (10.3 mg, 0.1 mmol) were added and stirred at 25 °C. Alkene **14** (121 mg, 0.5 mmol) was then added and the resulting reaction mixture was monitored by TLC. After completion, the reaction was quenched by addition of water (5 mL) and extracted three times with CH_2Cl_2. The combined organic layers were subsequently washed with brine and dried over Na_2SO_4. After evaporation of the solvent, the product was directly used for the next step without

further purification. The l/b selectivity (l/b = 15:1) was determined by ^{19}F NMR using 1,4-difluorobenzen as internal standard.

To the above obtained product, 1.1 equivalent of $NaBH_4$ and MeOH (2 mL) were introduced. The reaction mixture was stirred at 0 °C for 1 hour before quench with 10 mL water. The resulting mixture was extracted with ethyl acetate (3 x 20 mL) and washed with $NaHCO_3$ (10 mL) and brine (10 mL). The combined organic layer was dried over anhydrous Na_2SO_4, filtered and concentrated. The residue was purified by chromatography on silica gel (Pentane/Ethyl acetate gradient from 90/10 to 70/30) to afford the desired product **71** as a white solid (80 mg, yield 62%).

^{1}H NMR (300 MHz, $CDCl_3$) δ 7.12 – 7.04 (m, 2H), 6.89 – 6.79 (m, 2H), 3.96 (t, *J* = 6.0 Hz, 2H), 3.93 (t, *J* = 6.5 Hz, 2H)2.43 (tt, *J* = 10.9, 6.1 Hz, 2H), 1.95 (s, 1H), 1.81 – 1.70 (m, 2H), 1.56 – 1.41 (m, 2H), 0.97 (t, *J* = 7.4 Hz, 3H).

^{13}C NMR (75 MHz, $CDCl_3$) δ 157.1, 143.2, 124.5(t, *J* = 263.3 Hz), 123.3, 115.0, 68.2, 57.3, 38.8 (t, J = 27.6 Hz), 31.4, 19.4, 14.0.

^{19}F NMR (282 MHz, $CDCl_3$) δ -68.8 (t, *J* = 10.9 Hz, 2F).

HRMS (EI): calculated for $C_{13}H_{18}F_2O_3$ $[M]^+$: 260.1219, found: 260.1222.

3-(4-Butoxyphenoxy)-3,3-difluoropropyl methanesulfonate (72)

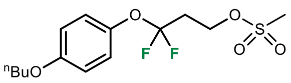

Chemical Formula: $C_{14}H_{20}F_2O_5S$
Exact Mass: 338.1000

To a 25 mL oven-dried round-bottom flask equipped with a magnetic stir bar were added compound **71** (65 mg, 0.25 mmol), triethylamine (42 μL, 0.3 mmol) and dry dichloromethane (2 mL). Then methanesulfonyl chloride (23 μL, 0.3 mmol) was added dropwise under argon atmosphere at 0 °C in an ice-bath. The resulting mixture was vigorously stirred at 0 °C for 3 hours. The reaction mixture was extracted with DCM (20 mL) and washed with $NaHCO_3$ (10 mL) and brine (10 mL). The combined organic layer was dried over anhydrous Na_2SO_4, filtered and concentrated. the residue was purified by chromatography on silica gel (Pentane/Ethyl acetate gradient from 90/10 to 0/1) to afford the desired product **72** as a colorless oil (85 mg, yield 100%).

^{1}H NMR (300 MHz, $CDCl_3$) δ 7.12 – 7.01 (m, 2H), 6.90 – 6.78 (m, 2H), 4.52 (t, *J* = 6.6 Hz, 2H), 3.93 (t, *J* = 6.5 Hz, 2H), 3.05 (s, 3H), 2.63 (tt, *J* = 10.2, 6.6 Hz, 2H), 1.83 – 1.68 (m, 2H), 1.58 – 1.39 (m, 2H), 0.97 (t, *J* = 7.4 Hz, 3H).

^{13}C NMR (75 MHz, $CDCl_3$) δ 157.2, 143.0, 123.2, 123.0 (t, *J* = 263.3 Hz), 115.1, 68.2, 63.4 (t, *J* = 3.8 Hz), 37.7, 35.9 (t, *J* = 30.3 Hz), 31.4, 19.4, 14.0.

^{19}F NMR (282 MHz, $CDCl_3$) δ -69.01 (t, *J* = 10.2 Hz, 2F).

HRMS (EI): calculated for $C_{14}H_{20}F_2O_5S$ $[M]^+$: 338.0994, found: 338.0992.

4-(3-(4-Butoxyphenoxy)-3,3-difluoropropyl)morpholine (73)

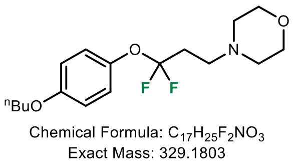

To a 25 mL oven-dried round-bottom flask equipped with a magnetic stir bar were added compound **73** (60 mg, 0.18 mmol) and Na_2CO_3 (94 mg, 0.89 mmol), and dry acetonitrile (5 mL). A solution of morpholine (77 mg, 0.89 mmol) in acetonitrile (1 mL) was added dropwise under argon atmosphere. The resulting mixture was vigorously stirred at 85 °C for 14 hours. The reaction mixture was extracted with ethyl acetate (20 mL) and washed with water (10 mL) and brine (10 mL). The combined organic layer was dried over anhydrous Na_2SO_4, filtered and concentrated. the residue was purified by chromatography on silica gel (Pentane/Ethyl acetate gradient from 90/10 to 0/1) to afford the desired product **73** as a colorless oil (50 mg, yield 86%).

^{1}H NMR (300 MHz, $CDCl_3$) δ 7.11 – 7.00 (m, 2H), 6.89 – 6.75 (m, 2H), 3.92 (t, *J* = 6.5 Hz, 2H), 3.79 – 3.68 (m, 4H), 2.75 – 2.64 (m, 2H), 2.57 – 2.43 (m, 4H), 2.44 – 2.25 (m, 2H), 1.83 – 1.67 (m, 2H), 1.57 – 1.39 (m, 2H), 0.97 (t, *J* = 7.4 Hz, 3H).

^{13}C NMR (75 MHz, $CDCl_3$) δ 157.0, 143.5, 124.5 (t, *J* = 262.5 Hz), 123.2, 115.0, 68.2, 67.0, 53.7, 52.3 (t, *J* = 3.0 Hz), 33.4 (t, *J* = 28.5 Hz), 31.4, 19.4, 14.0.

^{19}F NMR (282 MHz, $CDCl_3$) δ -69.7 (t, *J* = 10.8 Hz, 2F).

HRMS (EI): calculated for $C_{17}H_{25}F_2NO_3$ $[M]^+$: 329.1797, found: 329.1797.

4. X-ray crystal structure analysis of compound 44 and 70

Data were collected on a Bruker Kappa APEX II Duo diffractometer. The structures were solved by direct methods (SHELXS-97: Sheldrick, G. M. *Acta Cryst.* **2008**, *A64*, 112.) and refined by full-matrix least-squares procedures on F^2 (SHELXL-2018: Sheldrick, G. M. *Acta Cryst.* **2015**, *C71*, 3.). XP (Bruker AXS) was used for graphical representations.

CCDC 2035829 (**44**) and 2035828 (**70**) contain the supplementary crystallographic data for this paper. These data are provided free of charge by the joint Cambridge Crystallographic Data Centre and Fachinformationszentrum Karlsruhe Access Structures service www.ccdc.cam.ac.uk/structures.

Crystal data of **44**: $C_{21}H_{24}F_2O_2$, M = 346.40, triclinic, space group $P1$, a = 6.7111(4), b = 7.8579(5), c = 8.9607(6) Å, α = 92.668(3), β = 111.349(3), γ = 92.915(3)°, V = 438.47(5) Å^3, T = 150(2) K, Z = 1, 7401 reflections measured, 2972 independent reflections (R_{int} = 0.0171), final R values ($I > 2\sigma(I)$): R_1 = 0.0373, wR_2 = 0.0973, final R values (all data): R_1 = 0.0378, wR_2 = 0.0983, 228 parameters, Flack parameter x = 0.01(11).

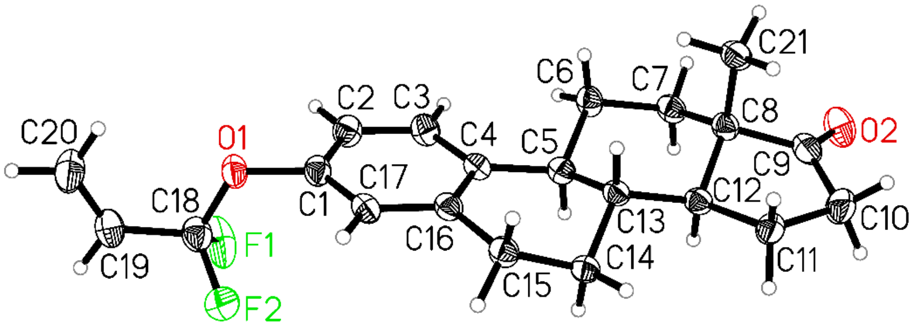

Supplementary Figure 4. Molecular structure of 44. Displacement ellipsoids correspond to 50% probability.

Crystal data of **70**: $C_{23}H_{25}Cl_2F_2N_3O_2$, M = 484.36, monoclinic, space group $P2_1$, a = 8.6081(10), b = 7.6103(8), c = 17.230(2) Å, β = 96.149(2)°, V = 1122.2(2) Å^3, T = 150(2) K, Z = 2, 16540 reflections measured, 5985 independent reflections (R_{int} = 0.025), final R values ($I > 2\sigma(I)$): R_1 = 0.0316, wR_2 = 0.0765, final R values (all data): R_1 = 0.0345, wR_2 = 0.0790, 294 parameters, Flack parameter x = 0.48(5).

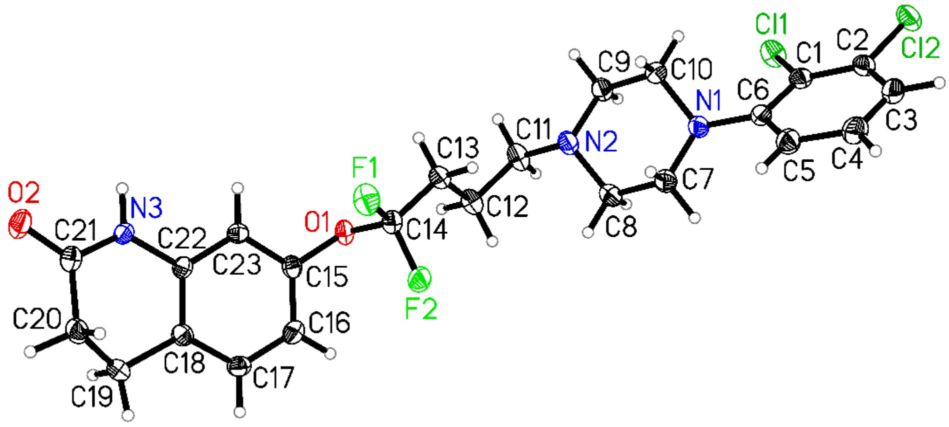

Supplementary Figure 5. Molecular structure of 70. Displacement ellipsoids correspond to 50% probability.

5. ^{1}H, ^{13}C, ^{19}F NMR spectra for new compounds

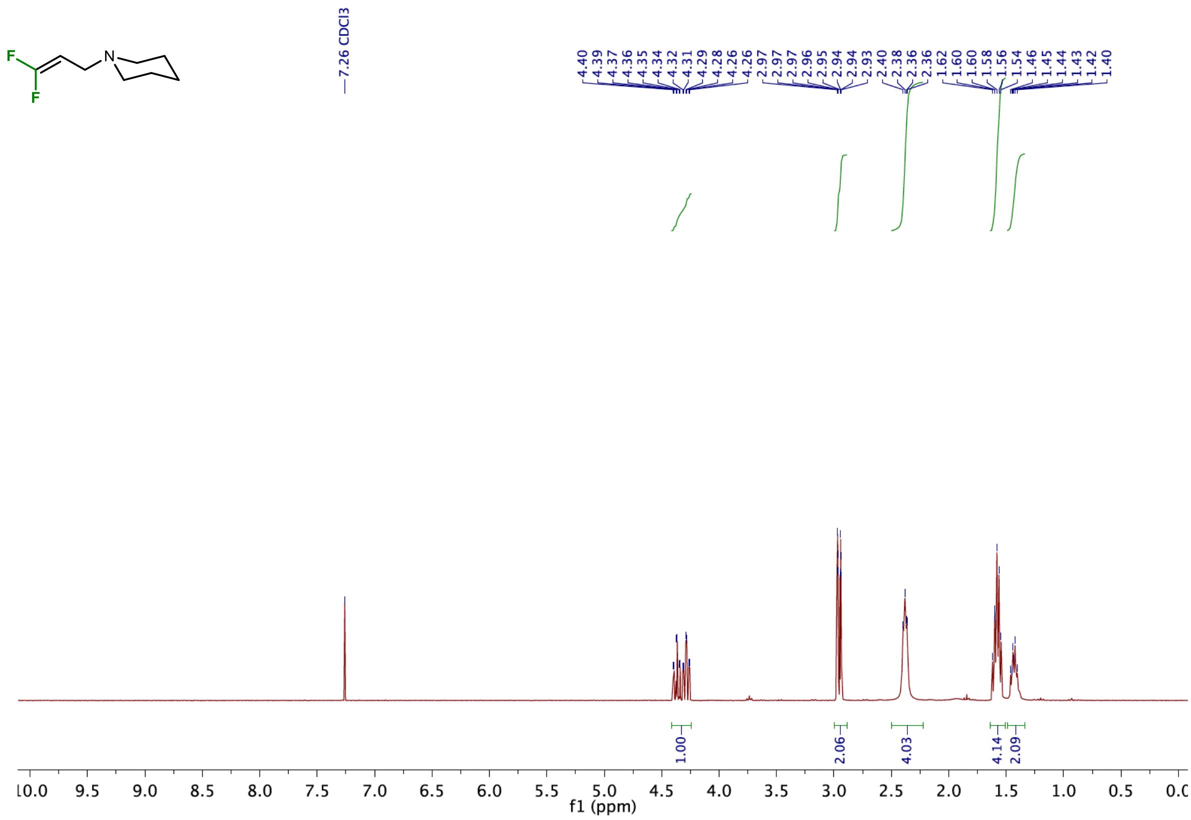

Supplementary Figure 6. ^{1}H NMR spectra of compound S1a

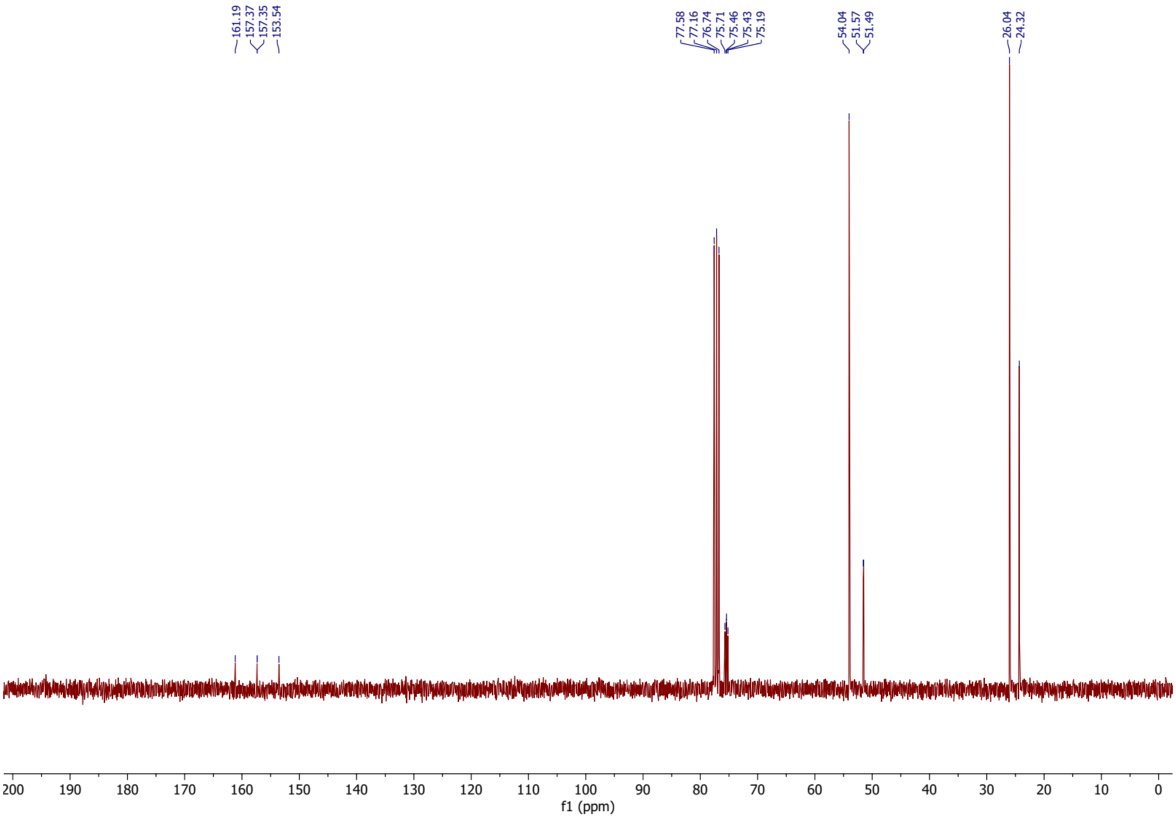

Supplementary Figure 7. ^{13}C NMR spectra of compound S1a

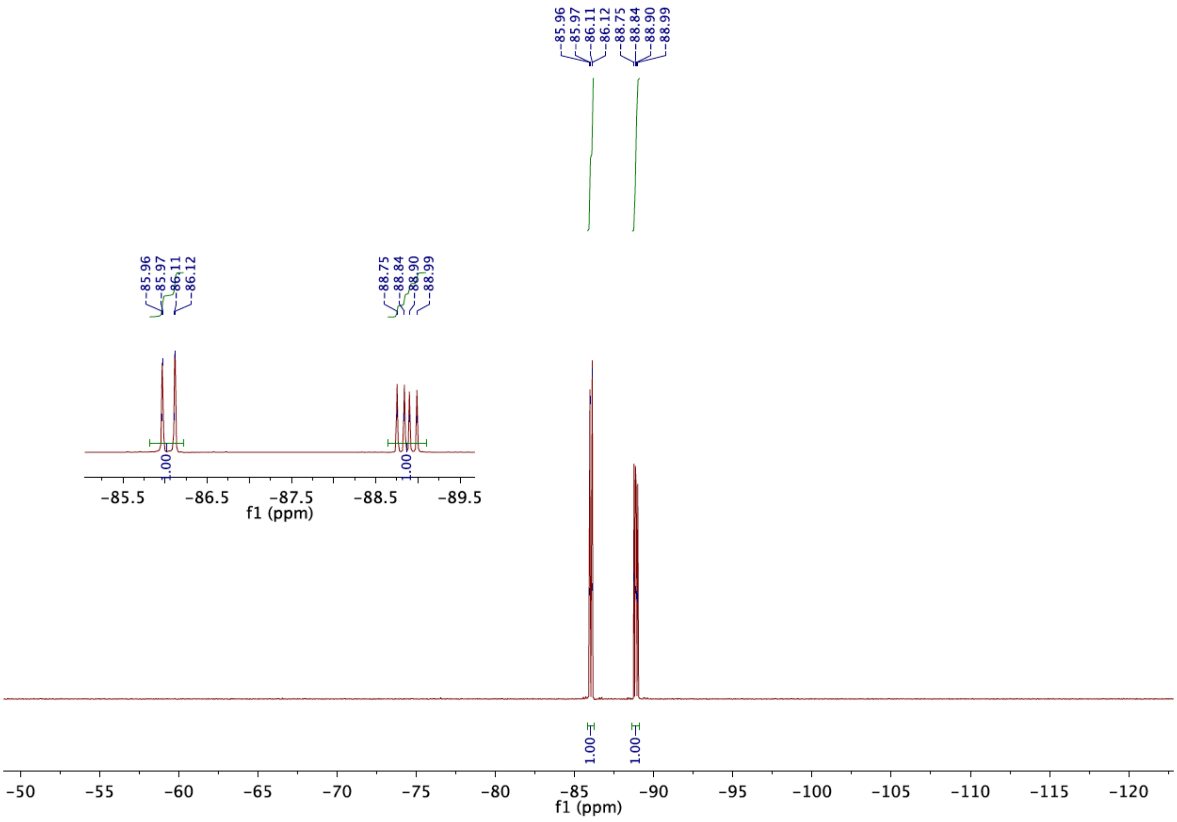

Supplementary Figure 8. ^{19}F NMR spectra of compound S1a

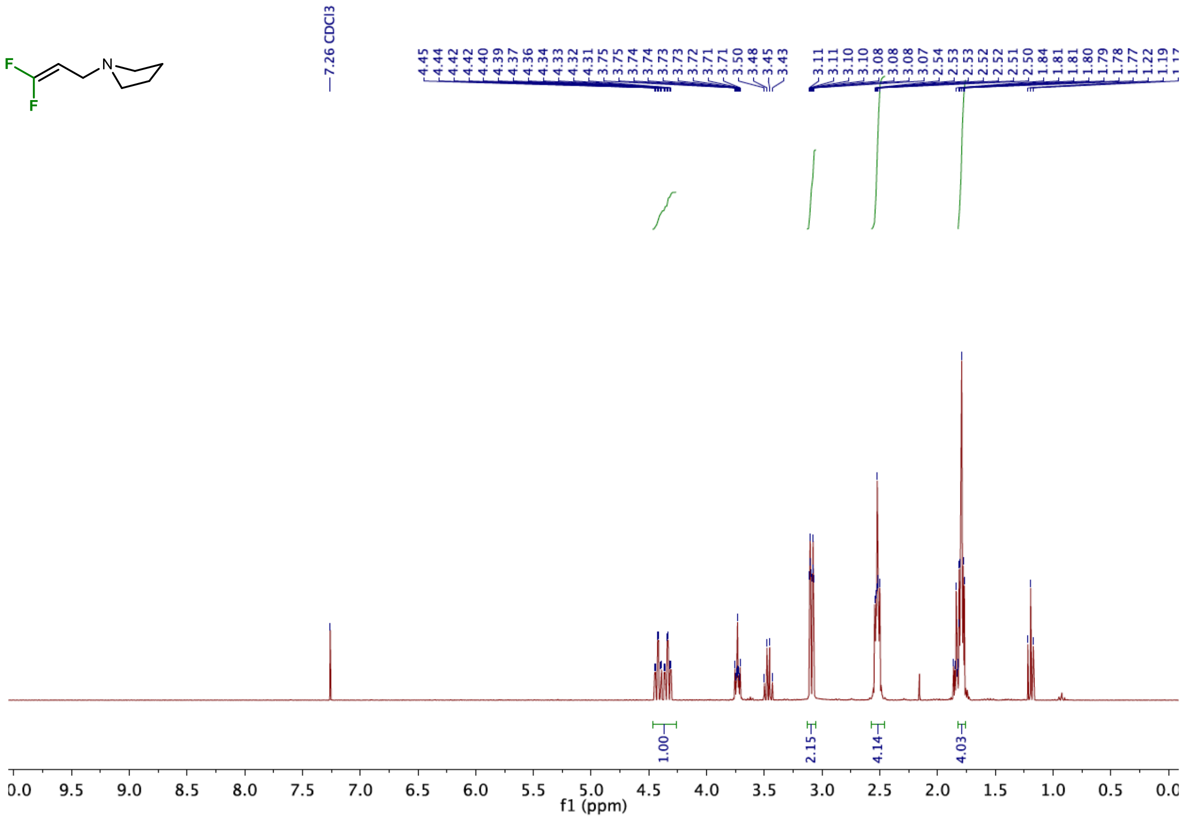

Supplementary Figure 9. ^{1}H NMR spectra of compound S1b

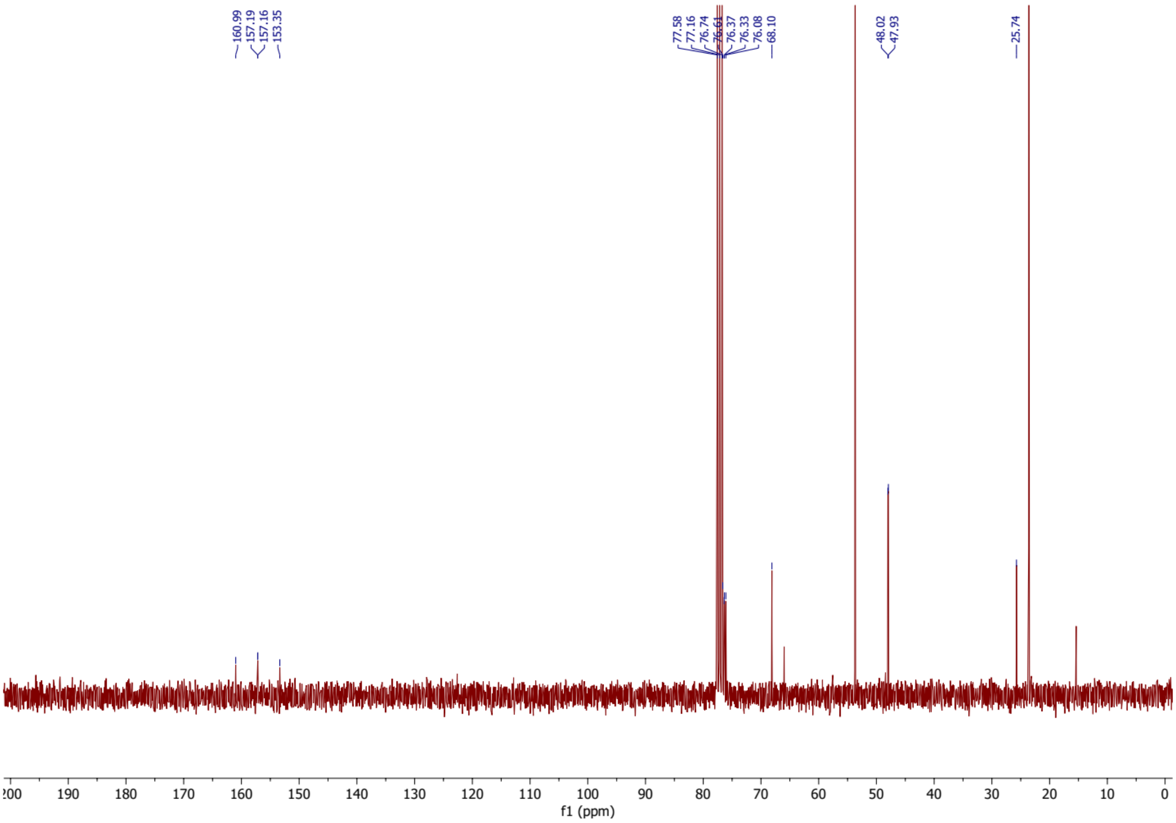

Supplementary Figure 10. ^{13}C NMR spectra of compound S1b

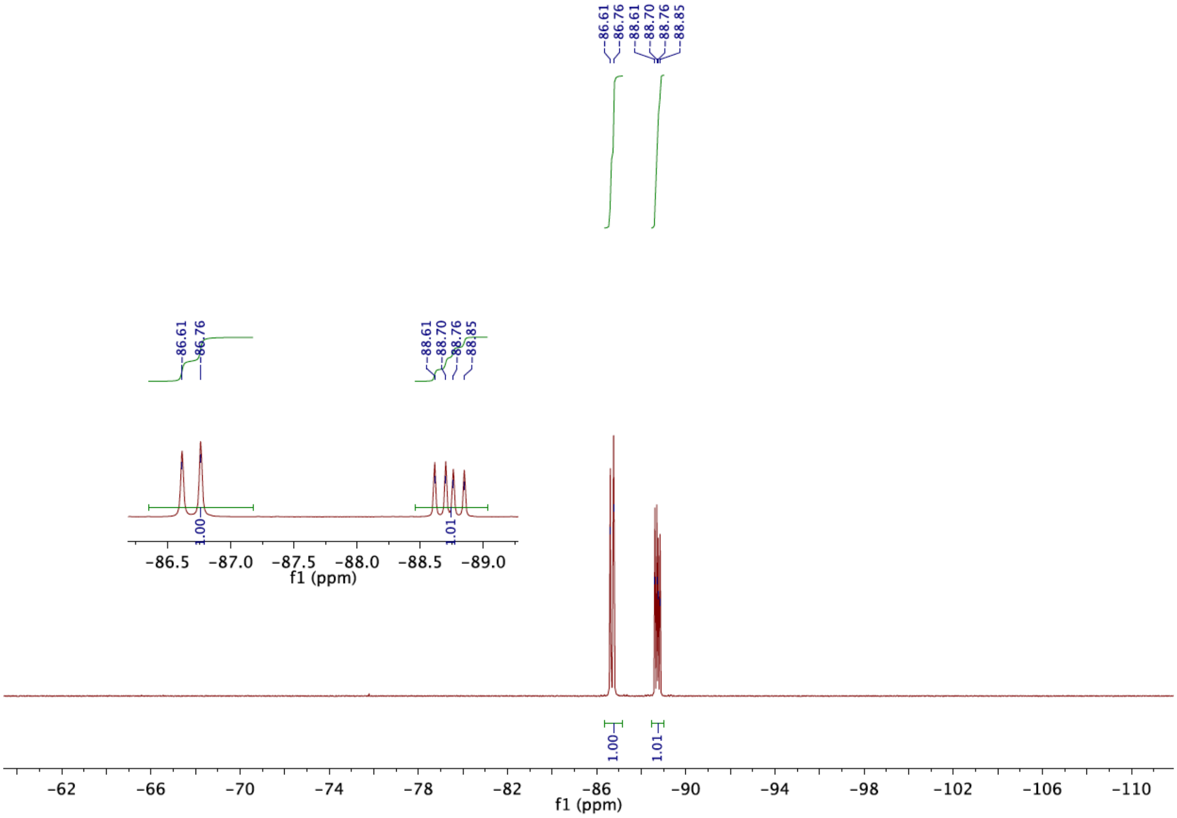

Supplementary Figure 11. ^{19}F NMR spectra of compound S1b

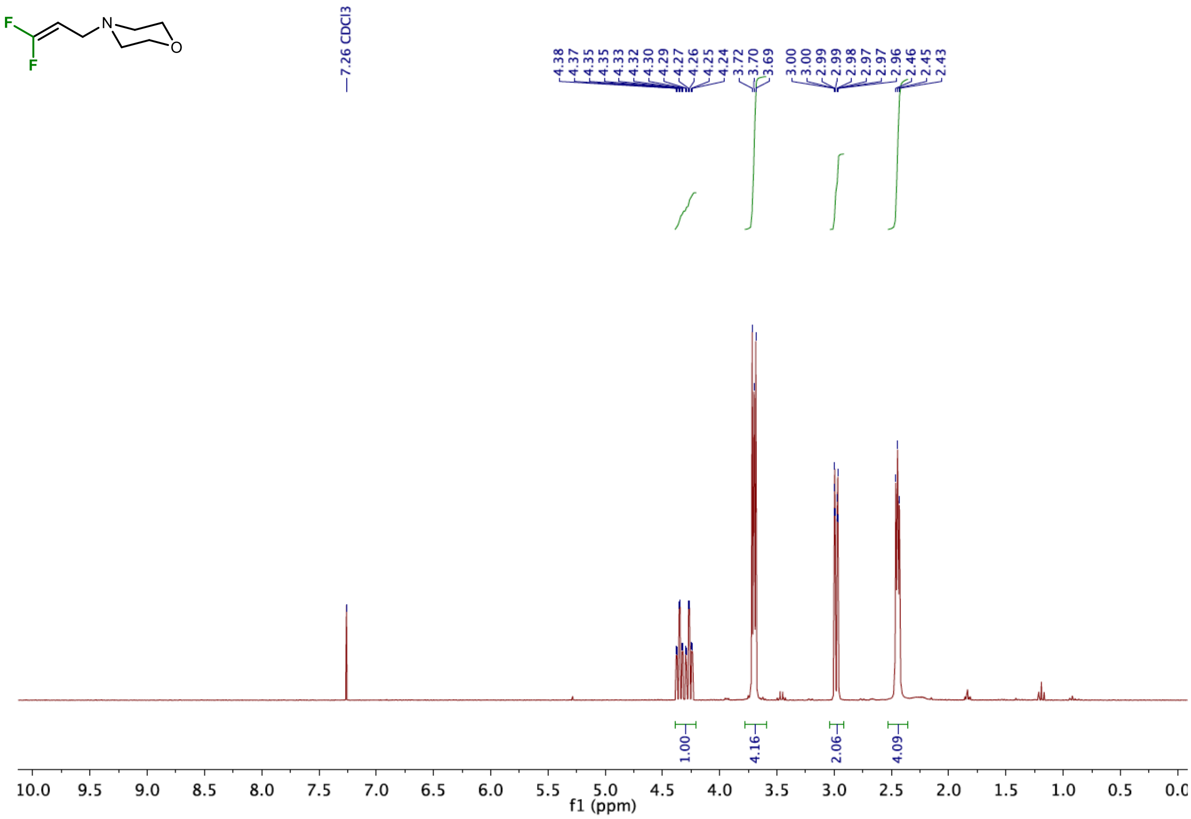

Supplementary Figure 12. ^{1}H NMR spectra of compound S1c

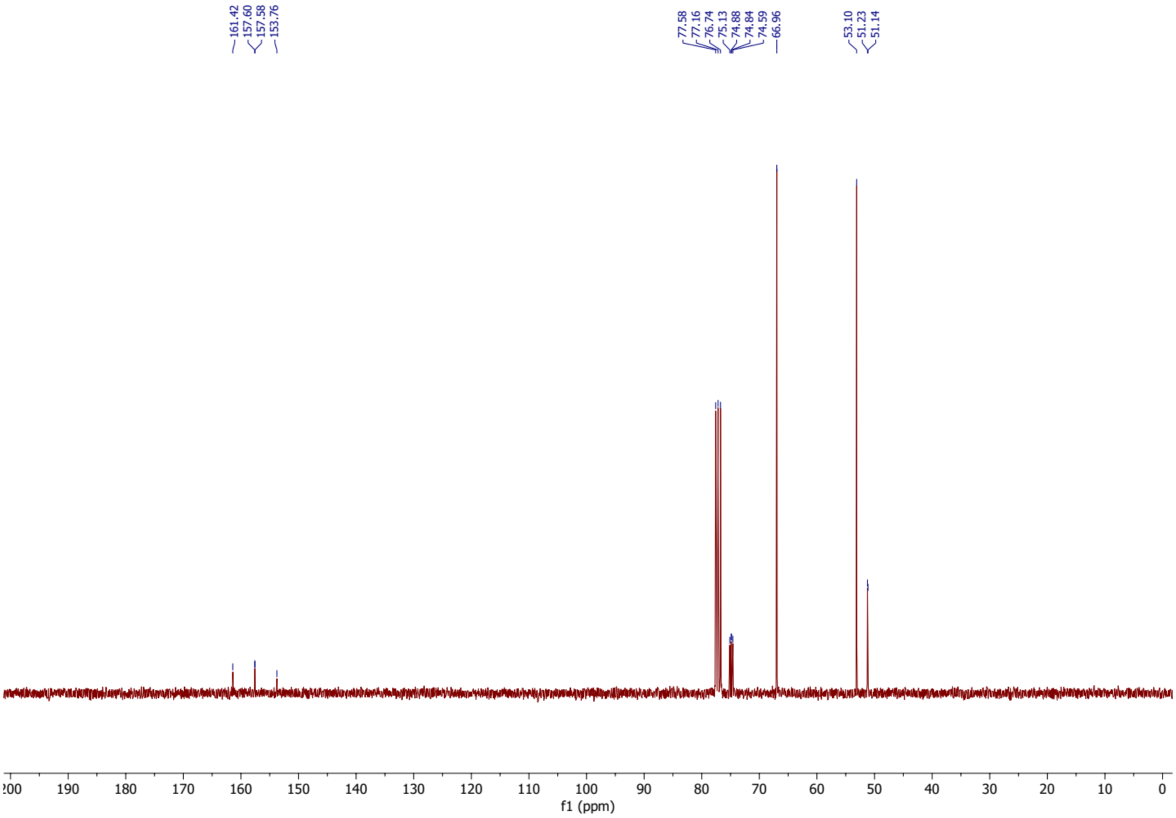

Supplementary Figure 13. ^{13}C NMR spectra of compound S1c

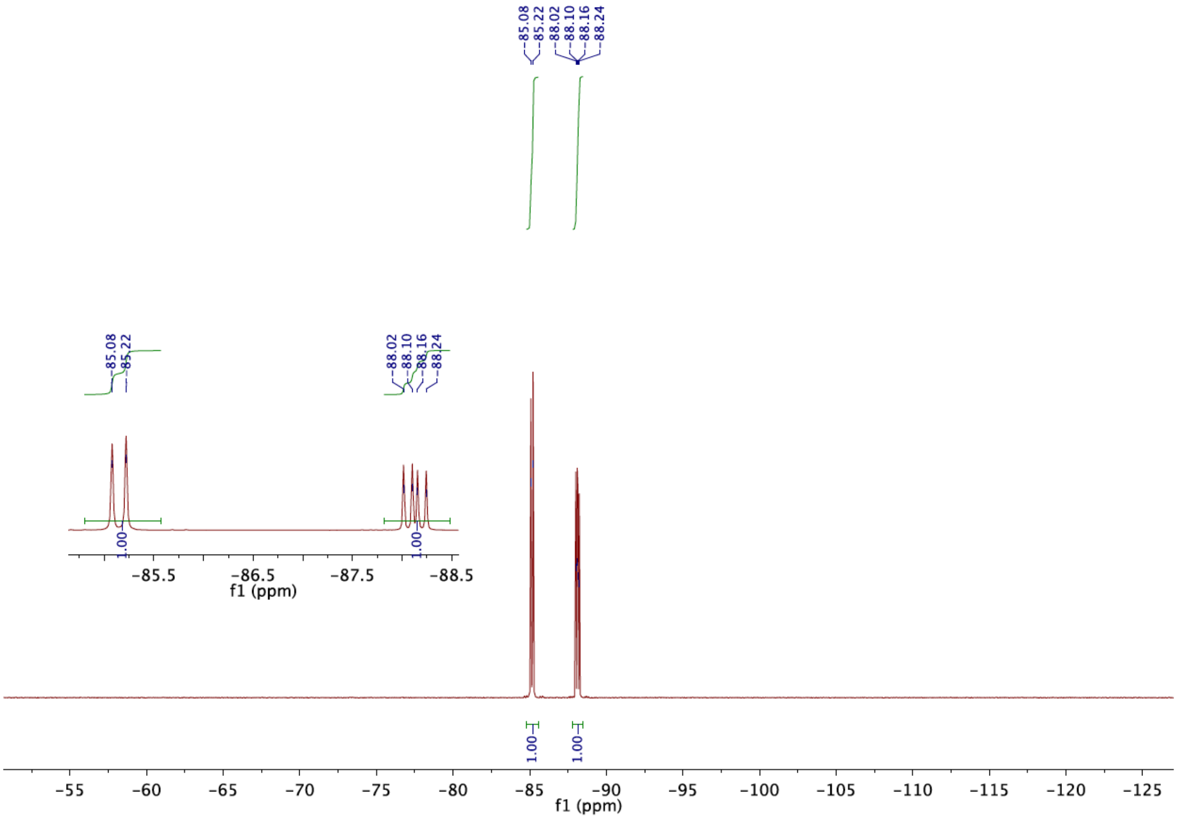

Supplementary Figure 14. ^{19}F NMR spectra of compound S1c

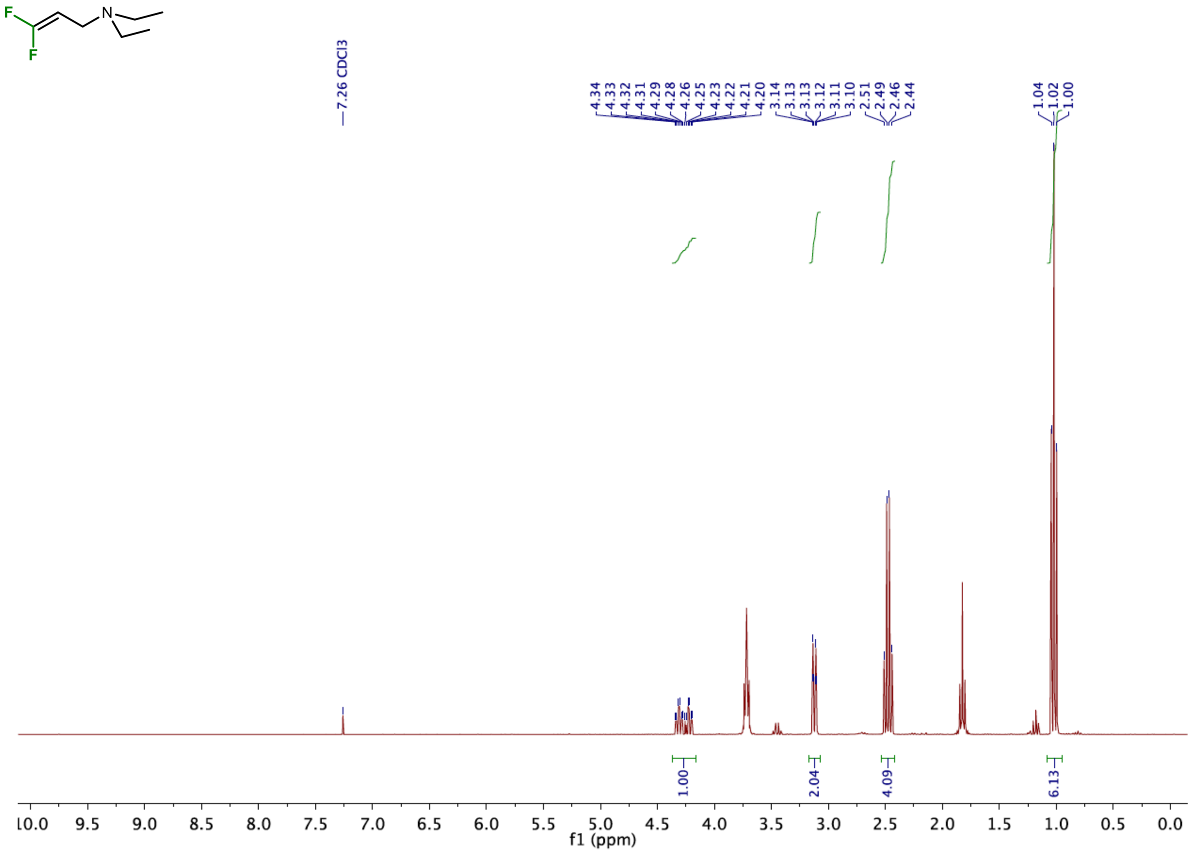

Supplementary Figure 15. ^{1}H NMR spectra of compound S1d

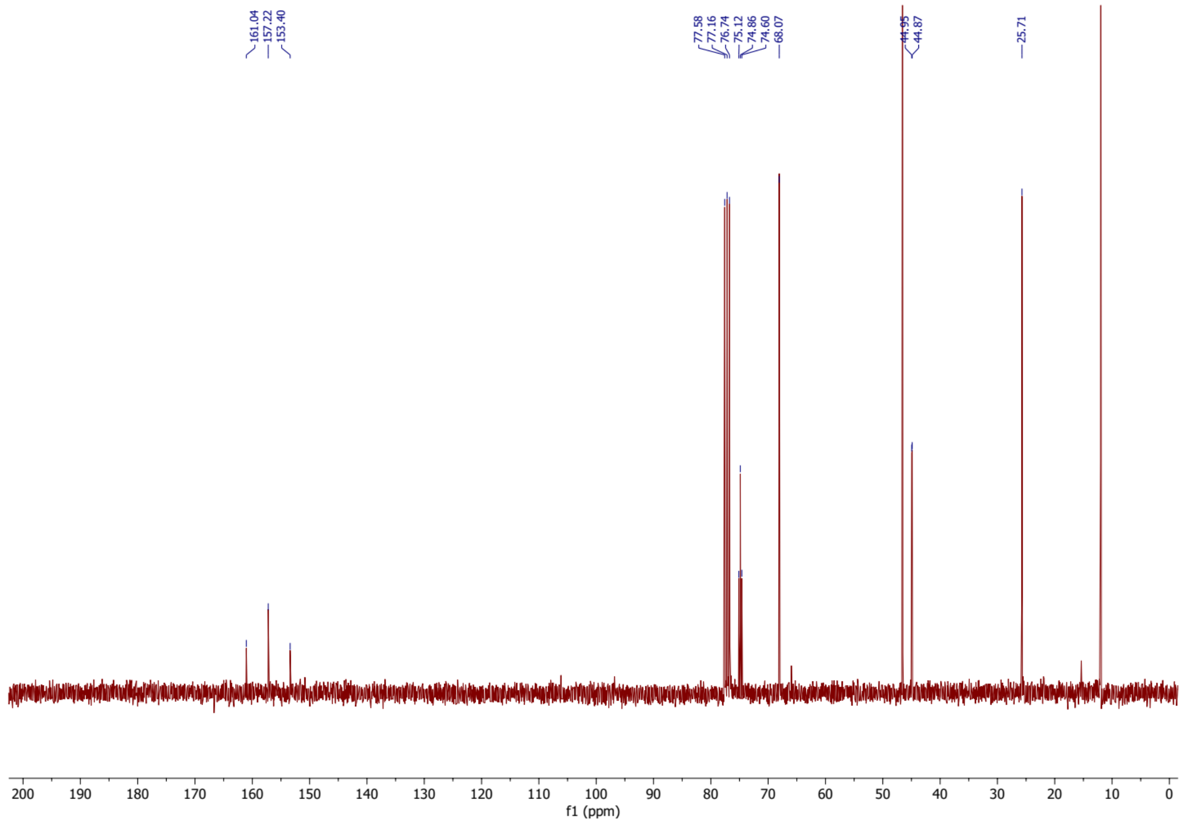

Supplementary Figure 16. ^{13}C NMR spectra of compound S1d

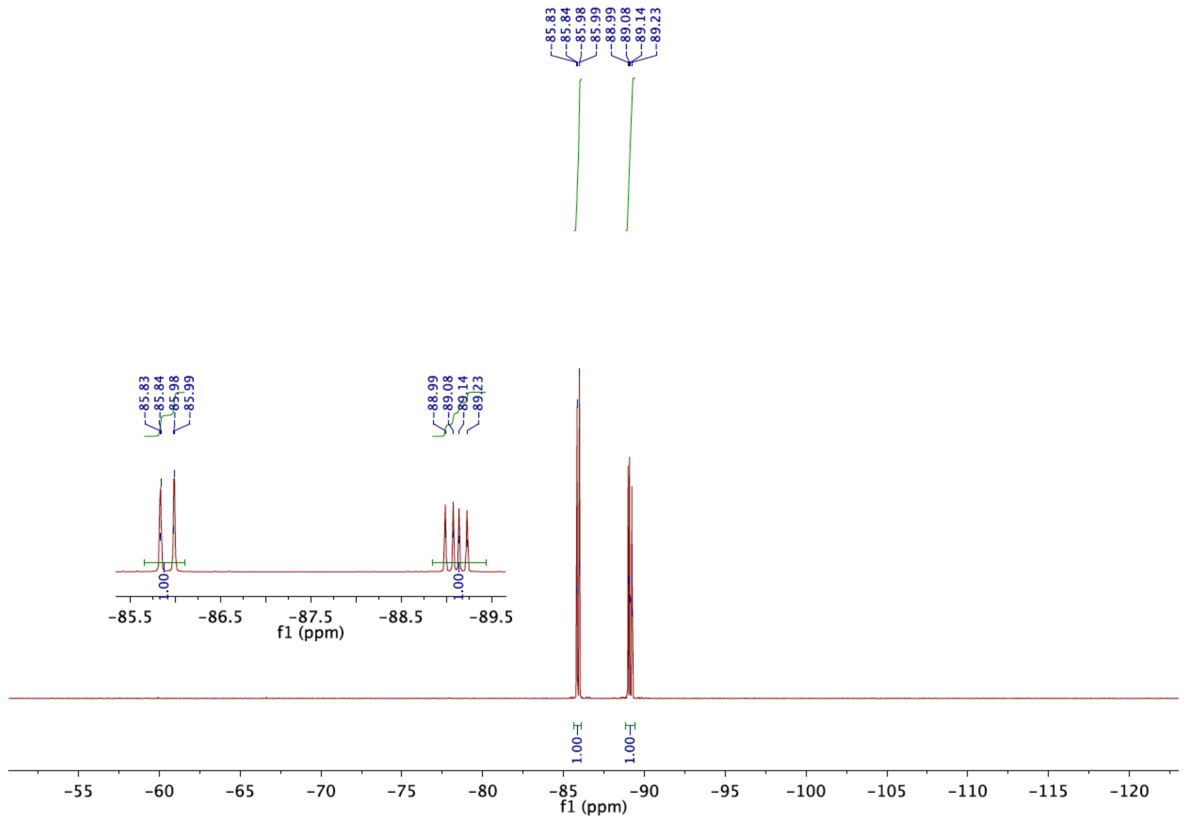

Supplementary Figure 17. ^{19}F NMR spectra of compound S1d

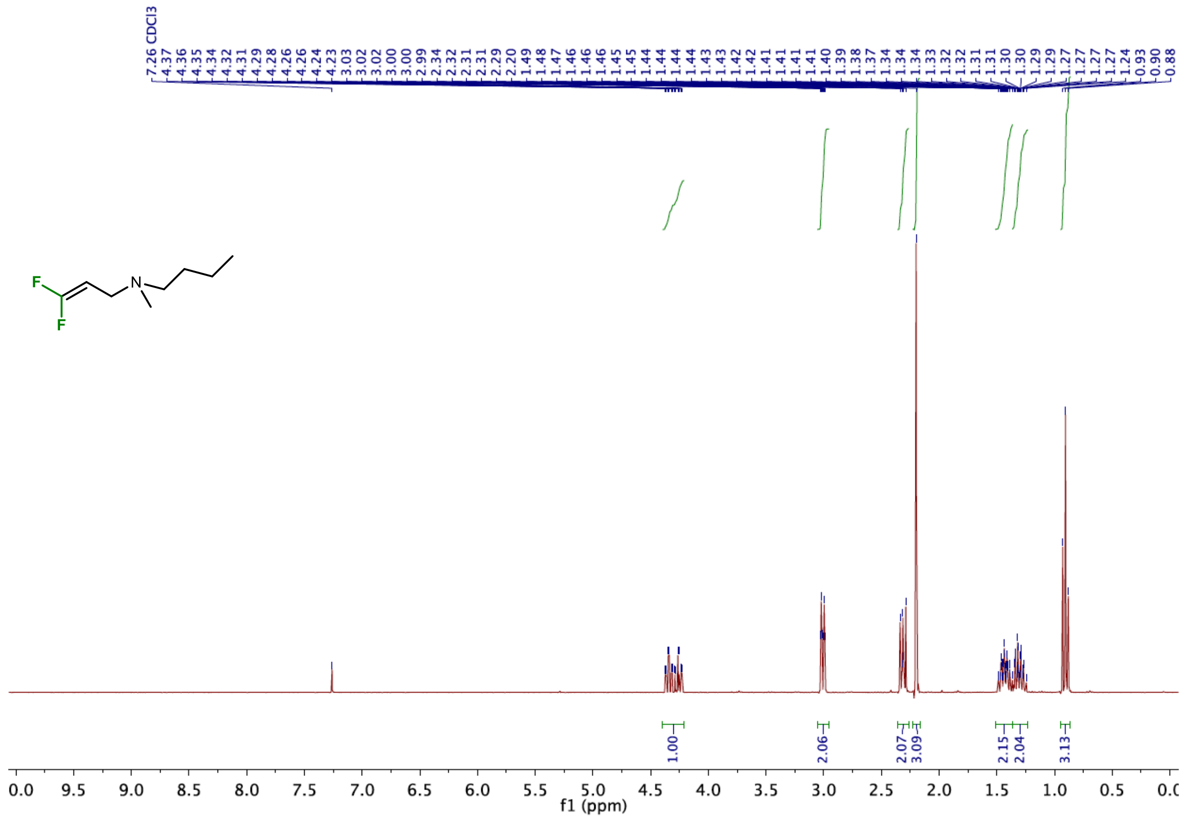

Supplementary Figure 18. ^{1}H NMR spectra of compound S1e

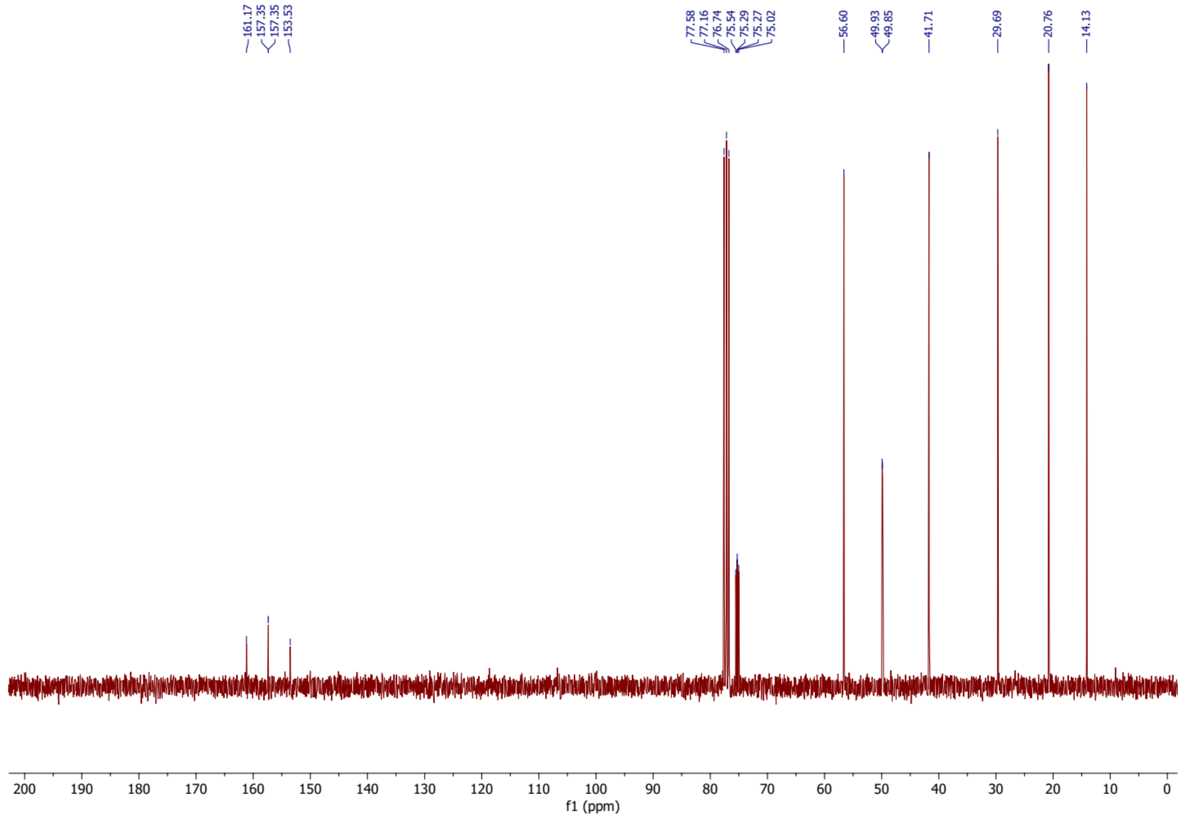

Supplementary Figure 19. ^{13}C NMR spectra of compound S1e

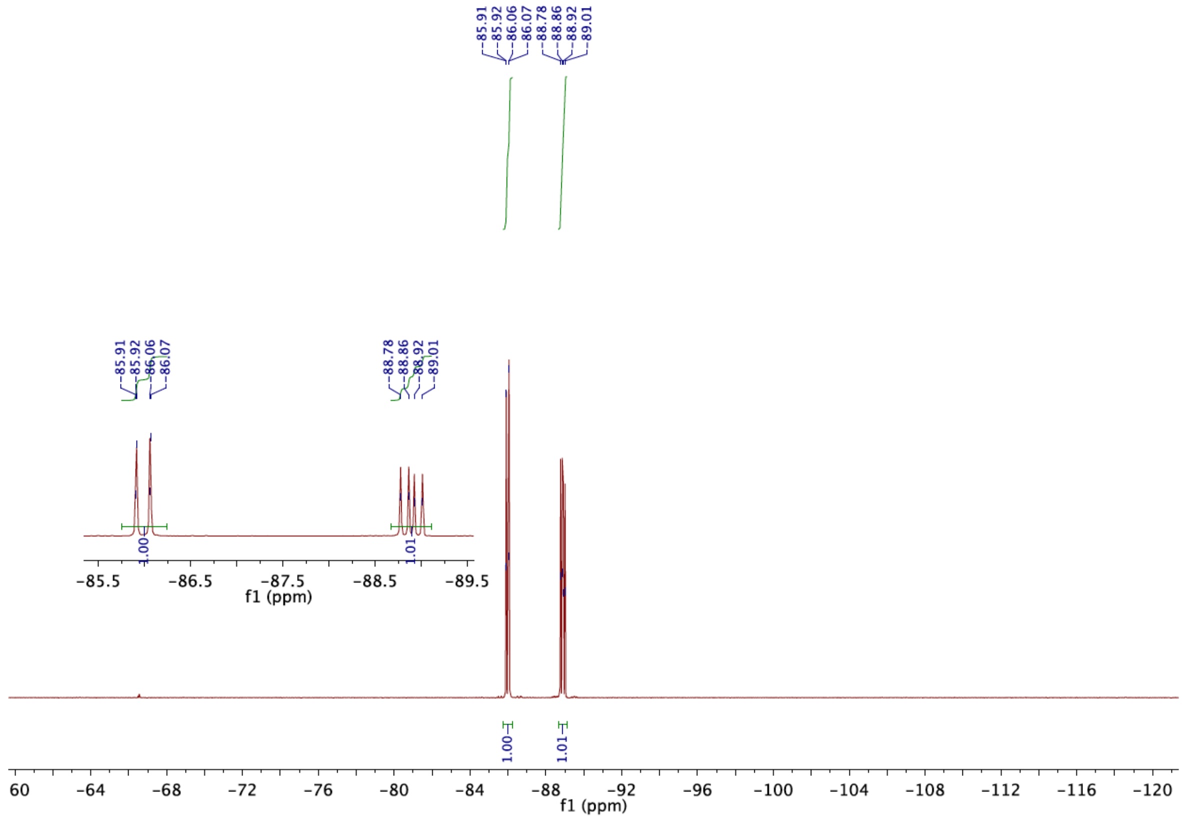

Supplementary Figure 20. ^{19}F NMR spectra of compound S1e

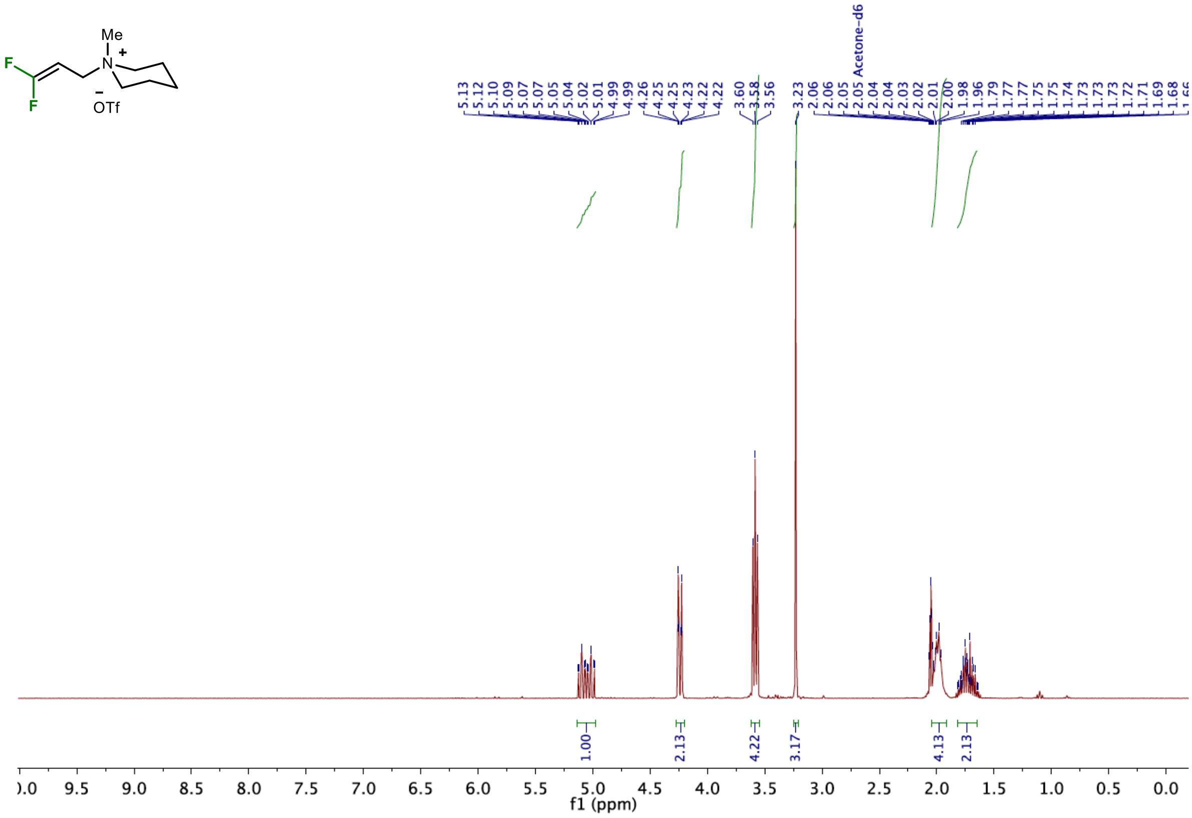

Supplementary Figure 21. ^{1}H NMR spectra of compound 1a

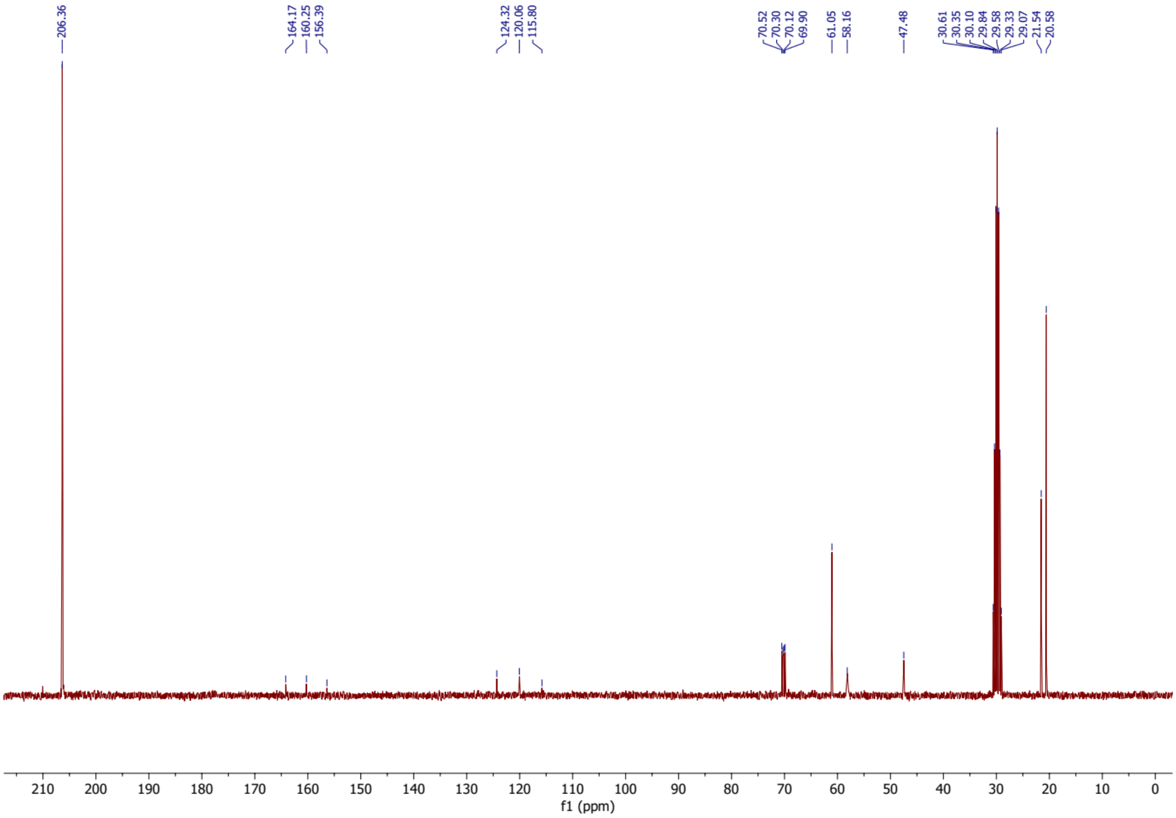

Supplementary Figure 22. ^{13}C NMR spectra of compound 1a

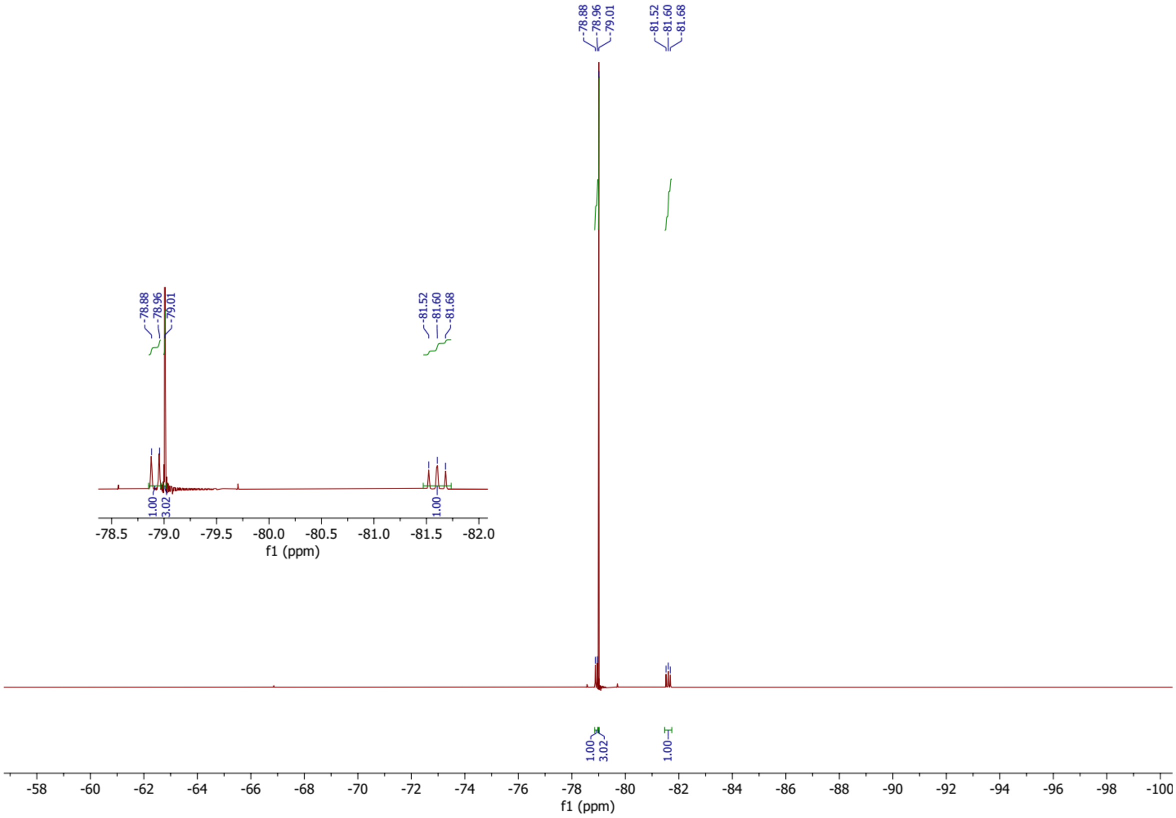

Supplementary Figure 23. ^{19}F NMR spectra of compound 1a

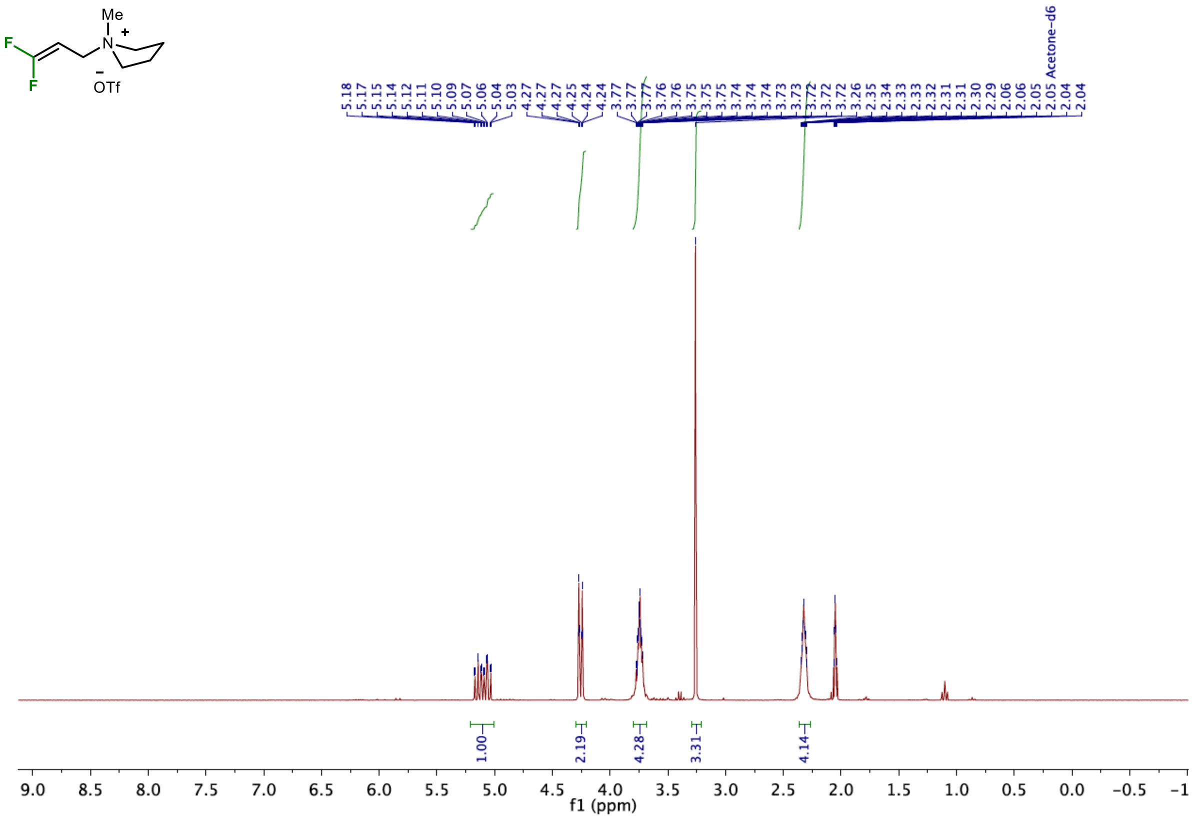

Supplementary Figure 24. ^{1}**H NMR spectra of compound 1b**

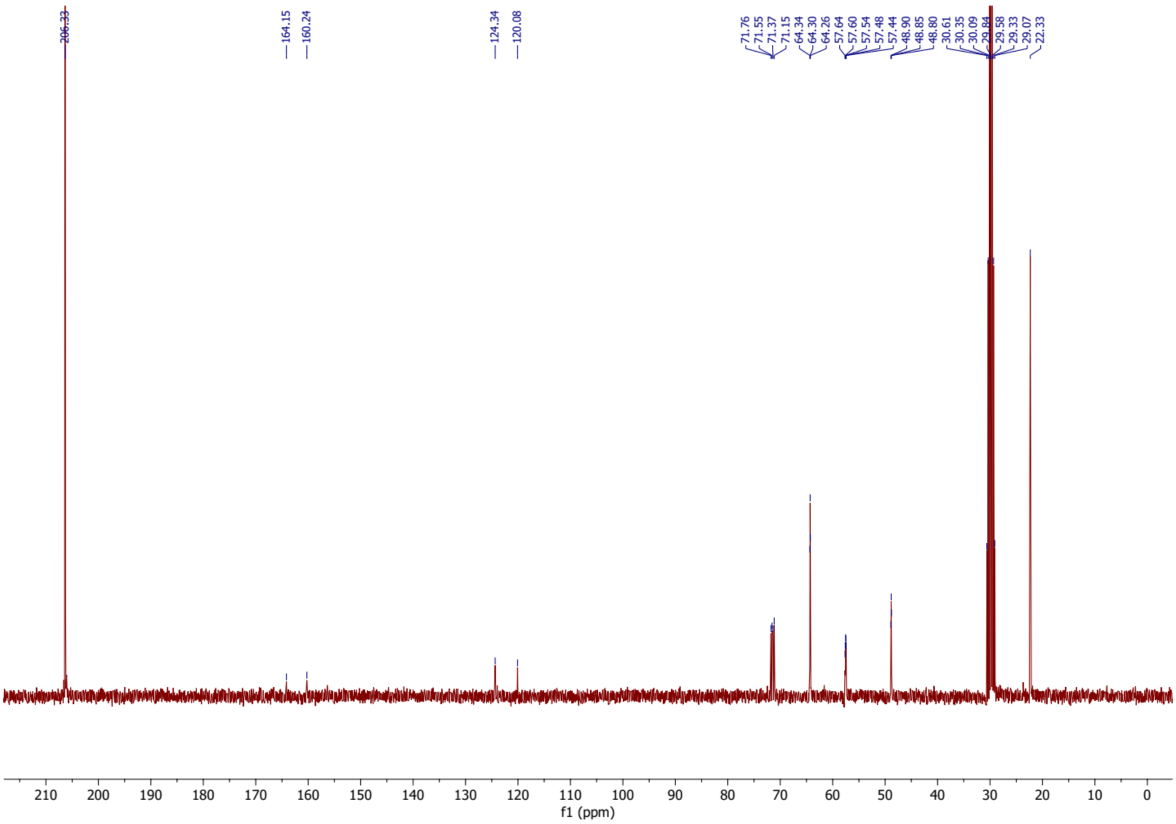

Supplementary Figure 25. ^{13}**C NMR spectra of compound 1b**

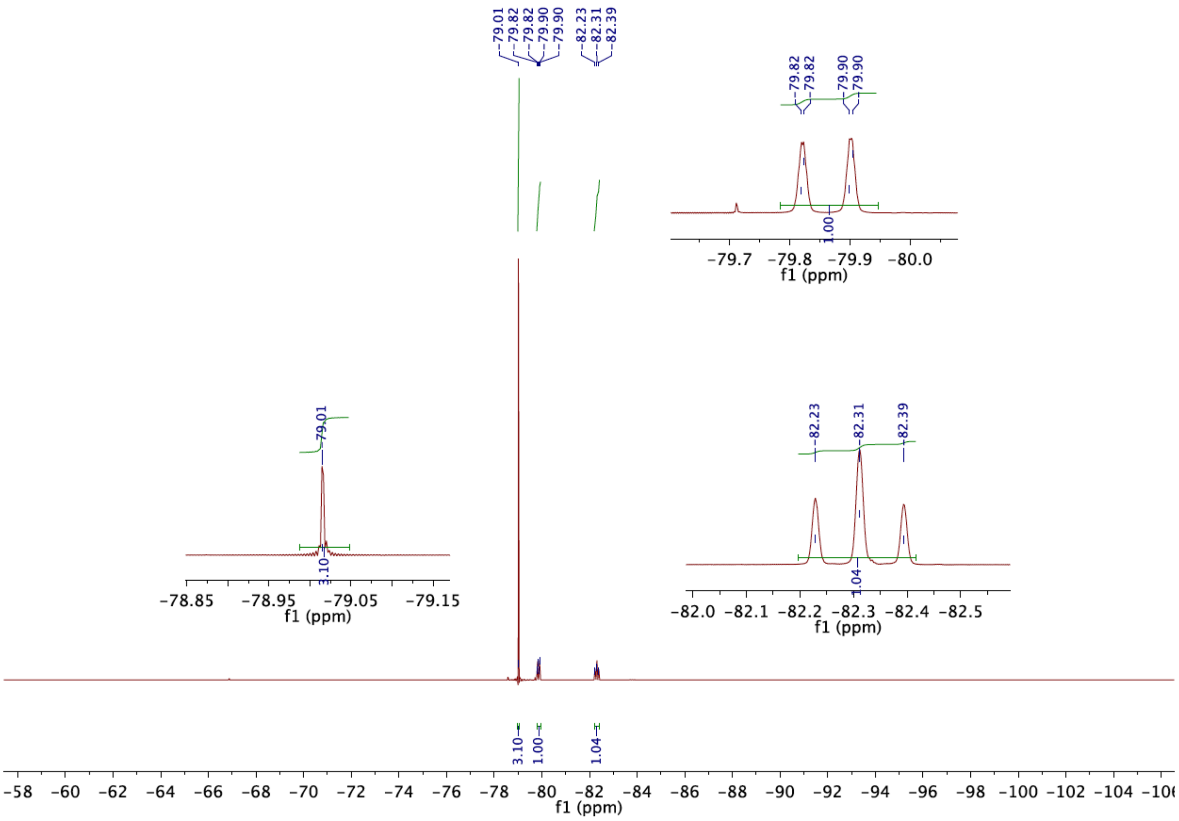

Supplementary Figure 26. ^{19}F NMR spectra of compound 1b

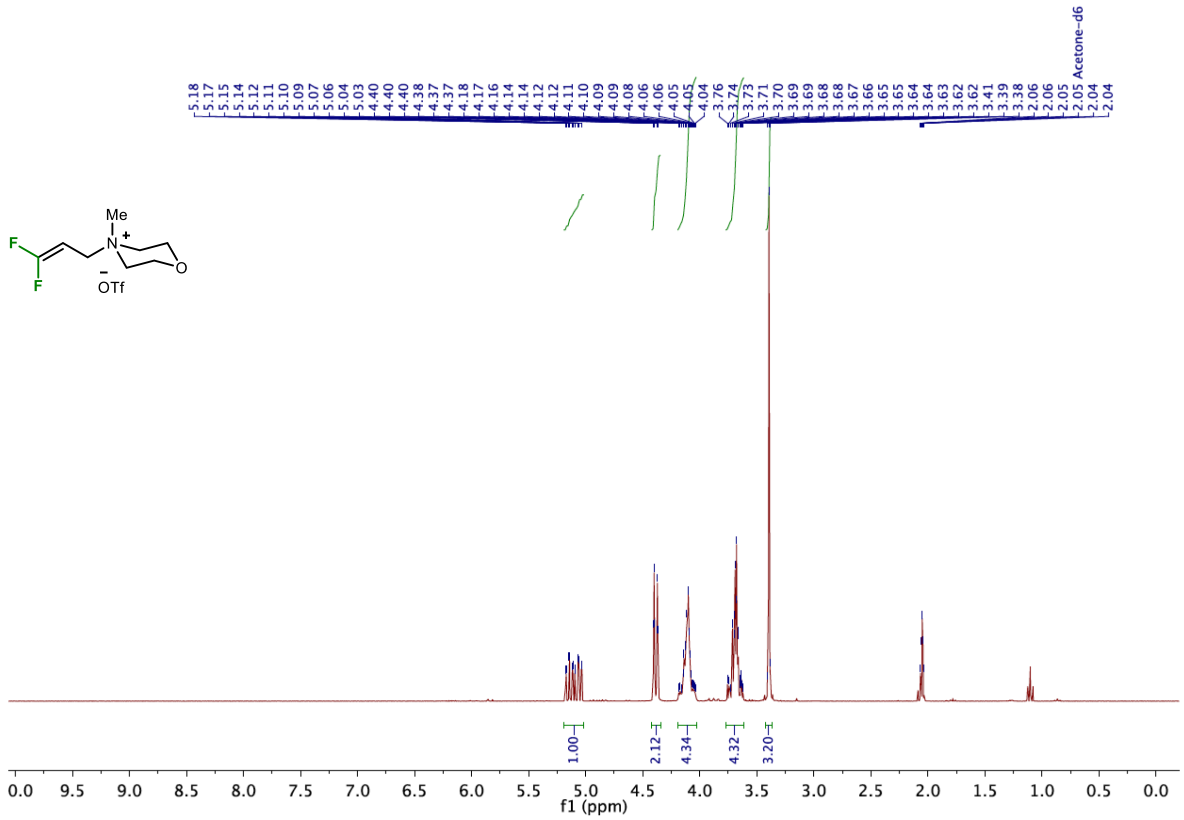

Supplementary Figure 27. ^{1}H NMR spectra of compound 1c

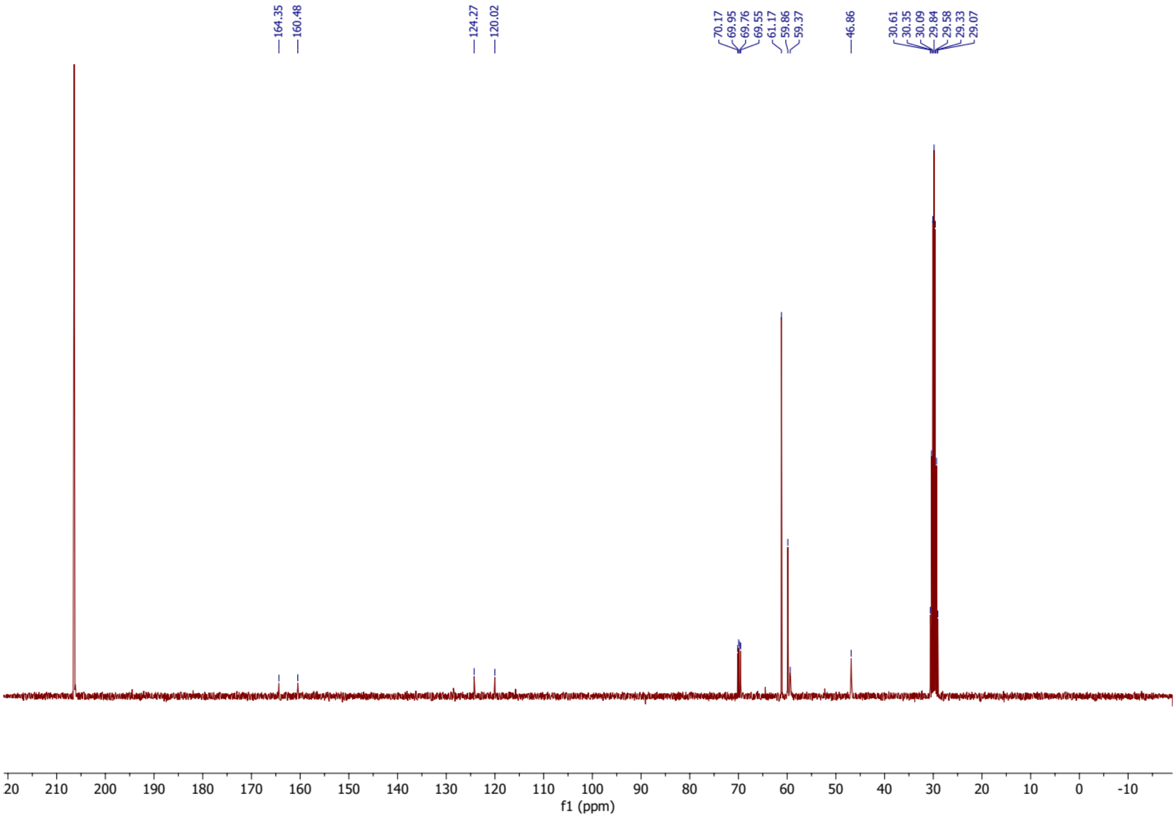

Supplementary Figure 28. ^{13}C NMR spectra of compound 1c

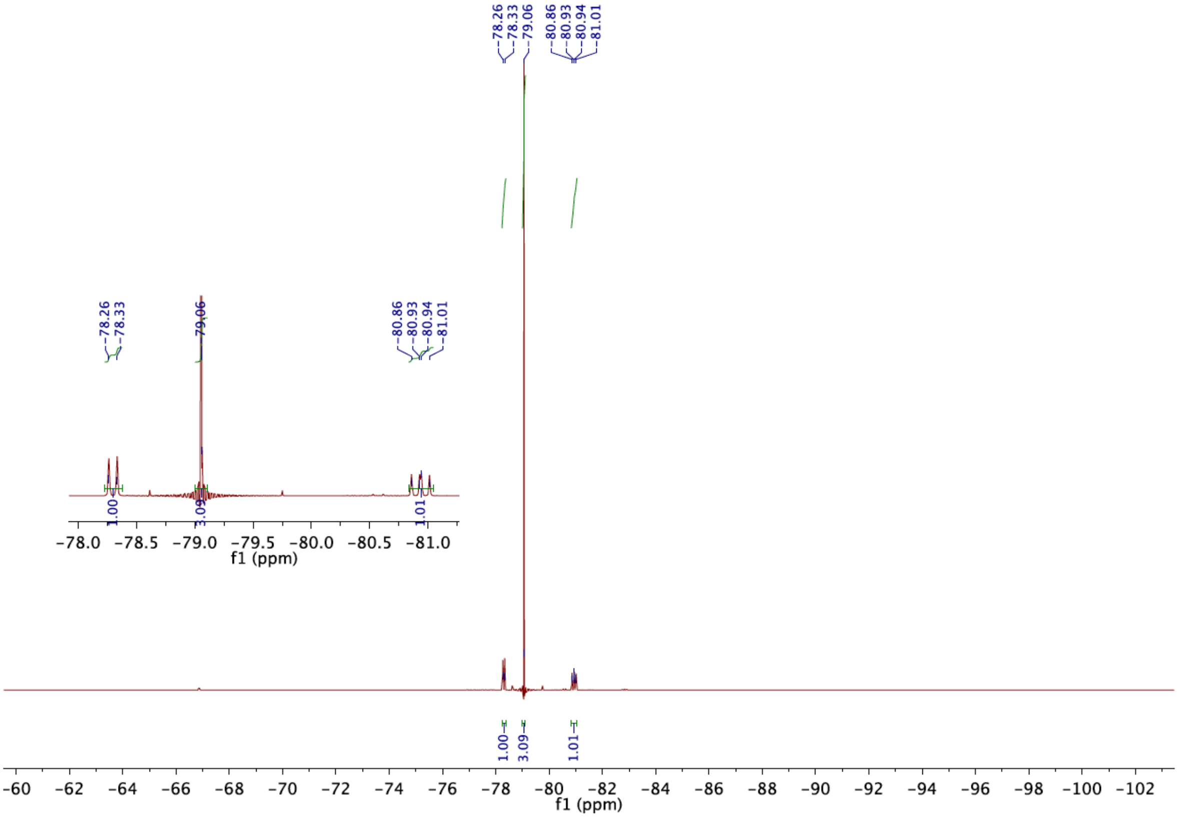

Supplementary Figure 29. ^{19}F NMR spectra of compound 1c

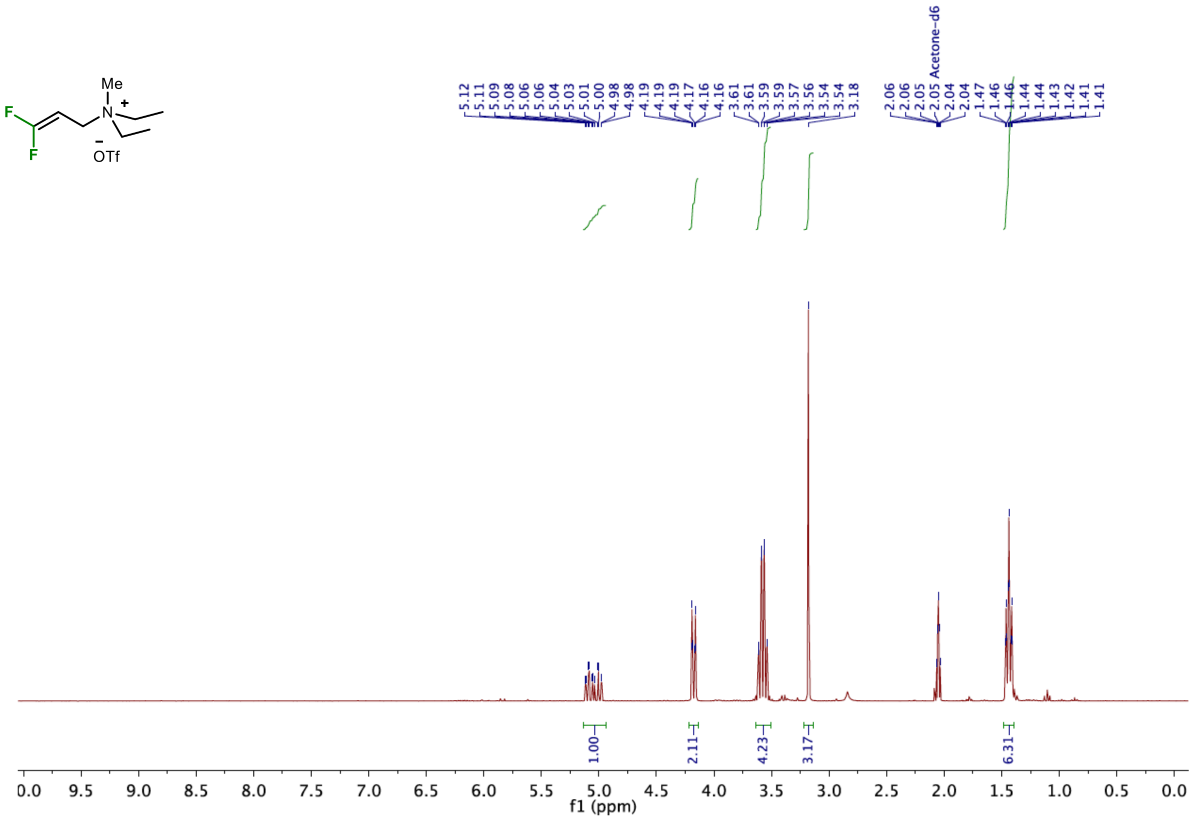

Supplementary Figure 30. ^{1}H NMR spectra of compound 1d

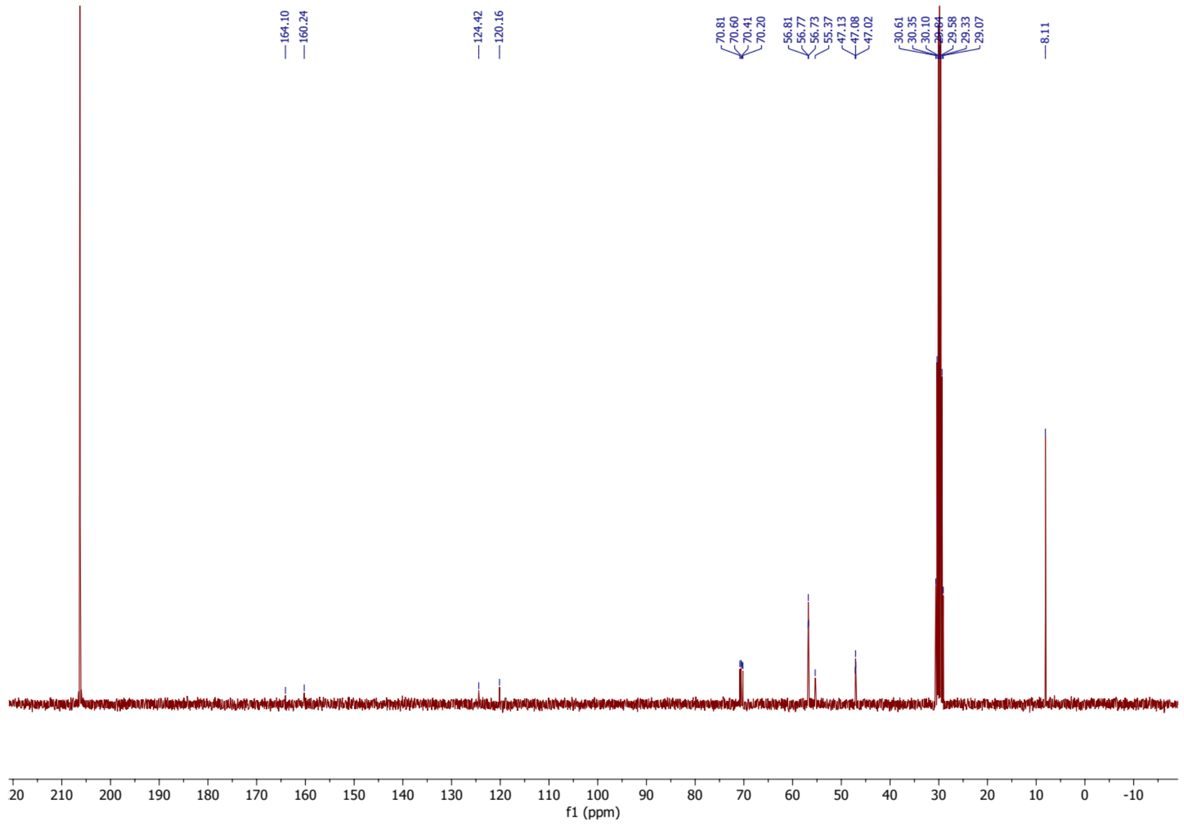

Supplementary Figure 31. ^{13}C NMR spectra of compound 1d

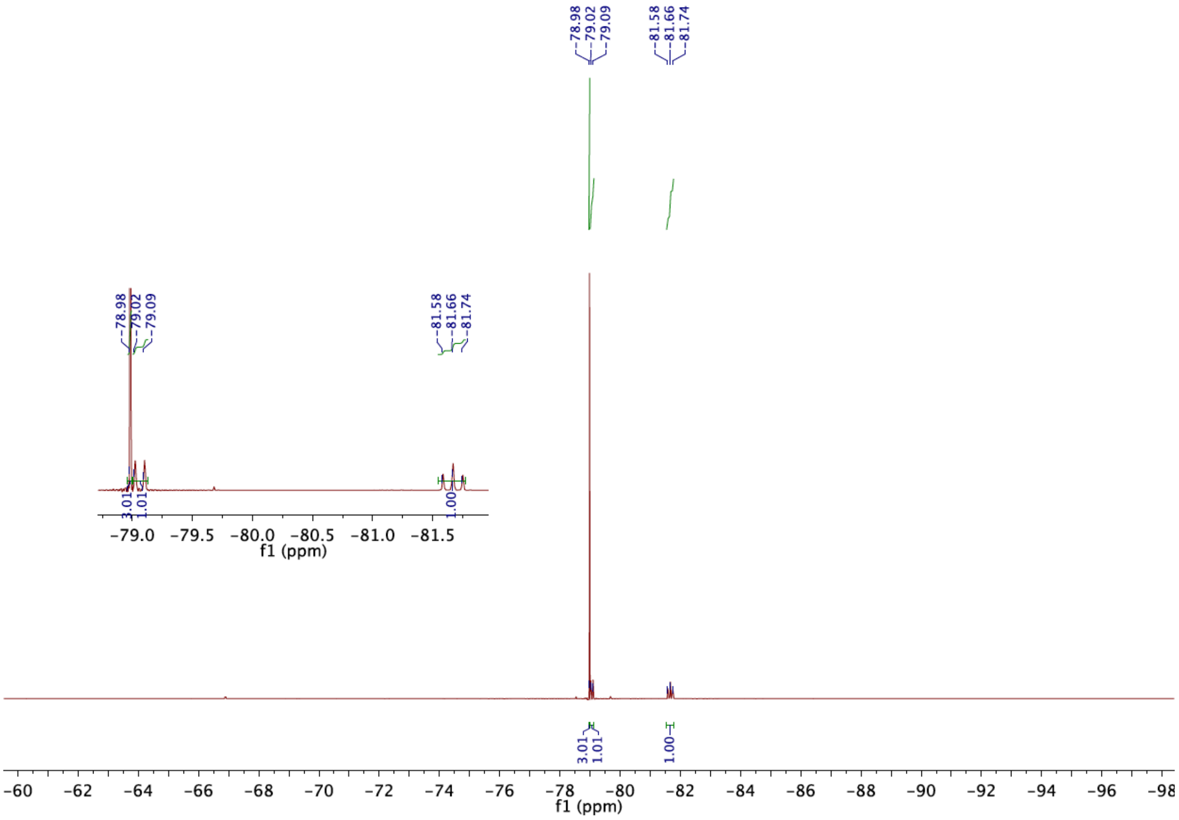

Supplementary Figure 32. ^{19}F NMR spectra of compound 1d

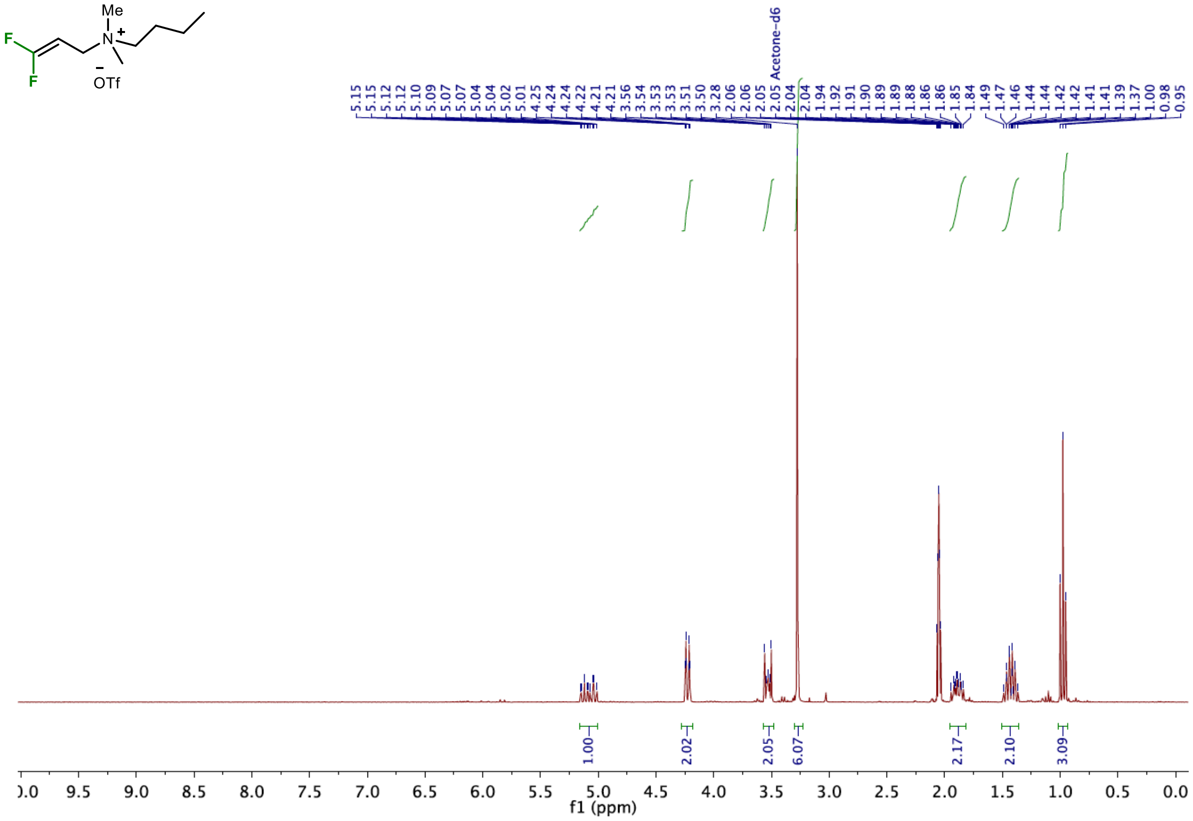

Supplementary Figure 33. ^{1}H NMR spectra of compound 1e

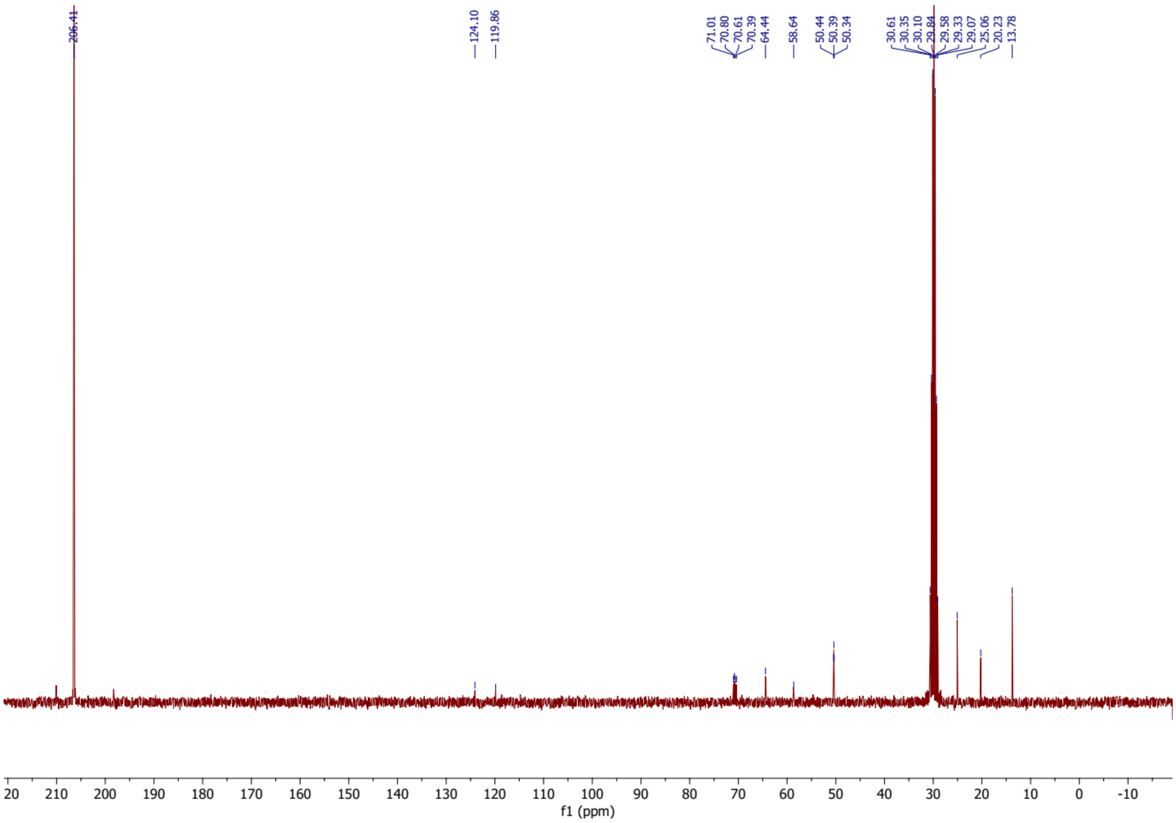

Supplementary Figure 34. ^{13}C NMR spectra of compound 1e

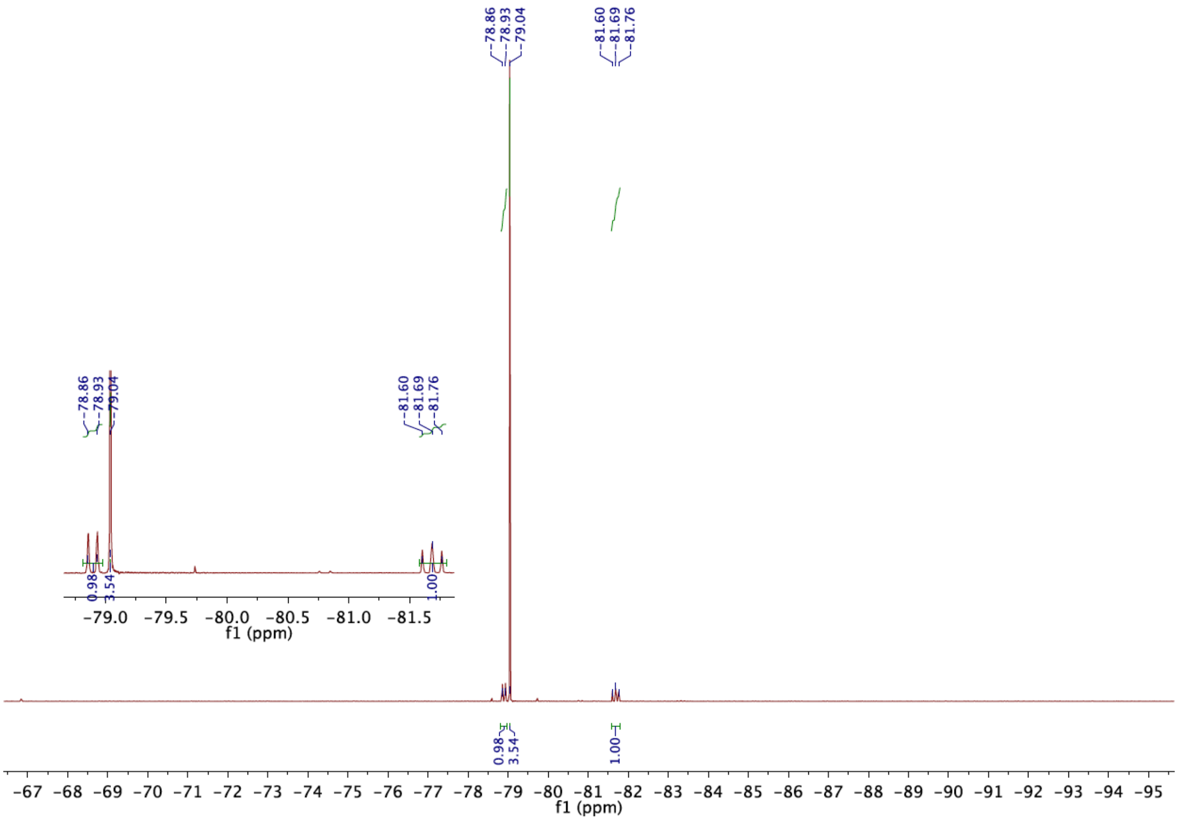

Supplementary Figure 35. ^{19}F NMR spectra of compound 1e

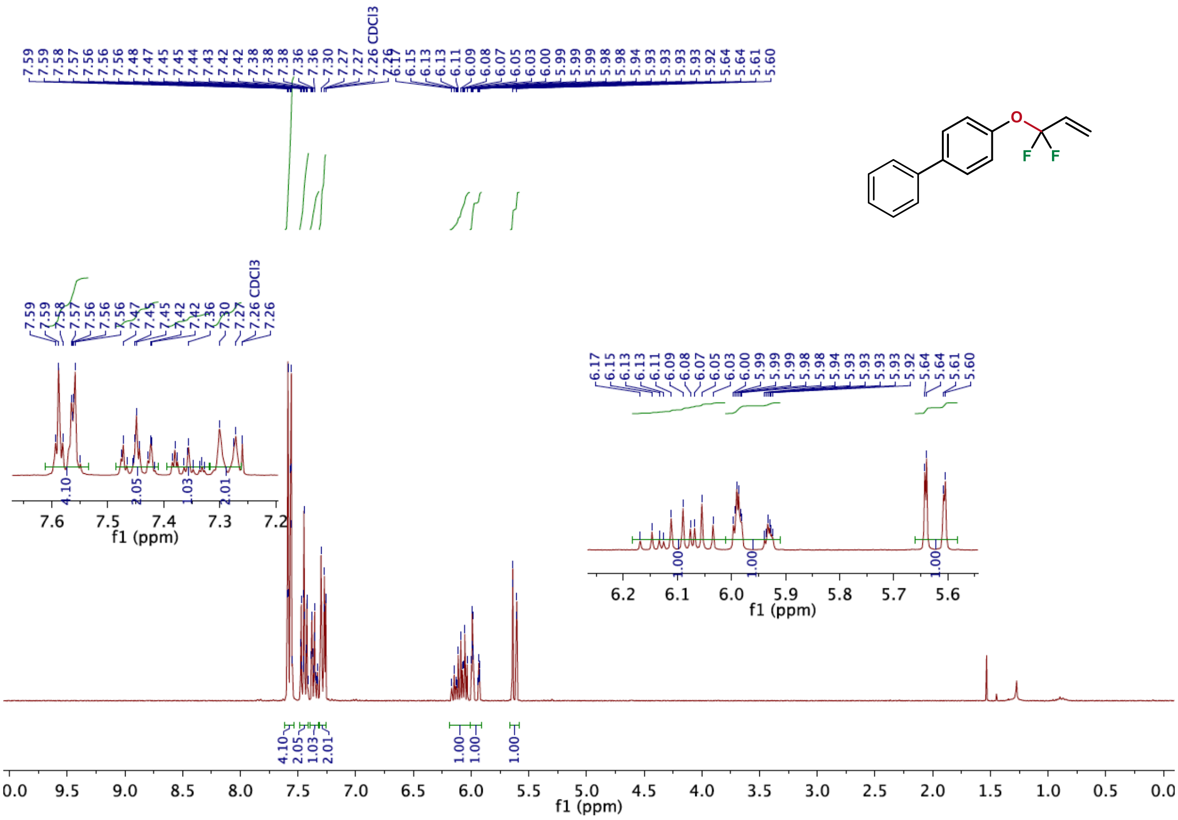

Supplementary Figure 36. ^{1}H NMR spectra of compound 3

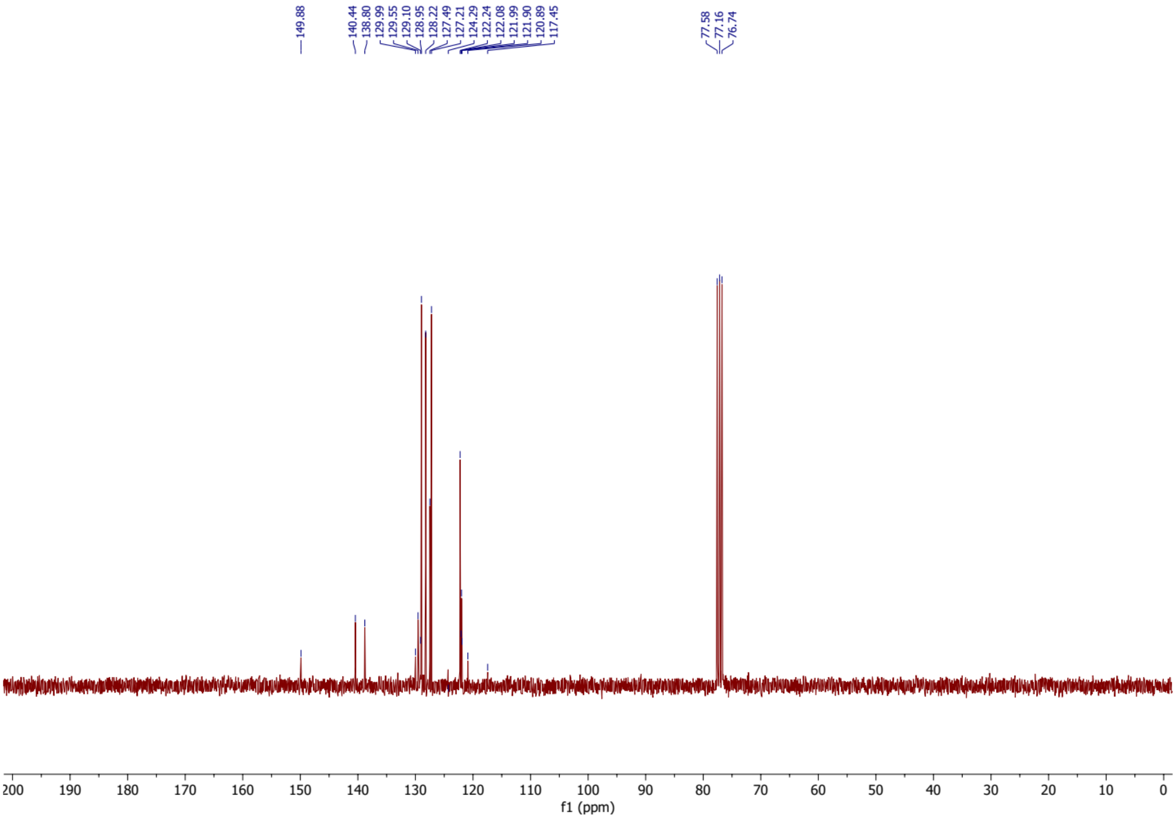

Supplementary Figure 37. ^{13}C NMR spectra of compound 3

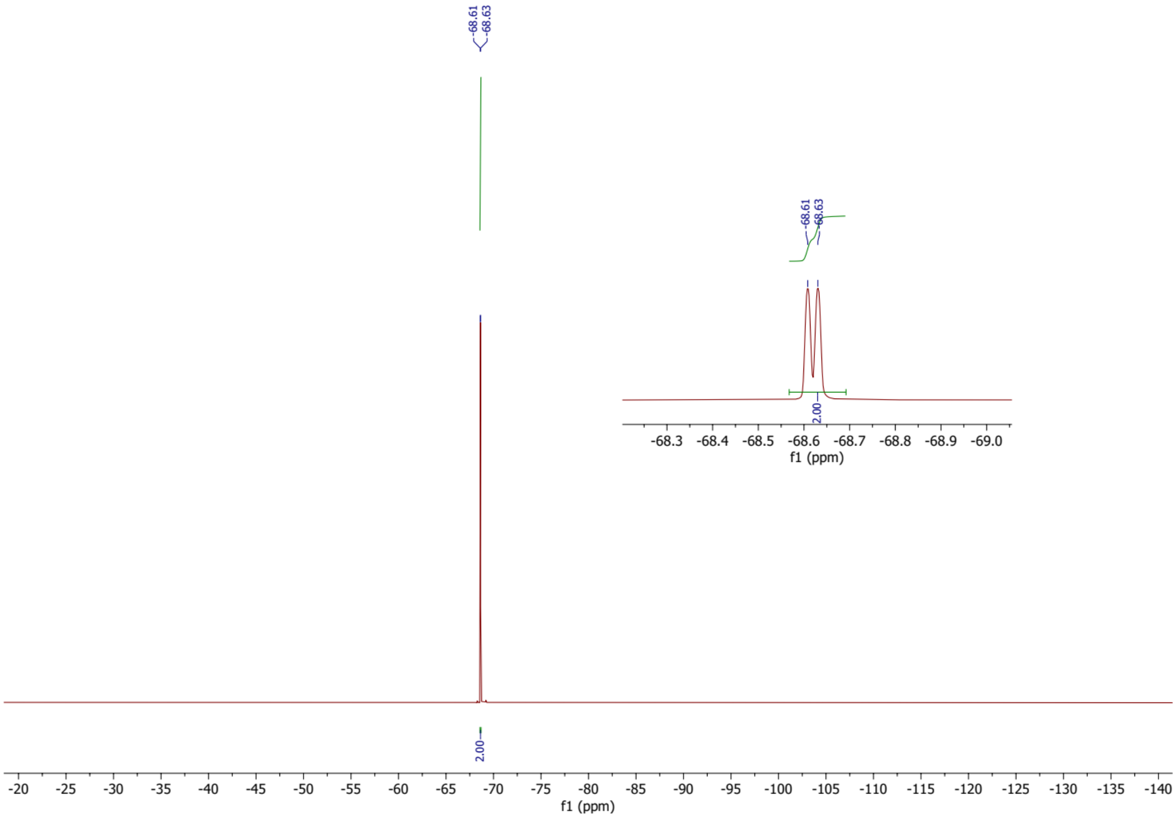

Supplementary Figure 38. ^{19}F NMR spectra of compound 3

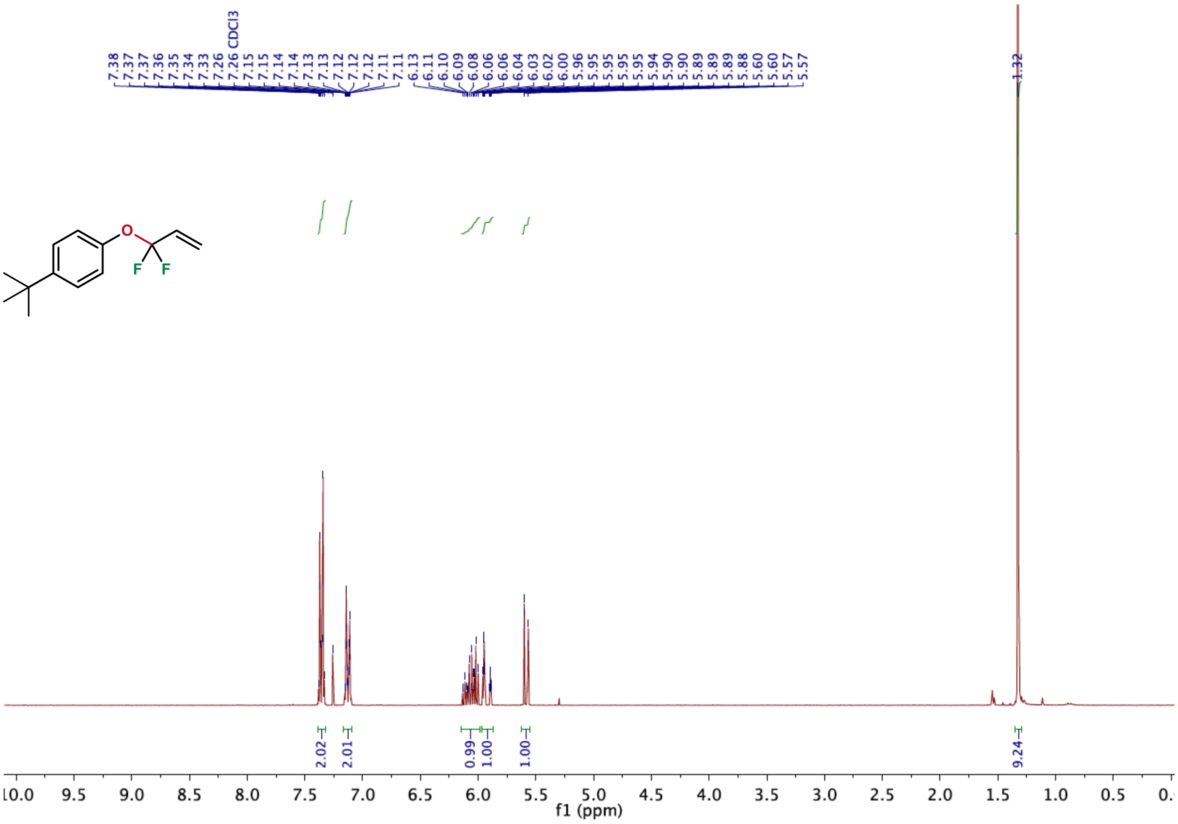

Supplementary Figure 39. ^{1}H NMR spectra of compound 4

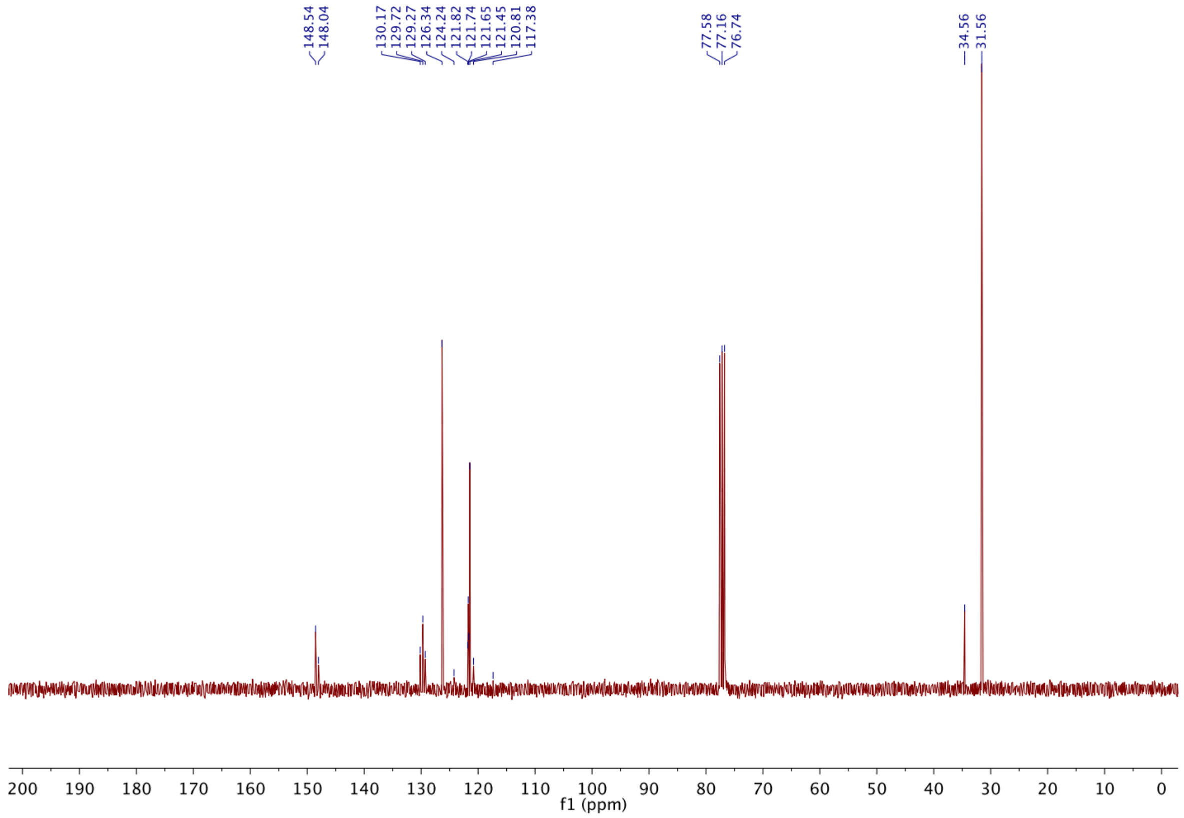

Supplementary Figure 40. ^{13}C NMR spectra of compound 4

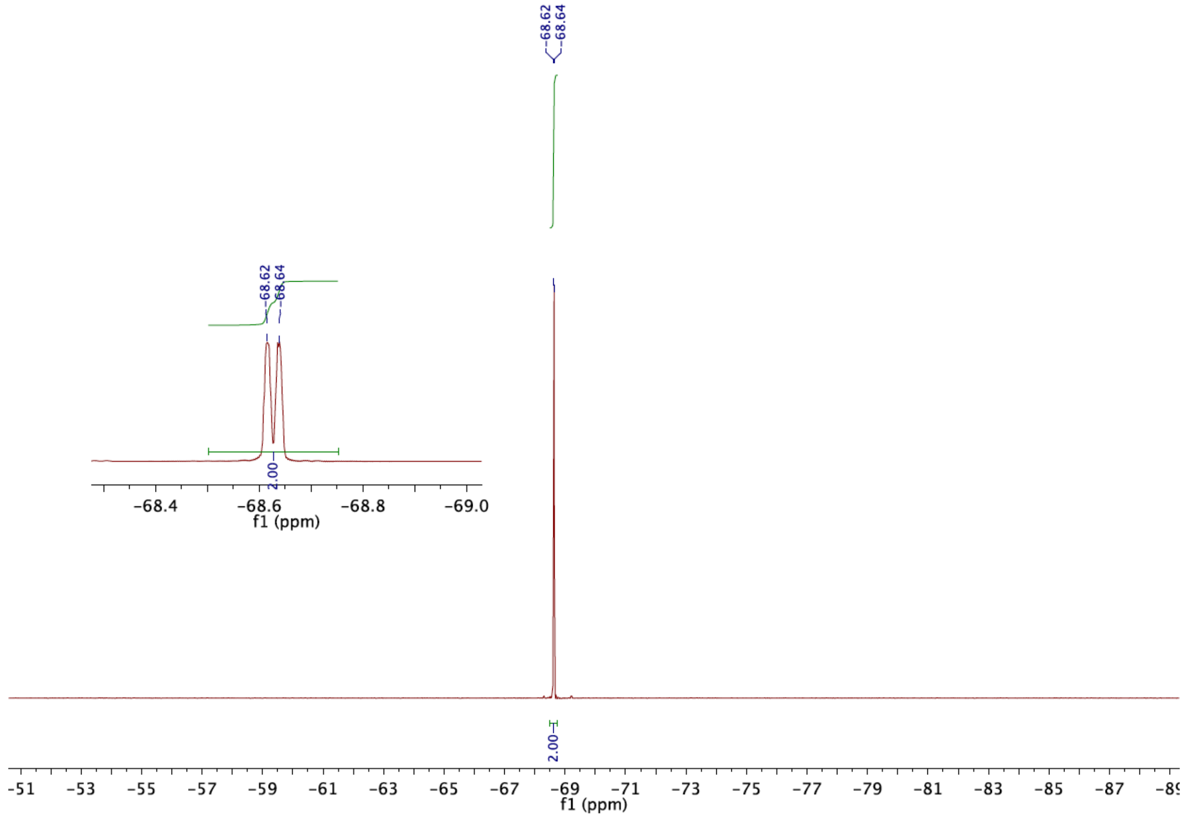

Supplementary Figure 41. ^{19}F NMR spectra of compound 4

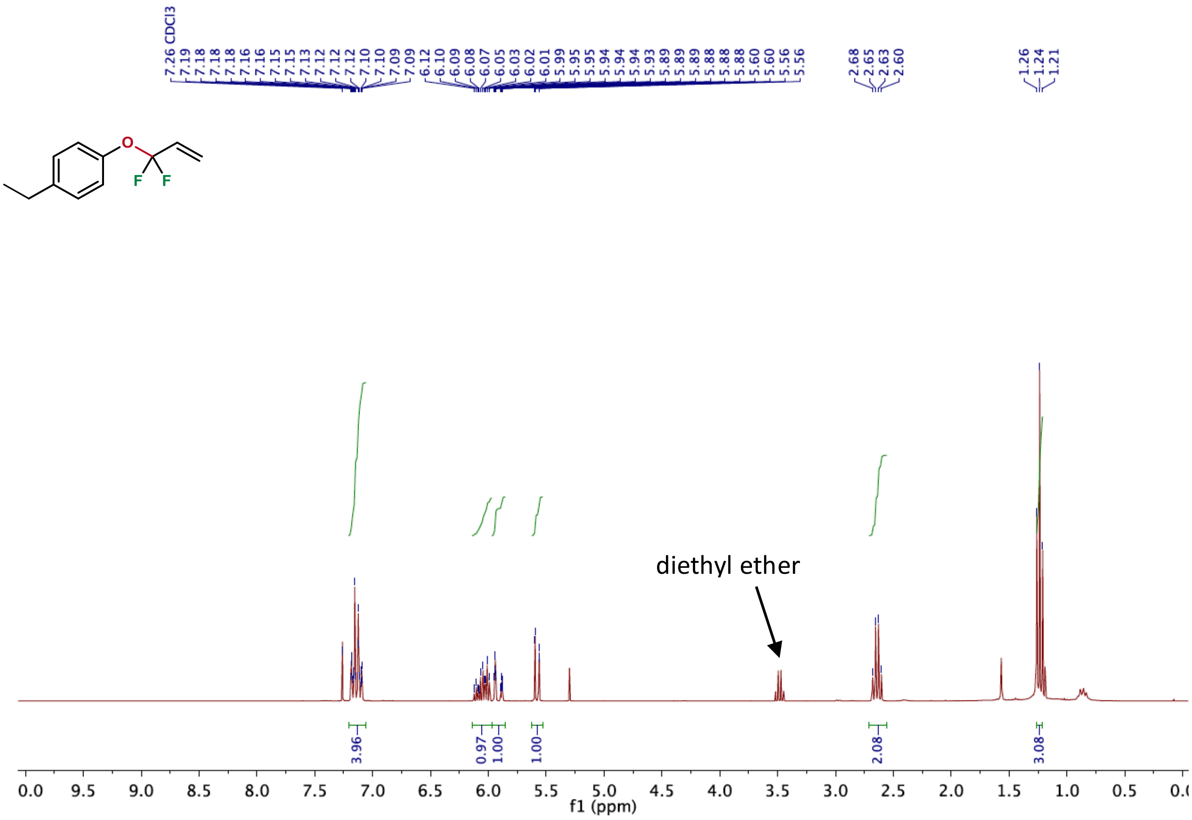

Supplementary Figure 42. ^{1}H NMR spectra of compound 5

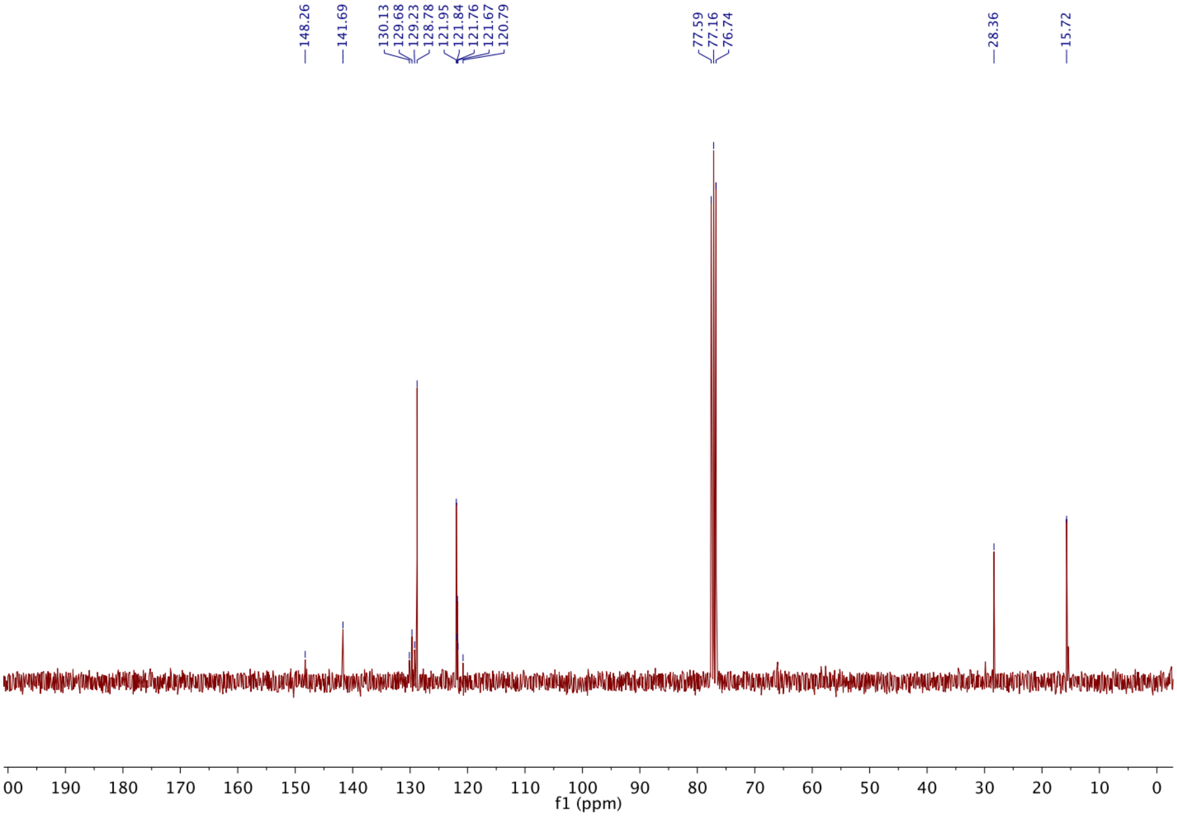

Supplementary Figure 43. ^{13}C NMR spectra of compound 5

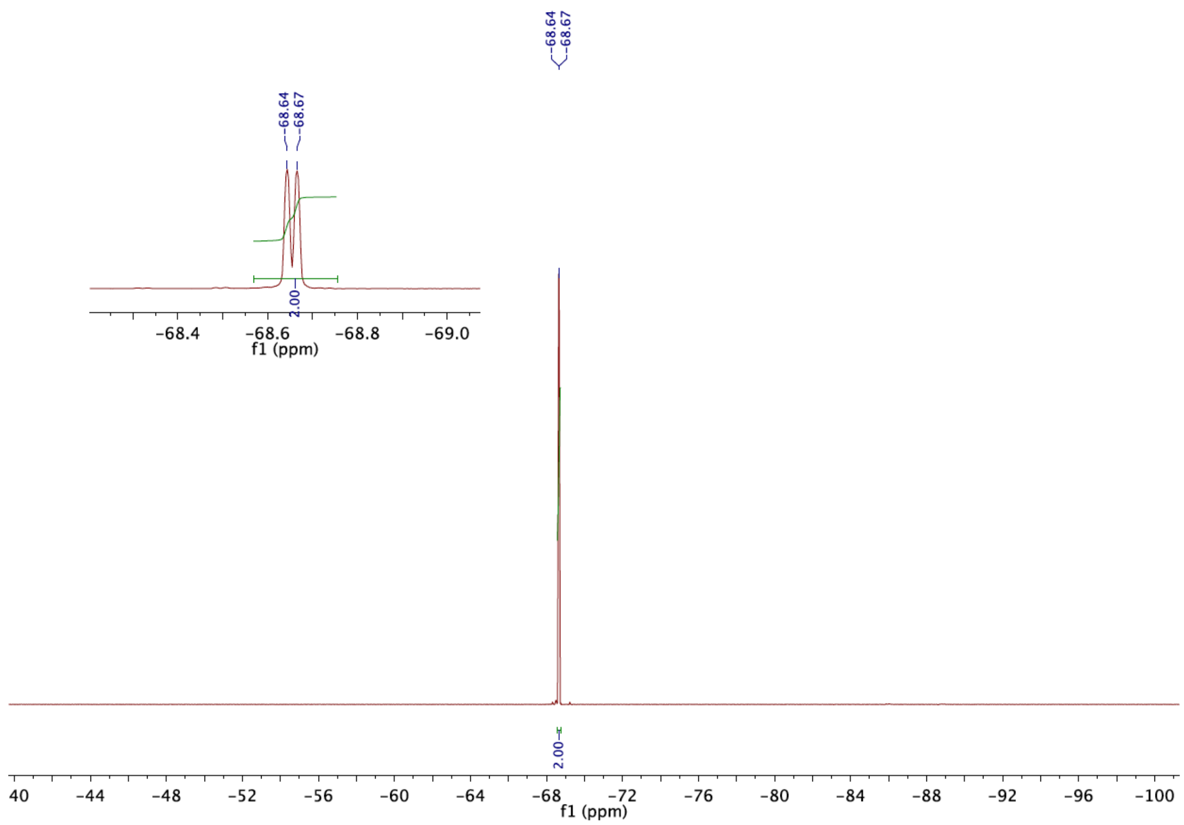

Supplementary Figure 44. ^{19}F NMR spectra of compound 5

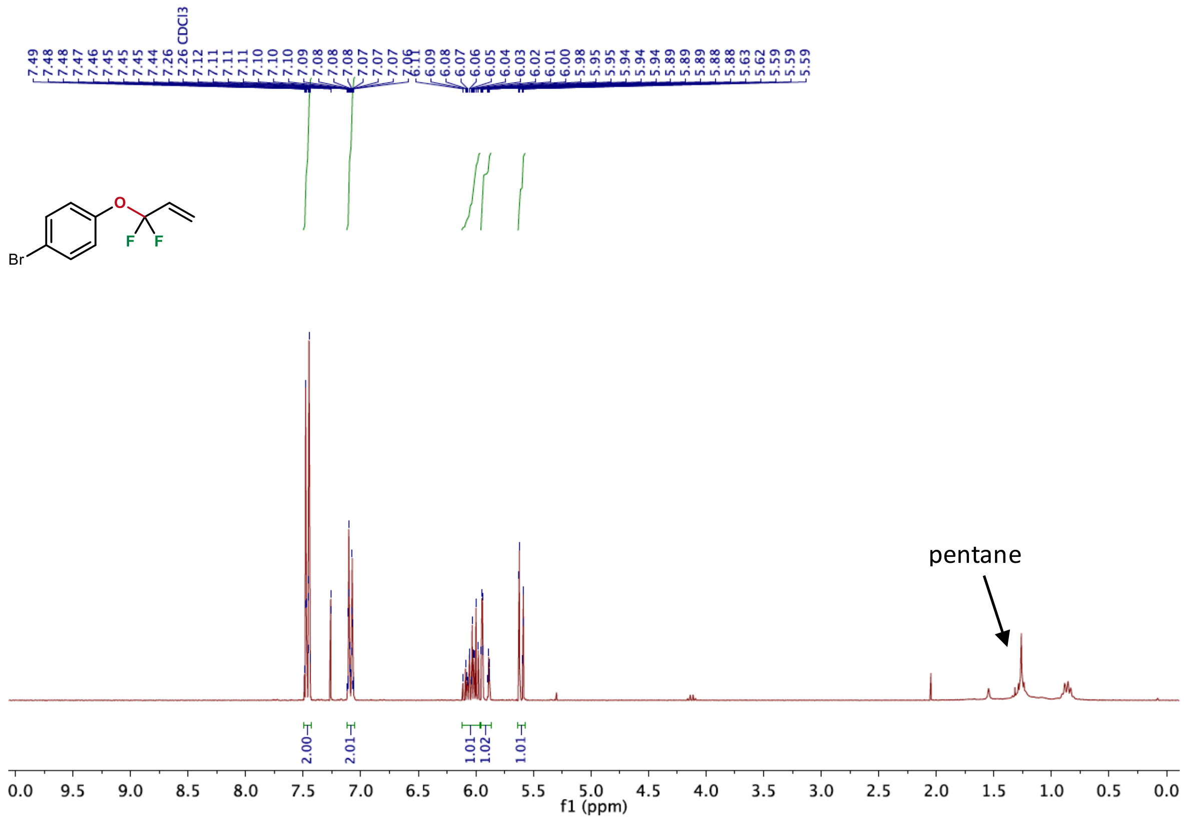

Supplementary Figure 45. ^{1}H NMR spectra of compound 6

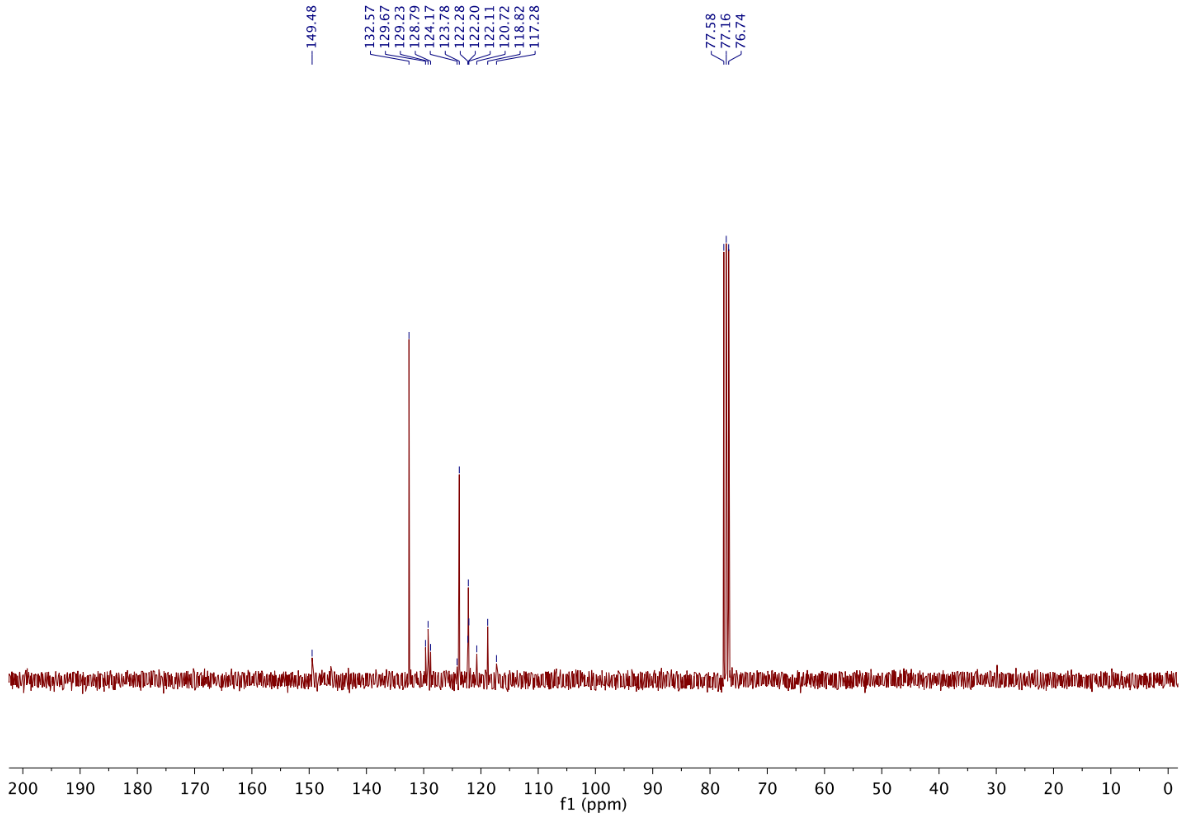

Supplementary Figure 46. ^{13}C NMR spectra of compound 6

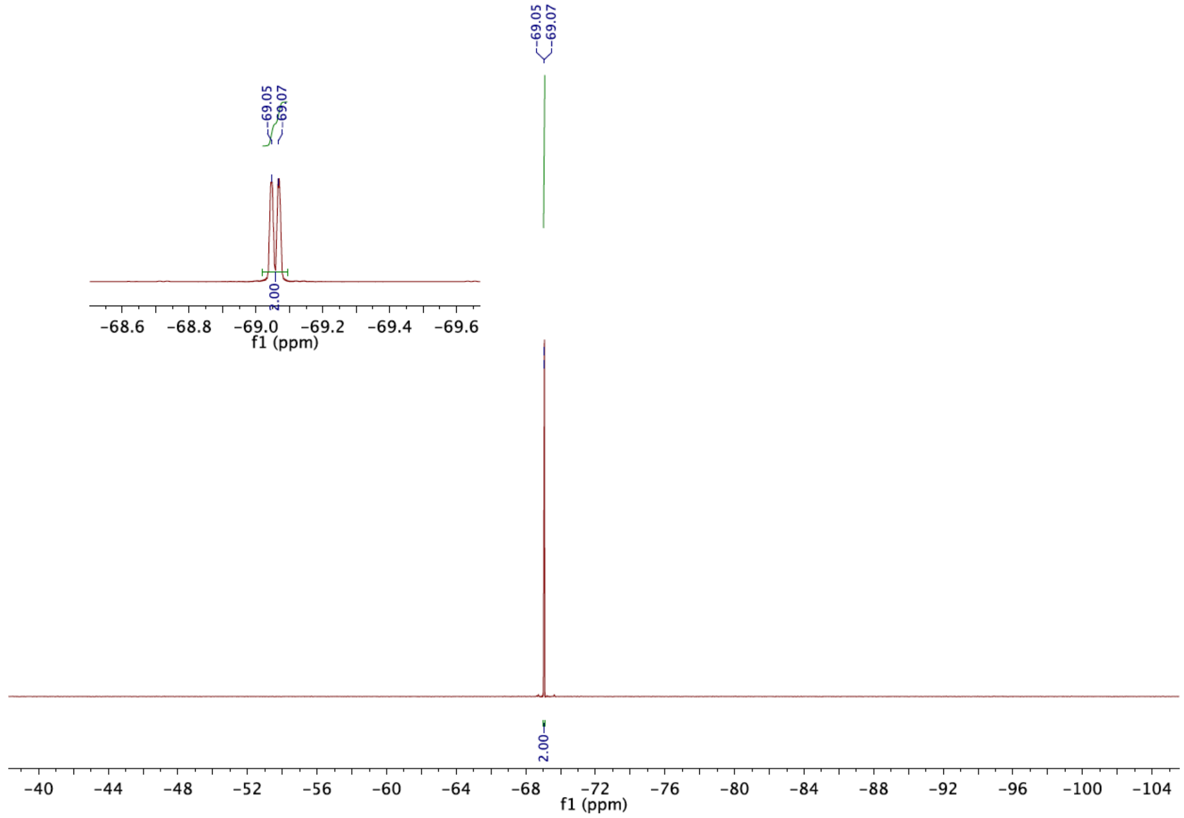

Supplementary Figure 47. ^{19}F NMR spectra of compound 6

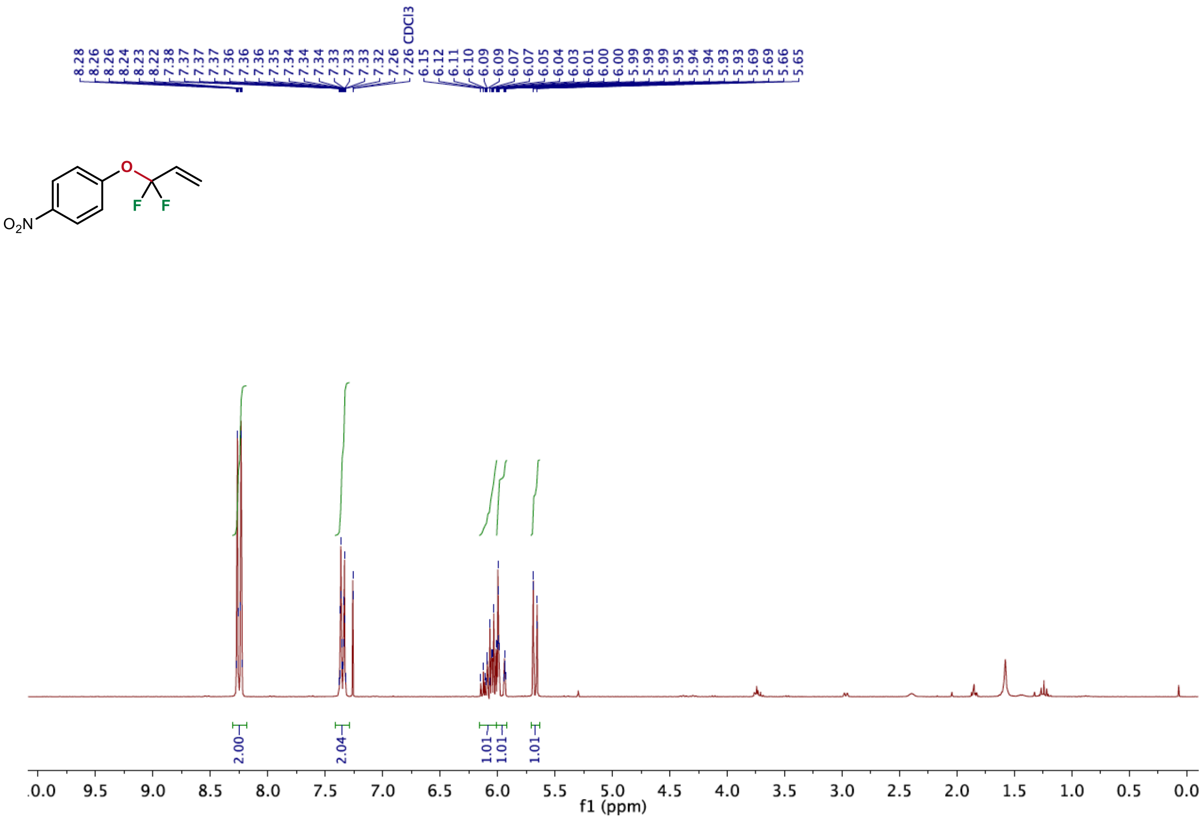

Supplementary Figure 48. ^{1}H NMR spectra of compound 7

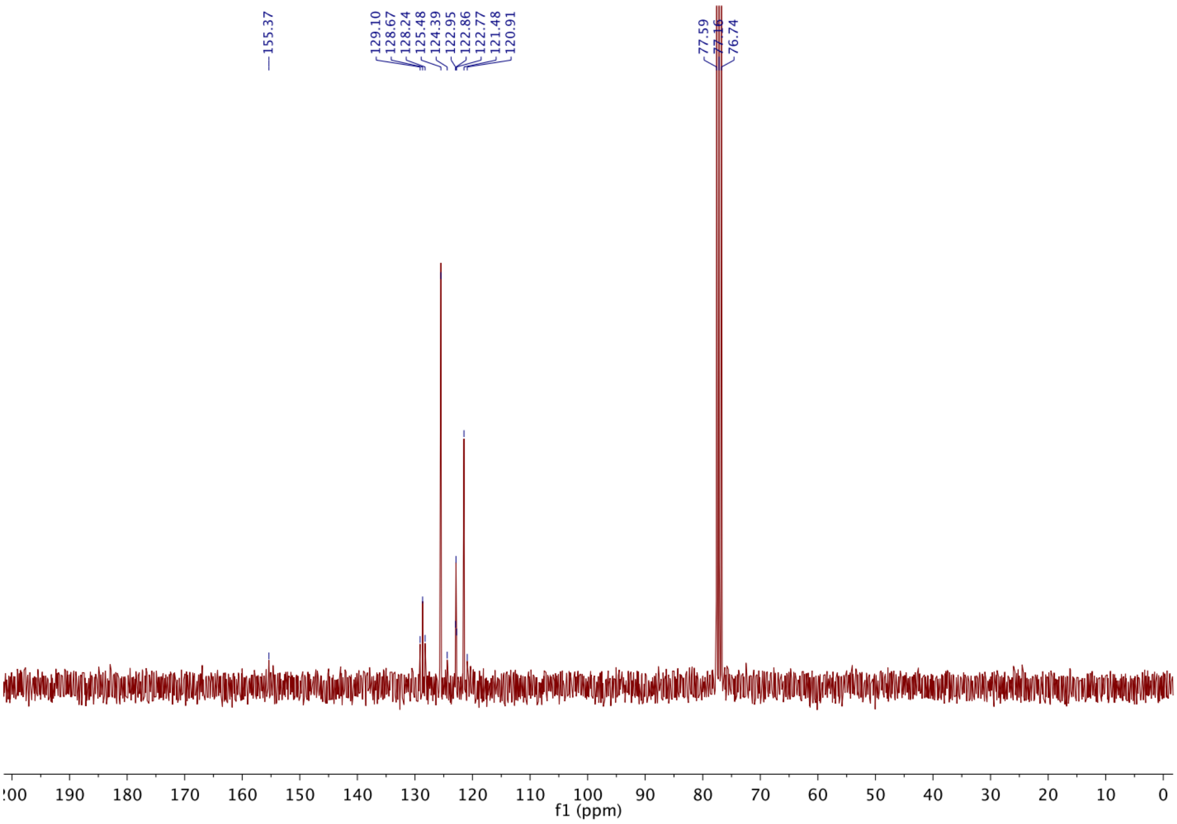

Supplementary Figure 48. ^{13}C NMR spectra of compound 7

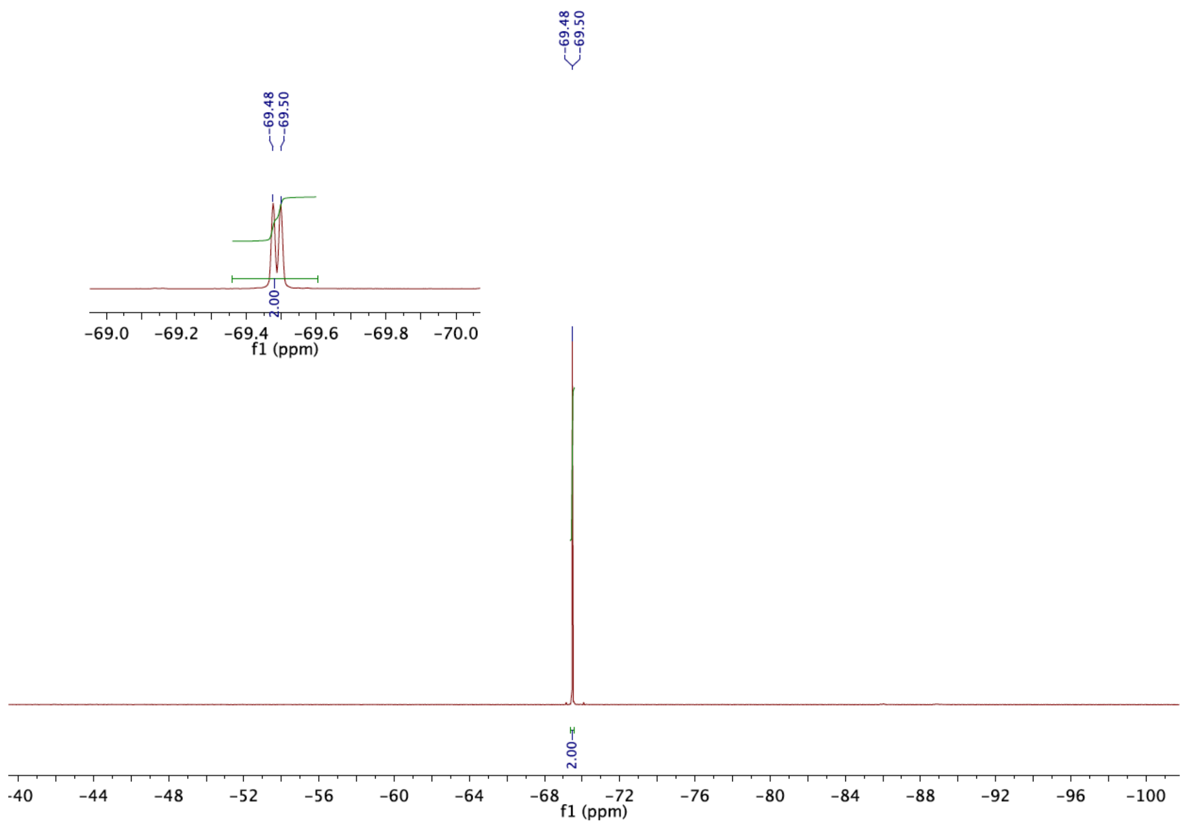

Supplementary Figure 49. ^{19}F NMR spectra of compound 7

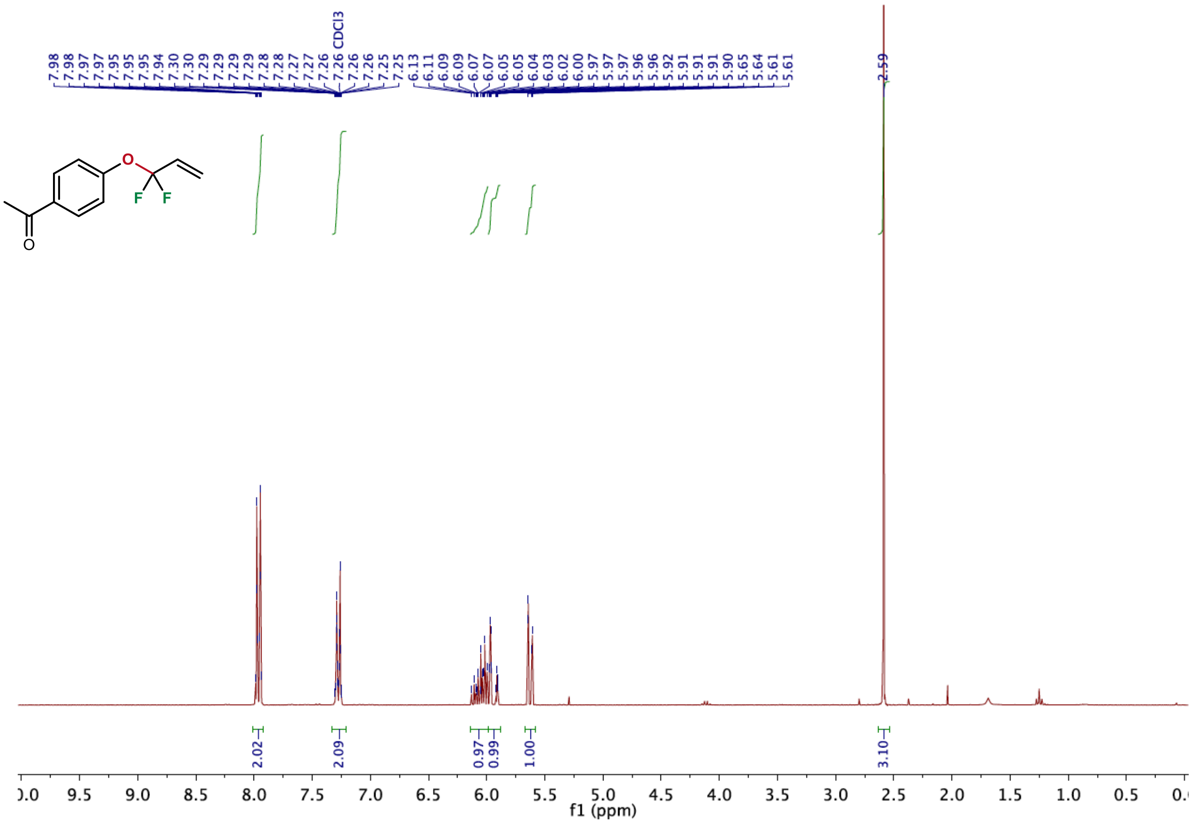

Supplementary Figure 50. ^{1}H NMR spectra of compound 8

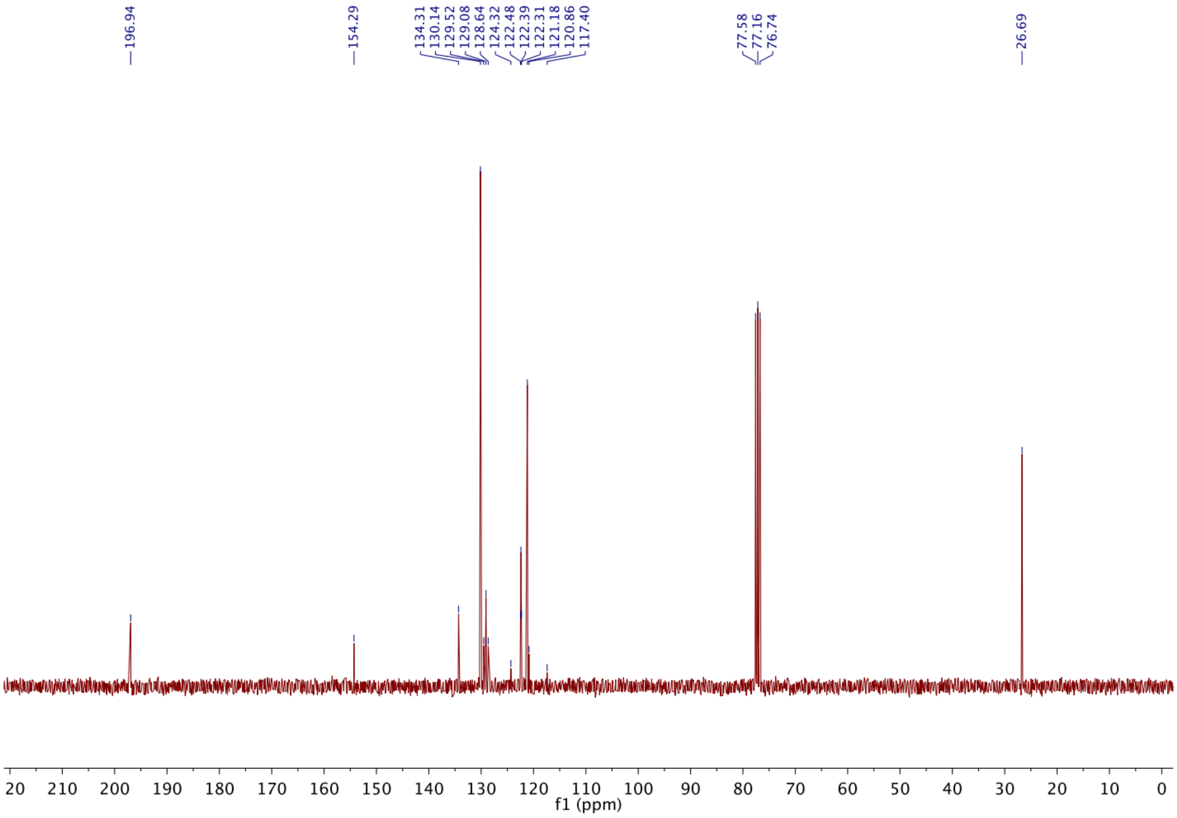

Supplementary Figure 51. ^{13}C NMR spectra of compound 8

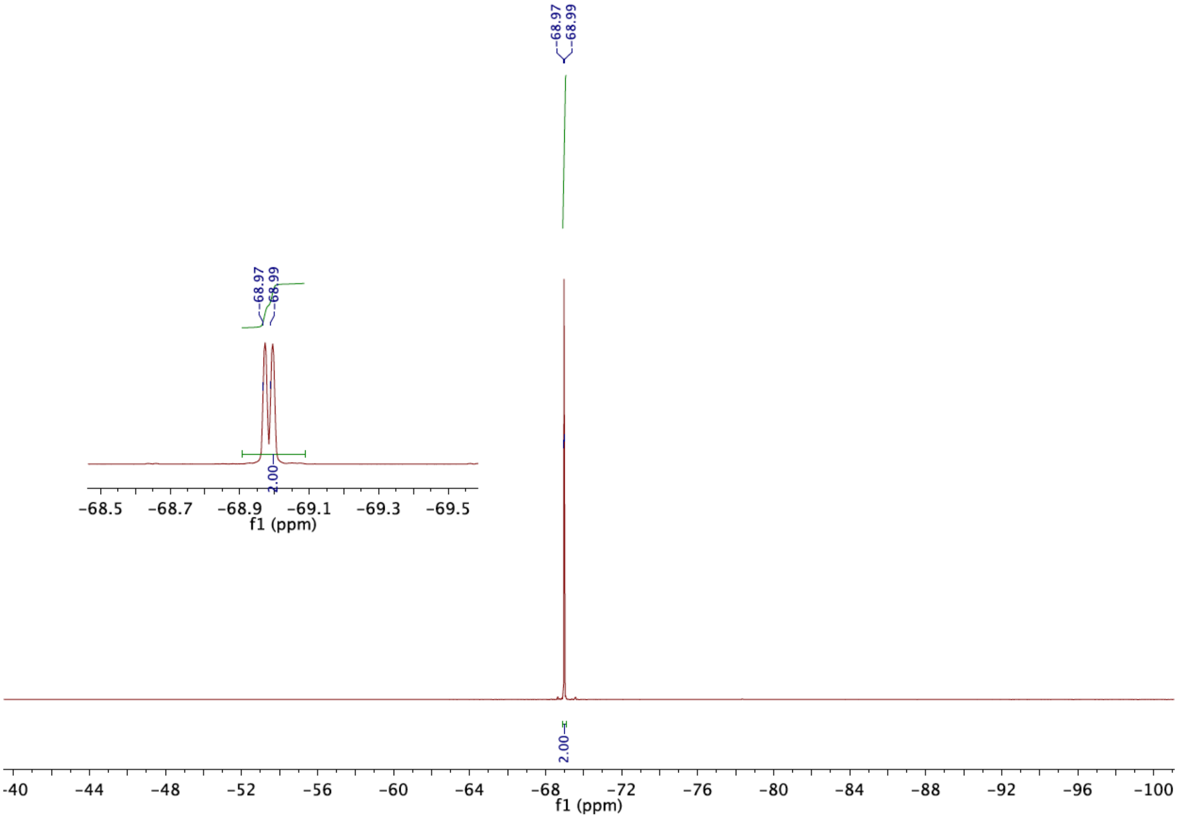

Supplementary Figure 52. ^{19}F NMR spectra of compound 8

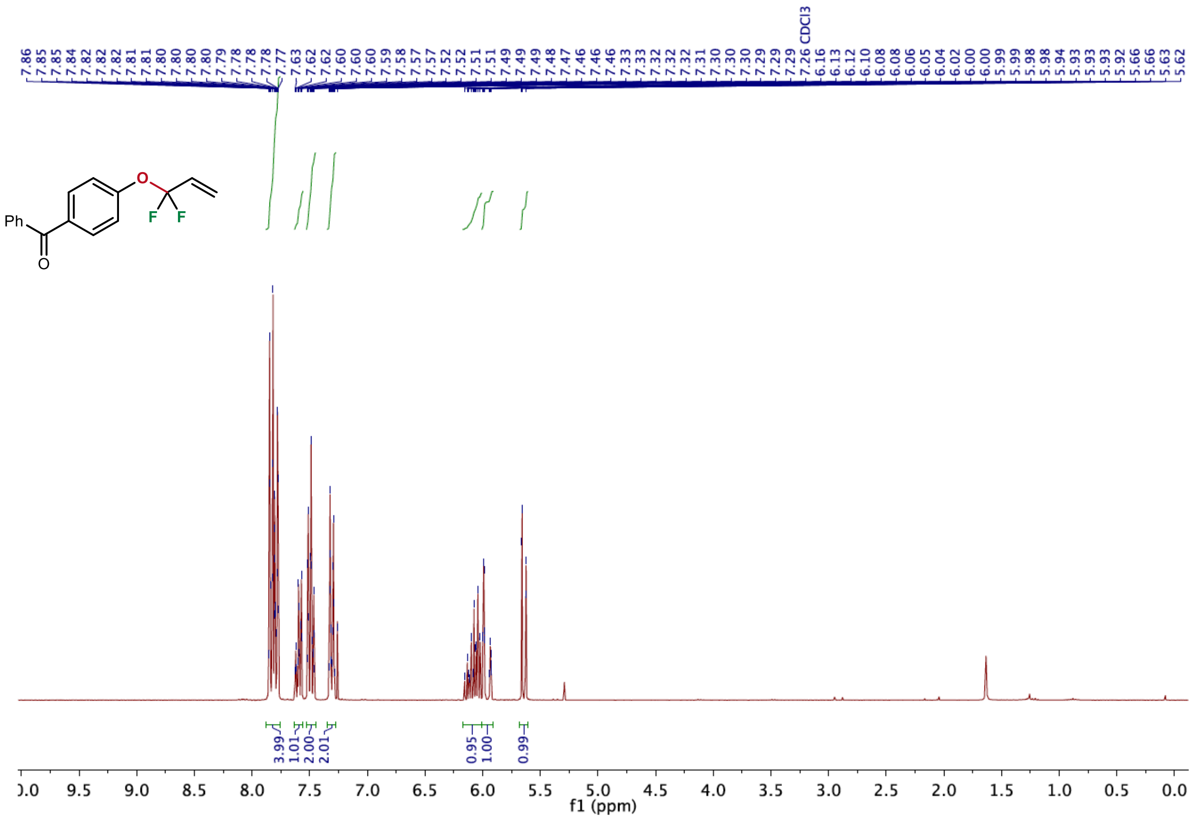

Supplementary Figure 53. ^{1}H NMR spectra of compound 9

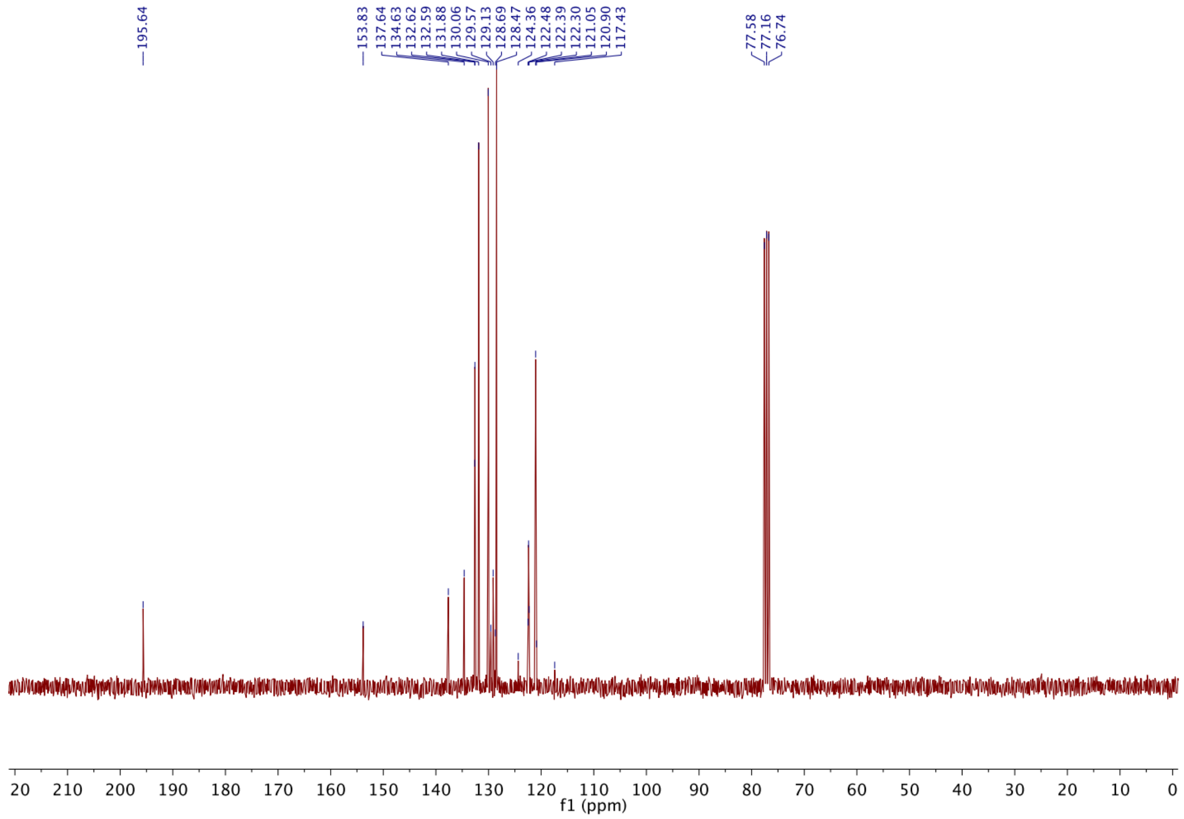

Supplementary Figure 54. ^{13}C NMR spectra of compound 9

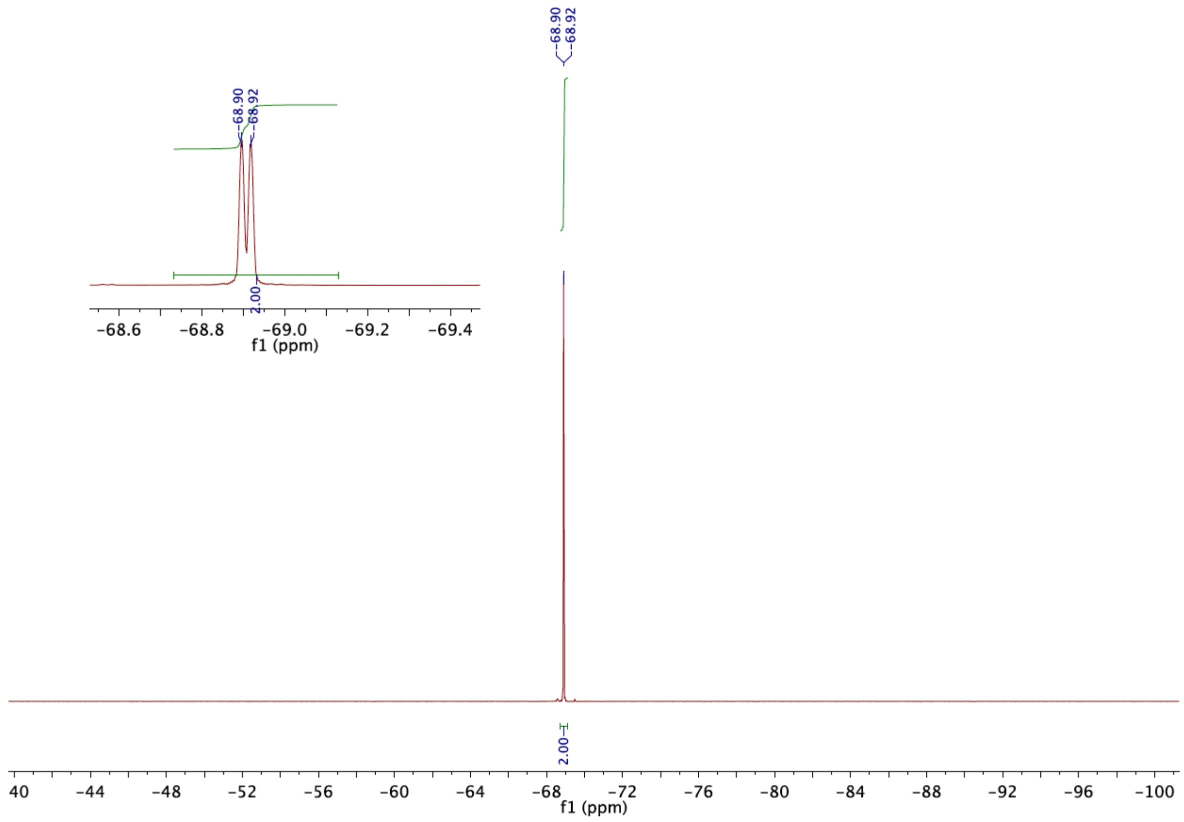

Supplementary Figure 55. ^{19}**F NMR spectra of compound 9**

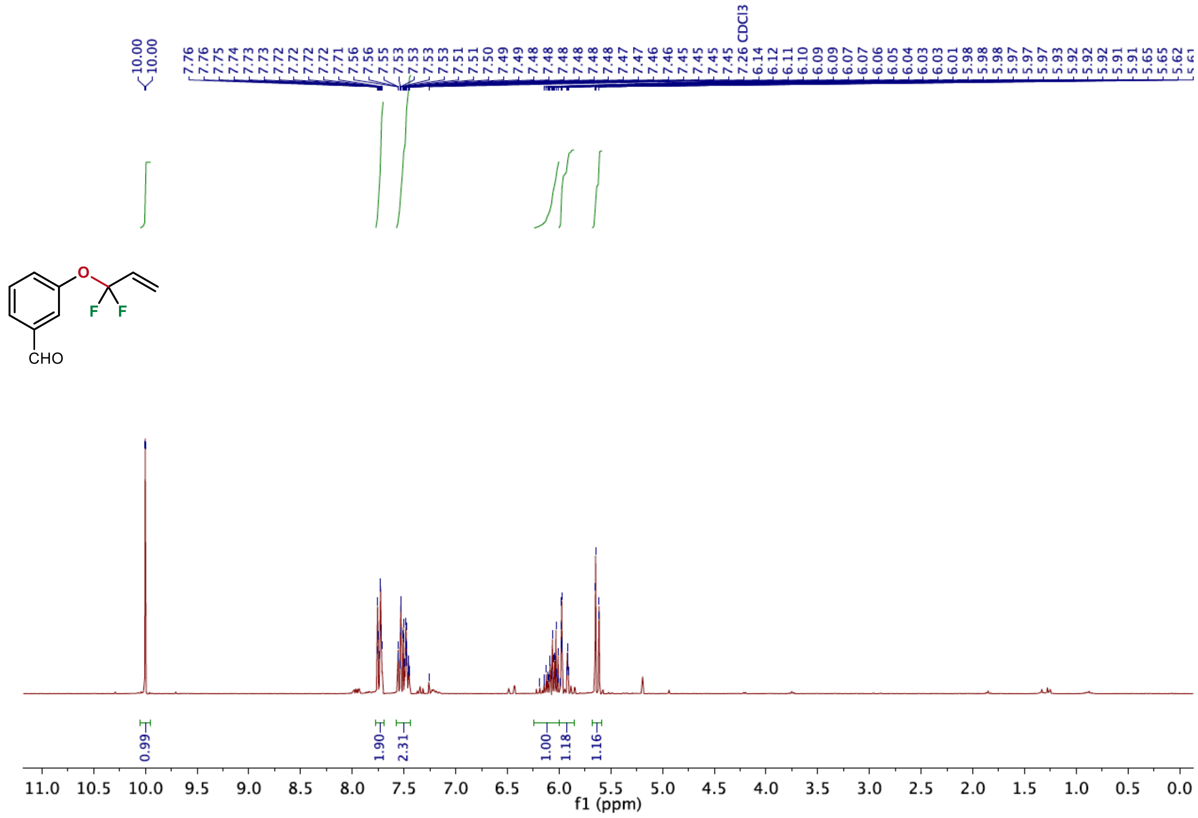

Supplementary Figure 56. ^{1}**H NMR spectra of compound 10**

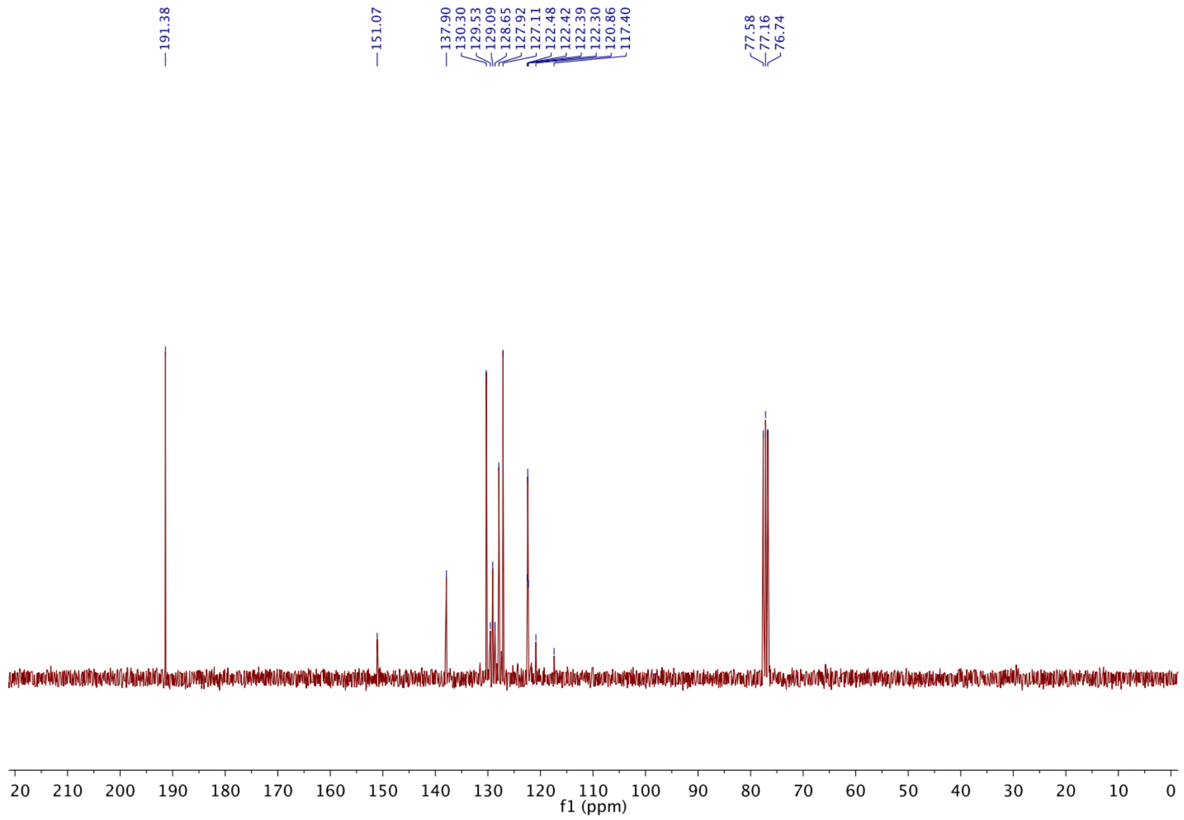

Supplementary Figure 57. ^{13}C NMR spectra of compound 10

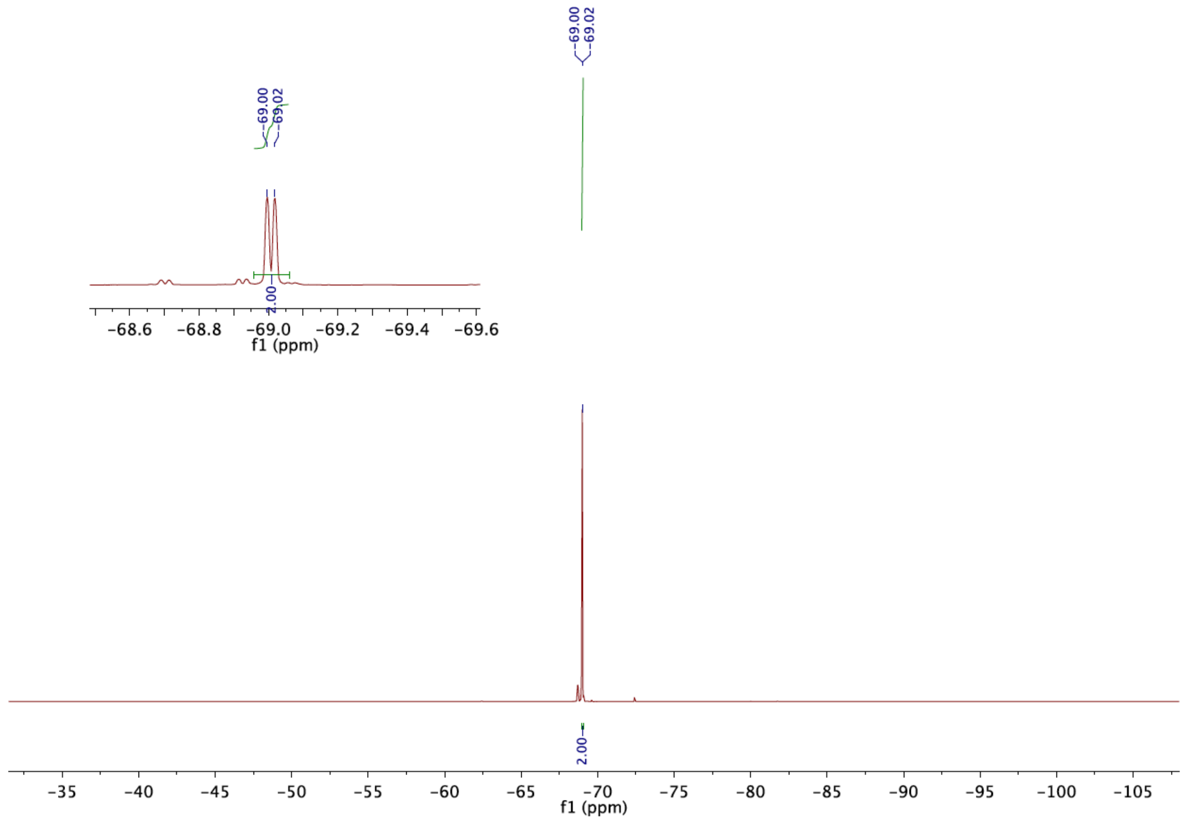

Supplementary Figure 58. ^{19}F NMR spectra of compound 10

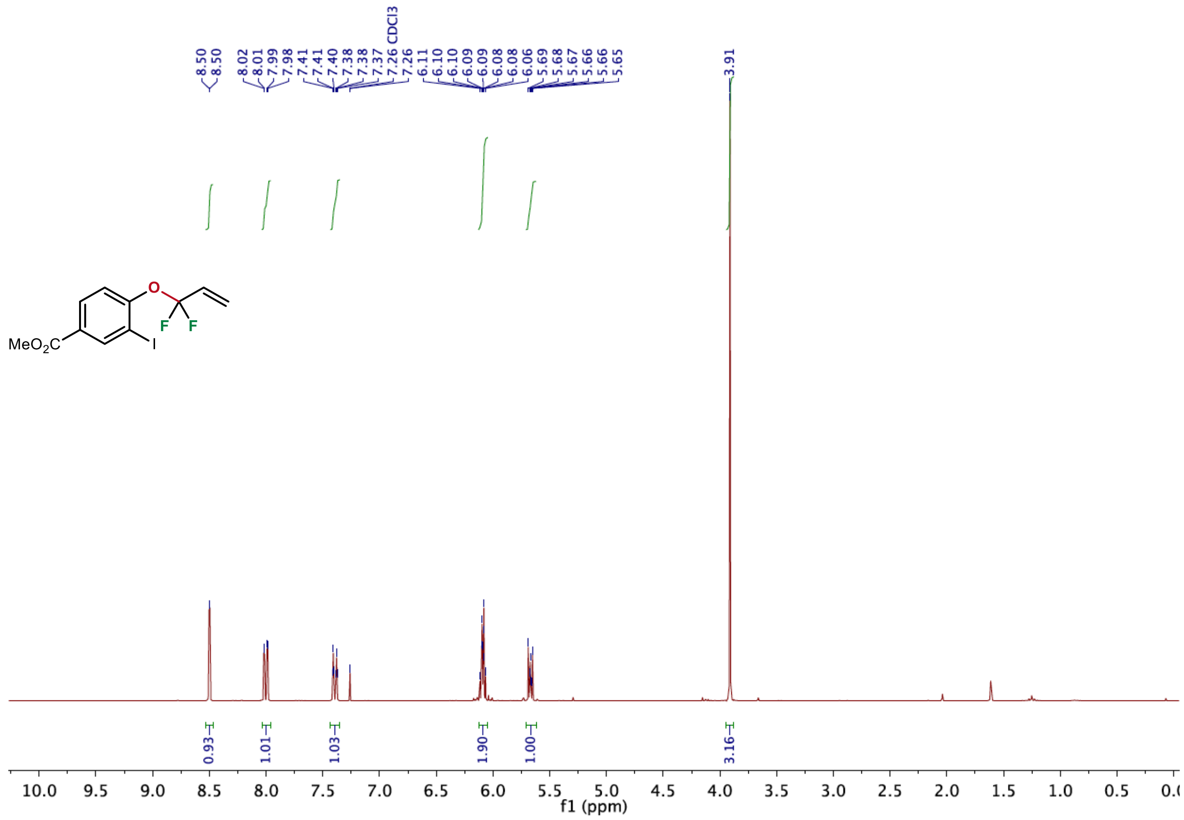

Supplementary Figure 59. ^{1}H NMR spectra of compound 11

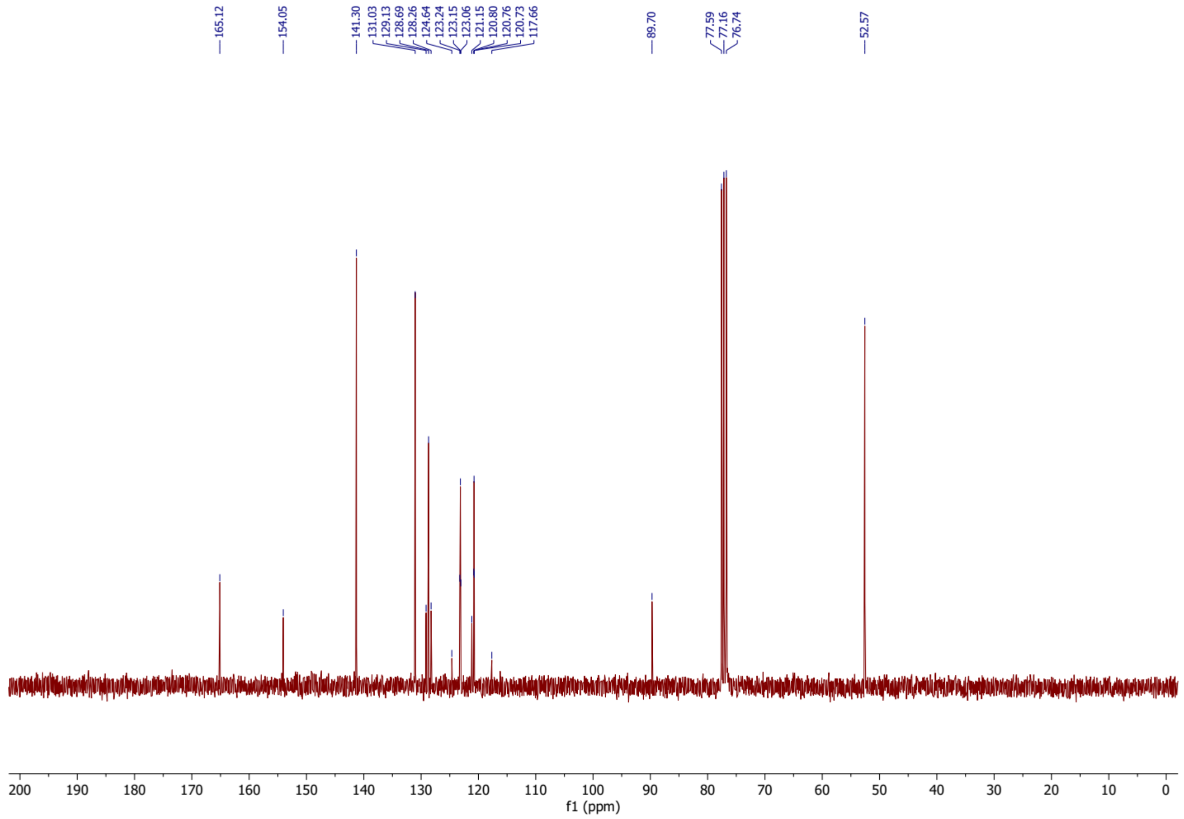

Supplementary Figure 60. ^{13}C NMR spectra of compound 11

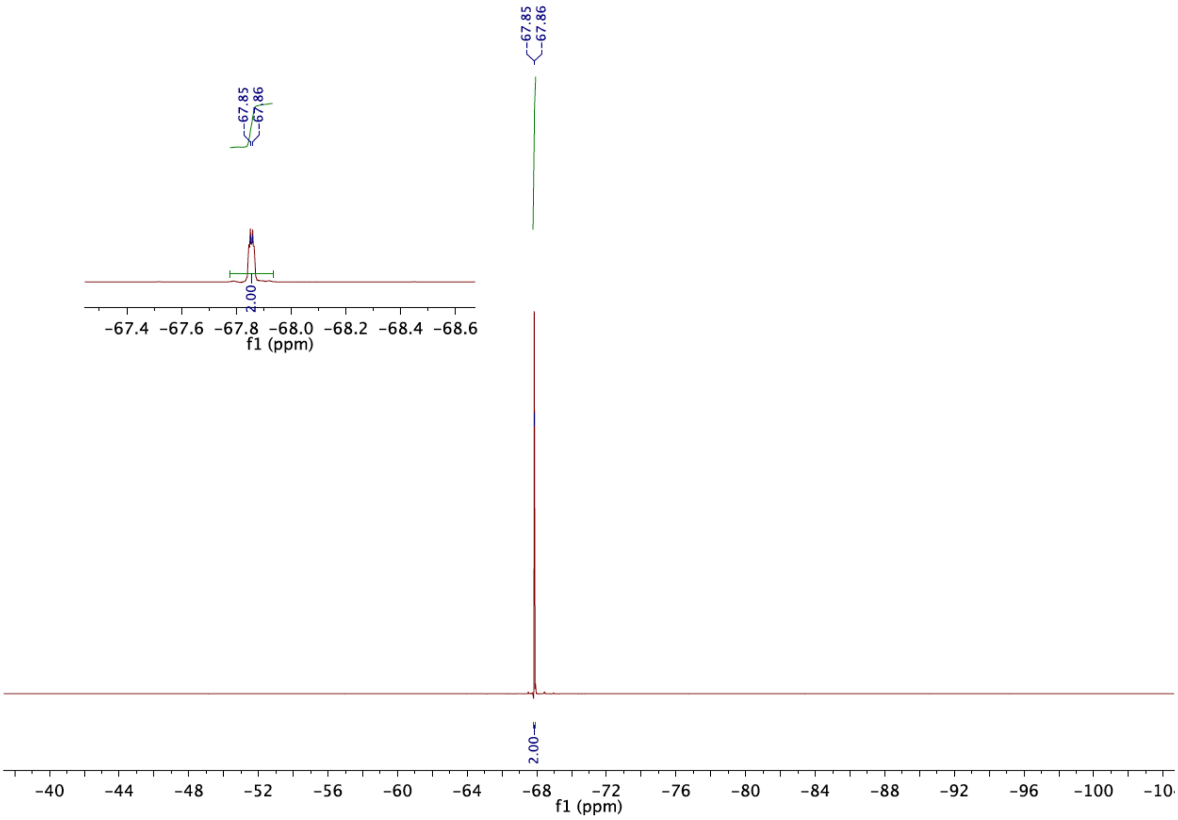

Supplementary Figure 61. ^{13}C NMR spectra of compound 11

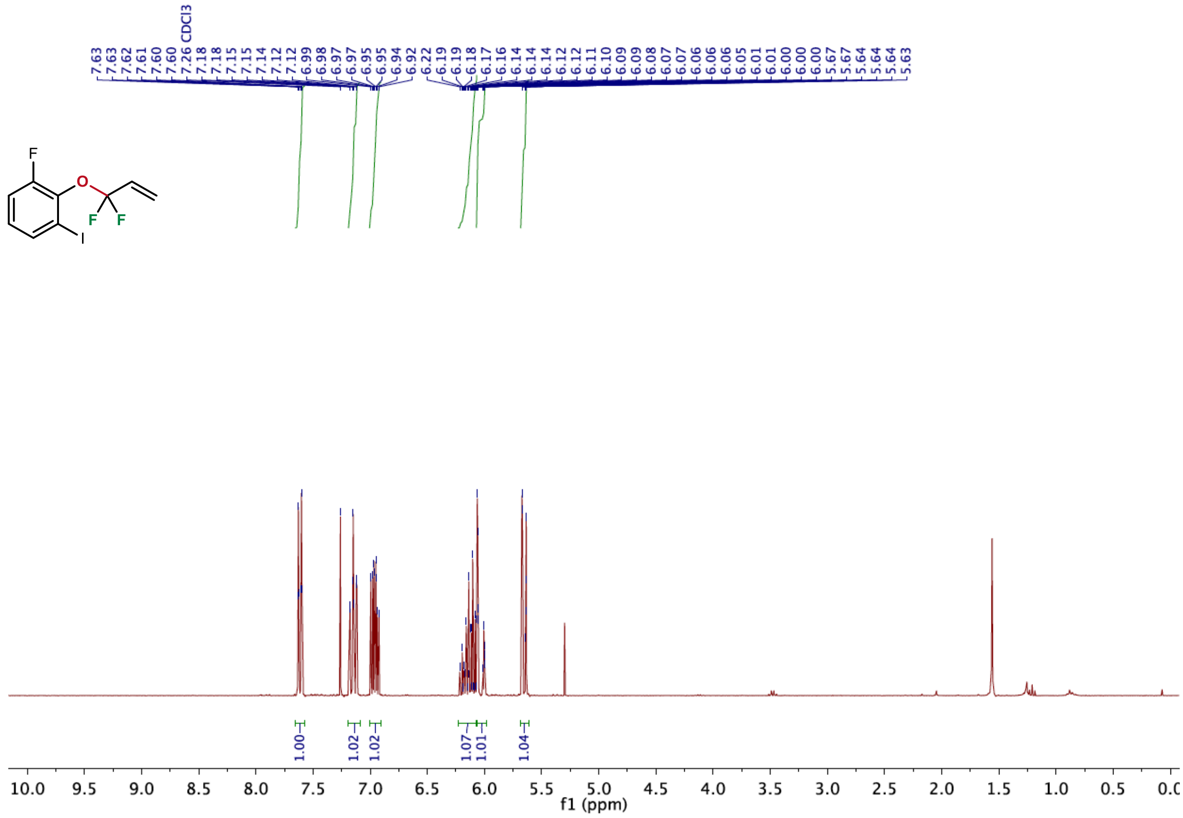

Supplementary Figure 62. ^{1}H NMR spectra of compound 12

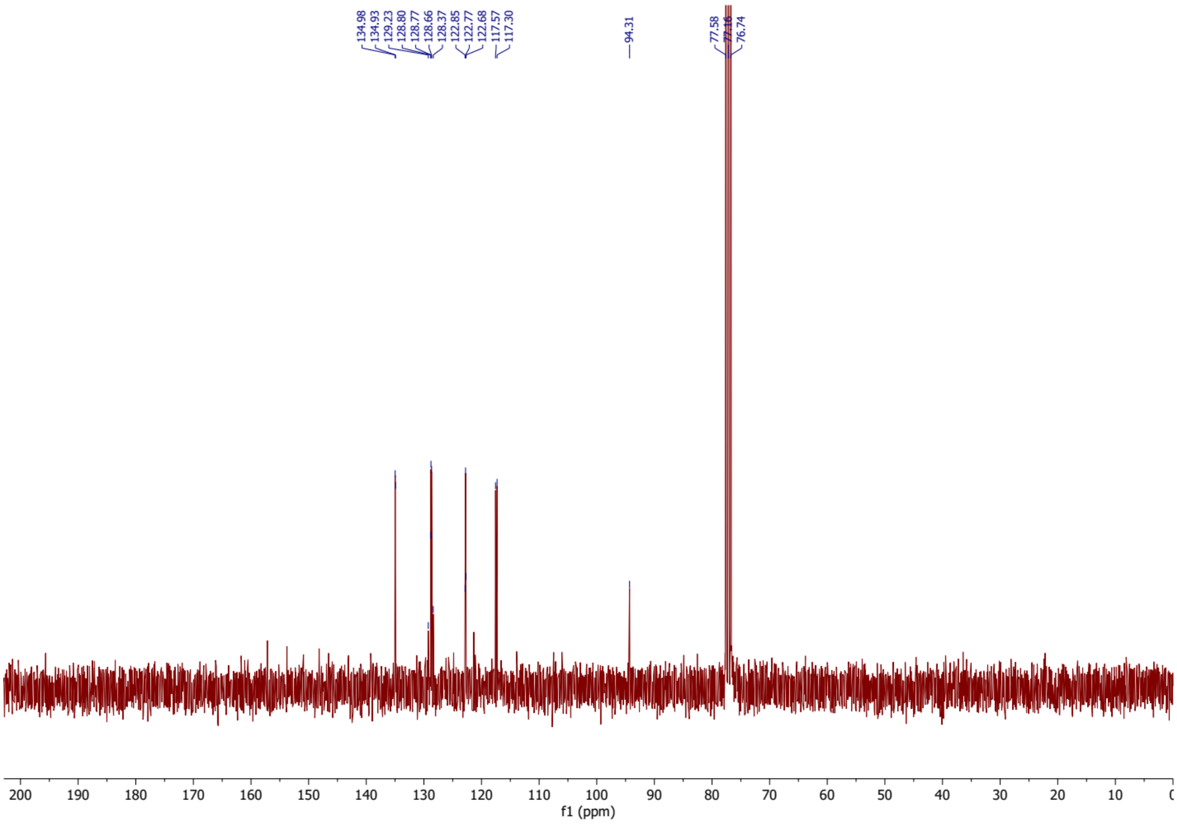

Supplementary Figure 63. ^{13}C NMR spectra of compound 12

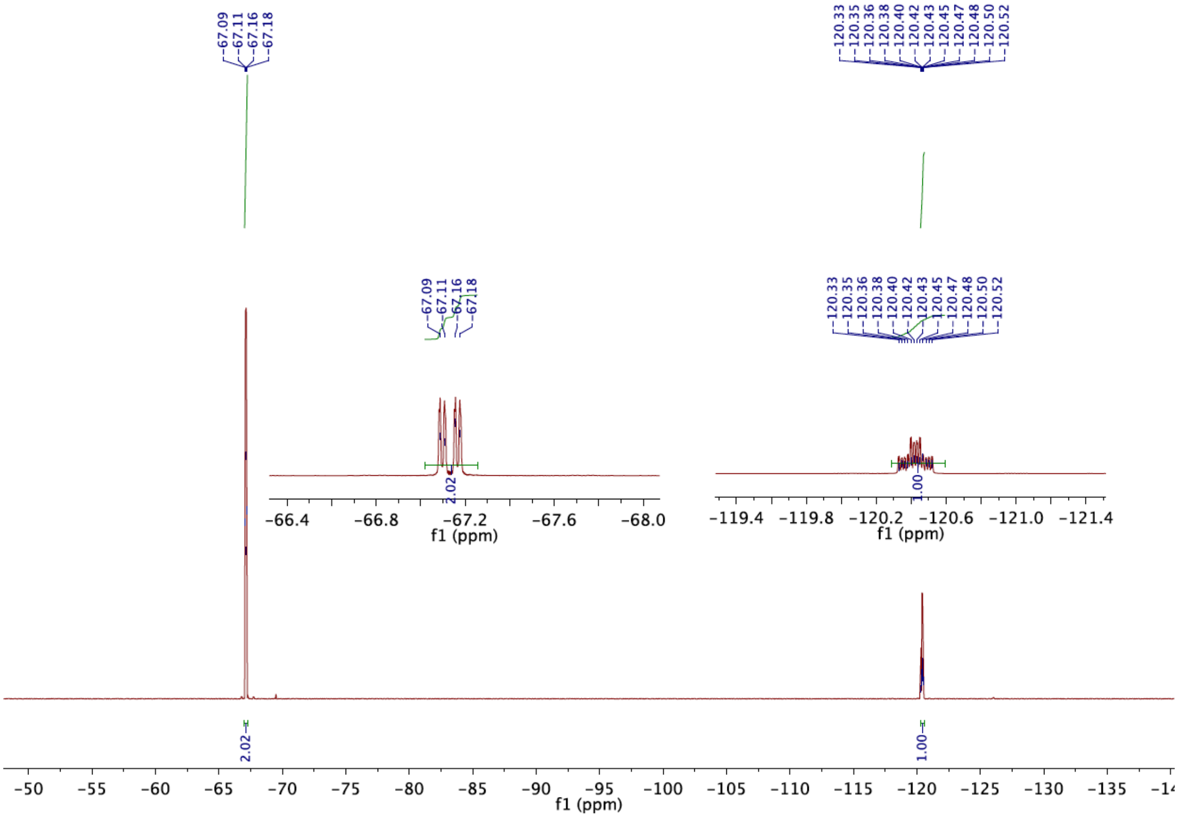

Supplementary Figure 64. ^{19}F NMR spectra of compound 12

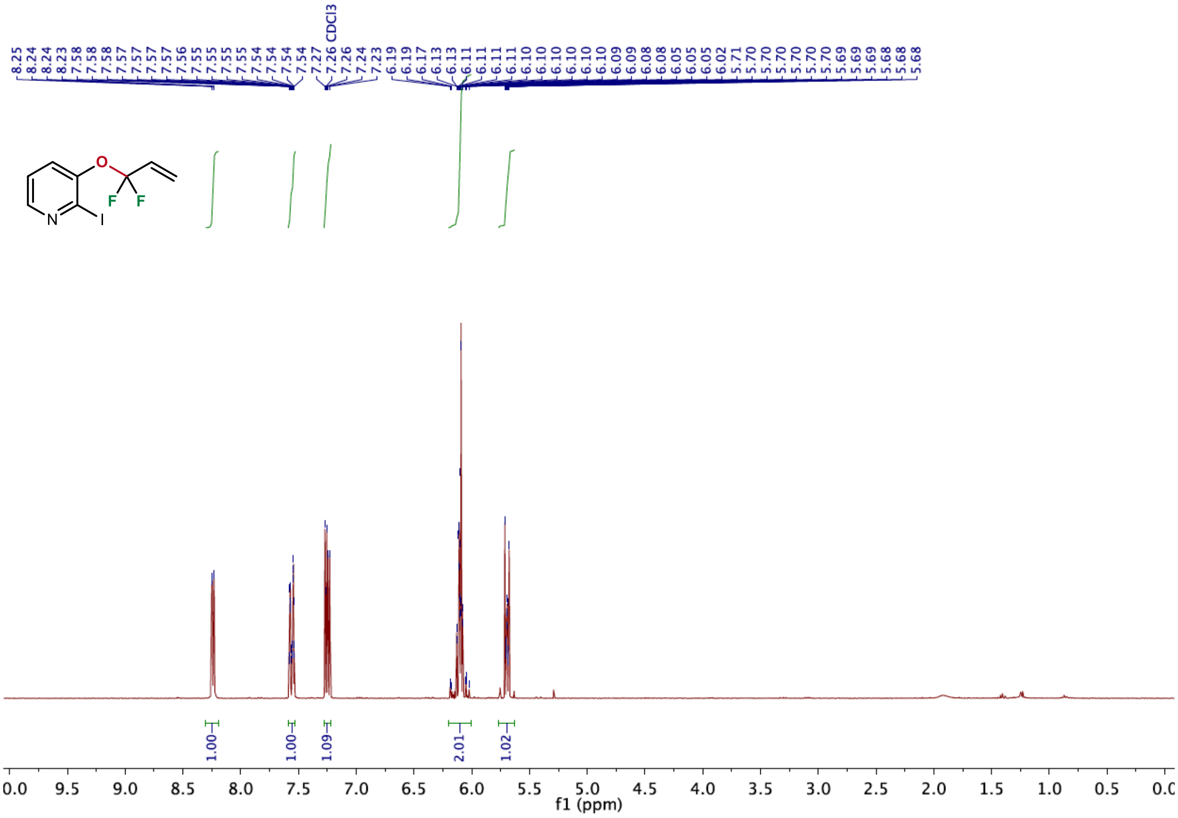

Supplementary Figure 65. ^{1}H NMR spectra of compound 13

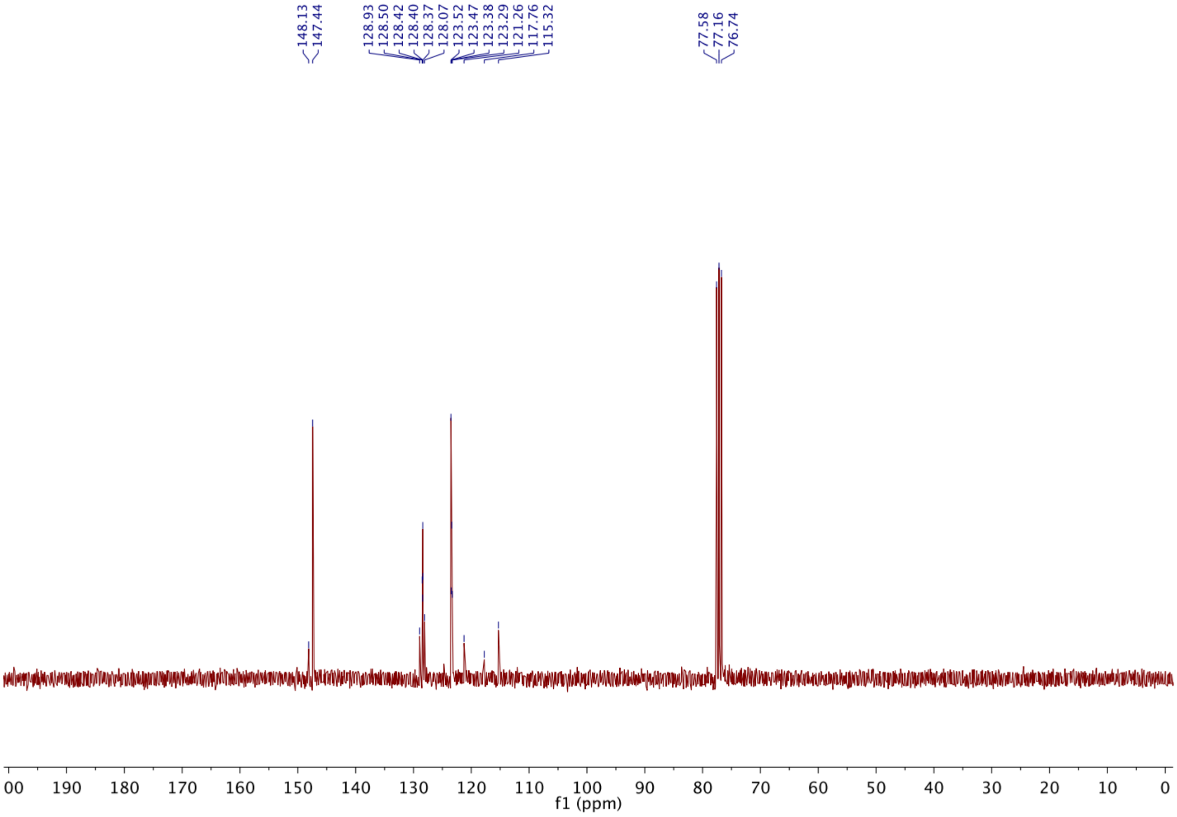

Supplementary Figure 66. ^{13}C NMR spectra of compound 13

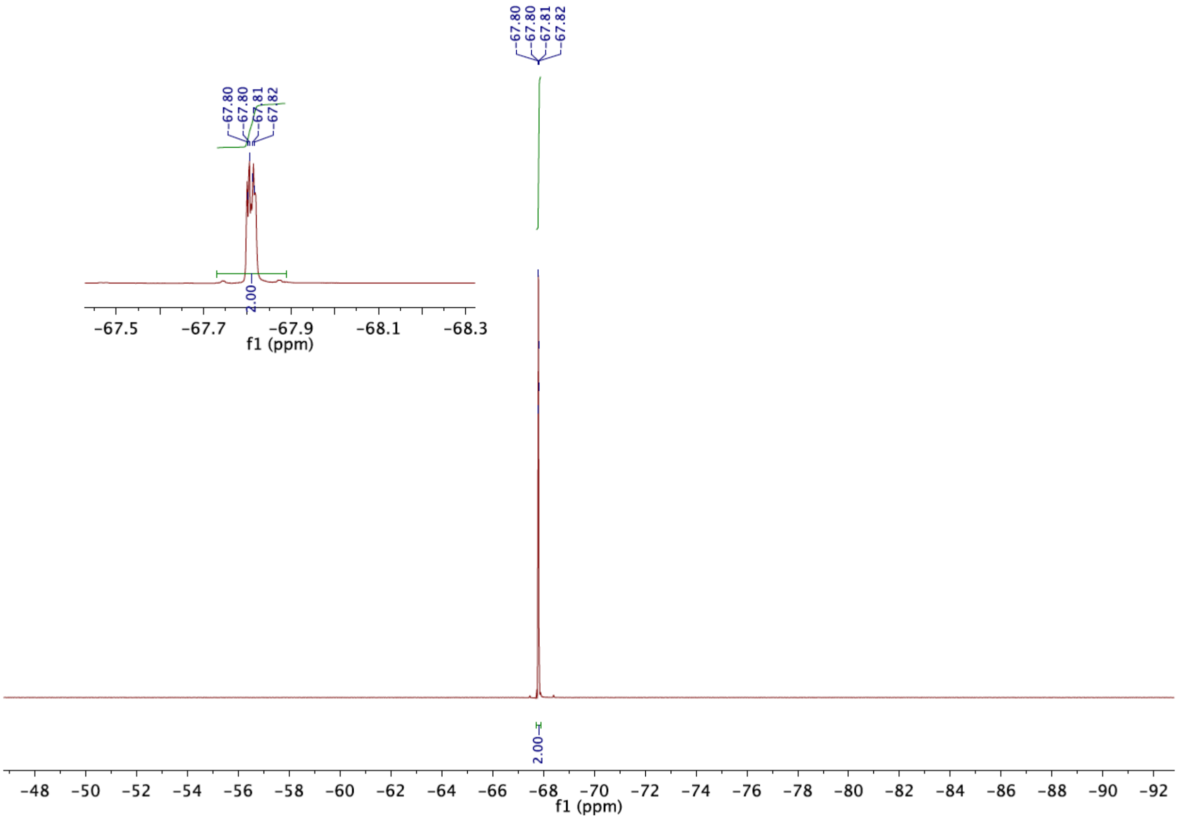

Supplementary Figure 67. ^{19}F NMR spectra of compound 13

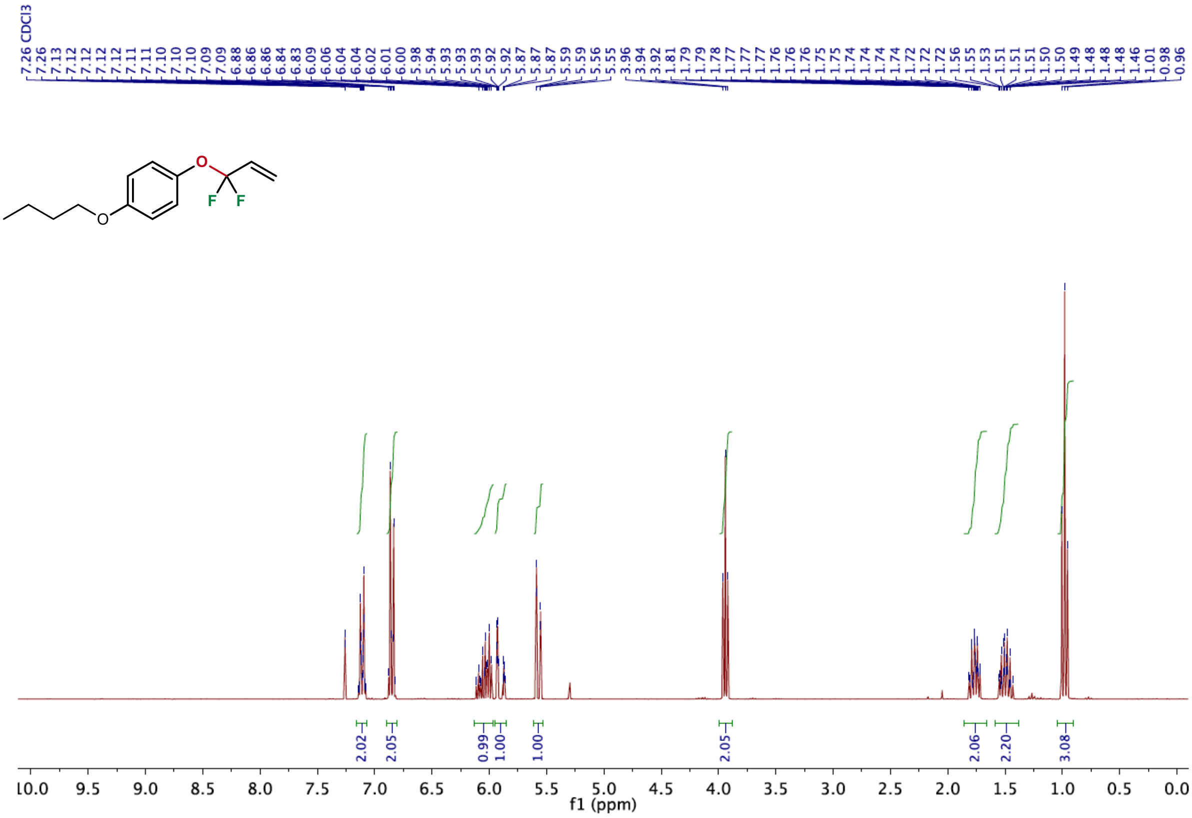

Supplementary Figure 68. ^{1}H NMR spectra of compound 14

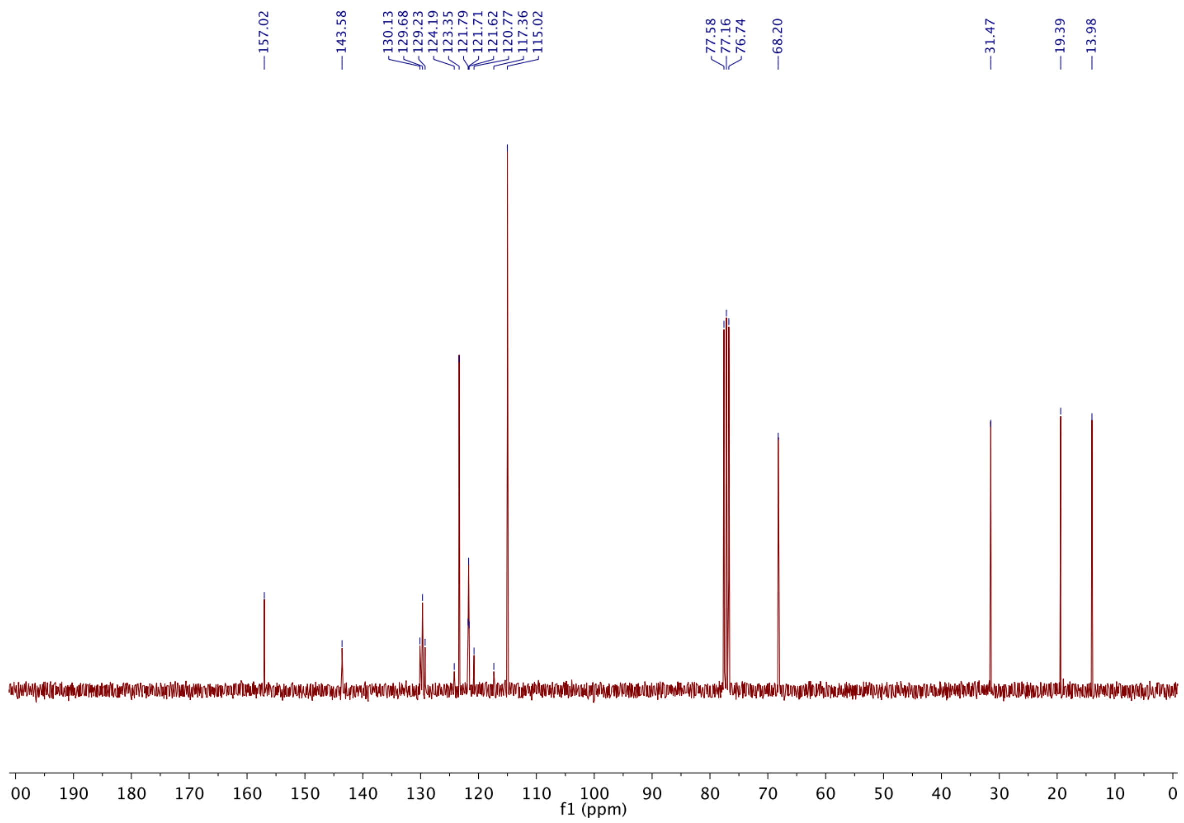

Supplementary Figure 69. ^{13}C NMR spectra of compound 14

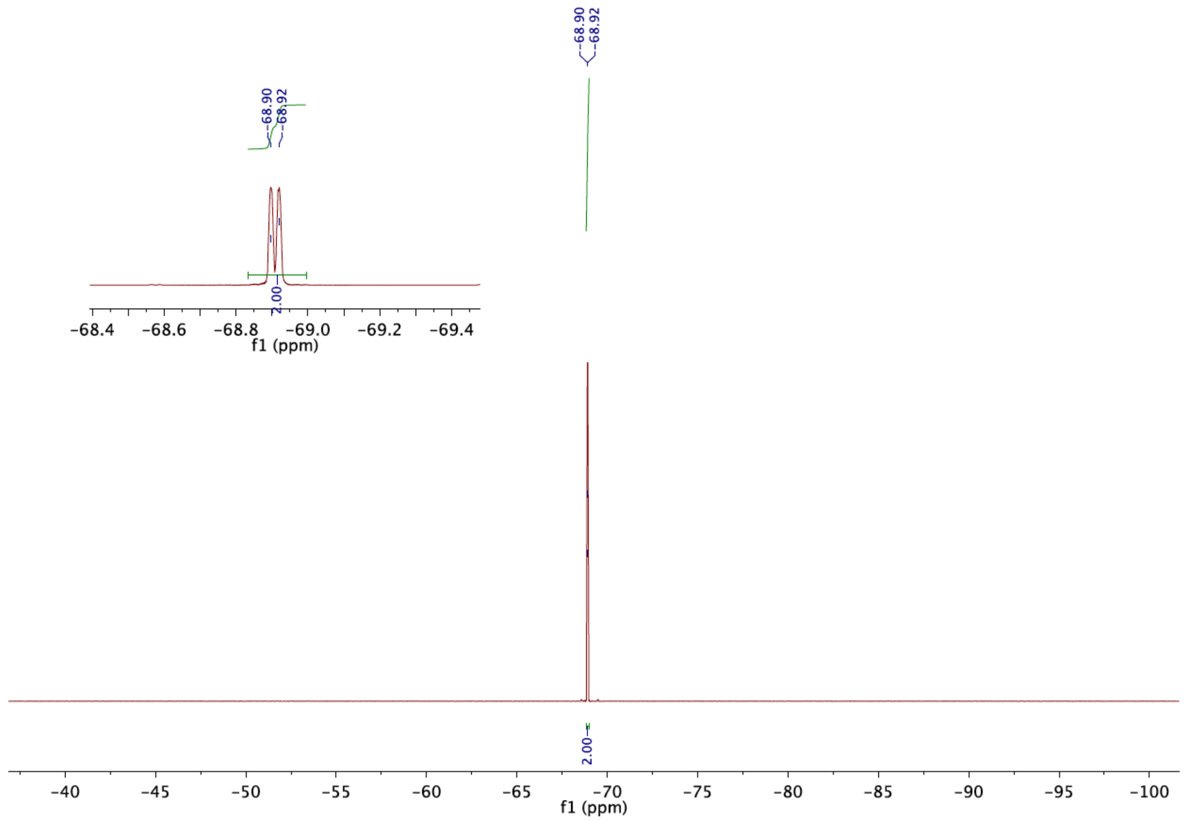

Supplementary Figure 70. ^{19}F NMR spectra of compound 14

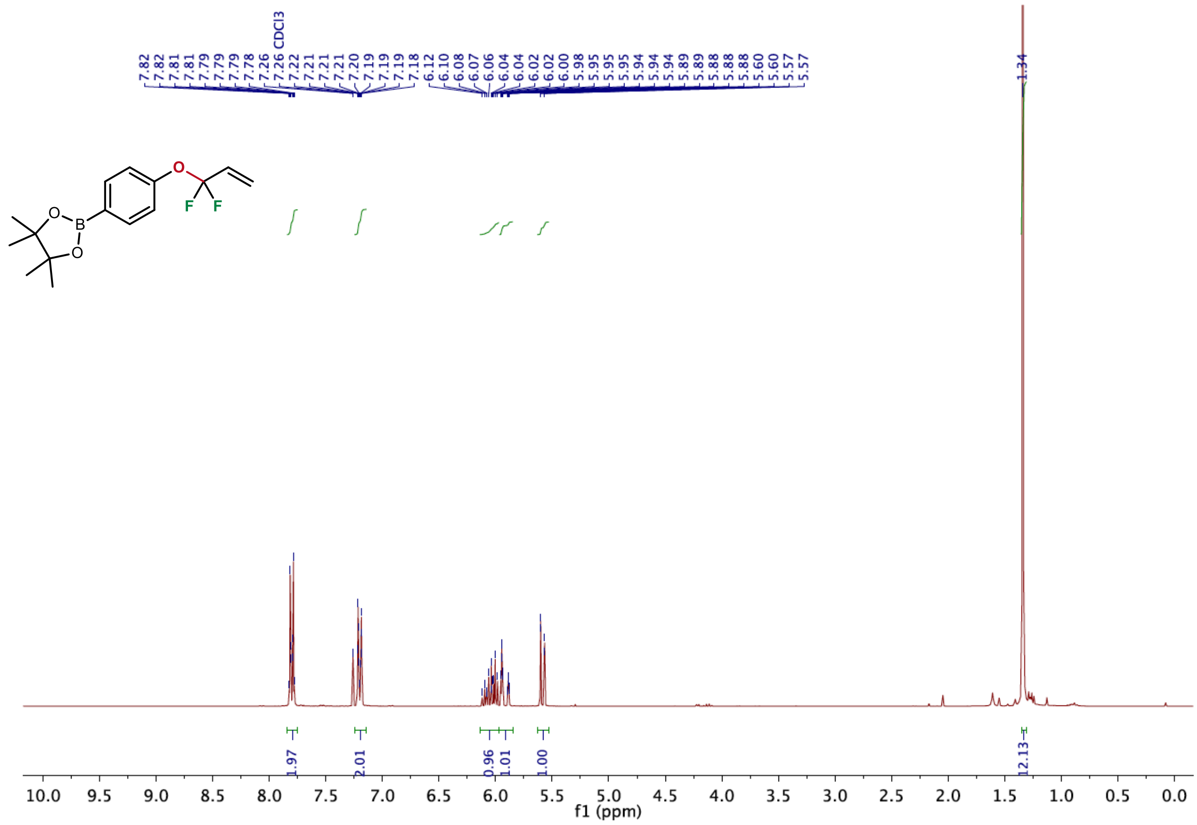

Supplementary Figure 71. ^{1}H NMR spectra of compound 15

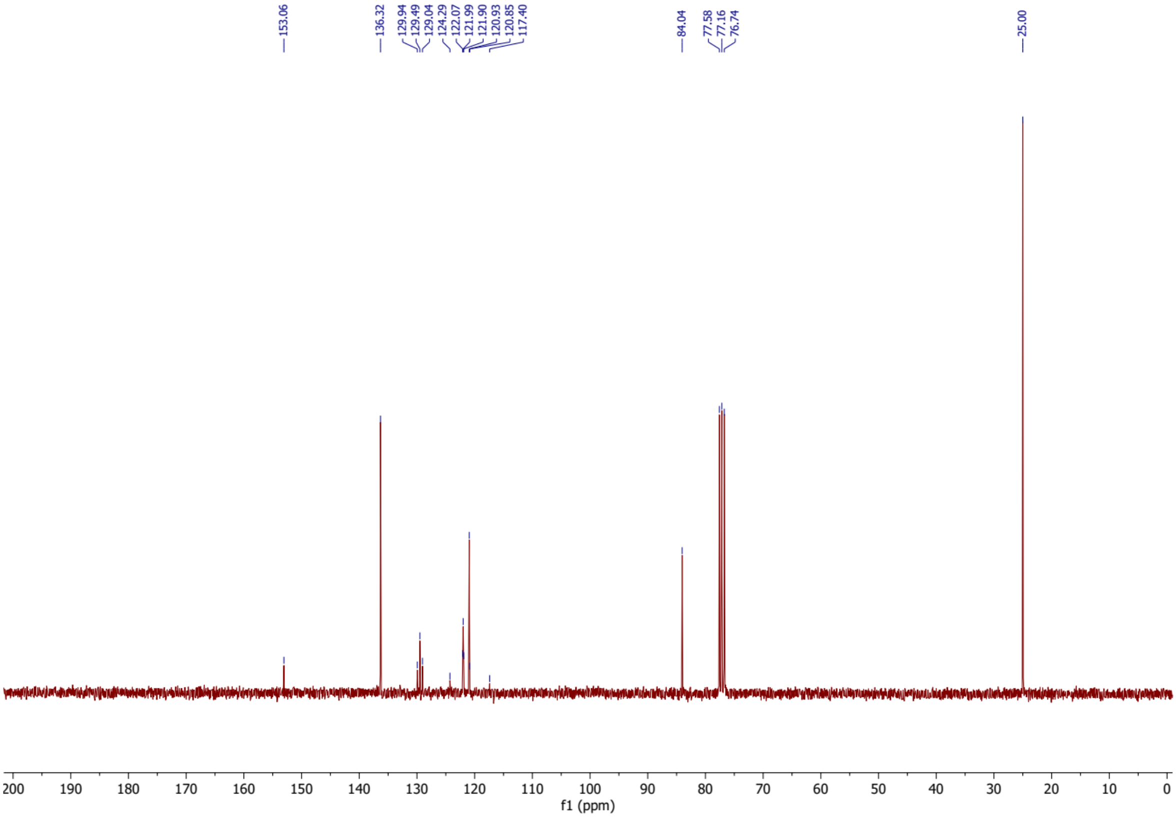

Supplementary Figure 72. ^{13}C NMR spectra of compound 15

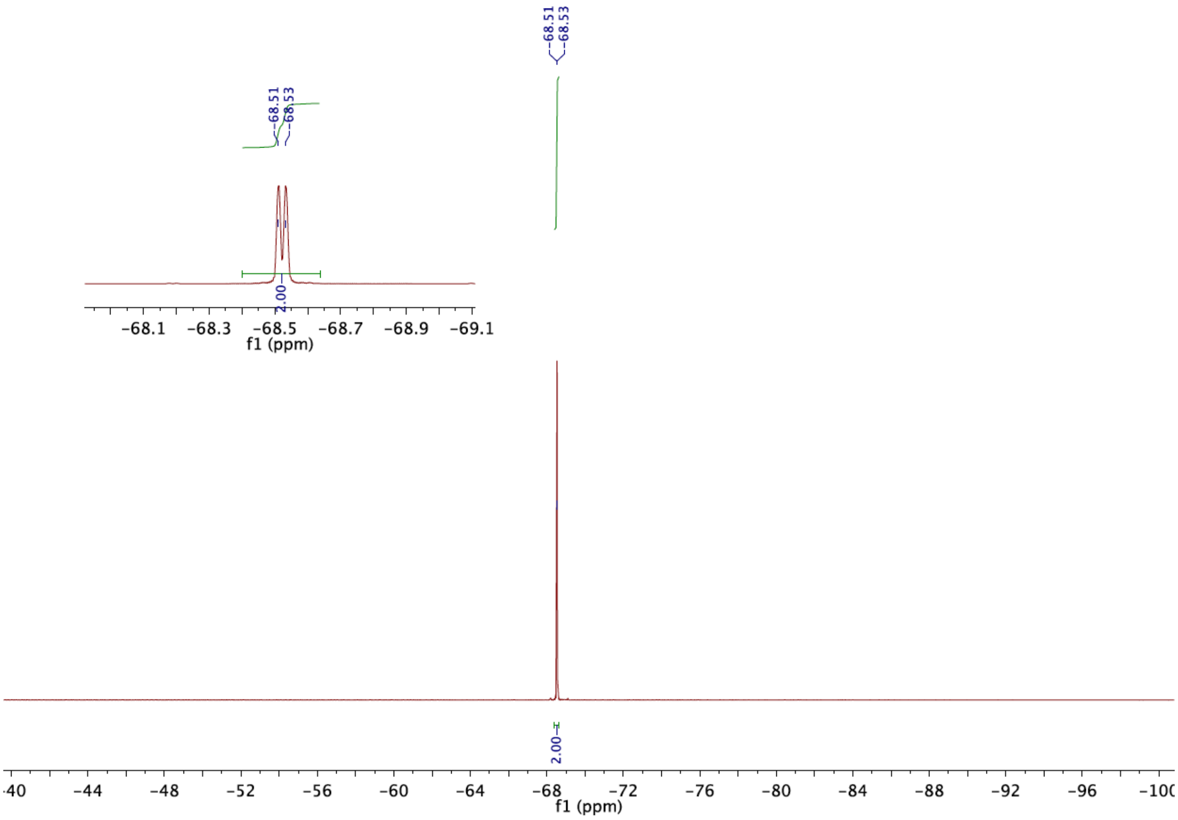

Supplementary Figure 73. ^{19}F NMR spectra of compound 15

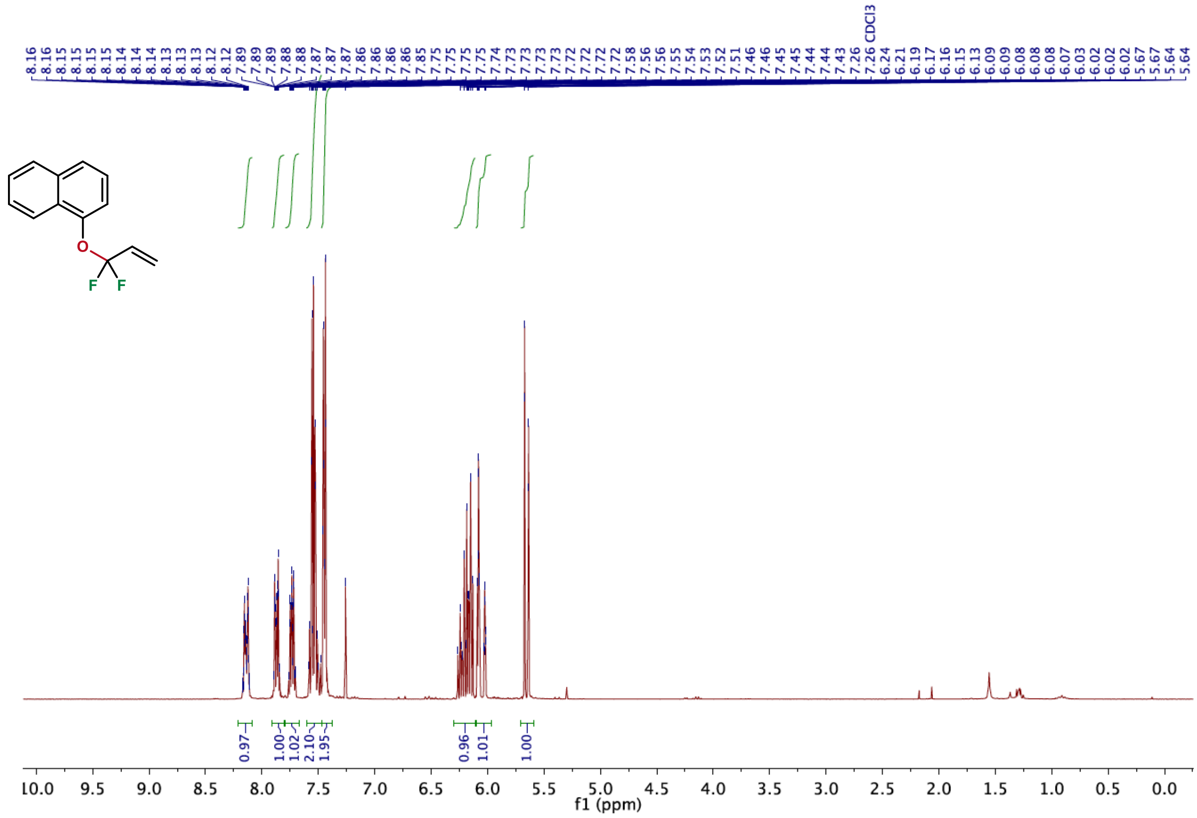

Supplementary Figure 74. ^{1}H NMR spectra of compound 16

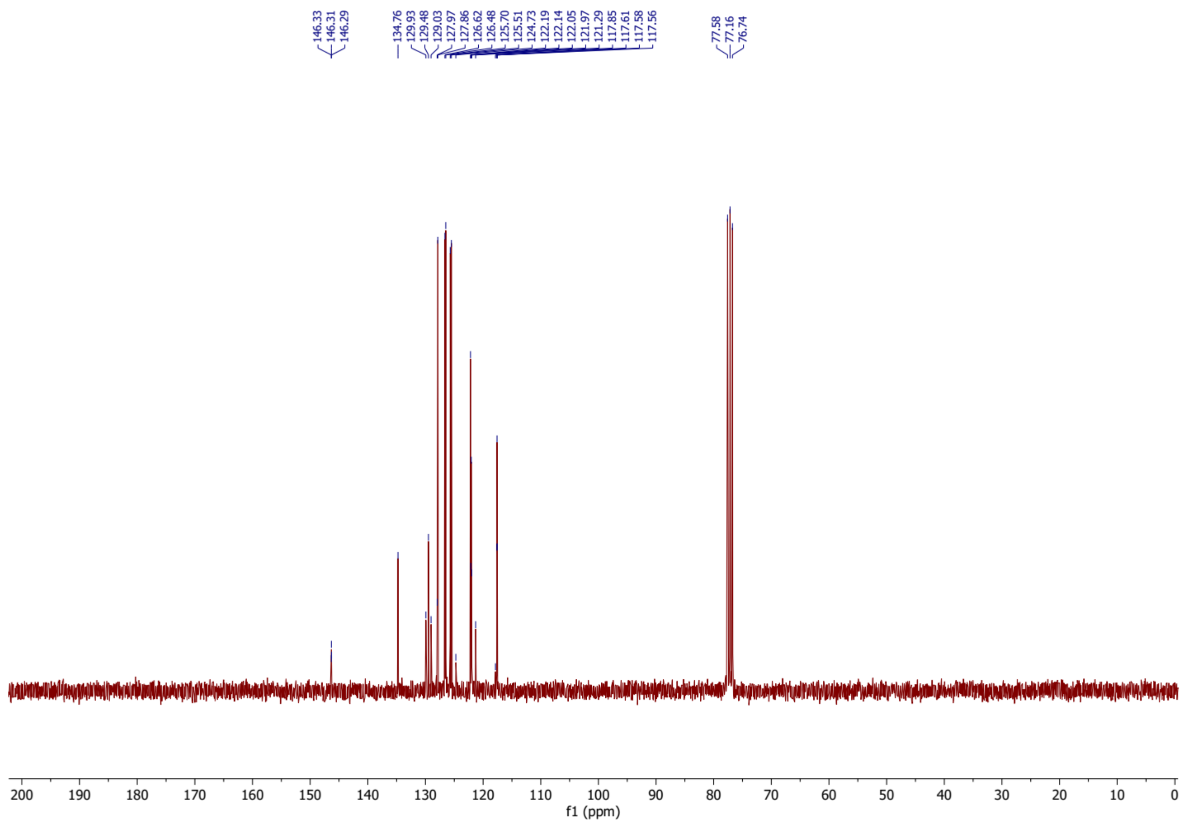

Supplementary Figure 75. ^{13}C NMR spectra of compound 16

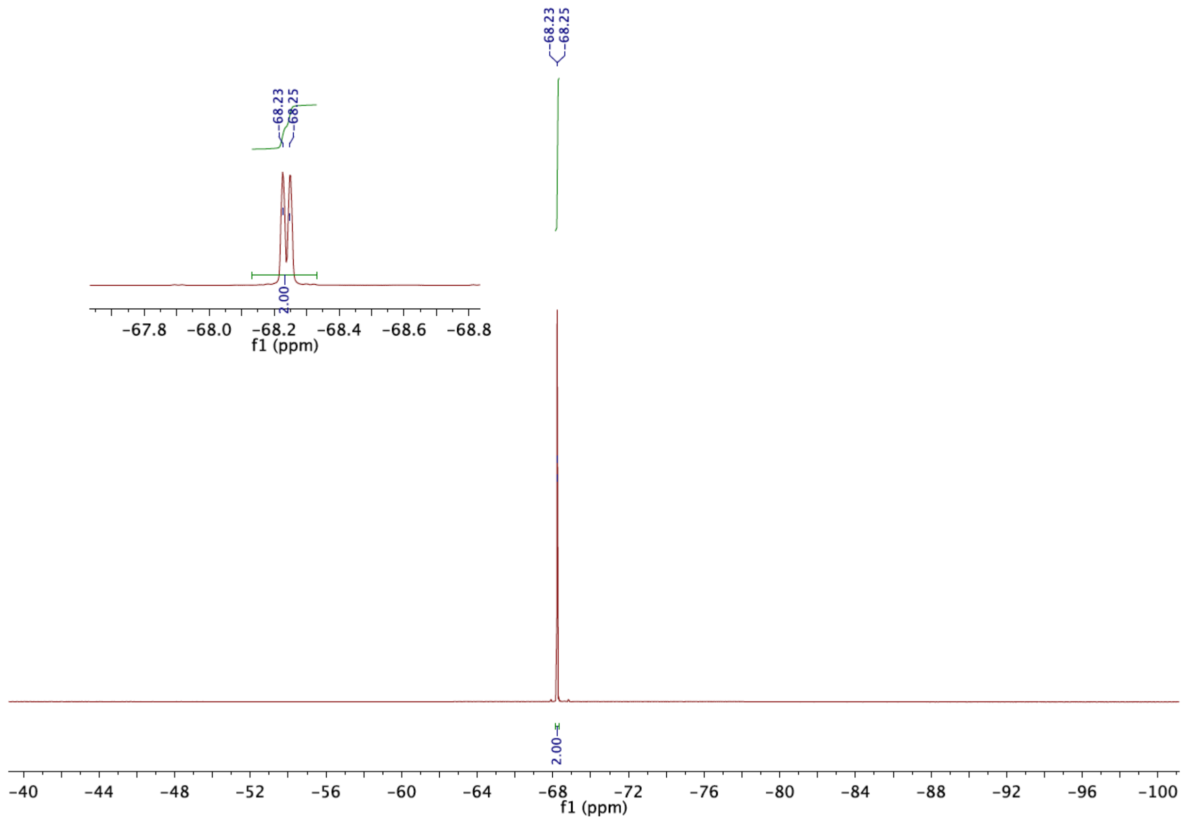

Supplementary Figure 76. ^{19}F NMR spectra of compound 16

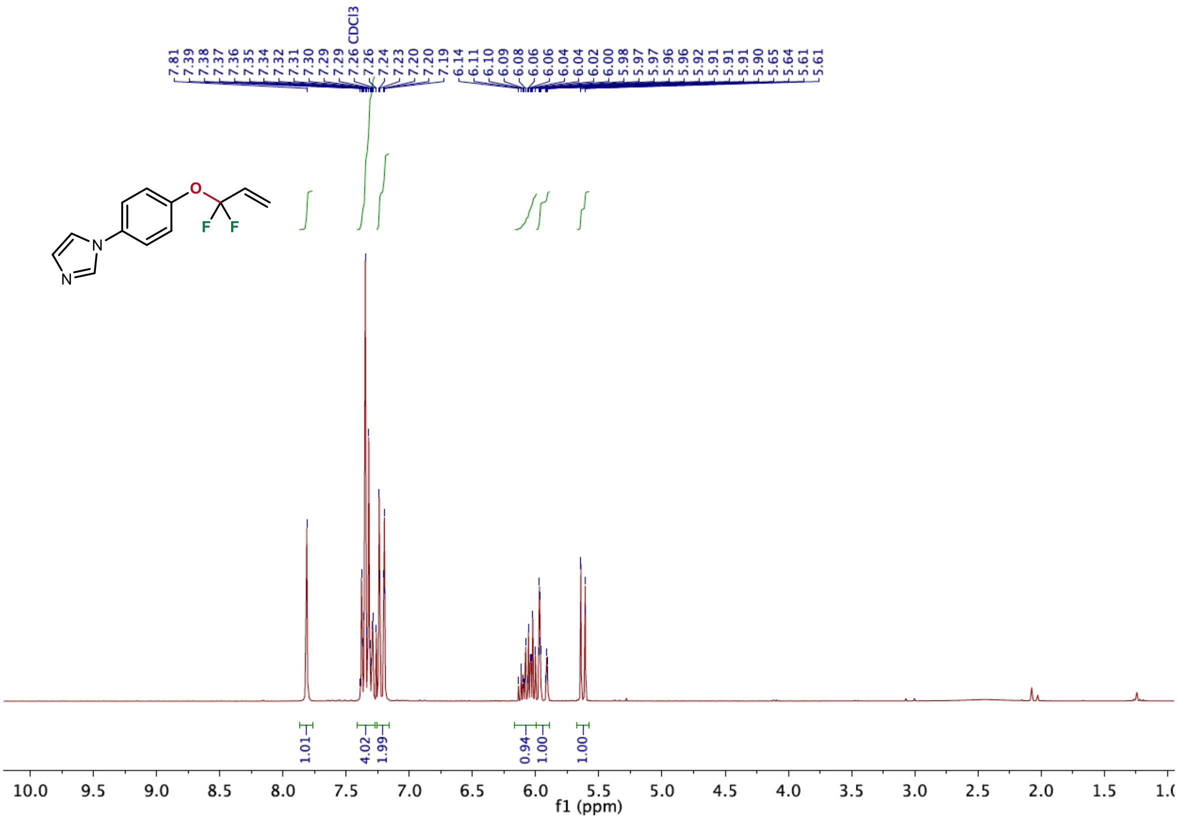

Supplementary Figure 77. ^{1}H NMR spectra of compound 17

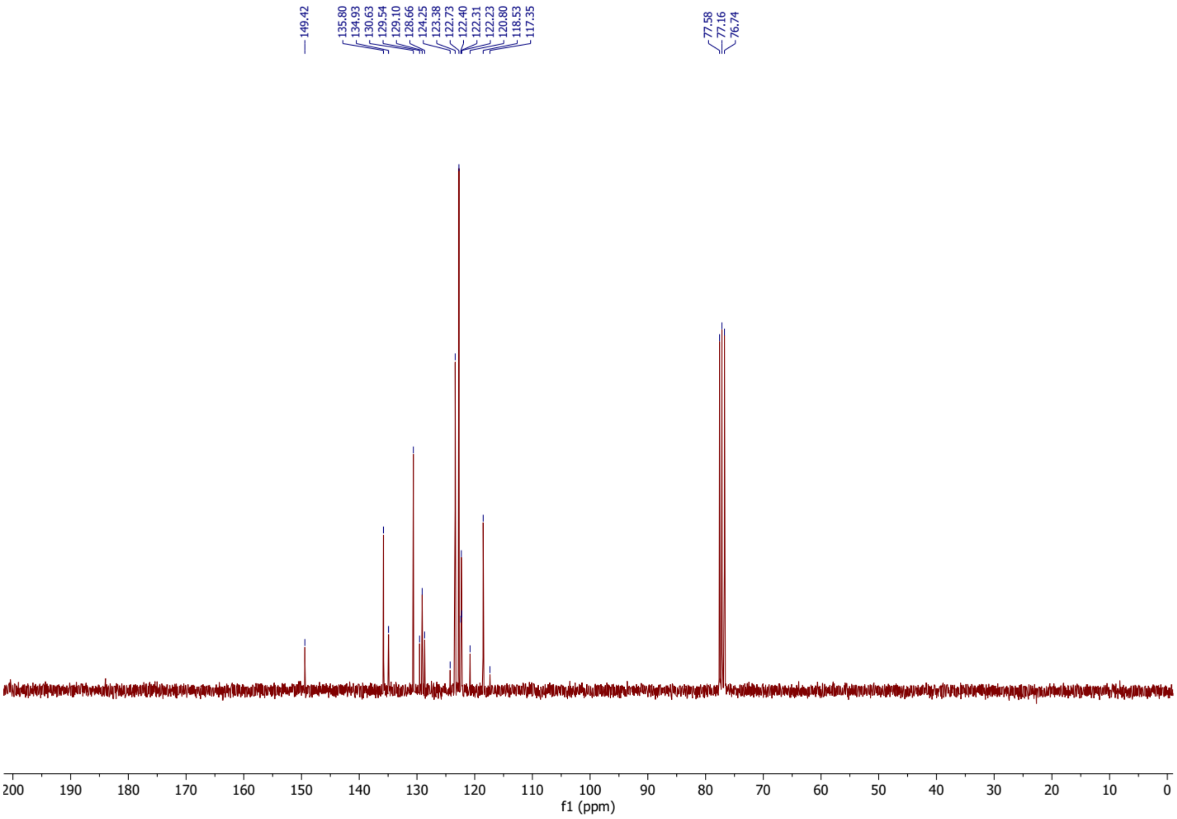

Supplementary Figure 78. ^{13}C NMR spectra of compound 17

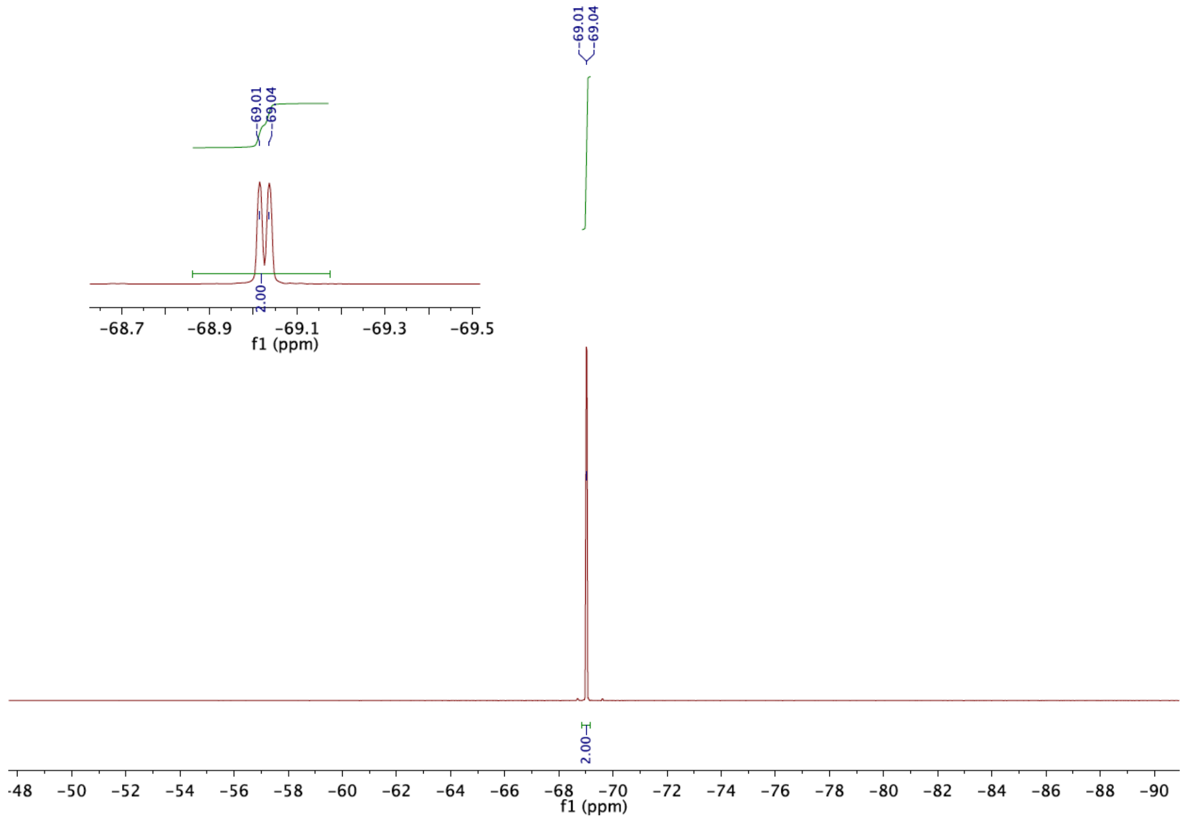

Supplementary Figure 79. ^{19}F NMR spectra of compound 17

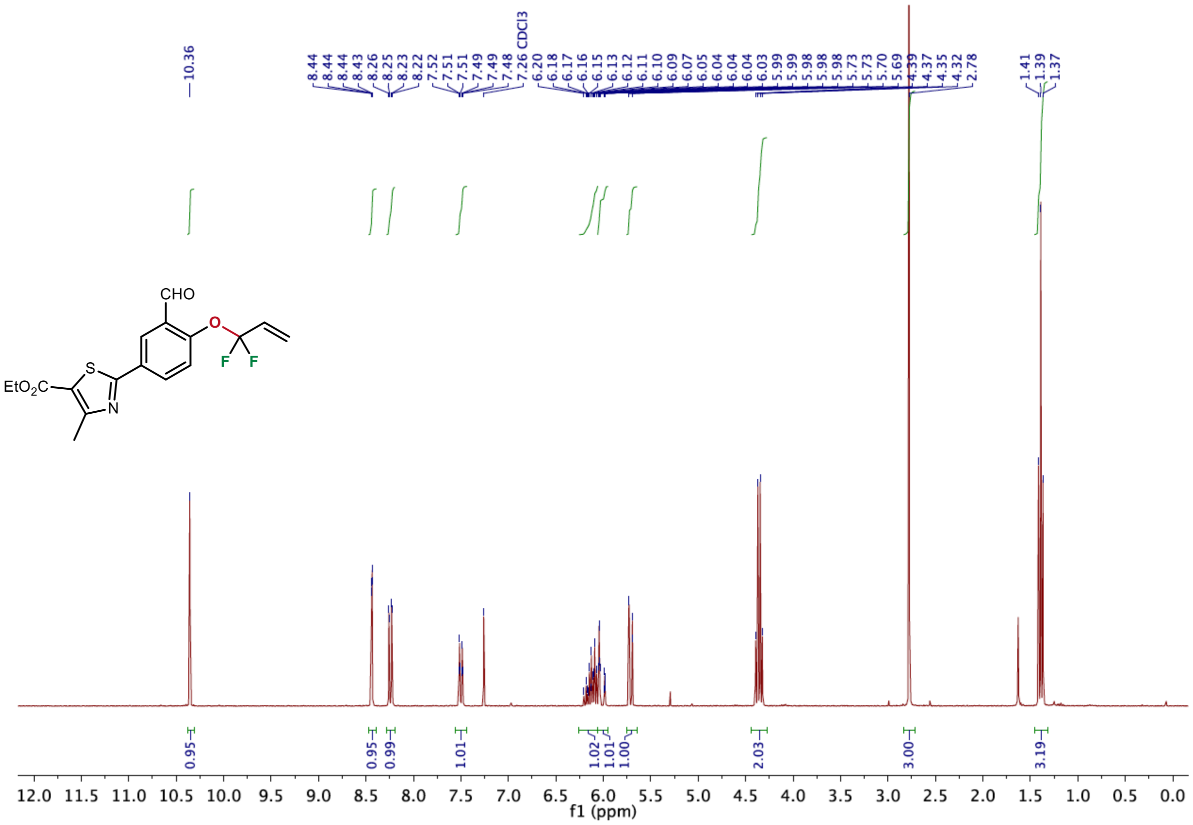

Supplementary Figure 80. ^{1}H NMR spectra of compound 18

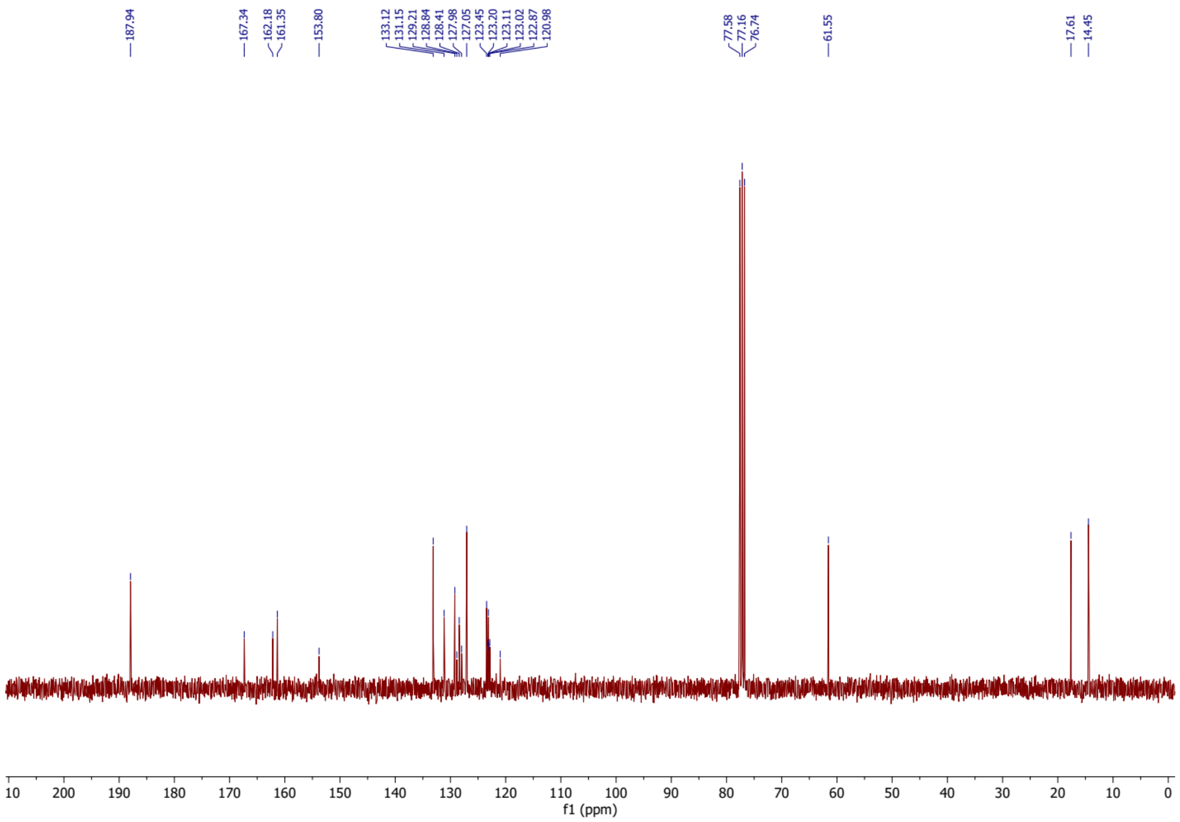

Supplementary Figure 81. ^{13}C NMR spectra of compound 18

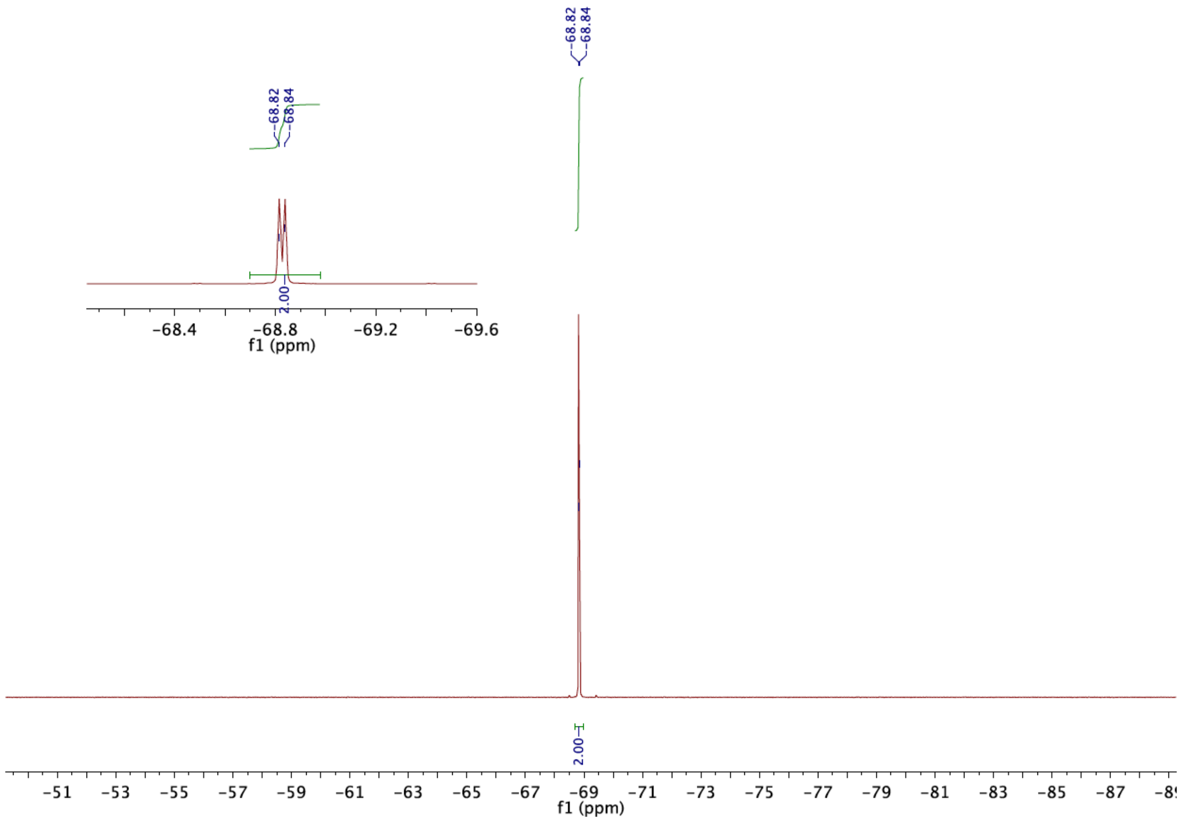

Supplementary Figure 82. ^{19}F NMR spectra of compound 18

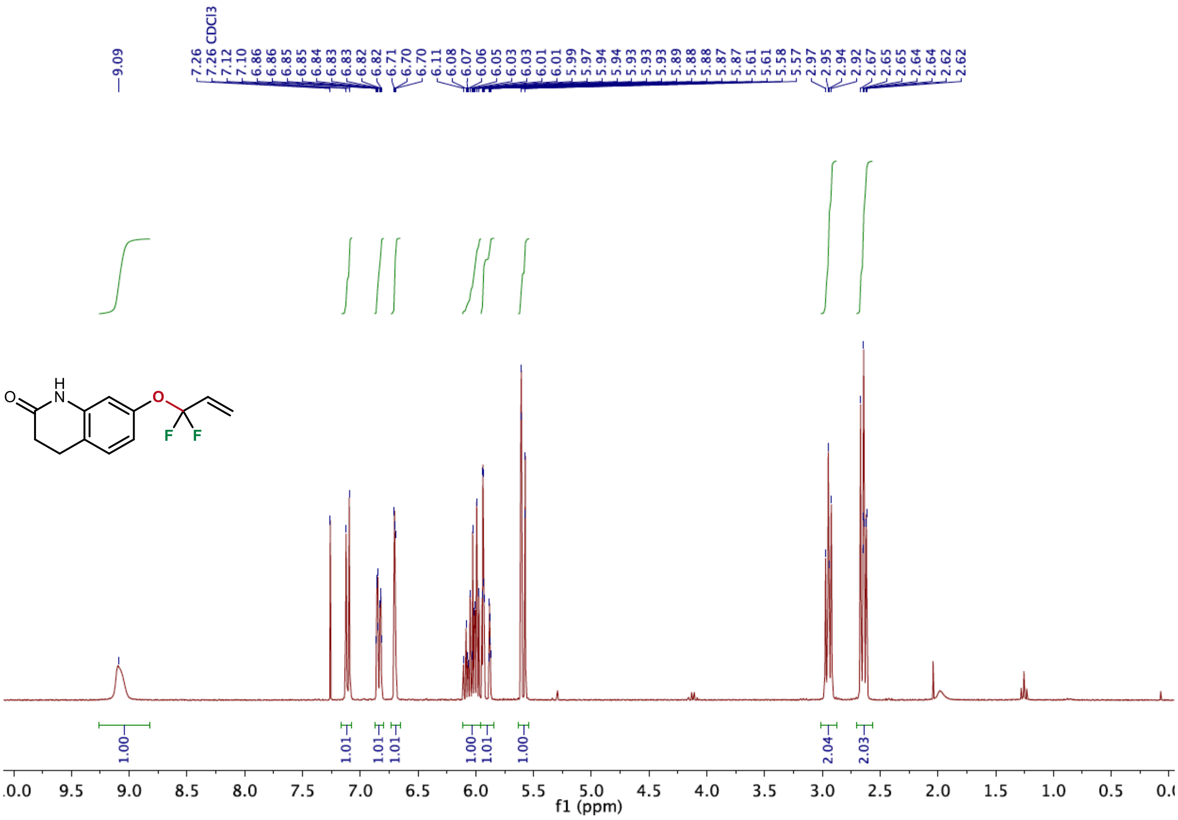

Supplementary Figure 83. ^{1}H NMR spectra of compound 19

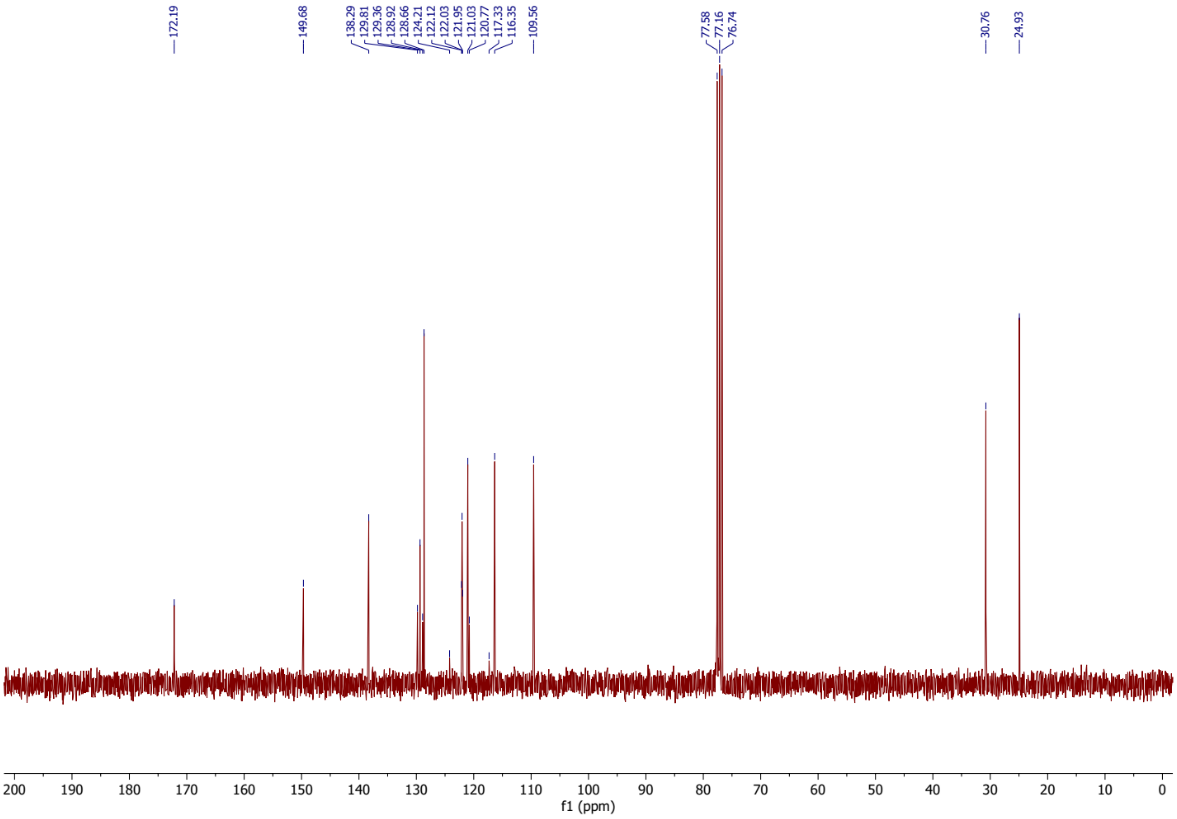

Supplementary Figure 84. ^{13}C NMR spectra of compound 19

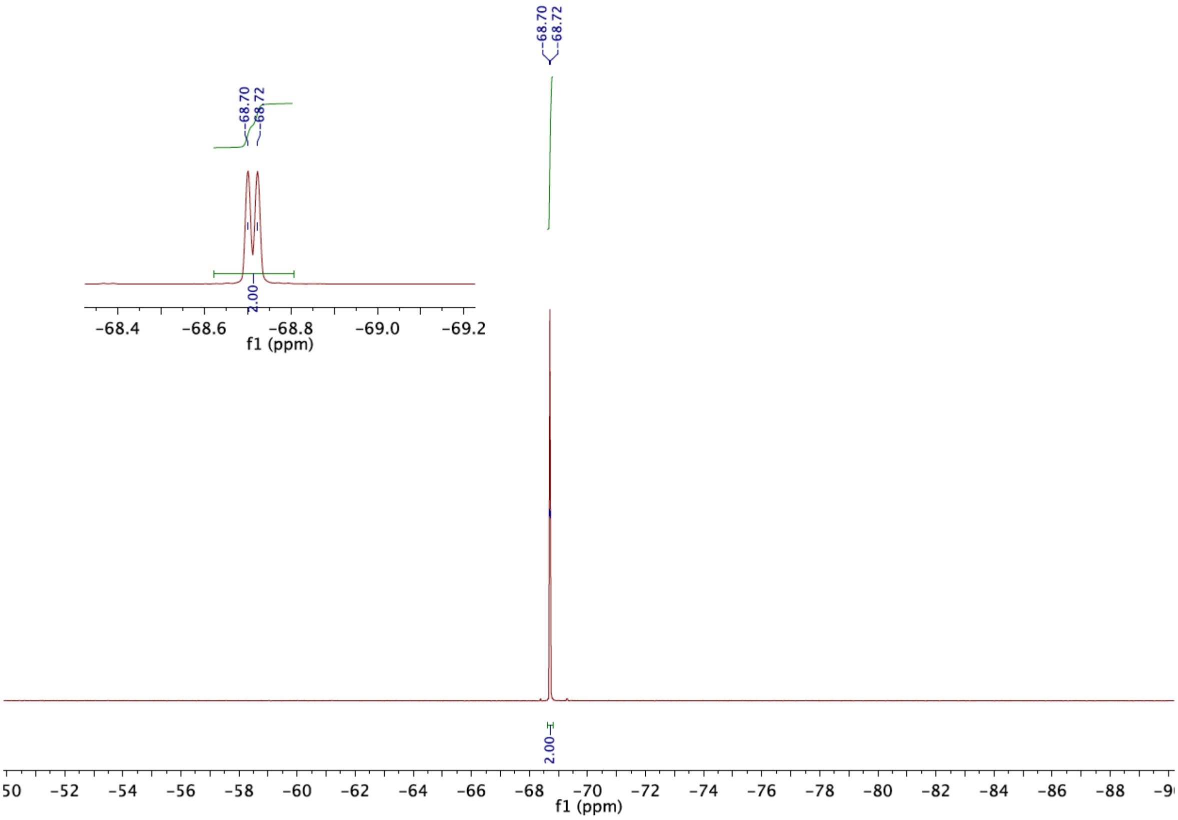

Supplementary Figure 85. ^{19}F NMR spectra of compound 19

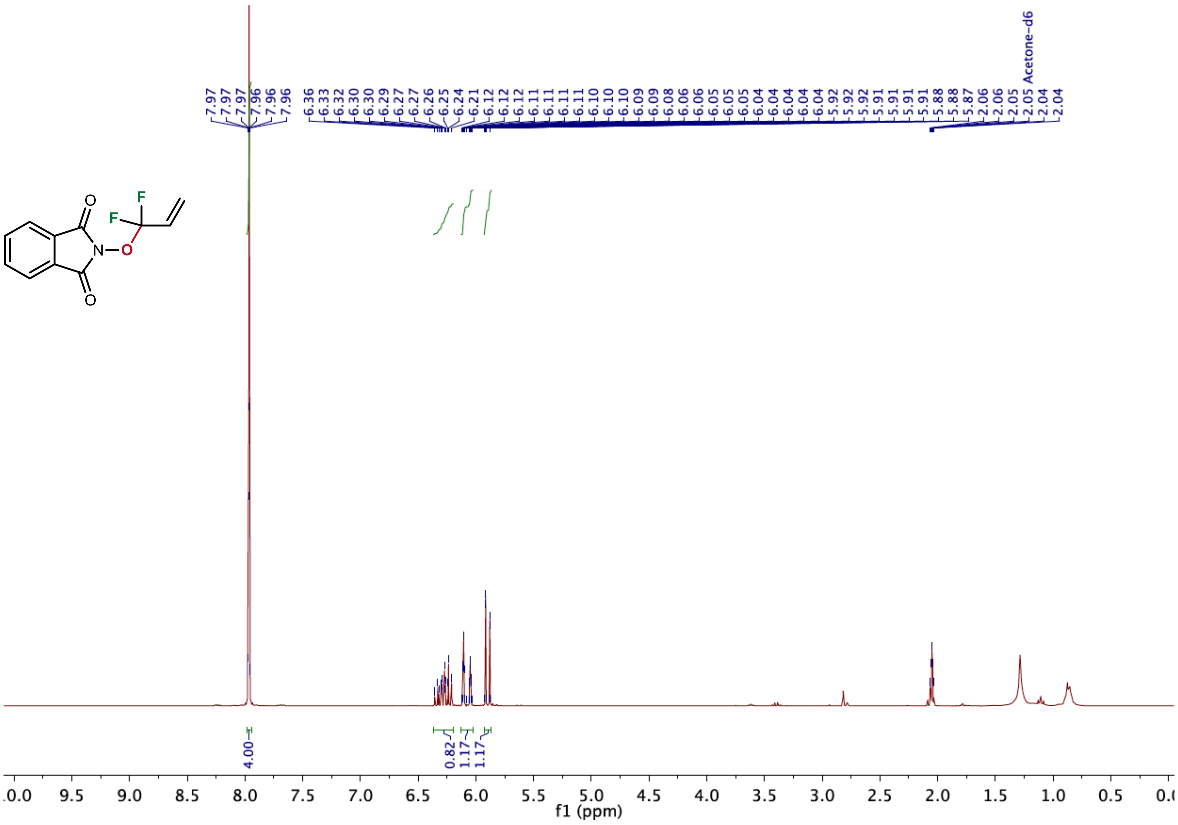

Supplementary Figure 86. ^{1}H NMR spectra of compound 20

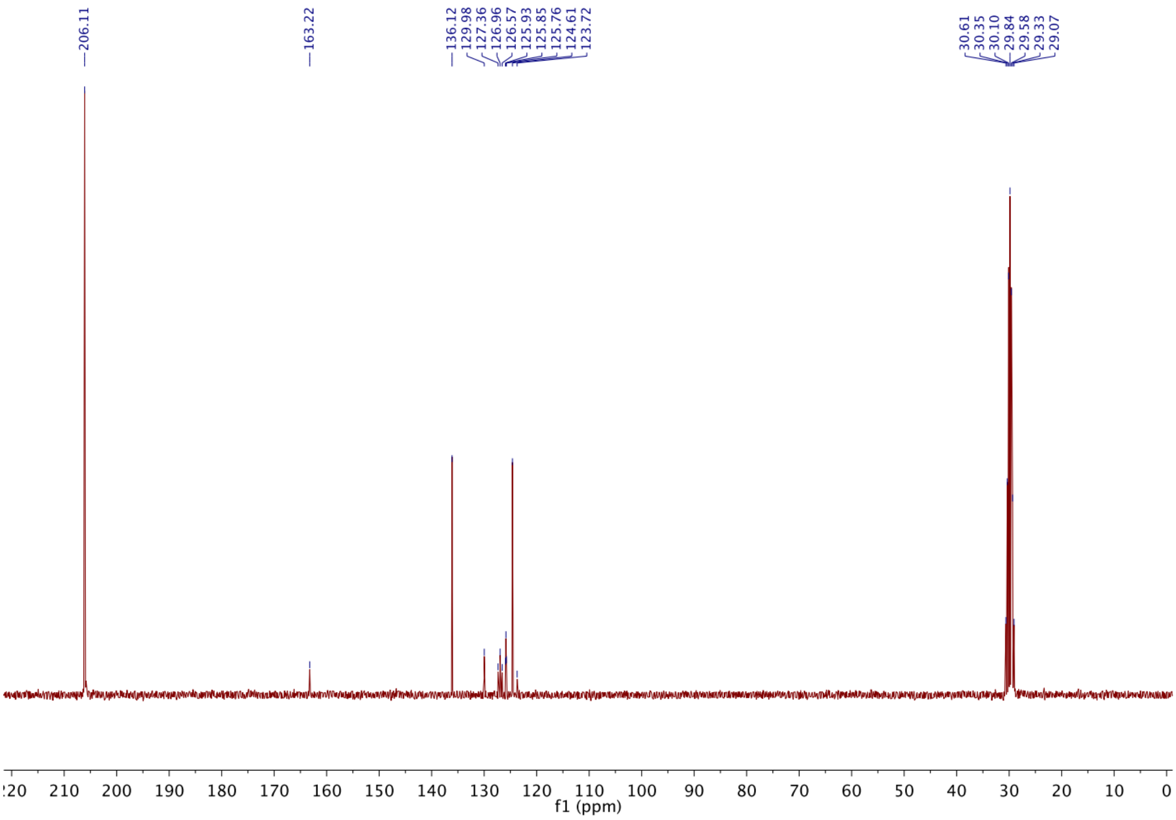

Supplementary Figure 87. ^{13}C NMR spectra of compound 20

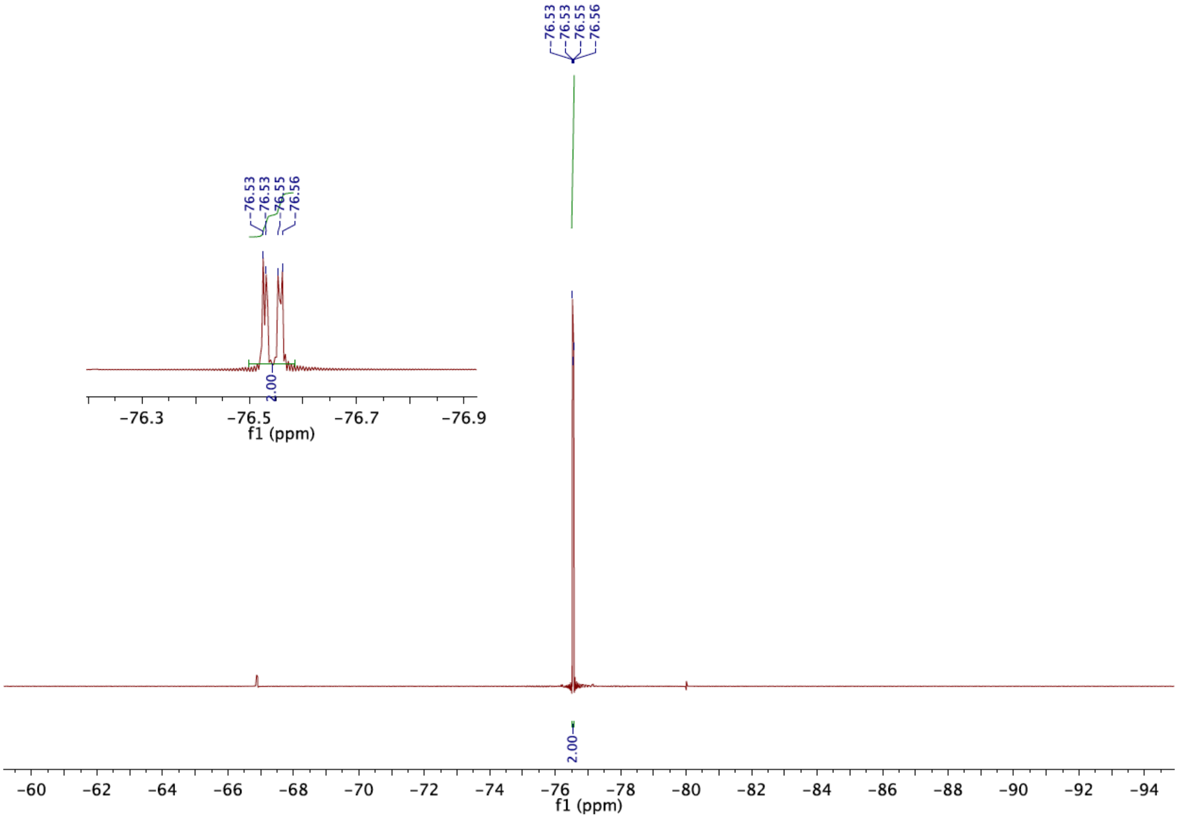

Supplementary Figure 88. ^{19}F NMR spectra of compound 20

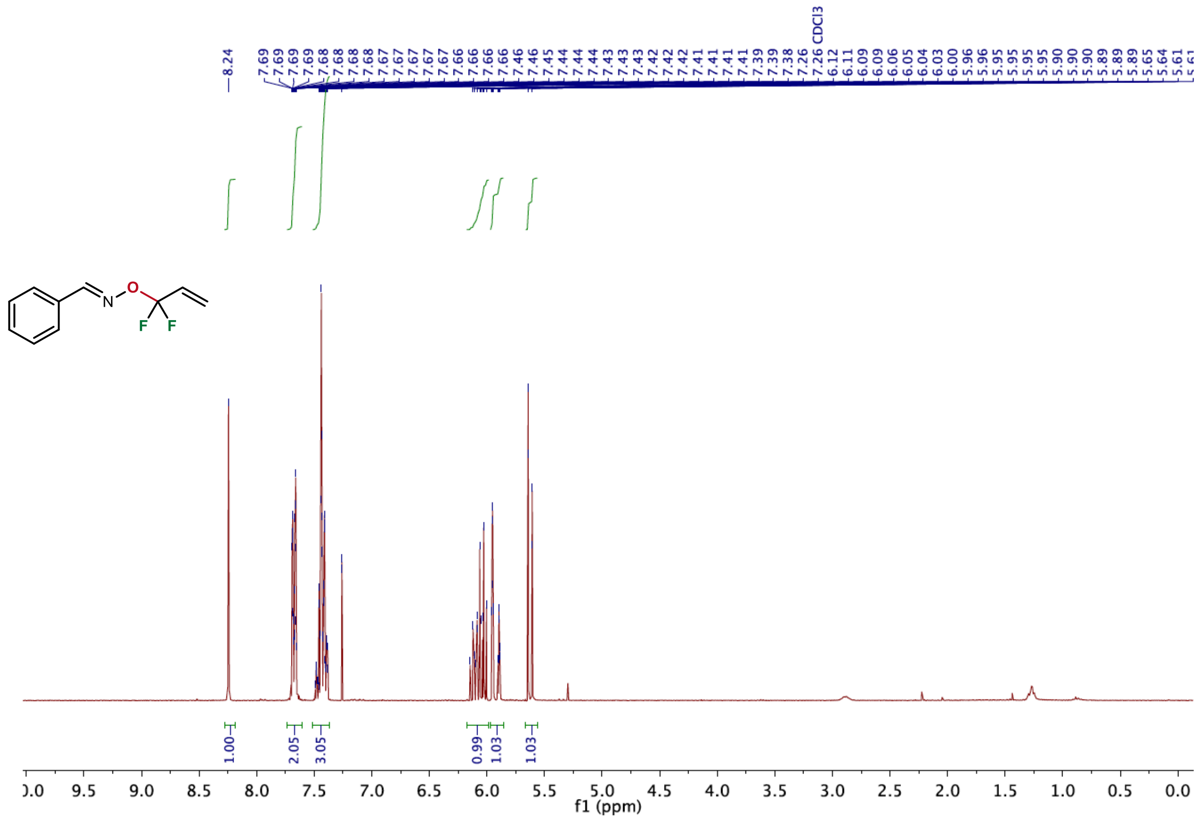

Supplementary Figure 89. ^{1}H NMR spectra of compound 21

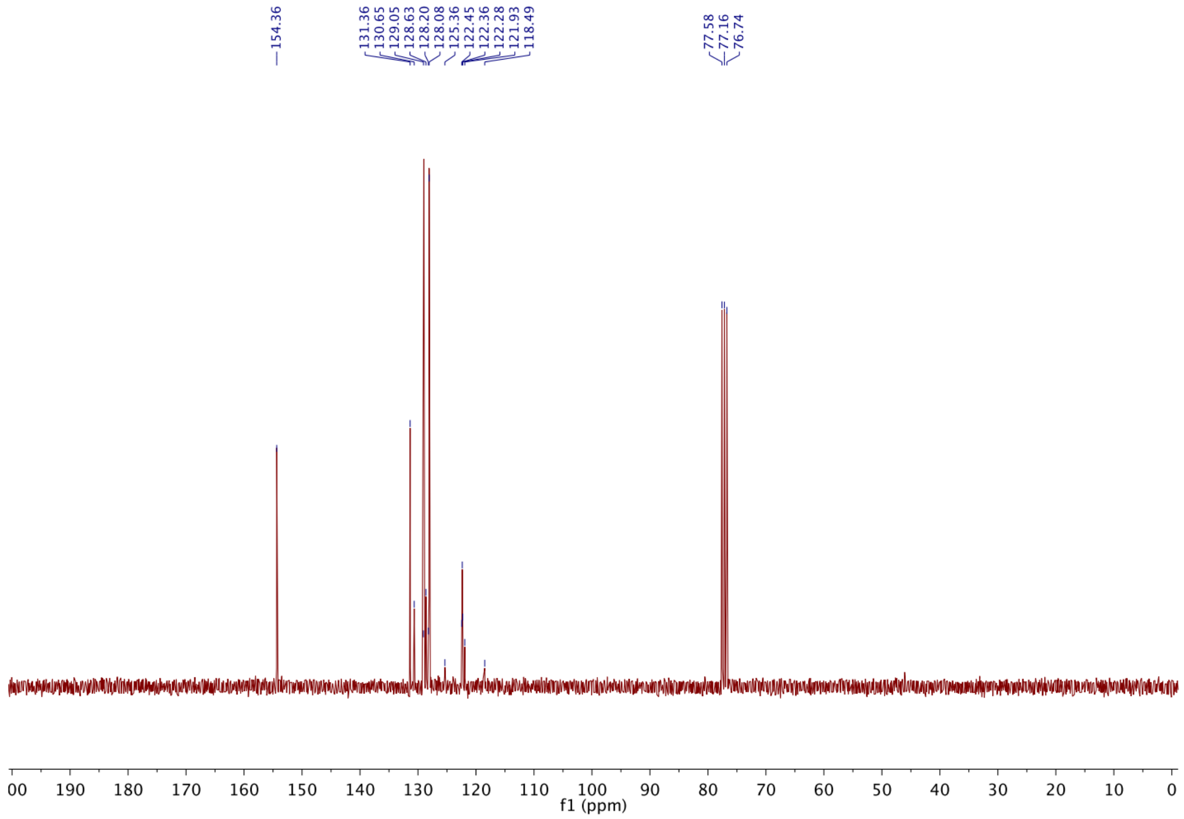

Supplementary Figure 90. ^{13}C NMR spectra of compound 21

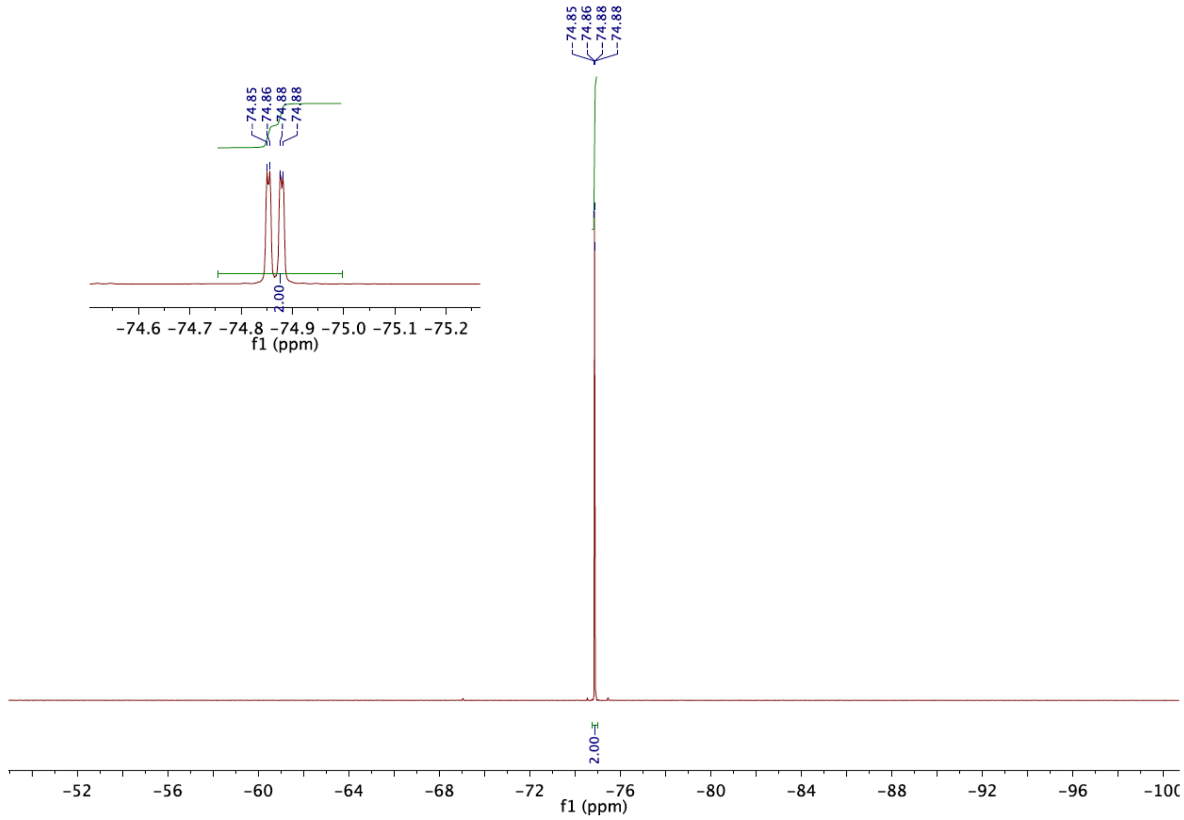

Supplementary Figure 91. ^{19}F NMR spectra of compound 21

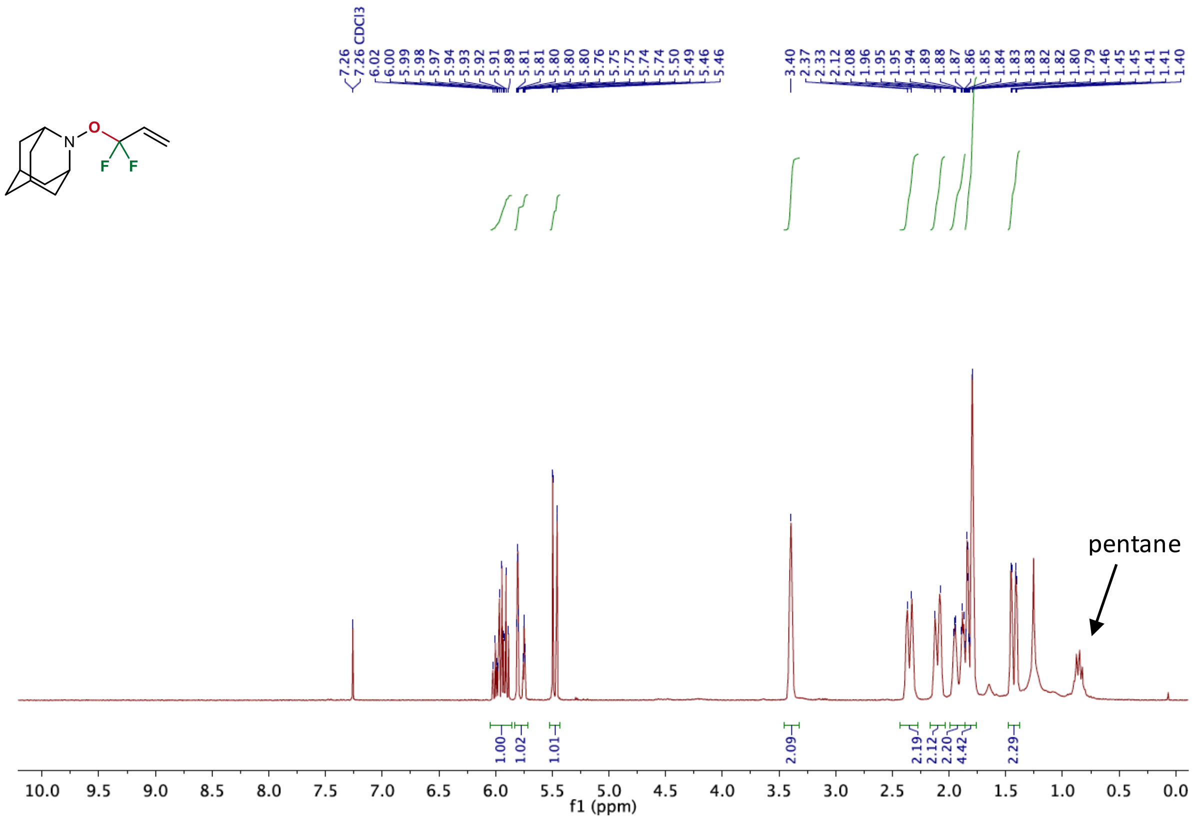

Supplementary Figure 92. ^{1}H NMR spectra of compound 22

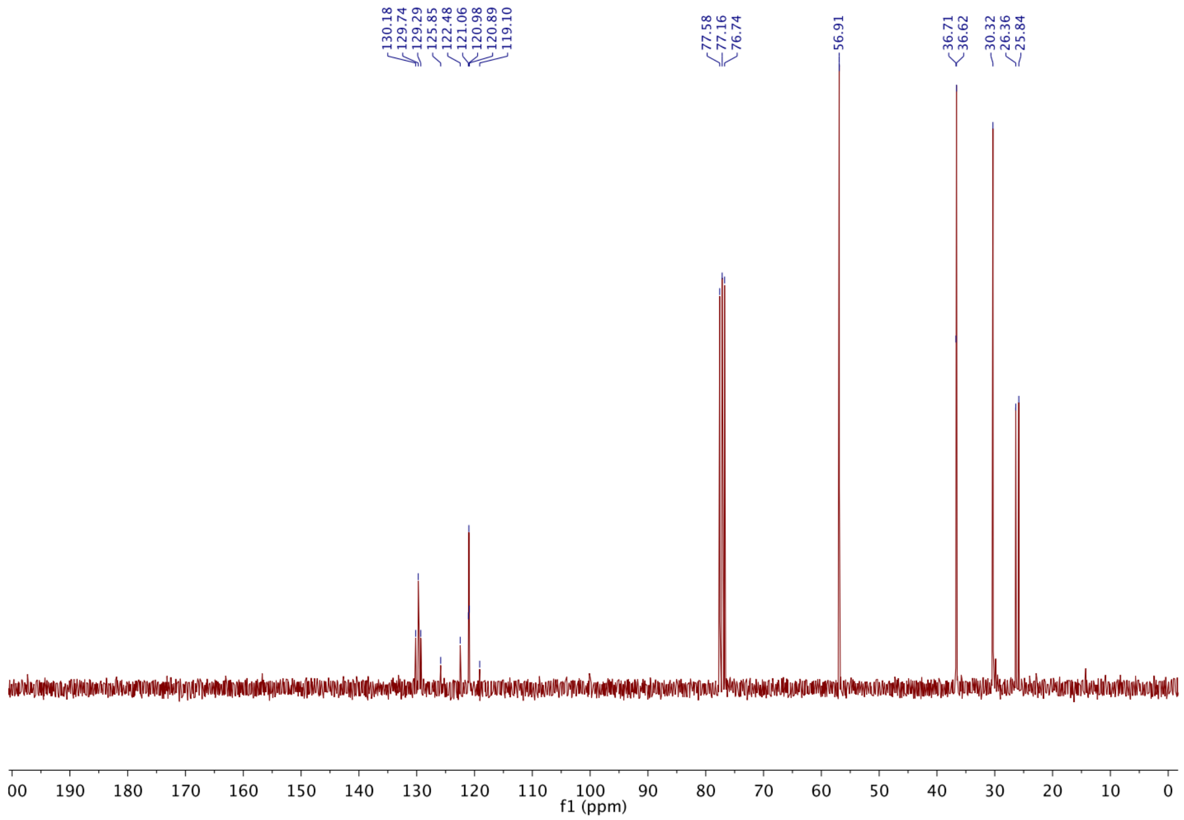

Supplementary Figure 93. ^{13}C NMR spectra of compound 22

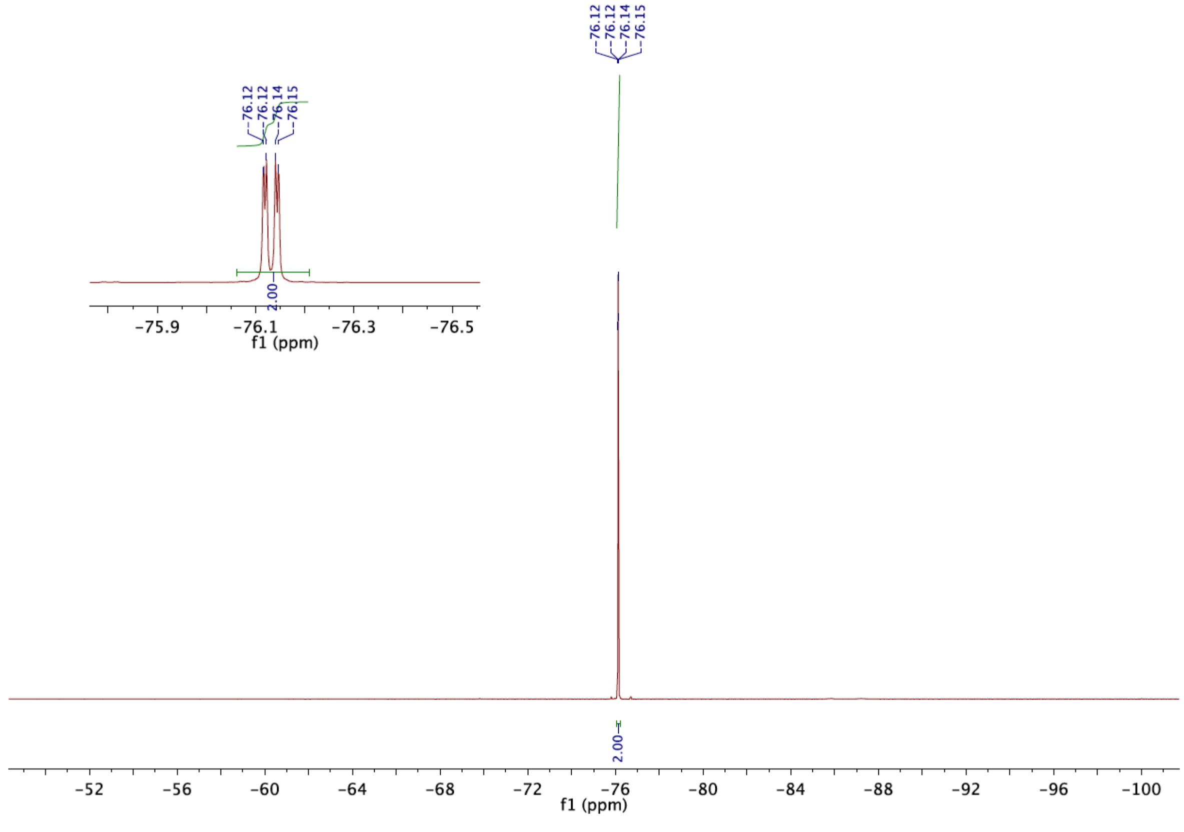

Supplementary Figure 94. ^{19}F NMR spectra of compound 22

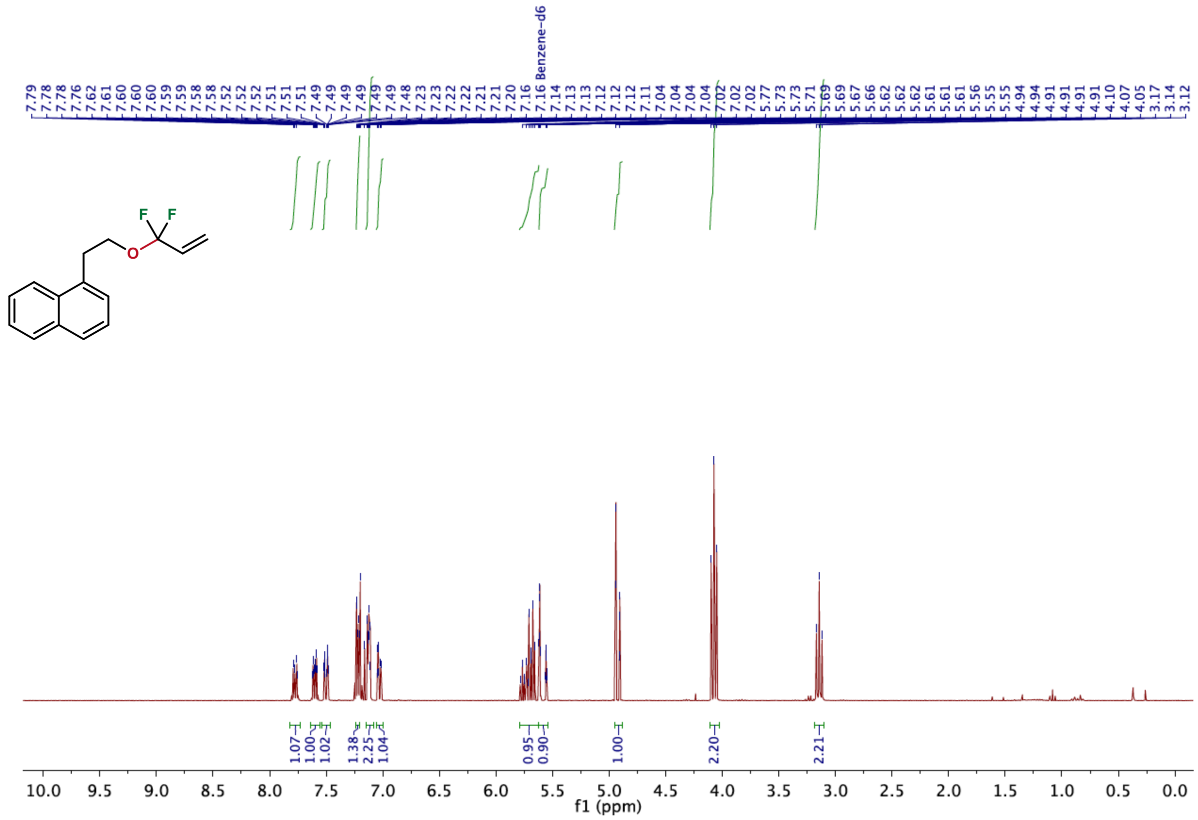

Supplementary Figure 95. ^{1}H NMR spectra of compound 23

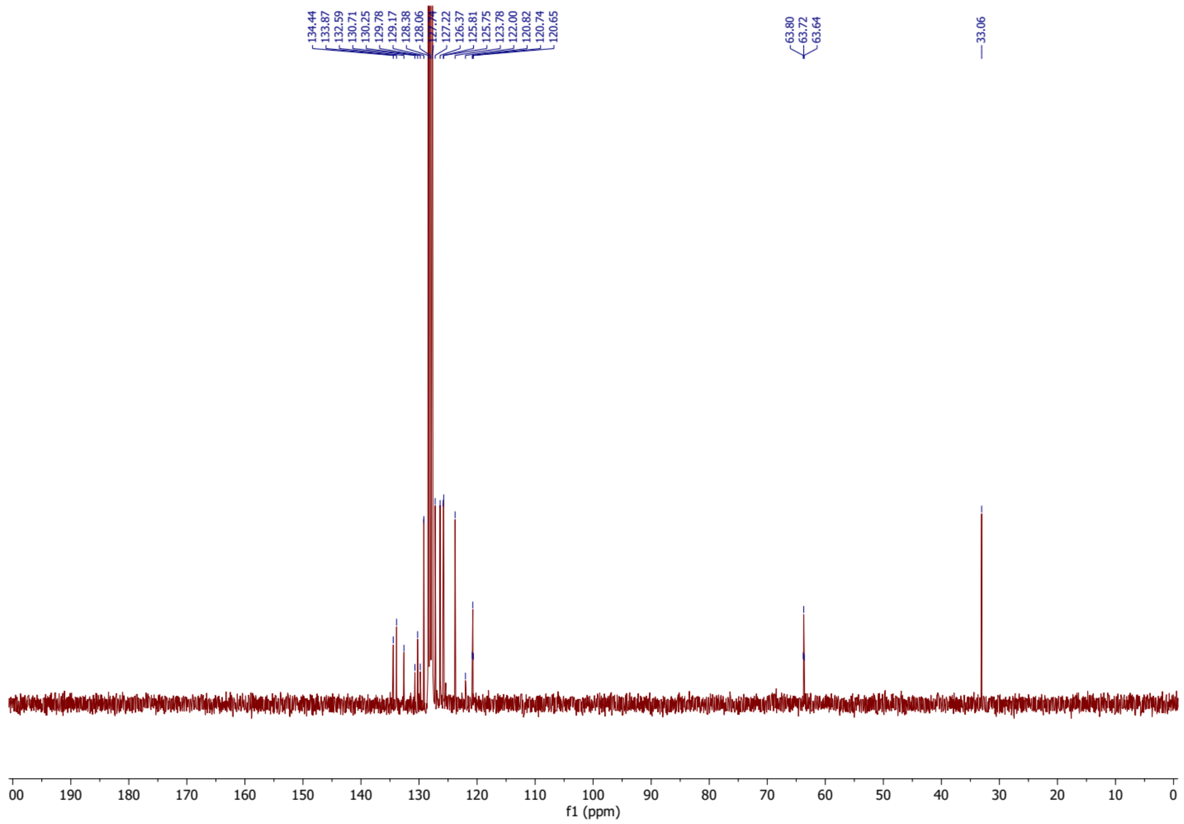

Supplementary Figure 96. ^{13}C NMR spectra of compound 23

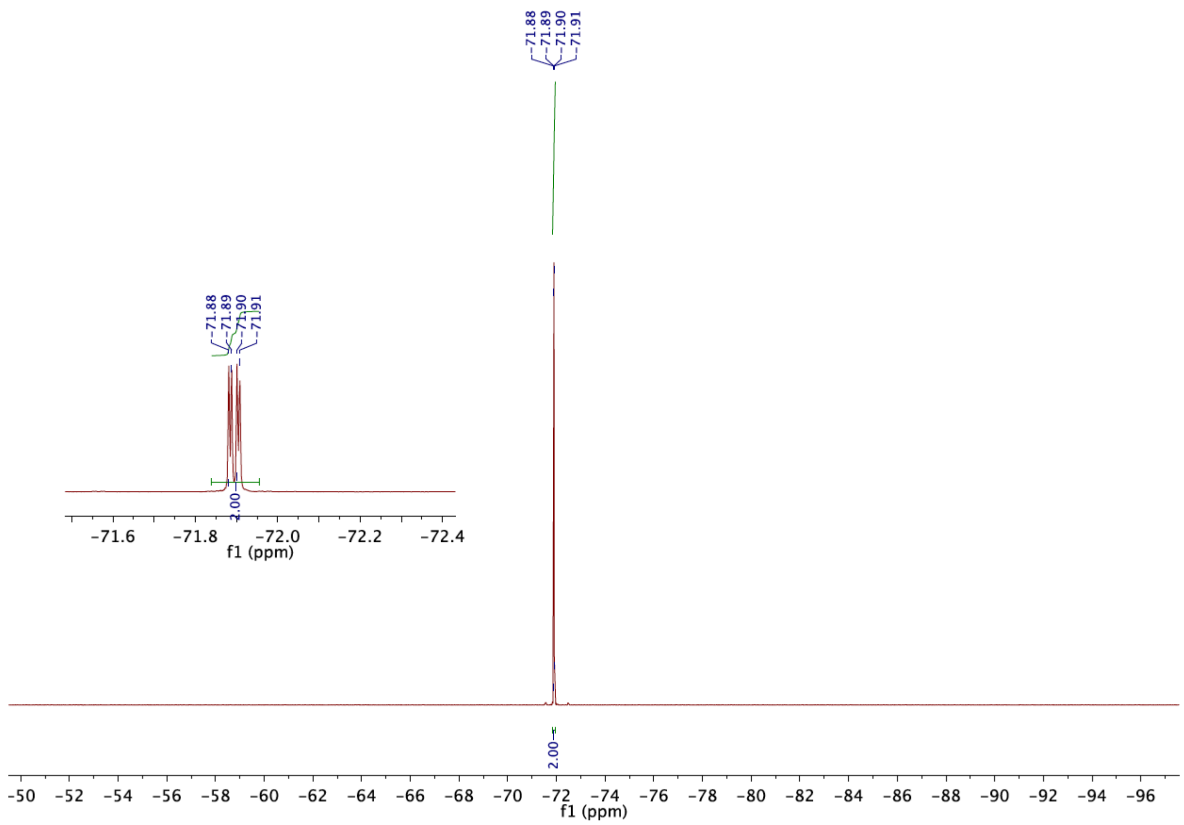

Supplementary Figure 97. ^{19}F NMR spectra of compound 23

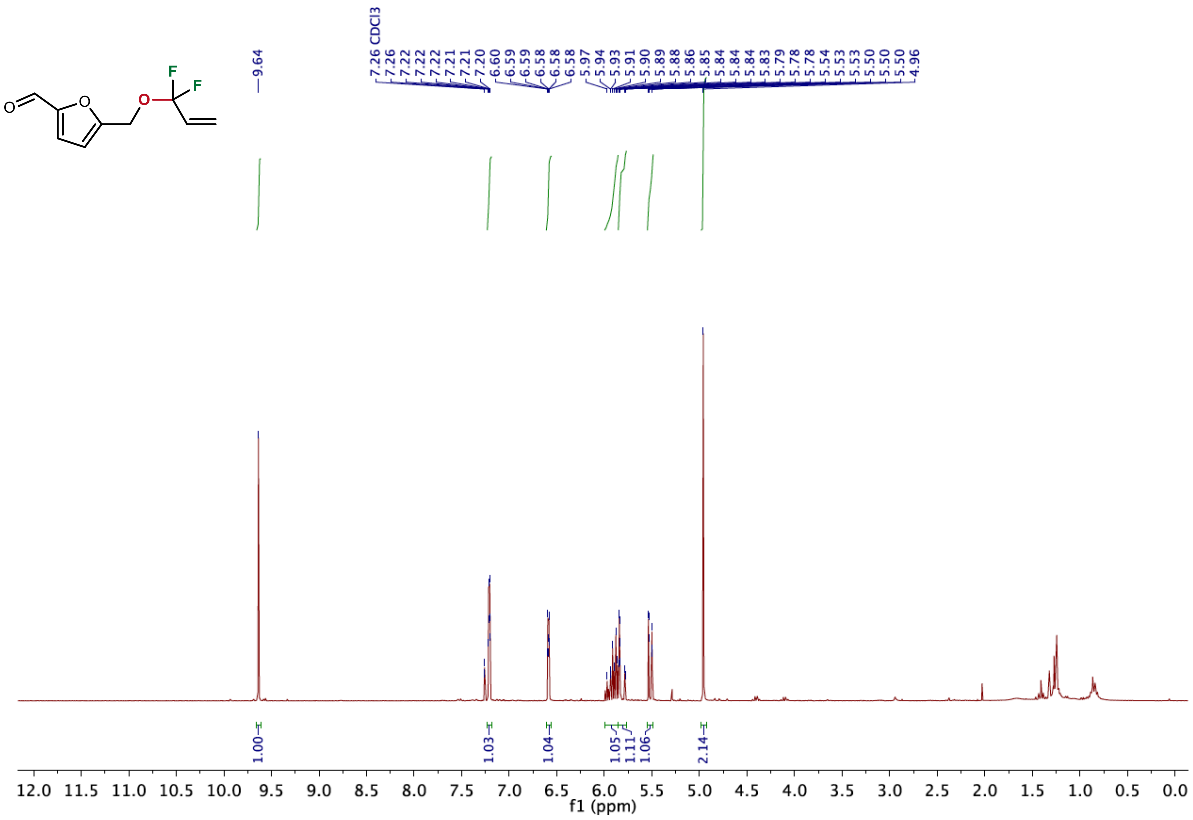

Supplementary Figure 98. ^{1}H NMR spectra of compound 24

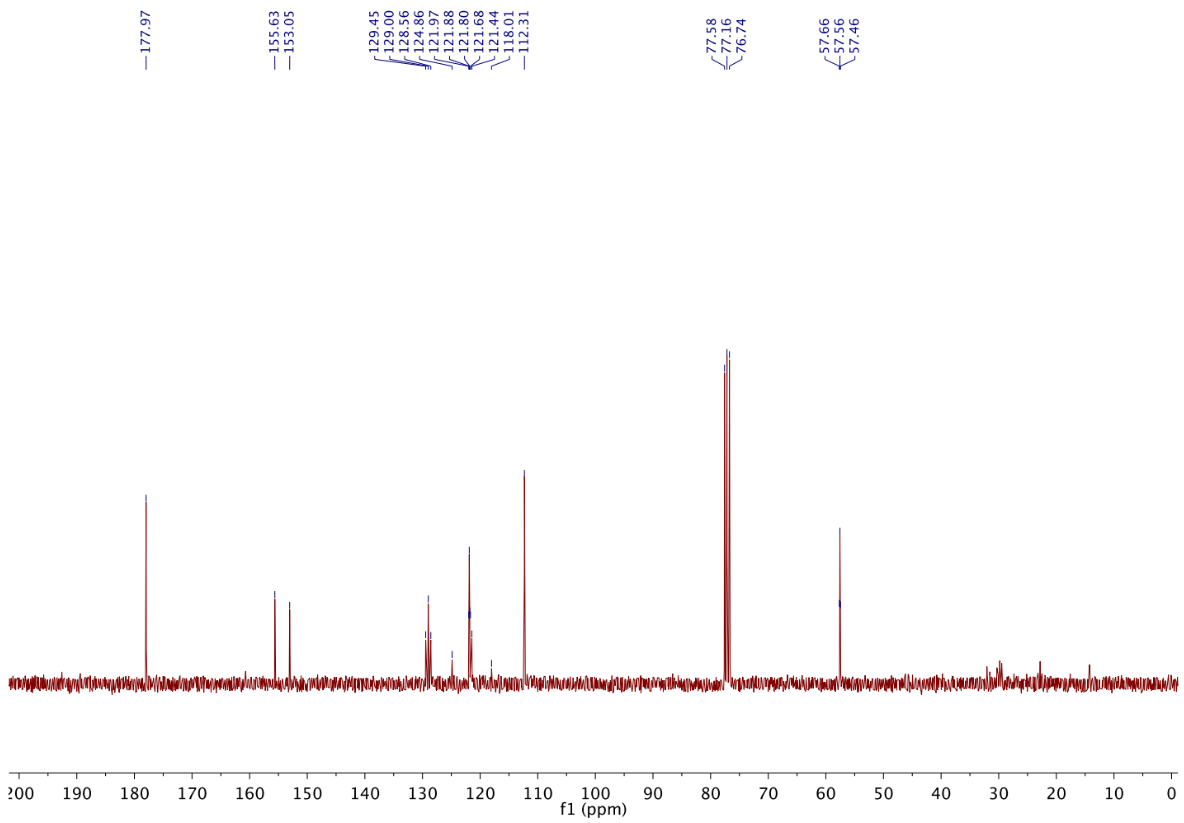

Supplementary Figure 99. ^{13}C NMR spectra of compound 24

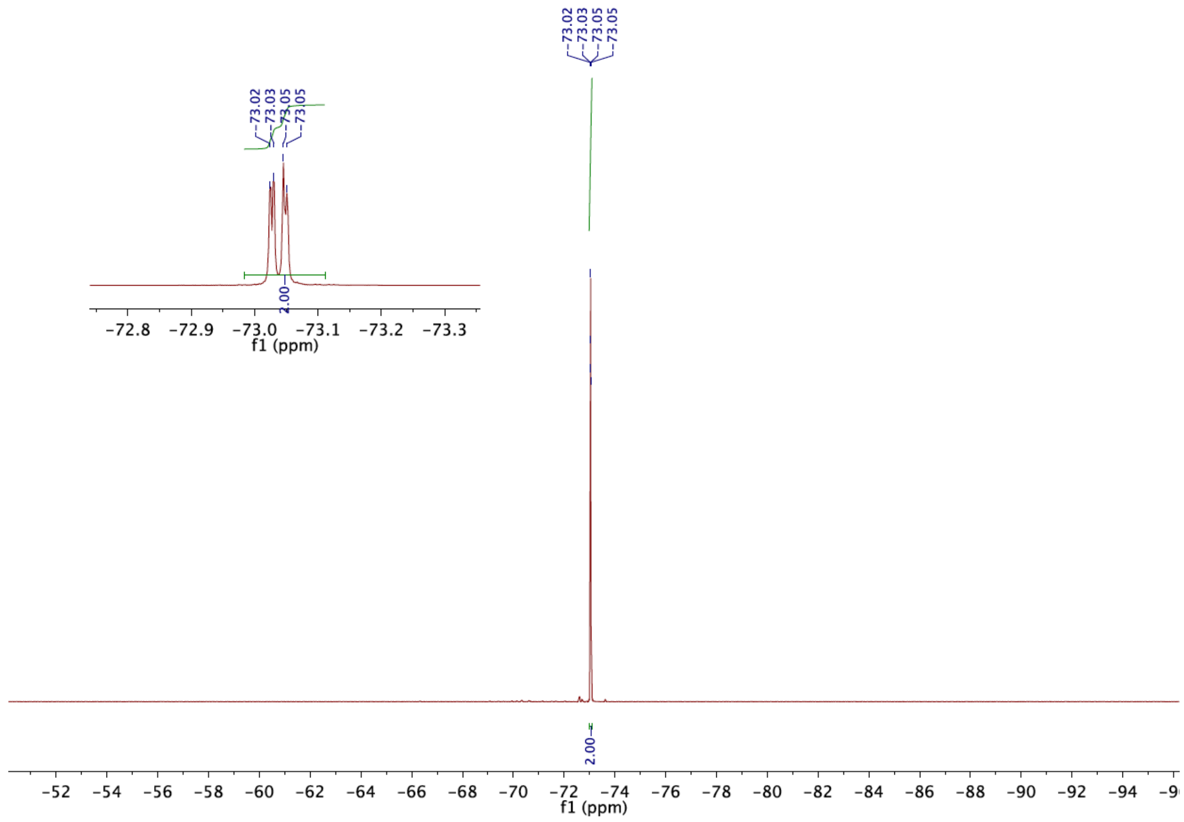

Supplementary Figure 100. ^{19}F NMR spectra of compound 24

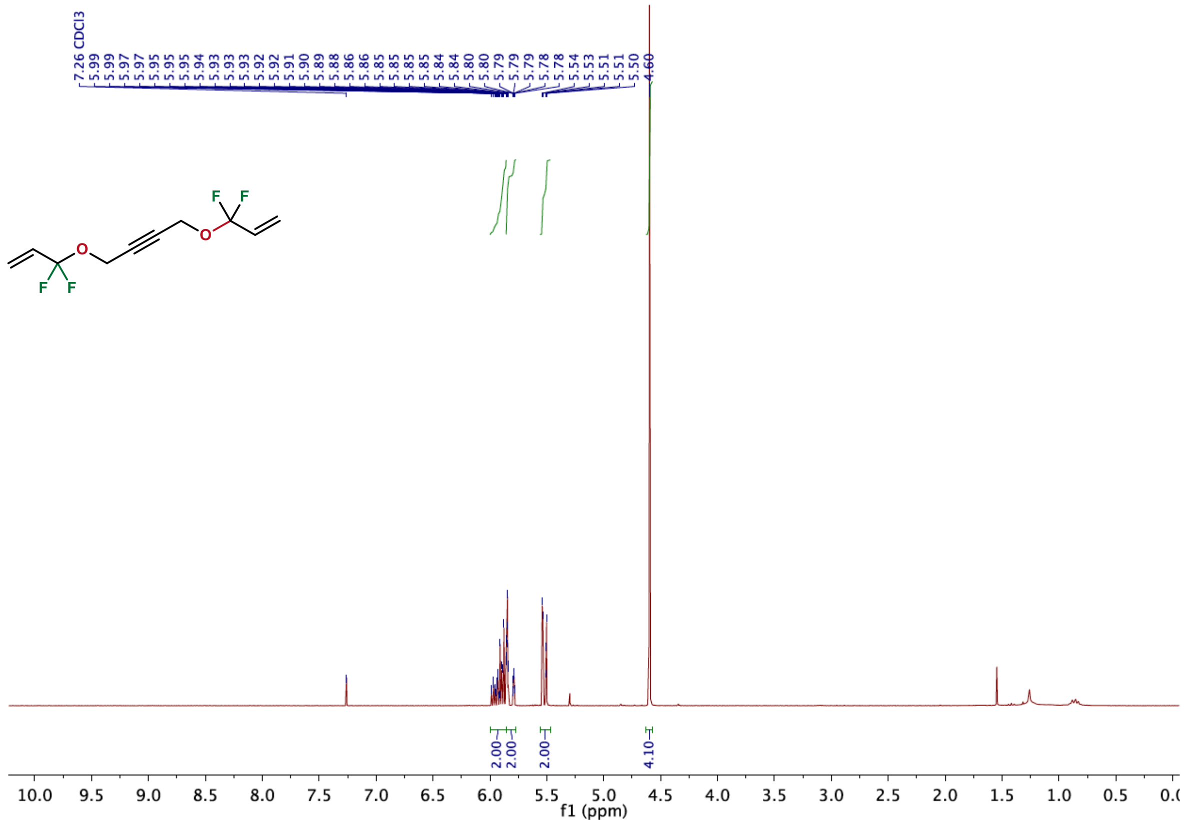

Supplementary Figure 101. ^{1}H NMR spectra of compound 25

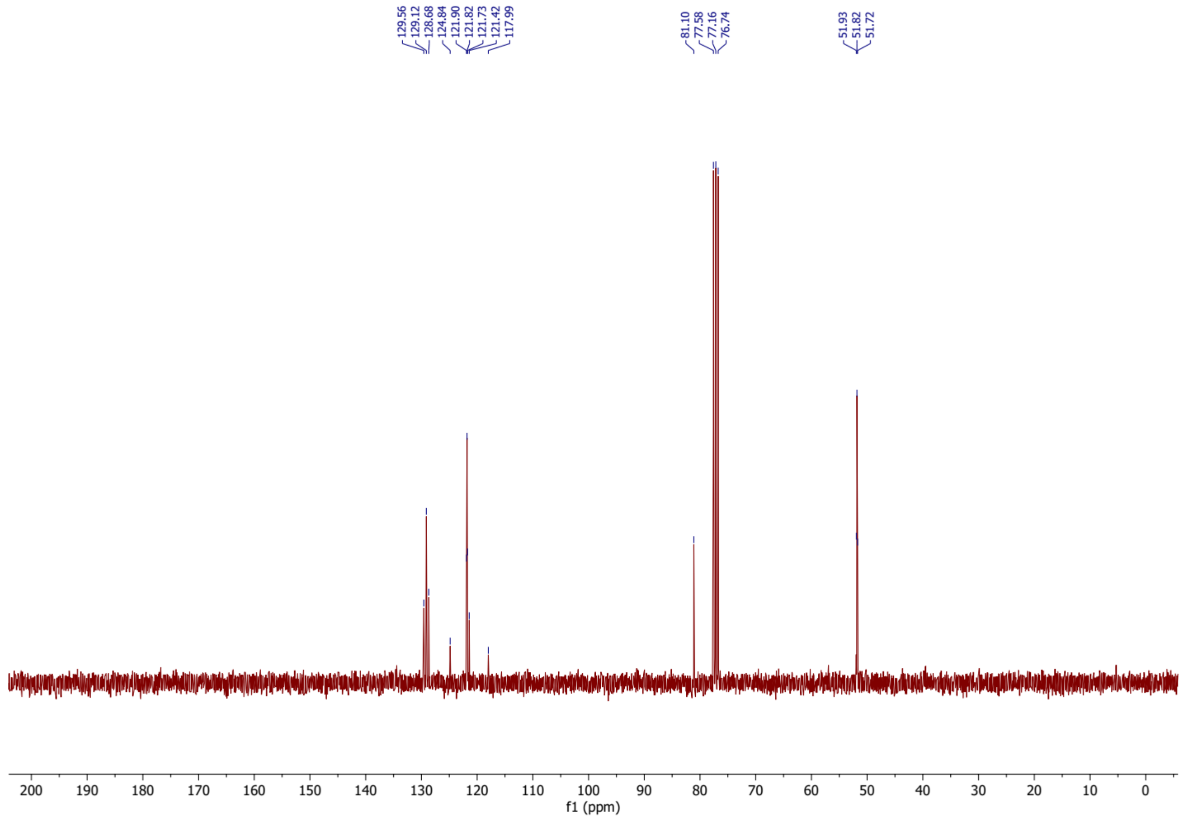

Supplementary Figure 102. ^{13}C NMR spectra of compound 25

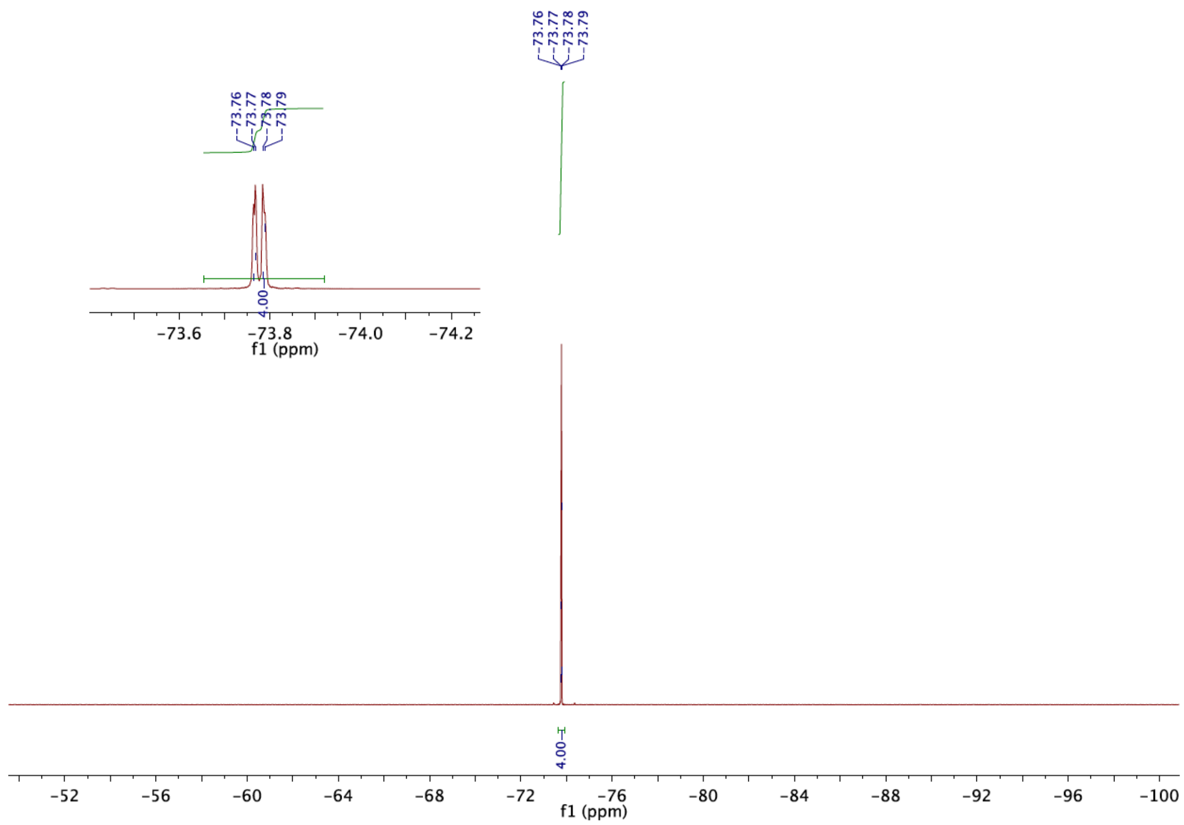

Supplementary Figure 103. ^{19}F NMR spectra of compound 25

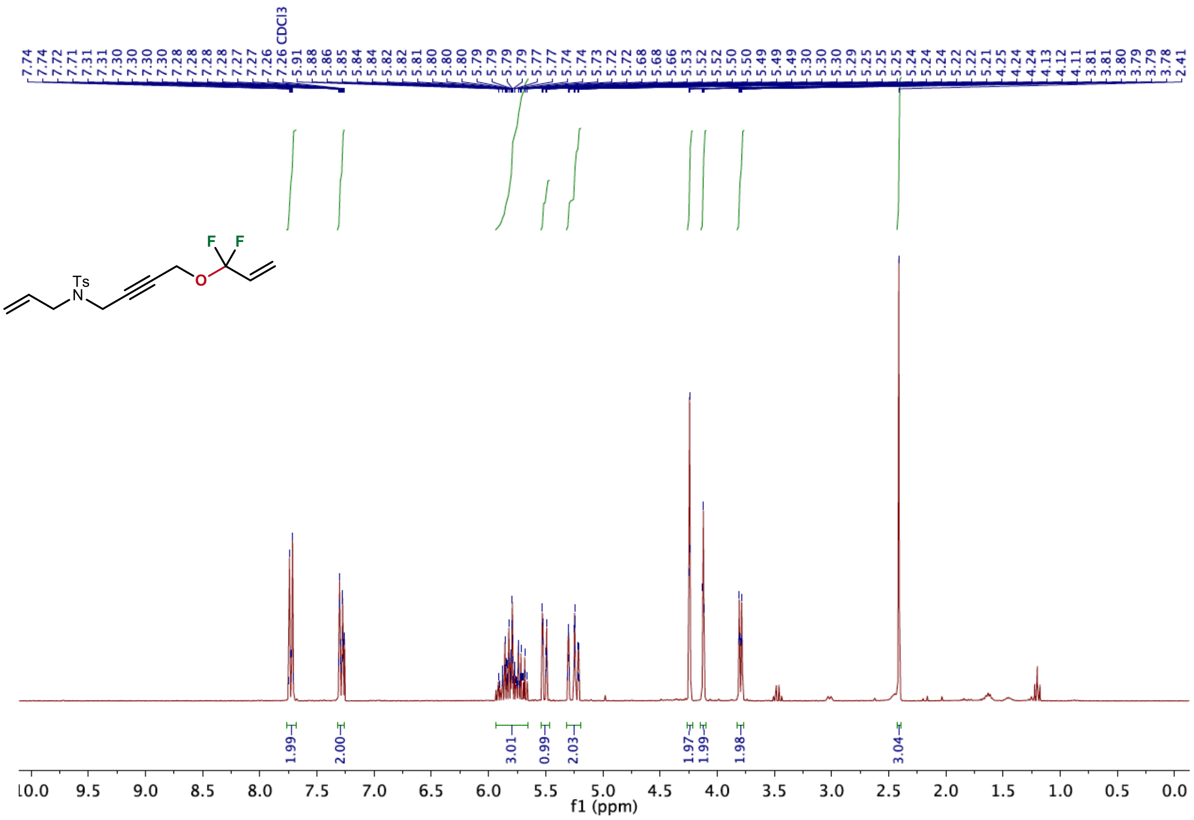

Supplementary Figure 104. ^{1}H NMR spectra of compound 26

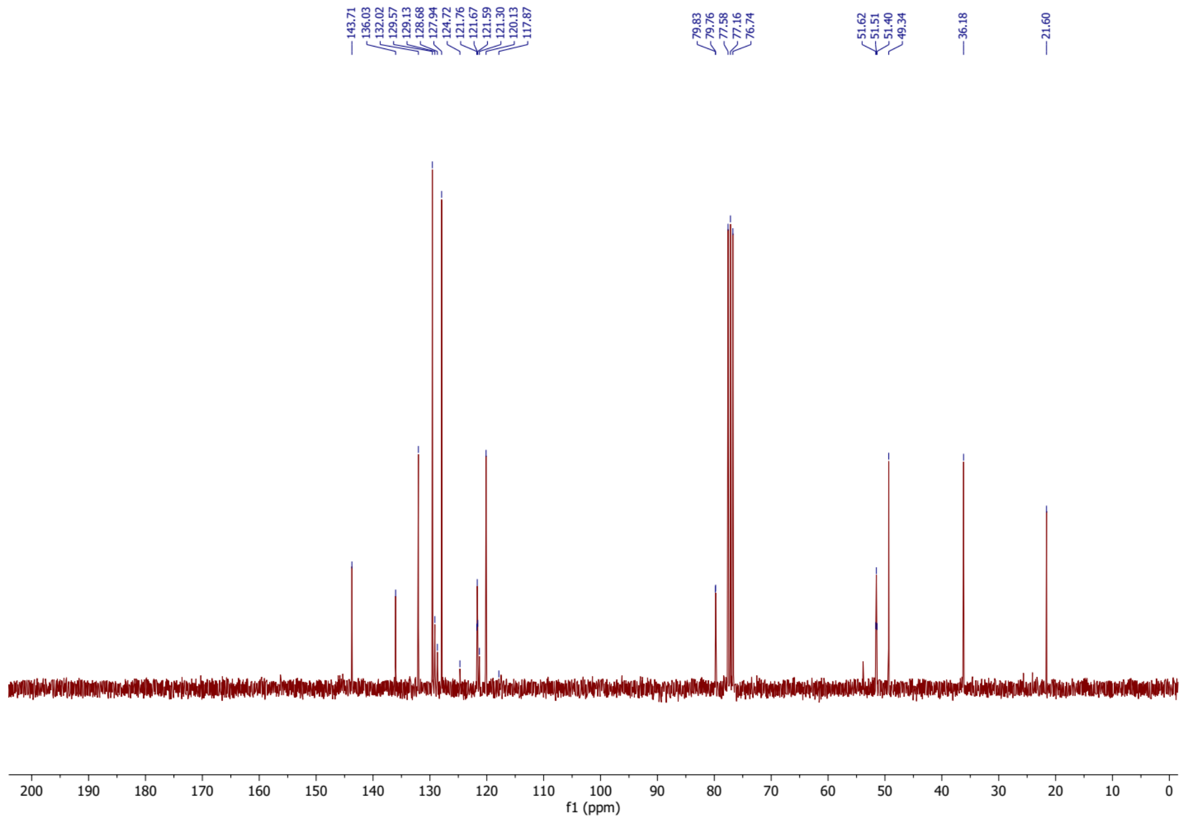

Supplementary Figure 105. ^{13}C NMR spectra of compound 26

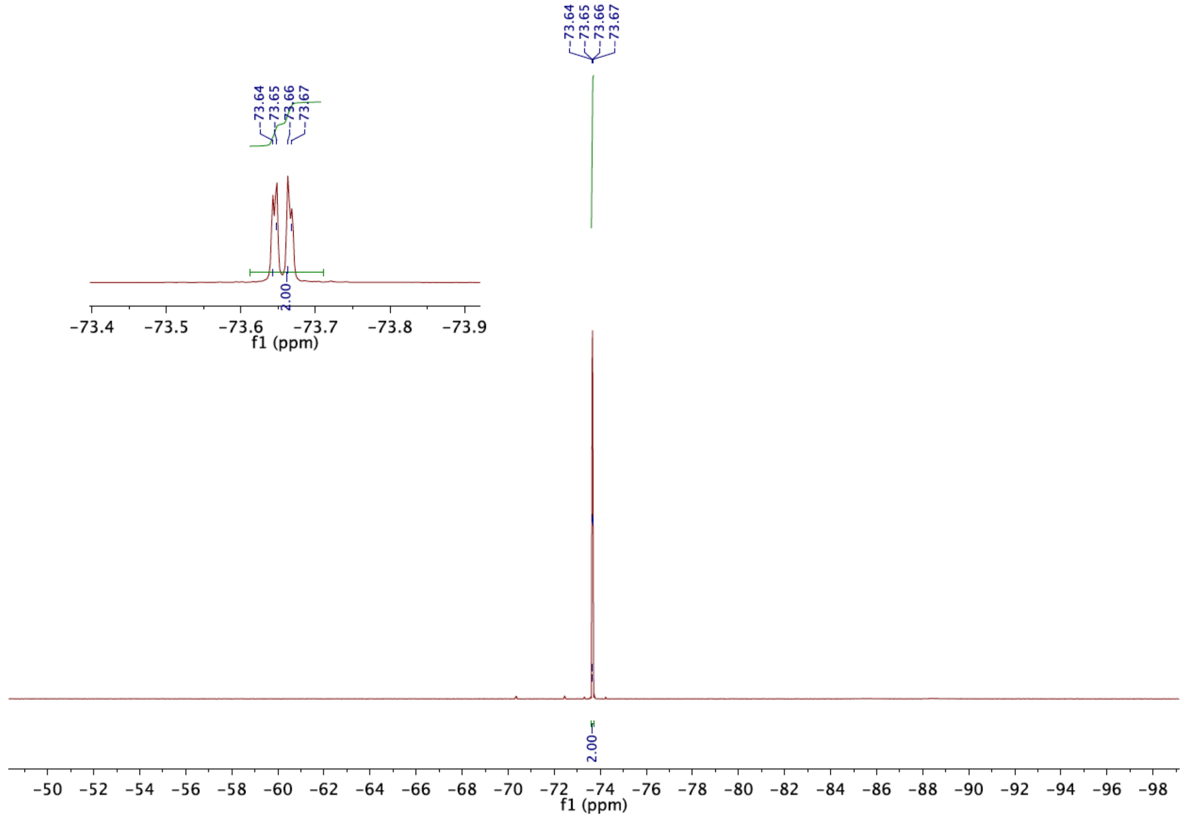

Supplementary Figure 106. ^{19}C NMR spectra of compound 26

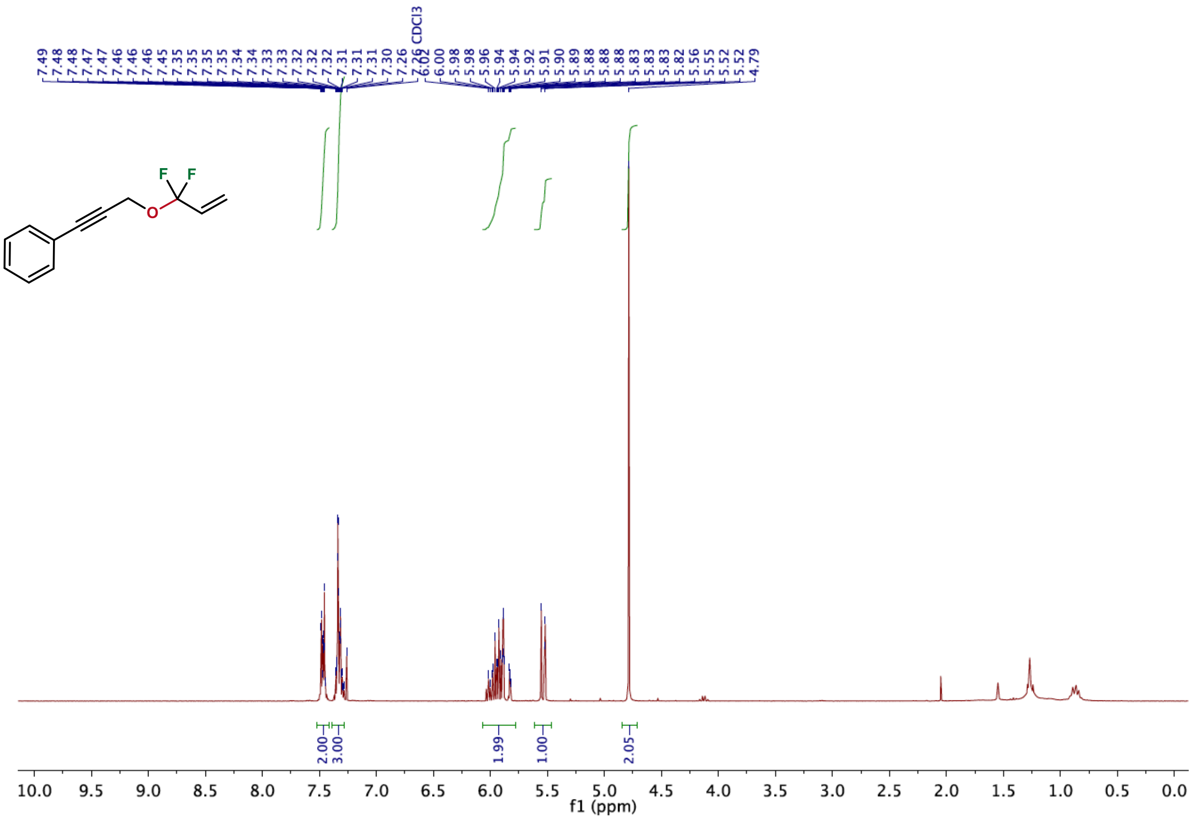

Supplementary Figure 107. ^{1}H NMR spectra of compound 27

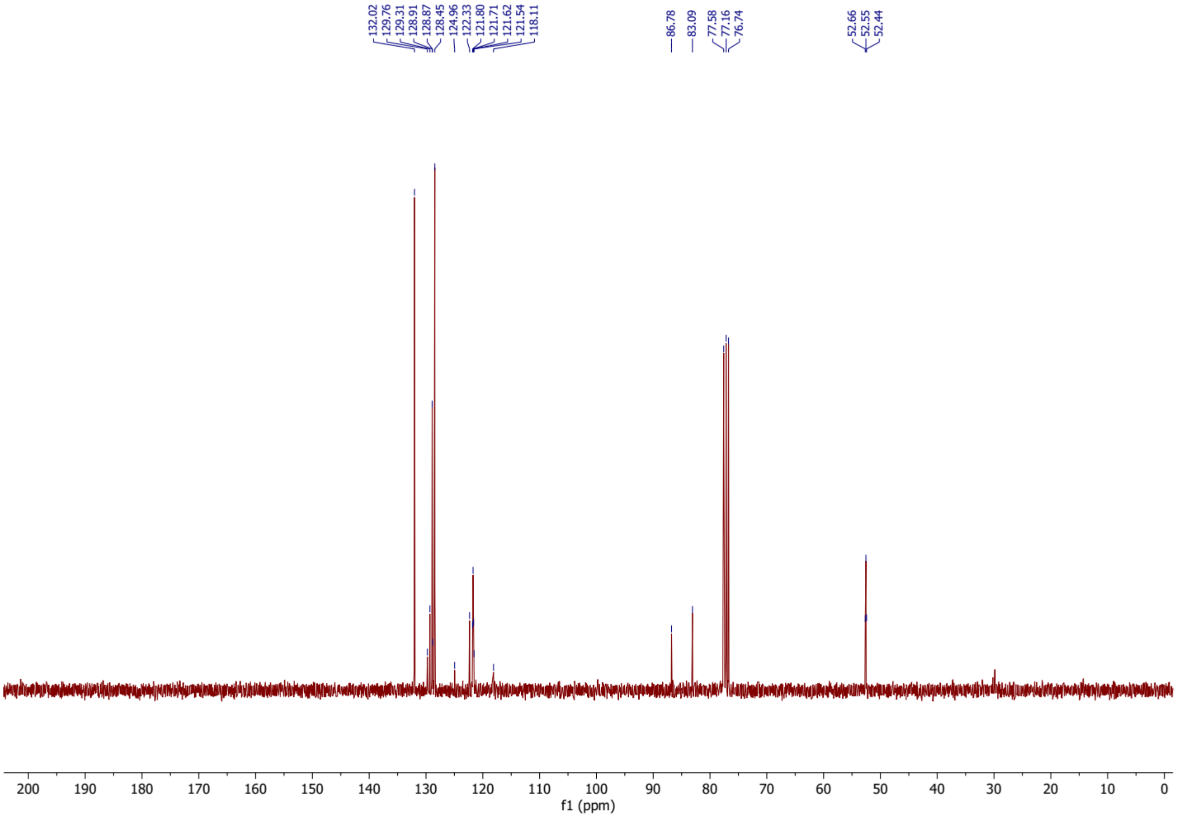

Supplementary Figure 108. ^{13}C NMR spectra of compound 27

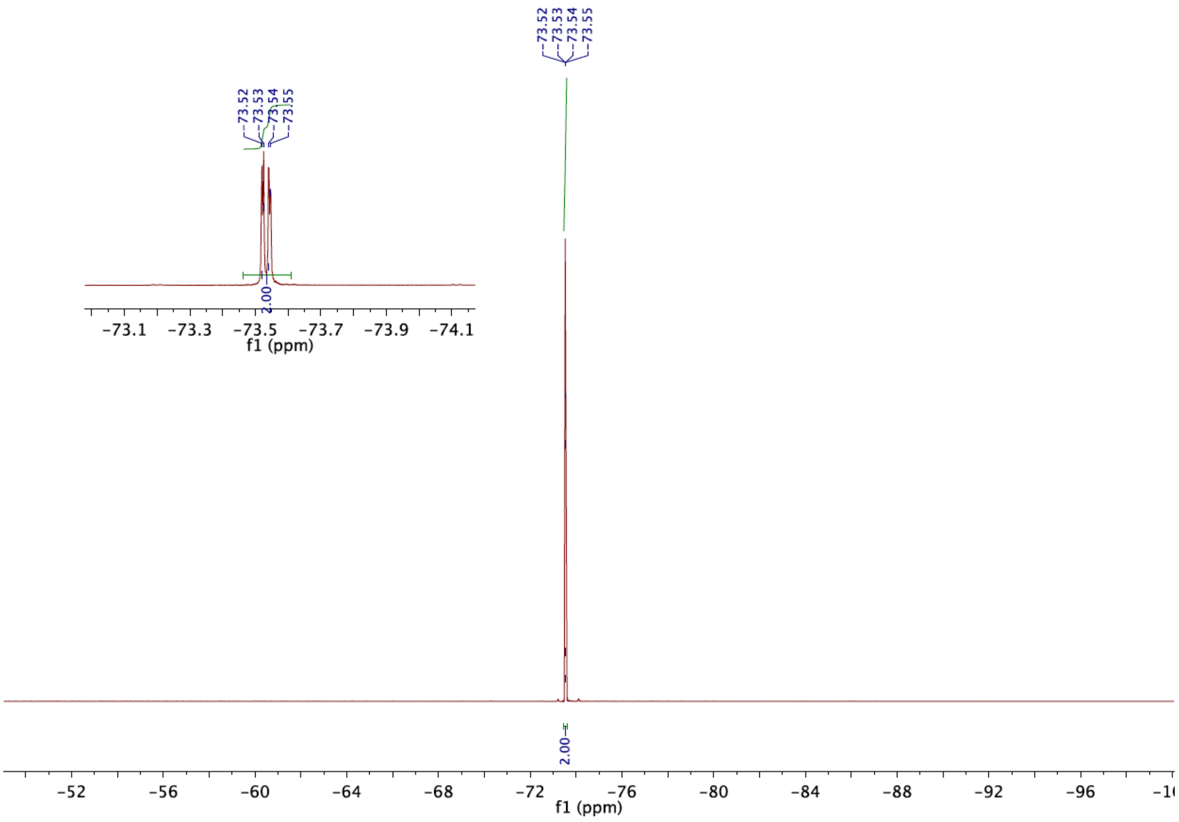

Supplementary Figure 109. ^{19}F NMR spectra of compound 27

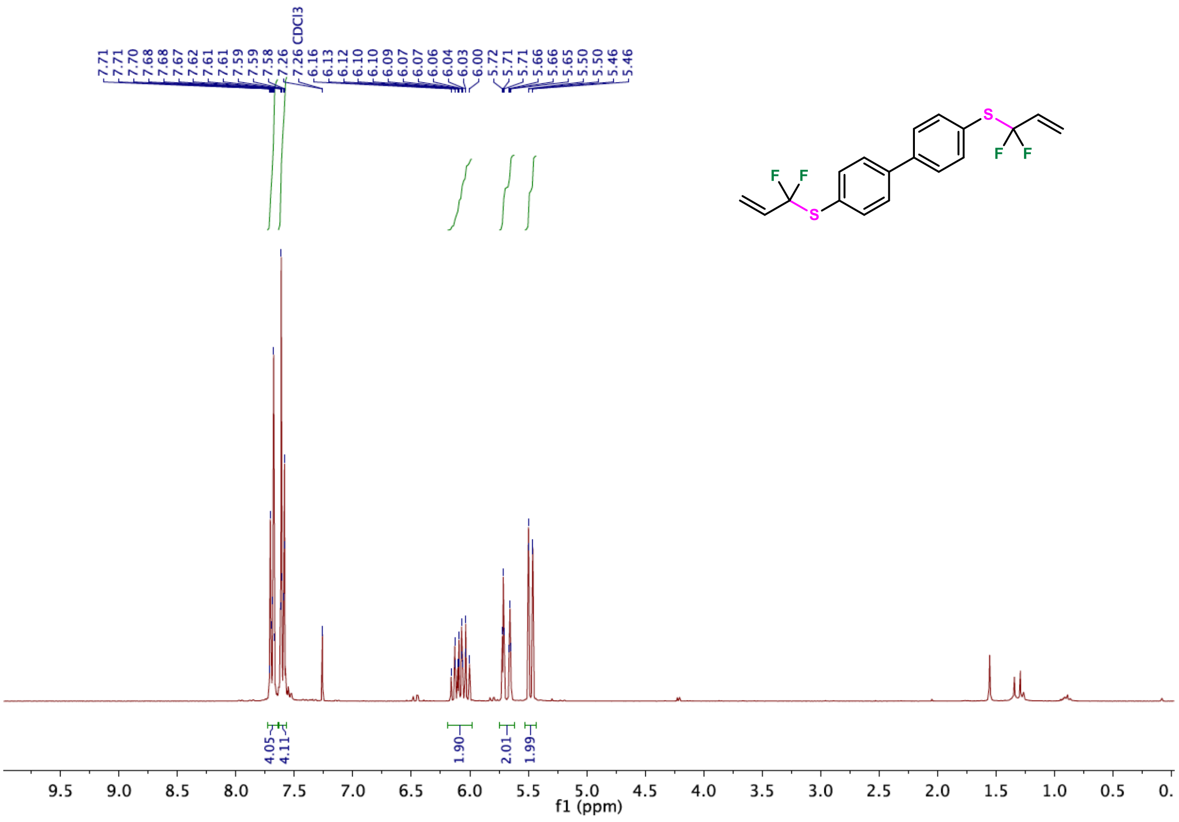

Supplementary Figure 110. ^{1}H NMR spectra of compound 28

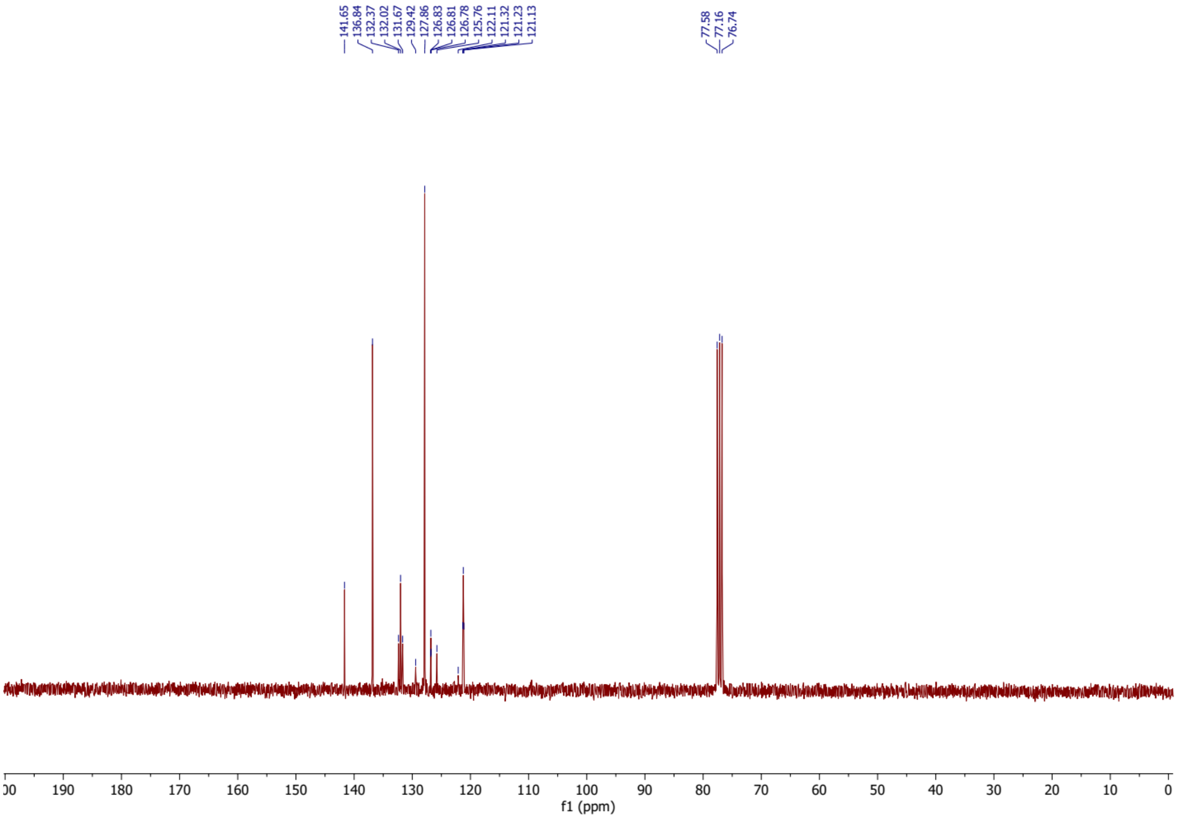

Supplementary Figure 111. ^{13}C NMR spectra of compound 28

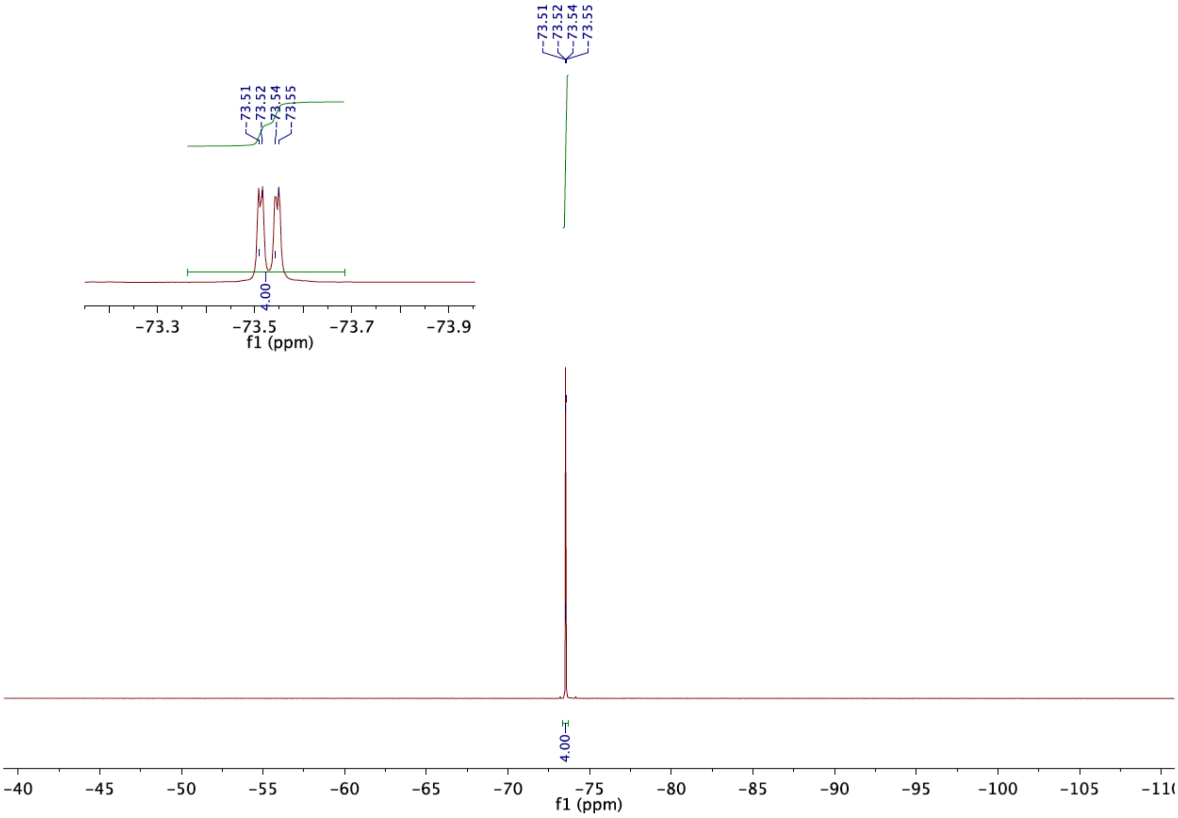

Supplementary Figure 112. ^{19}F NMR spectra of compound 28

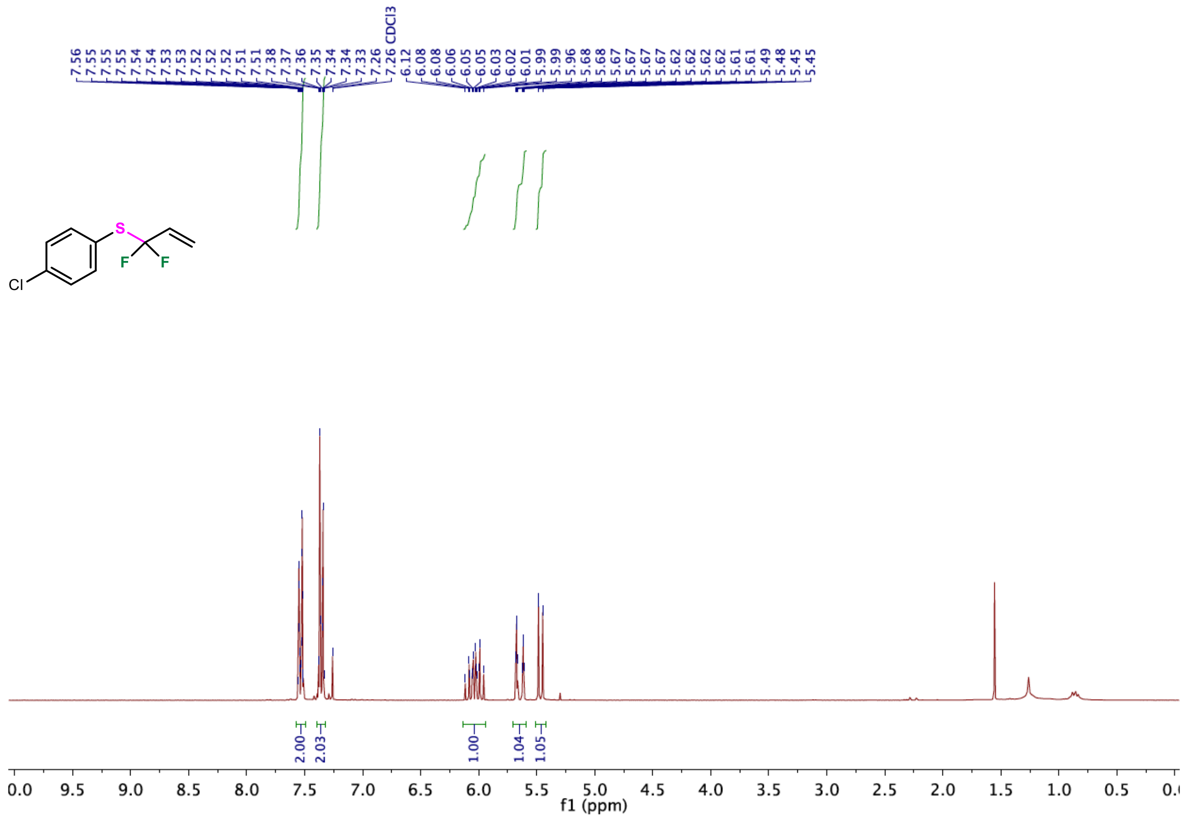

Supplementary Figure 113. ^{1}H NMR spectra of compound 29

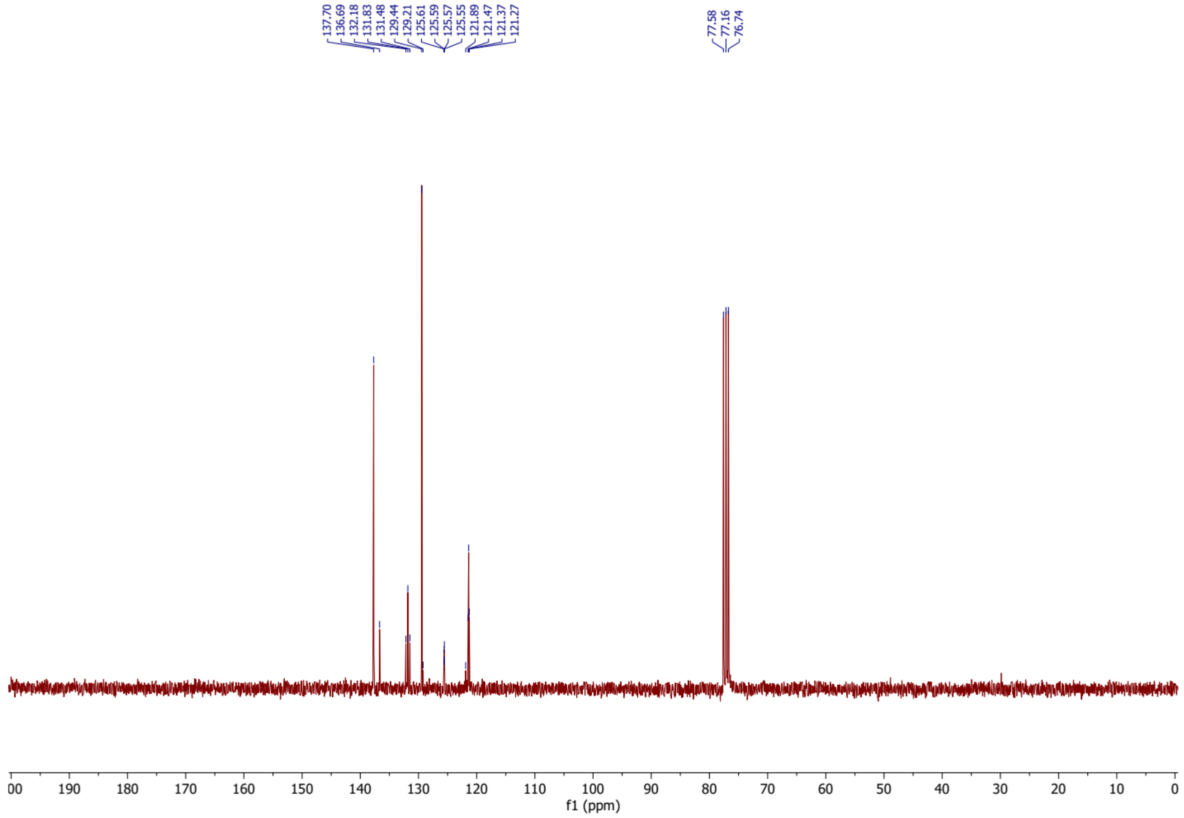

Supplementary Figure 114. ^{13}C NMR spectra of compound 29

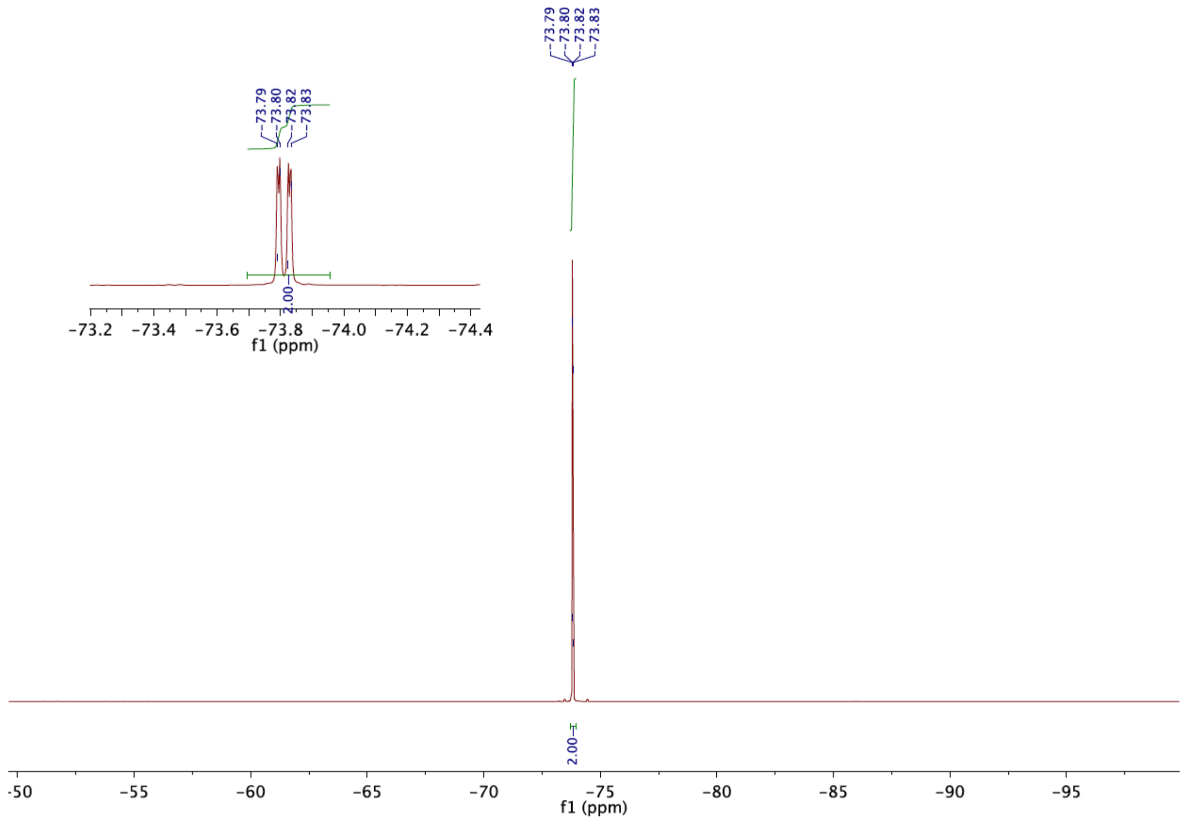

Supplementary Figure 115. ^{19}F NMR spectra of compound 29

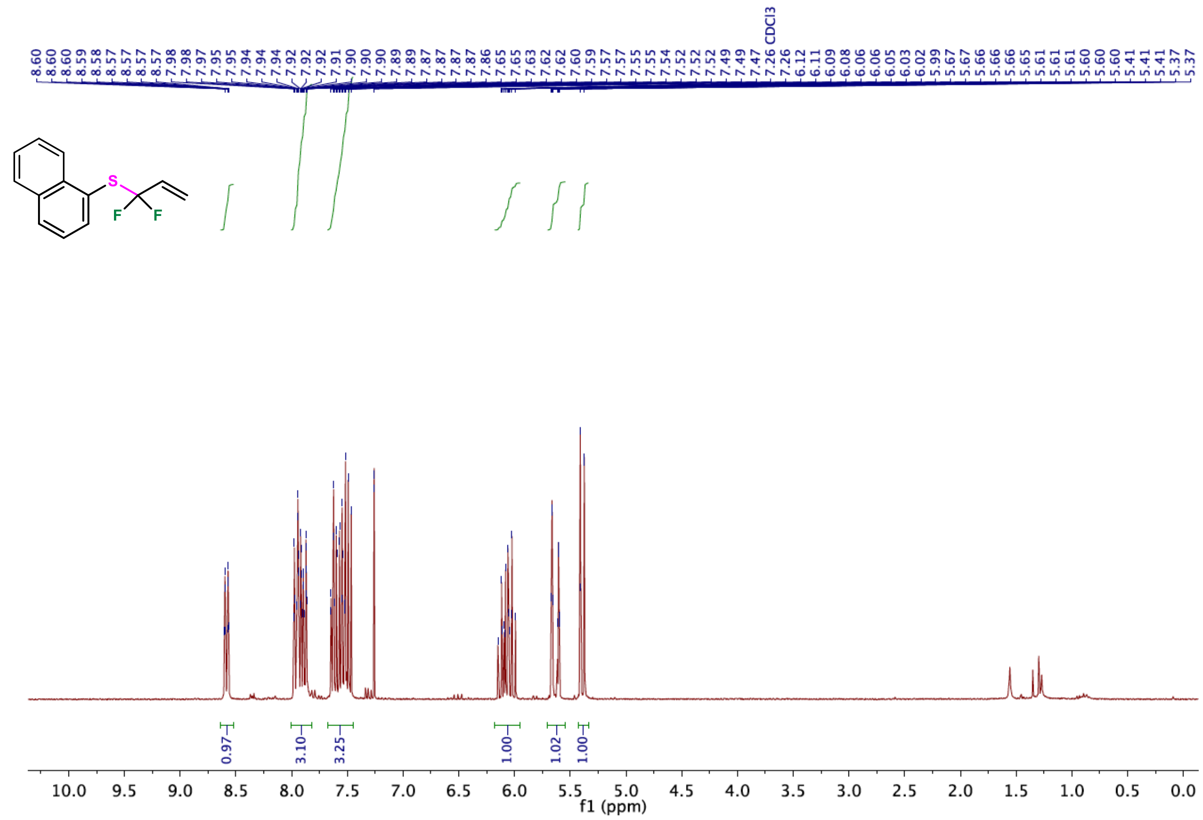

Supplementary Figure 116. ^{1}H NMR spectra of compound 30

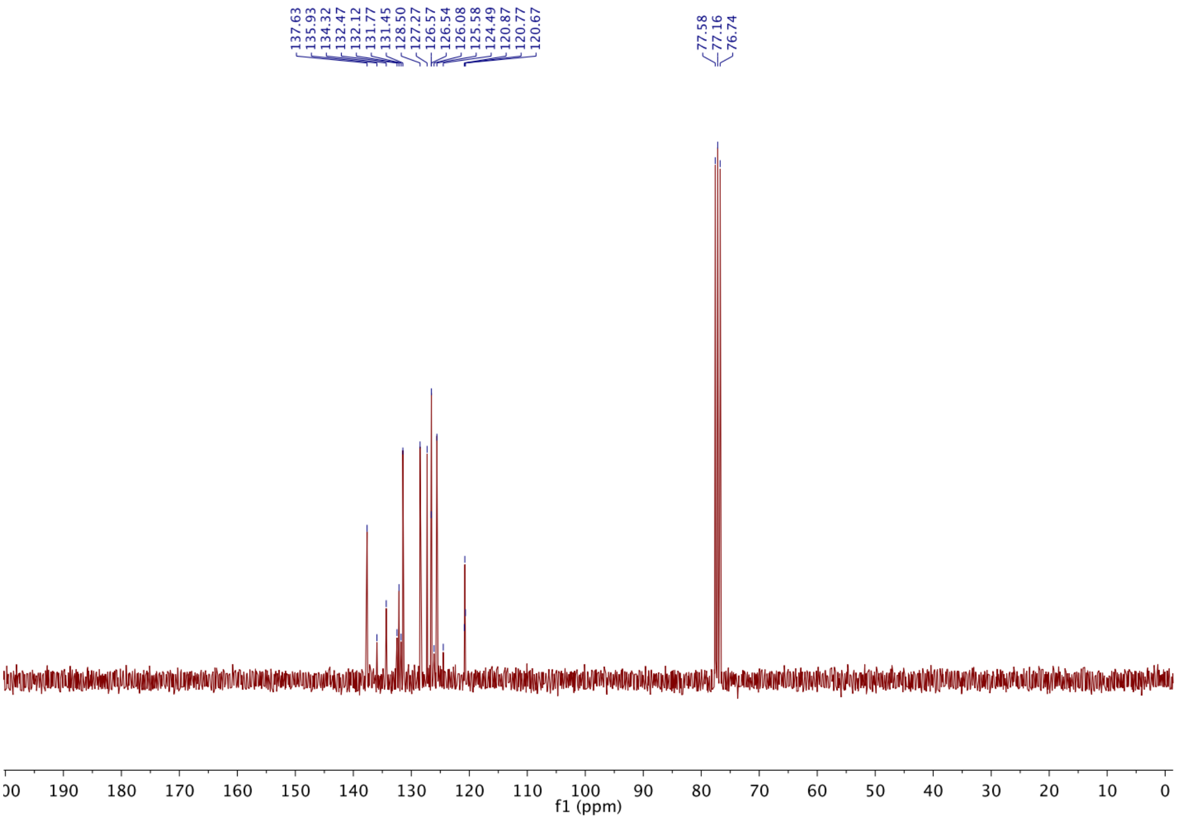

Supplementary Figure 117. ^{13}C NMR spectra of compound 30

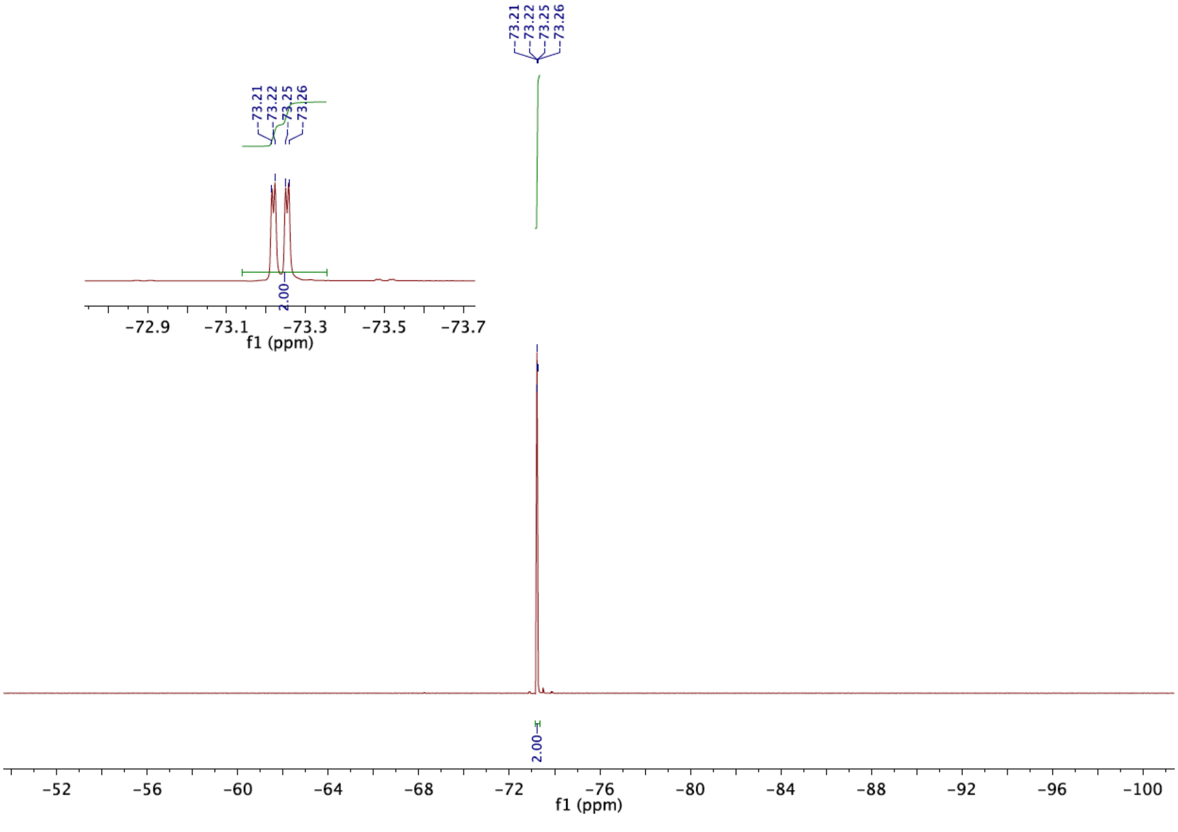

Supplementary Figure 118. ^{19}F NMR spectra of compound 30

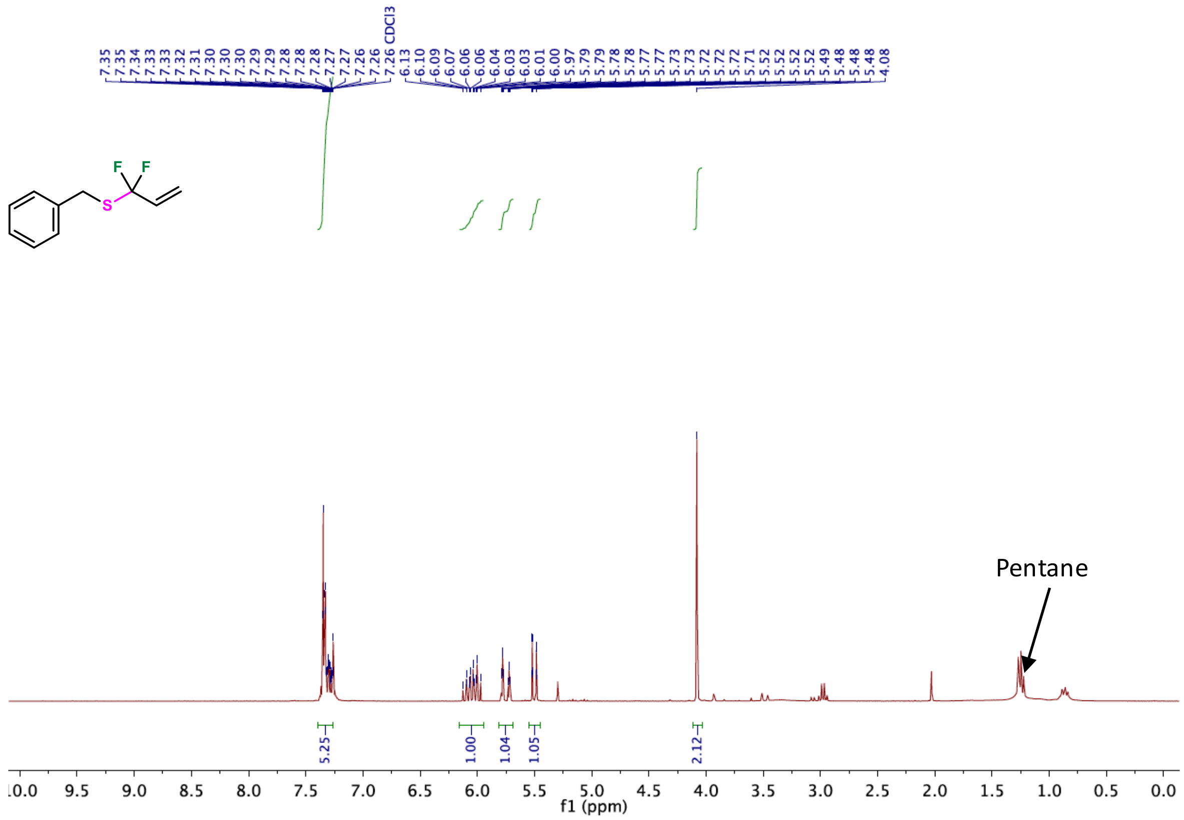

Supplementary Figure 119. ^{1}H NMR spectra of compound 31

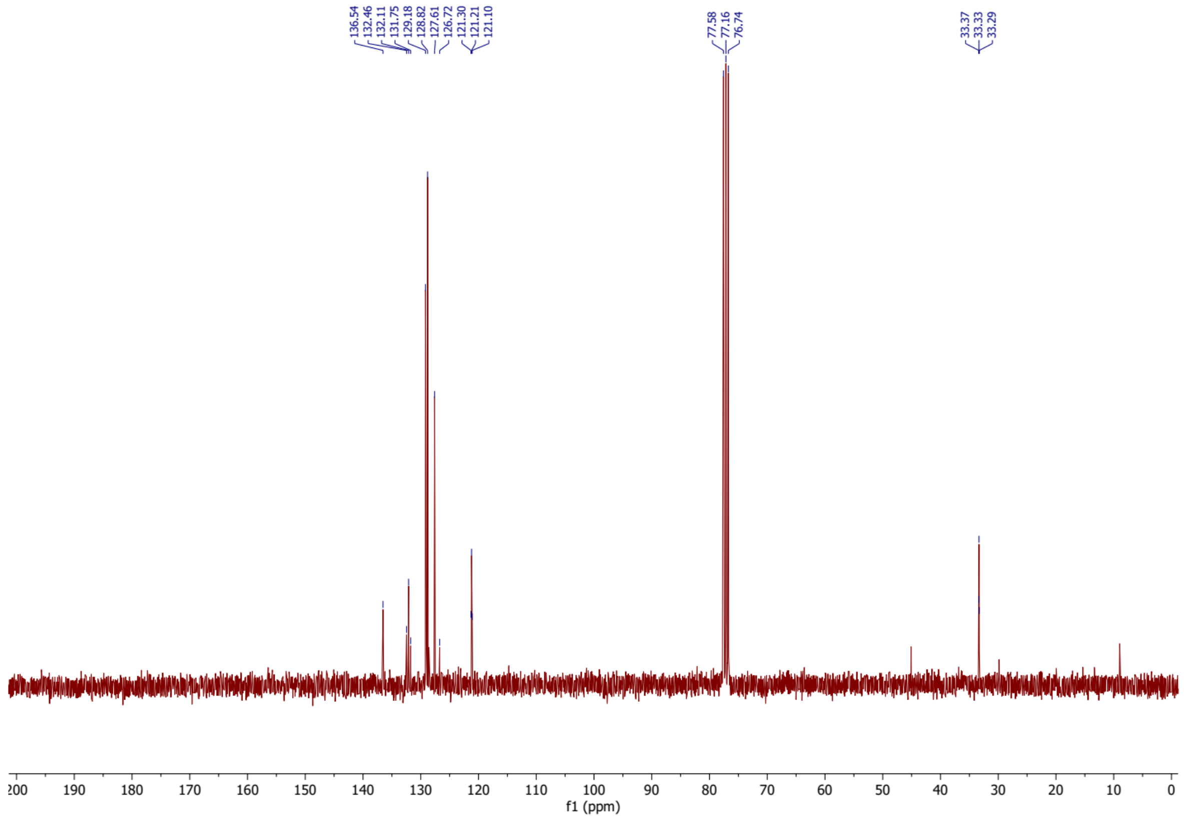

Supplementary Figure 120. ^{13}C NMR spectra of compound 31

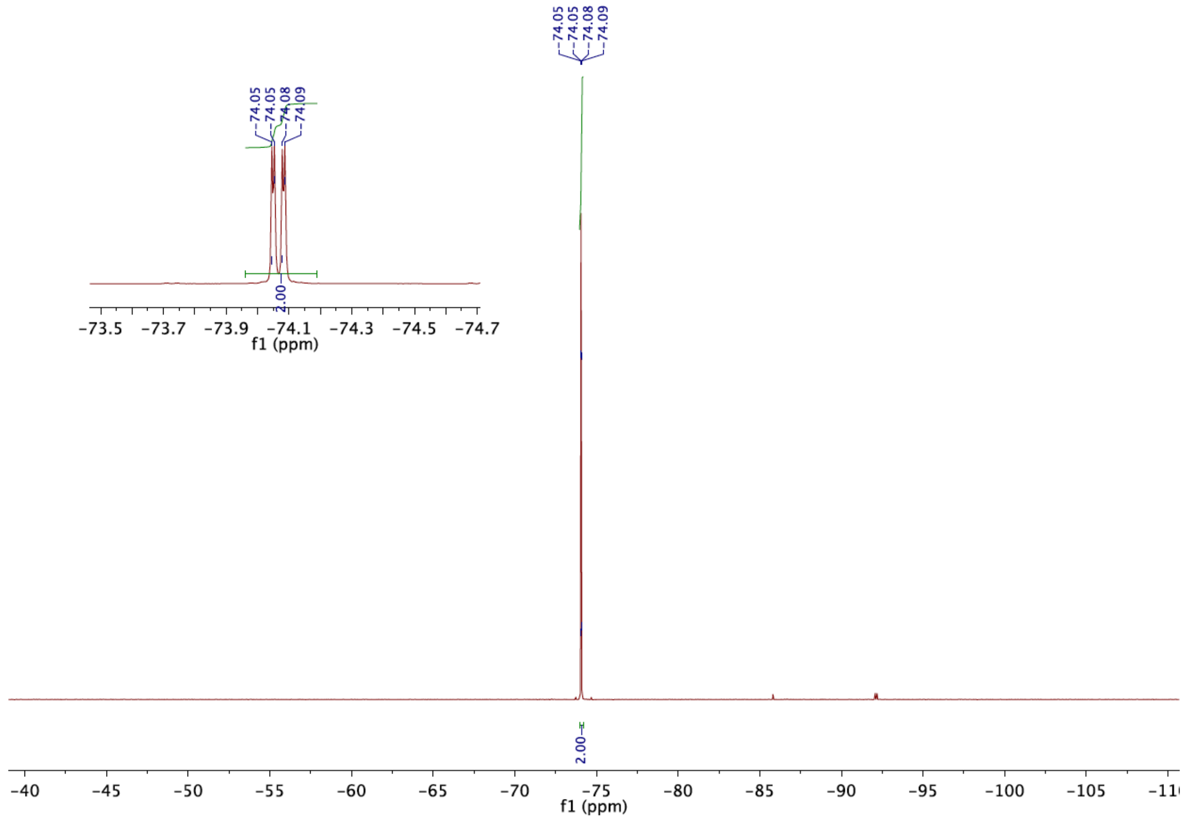

Supplementary Figure 121. ^{19}F NMR spectra of compound 31

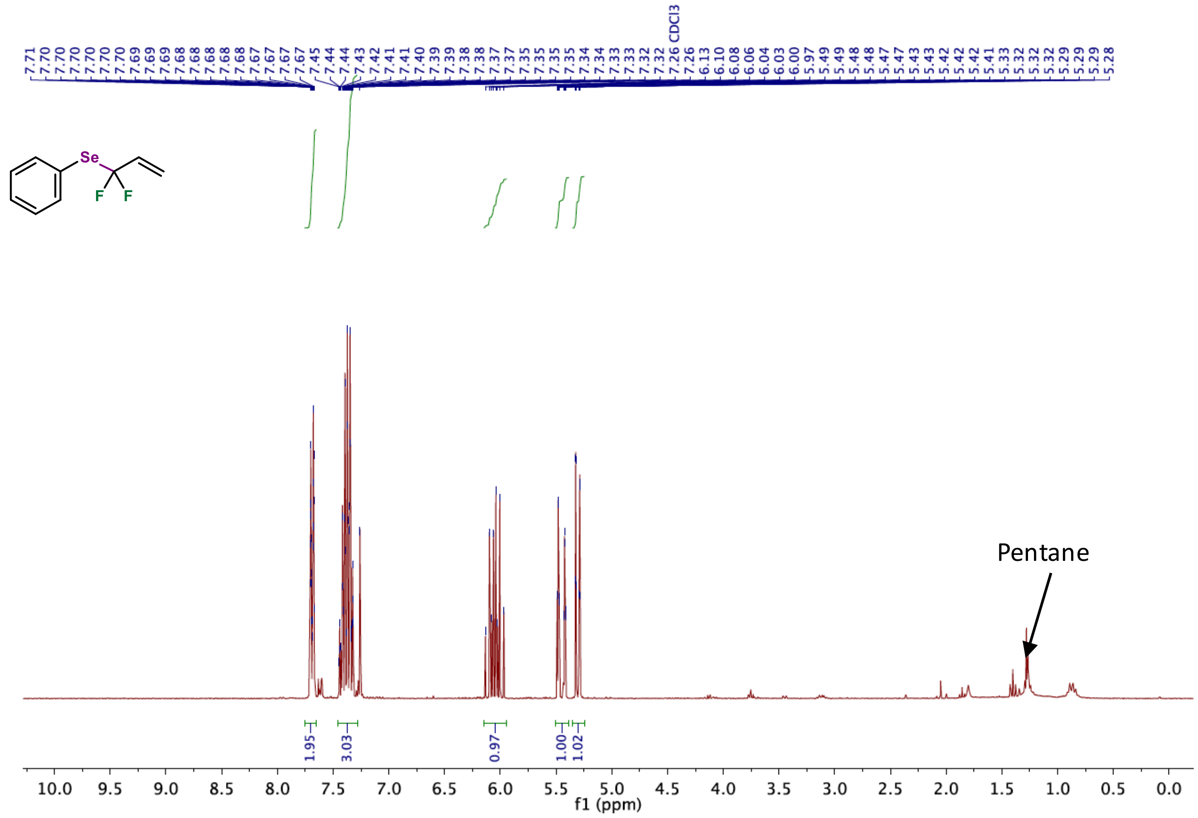

Supplementary Figure 122. ^{1}H NMR spectra of compound 32

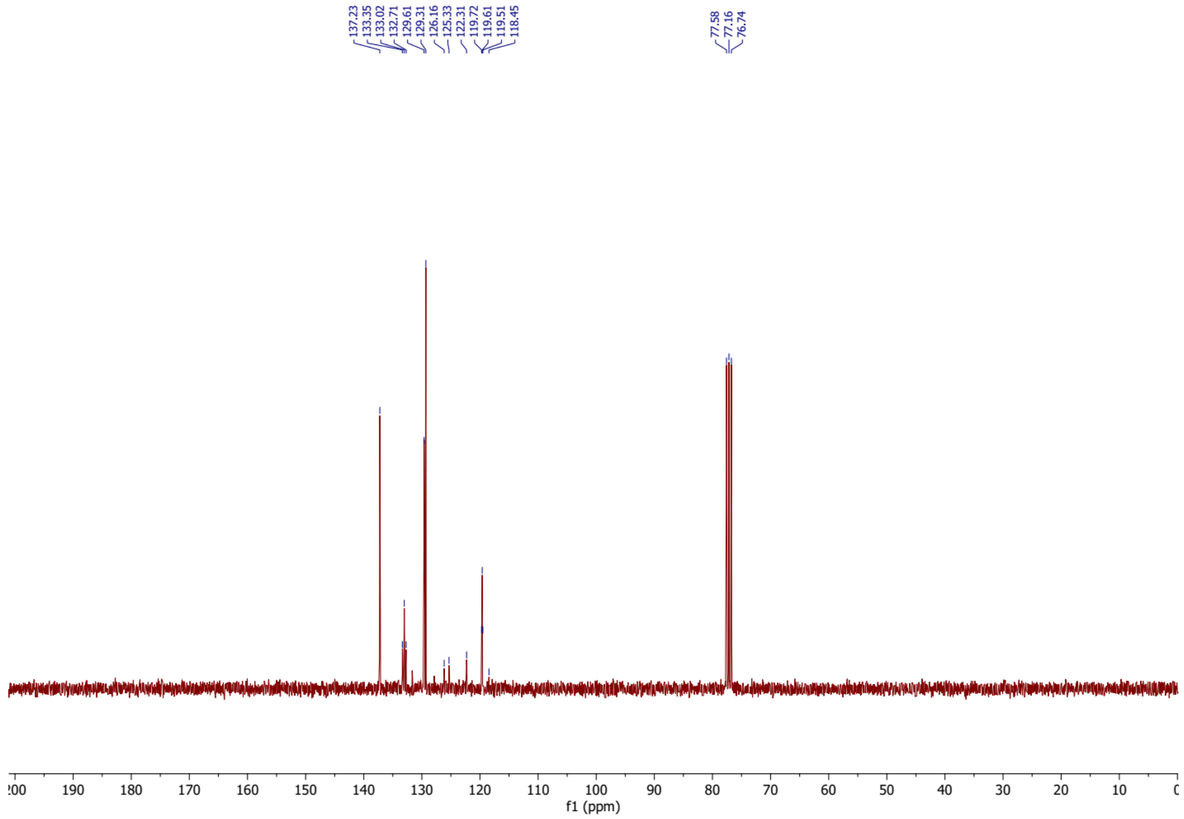

Supplementary Figure 123. ^{13}C NMR spectra of compound 32

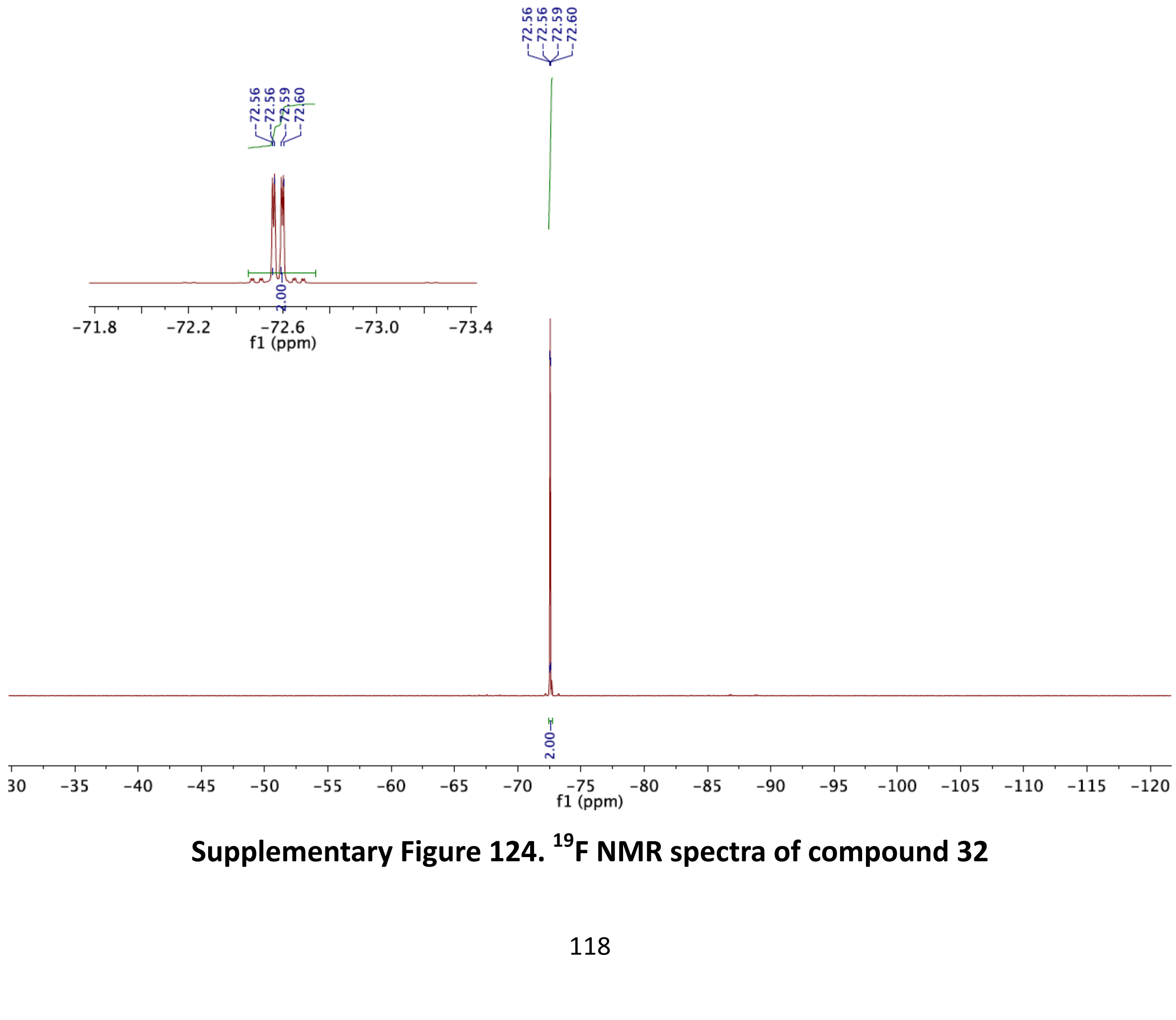

Supplementary Figure 124. ^{19}F NMR spectra of compound 32

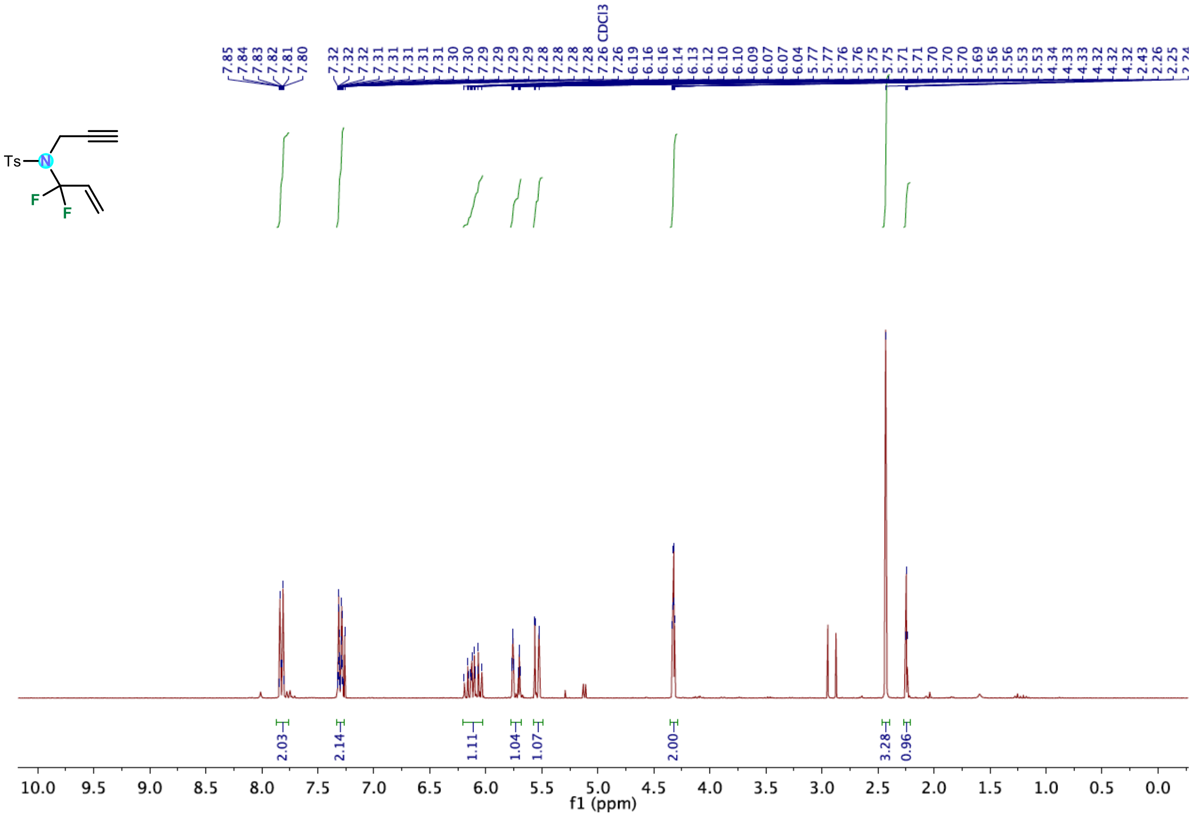

Supplementary Figure 125. ^{1}H NMR spectra of compound 33

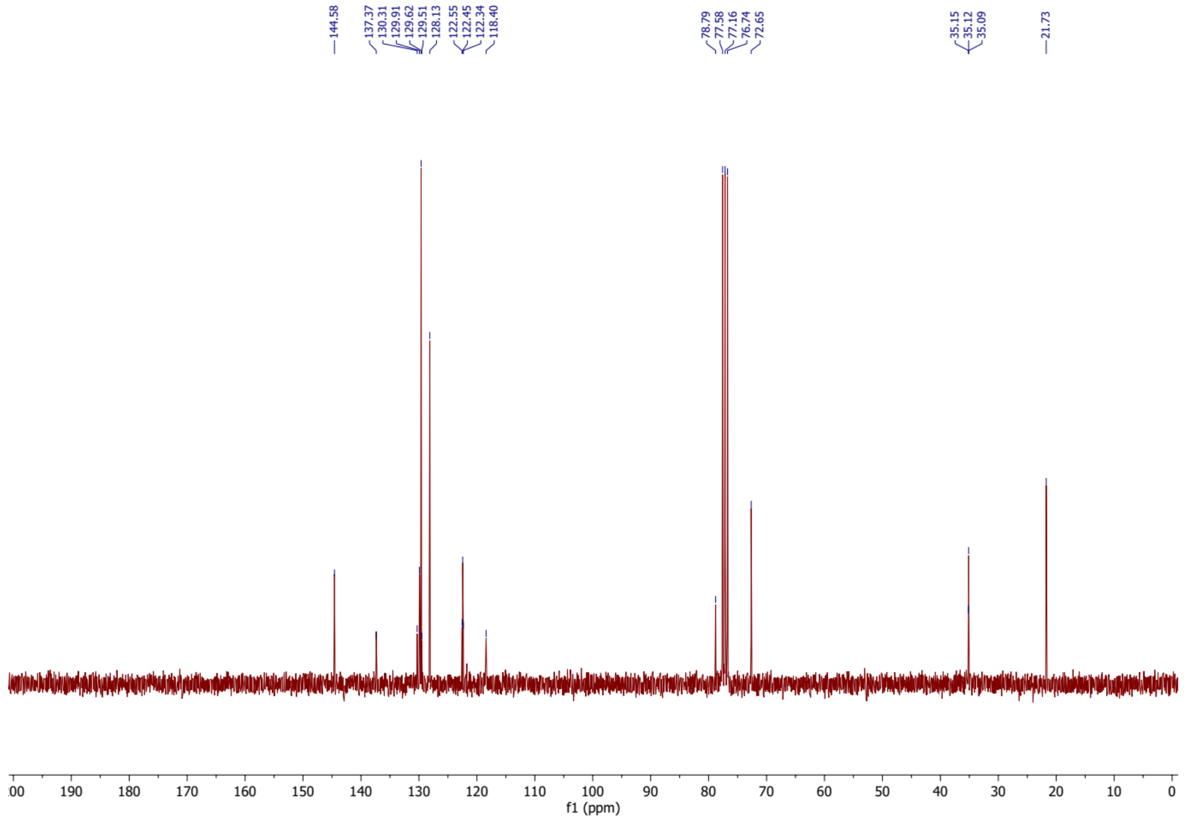

Supplementary Figure 126. ^{13}C NMR spectra of compound 33

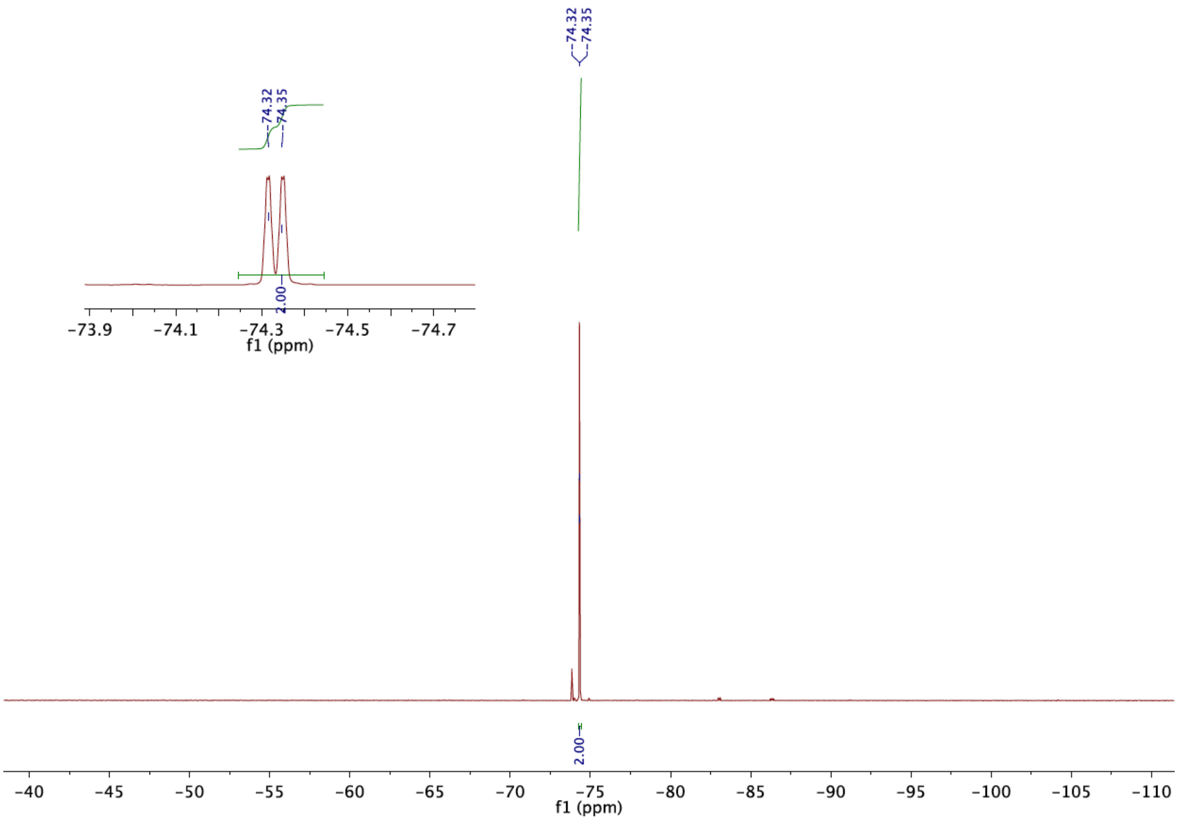

Supplementary Figure 127. ^{19}F NMR spectra of compound 33

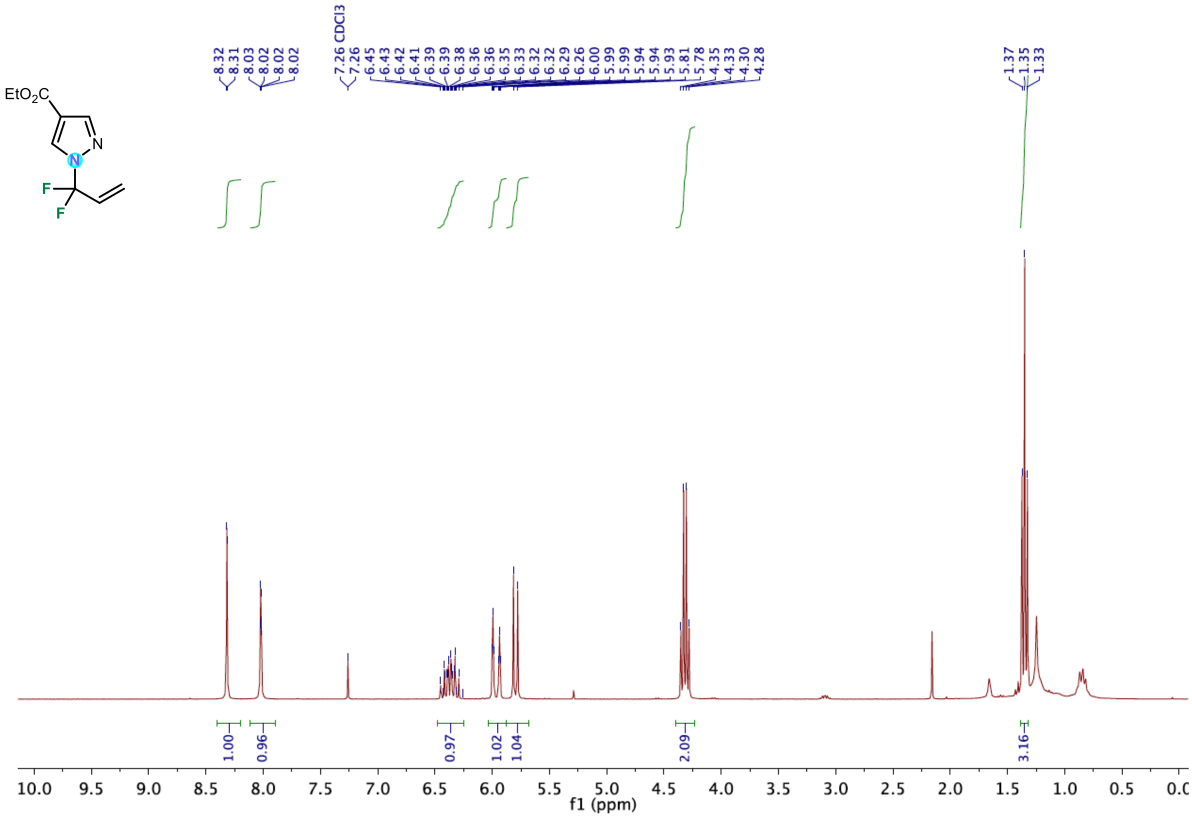

Supplementary Figure 128. ^{1}H NMR spectra of compound 34

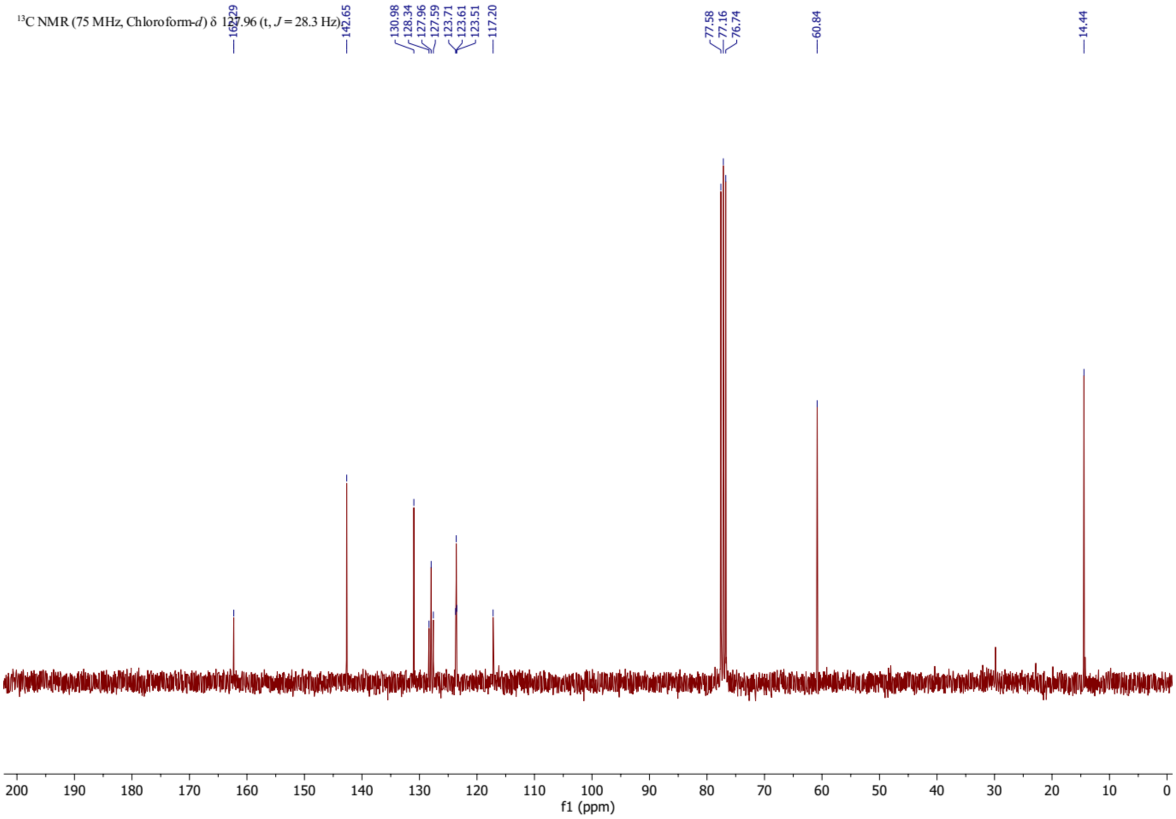

Supplementary Figure 129. ^{13}C NMR spectra of compound 34

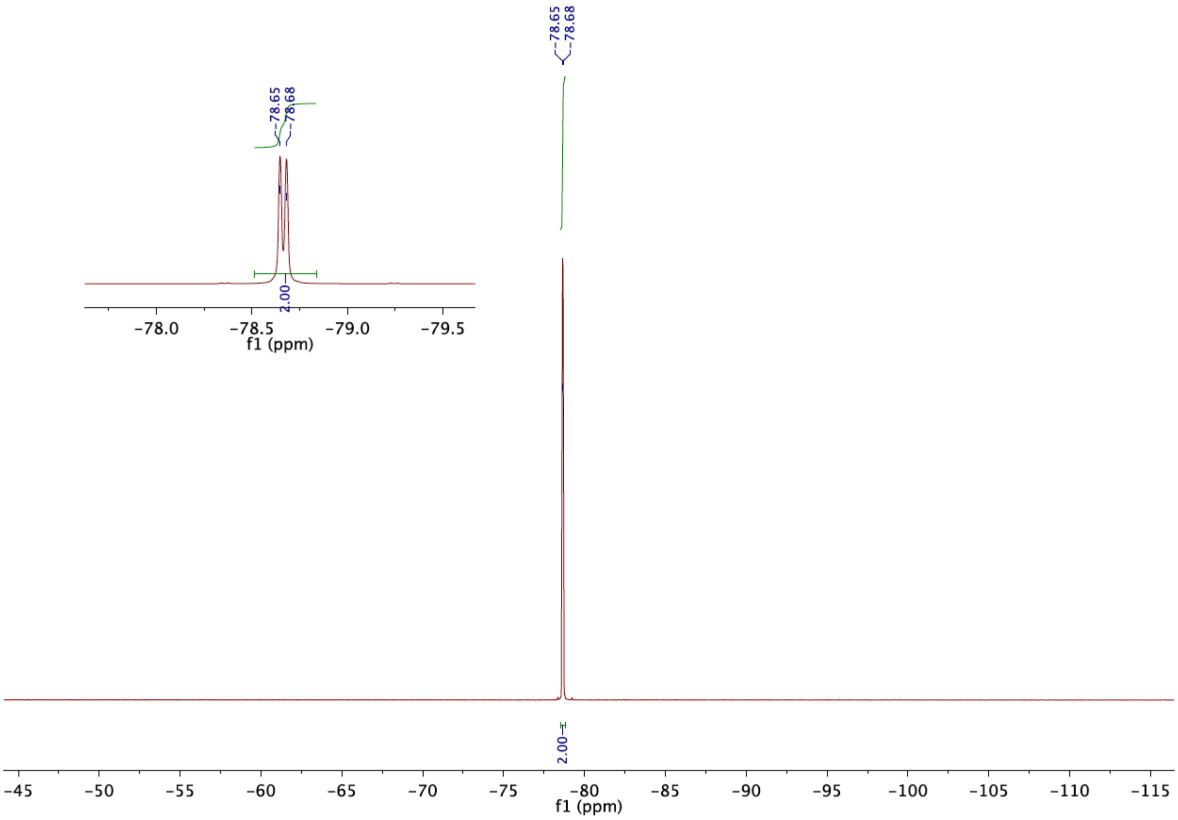

Supplementary Figure 130. ^{19}F NMR spectra of compound 34

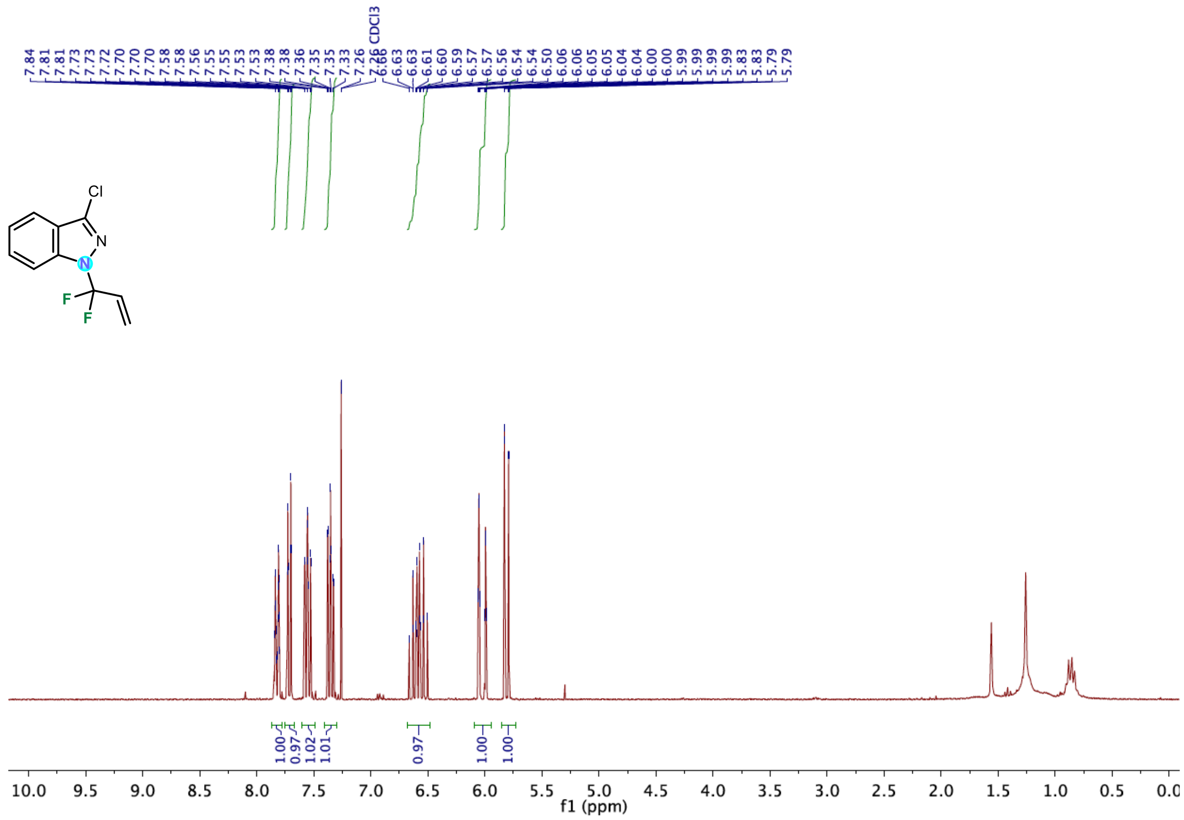

Supplementary Figure 131. ^{1}H NMR spectra of compound 35

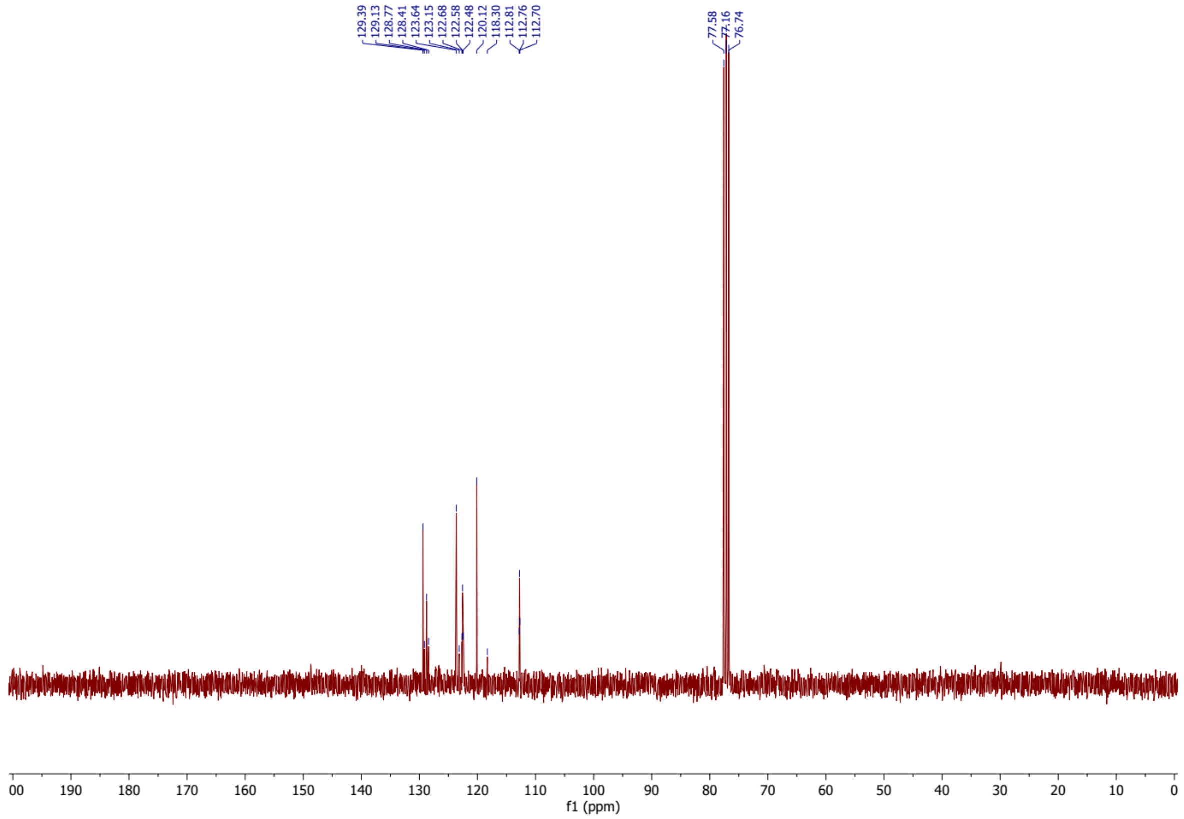

Supplementary Figure 132. ^{13}C NMR spectra of compound 35

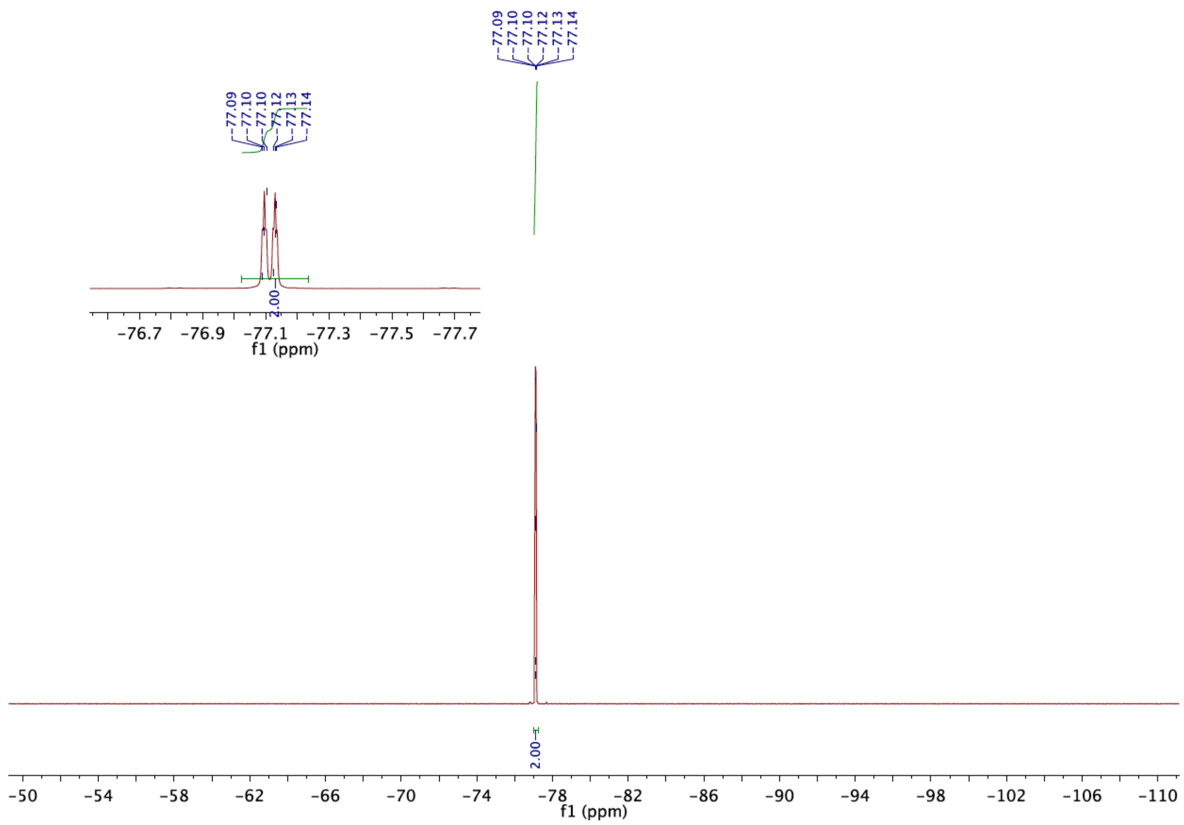

Supplementary Figure 133. ^{19}F NMR spectra of compound 35

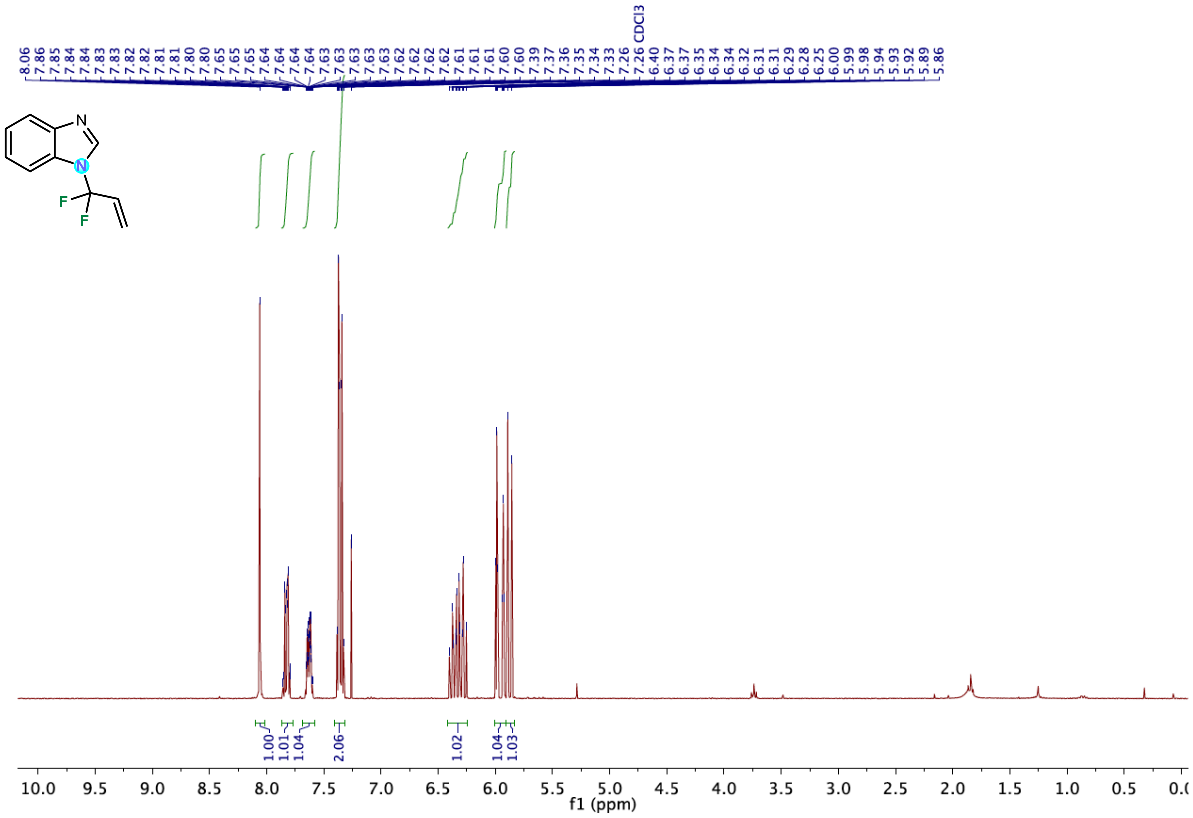

Supplementary Figure 134. ^{1}H NMR spectra of compound 36

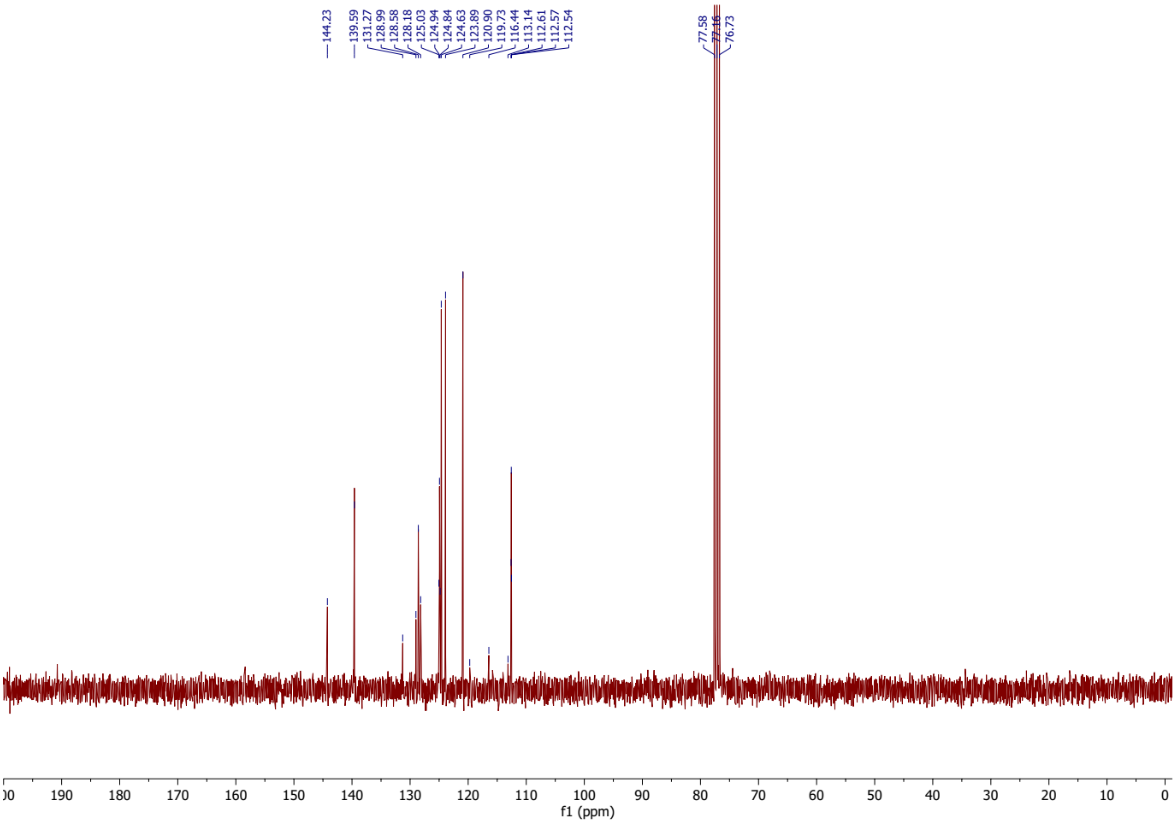

Supplementary Figure 135. ^{13}C NMR spectra of compound 36

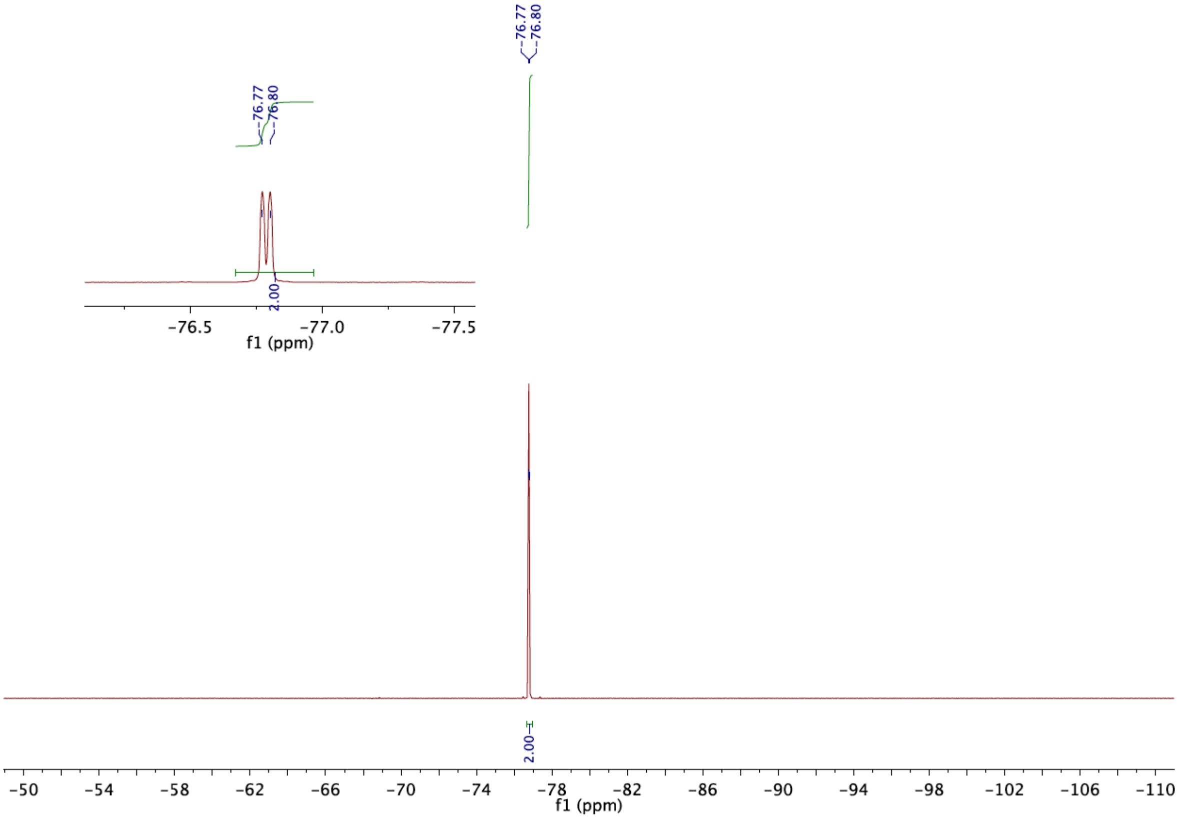

Supplementary Figure 136. ^{19}F NMR spectra of compound 36

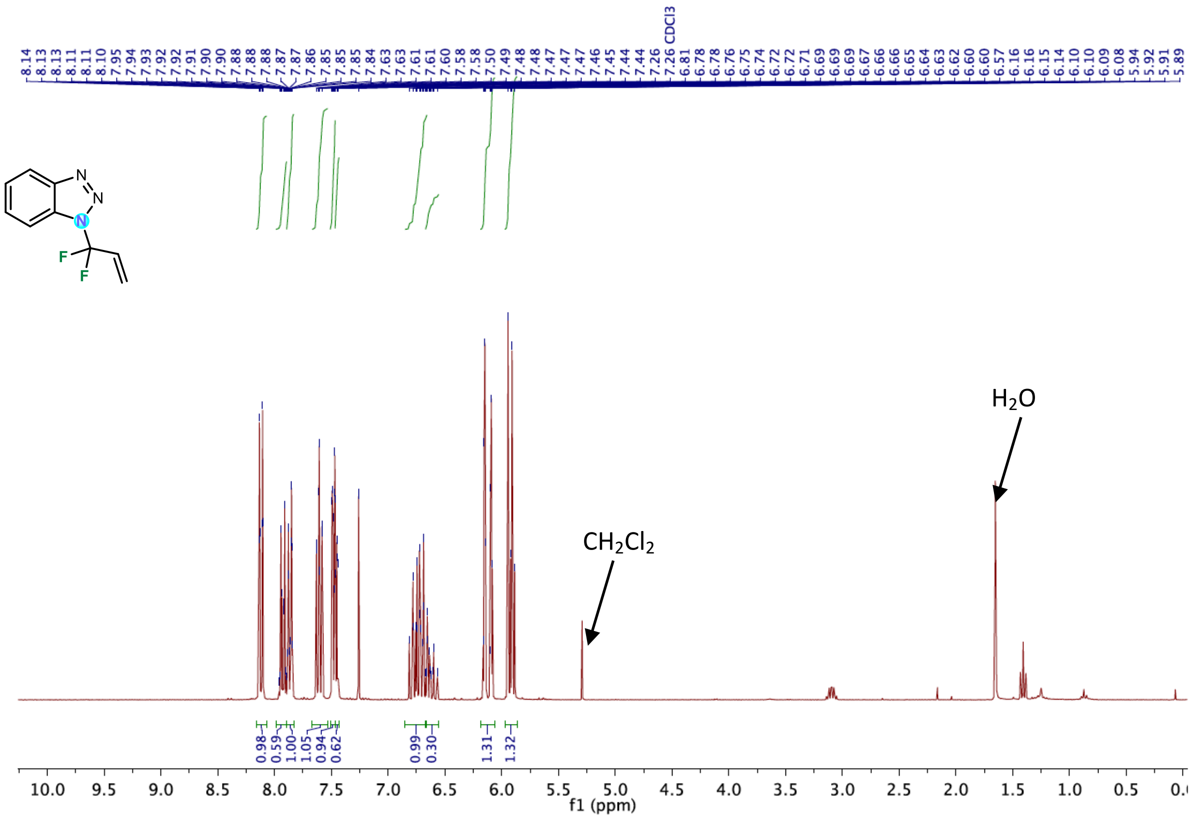

Supplementary Figure 137. ^{1}H NMR spectra of compound 37

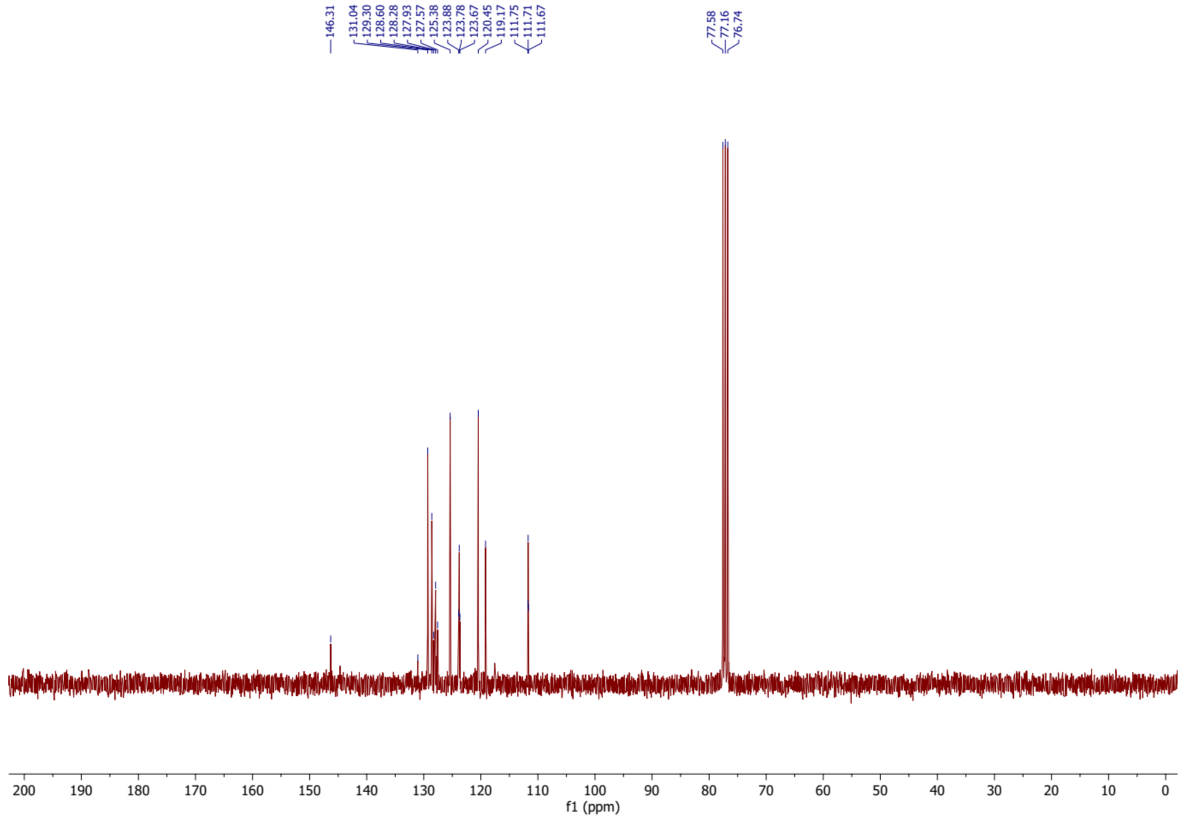

Supplementary Figure 138. ^{13}C NMR spectra of compound 37

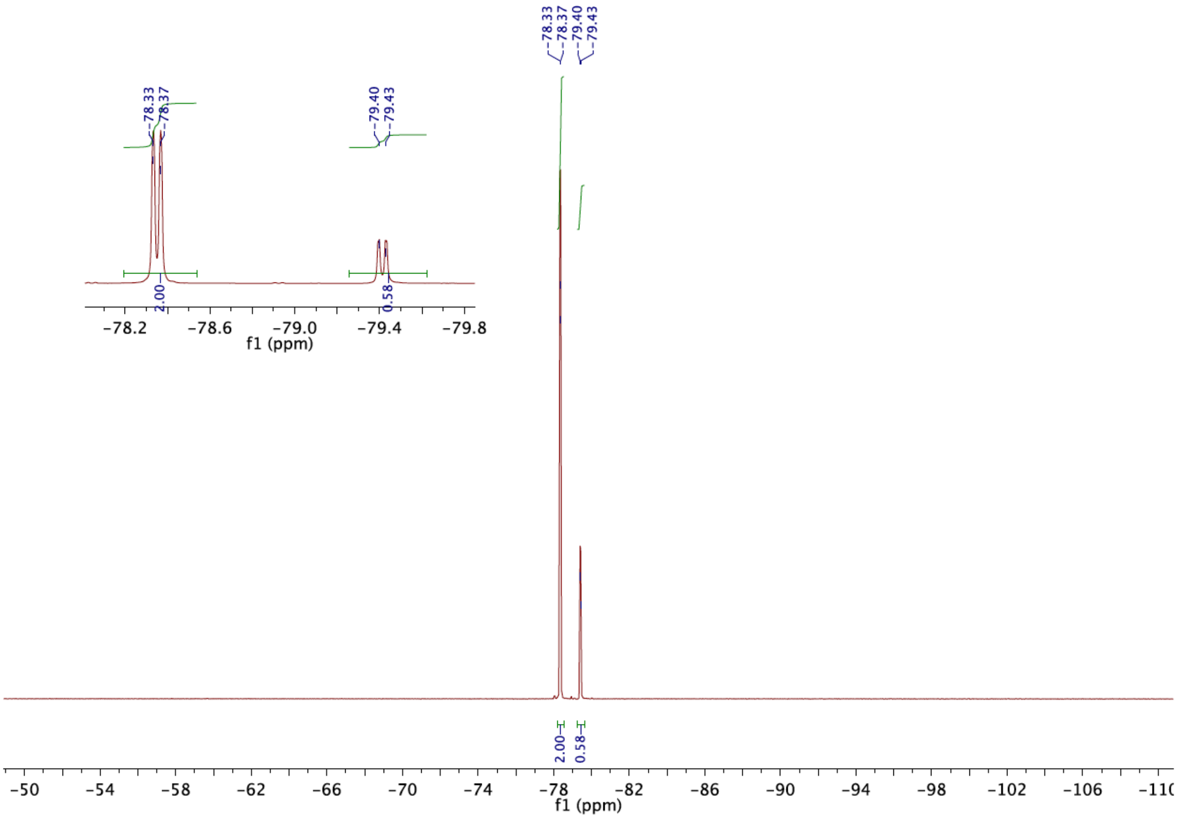

Supplementary Figure 139. ^{19}F NMR spectra of compound 37

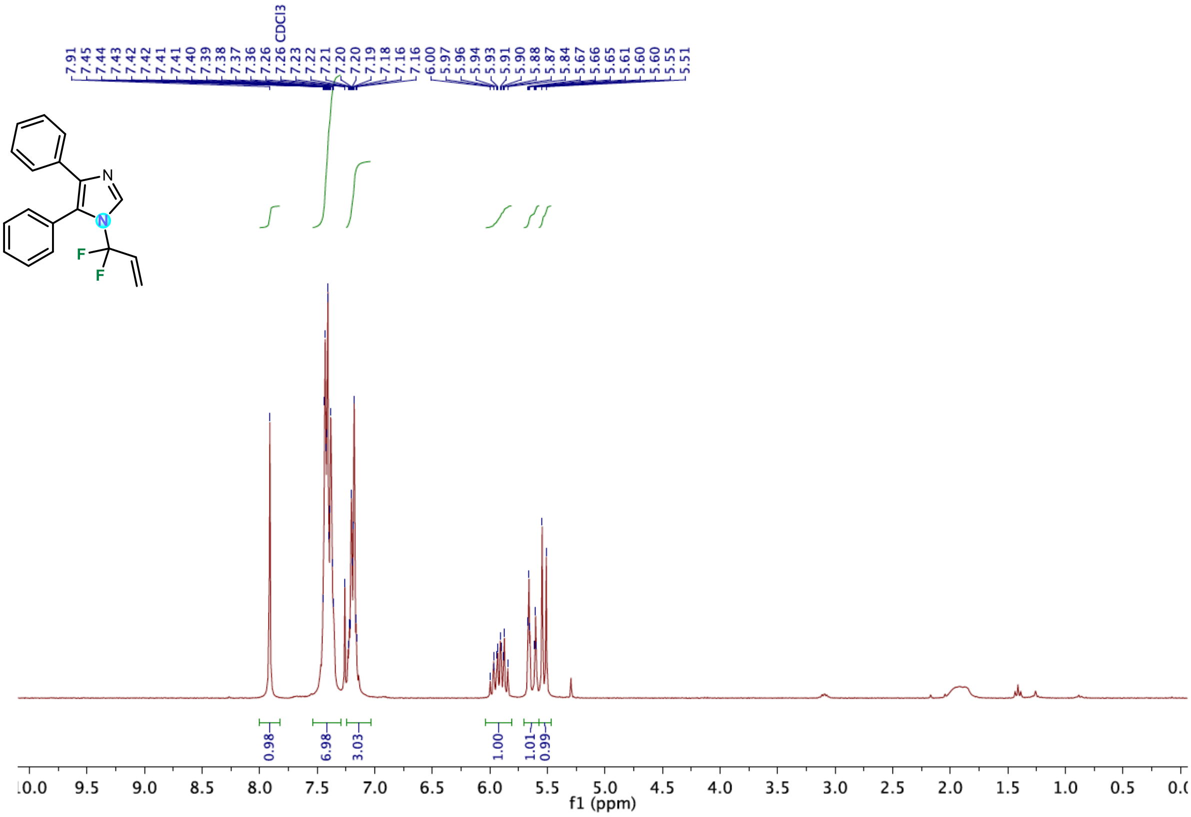

Supplementary Figure 140. ^{1}H NMR spectra of compound 38

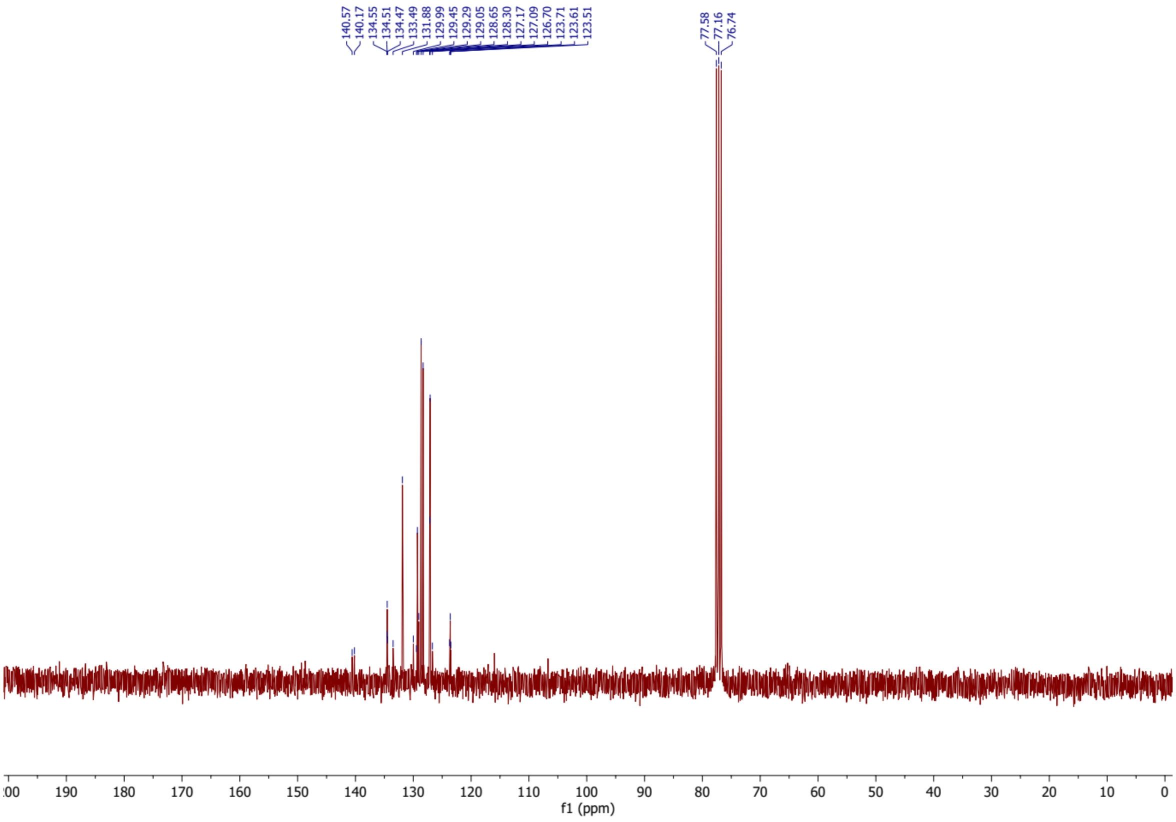

Supplementary Figure 141. ^{13}**C NMR spectra of compound 38**

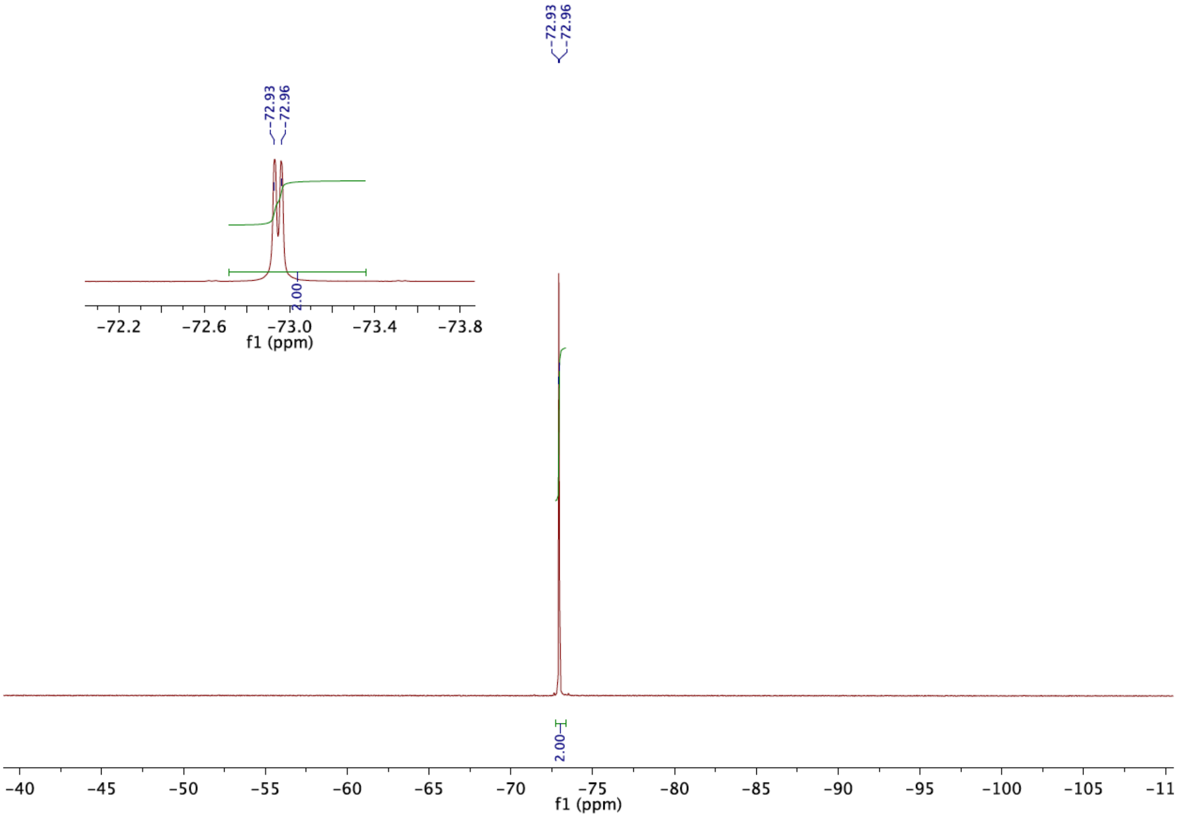

Supplementary Figure 142. ^{19}**F NMR spectra of compound 38**

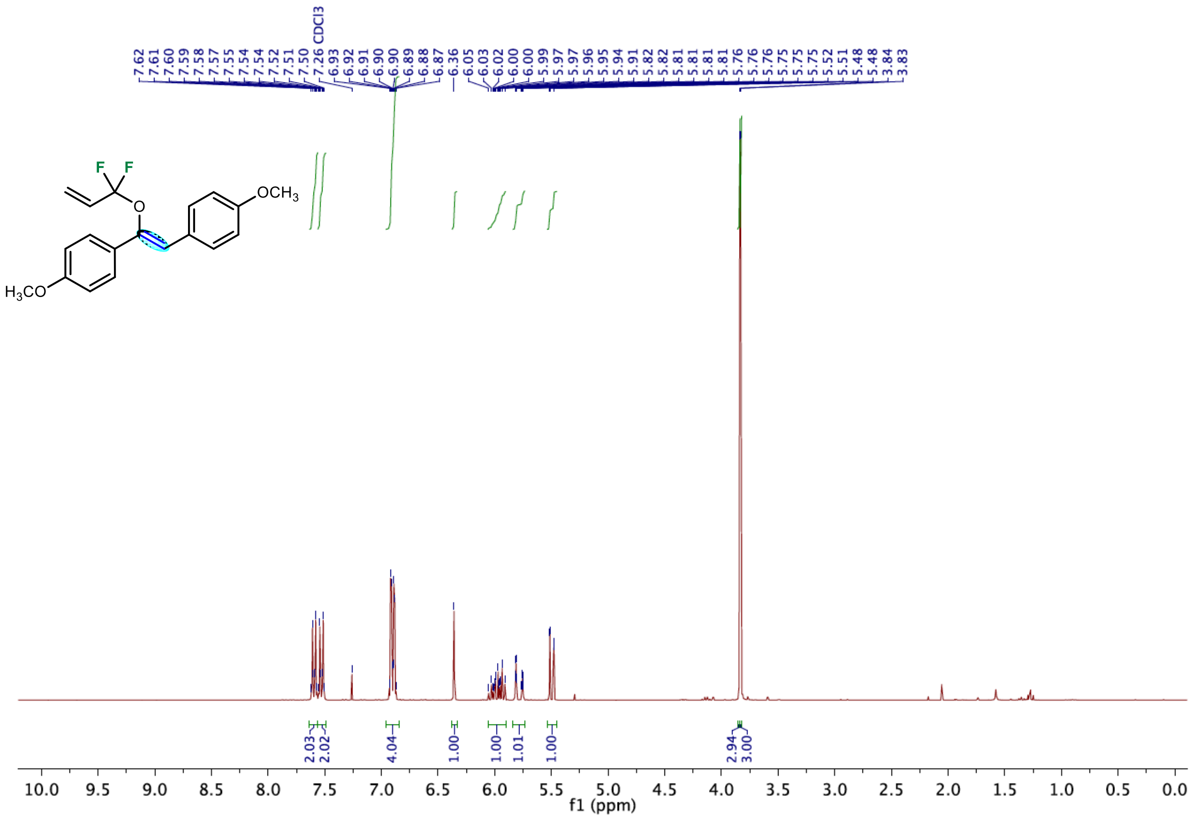

Supplementary Figure 143. ^{1}H NMR spectra of compound 39

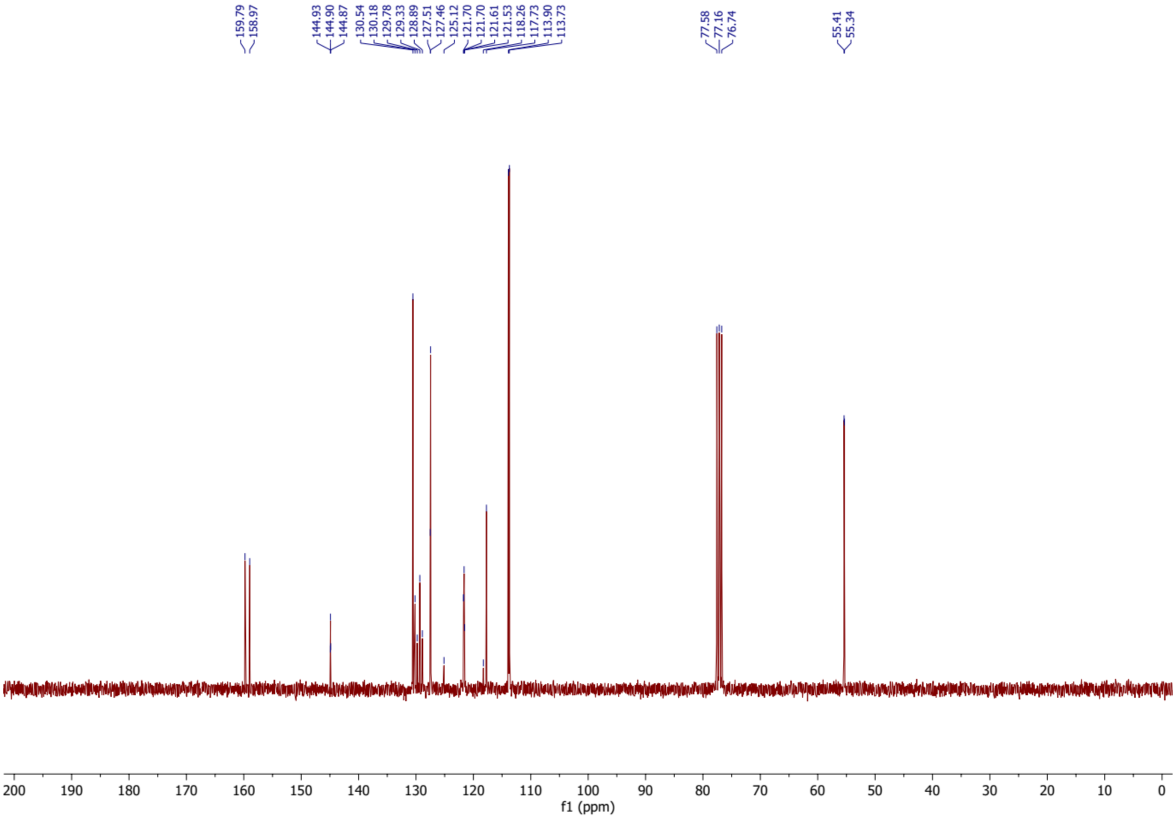

Supplementary Figure 144. ^{13}C NMR spectra of compound 39

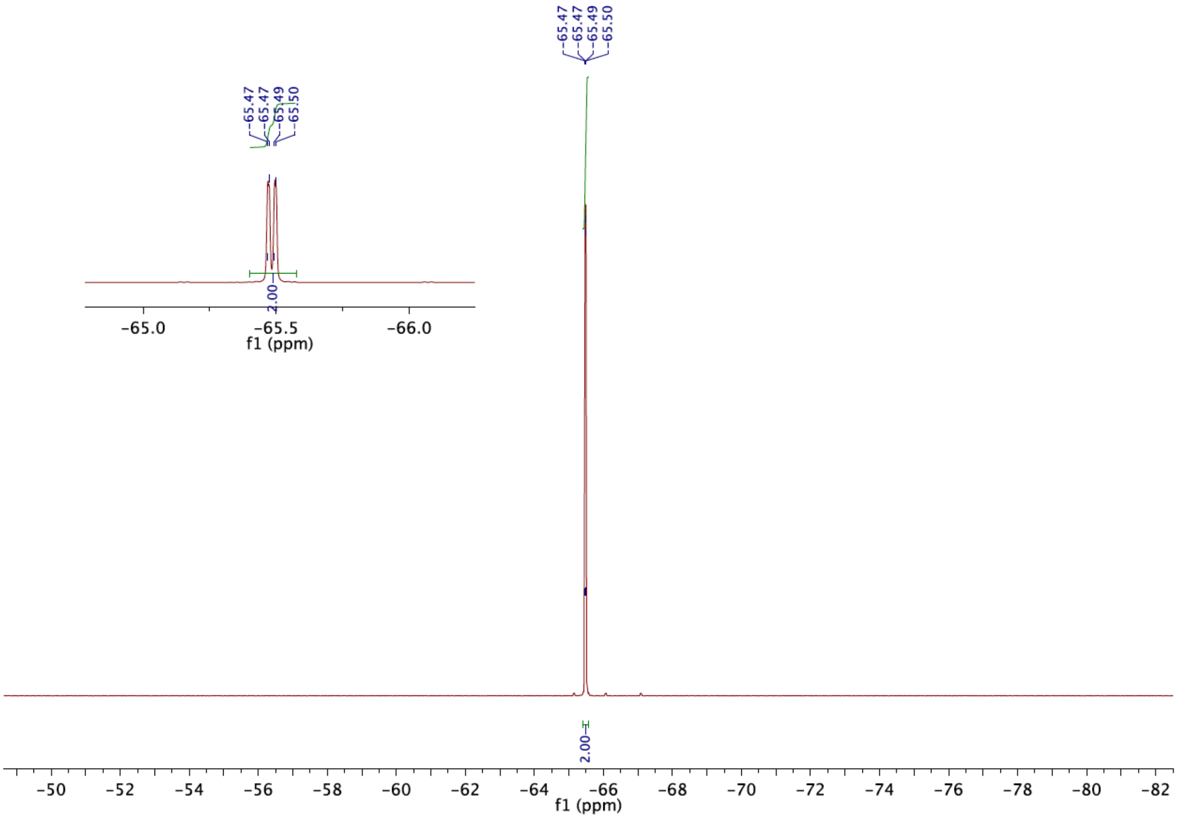

Supplementary Figure 145. ^{19}F NMR spectra of compound 39

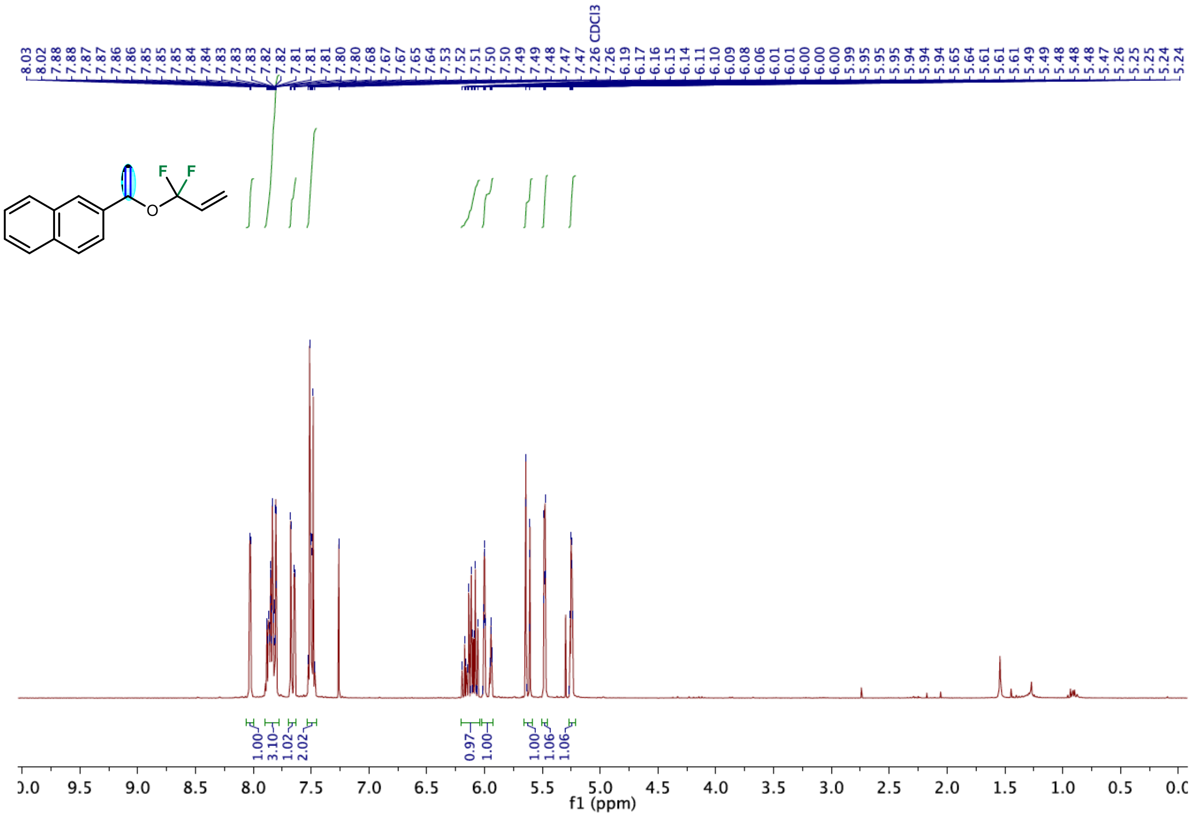

Supplementary Figure 146. ^{1}H NMR spectra of compound 40

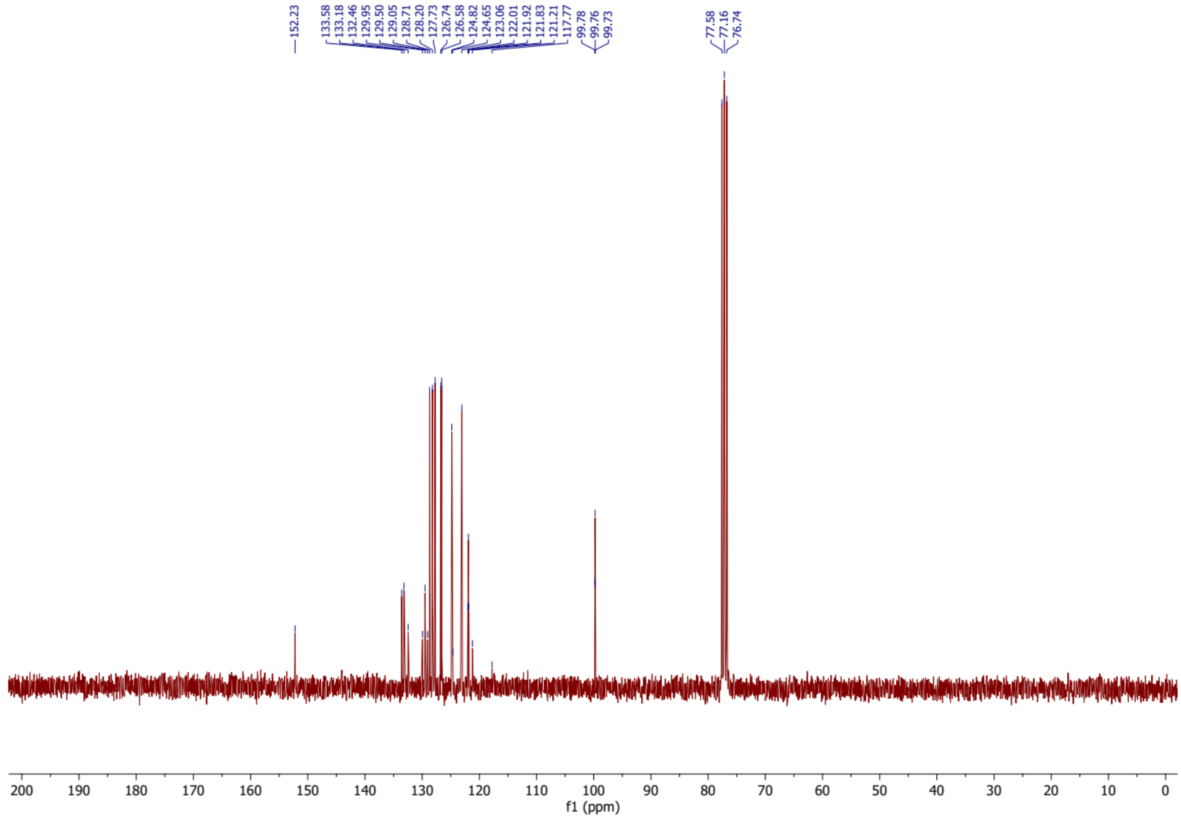

Supplementary Figure 147. ^{13}**C NMR spectra of compound 40**

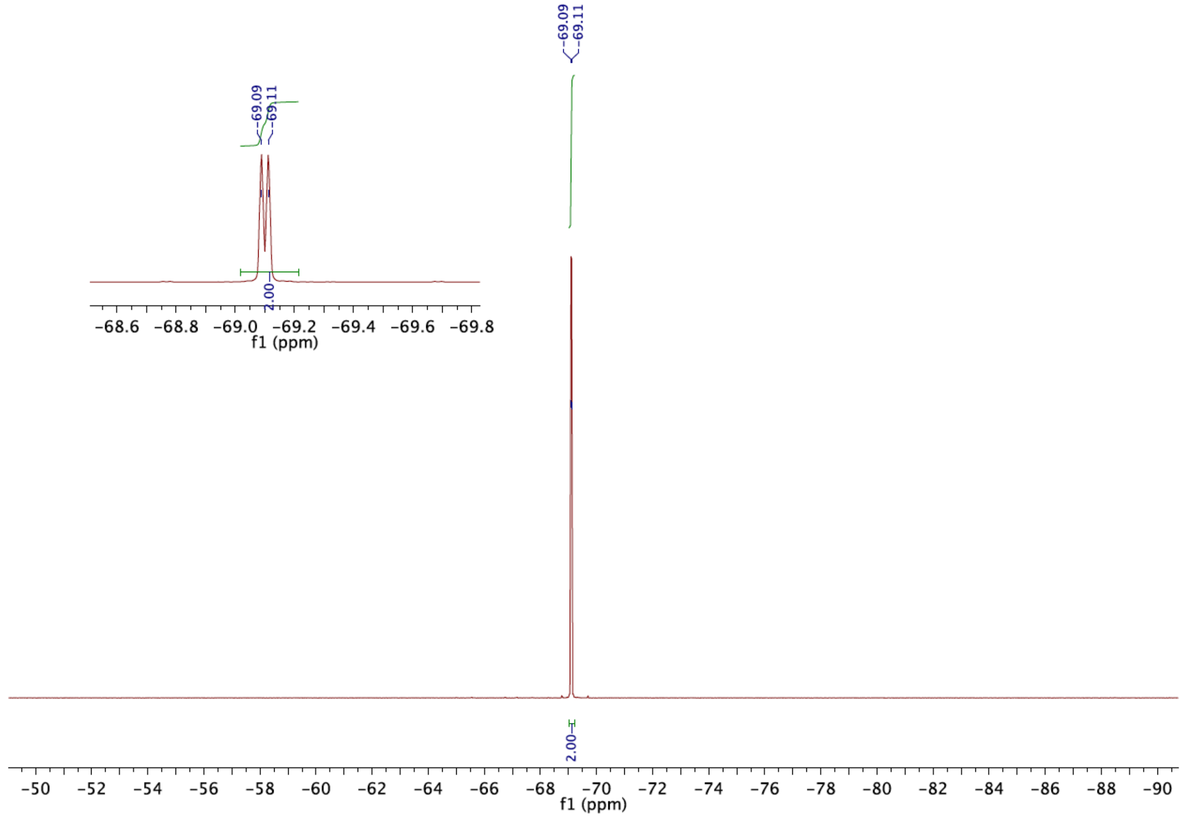

Supplementary Figure 148. ^{19}**F NMR spectra of compound 40**

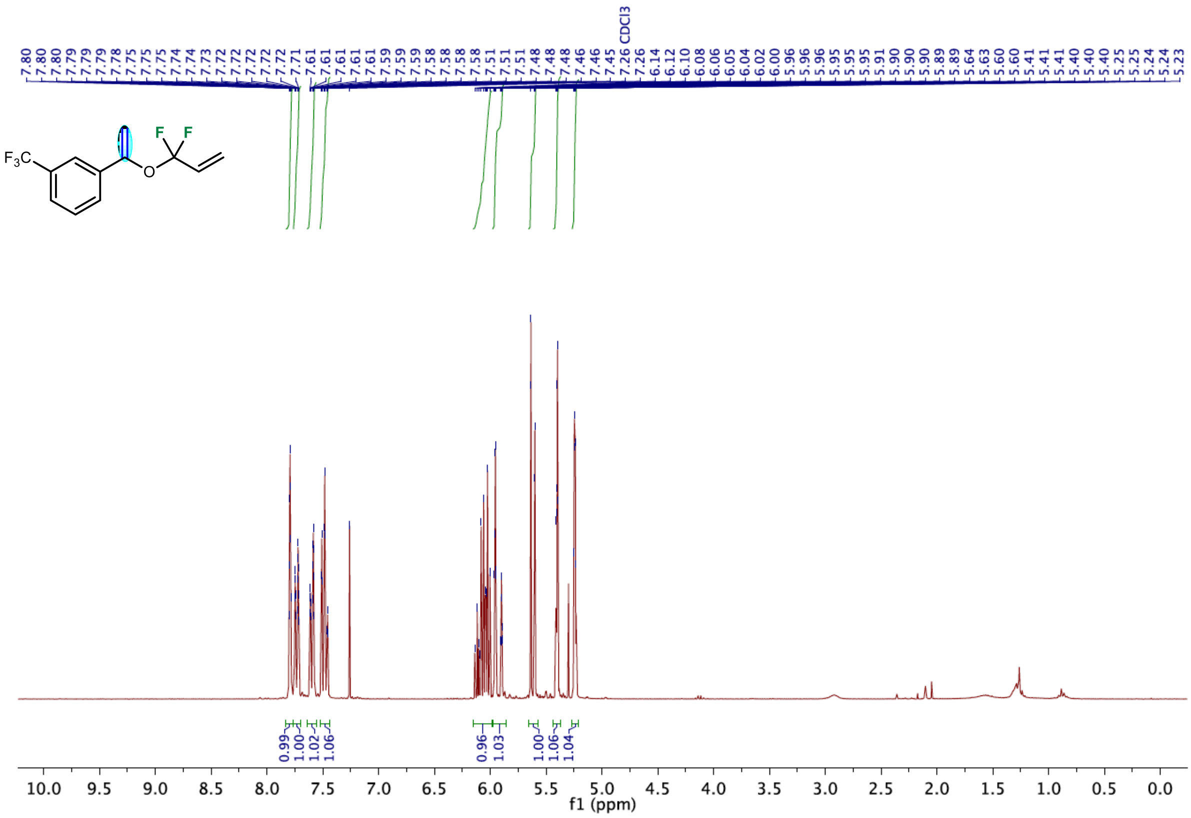

Supplementary Figure 149. ^{1}H NMR spectra of compound 41

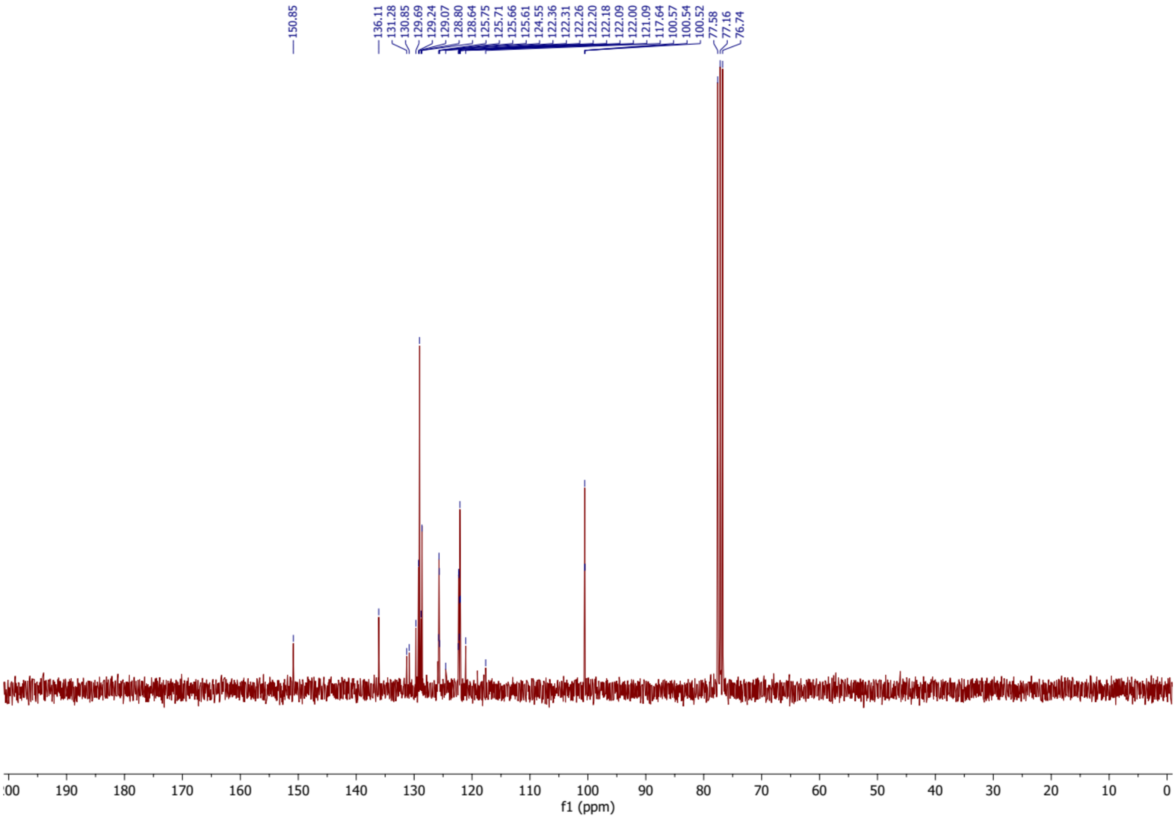

Supplementary Figure 150. ^{13}C NMR spectra of compound 41

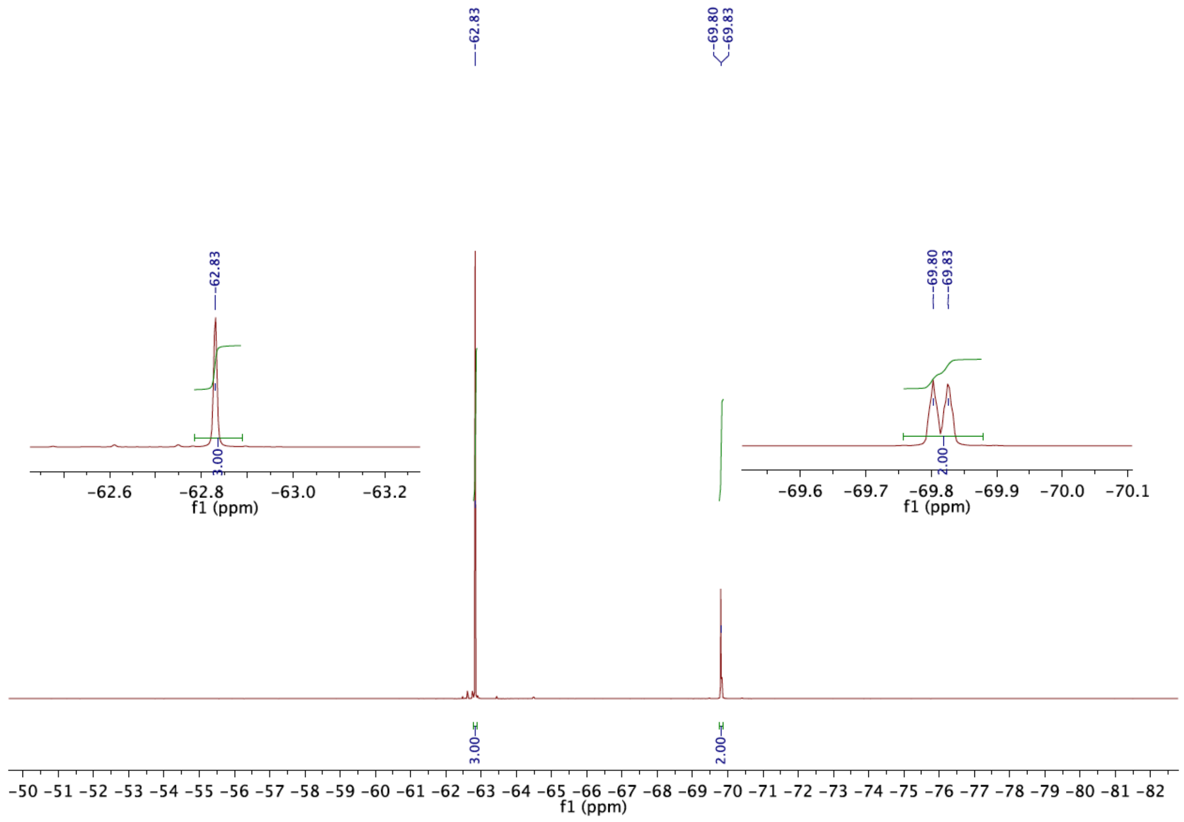

Supplementary Figure 151. ^{19}F NMR spectra of compound 41

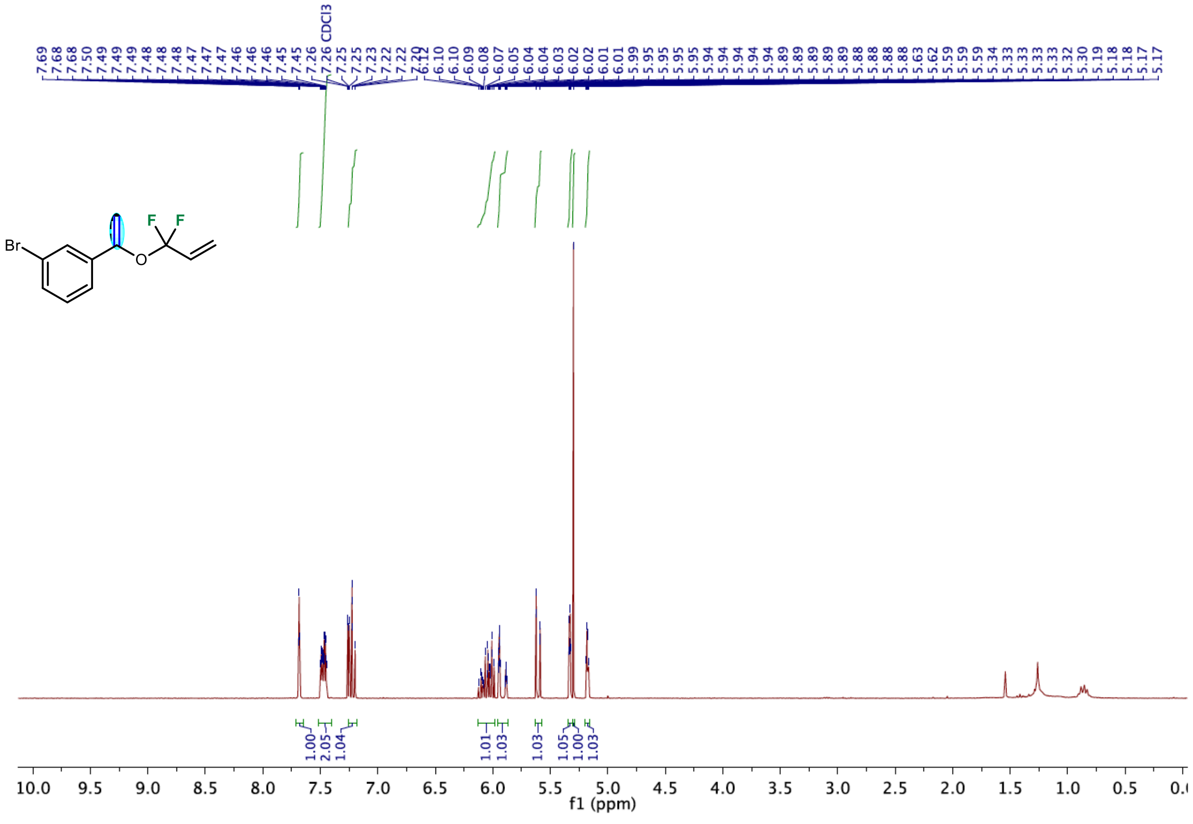

Supplementary Figure 152. ^{1}H NMR spectra of compound 42

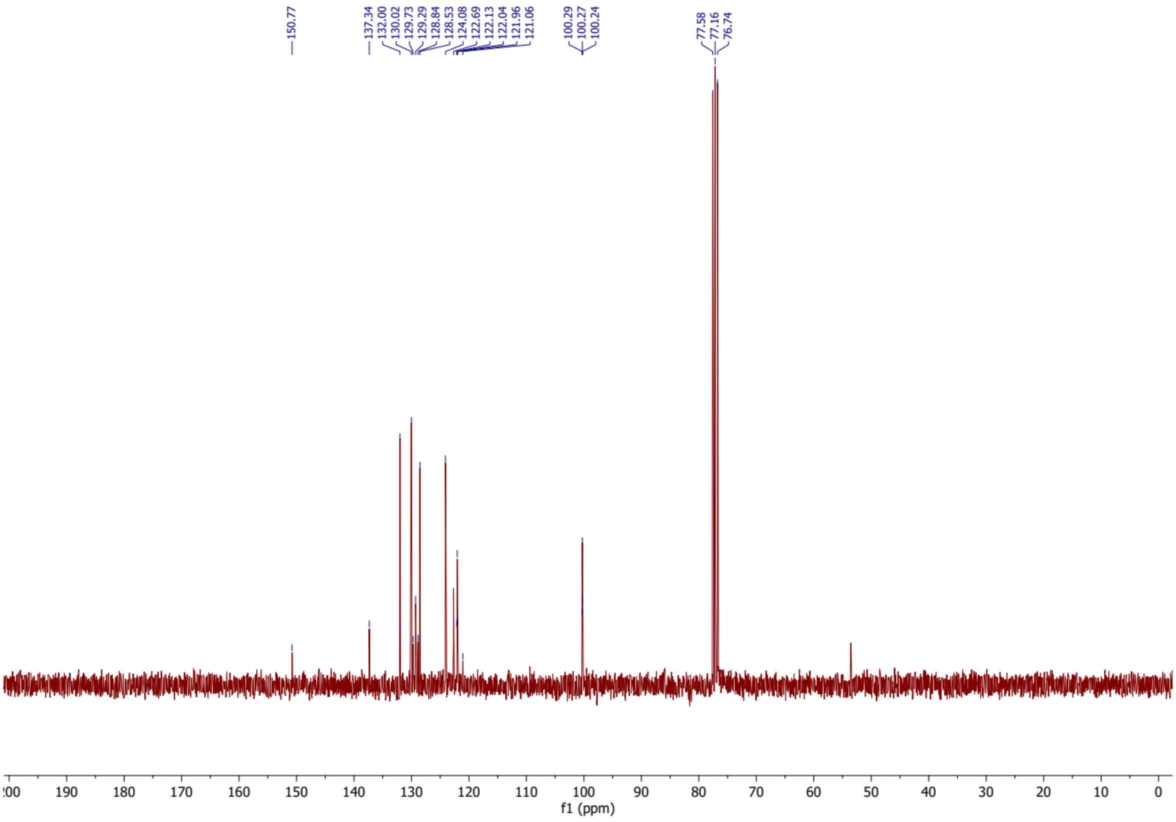

Supplementary Figure 153. ^{13}C NMR spectra of compound 42

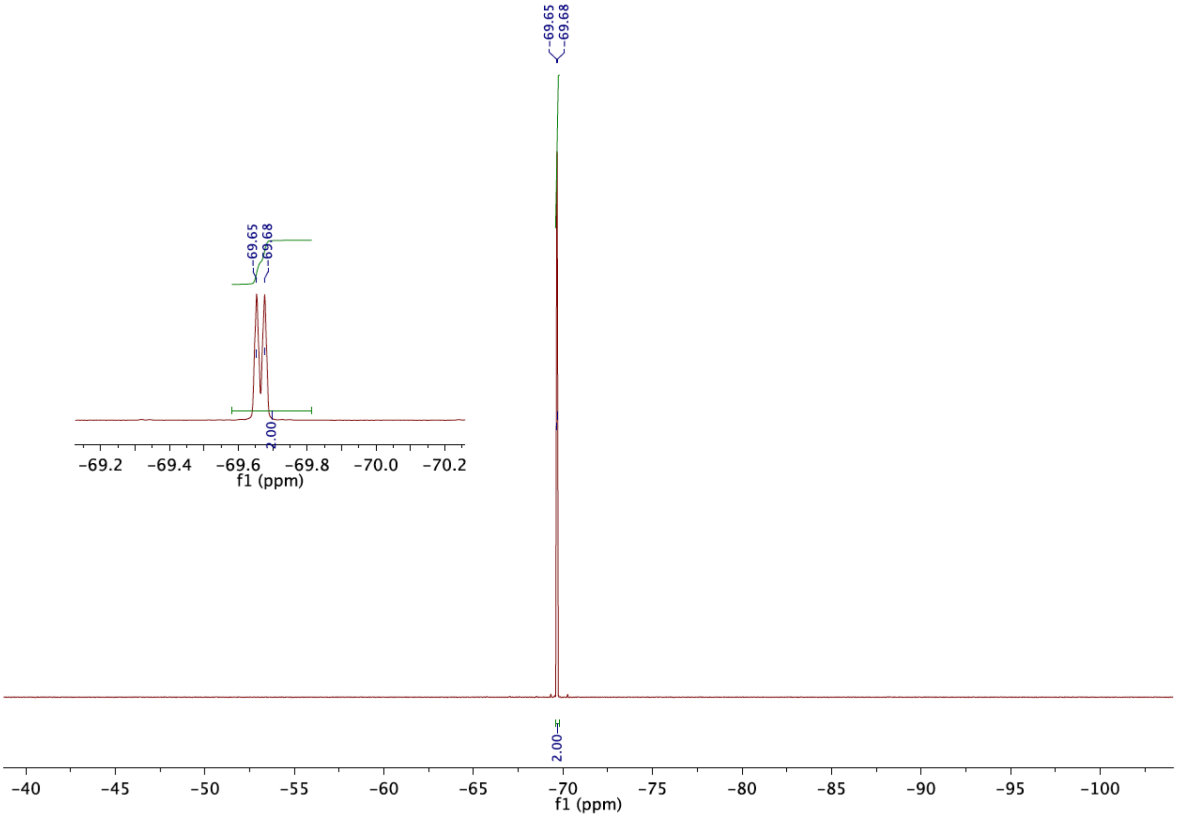

Supplementary Figure 154. ^{19}F NMR spectra of compound 42

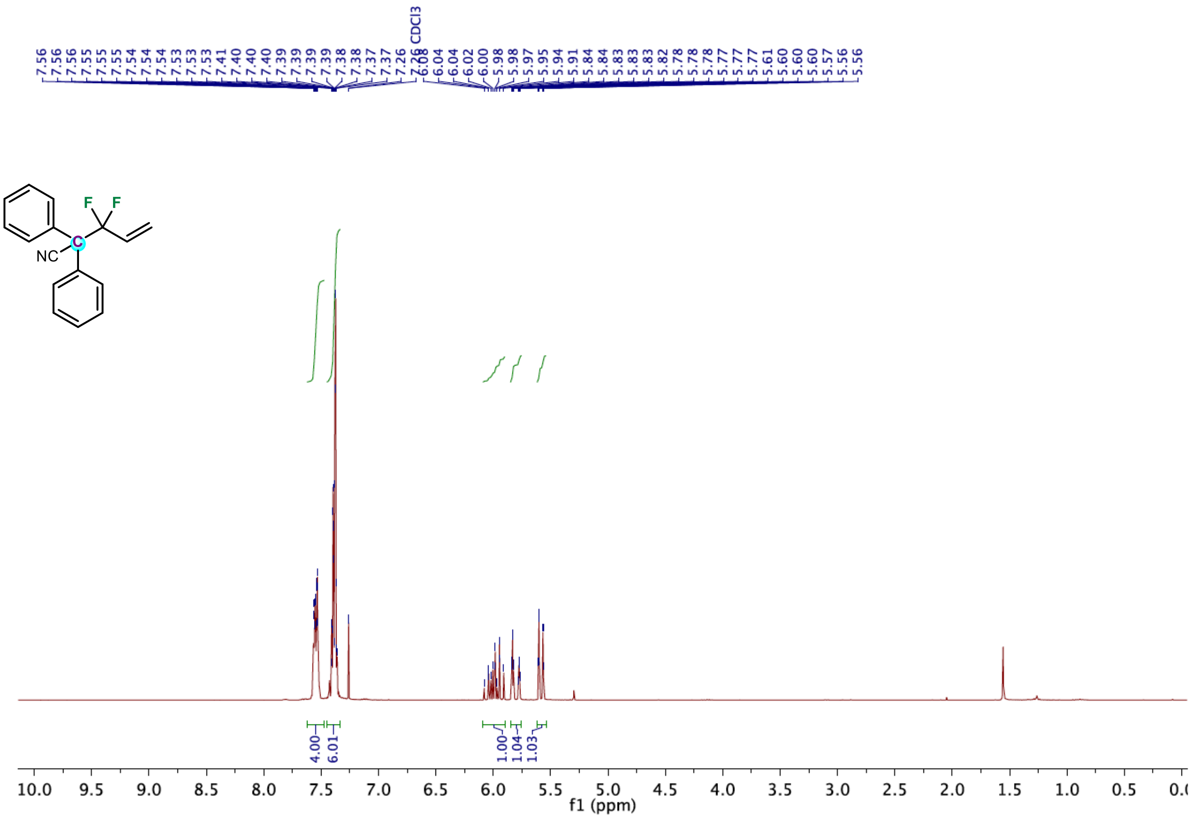

Supplementary Figure 155. ^{1}H NMR spectra of compound 43

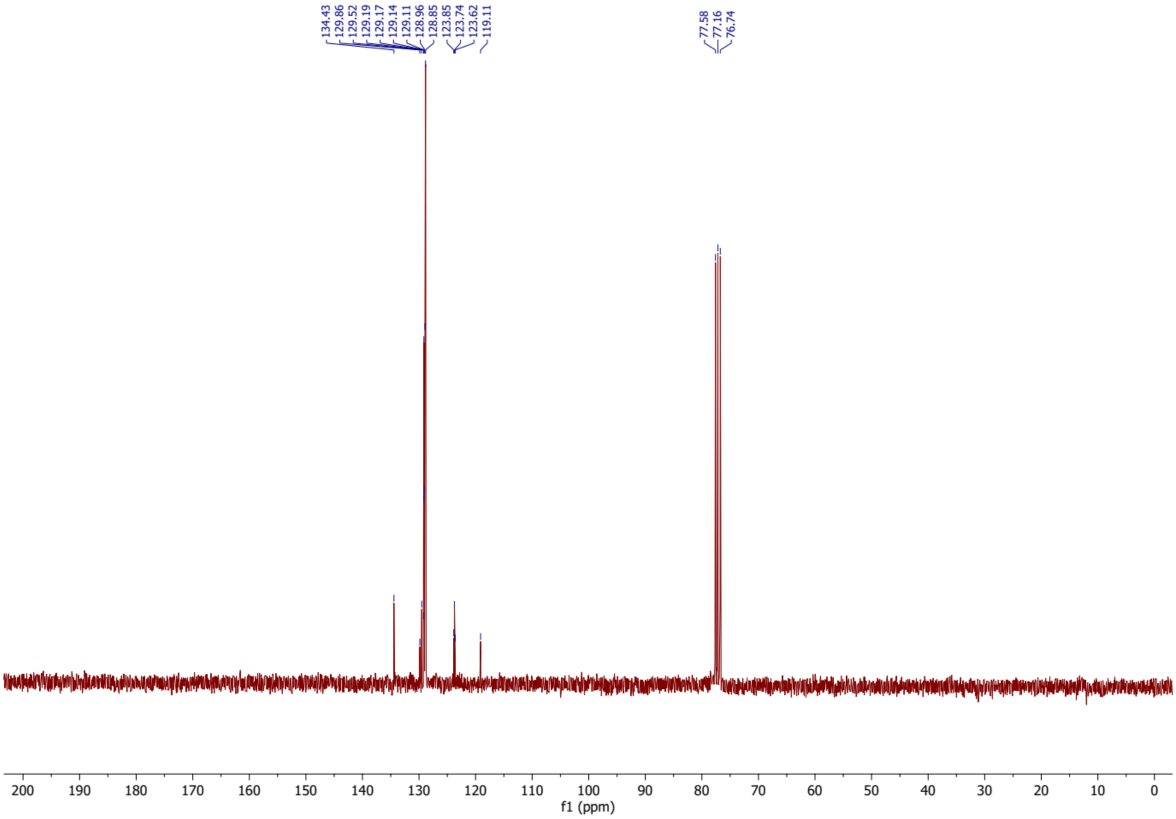

Supplementary Figure 156. ^{13}C NMR spectra of compound 43

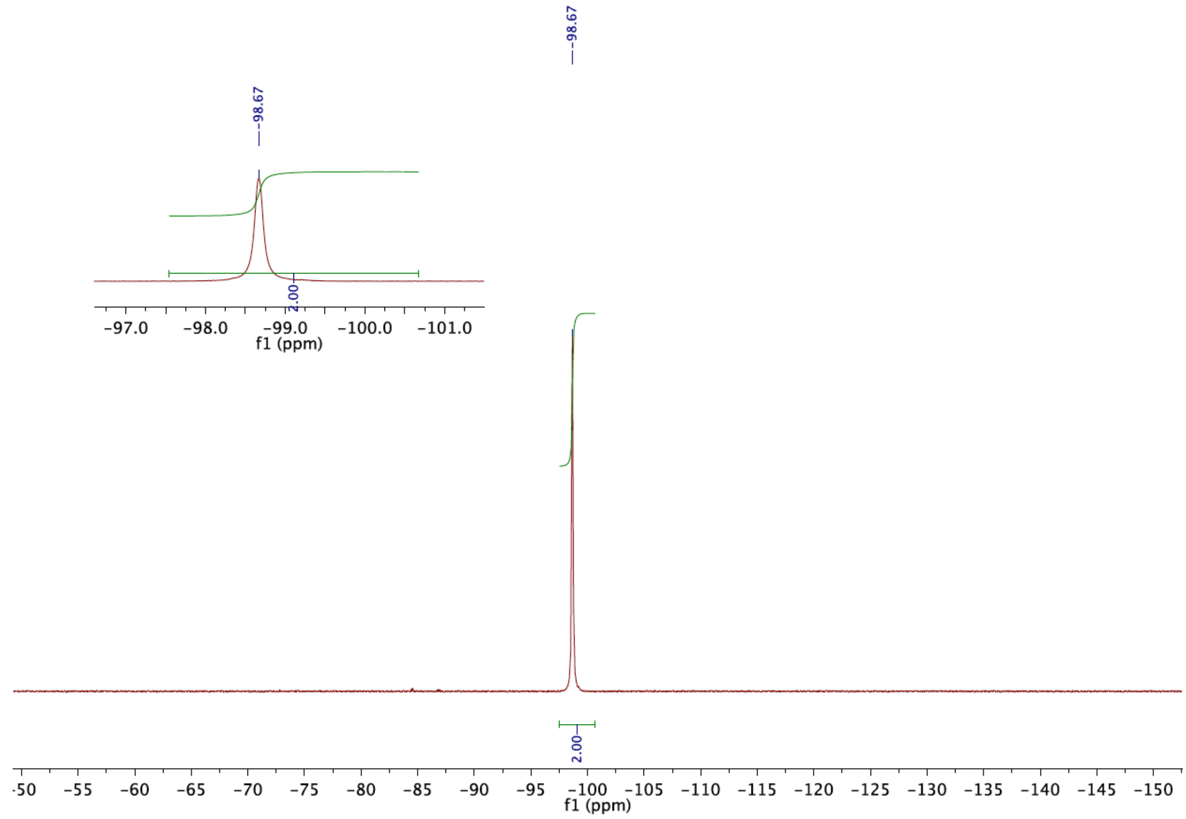

Supplementary Figure 157. ^{19}F NMR spectra of compound 43

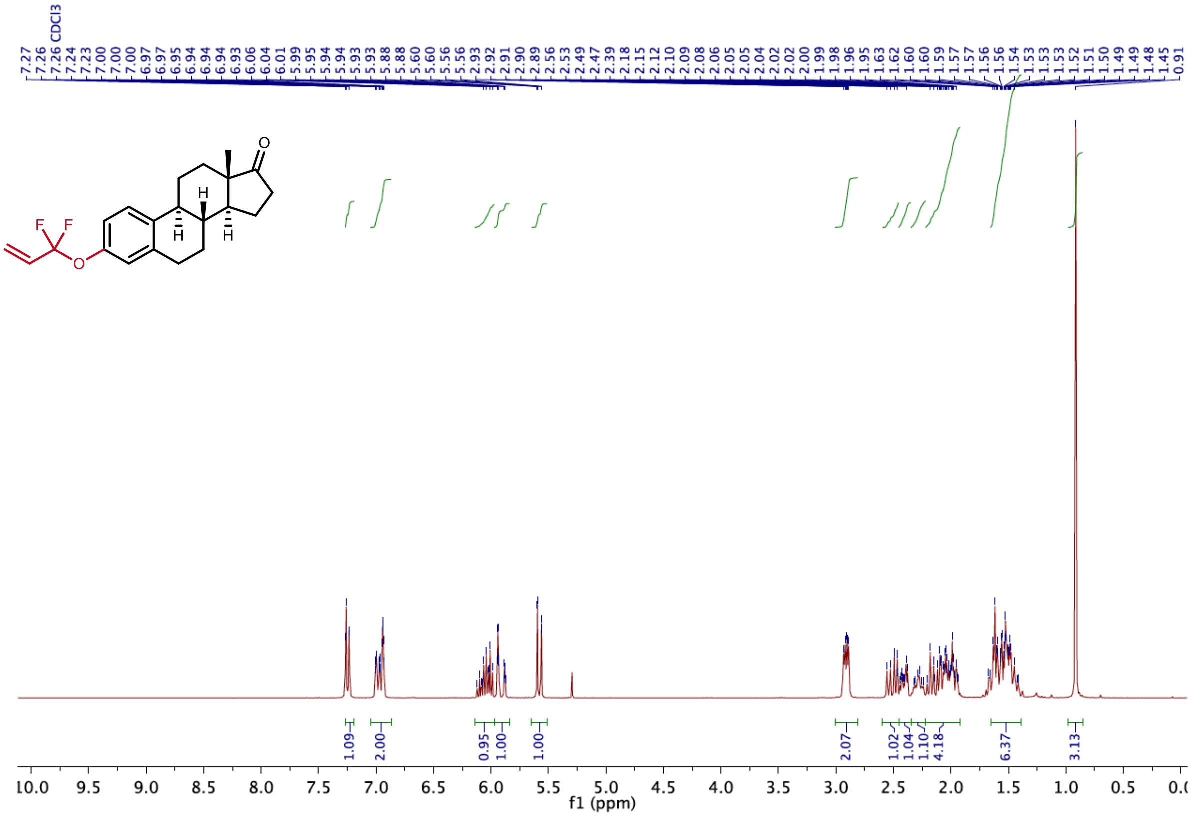

Supplementary Figure 158. ^{1}H NMR spectra of compound 44

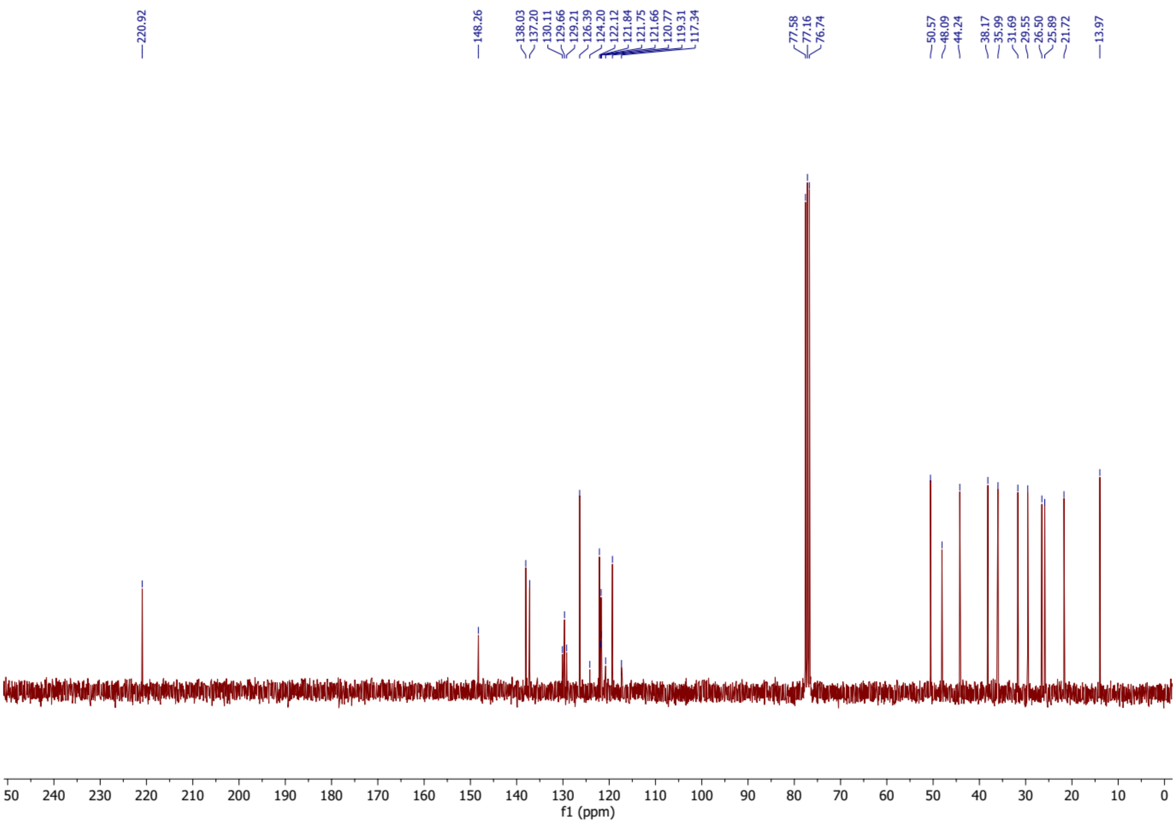

Supplementary Figure 159. ^{13}C NMR spectra of compound 44

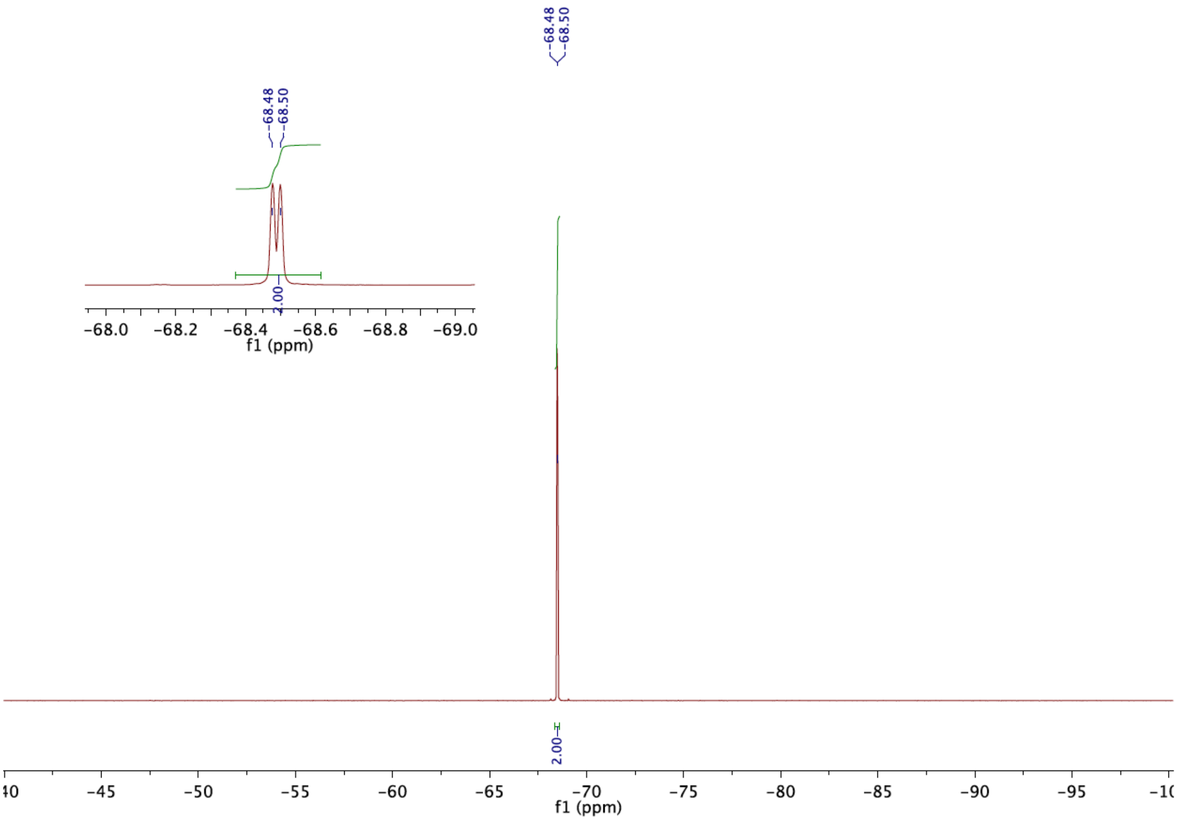

Supplementary Figure 160. ^{19}F NMR spectra of compound 44

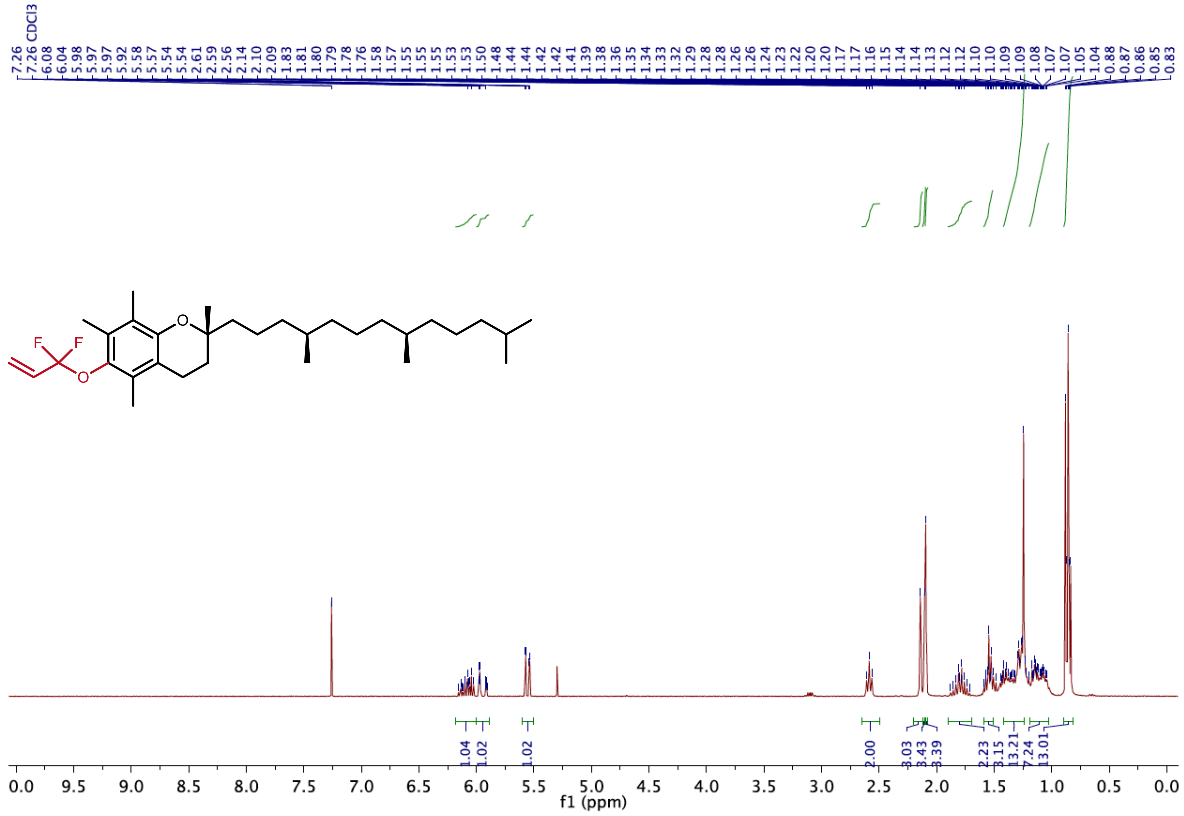

Supplementary Figure 161. ^{1}**H NMR spectra of compound 45**

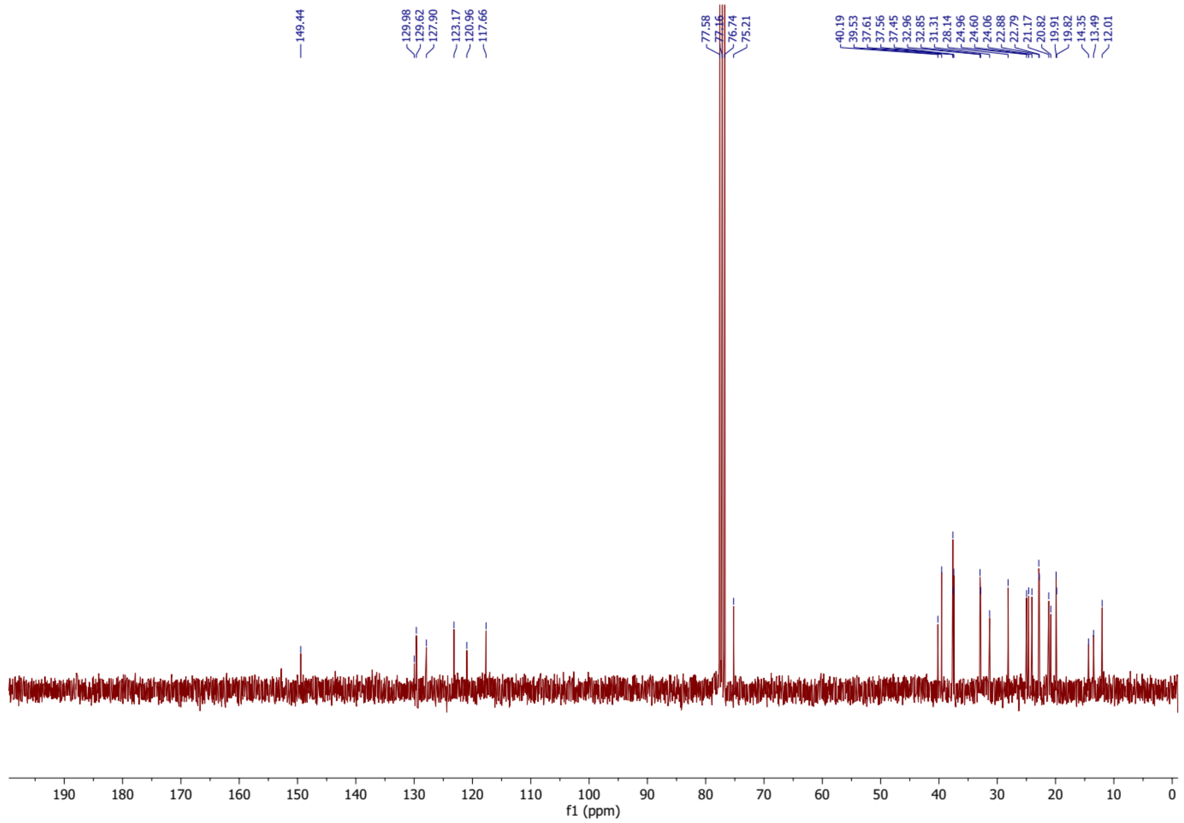

Supplementary Figure 162. ^{13}**C NMR spectra of compound 45**

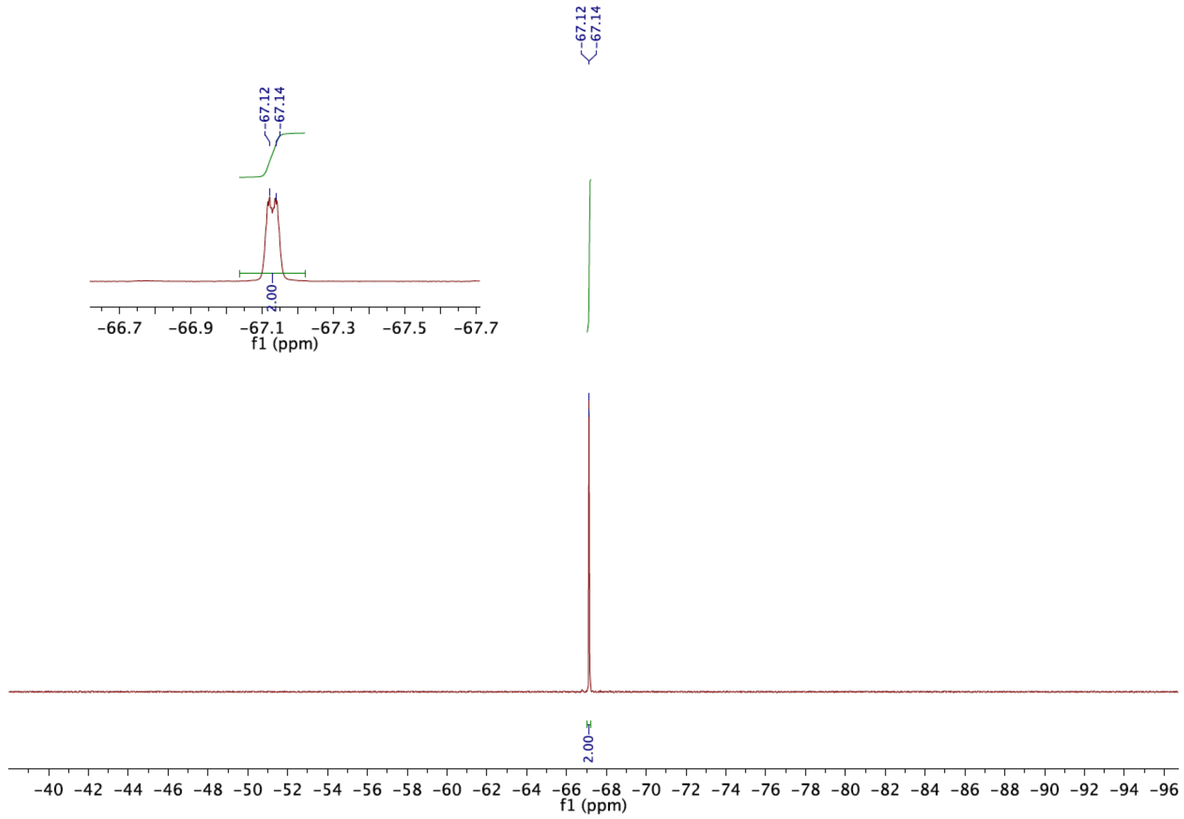

Supplementary Figure 163. ^{19}F NMR spectra of compound 45

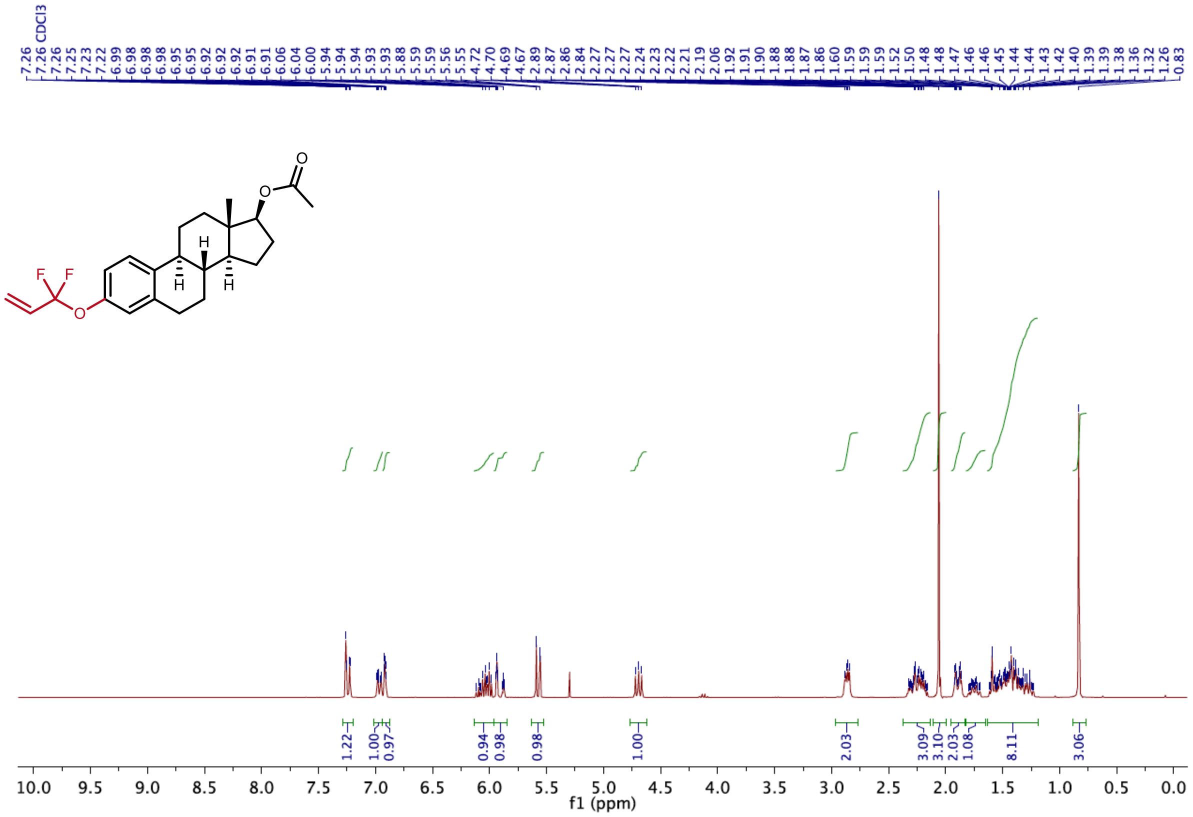

Supplementary Figure 164. ^{1}H NMR spectra of compound 46

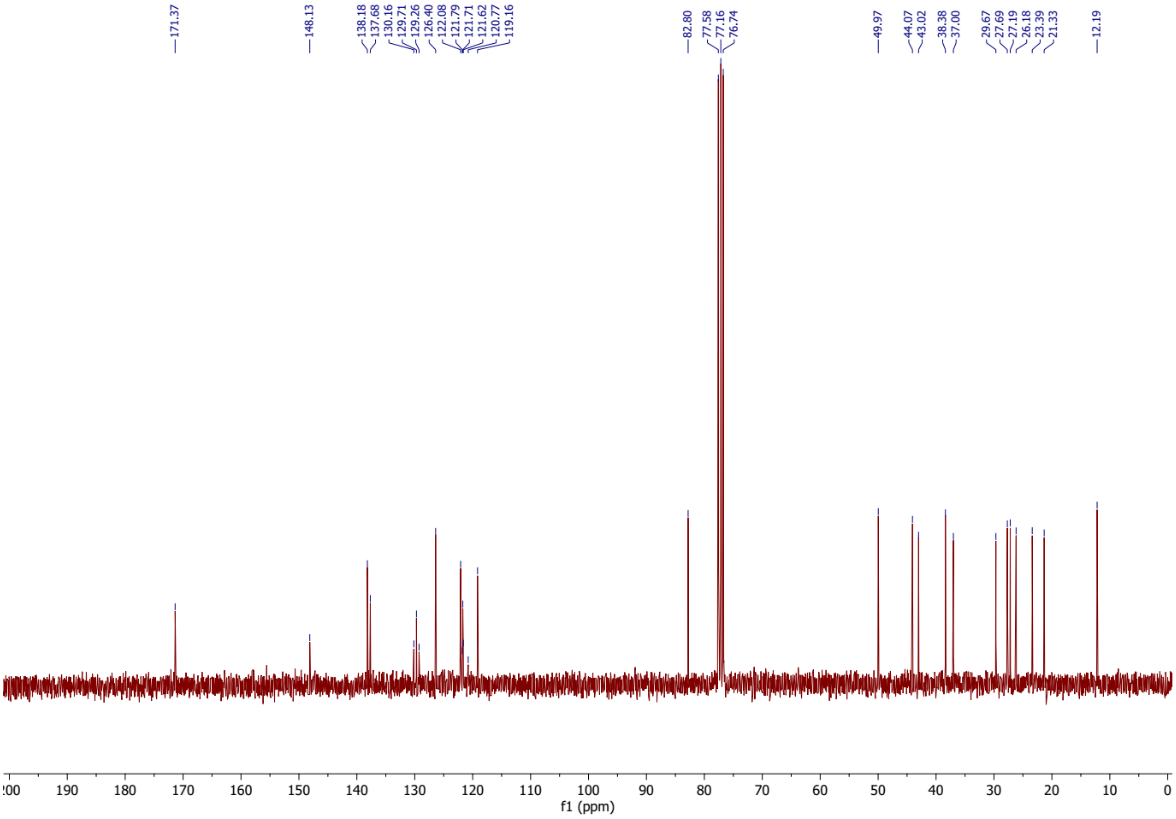

Supplementary Figure 165. ^{13}C NMR spectra of compound 46

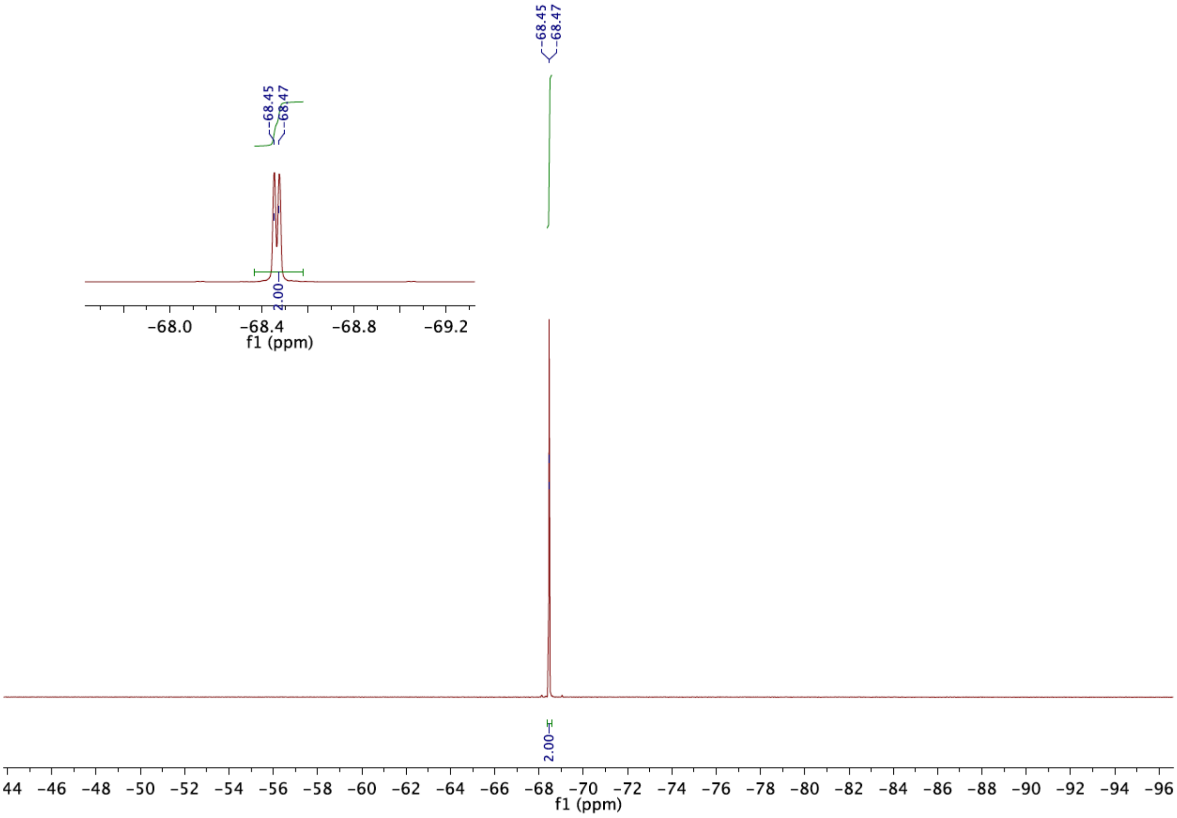

Supplementary Figure 166. ^{19}F NMR spectra of compound 46

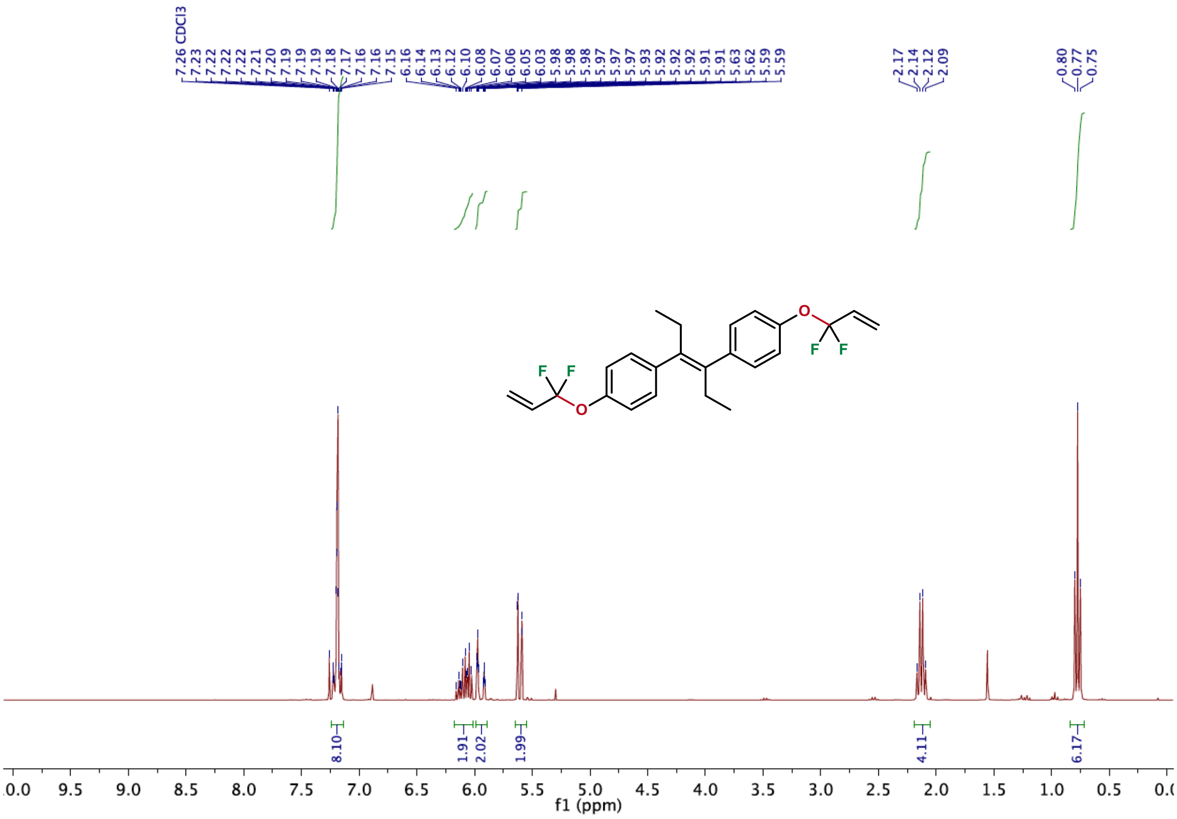

Supplementary Figure 167. ^{1}H NMR spectra of compound 47

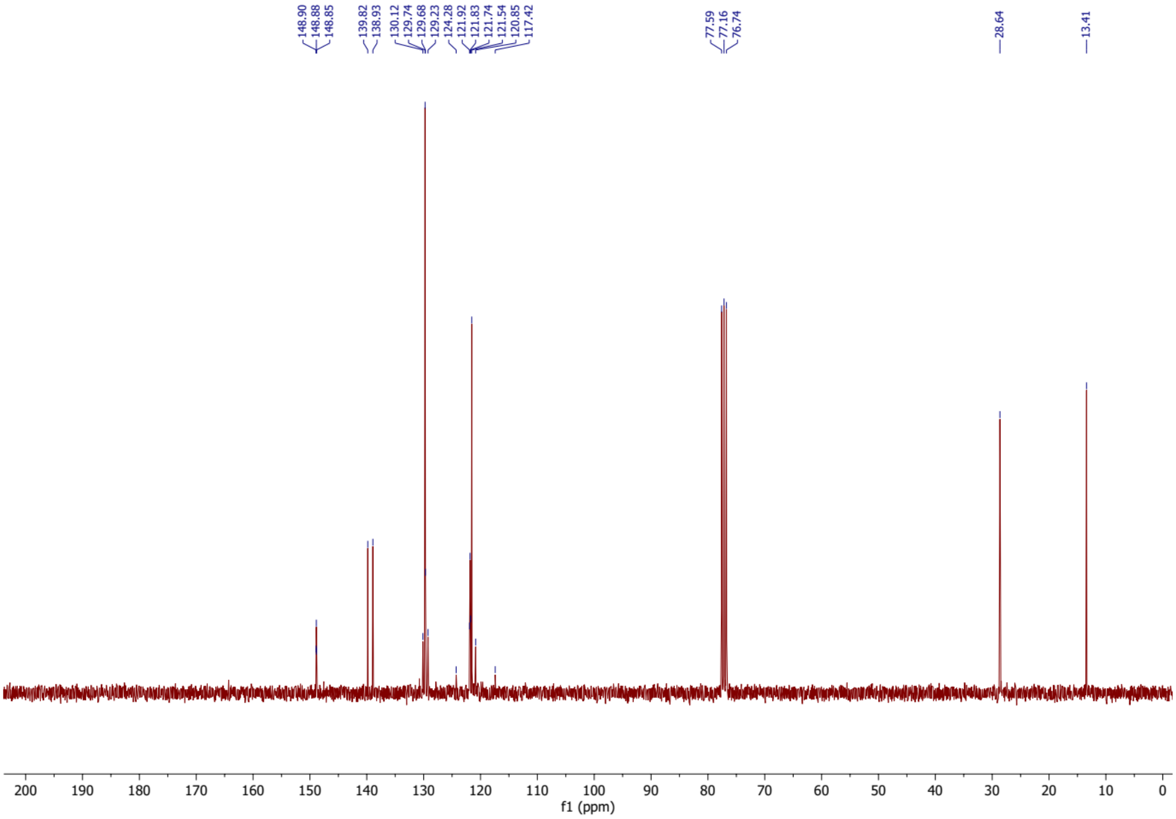

Supplementary Figure 168. ^{13}C NMR spectra of compound 47

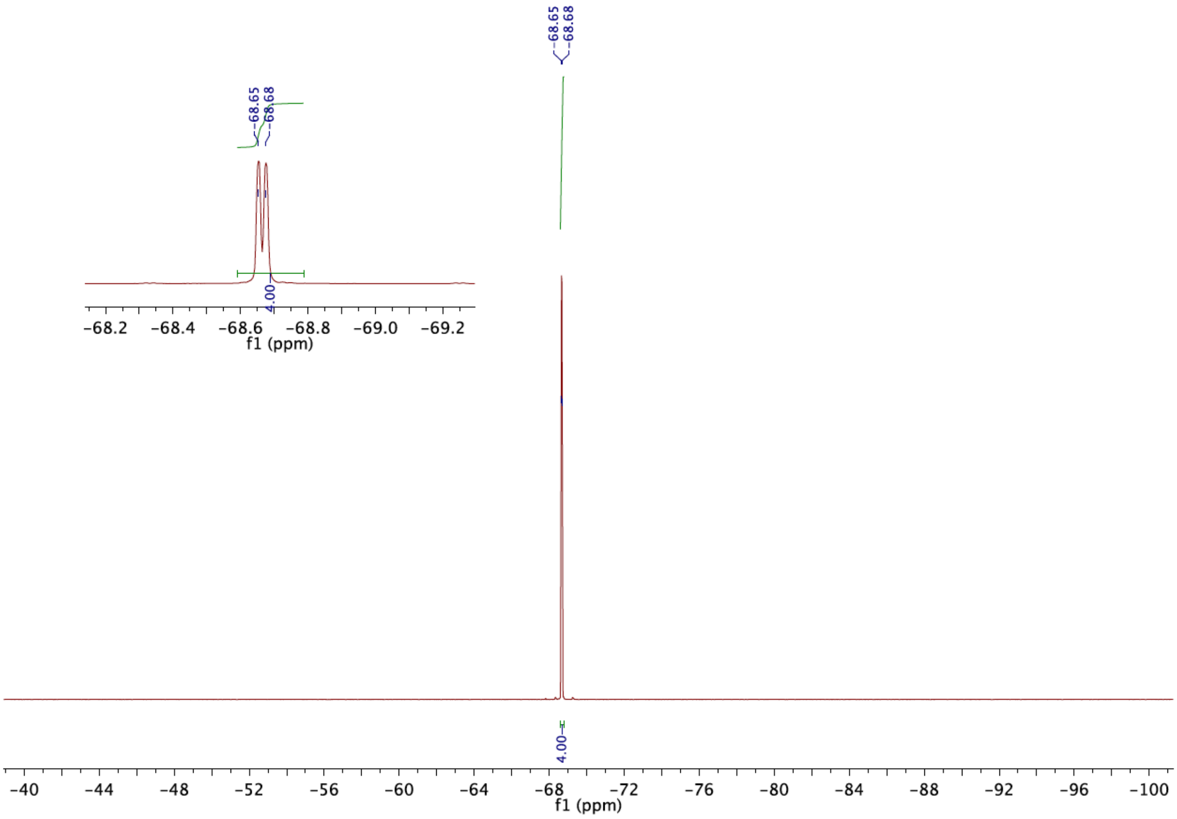

Supplementary Figure 169. ^{19}F NMR spectra of compound 47

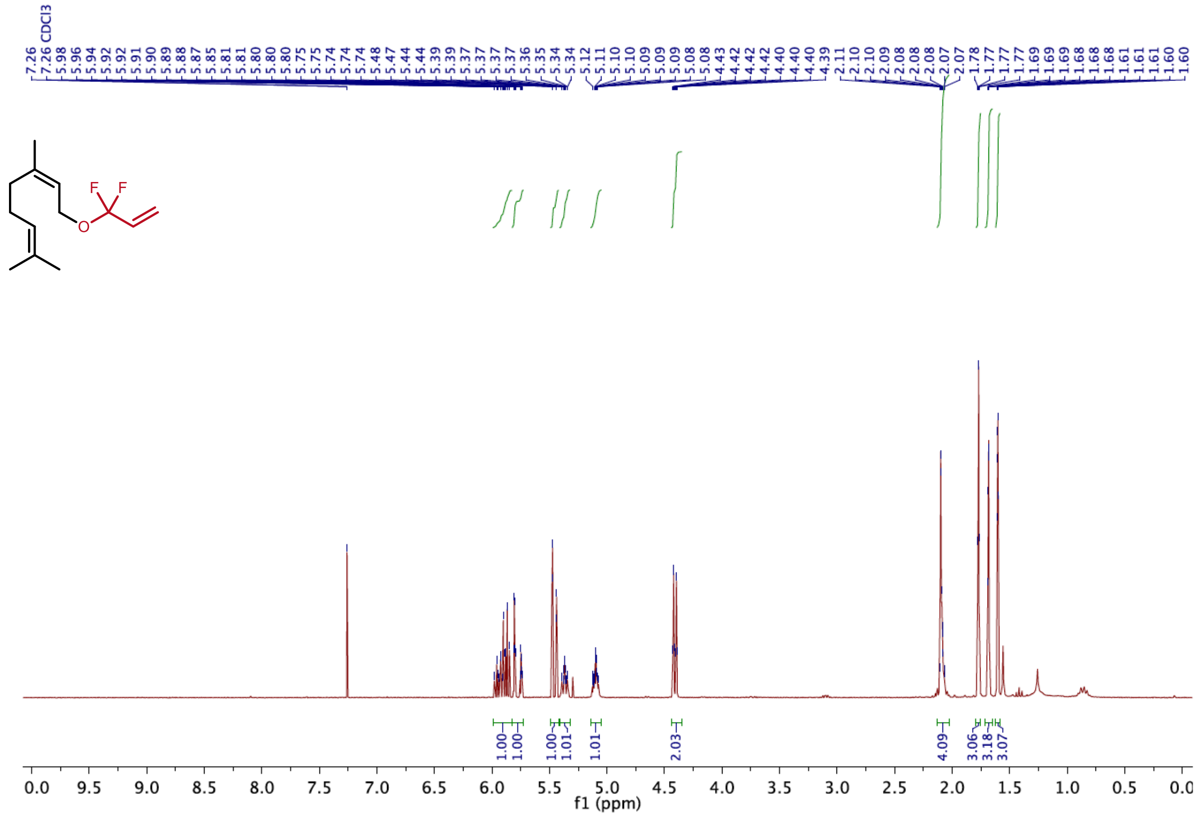

Supplementary Figure 170. ^{1}H NMR spectra of compound 48

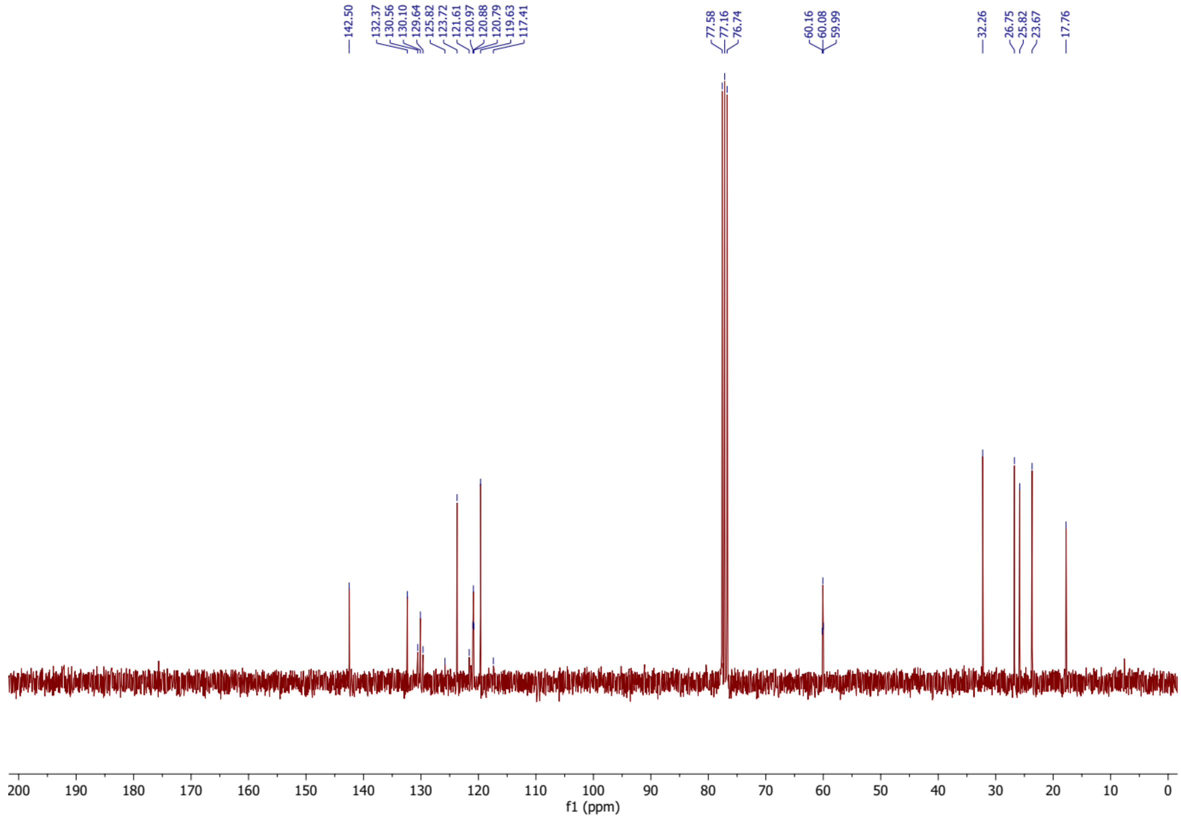

Supplementary Figure 171. ^{13}C NMR spectra of compound 48

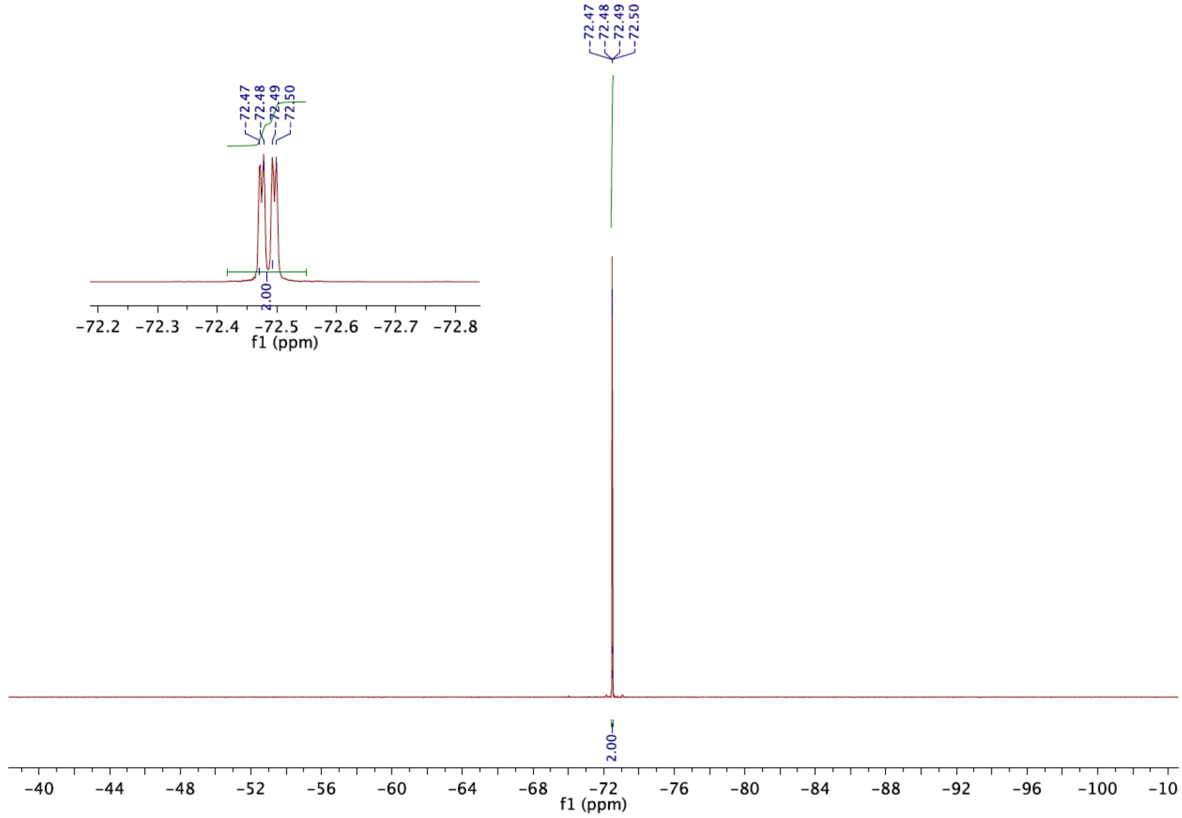

Supplementary Figure 172. ^{19}F NMR spectra of compound 48

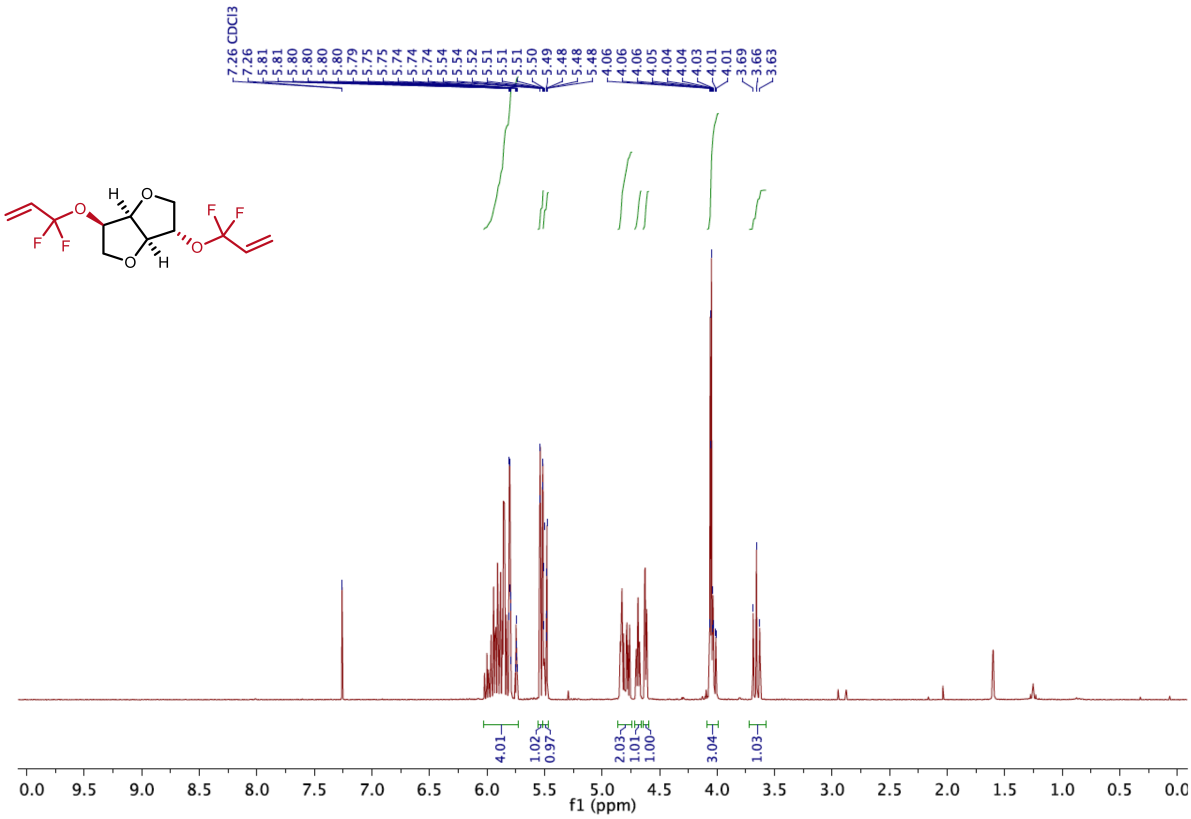

Supplementary Figure 173. ^{1}H NMR spectra of compound 49

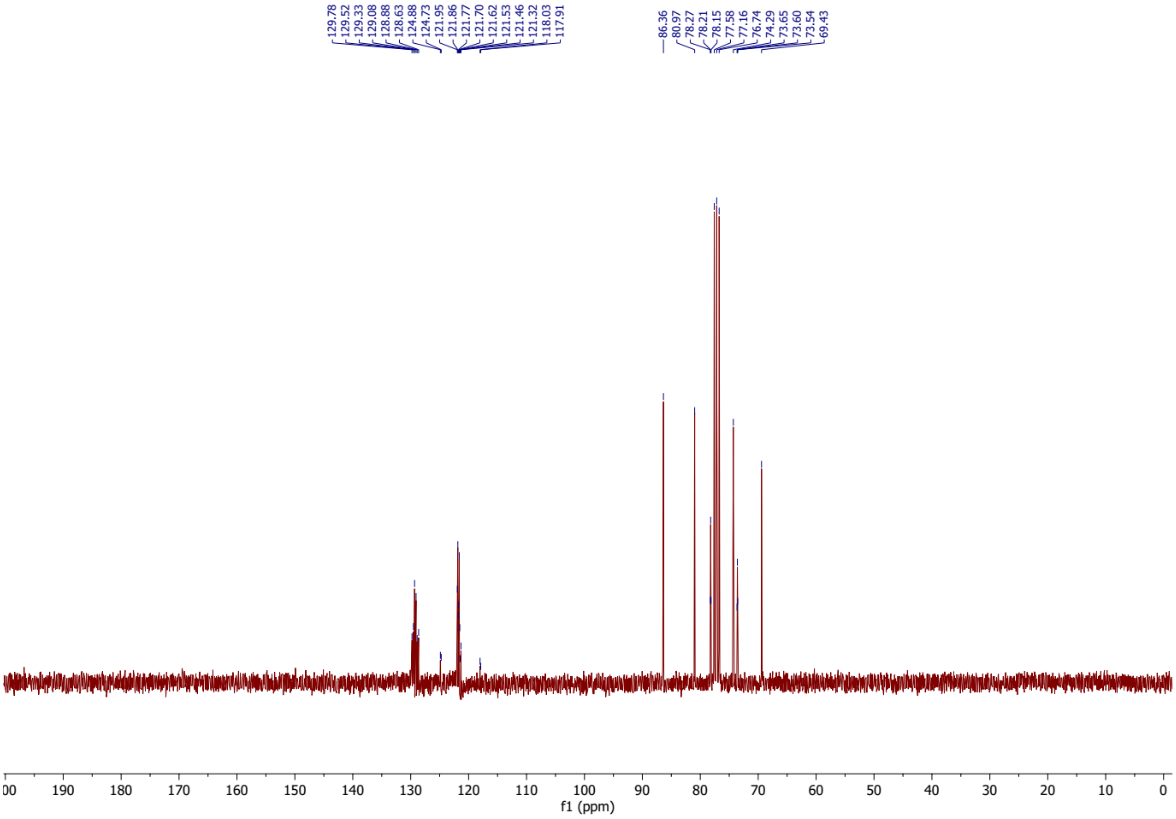

Supplementary Figure 174. ^{13}C NMR spectra of compound 49

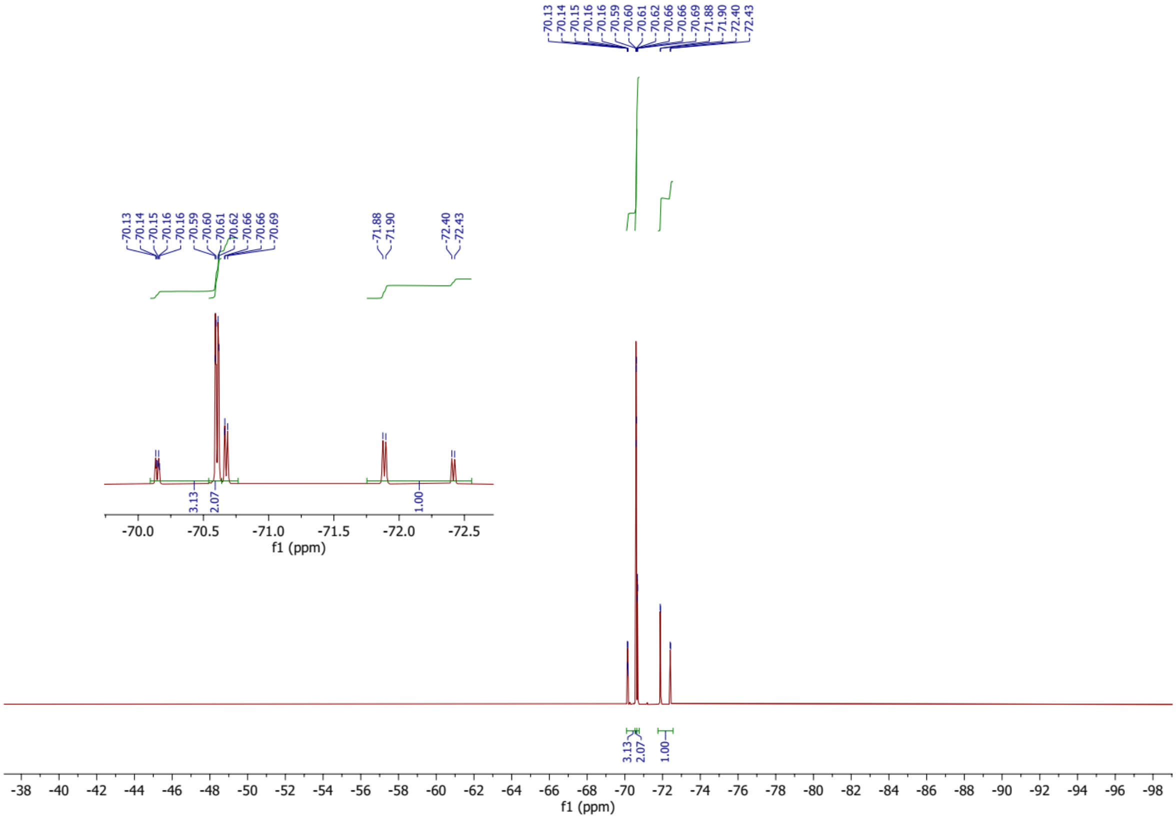

Supplementary Figure 175. ^{19}F NMR spectra of compound 49

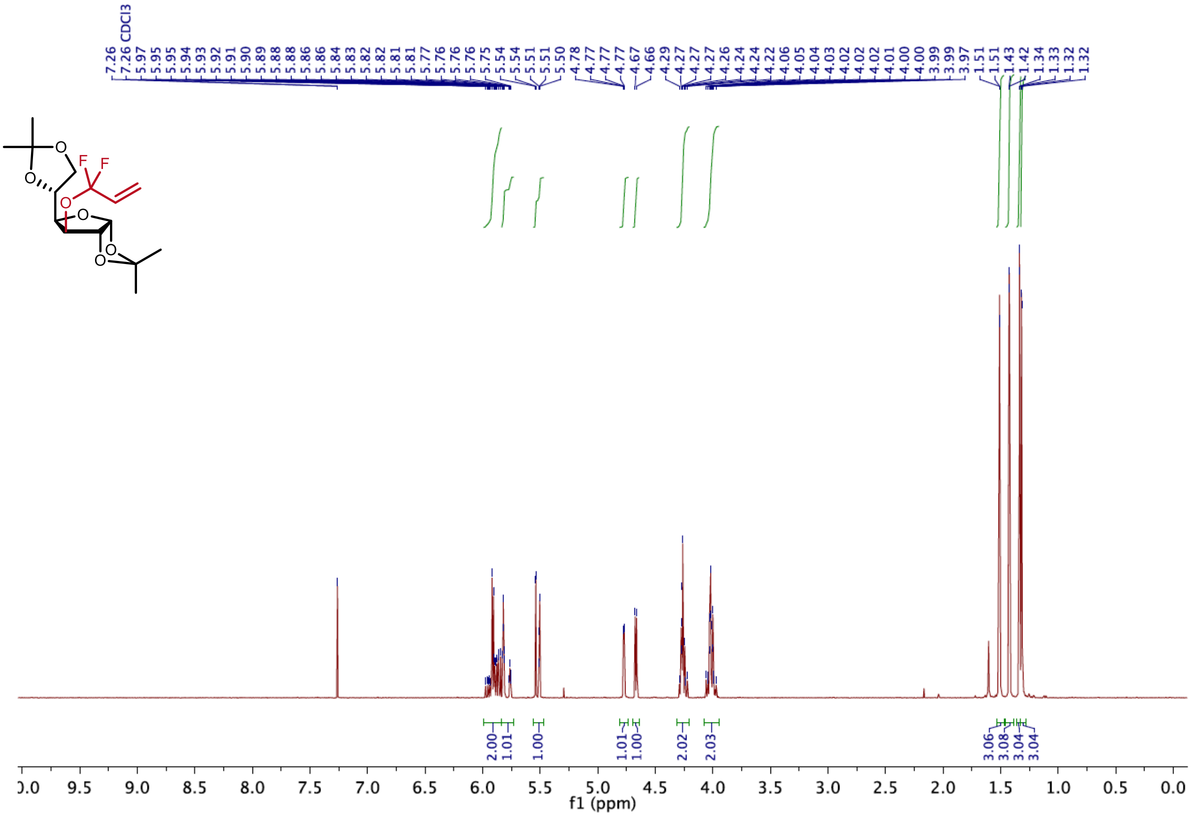

Supplementary Figure 176. ^{1}H NMR spectra of compound 50

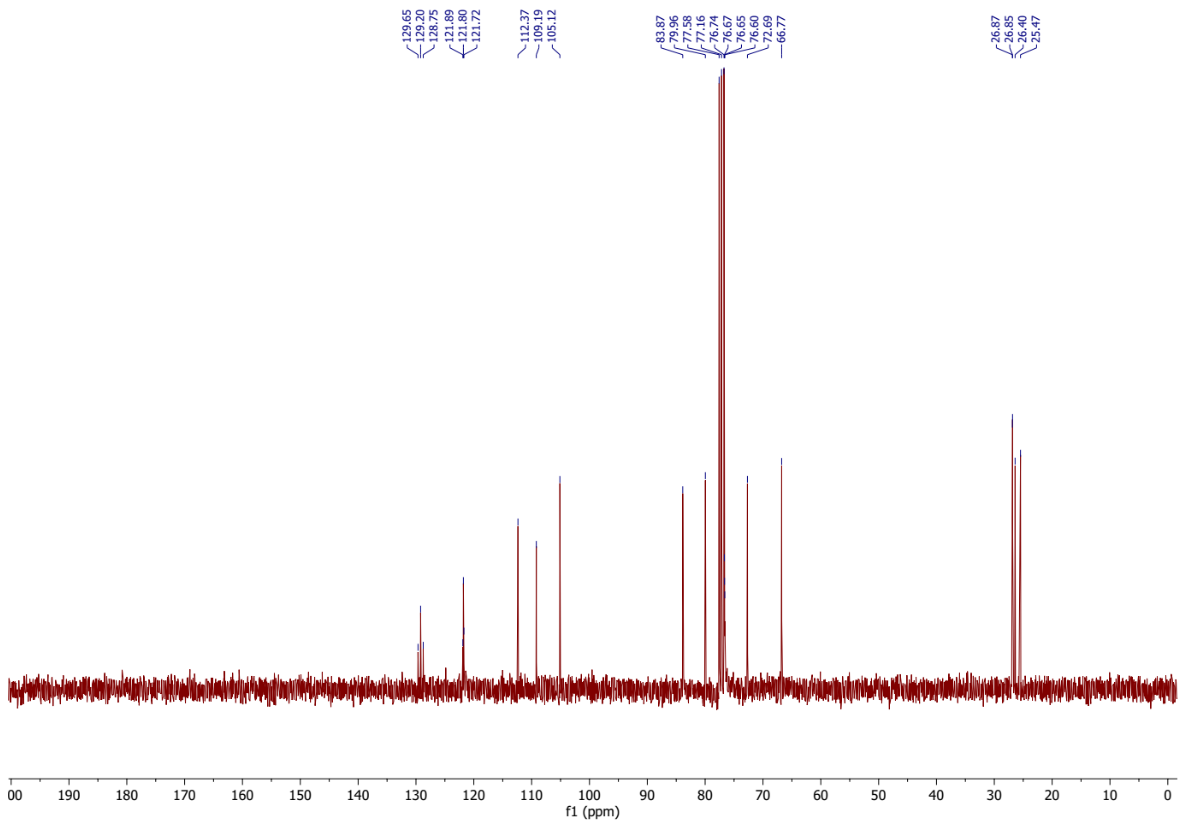

Supplementary Figure 177. ^{13}C NMR spectra of compound 50

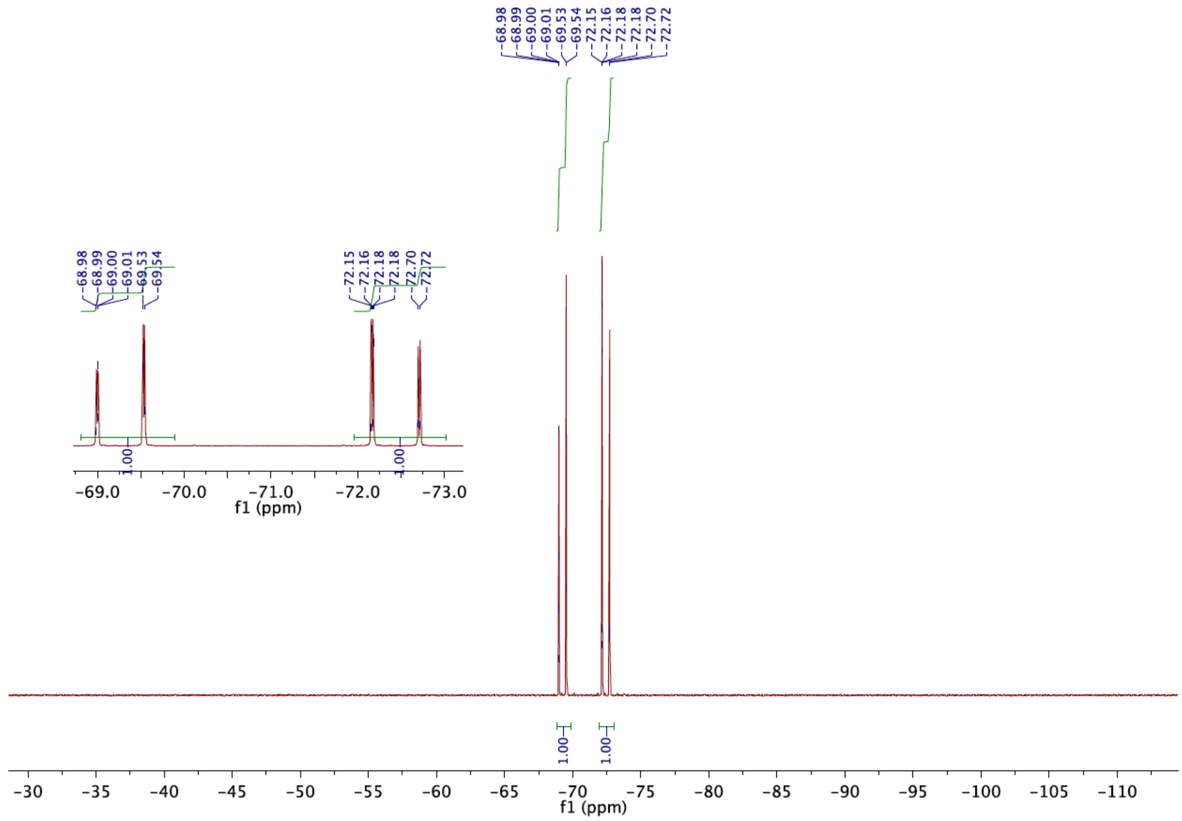

Supplementary Figure 178. ^{13}C NMR spectra of compound 50

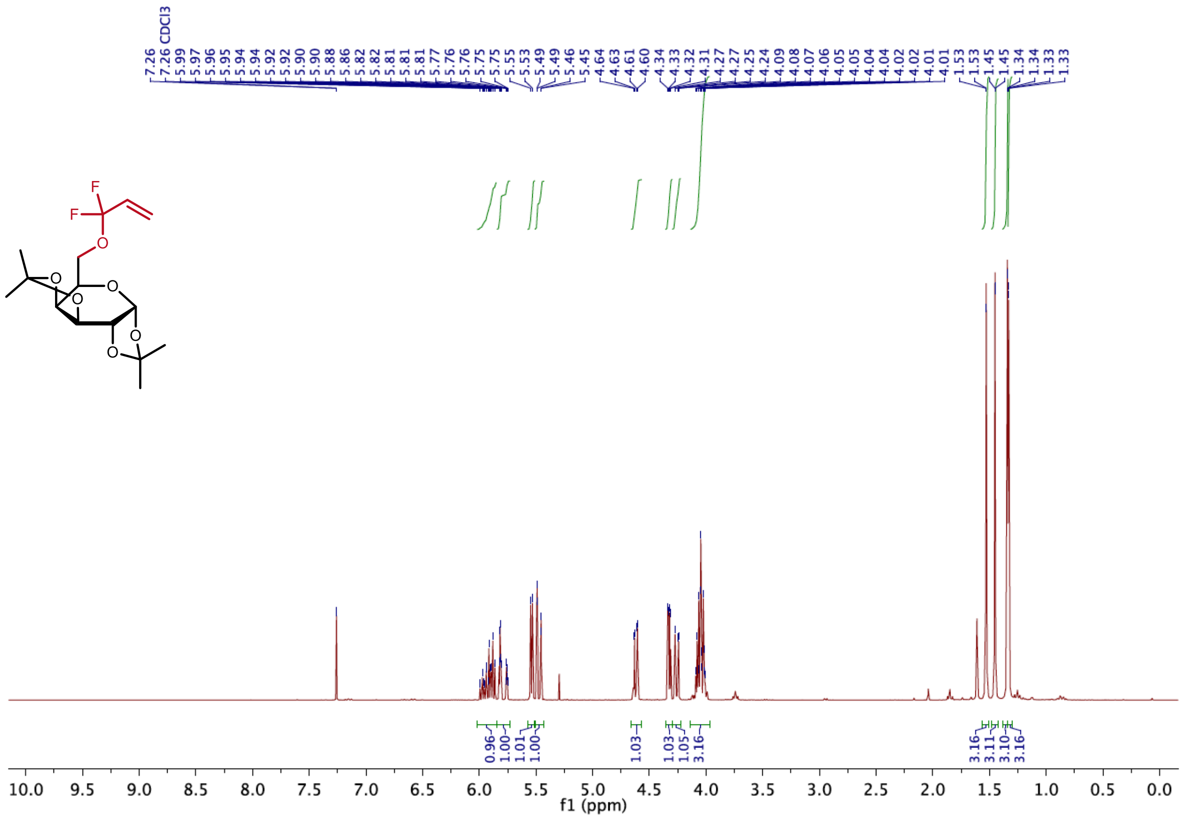

Supplementary Figure 179. ^{1}H NMR spectra of compound 51

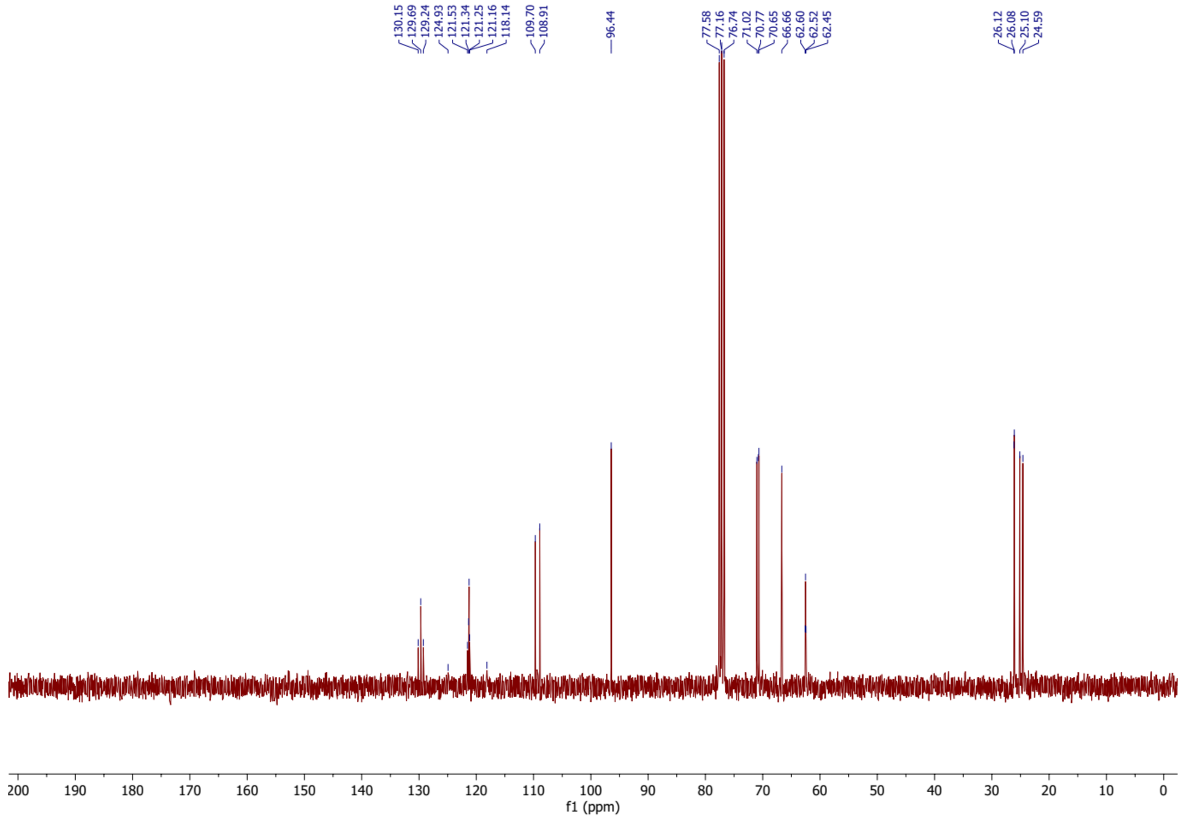

Supplementary Figure 180. ^{13}C NMR spectra of compound 51

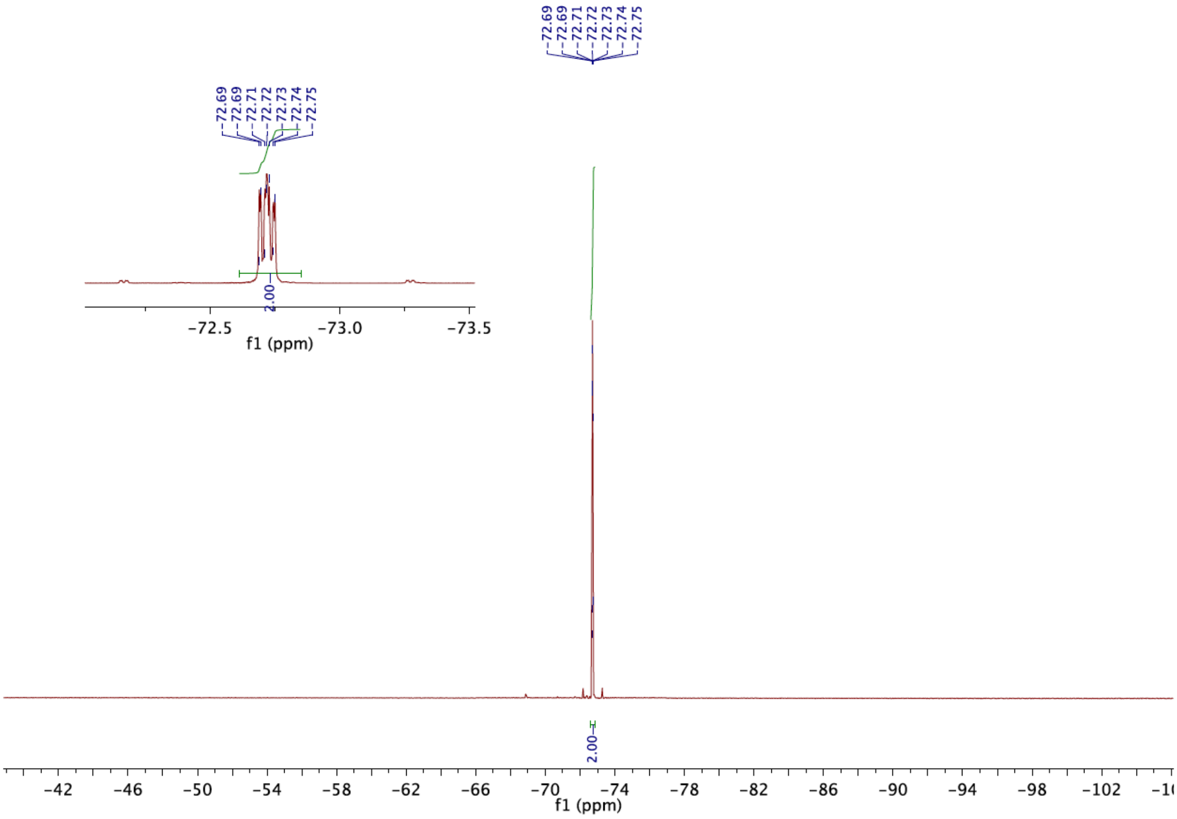

Supplementary Figure 181. ^{19}F NMR spectra of compound 51

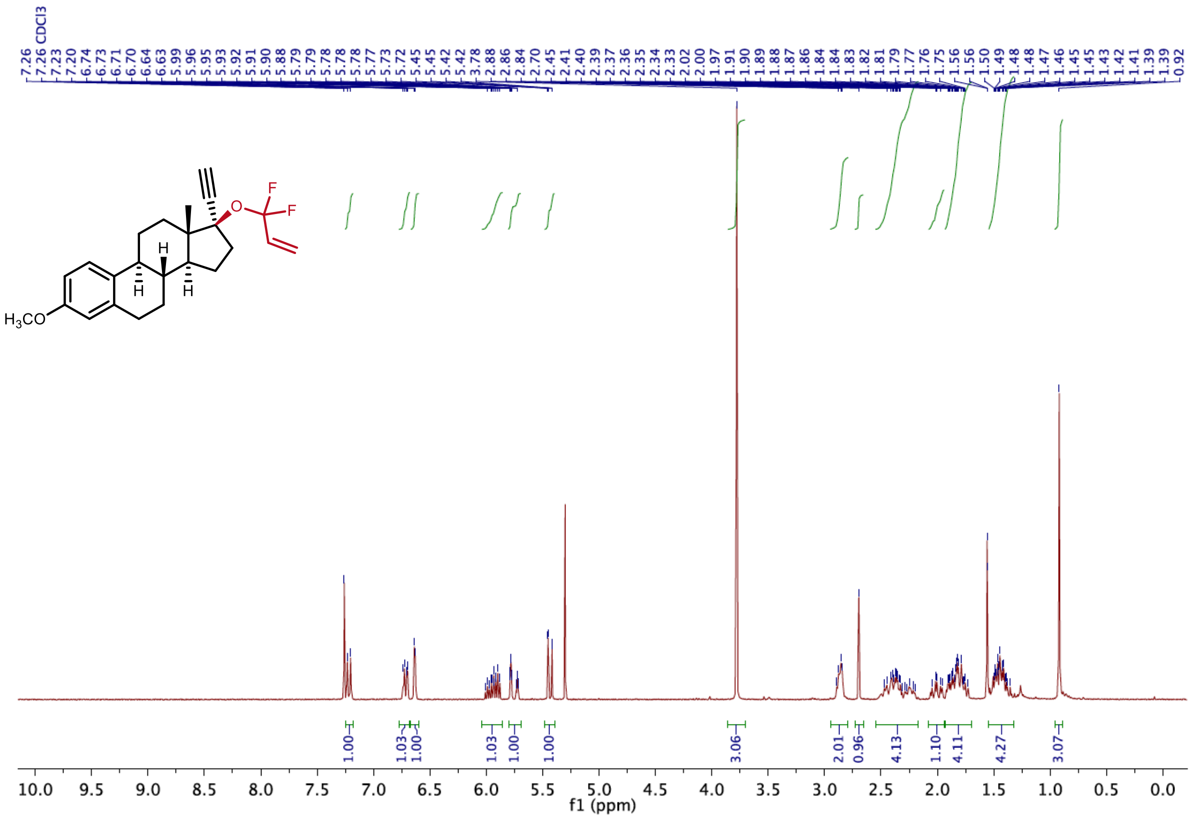

Supplementary Figure 182. ^{1}H NMR spectra of compound 52

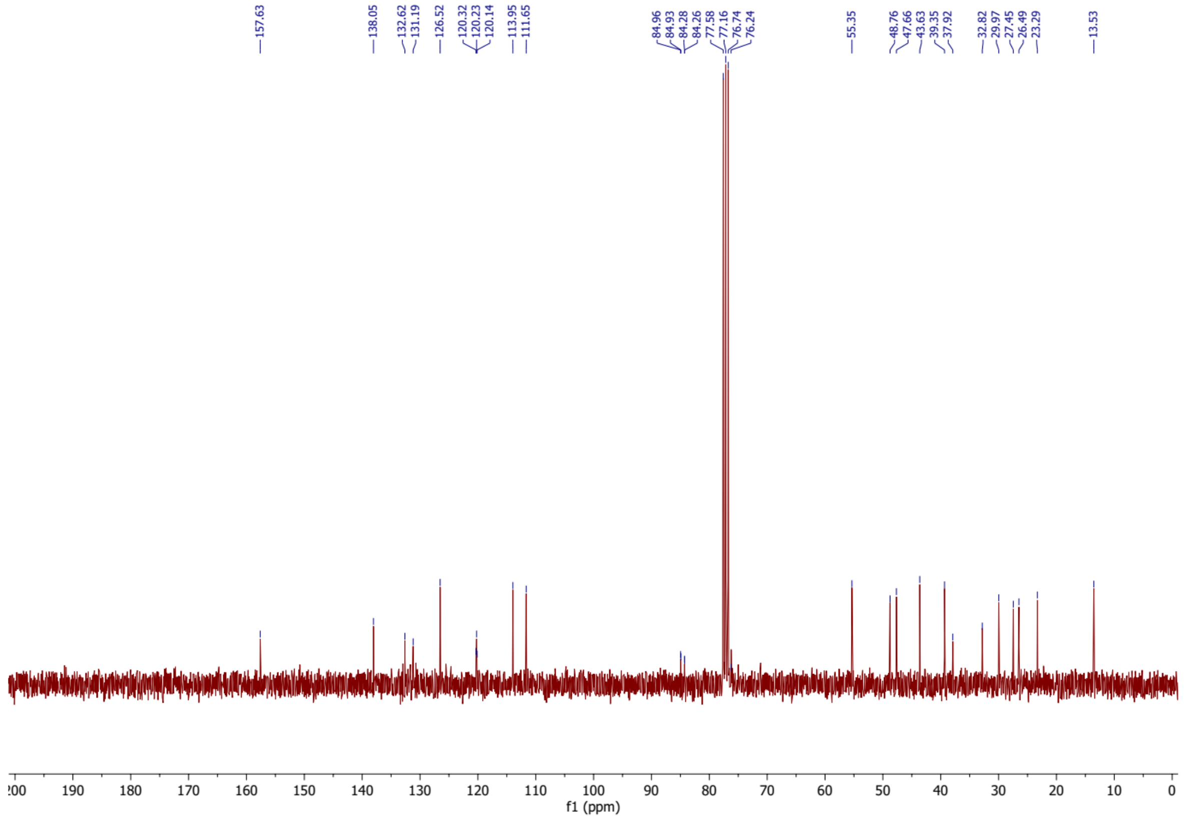

Supplementary Figure 183. 19C NMR spectra of compound 52

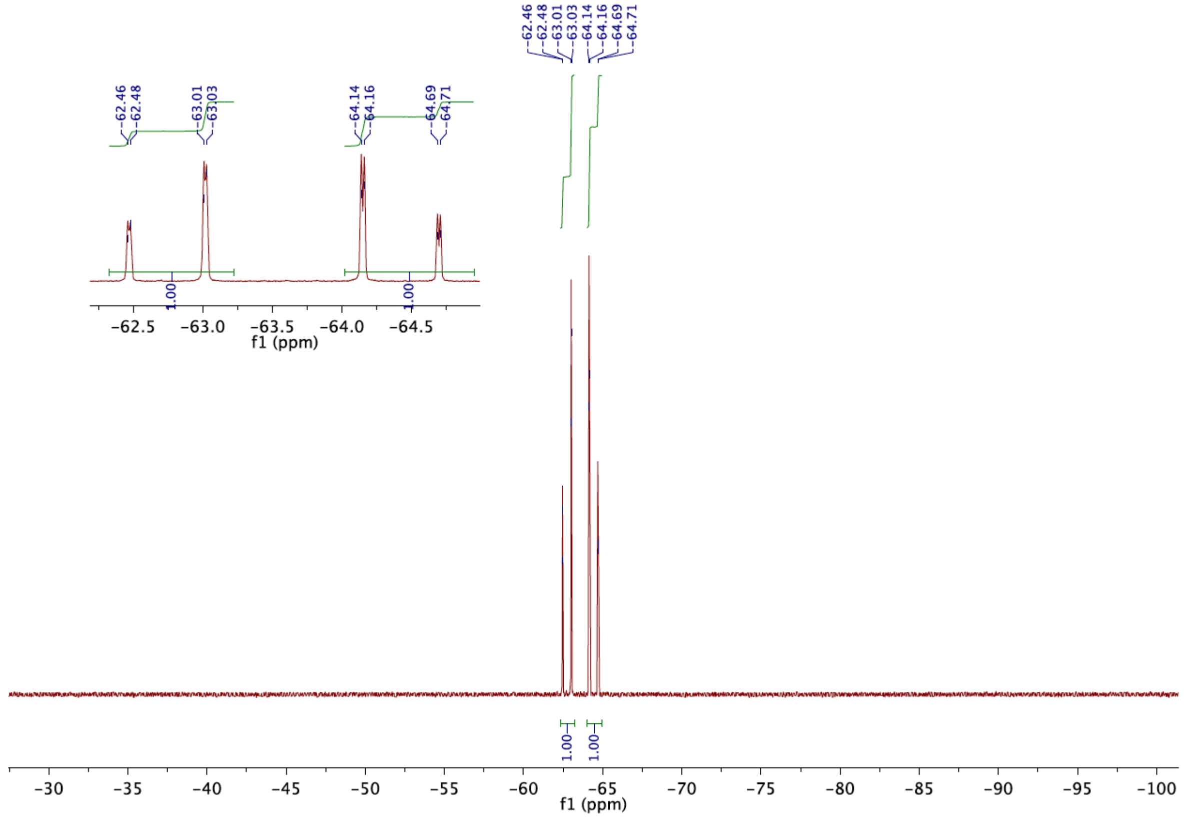

Supplementary Figure 184. 19F NMR spectra of compound 52

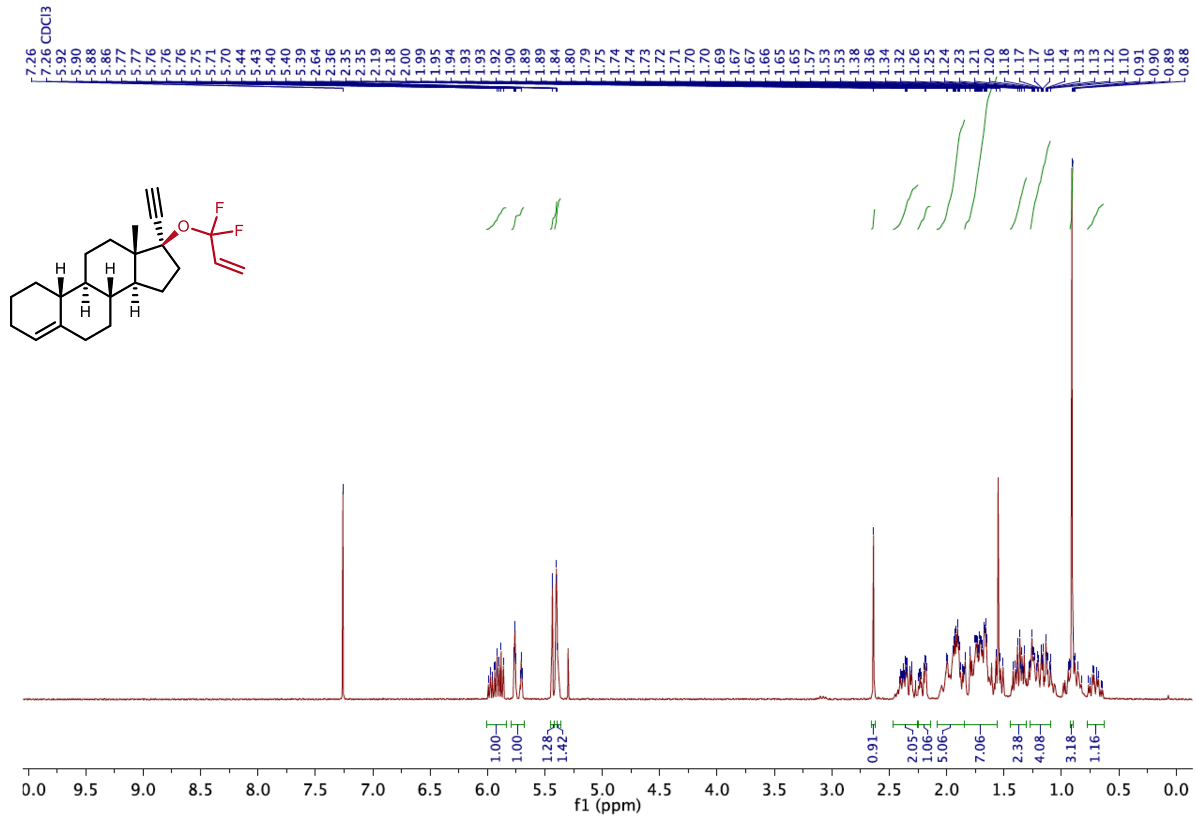

Supplementary Figure 185. ^{1}H NMR spectra of compound 53

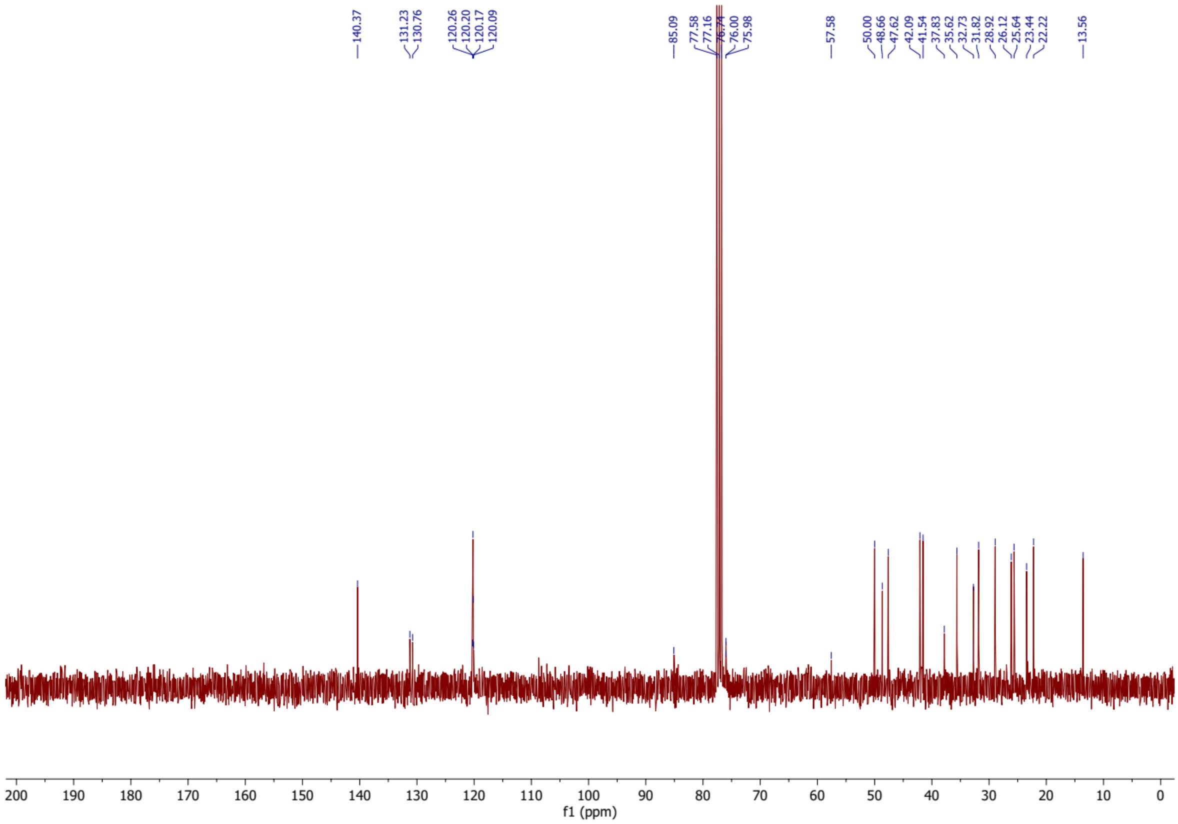

Supplementary Figure 186. ^{13}C NMR spectra of compound 53

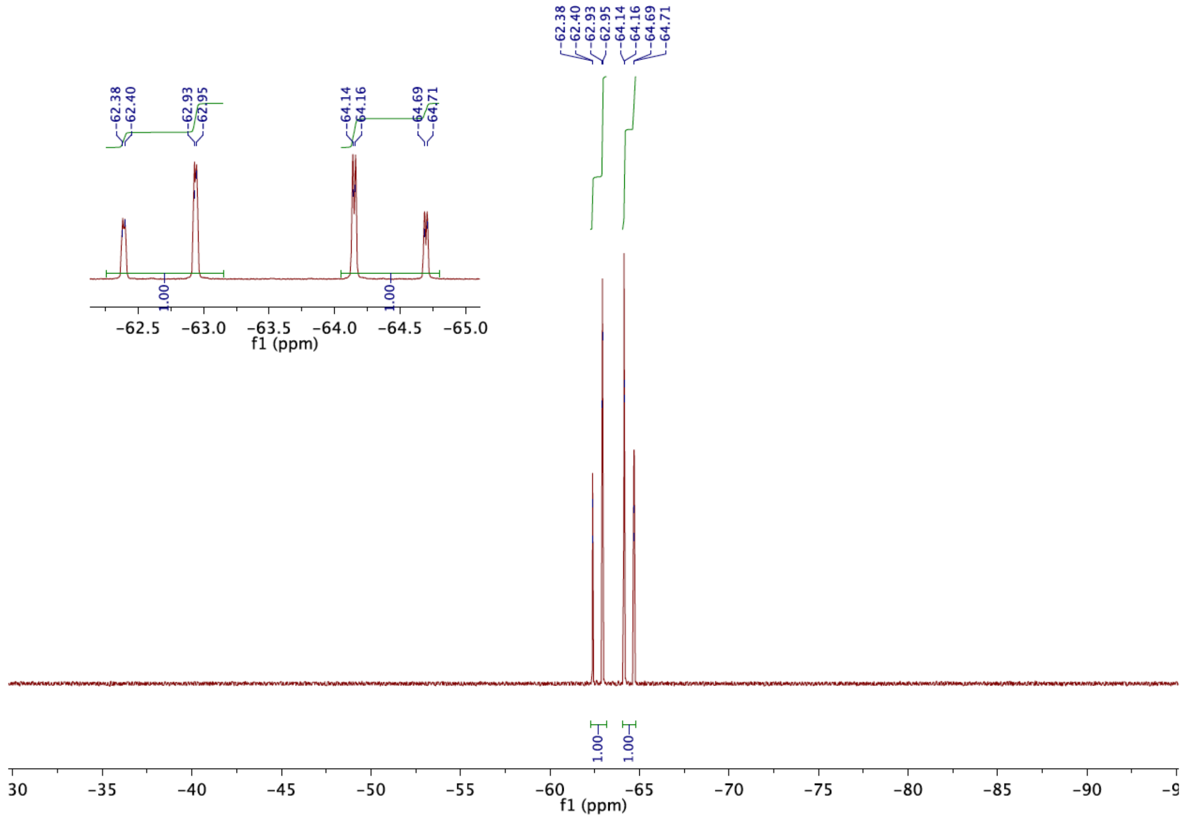

Supplementary Figure 187. ^{19}F NMR spectra of compound 53

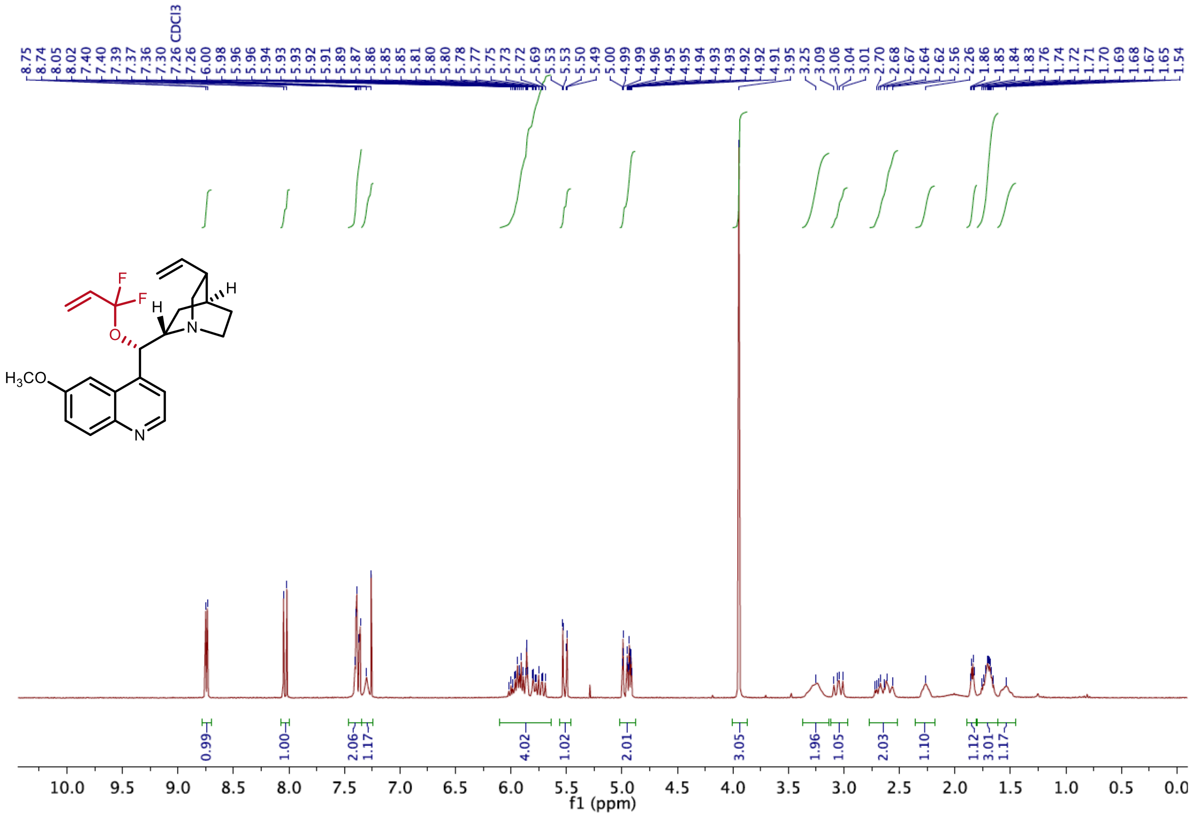

Supplementary Figure 188. ^{1}H NMR spectra of compound 54

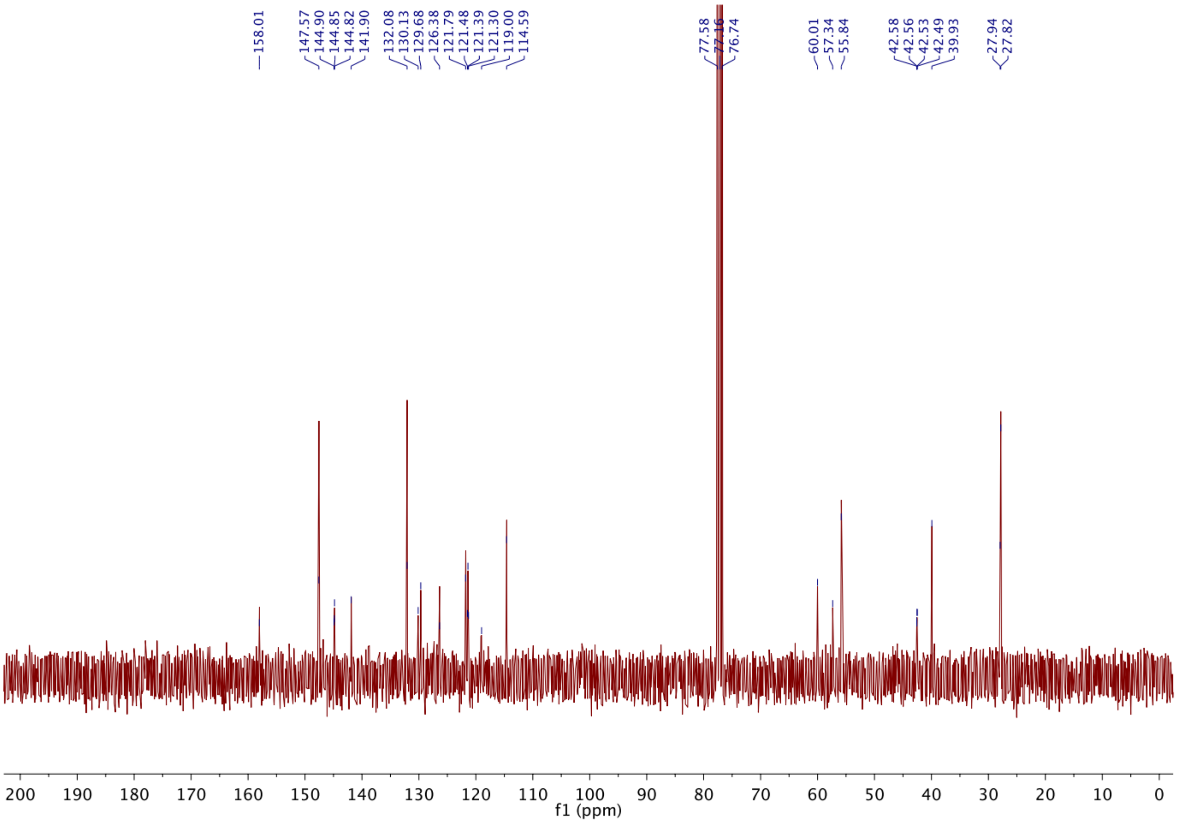

Supplementary Figure 189. ^{13}**C NMR spectra of compound 54**

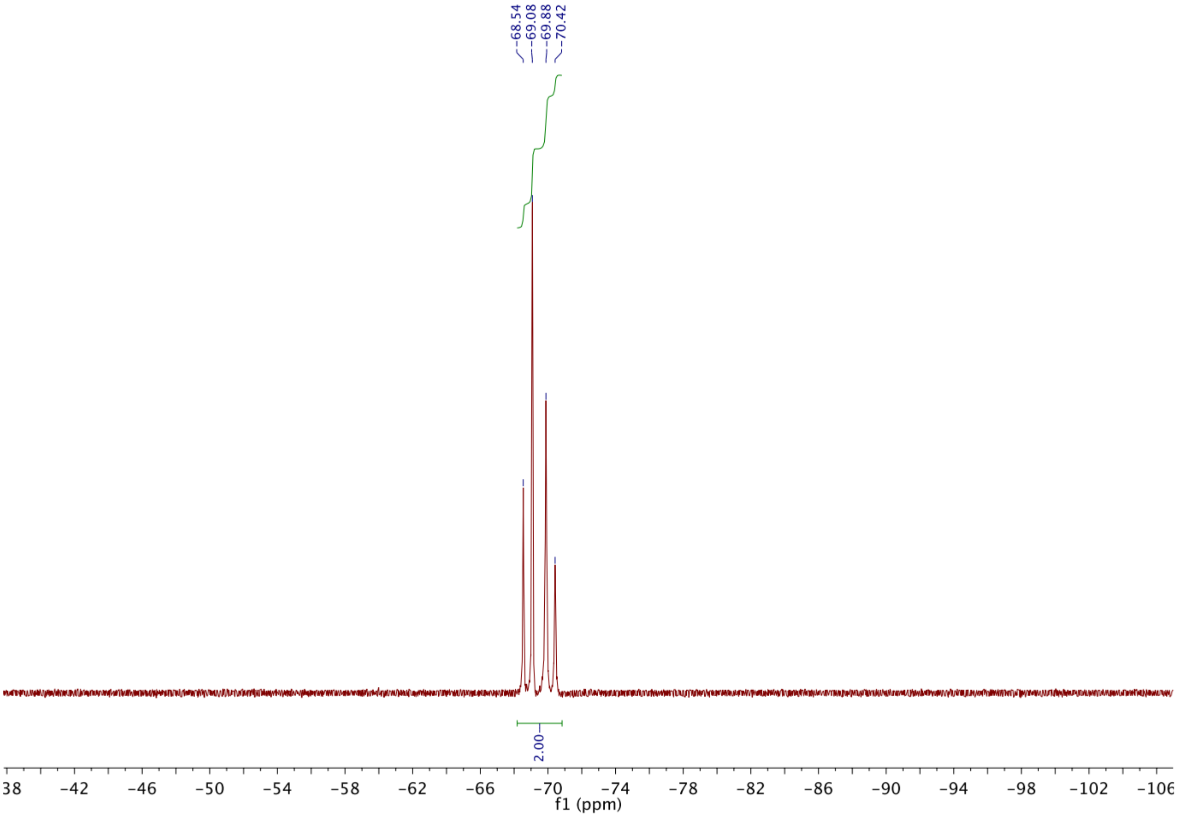

Supplementary Figure 190. ^{19}**F NMR spectra of compound 54**

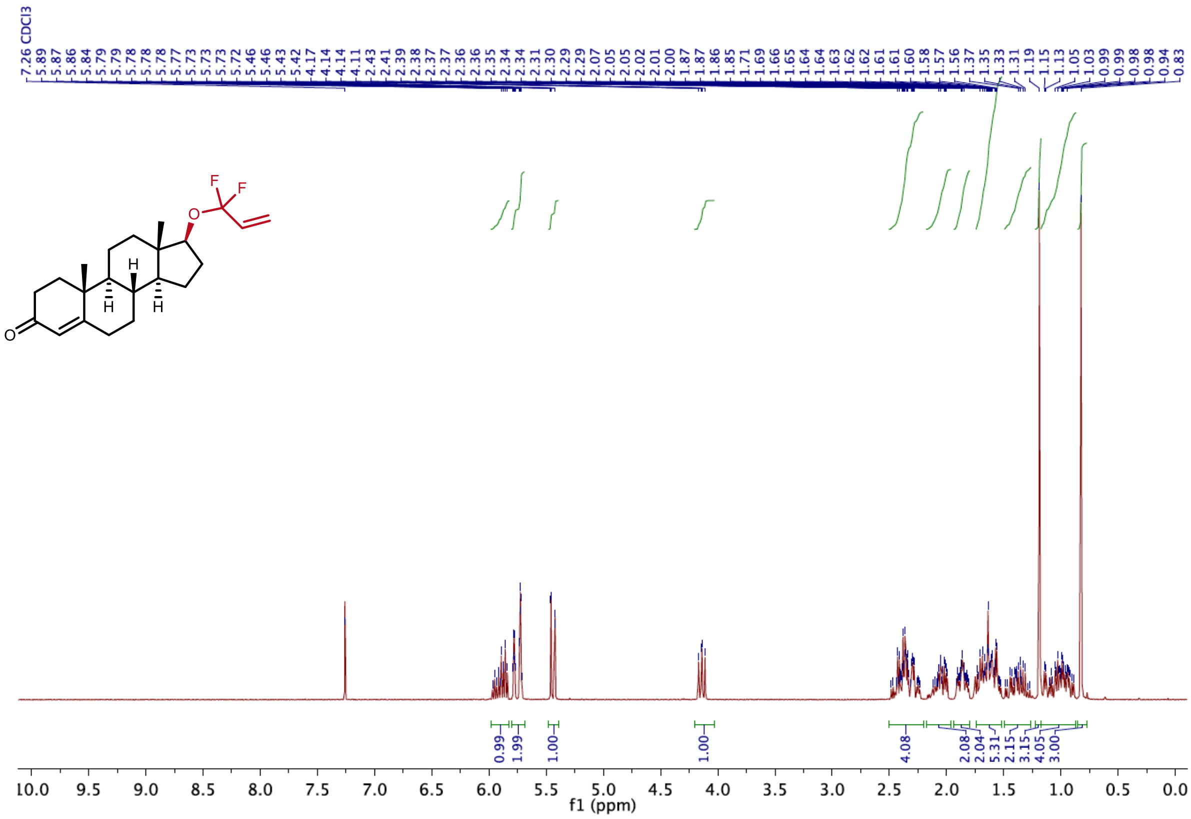

Supplementary Figure 191. ^{1}H NMR spectra of compound 55

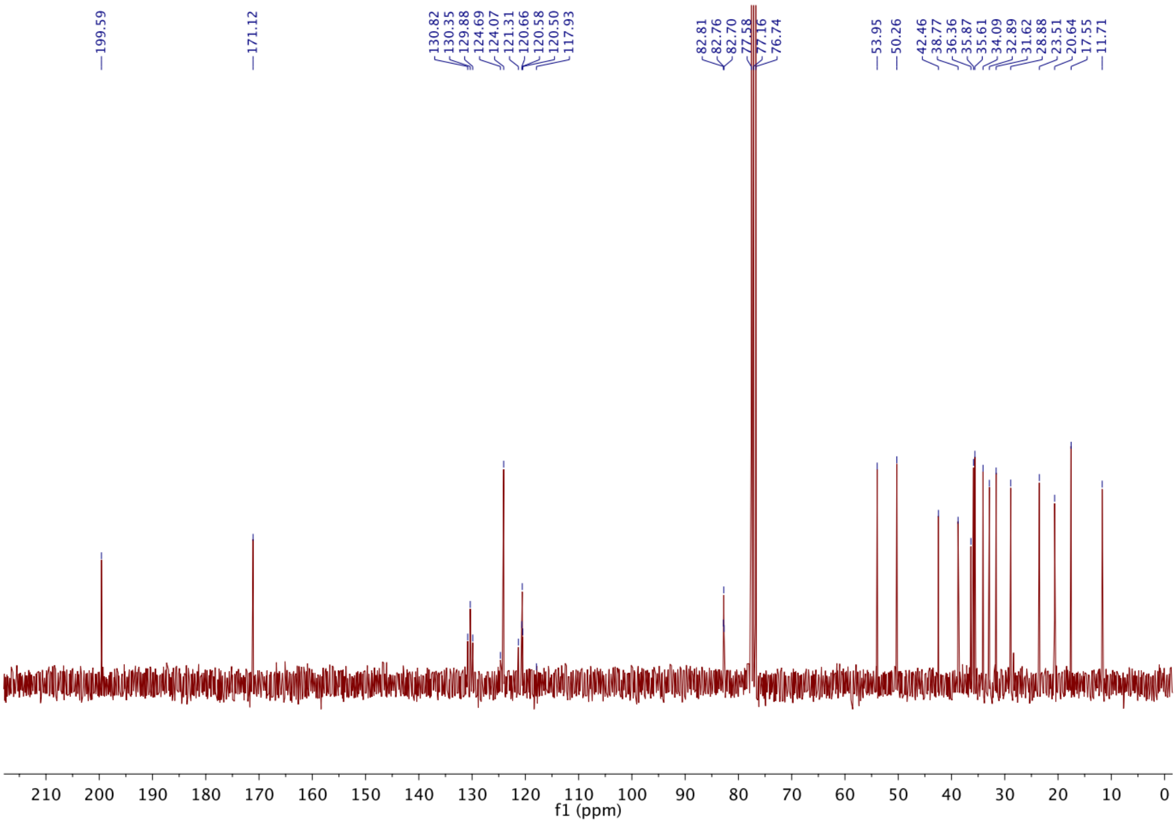

Supplementary Figure 192. ^{13}C NMR spectra of compound 55

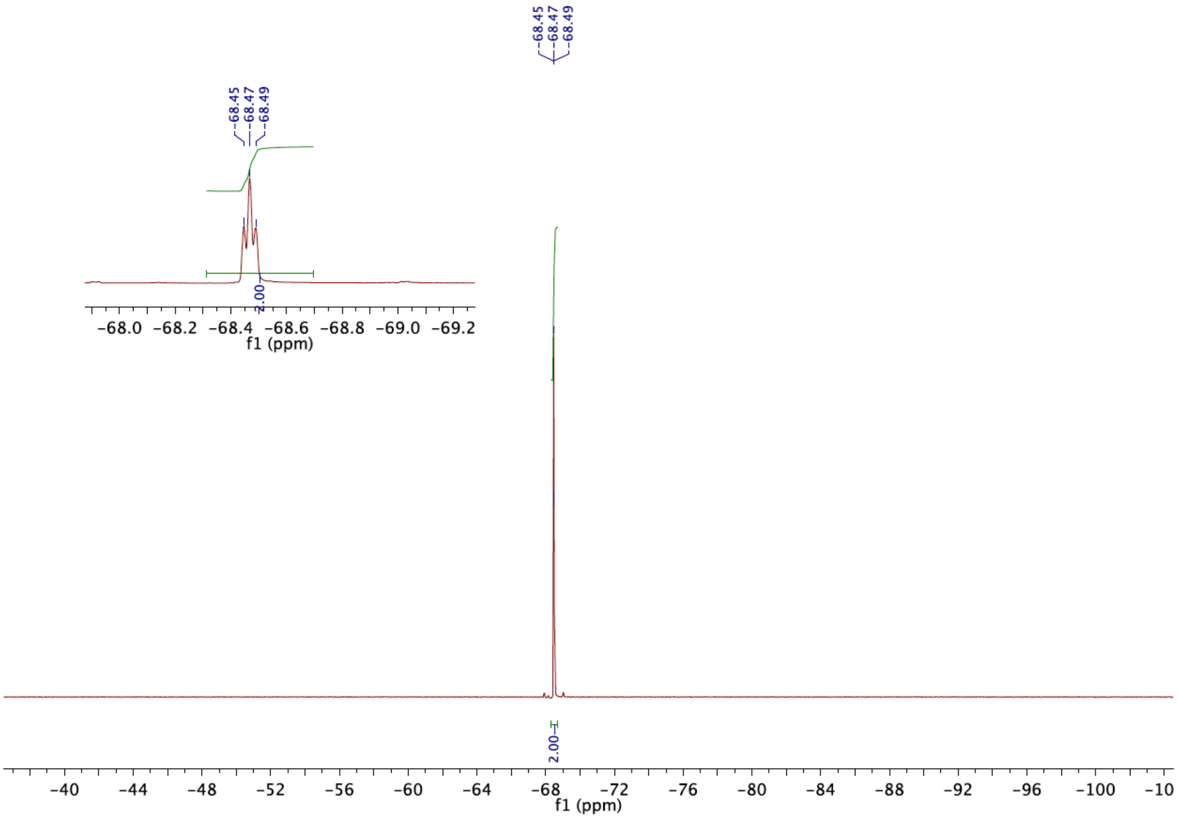

Supplementary Figure 193. ^{19}F NMR spectra of compound 55

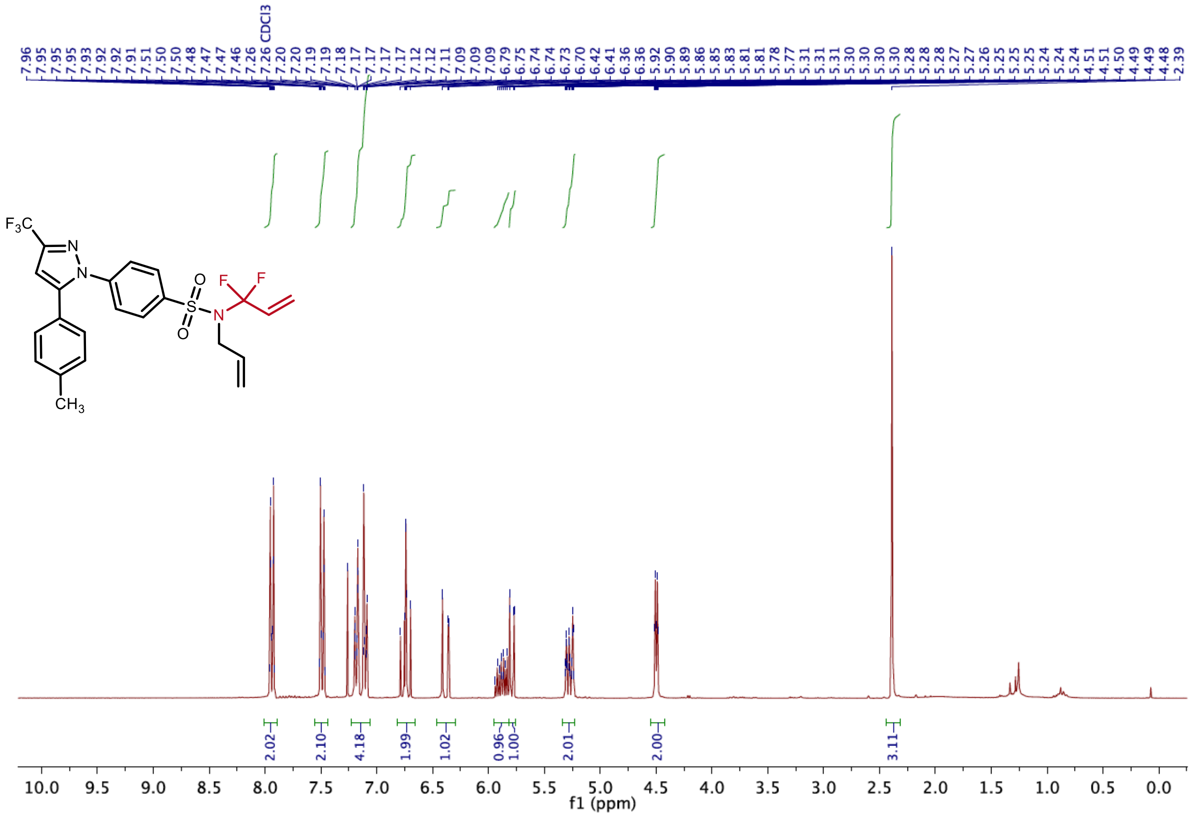

Supplementary Figure 194. ^{1}H NMR spectra of compound 56

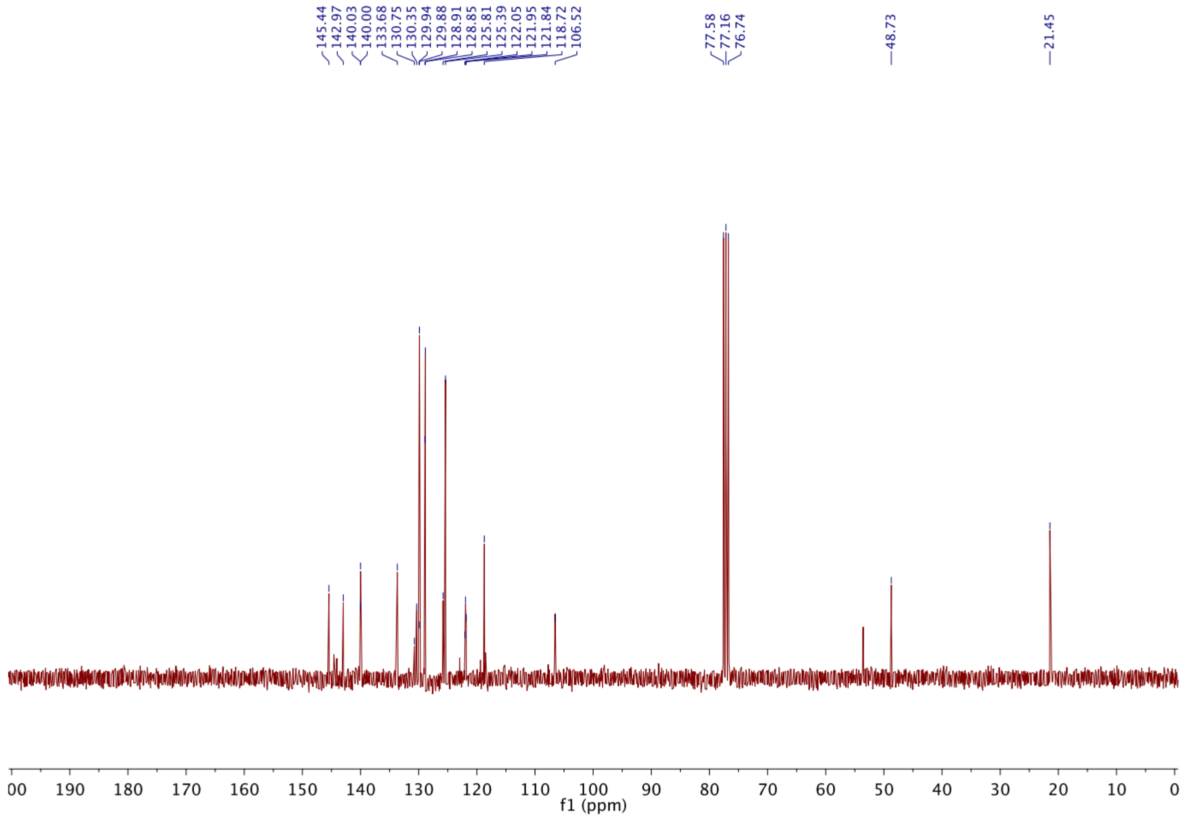

Supplementary Figure 195. ^{13}C NMR spectra of compound 56

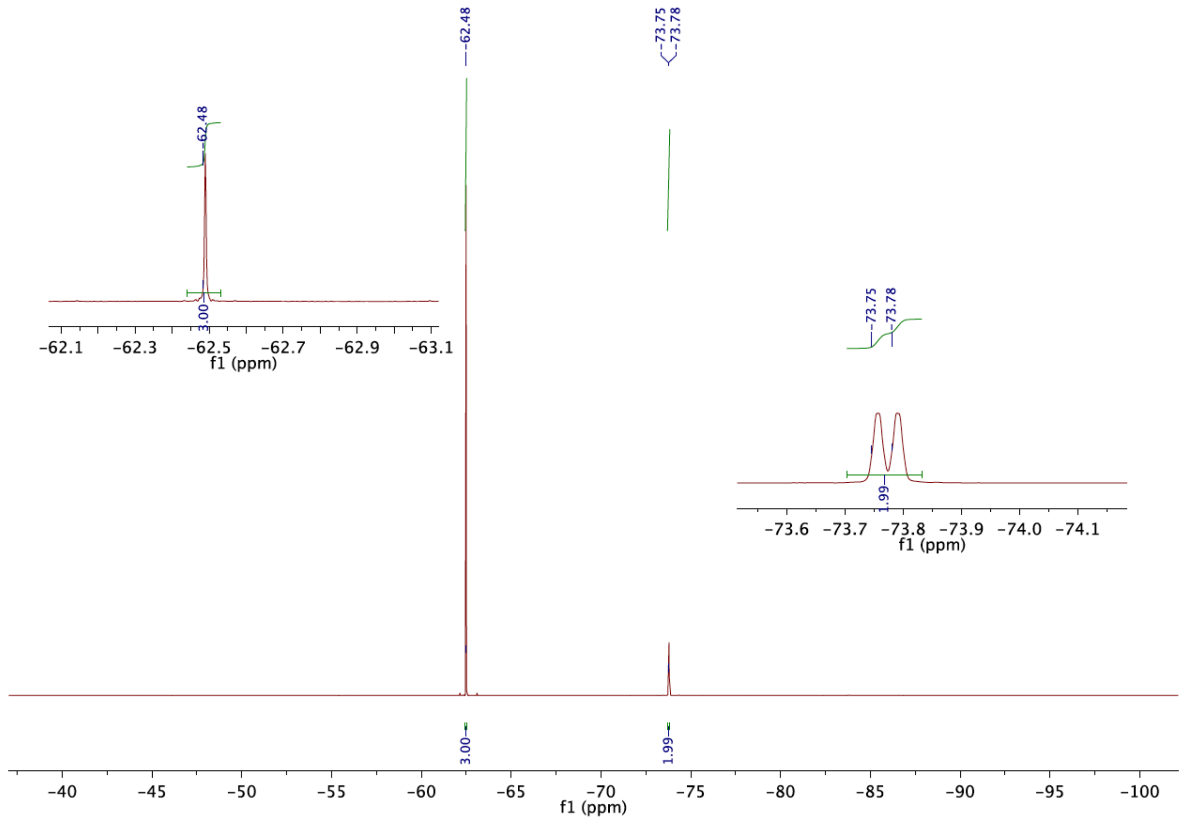

Supplementary Figure 196. ^{19}F NMR spectra of compound 56

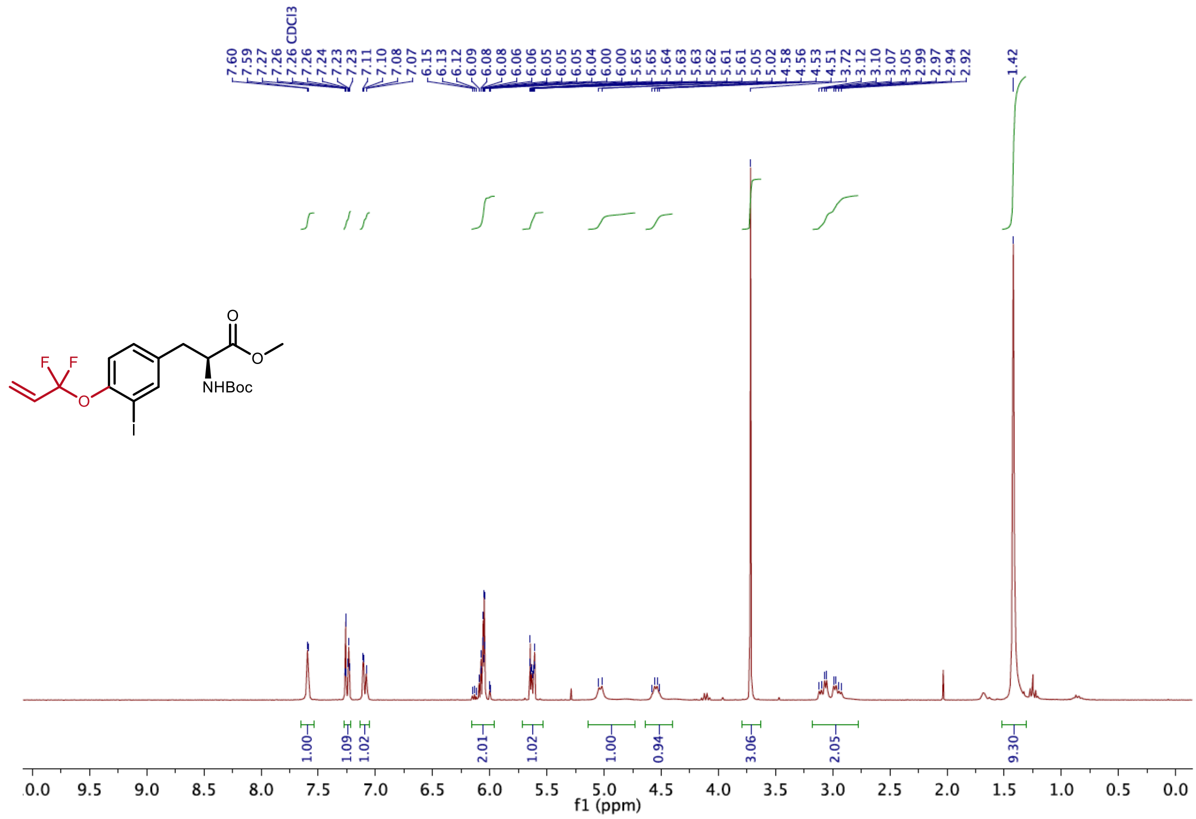

Supplementary Figure 197. ^{1}H NMR spectra of compound 57

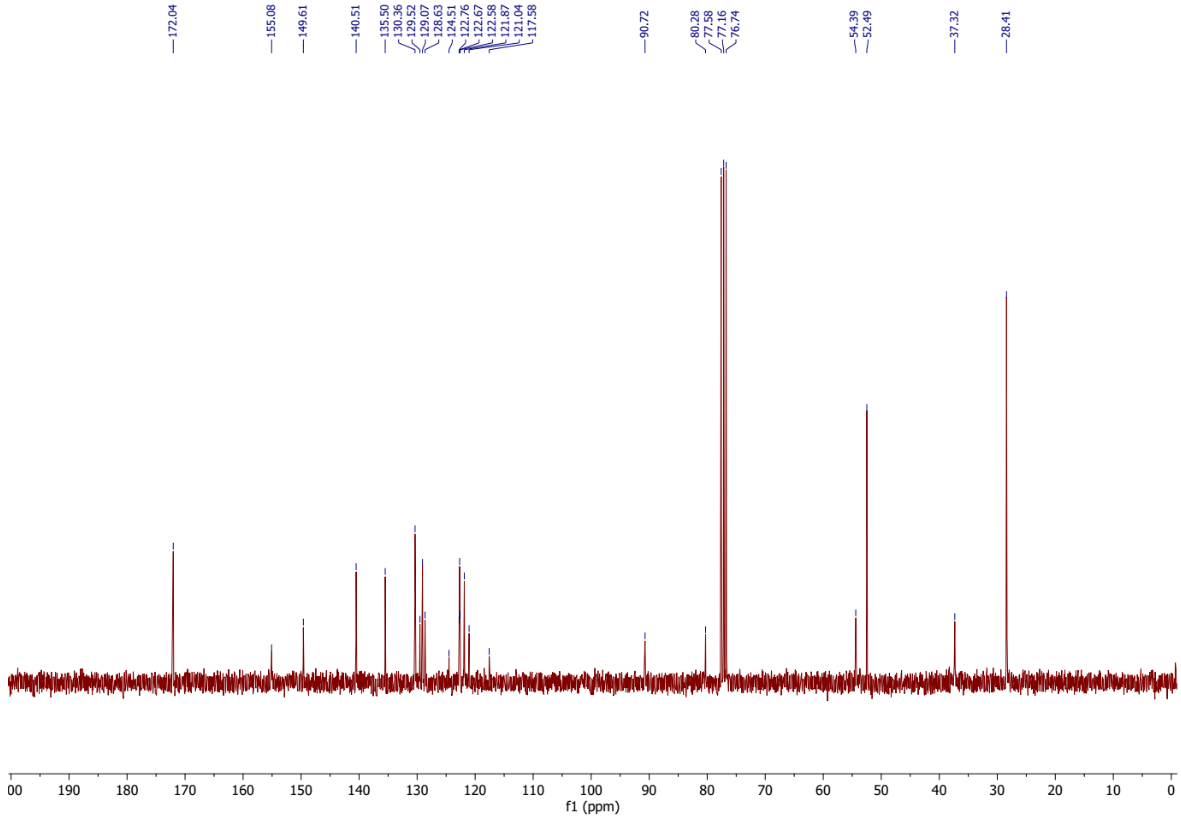

Supplementary Figure 198. ^{13}C NMR spectra of compound 57

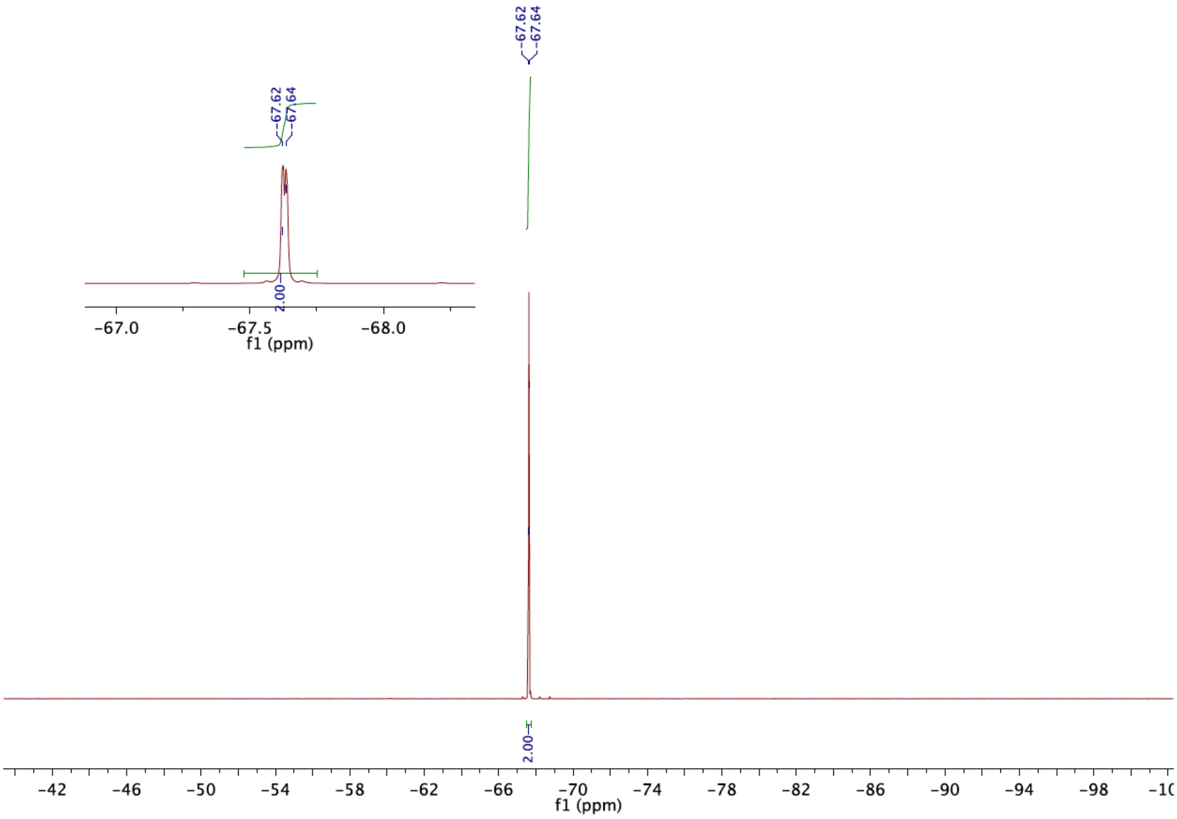

Supplementary Figure 199. ^{19}F NMR spectra of compound 57

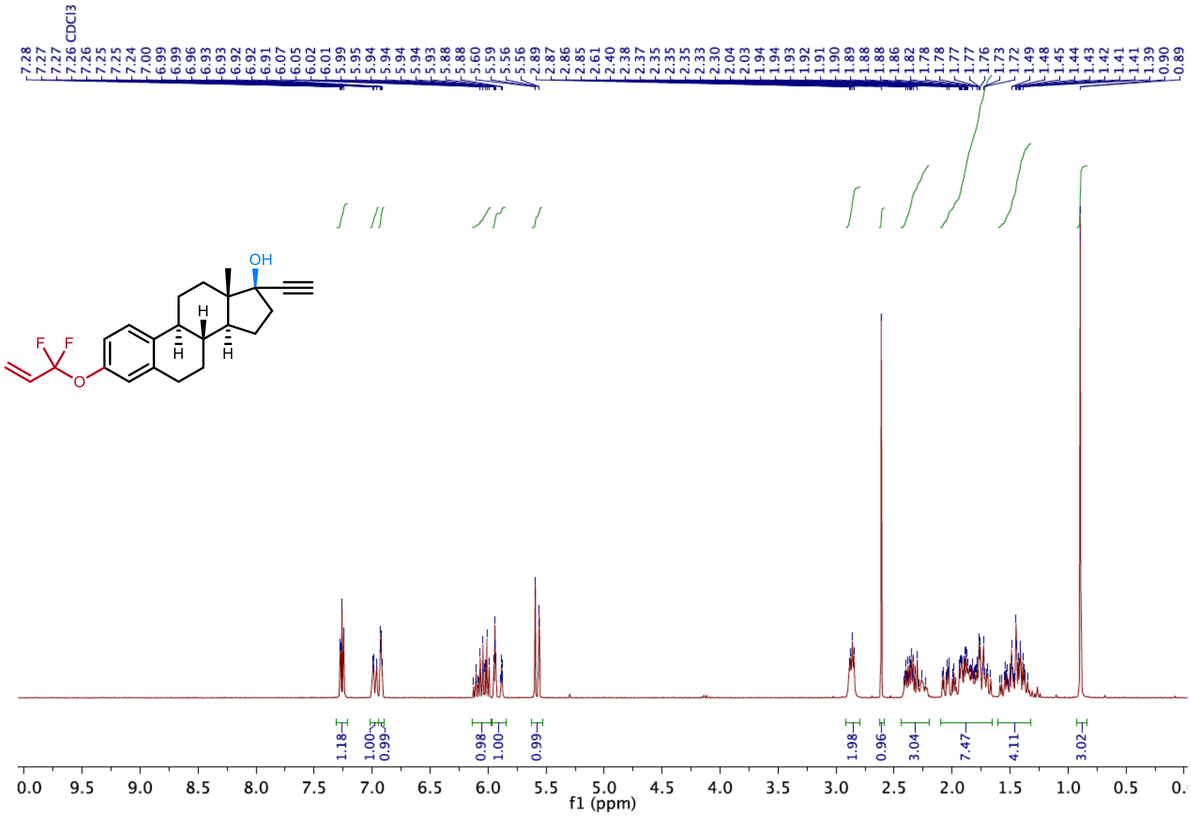

Supplementary Figure 200. ^{1}H NMR spectra of compound 58

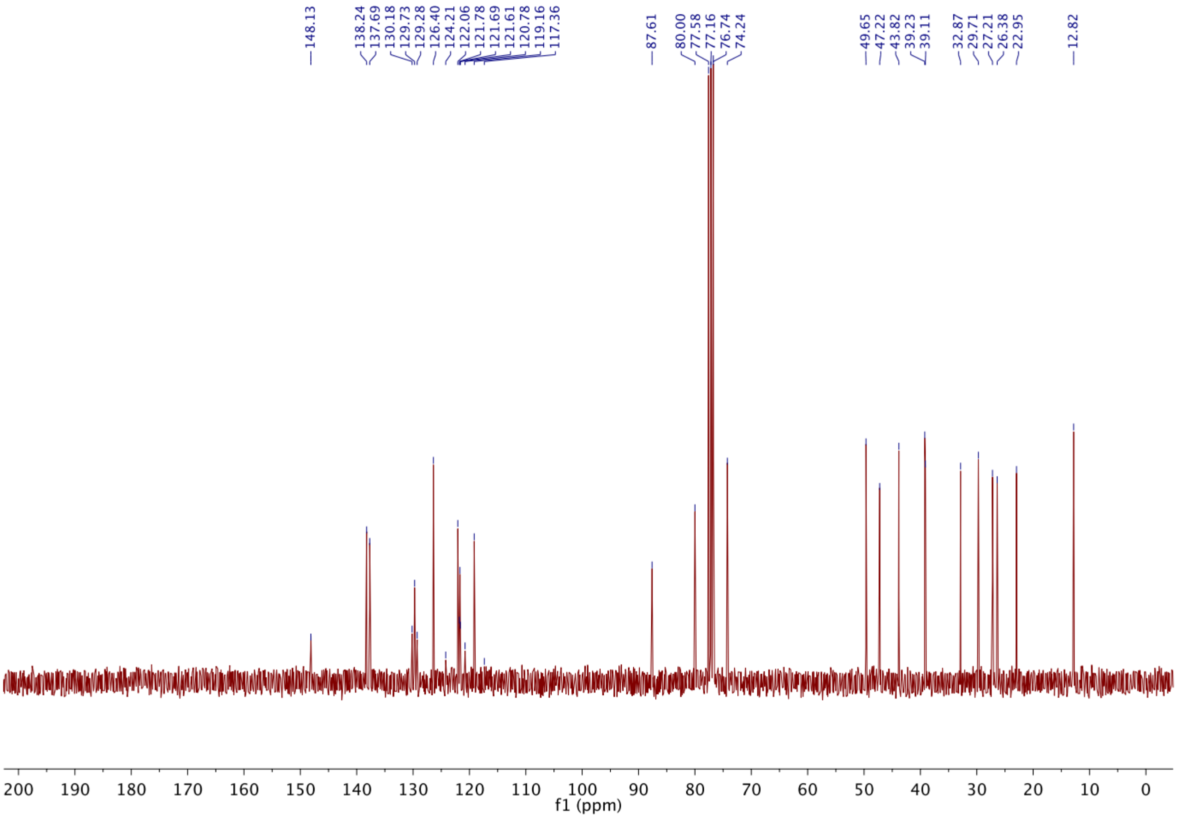

Supplementary Figure 201. ^{13}C NMR spectra of compound 58

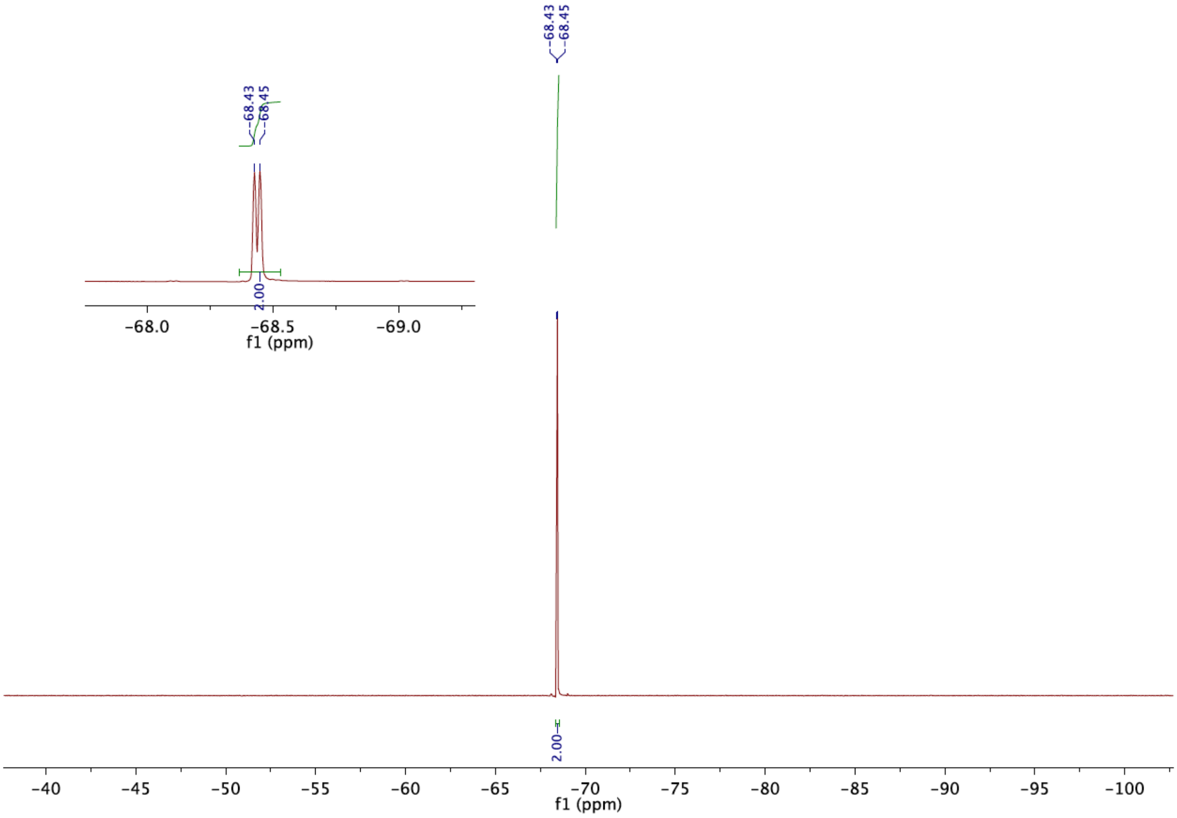

Supplementary Figure 202. ^{19}F NMR spectra of compound 58

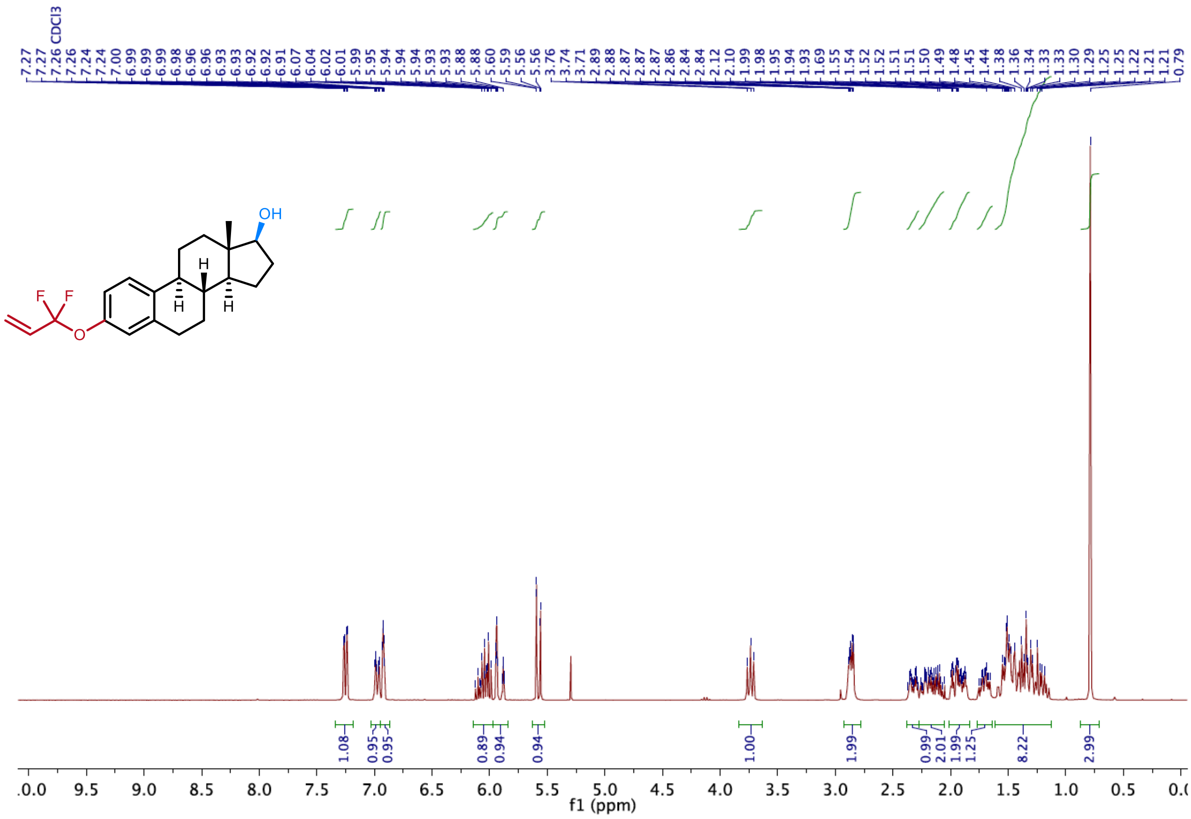

Supplementary Figure 203. ^{1}H NMR spectra of compound 59

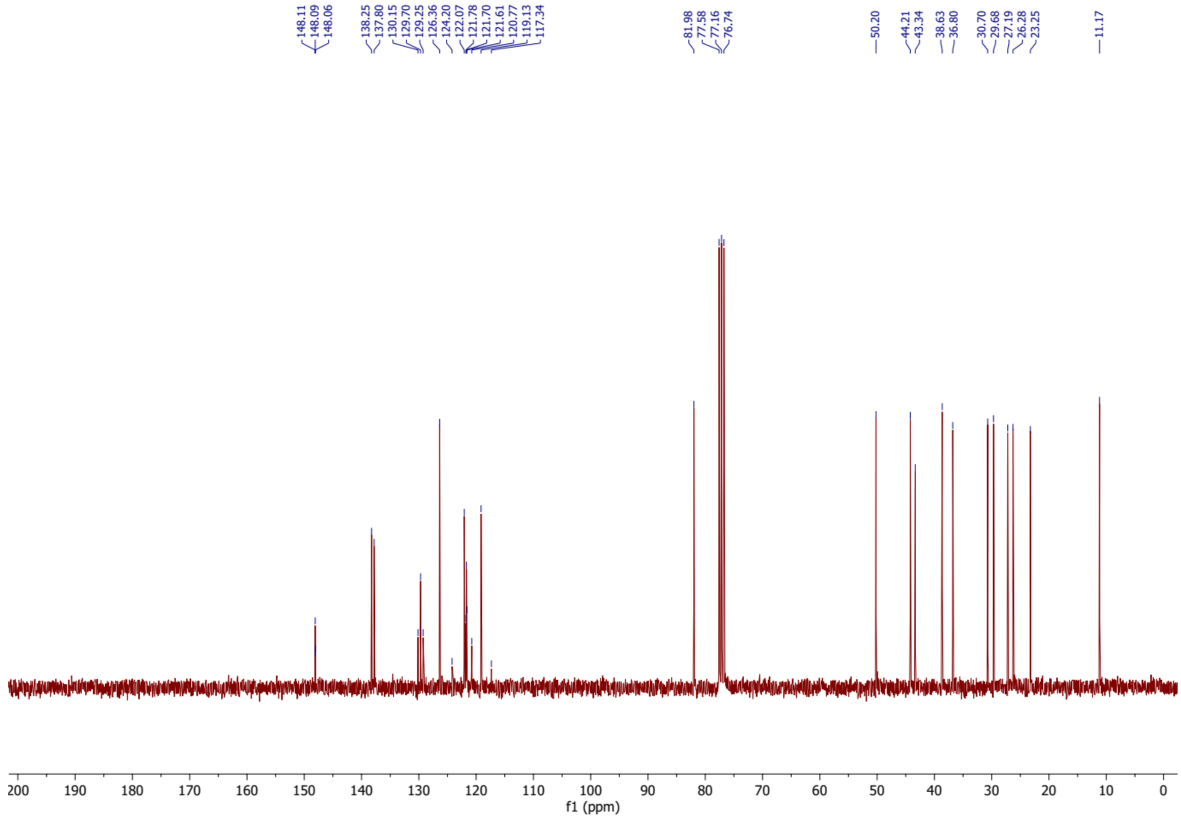

Supplementary Figure 204. ^{13}C NMR spectra of compound 59

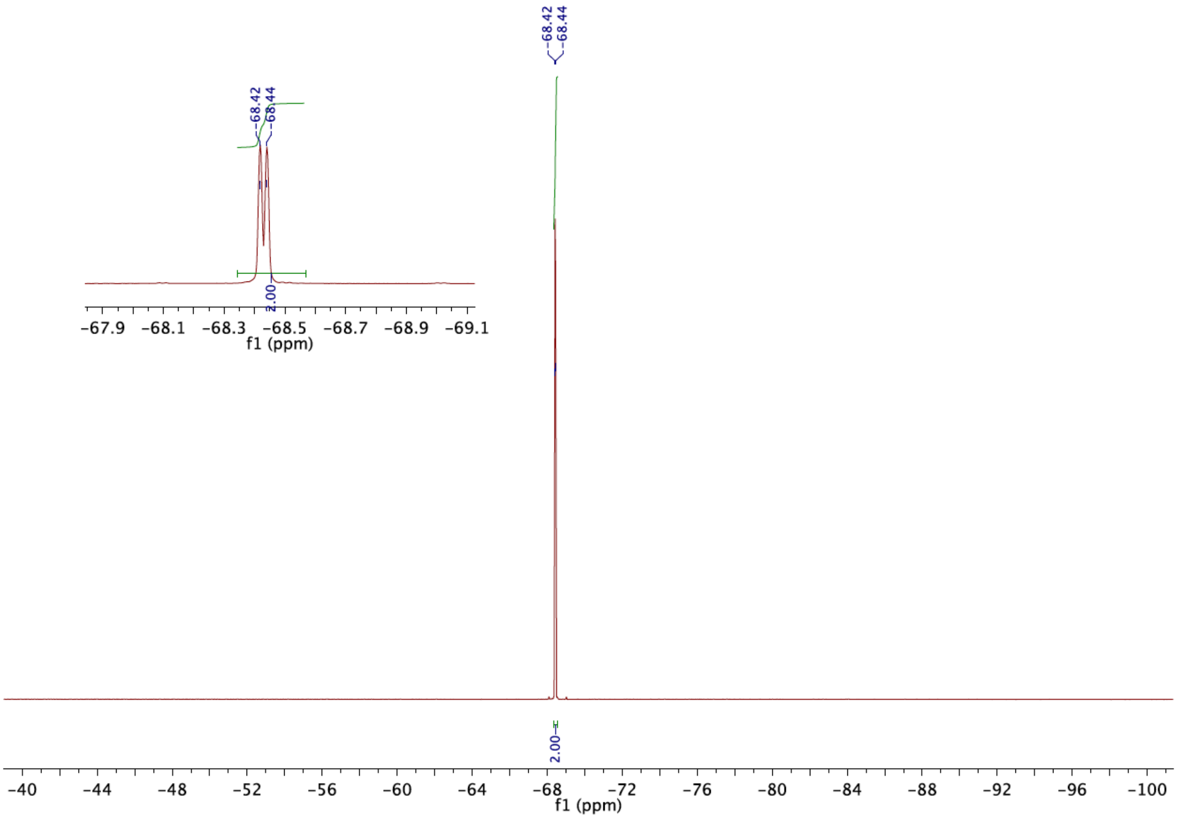

Supplementary Figure 205. ^{19}F NMR spectra of compound 59

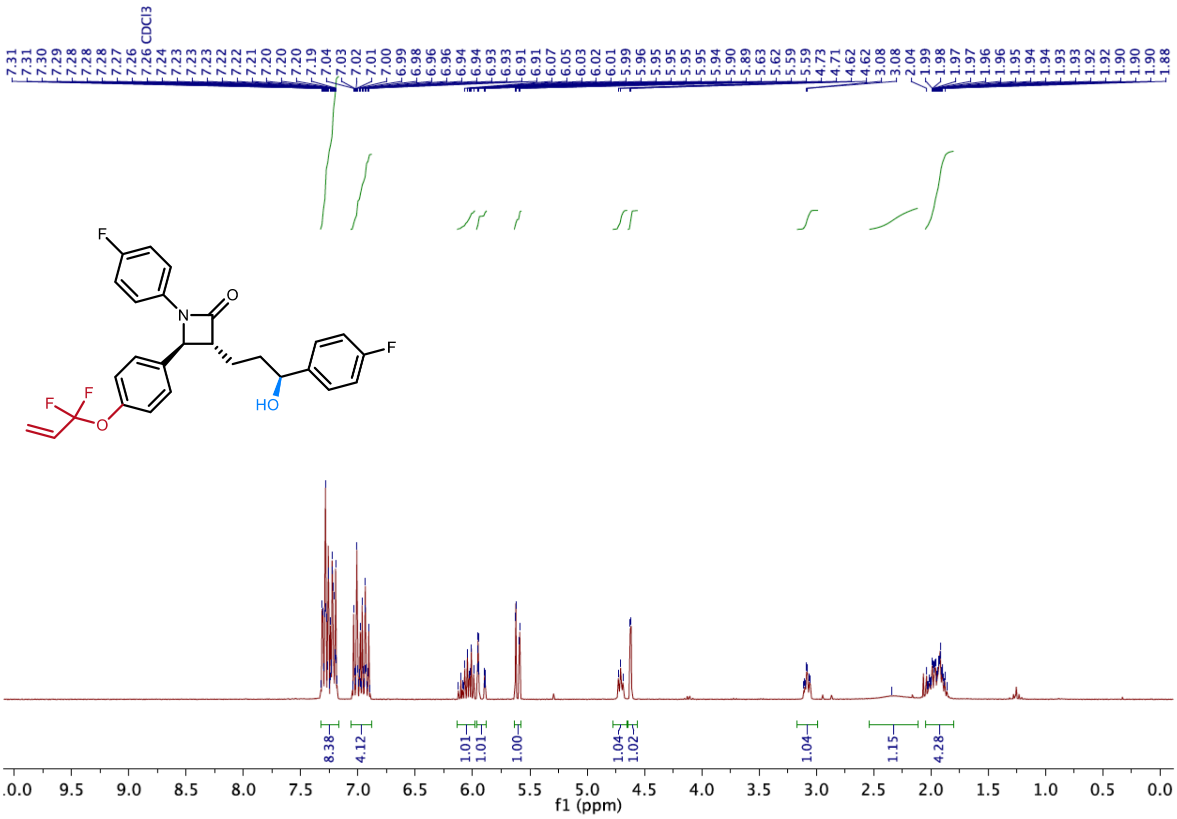

Supplementary Figure 206. ^{1}H NMR spectra of compound 60

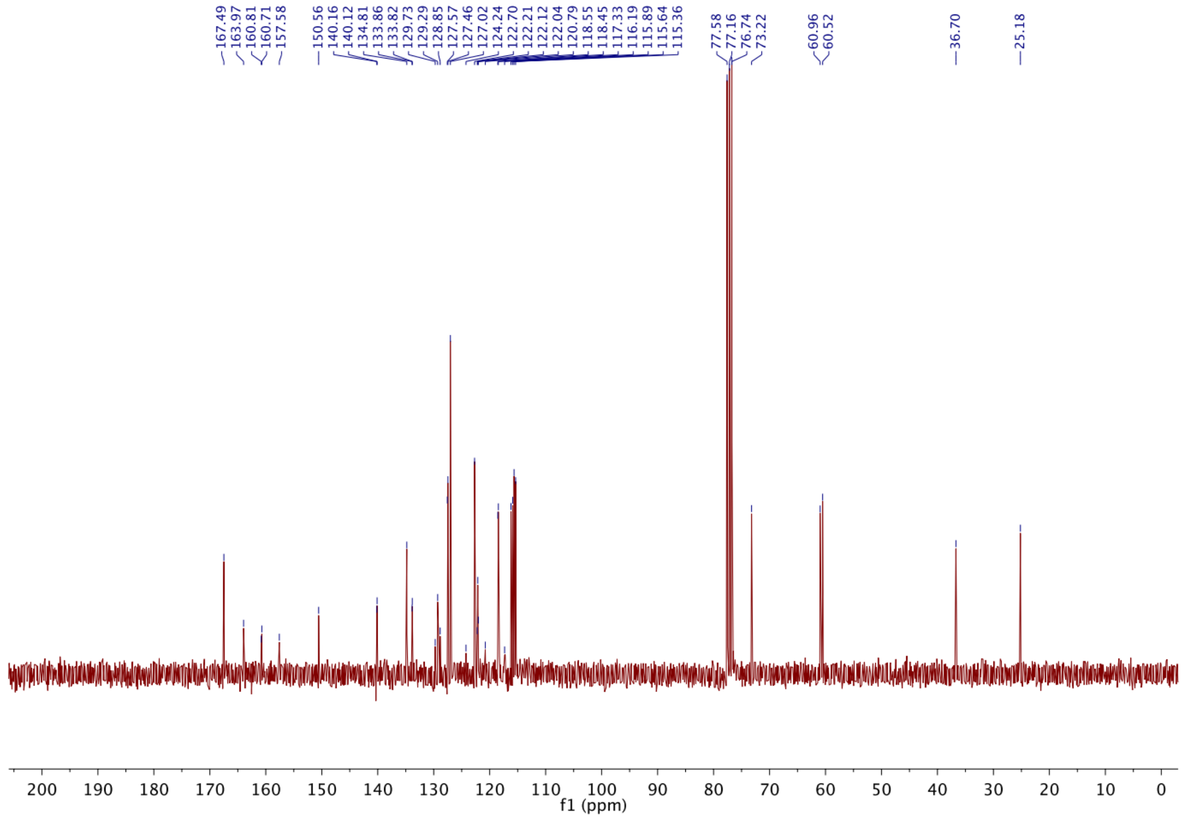

Supplementary Figure 207. ^{13}C NMR spectra of compound 60

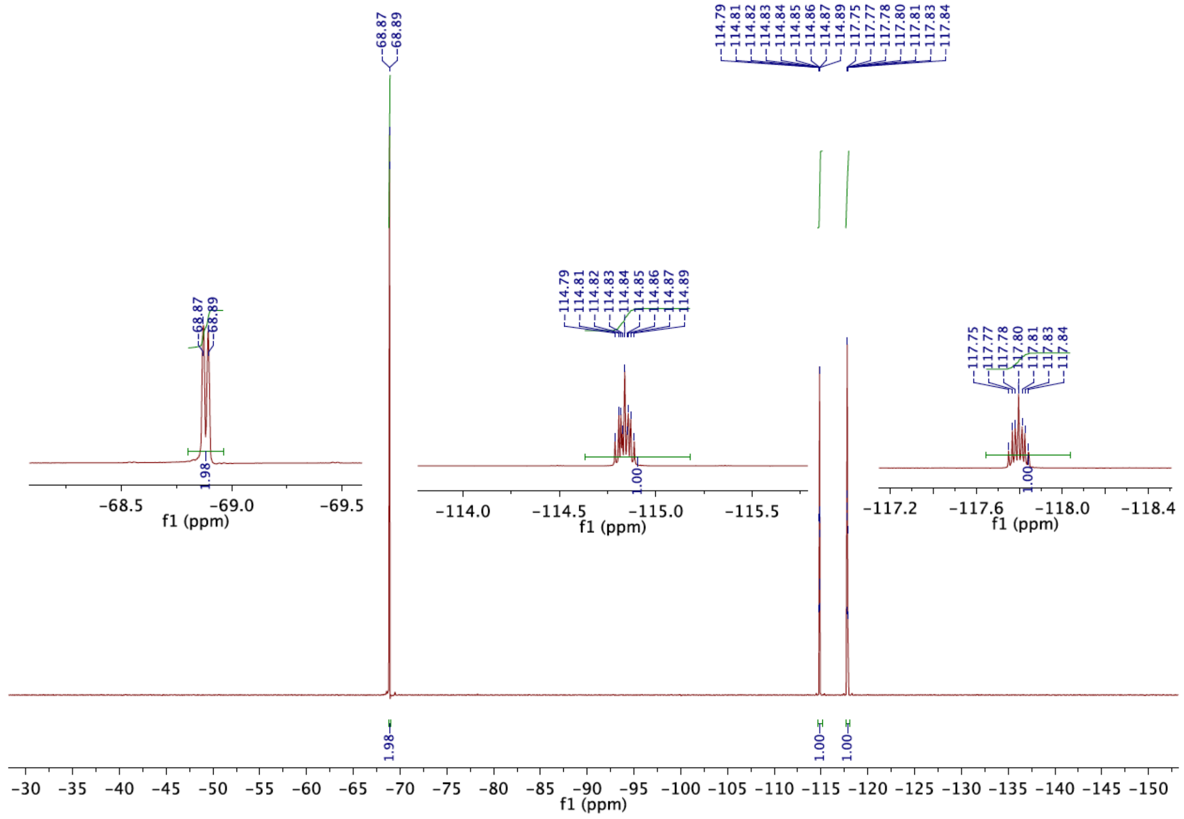

Supplementary Figure 208. ^{19}F NMR spectra of compound 60

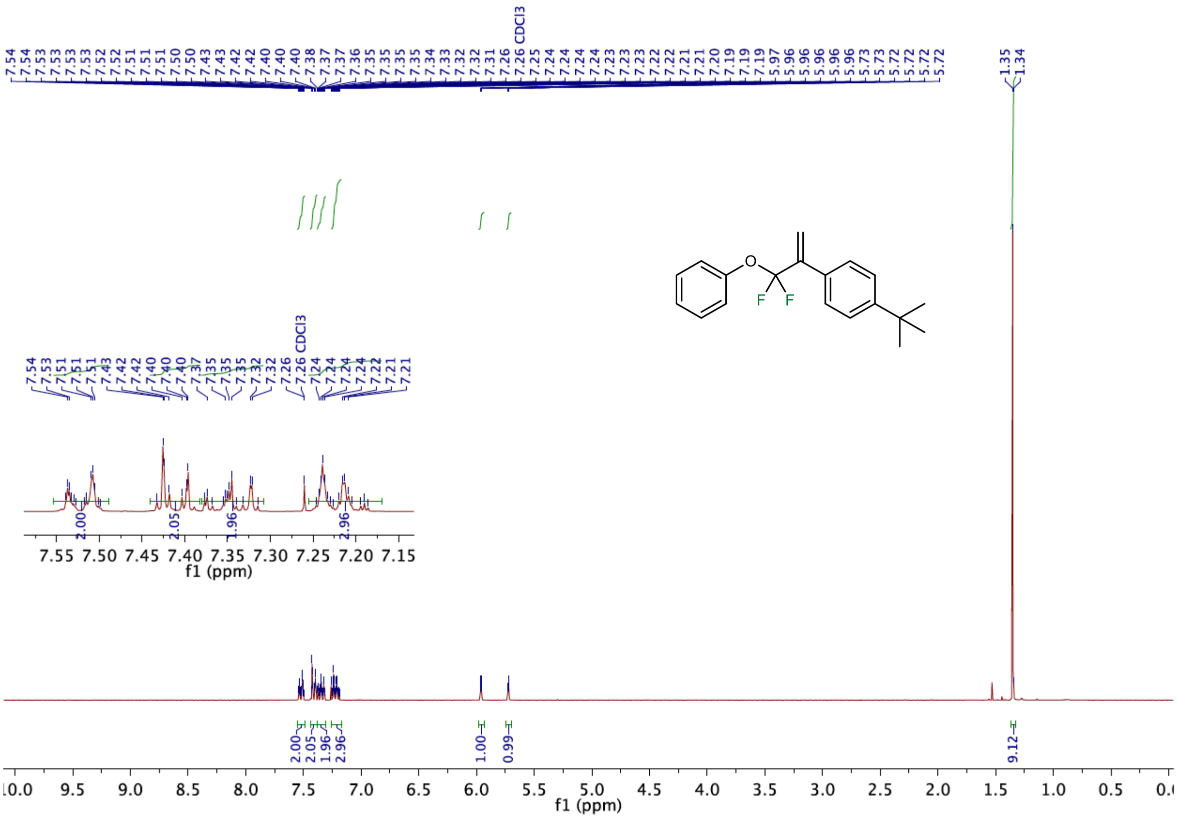

Supplementary Figure 209. ^{1}H NMR spectra of compound S1f

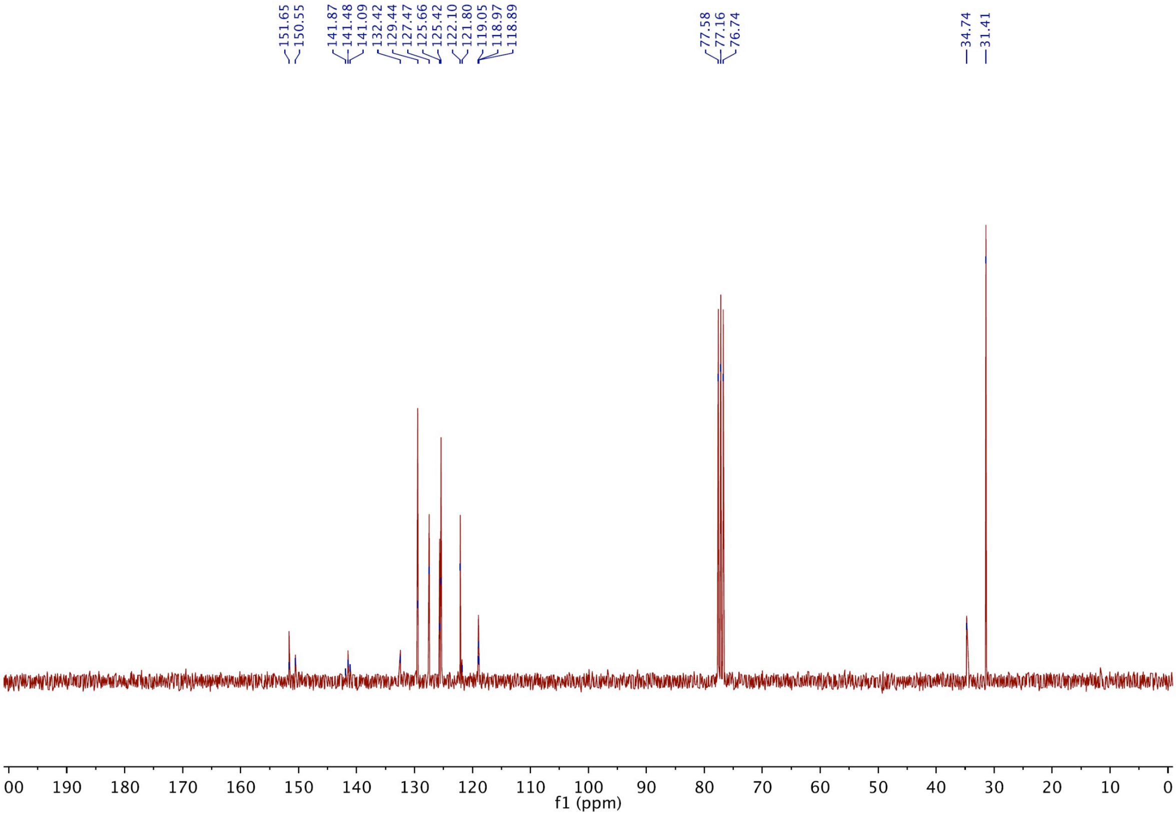

Supplementary Figure 210. ^{13}C NMR spectra of compound S1f

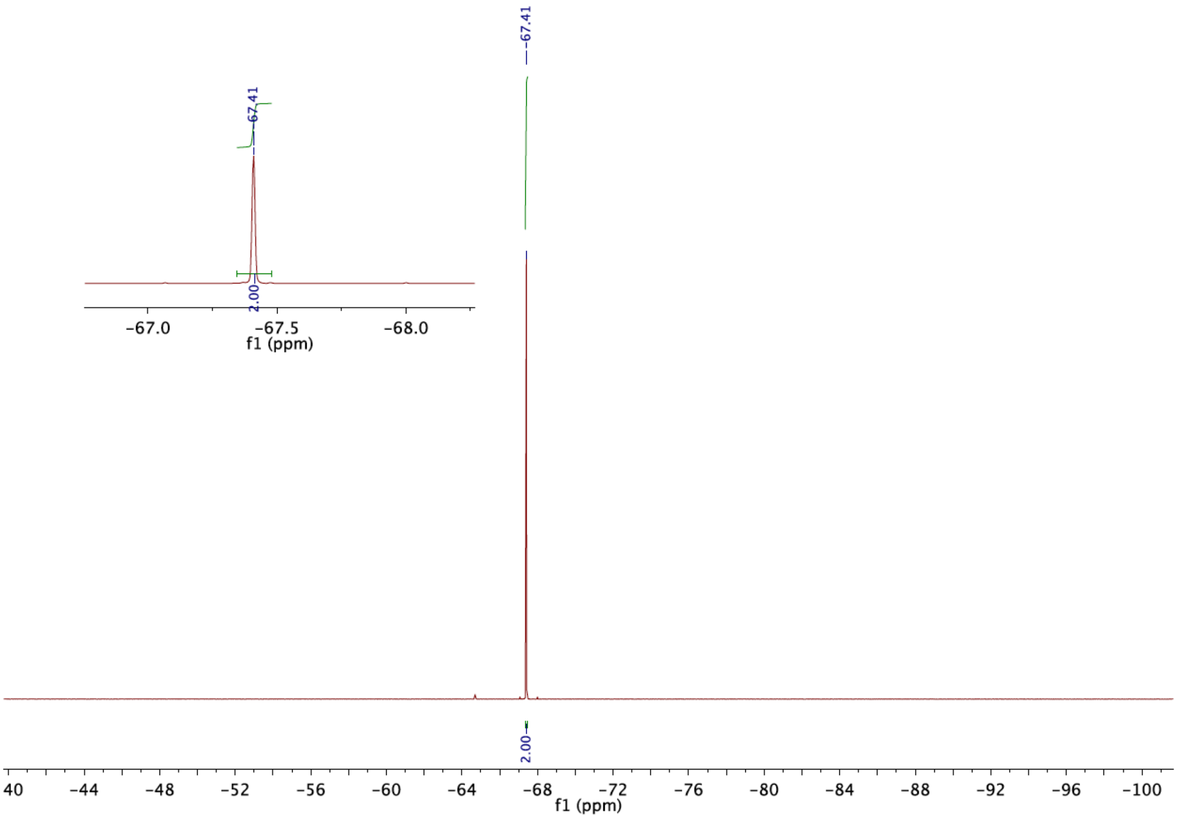

Supplementary Figure 211. ^{19}F NMR spectra of compound S1f

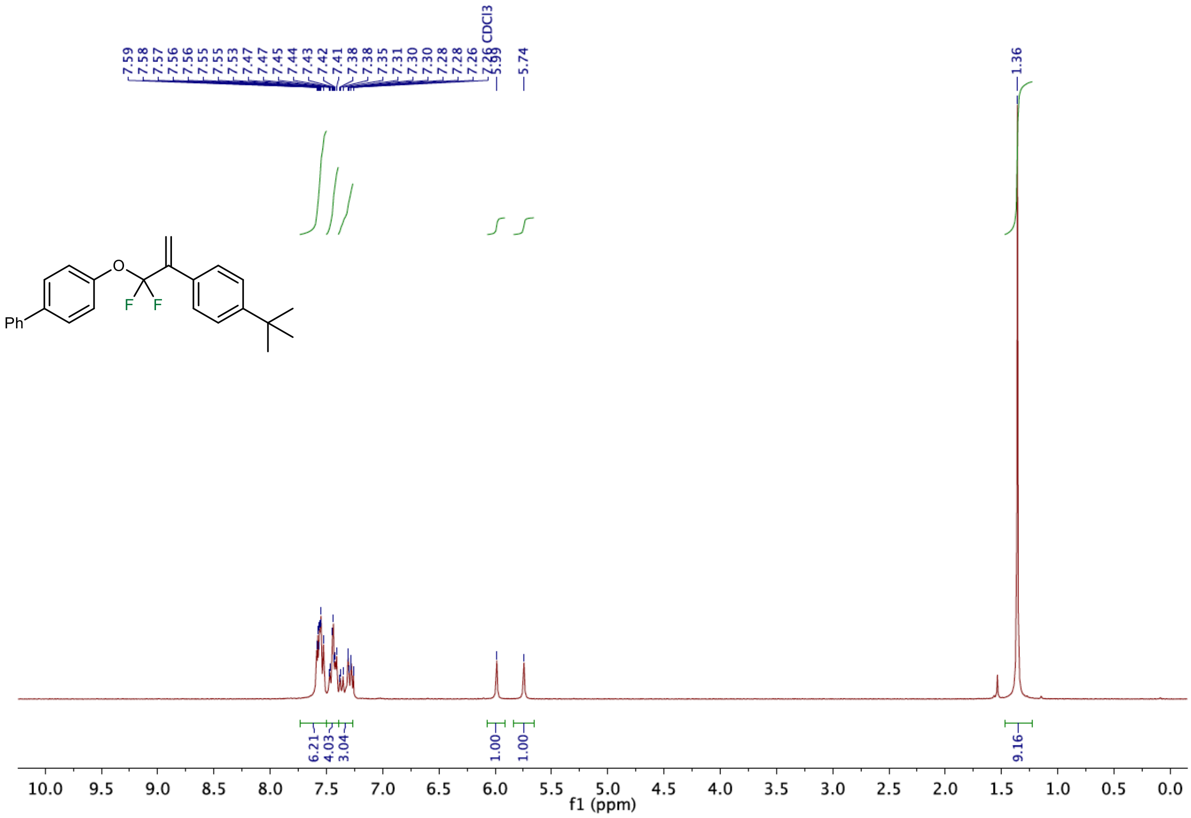

Supplementary Figure 212. ^{1}H NMR spectra of compound S1g

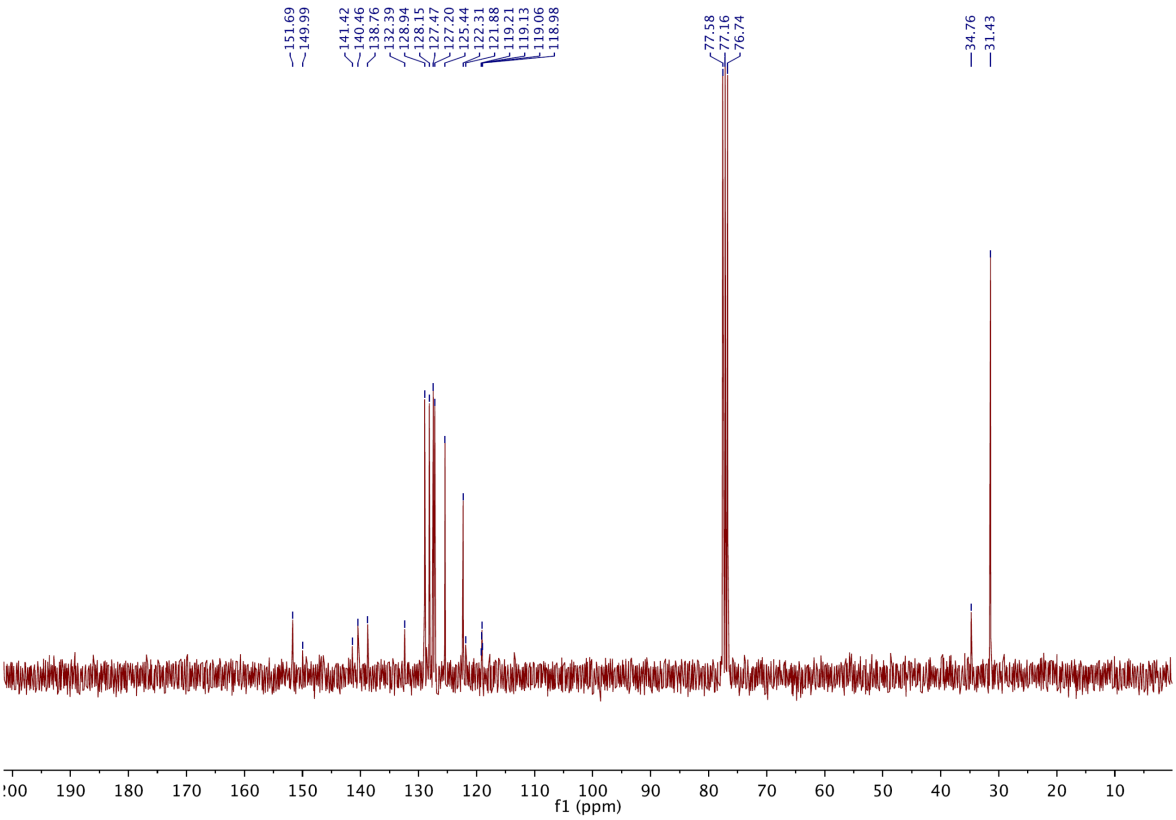

Supplementary Figure 213. ^{13}C NMR spectra of compound S1g

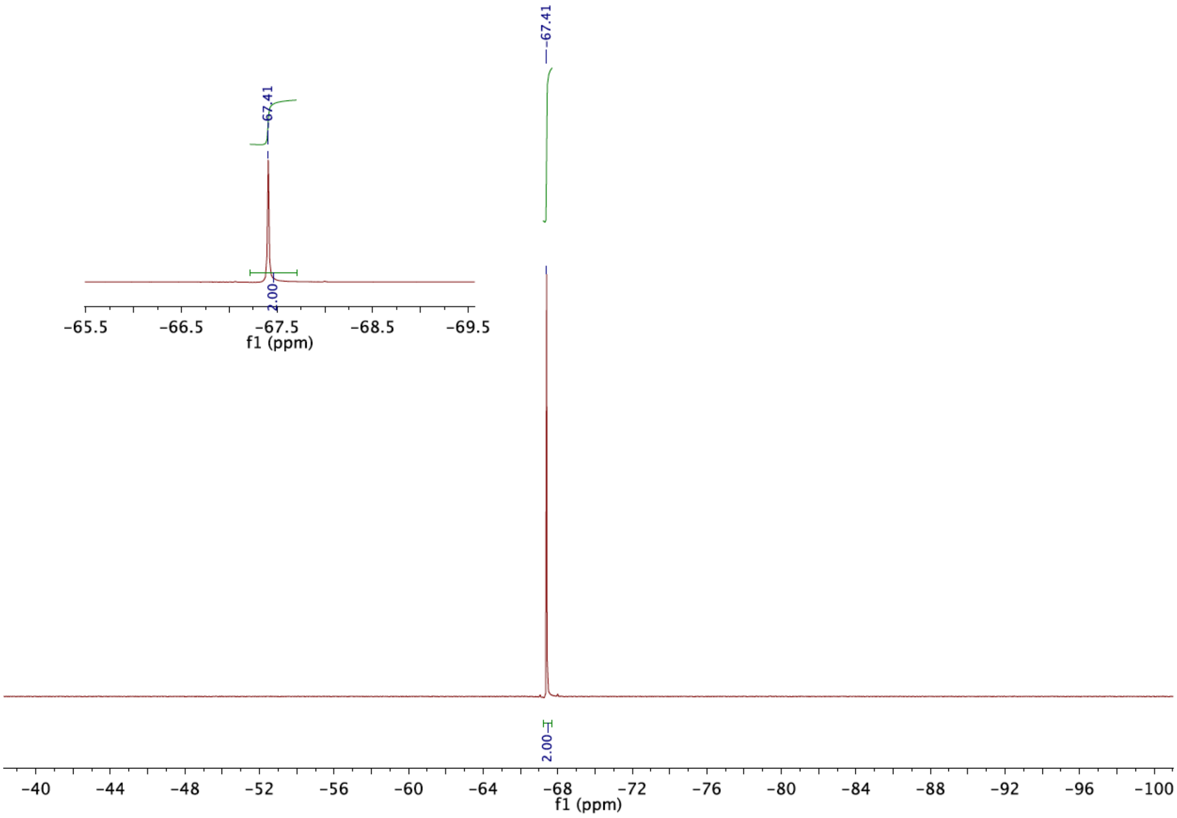

Supplementary Figure 214. ^{19}F NMR spectra of compound S1g

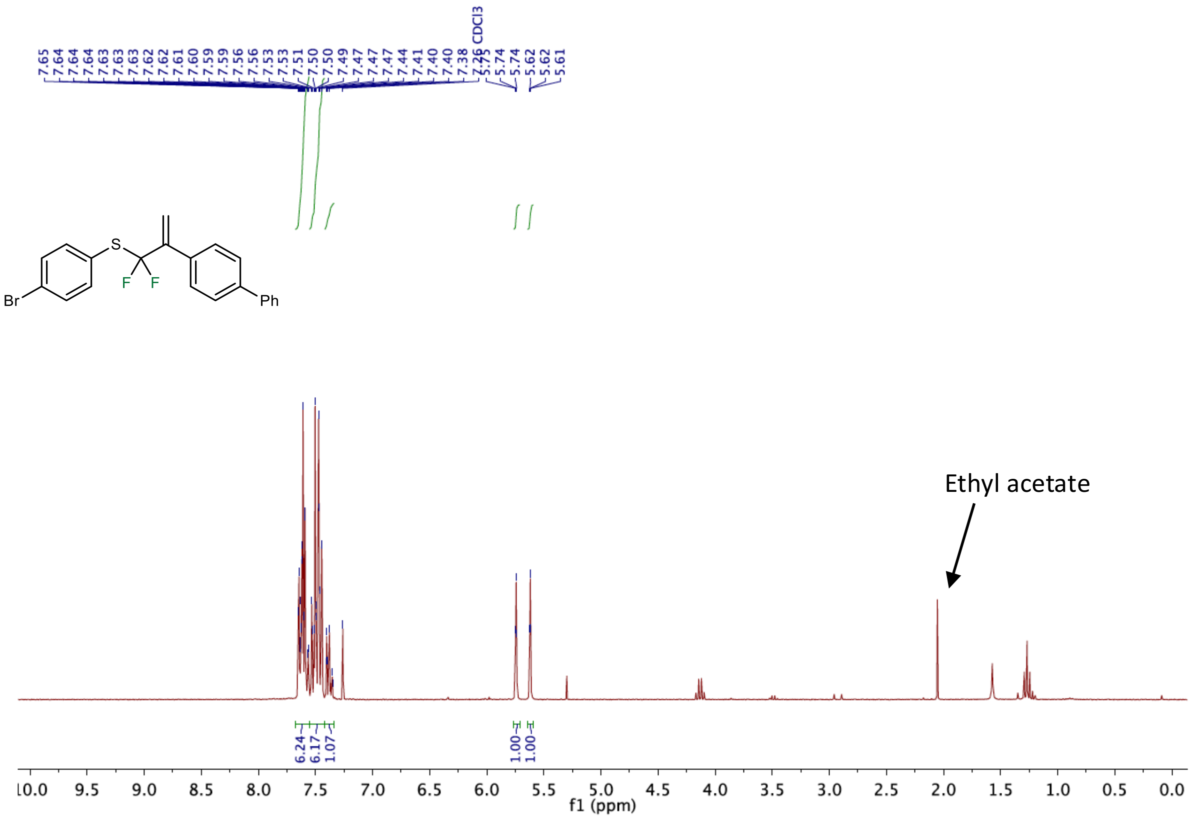

Supplementary Figure 215. ^{1}H NMR spectra of compound S1h

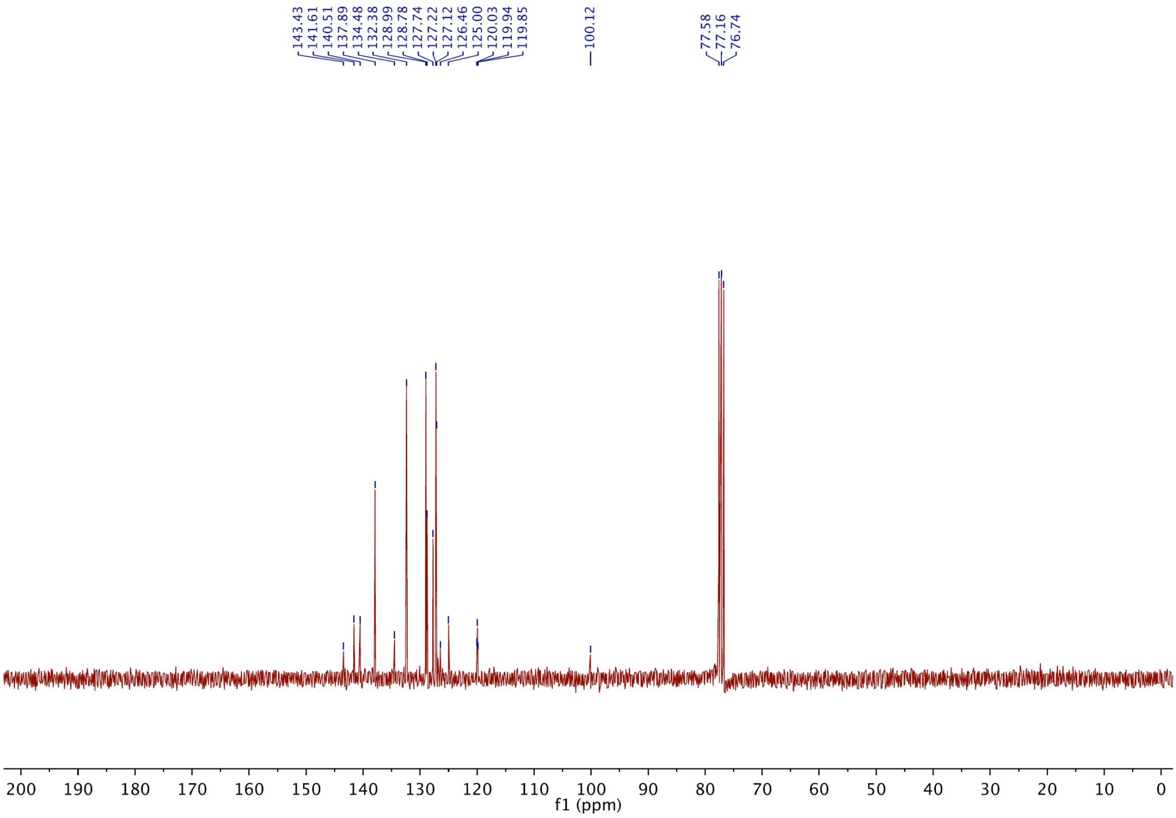

Supplementary Figure 216. ^{13}C NMR spectra of compound S1h

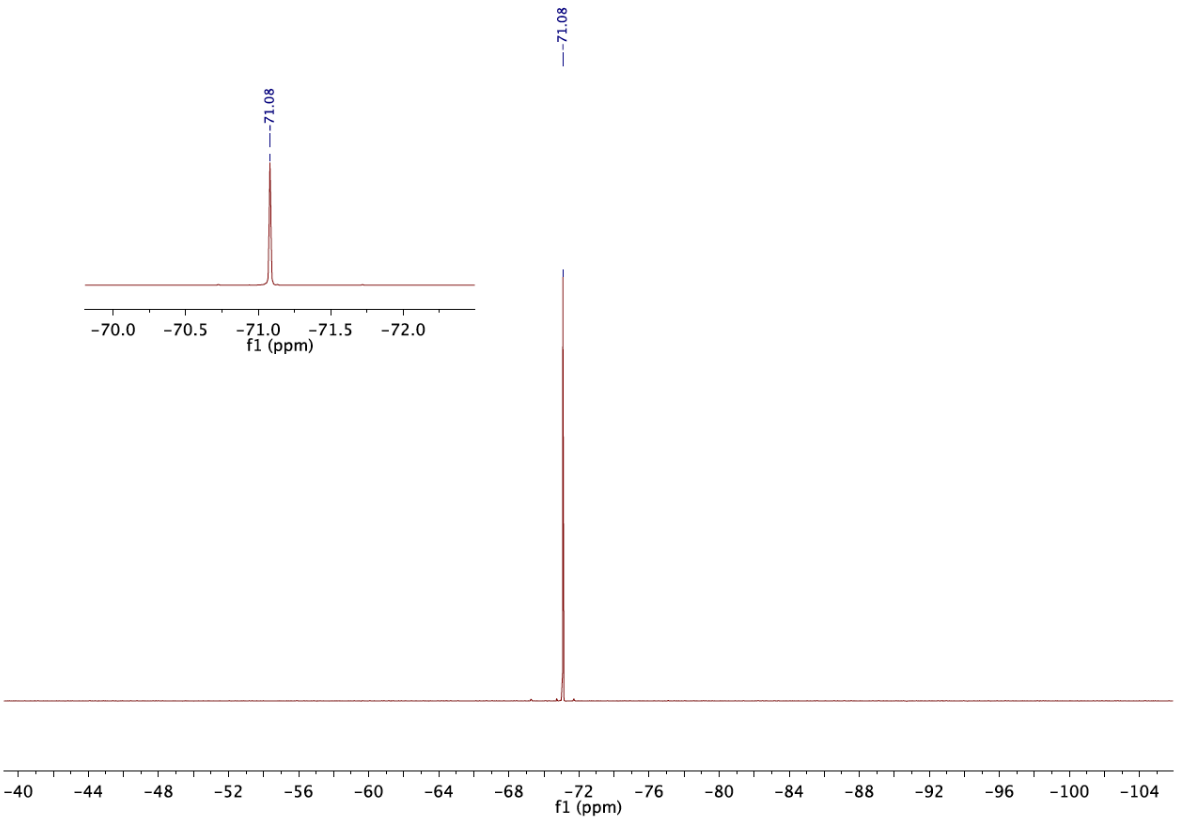

Supplementary Figure 217. ^{19}F NMR spectra of compound S1h

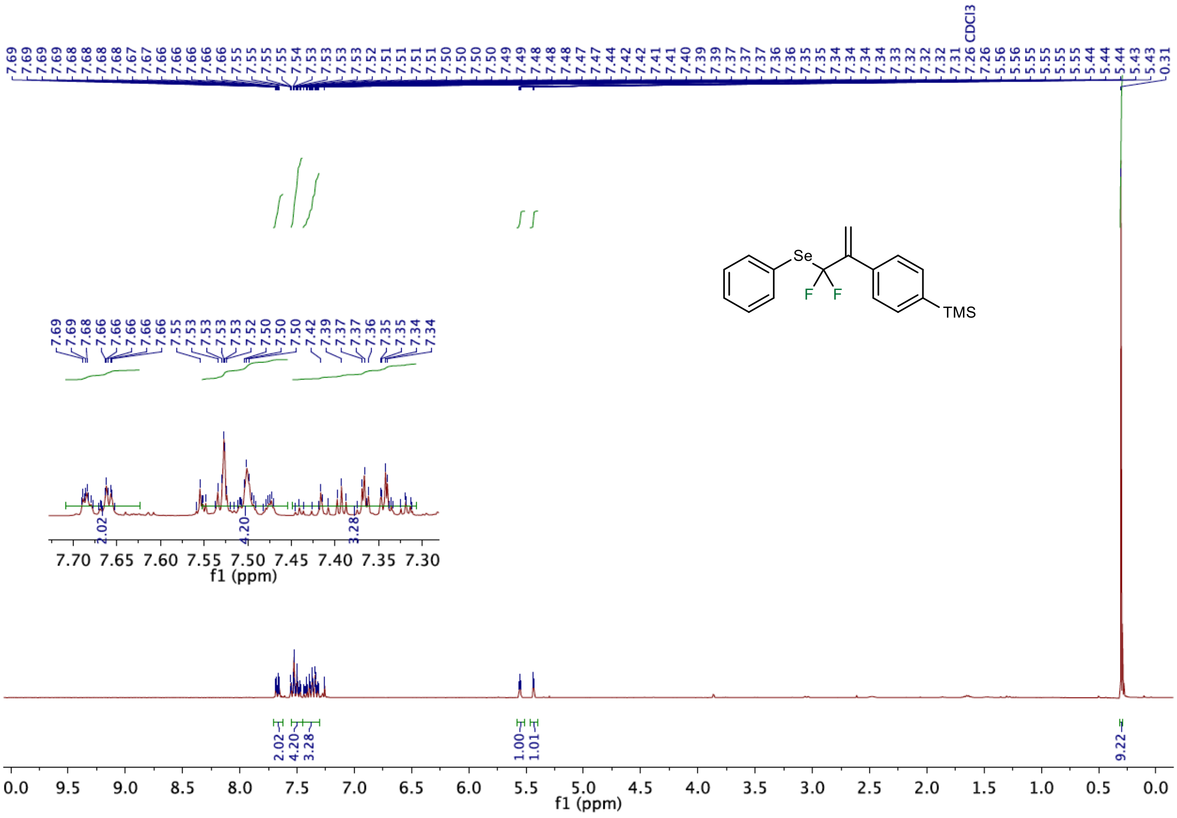

Supplementary Figure 218. ^{1}H NMR spectra of compound S1i

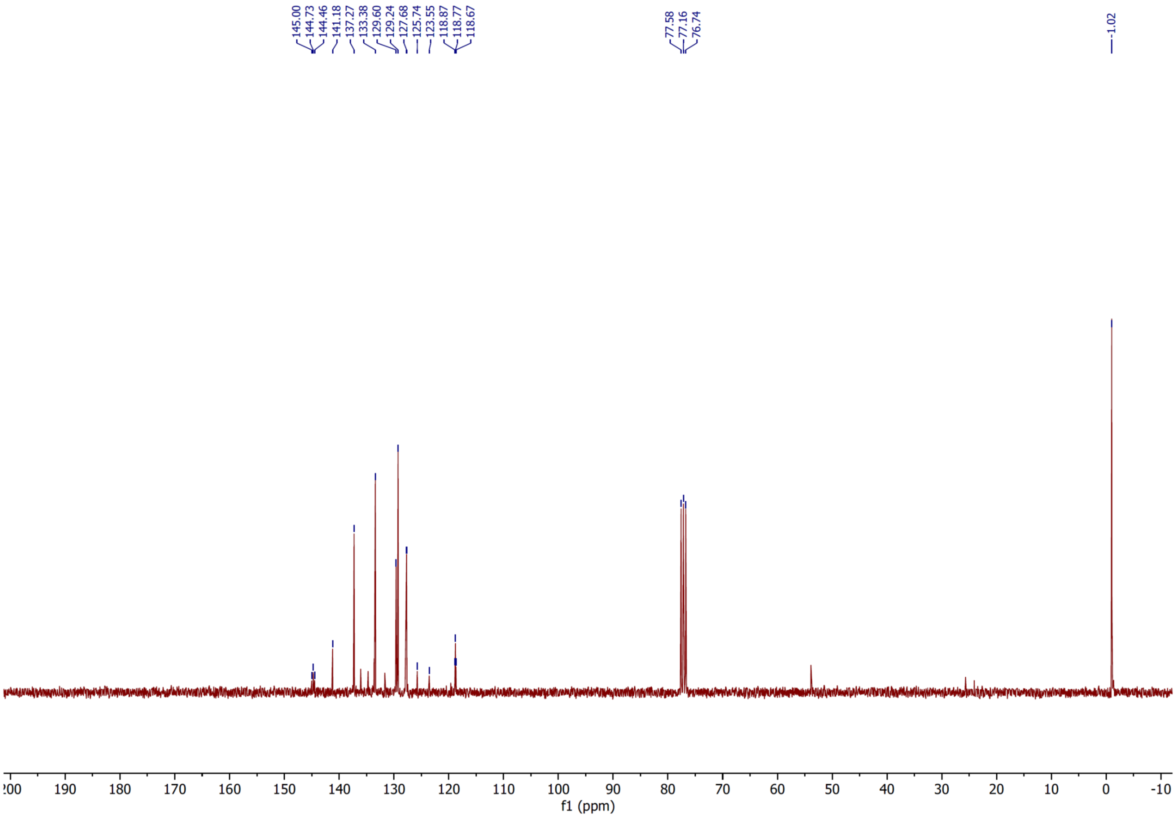

Supplementary Figure 219. ^{13}C **NMR spectra of compound S1i**

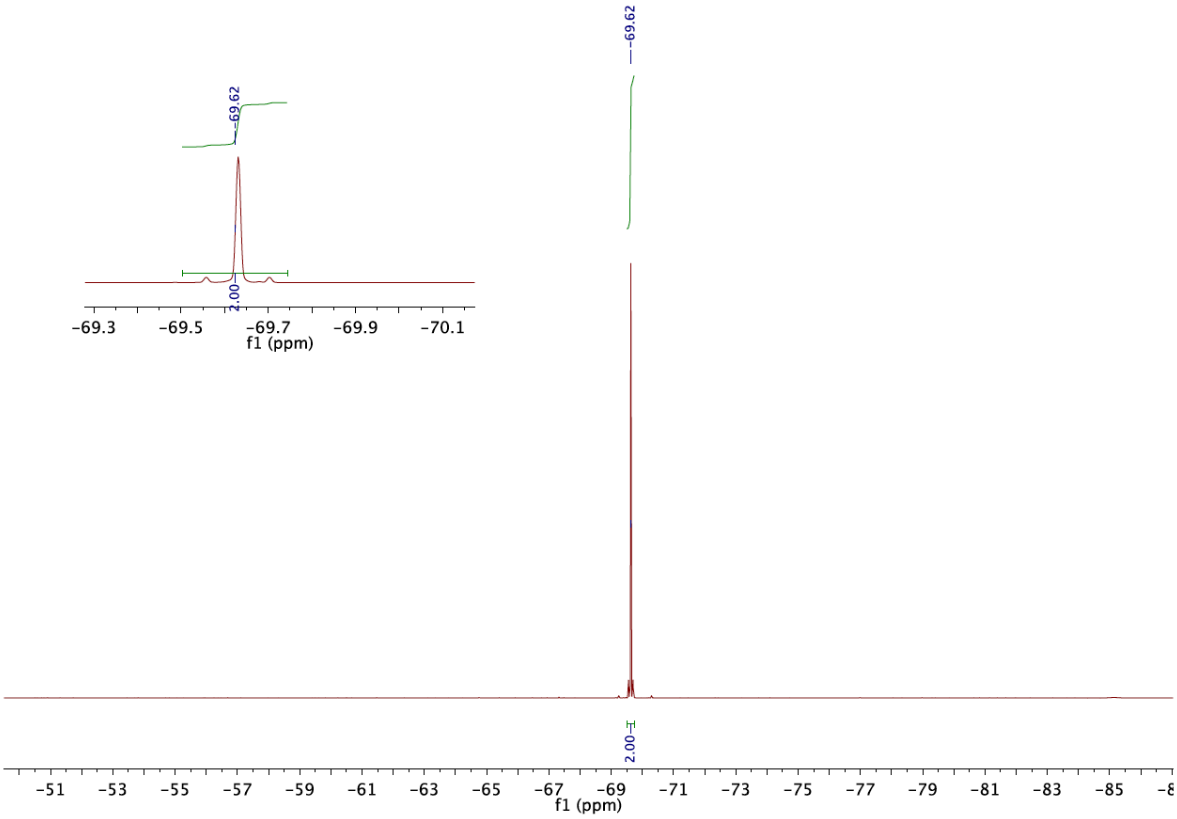

Supplementary Figure 220. ^{19}F **NMR spectra of compound S1i**

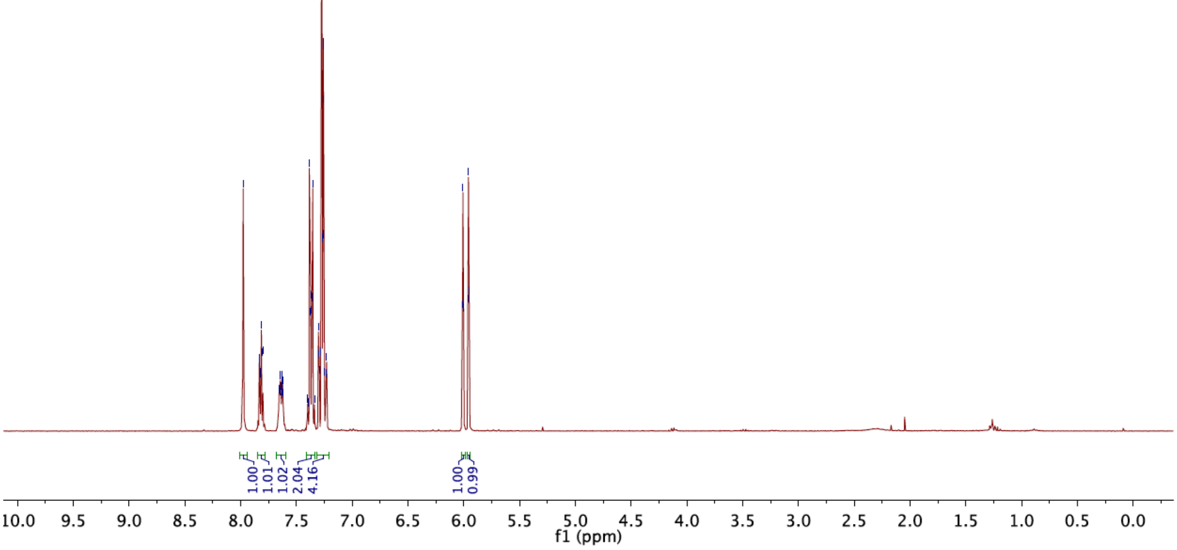

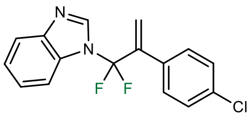

Supplementary Figure 221. ^{1}H NMR spectra of compound S1j

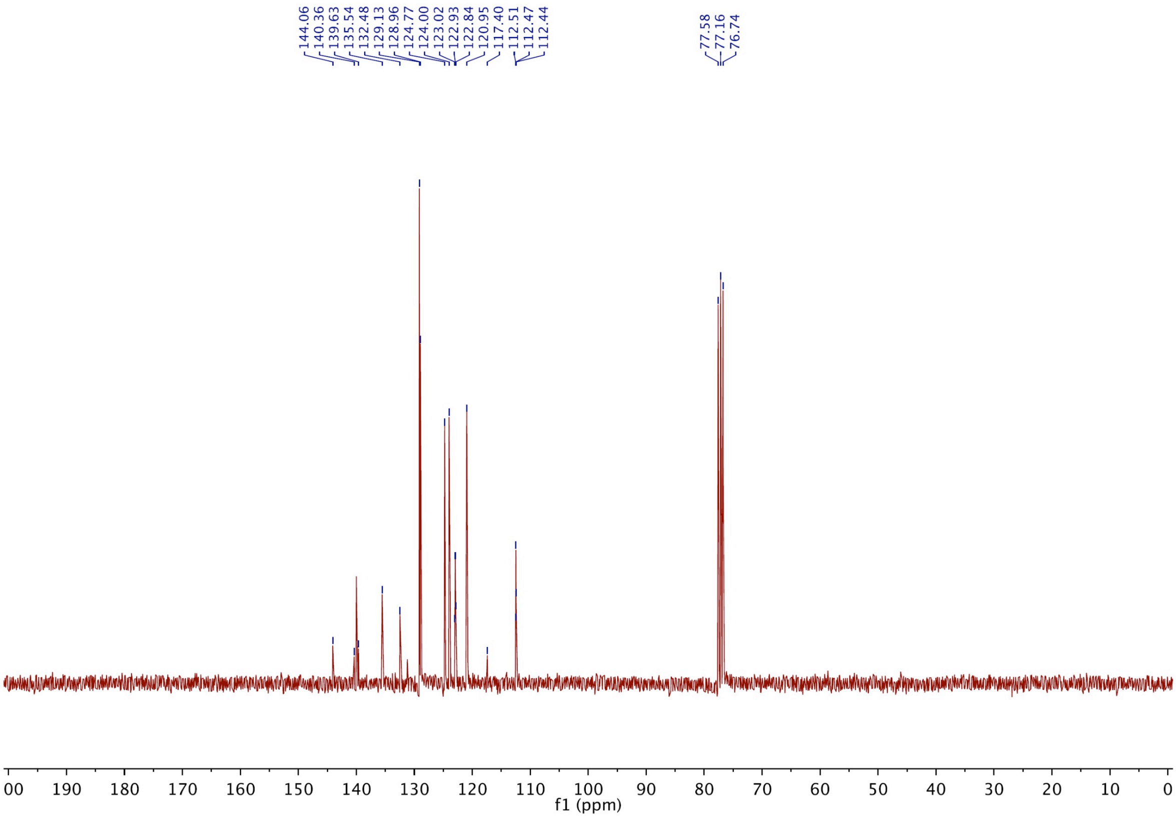

Supplementary Figure 222. ^{13}C NMR spectra of compound S1j

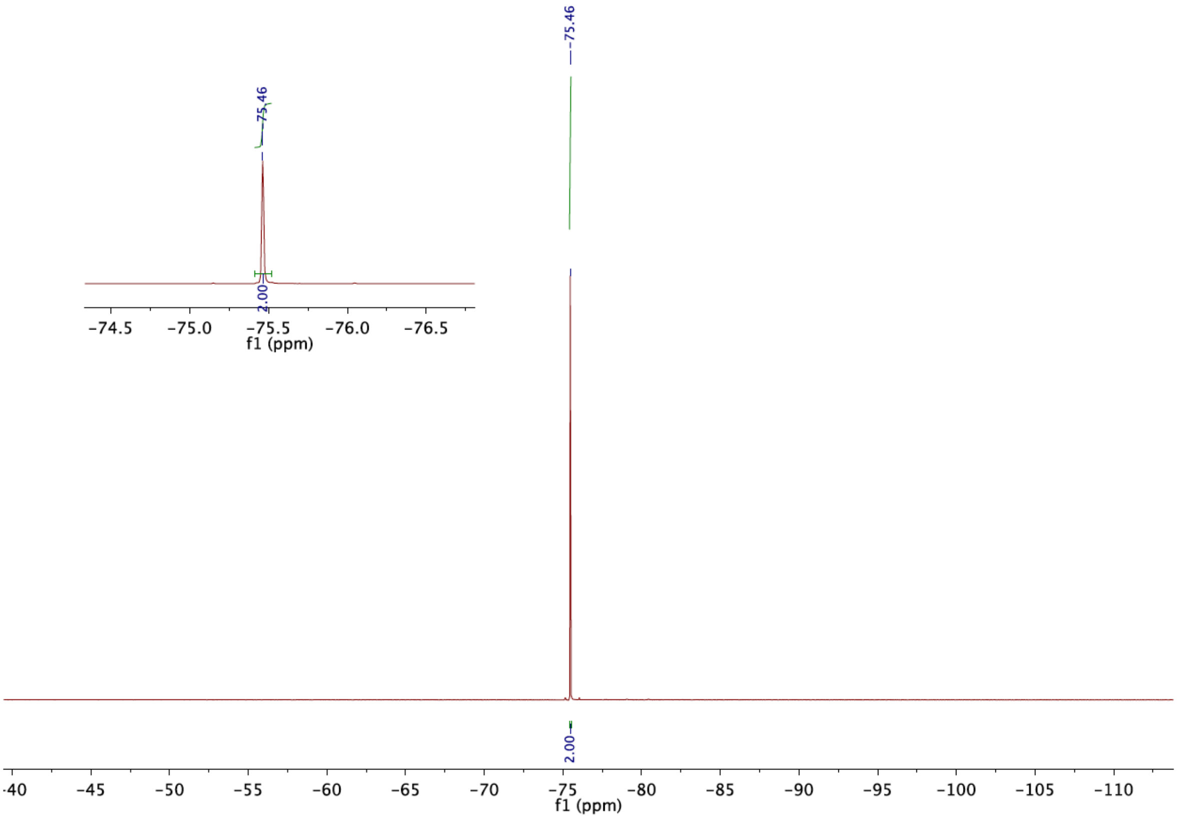

Supplementary Figure 223. ^{19}F NMR spectra of compound S1j

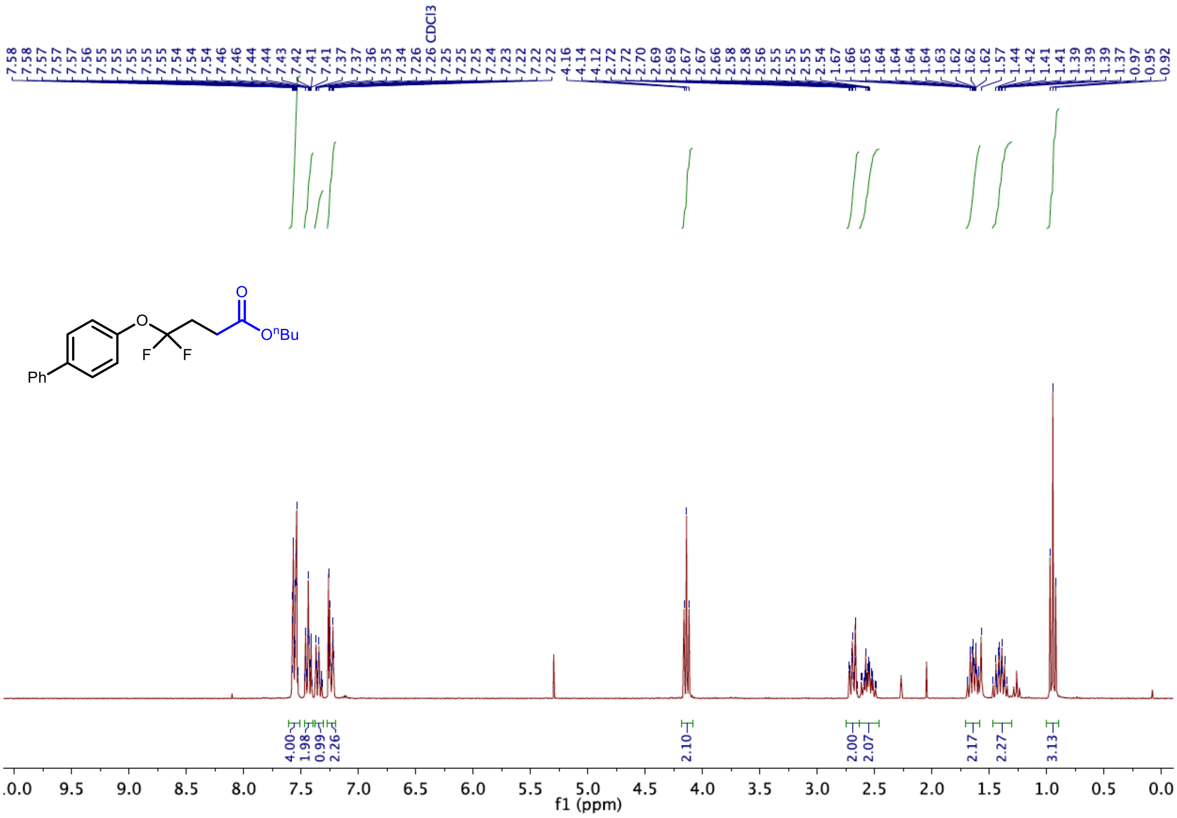

Supplementary Figure 224. ^{1}H NMR spectra of compound 61

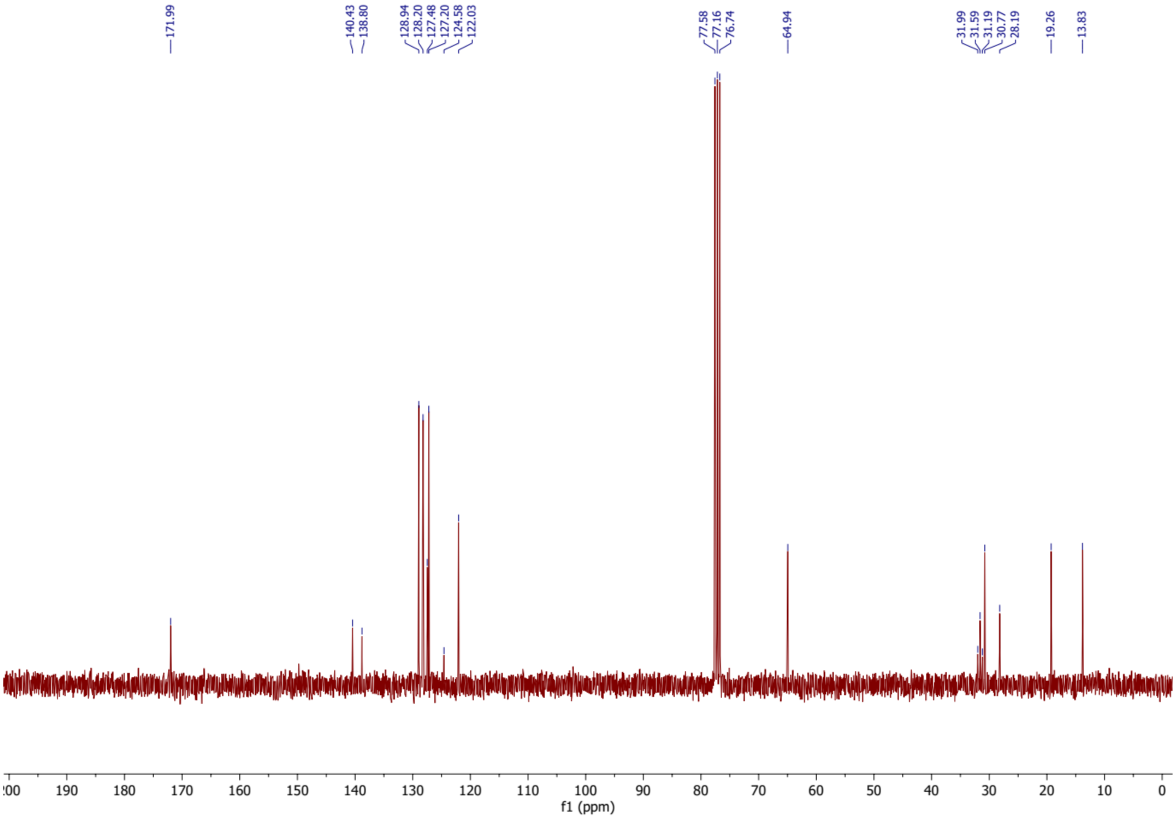

Supplementary Figure 225. ^{13}C NMR spectra of compound 61

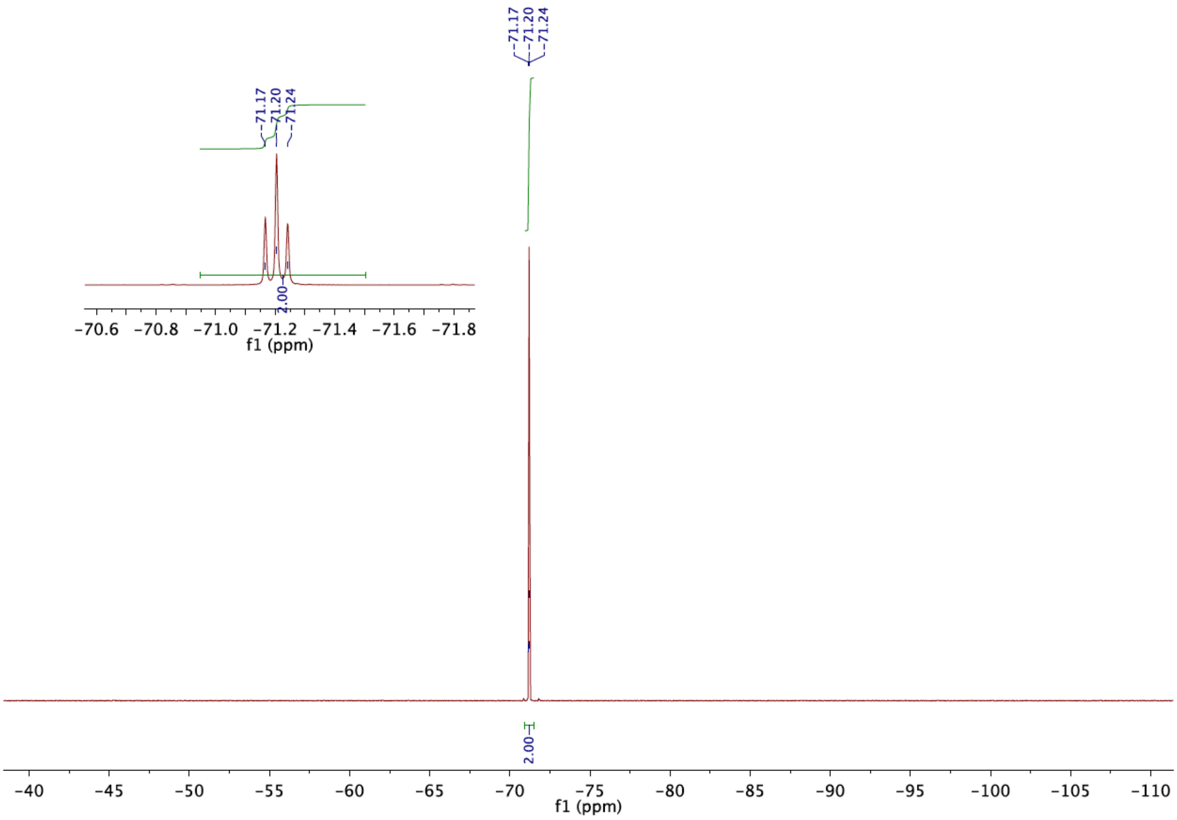

Supplementary Figure 226. ^{19}F NMR spectra of compound 61

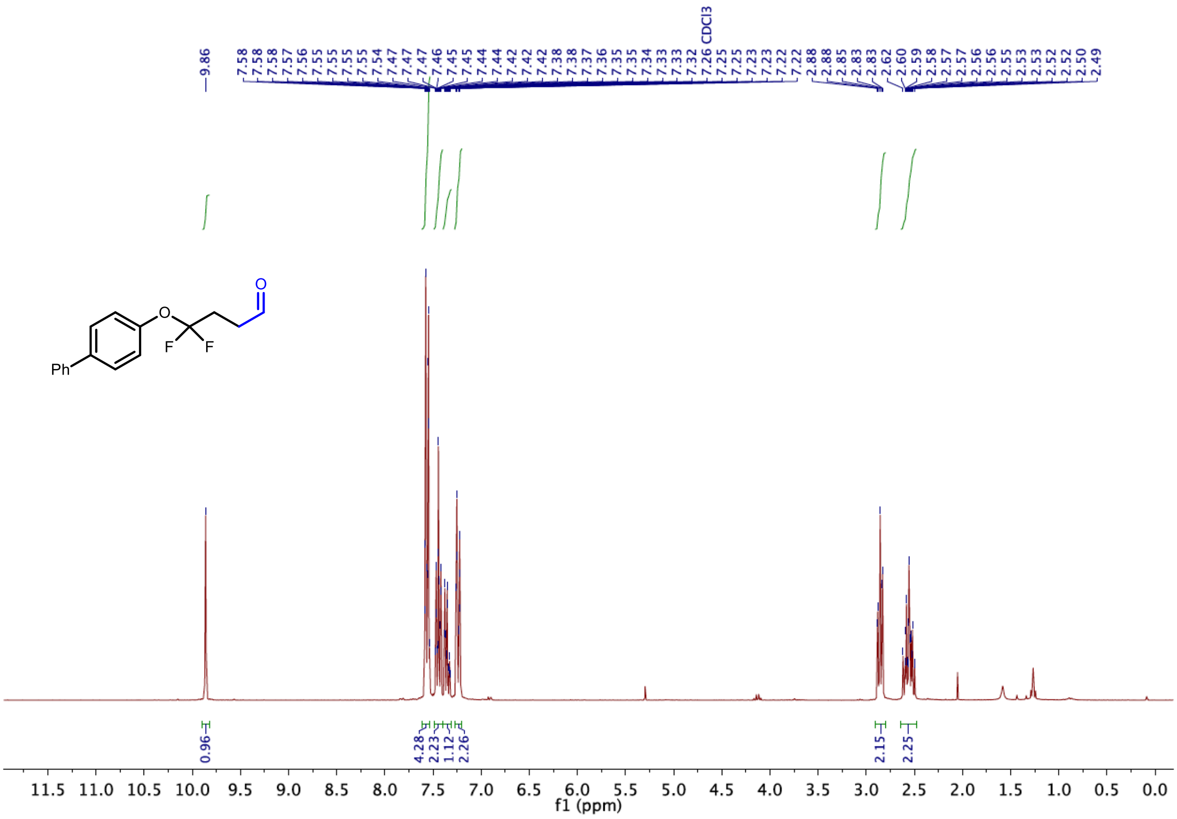

Supplementary Figure 227. ^{1}**H NMR spectra of compound 62**

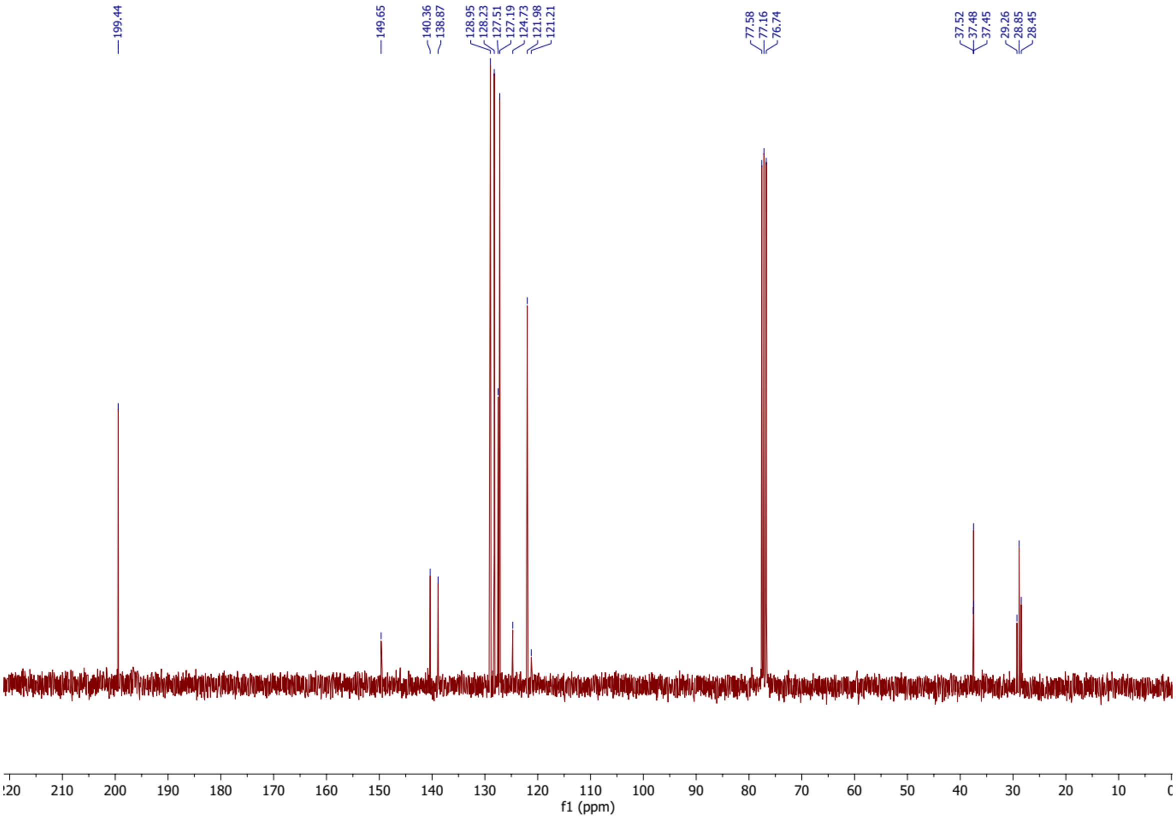

Supplementary Figure 228. ^{13}**C NMR spectra of compound 62**

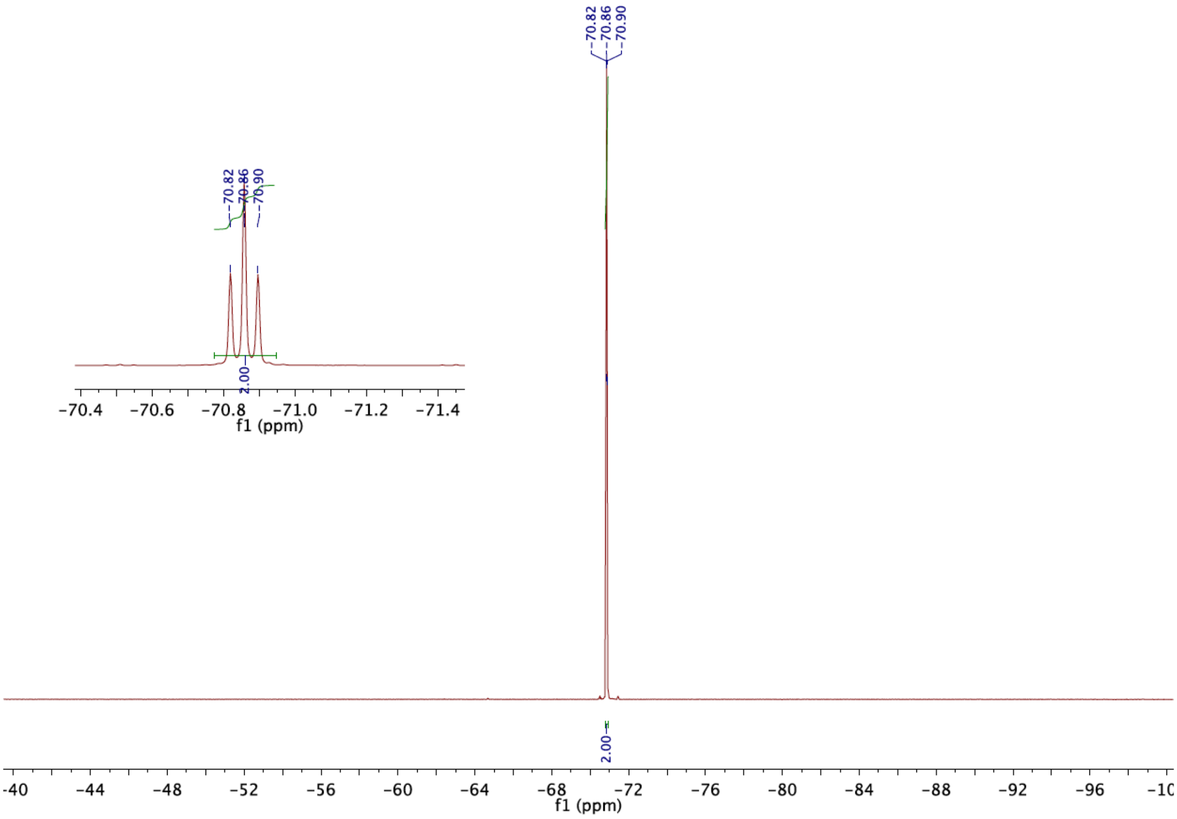

Supplementary Figure 229. ^{19}F NMR spectra of compound 62

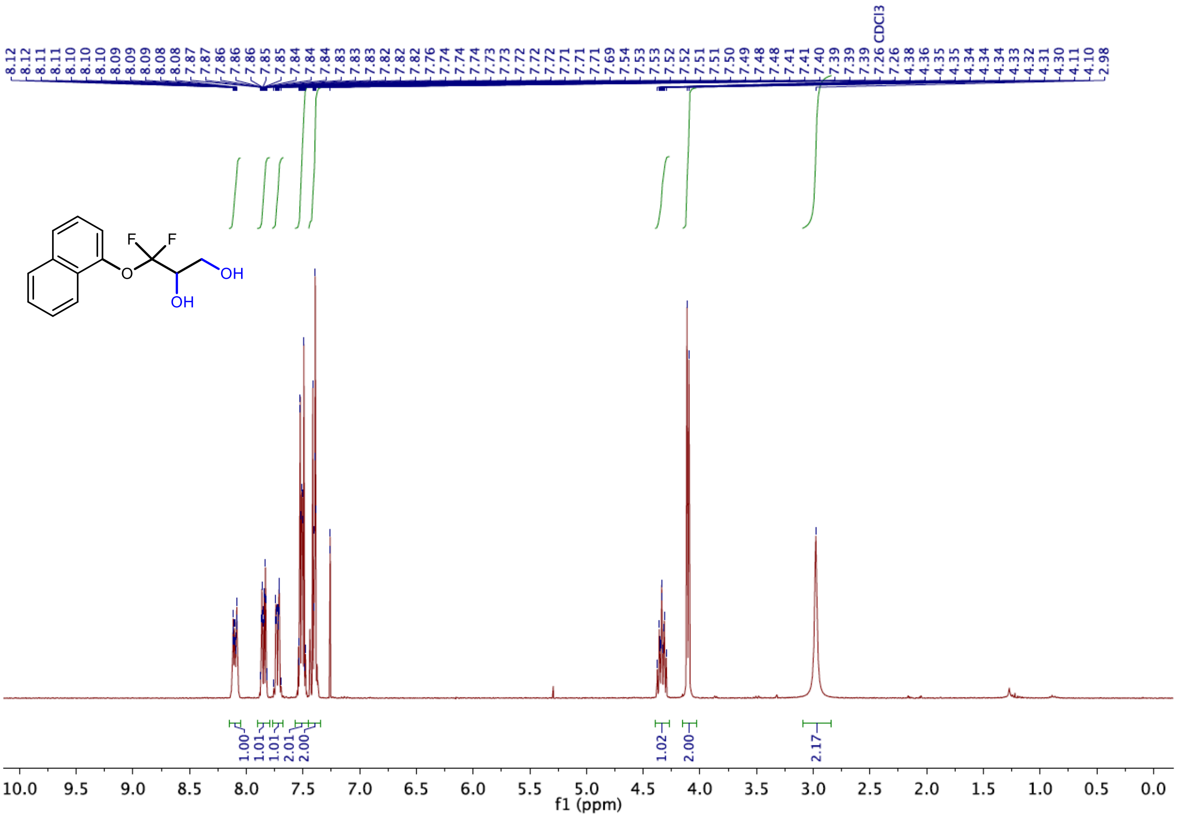

Supplementary Figure 230. ^{1}H NMR spectra of compound 63

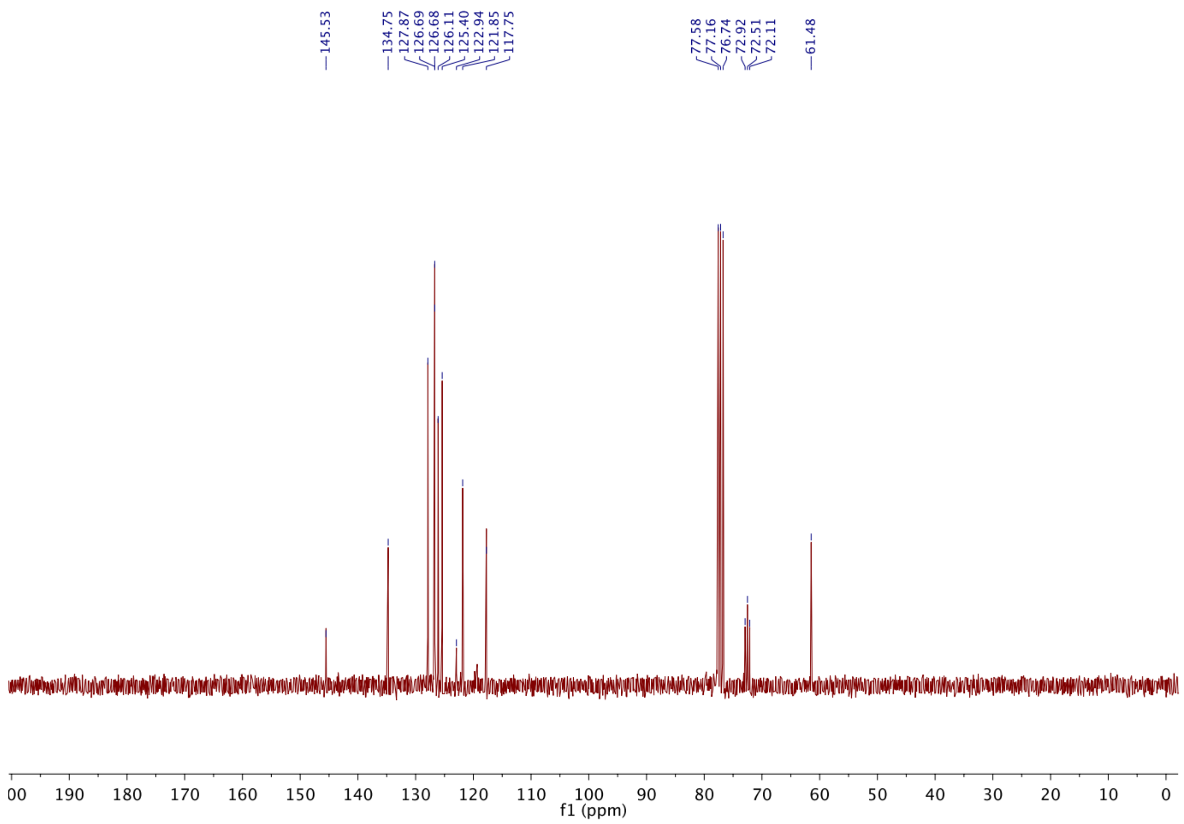

Supplementary Figure 231. ^{13}C NMR spectra of compound 63

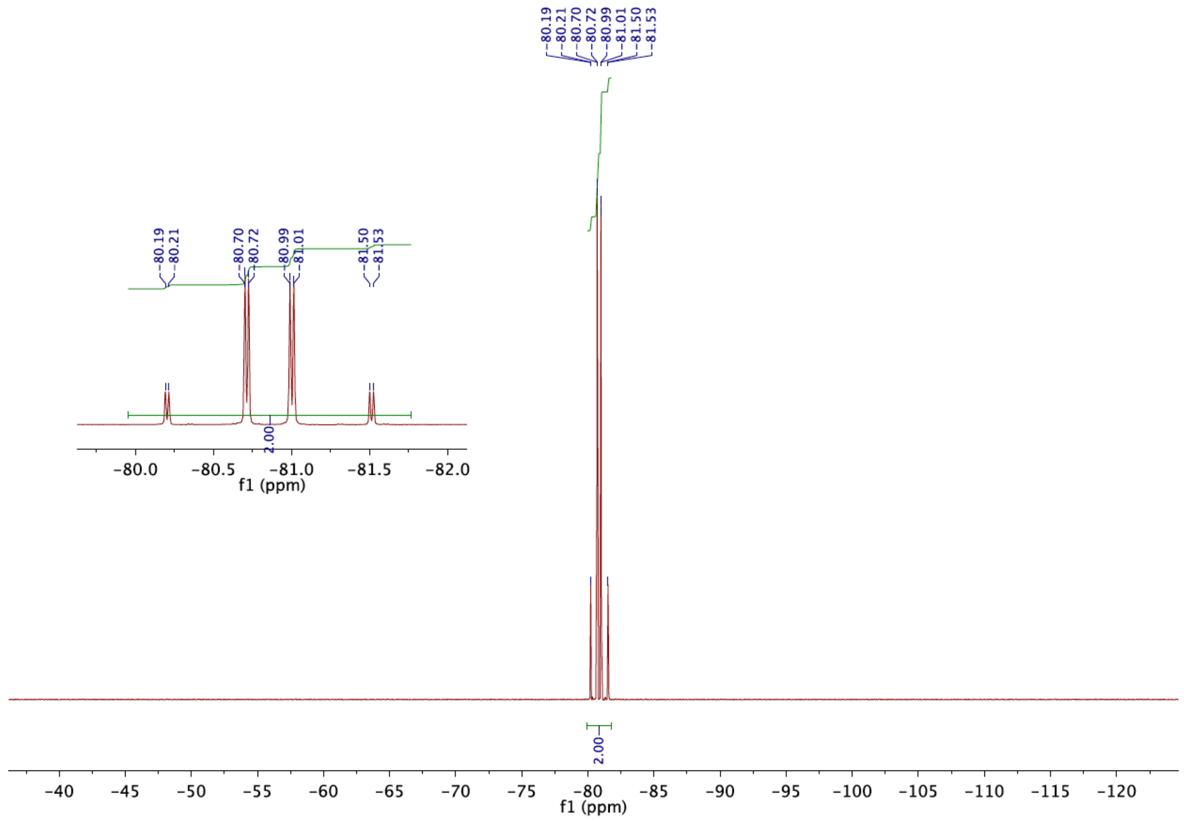

Supplementary Figure 232. ^{19}F NMR spectra of compound 63

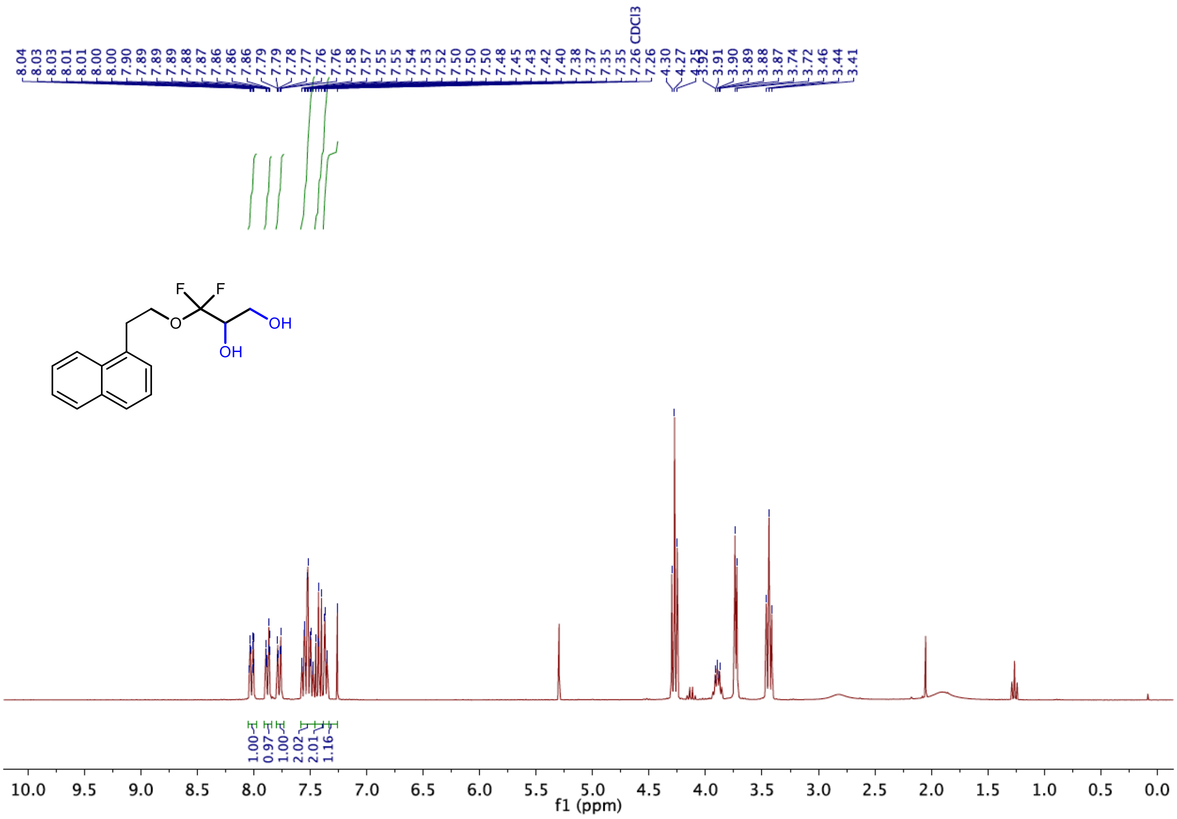

Supplementary Figure 233. ^{1}H NMR spectra of compound 64

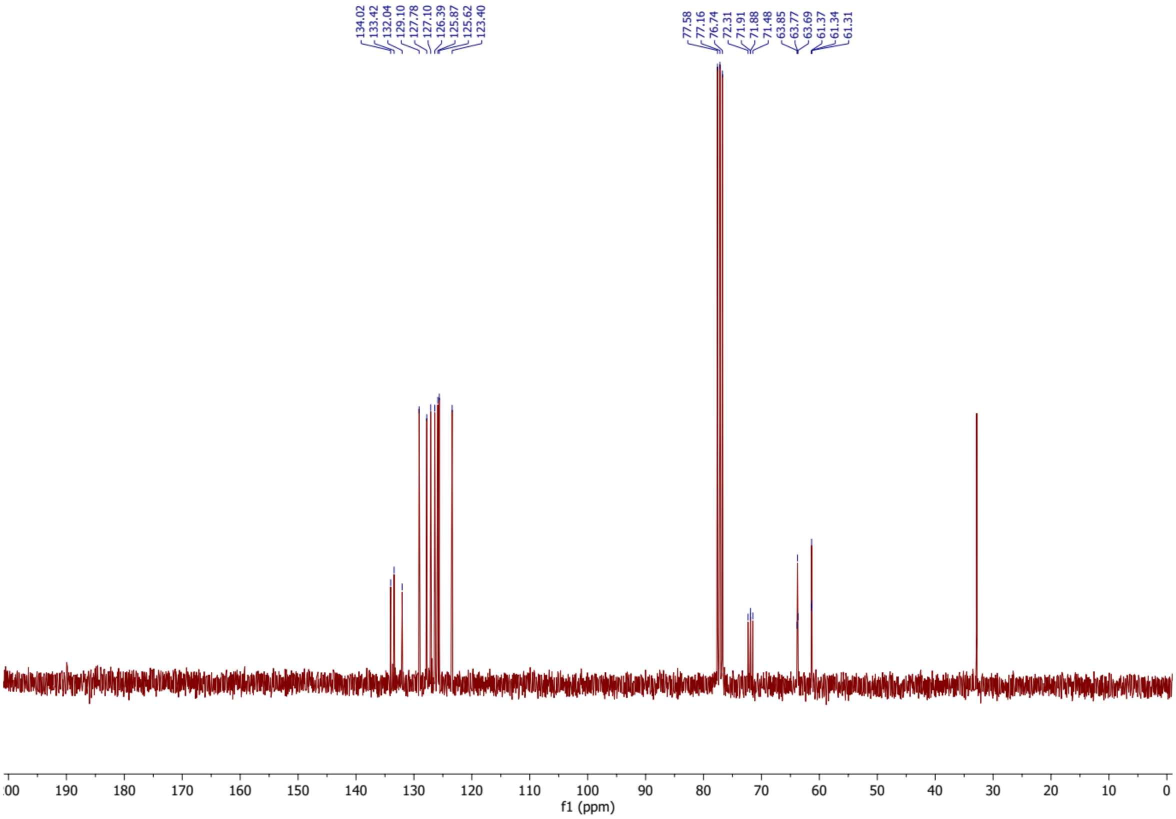

Supplementary Figure 234. ^{13}C NMR spectra of compound 64

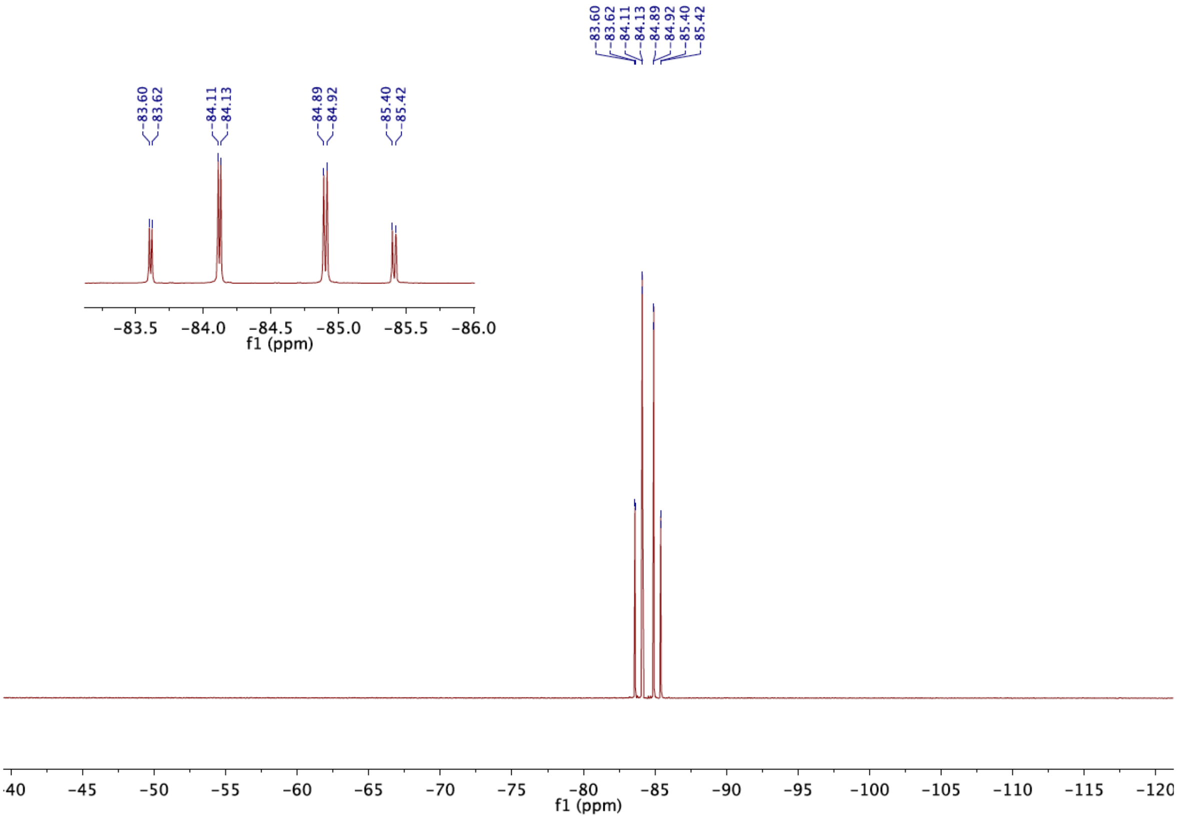

Supplementary Figure 235. ^{19}F NMR spectra of compound 64

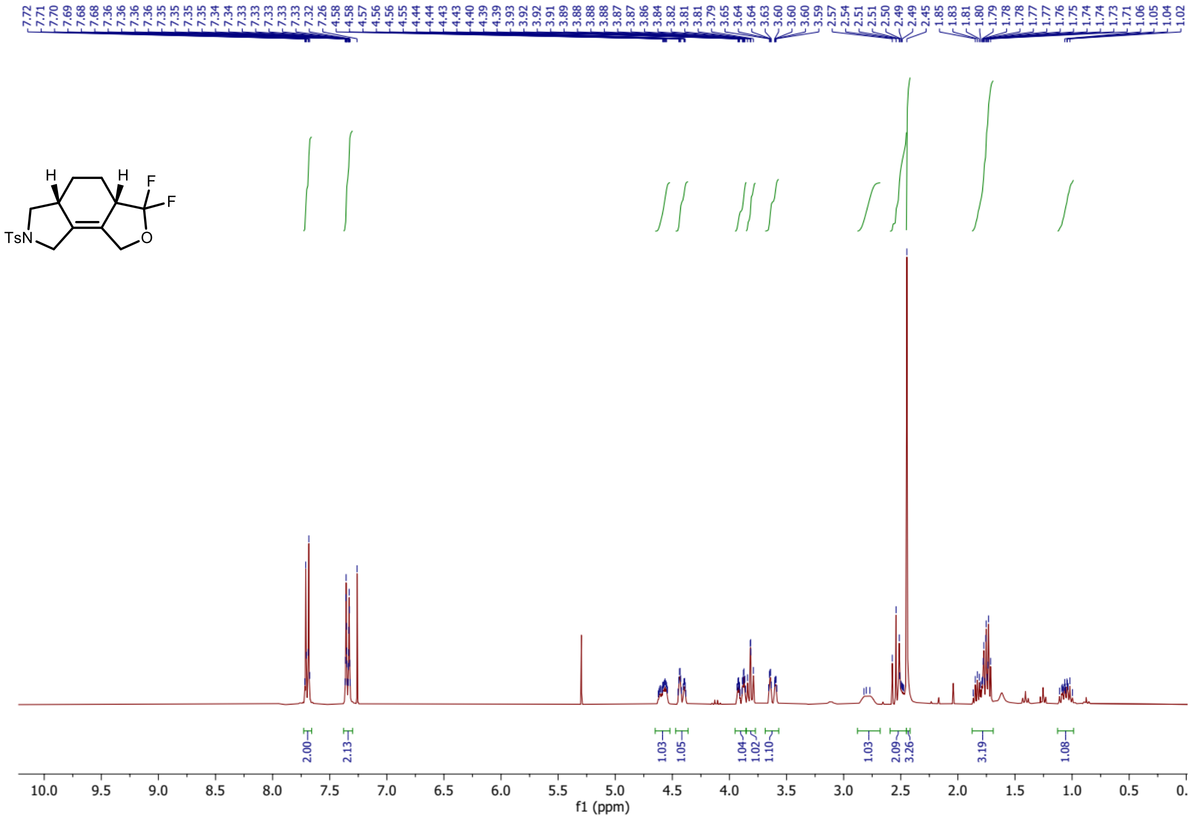

Supplementary Figure 236. ^{1}H NMR spectra of compound 65

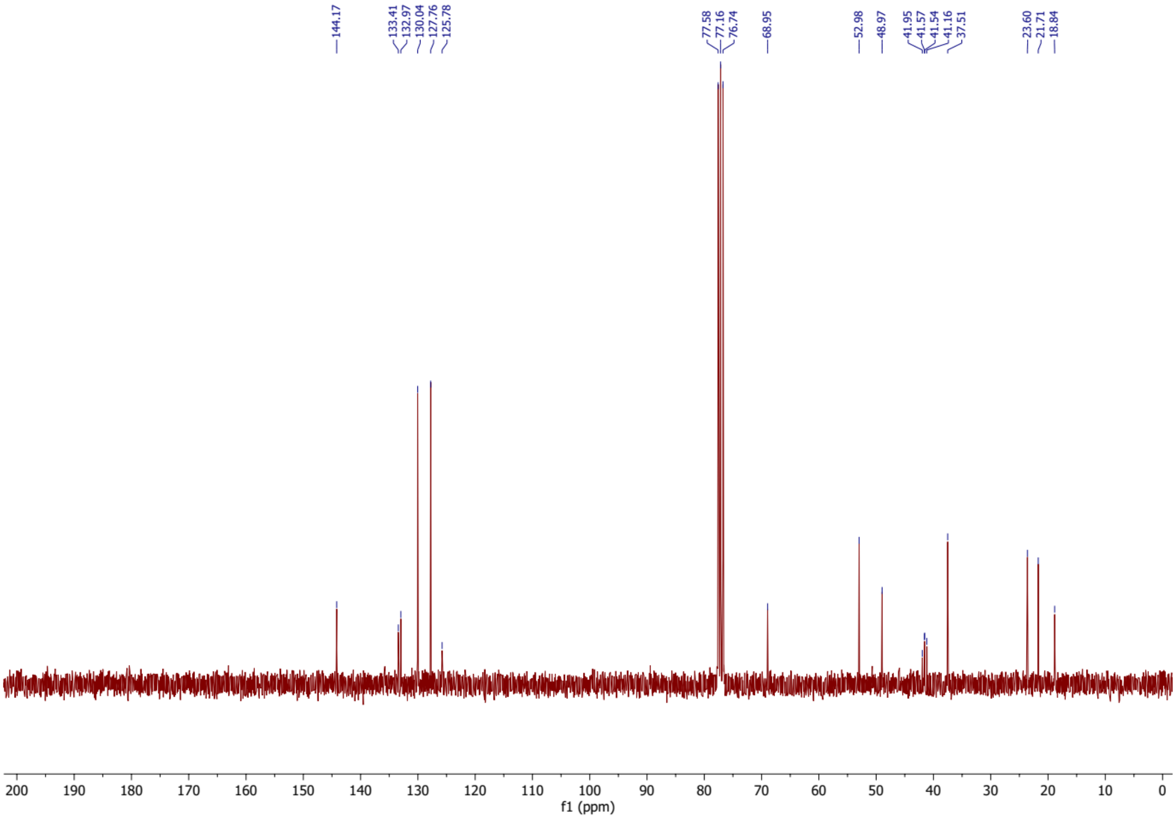

Supplementary Figure 237. ^{13}C NMR spectra of compound 65

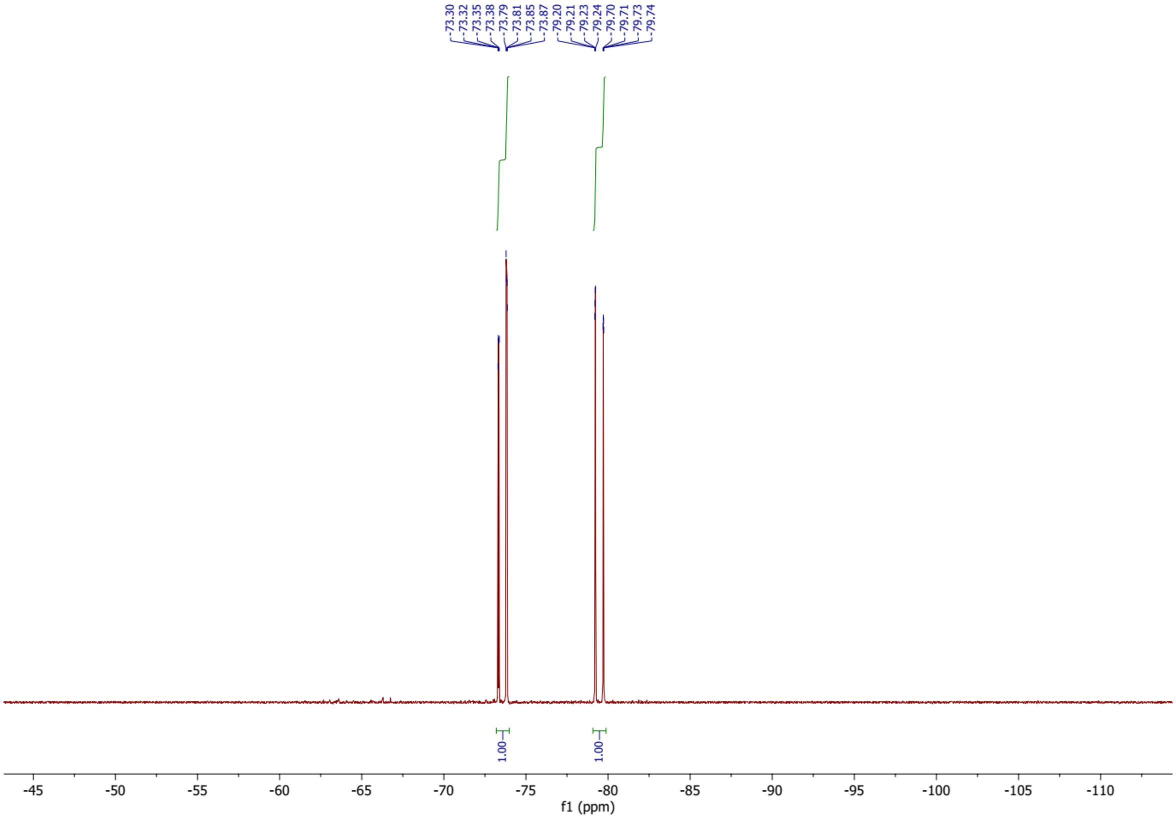

Supplementary Figure 238. ^{19}F NMR spectra of compound 65

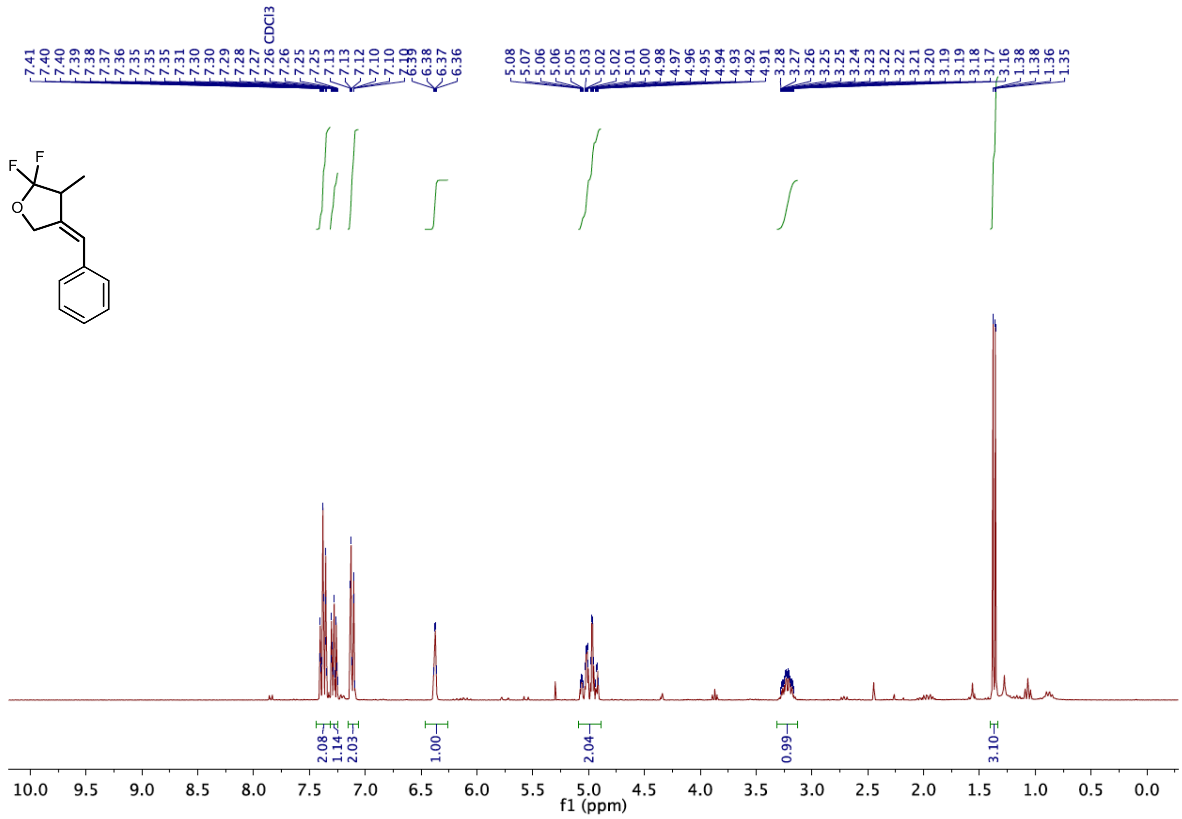

Supplementary Figure 239. ^{1}H NMR spectra of compound 66

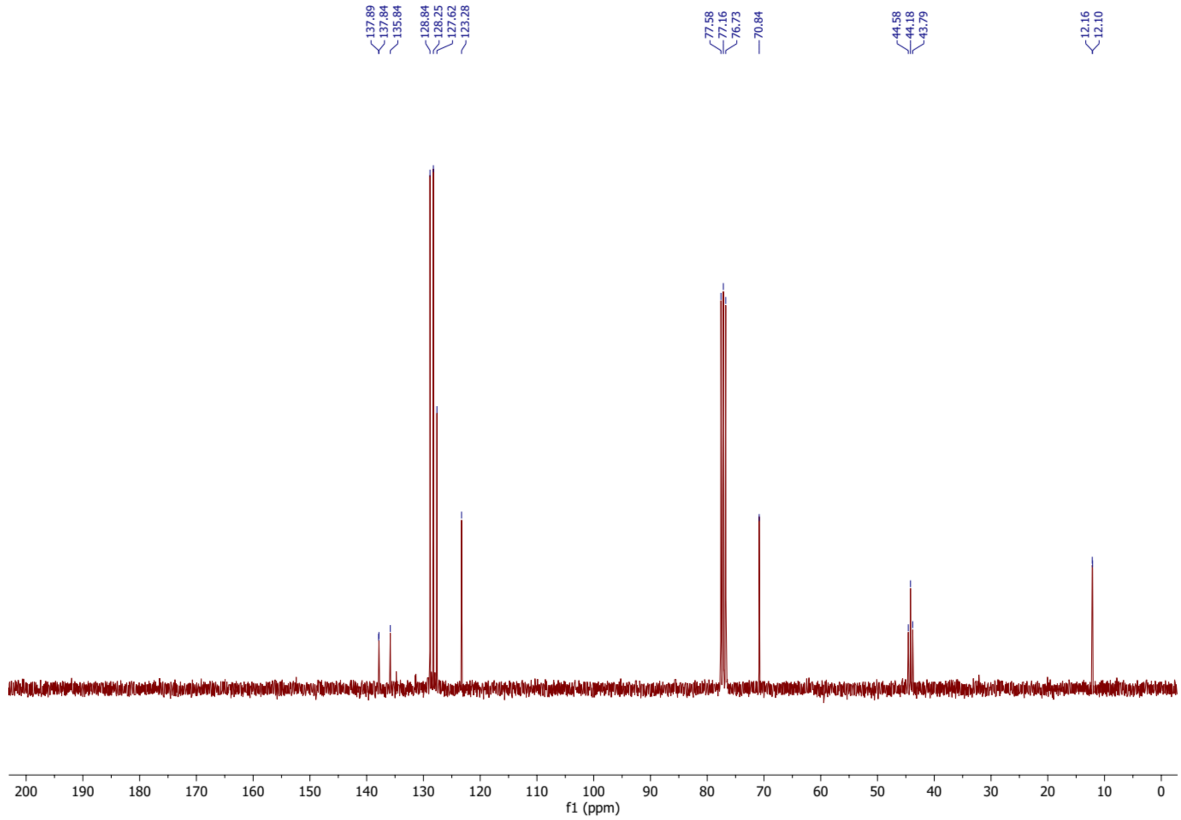

Supplementary Figure 240. ^{13}C NMR spectra of compound 66

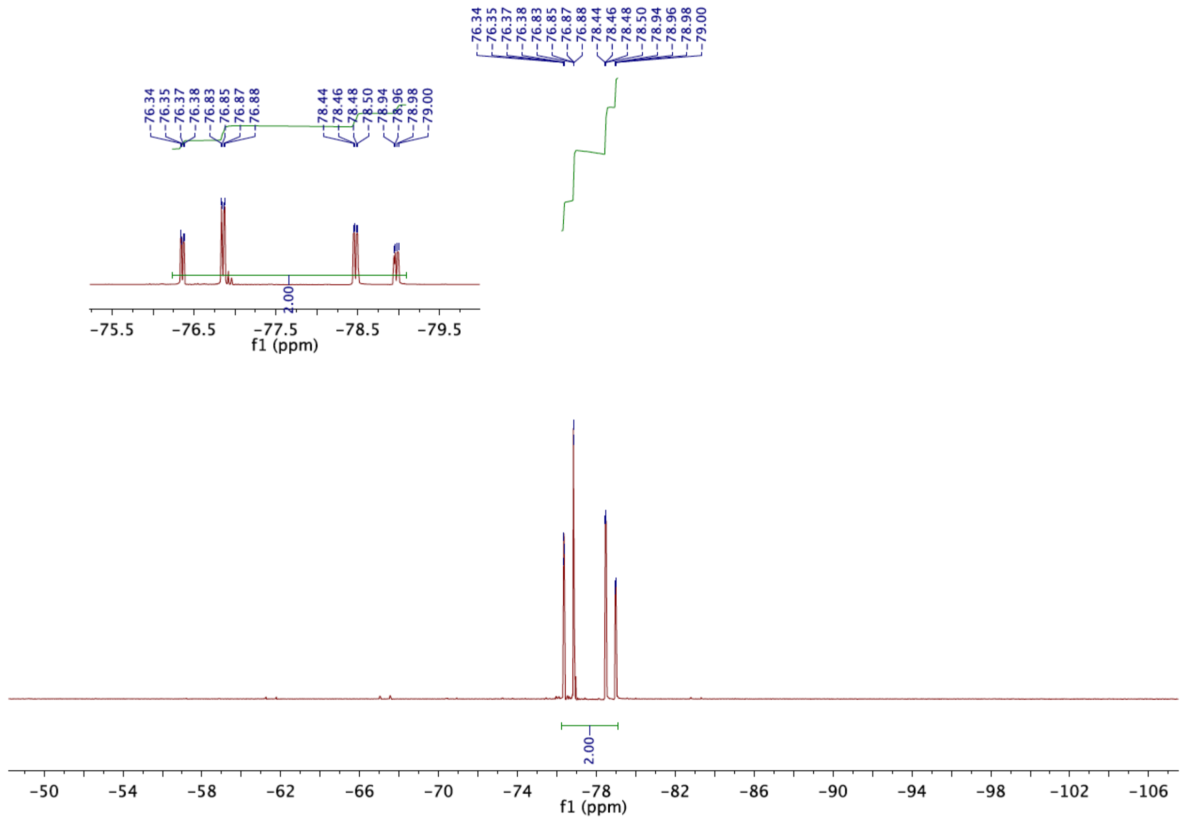

Supplementary Figure 241. ^{19}F NMR spectra of compound 66

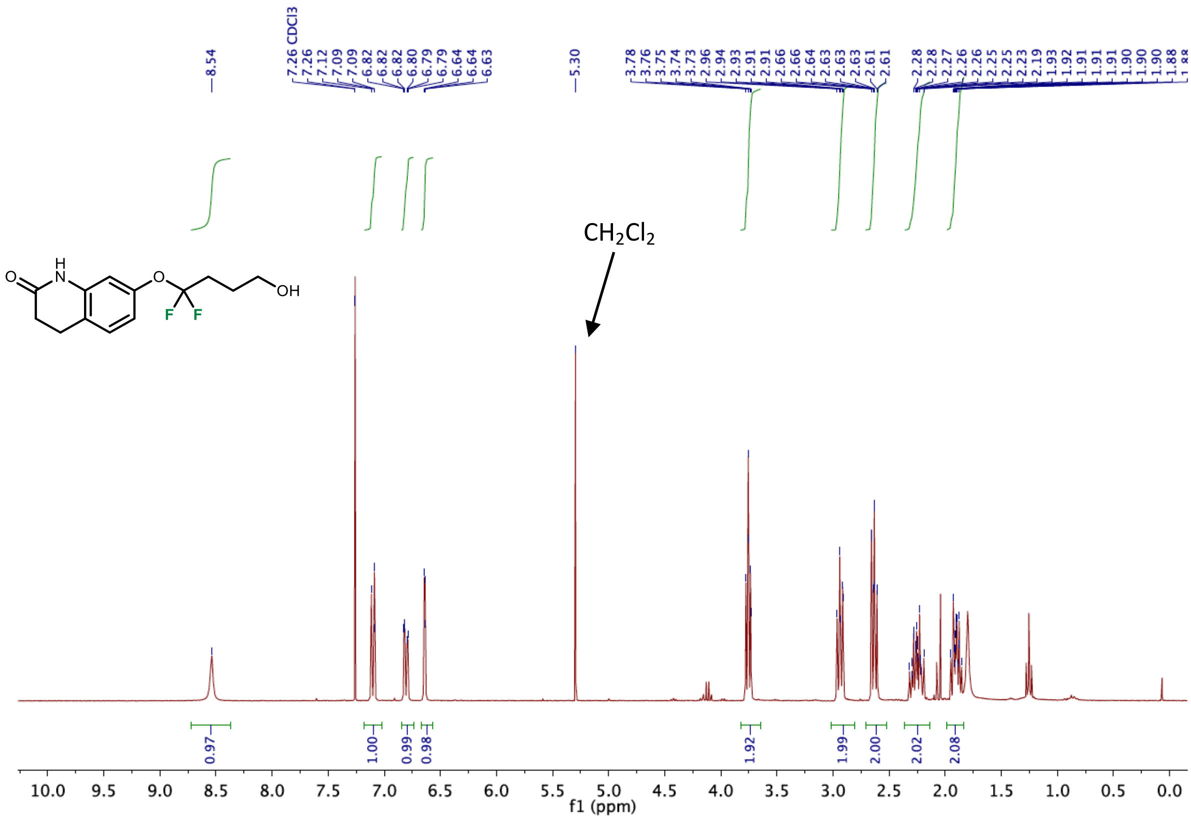

Supplementary Figure 248. ^{1}H NMR spectra of compound 67

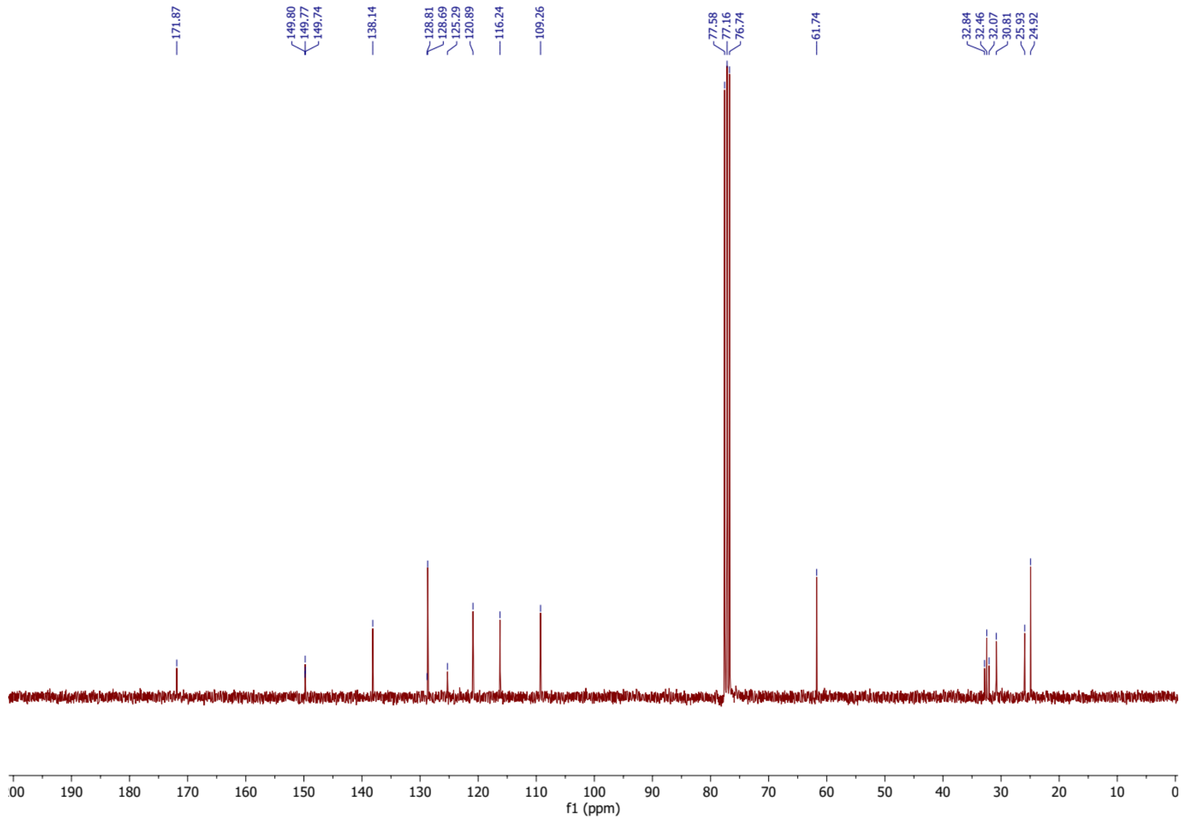

Supplementary Figure 249. ^{13}C NMR spectra of compound 67

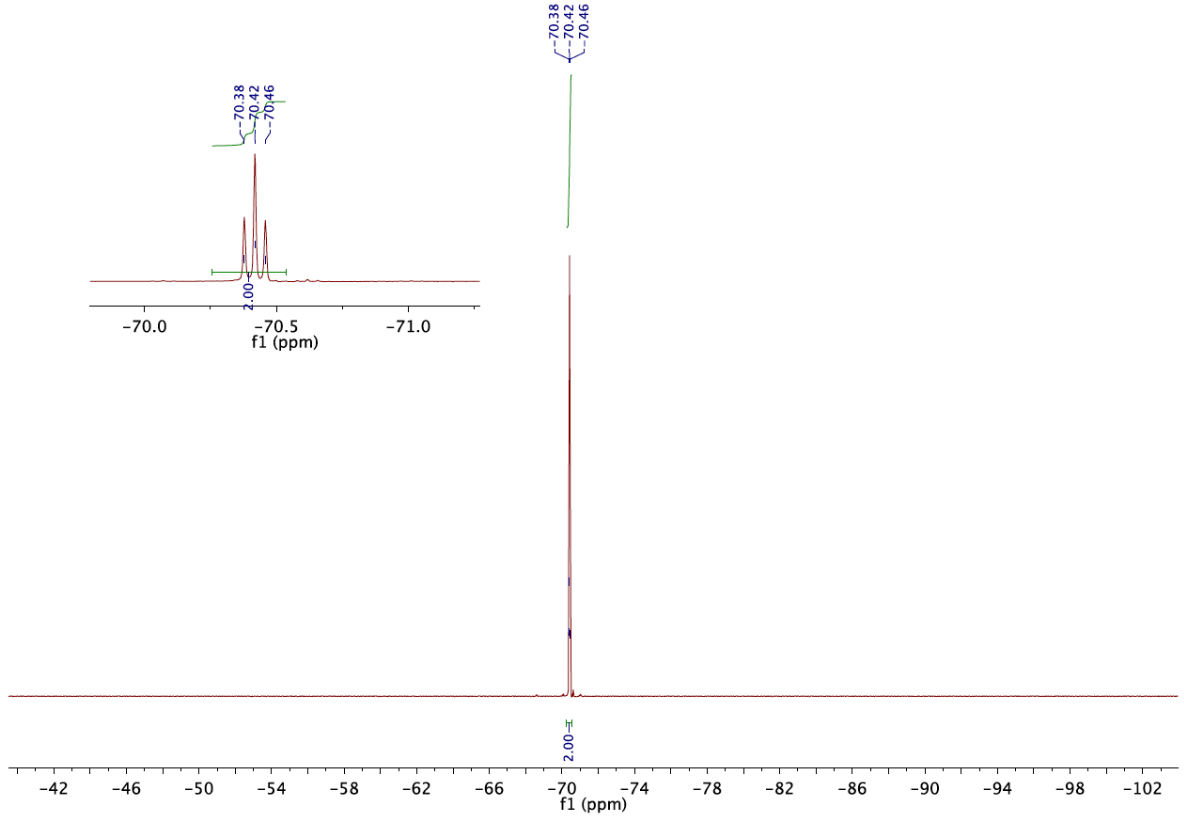

Supplementary Figure 250. ^{19}F NMR spectra of compound 67

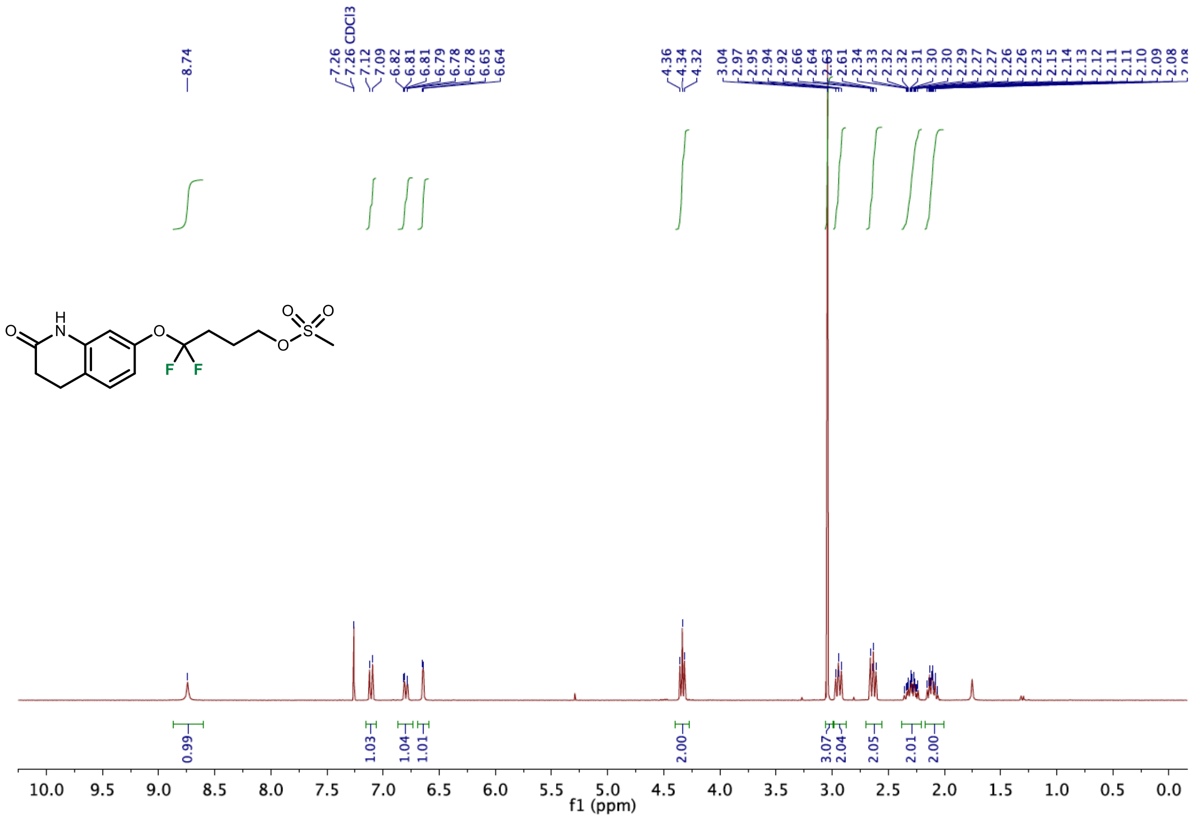

Supplementary Figure 251. ^{1}H NMR spectra of compound 68

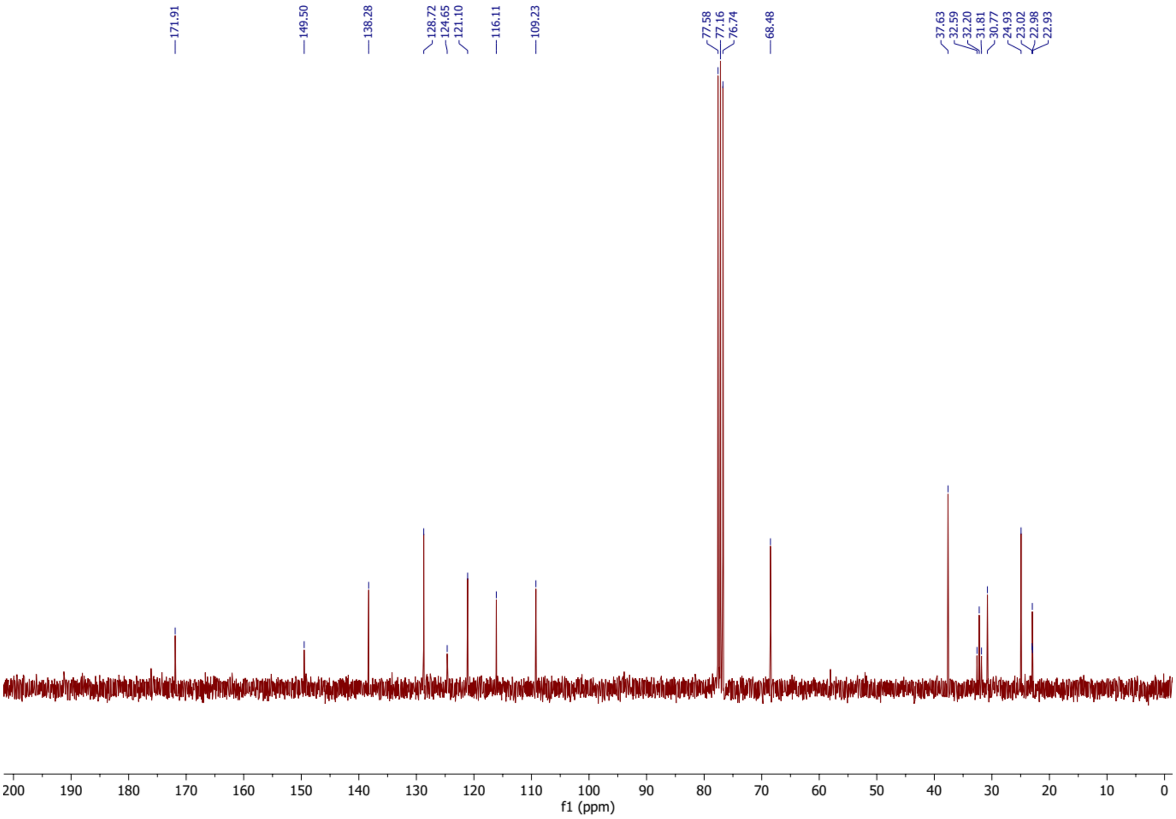

Supplementary Figure 252. ^{13}C NMR spectra of compound 68

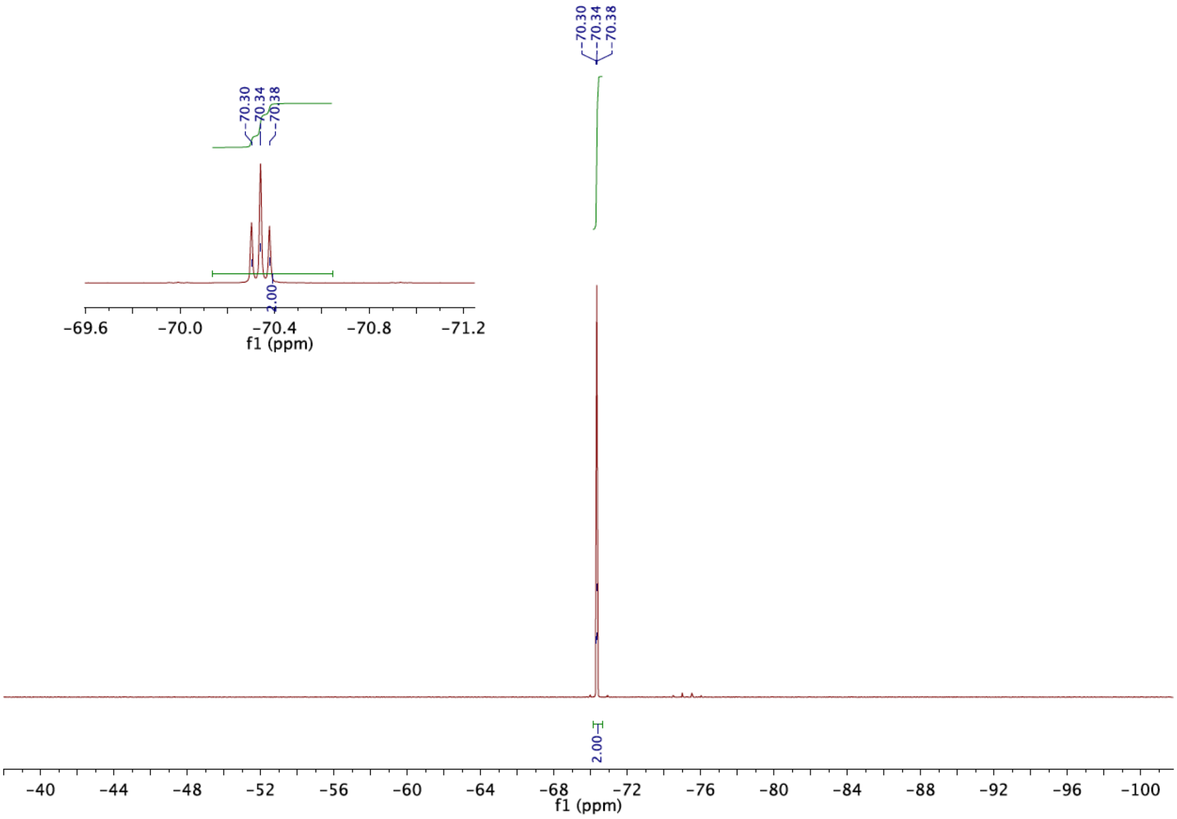

Supplementary Figure 253. ^{19}F NMR spectra of compound 68

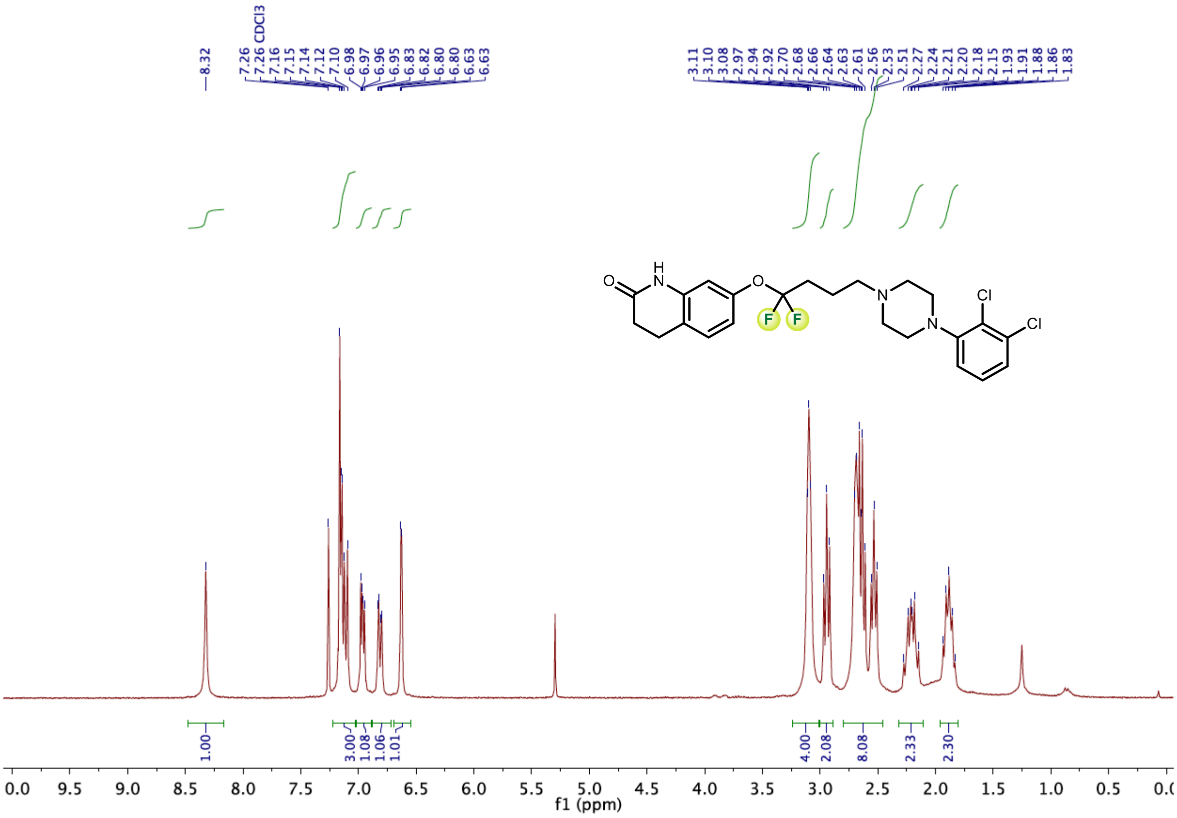

Supplementary Figure 254. ^{1}H NMR spectra of compound 70

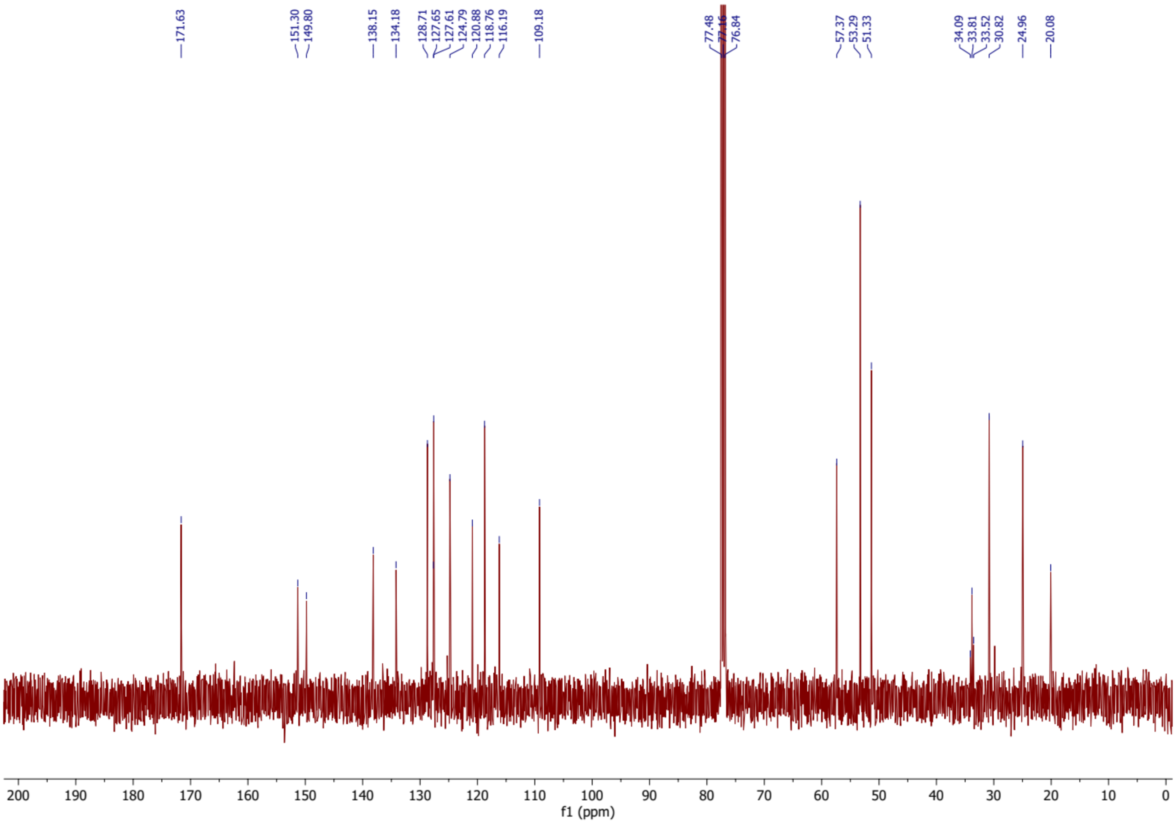

Supplementary Figure 255. ^{13}**C NMR spectra of compound 70**

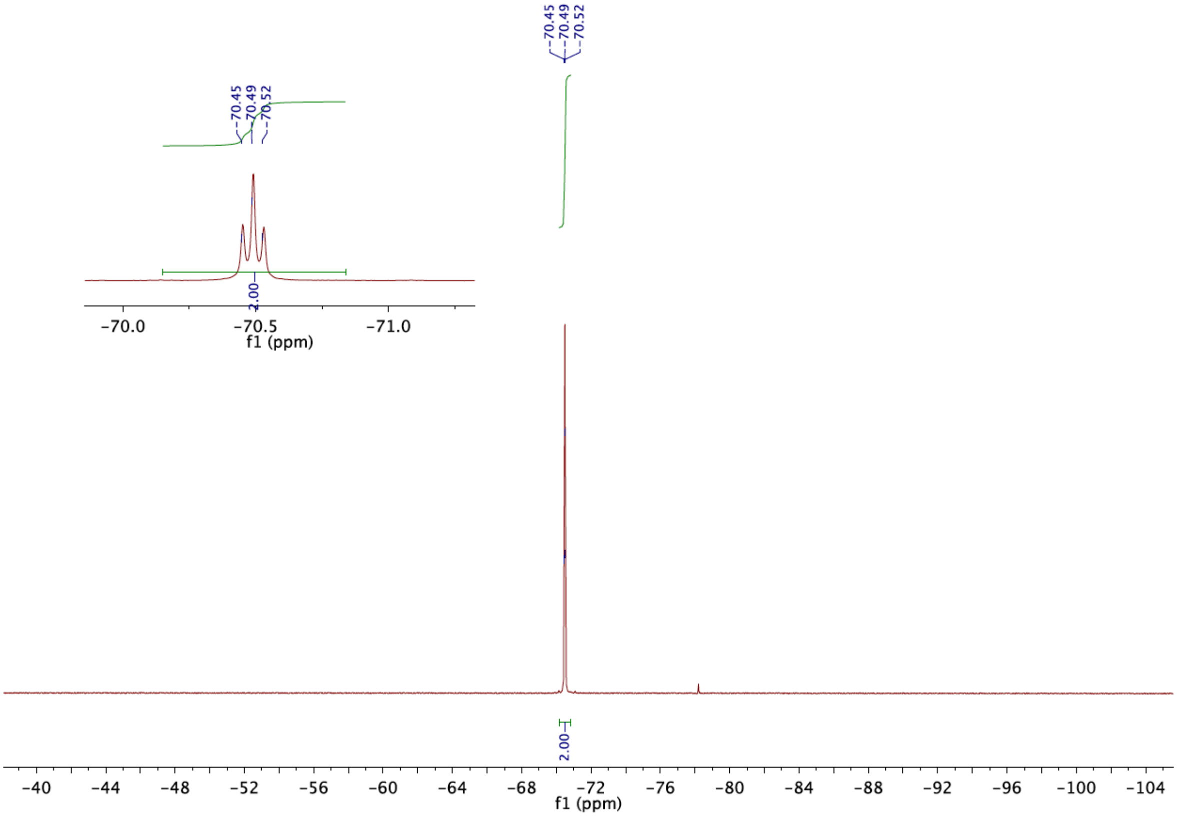

Supplementary Figure 256. ^{19}**F NMR spectra of compound 70**

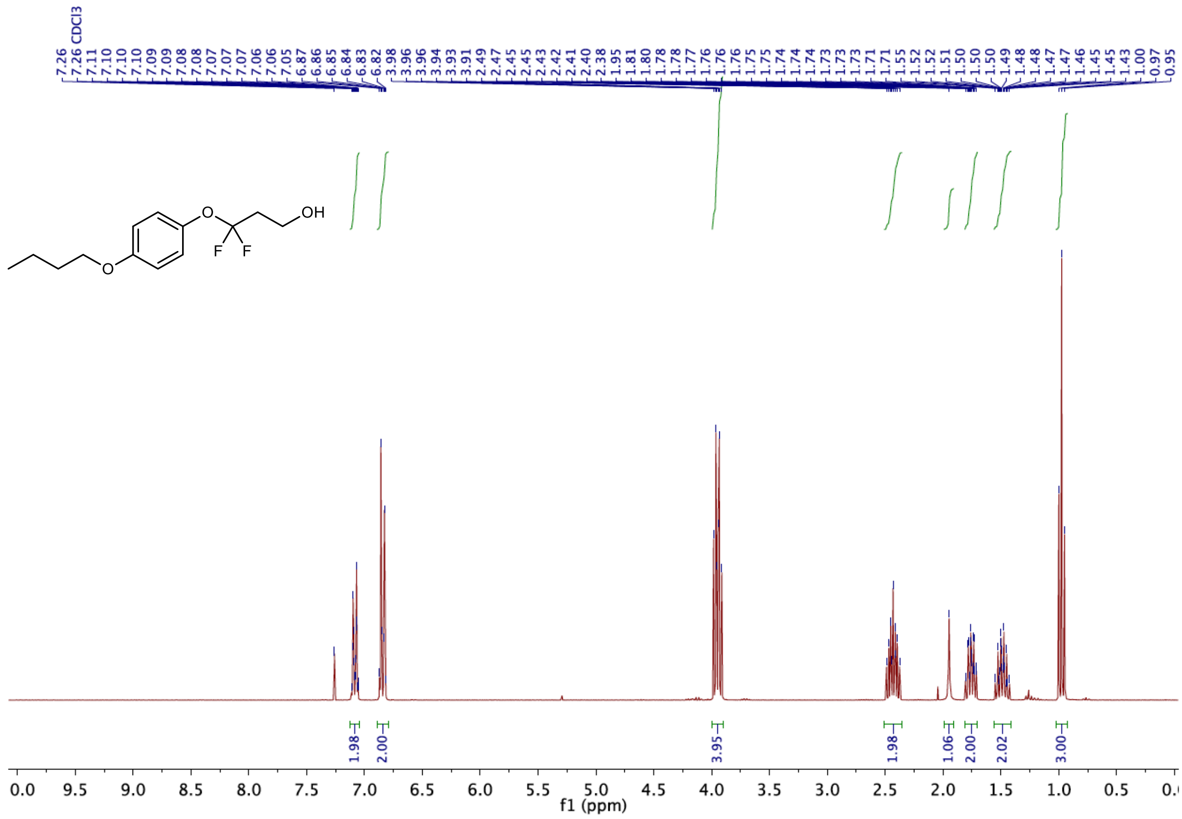

Supplementary Figure 257. ^{1}H NMR spectra of compound 71

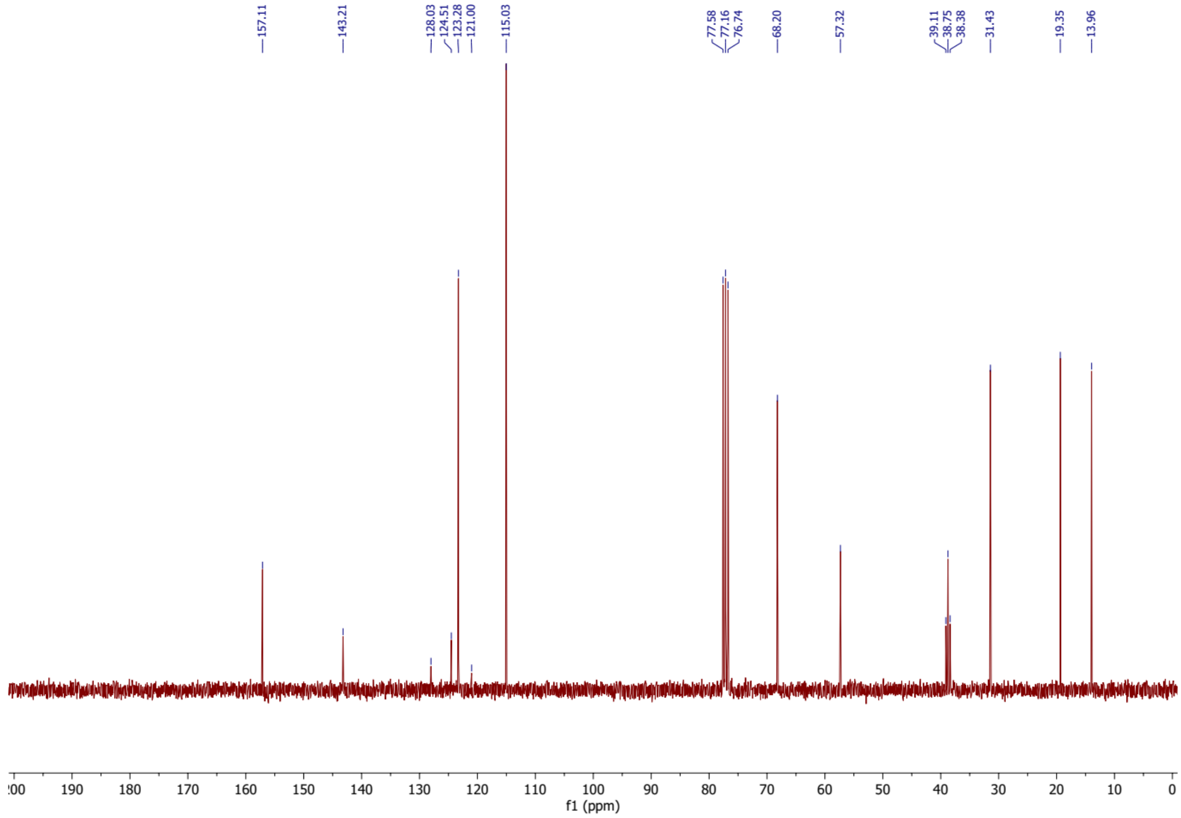

Supplementary Figure 258. ^{13}C NMR spectra of compound 71

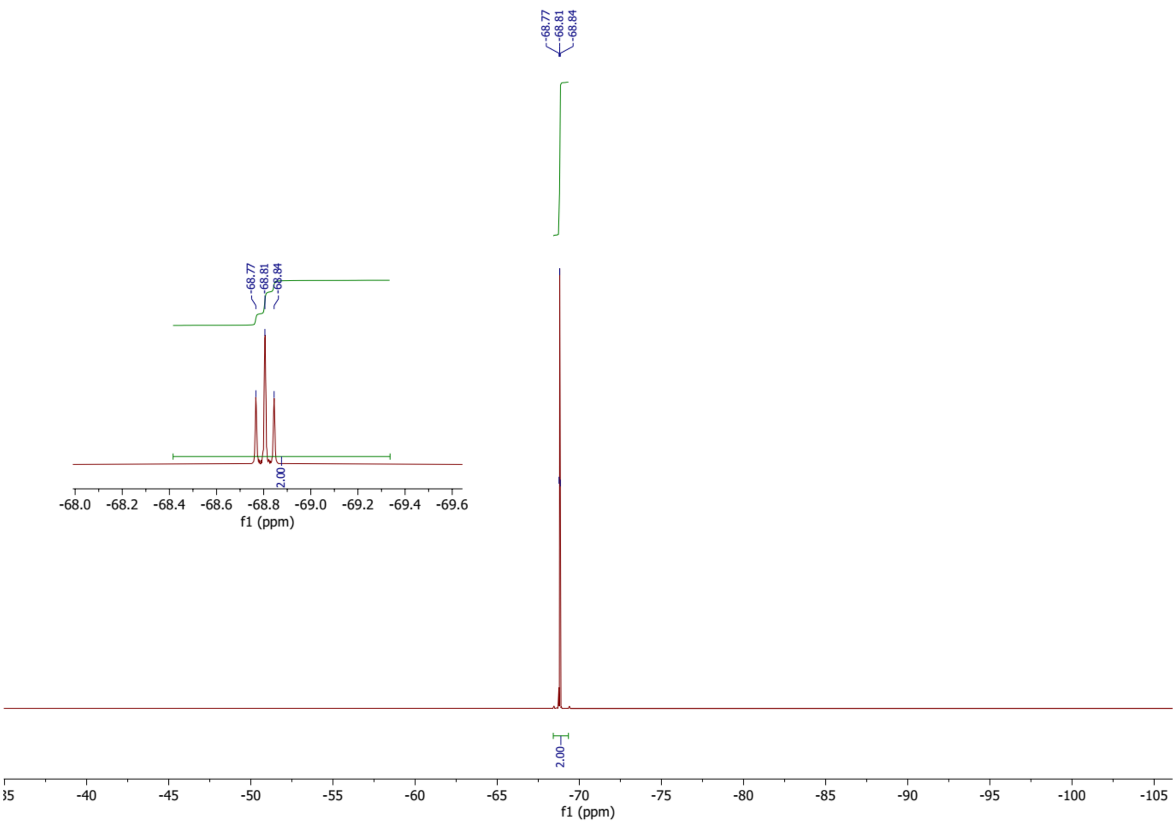

Supplementary Figure 259. ^{19}F NMR spectra of compound 71

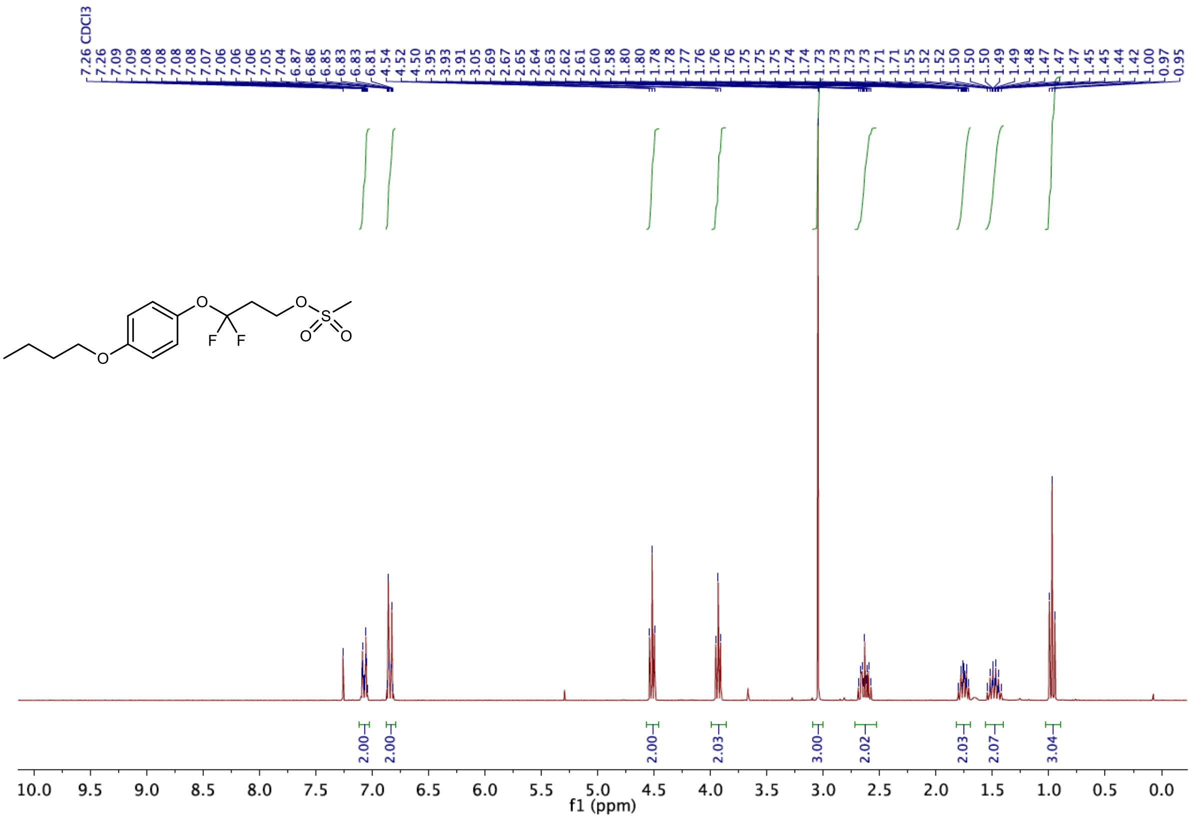

Supplementary Figure 260. ^{1}H NMR spectra of compound 72

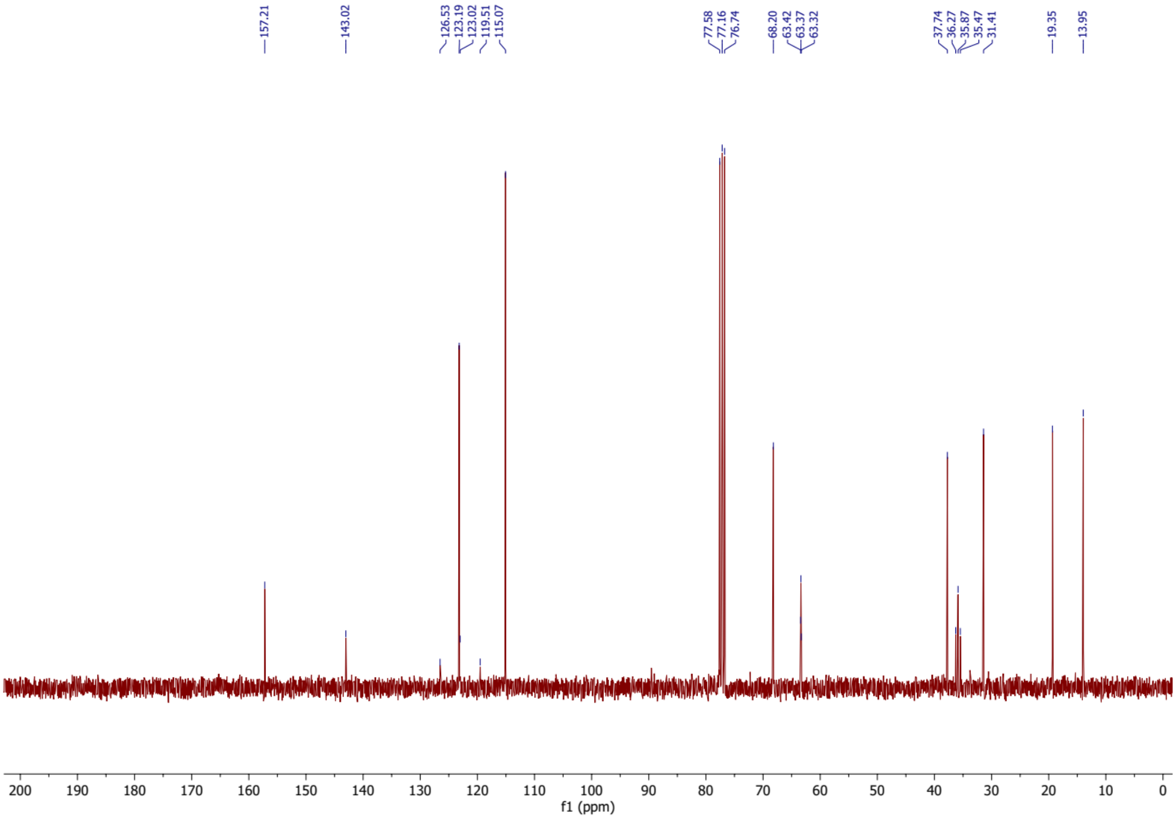

Supplementary Figure 261. ^{13}C NMR spectra of compound 72

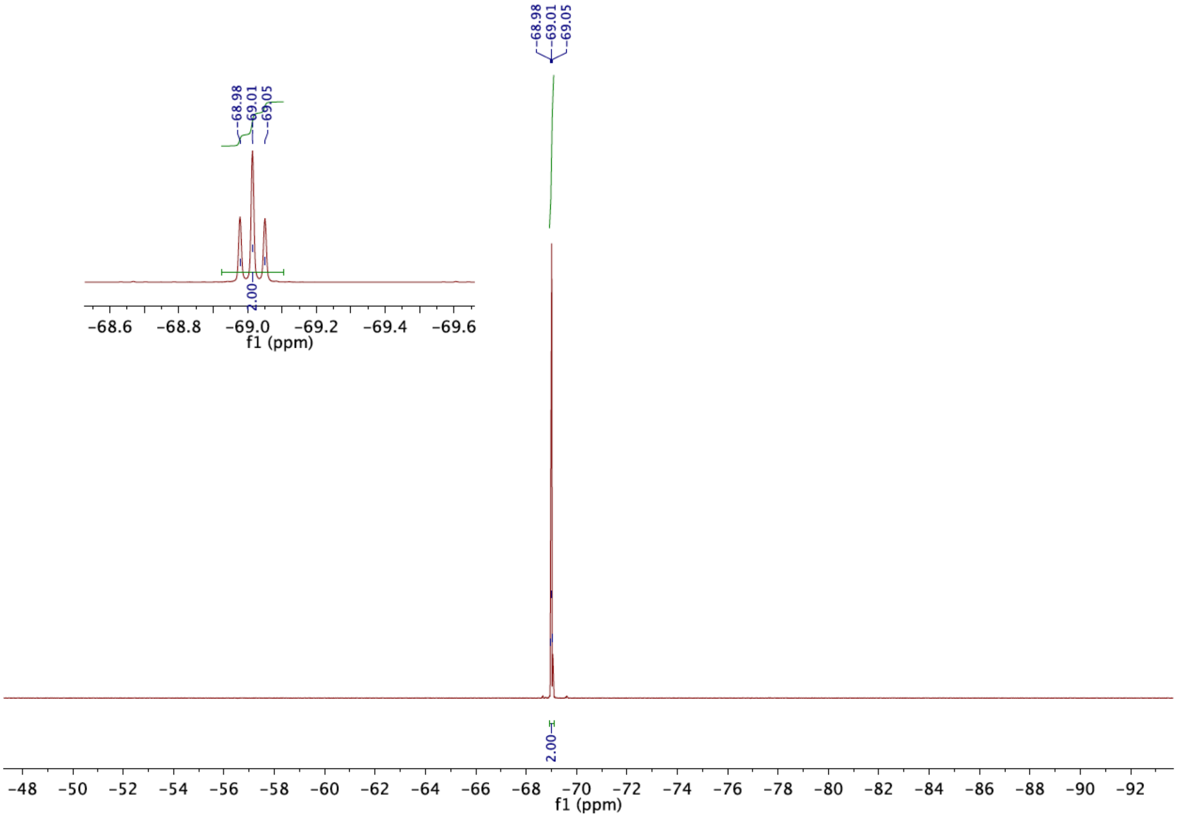

Supplementary Figure 262. ^{19}F NMR spectra of compound 72

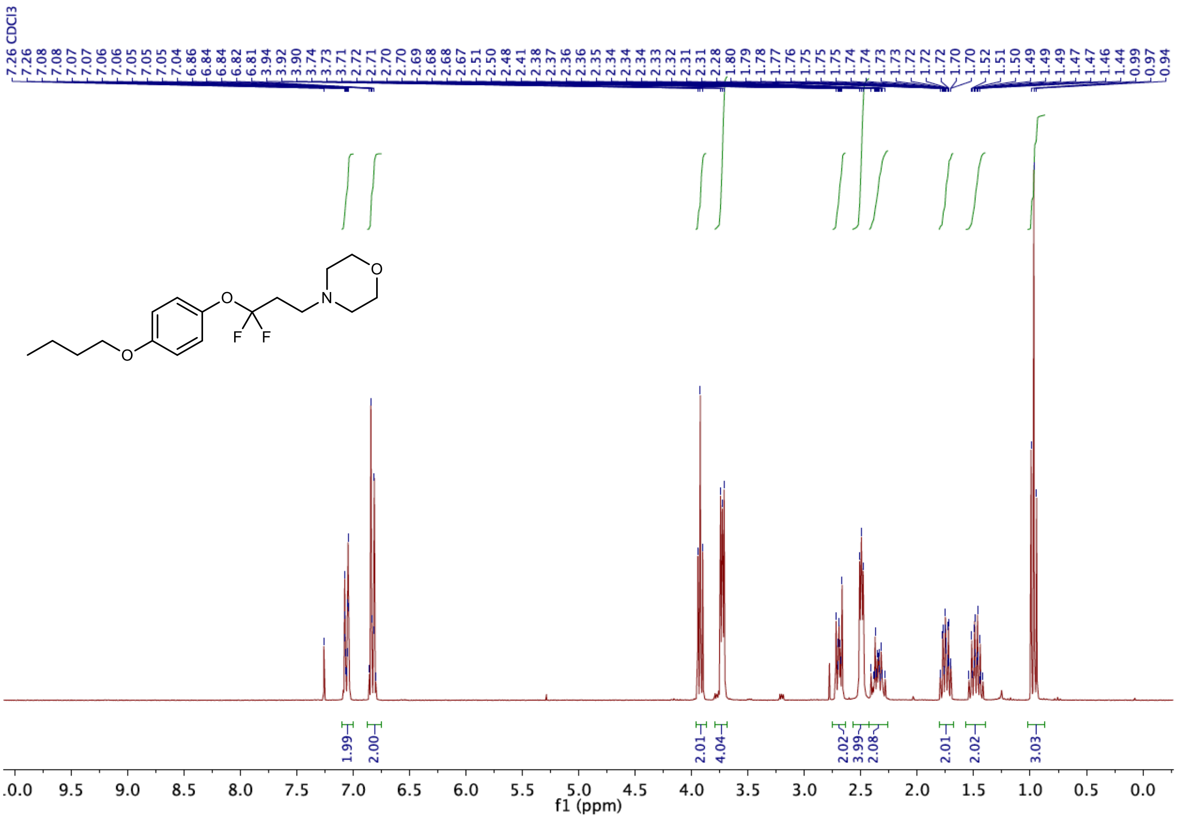

Supplementary Figure 263. ^{1}H NMR spectra of compound 73

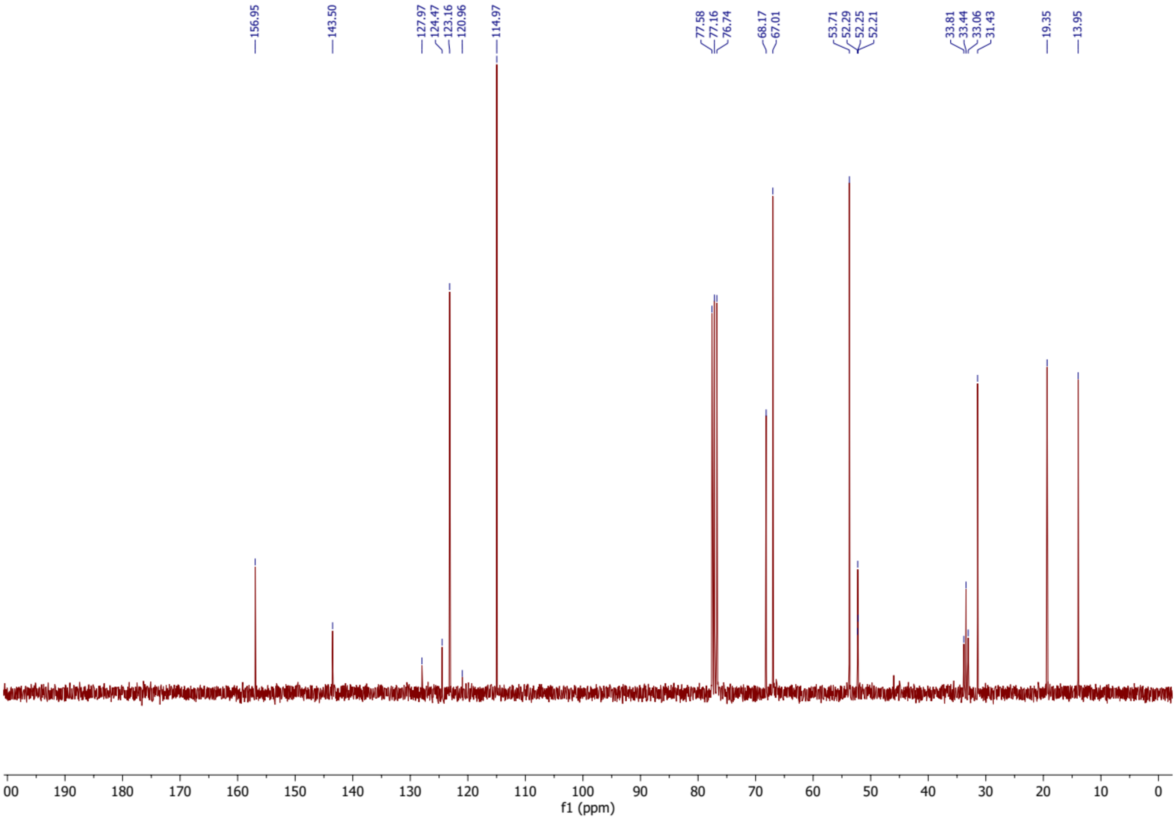

Supplementary Figure 264. ^{13}C NMR spectra of compound 73

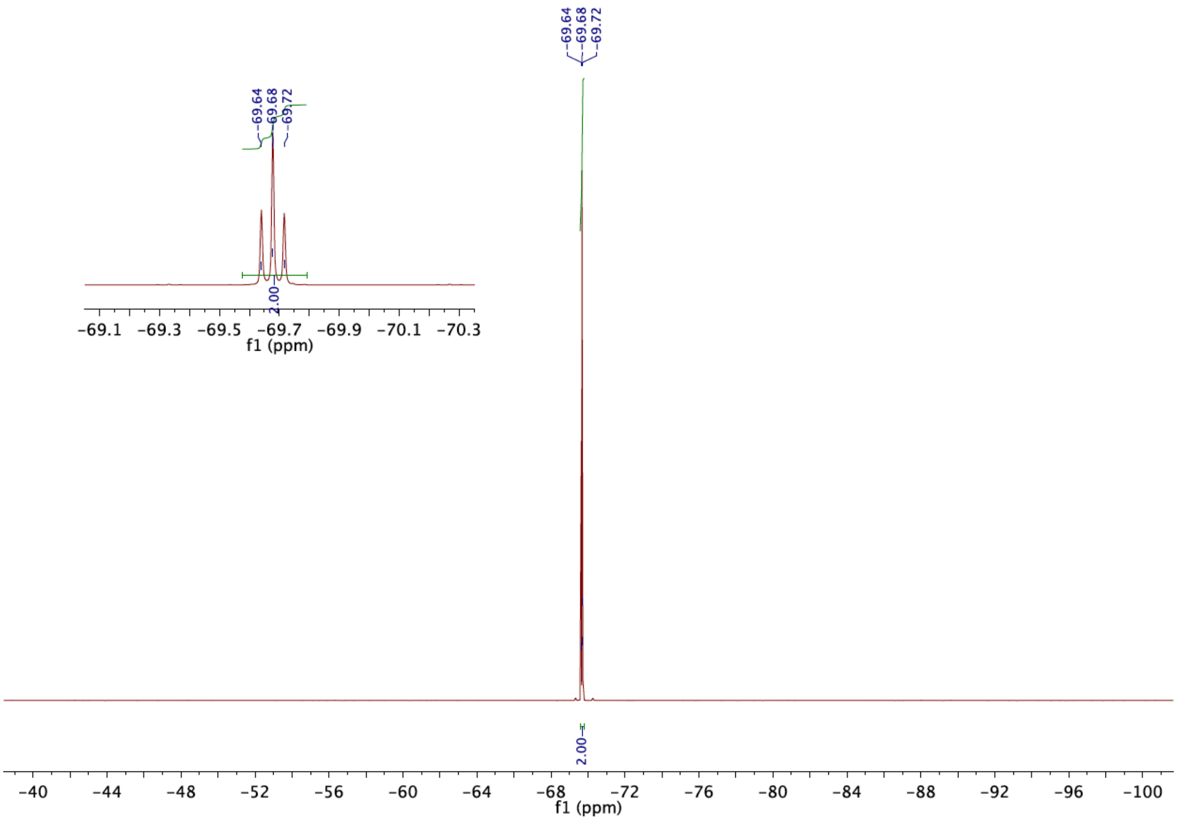

Supplementary Figure 265. ^{19}F NMR spectra of compound 73

6. HPLC spectra for compound 66

Racemic **66**

Data File D:\CHEM32\1\DATA\2008\2008002701.D
Sample Name: YF678-1-p

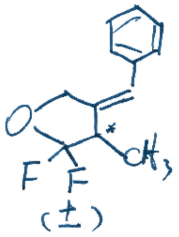

===

Acq. Operator : Analytik Seq. Line : 1
Acq. Instrument : LC5 Location : Vial 51
Injection Date : 8/27/2020 9:06:25 AM Inj : 1
Inj Volume : 0.2 µl
Acq. Method : C:\CHEM32\1\METHODS\FISCHER1.M
Last changed : 8/27/2020 9:23:09 AM by Analytik
(modified after loading)
Analysis Method : C:\CHEM32\1\METHODS\FISCHER1.M
Last changed : 8/27/2020 10:46:22 AM by Analytik
(modified after loading)
Method Info : AD-H, Heptan/EtOH 99:1, Fluss:0.5ml/min

Additional Info : Peak(s) manually integrated

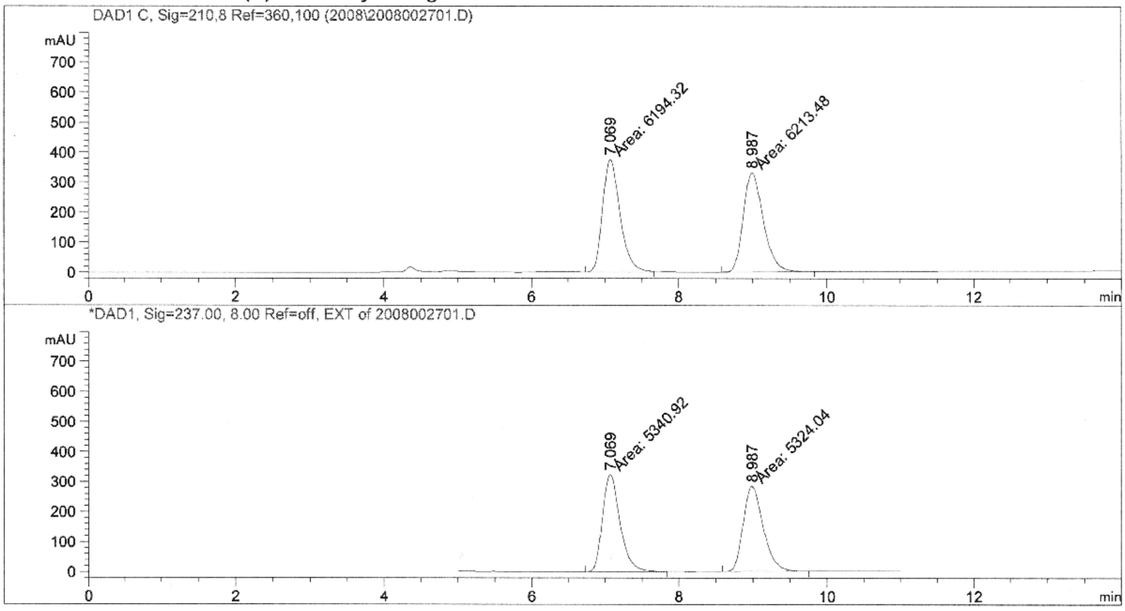

===
Area Percent Report
===

Sorted By : Signal
Multiplier : 1.0000
Dilution : 1.0000
Use Multiplier & Dilution Factor with ISTDs

Signal 1: DAD1 C, Sig=210,8 Ref=360,100

Peak #	RetTime [min]	Type	Width [min]	Area [mAU*s]	Height [mAU]	Area %
1	7.069	MF	0.2750	6194.31689	375.45319	49.9228
2	8.987	MM	0.3133	6213.48145	330.50888	50.0772

Totals : 1.24078e4 705.96207

Chiral **66**

Data File D:\CHEM32\1\DATA\2009\20090000304.D
Sample Name: YF678-2-p

===

Acq. Operator : Analytik Seq. Line : 5
Acq. Instrument : LC5 Location : Vial 31
Injection Date : 9/3/2020 1:36:55 PM Inj : 1
Inj Volume : 0.2 µl
Acq. Method : C:\CHEM32\1\METHODS\FISCHER1.M
Last changed : 9/3/2020 12:12:16 PM by Analytik
Analysis Method : C:\CHEM32\1\METHODS\FISCHER1.M
Last changed : 9/3/2020 3:37:14 PM by Analytik
(modified after loading)
Method Info : AD-H1, Heptan/EtOH 99:1, Fluss:0.5ml/min

Additional Info : Peak(s) manually integrated

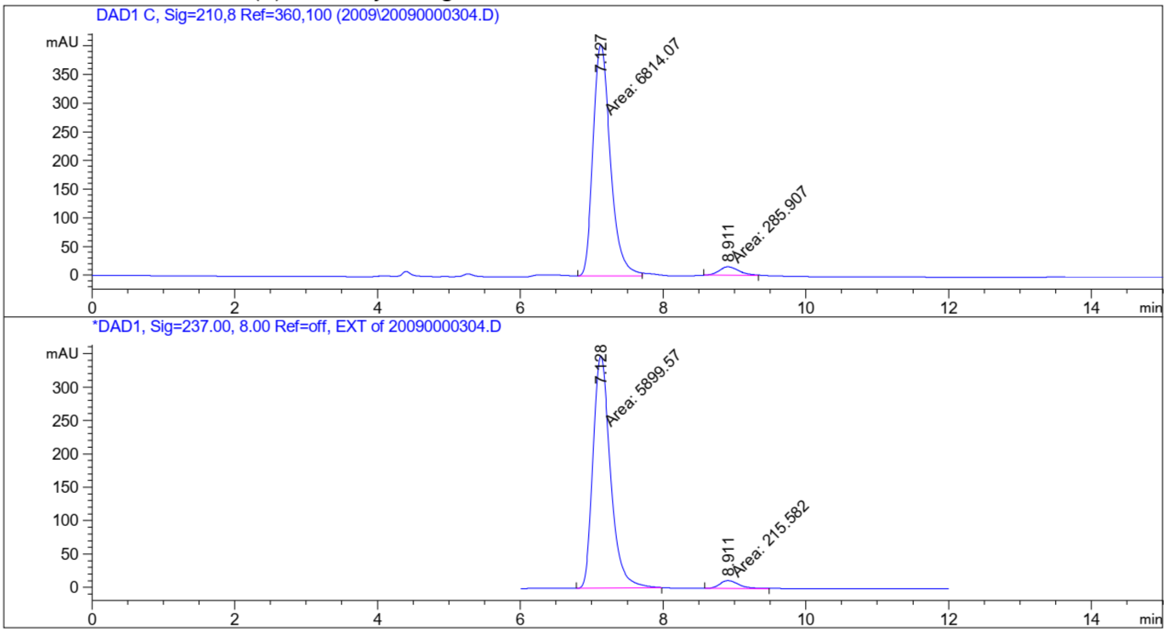

===
Area Percent Report
===

Sorted By : Signal
Multiplier : 1.0000
Dilution : 1.0000
Use Multiplier & Dilution Factor with ISTDs

Signal 1: DAD1 C, Sig=210,8 Ref=360,100

Peak #	RetTime [min]	Type	Width [min]	Area [mAU*s]	Height [mAU]	Area %
1	7.127	MF	0.2816	6814.06738	403.29559	95.9731
2	8.911	MM	0.3216	285.90665	14.81517	4.0269

Totals : 7099.97403 418.11076

Signal 2: DAD1, Sig=237.00, 8.00 Ref=off, EXT
Signal has been modified after loading from rawdata file!

Peak #	RetTime [min]	Type	Width [min]	Area [mAU*s]	Height [mAU]	Area %
1	7.128	MM	0.2831	5899.57080	347.26468	96.4746
2	8.911	MM	0.3062	215.58205	11.73613	3.5254

Totals : 6115.15285 359.00081

===
*** End of Report ***
